# Chemical Synthesis of Truncated Capsular Oligosaccharide of Serotypes 6C and 6D of *Streptococcus pneumoniae* with Their Immunological Studies

**DOI:** 10.1021/acsinfecdis.4c00147

**Published:** 2024-05-21

**Authors:** Ravinder Mettu, Yang-Yu Cheng, Hanmanth Reddy Vulupala, Yu-Hsuan Lih, Chiang-Yun Chen, Mei-Hua Hsu, Hong-Jay Lo, Kuo-Shiang Liao, Cheng-Hsun Chiu, Chung-Yi Wu

**Affiliations:** † Genomics Research Center, Academia Sinica, 128 Academia Road, Section 2, Nankang, Taipei 11529, Taiwan; ‡ Institute of Biochemistry and Molecular Biology, National Yang Ming Chiao Tung University, No. 155, Section 2, Linong Street, Taipei 112304, Taiwan; § Molecular Infectious Disease Research Center, Chang Gung Memorial Hospital, Chang Gung University College of Medicine, 259 Wenhua First Road, Guishan, Taoyuan 33302, Taiwan

**Keywords:** Streptococcus pneumoniae, pneumococcal conjugate vaccine, capsular polysaccharide, cross-reactive antibody

## Abstract

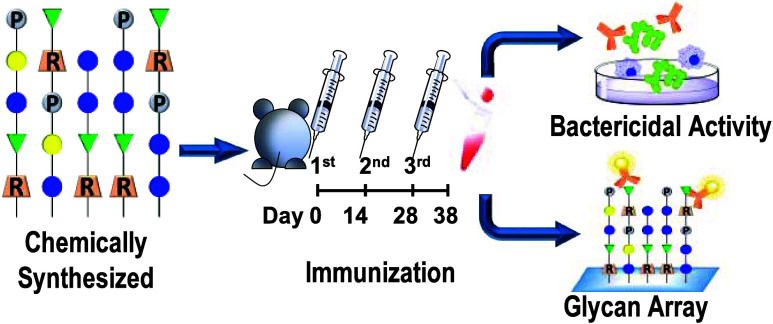

Serotypes 6C and 6D of *Streptococcus pneumoniae* are two major variants that cause invasive pneumococcal disease (IPD) in serogroup 6 alongside serotypes 6A and 6B. Since the introduction of the pneumococcal conjugate vaccines PCV7 and PCV13, the number of cases of IPD caused by pneumococcus in children and the elderly population has greatly decreased. However, with the widespread use of vaccines, a replacement effect has recently been observed among different serotypes and lowered the effectiveness of the vaccines. To investigate protection against the original serotypes and to explore protection against variants and replacement serotypes, we created a library of oligosaccharide fragments derived from the repeating units of the capsular polysaccharides of serotypes 6A, 6B, 6C, and 6D through chemical synthesis. The library includes nine pseudosaccharides with or without exposed terminal phosphate groups and four pseudotetrasaccharides bridged by phosphate groups. Six carbohydrate antigens related to 6C and 6D were prepared as glycoprotein vaccines for immunogenicity studies. Two 6A and two 6B glycoconjugate vaccines from previous studies were included in immunogenicity studies. We found that the conjugates containing four phosphate-bridged pseudotetrasaccharides were able to induce good immune antibodies and cross-immunogenicity by showing superior activity and broad cross-protective activity in OPKA bactericidal experiments.

According to the World Health Organization (2019), pneumonia and lower respiratory infections are the fourth leading cause of death and caused 740,180 deaths in children under 5 years of age.^1−3^*Streptococcus pneumoniae* (SPn) is one of the leading causes of invasive pneumonia disease (IPD) and can cause other serious infections including meningitis, peritonitis, and bacteremia.^4−6^ Moreover, pneumococcus that colonizes the human nasopharynx can easily cause community transmission, affecting children, the elderlies, and immunocompromised adults.^7−10^

*S. pneumoniae* can resist the host immune system through its outermost layer capsular polysaccharide (CPS), which is a major virulence factor, and the CPS can also regulate charge environments to promote nasopharyngeal colonization.^11−13^ Based on the differences in CPS, at least 100 different serotypes can be identified.^14^ To prevent pneumococcal disease, a series of pneumococcal conjugate vaccines (PCVs) have been developed using CPS as the specific antigen-binding carrier protein. Since PCV7 (4, 6B, 9 V, 14, 18C, 19F, 23F, Prevenar; Pfizer) was approved in 2000 followed by PCV10 (PCV7+ 1, 5, 7F, Synflorix; GlaxoSmithKline) in 2008 and PCV13 (PCV10+ 3, 6A, 19A, Prevenar 13; Pfizer) in 2010,^14−16^ the infection rate of IPD had significantly reduced, successfully achieving herd immunity to prevent a large number of community transmissions.^15,17−19^ However, large increases in nonvaccine serotype cases have been observed in some countries (Europe, UK, and USA),^20−22^ suggesting a replacement effect caused by the dominance of nonvaccine serotypes in the nasopharynx after vaccine serotypes are suppressed.^7,23^ In addition, pneumococcus will also change its CPS through gene mutation, recombination, and horizontal gene transfer with other nasopharyngeal pathogens to achieve immune escape, decreasing vaccine effectiveness.^23−27^ Therefore, the need for continuous improvement of vaccines remains critical.

*S. pneumoniae* serogroup 6 (SPn6) is a common cause of IPD, and the serotype replacement effect has occurred. Early serotype 6 only separated into serotype 6A (ST6A) and serotype 6B (ST6B), both of which have a CPS composed of galactose–glucose–rhamnose–ribitol. Due to the difference in the *wciP* gene, the rhamnose–ribitol was 1 → 3 linkage in ST6A and 1 → 4 linkage in ST6B.^28^ After 2007, two new serotypes, 6C (ST6C) and 6D (ST6D), were identified and have led to an increase in IPD cases;^29,30^ however, their CPS antigens are not yet included in any of the available vaccines. Serotypes 6C and 6D are similar to 6A and 6B but have different CPS compositions and linkages. The *wciN* of ST6C (glucose–glucose–rhamnose–ribitol) is different, allowing a glucose residue in its CPS instead of the galactose residue in ST6A. In contrast, ST6D is similar to ST6C but has a different *wciP*, resulting in a 1 → 4 linkage of rhamnose–ribitol (Figure 1).^31^

**Figure 1:**
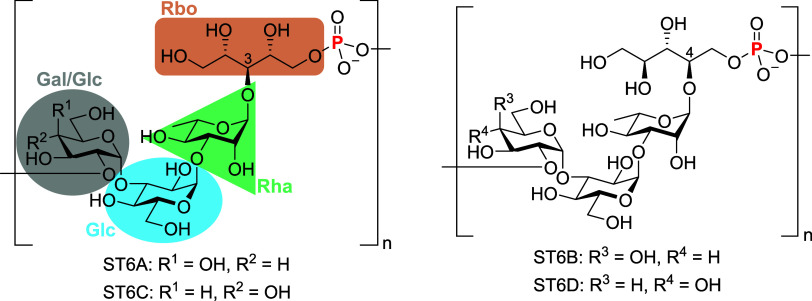
Repeating units of *S. pneumoniae* serotypes 6A, 6B, 6C, and 6D. Gal, galactose; Glc, glucose; Rha, rhamnose; and Rbo, ribitol.

Although PCV13 has been shown to reduce IPD of vaccine serotypes, ST6C and ST6D still cause carriage and IPD in some countries.^32−34^ Our strategy is to synthesize different CPS fragments of serogroup 6 through chemical methods, attempting to explore the minimum epitope that can induce cross-reactive antibodies against ST6A, ST6B, ST6C, and ST6D. Identifying the minimal epitope can help avoid redundant epitope preparation, resulting in significant cost savings during the manufacturing process. Moreover, the synthetic oligosaccharides offer a well-defined structure and stable purity, which supply significant advantages over CPS fragment mixtures obtained from natural sources in terms of vaccine development and the improvement of vaccine.^35,36^ Although in studies, various lengths of oligosaccharides related to ST6A and ST6B have been synthesized and their immunological properties were tested,^37−48^ none of them studied the role of the phosphate group and complete immunological studies for serogroup 6. Further, research on the synthesis of oligosaccharide and immemorial studies of ST6C^45^ and ST6D have been relatively limited to the best of our knowledge. Therefore, in order to find a suitable vaccine candidate against serogroup 6, we designed and synthesized the oligosaccharides of ST6A-6D with a phosphate group at various positions.

Herein, we report our studies of synthetic conjugate vaccines for serogroup 6 pneumococci. Chemical synthesis was employed to prepare various CPS fragments of ST6C and ST6D, including pseudotetrasaccharides with or without phosphate group exposure and with phosphate bridging as immune antigens. We conducted coevaluation with CPS and vaccines of ST6A and ST6B from a previous study and found that the position of the phosphate group and the phosphate bridging structure may enhance vaccine immunogenicity and cross-reactivity. Moreover, the use of homogeneous antigens synthesized chemically may reduce the production of non-neutralizing antibody. These findings are of great significance for advancing the research and development of the immunogenicity and cross-reactivity of SPn6 vaccines.

## Results

**Figure 2:**
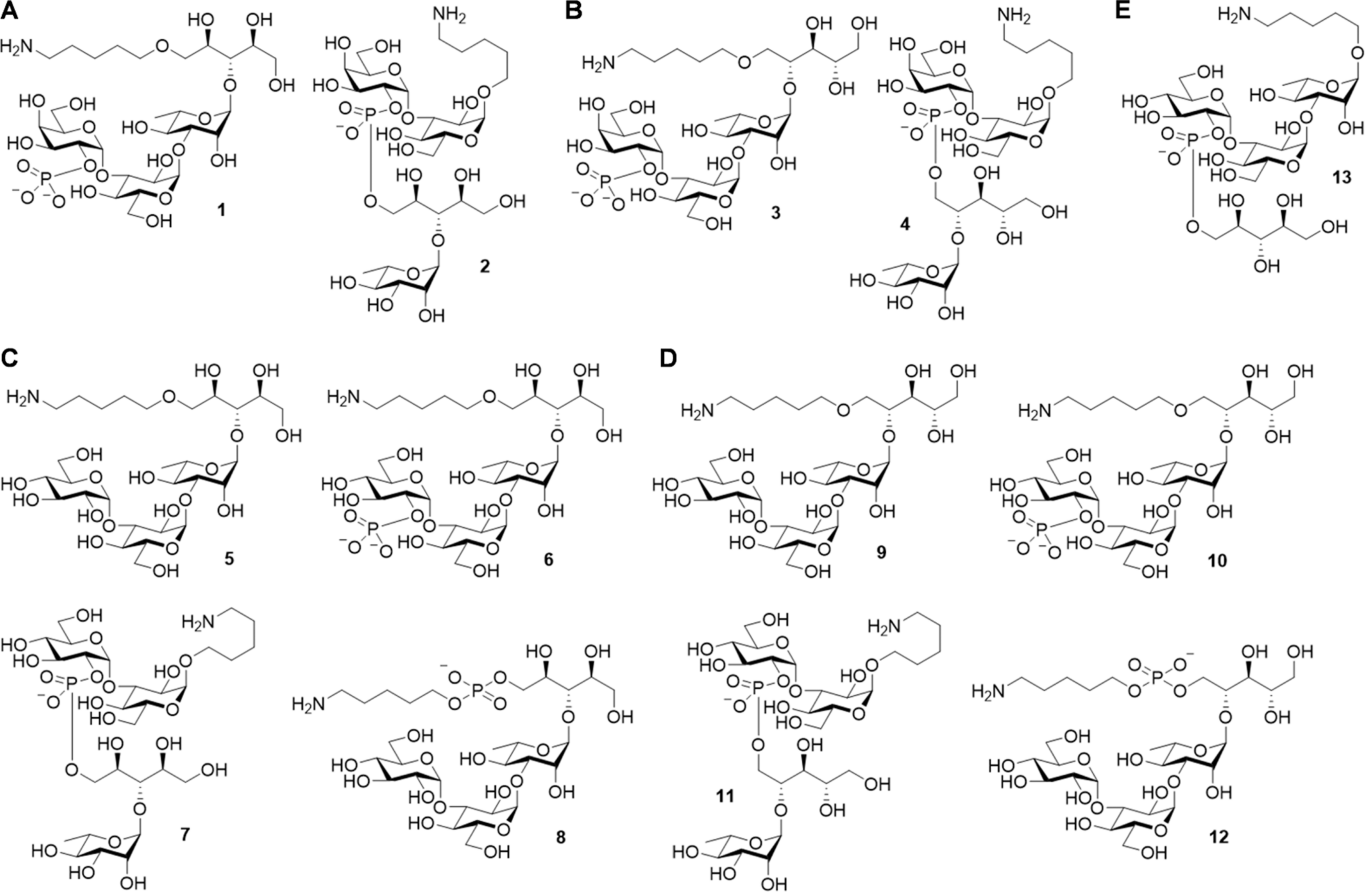
Synthesized pneumococcal capsular oligosaccharide fragments. (A) ST6A oligosaccharide antigens (**1**–**2**) and (B) ST6B oligosaccharide antigens (**3**–**4**) synthesized previously. (C) ST6C oligosaccharide antigens (**5**–**8**) synthesized in this work. (D) ST6D oligosaccharide antigens (**9**–**12**) synthesized in this work. (E) ST6C and ST6D common trisaccharide antigen (**13**) synthesized in this work.

In the investigation of pneumococcal vaccines, we synthesized four fragmentary capsular oligosaccharides for both ST6C (**5**–**8**; Figure 2C) and ST6D (**9**–**12**; Figure 2D) and one shared fragment (**13**; Figure 2E) to explore potential vaccine candidates for pneumococci ST6C and ST6D. Moreover, our previously synthesized ST6A and ST6B vaccines (**1**–**4**; Figure 2A,2B) were included in this study.^48^ These oligosaccharide antigens were conjugated to carrier proteins for immunological evaluation in a mouse model, and the mouse antisera were analyzed for glycan binding intensity and bactericidal activity in cross-reactivity.

### Synthesis of Pseudotetrasaccharides with or without Terminal Phosphate

**Scheme 1:**
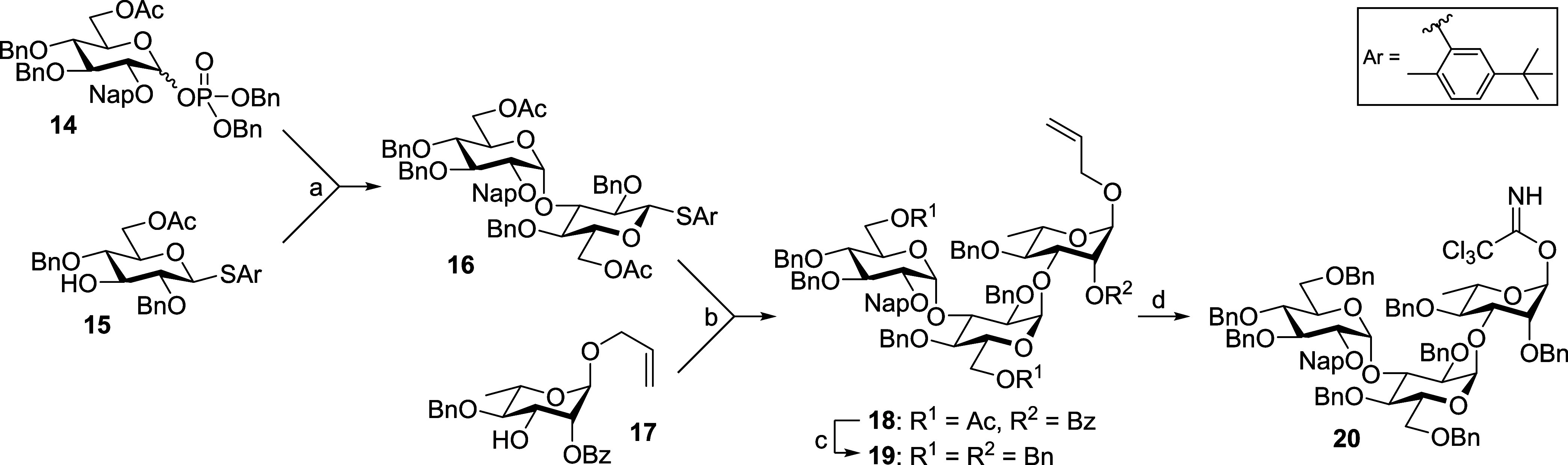
Synthesis of Main Trisaccharide Donor **20** Reagents and conditions: (a) TMSOTf, 4 Å MS, CH_2_Cl_2_, −40 °C, 4 h, and 75%; (b) NIS, TfOH, 4 Å MS, CH_2_Cl_2_, −30 °C, 4 h, and 60%; (c) (i) NaOMe, MeOH, CH_2_Cl_2_, rt, and 24 h; (ii) BnBr, DMF, 0 °C to rt, 4 h, and 75% over two steps; and (d) (i) PdCl_2_, MeOH, CH_2_Cl_2_, rt, 4 h, and 85% and (ii) CCl_3_CN, Cs_2_CO_3_, CH_2_Cl_2_, rt, 16 h, and 76%.

To synthesize the desired pseudotetrasaccharides, we first built the skeleton Glc-(α3)-Glc-(α3)-Rha trisaccharide **20** (Scheme 1). The trisaccharide was constructed through sequential glycosylation from the nonreducing end. In more detail, disaccharide **16** was prepared by 6-OAc-assisted α-glycosylation^49,50^ of glucosyl donor **14** with glucosyl acceptor **15**. The disaccharide donor **16** with preinstalled 6-OAc at the reducing end was then glycosylated with rhamnosyl acceptor **17** to provide main trisaccharide **18**. To improve the reactivity as a glycosyl donor, the ester protecting groups were replaced with benzyl groups, making the trisaccharide more “armed”. Subsequent introduction of anomeric imidate provided trisaccharide **20** for later constructions of pseudotetrasaccharides.

**Scheme 2:**
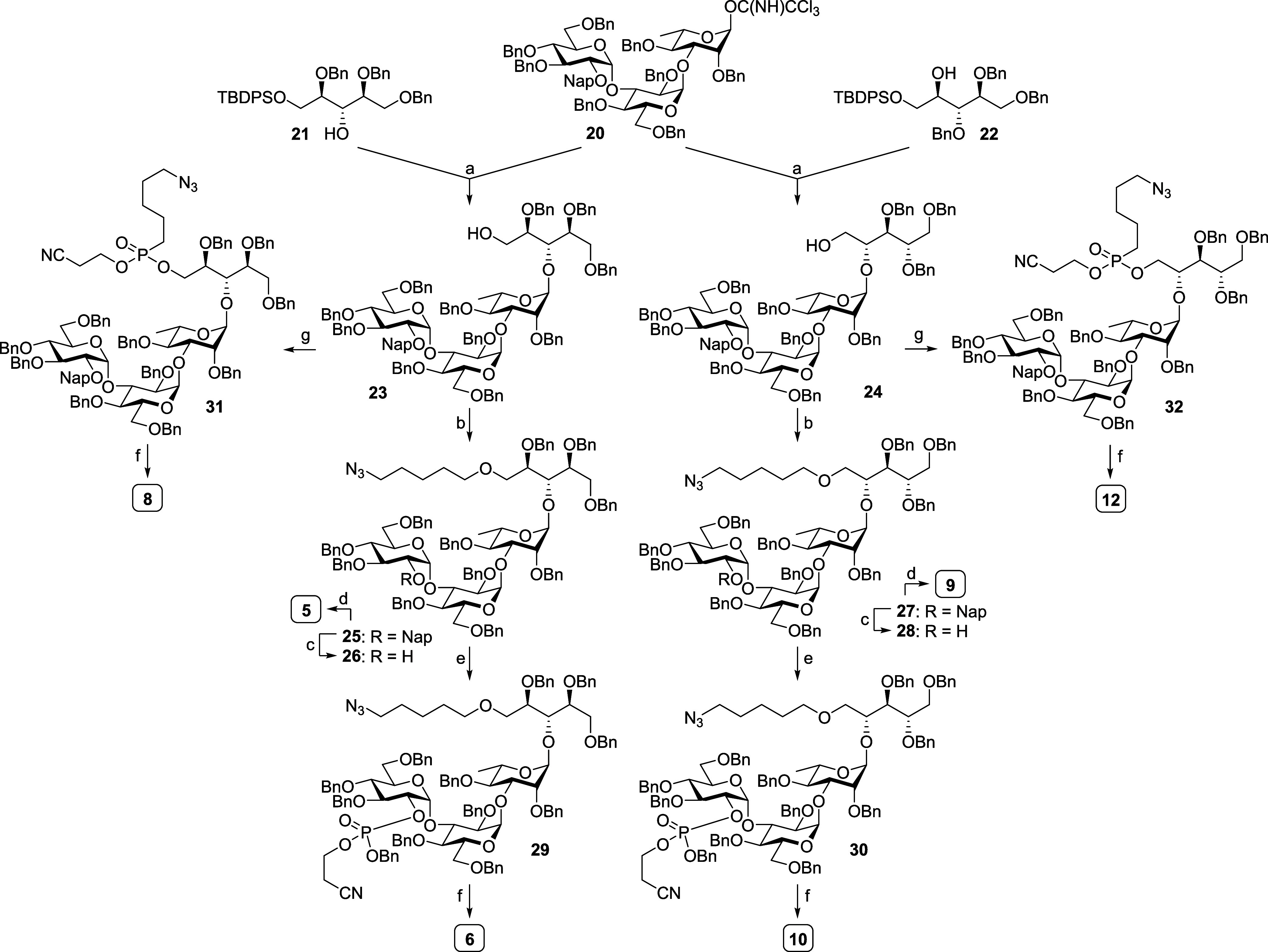
Synthesis of Pseudotetrasaccharides **5**, **6**, **8**, **9**, **10**, and **12** Reagents and conditions: (a) (i) TMSOTf, 4 Å MS, CH_2_Cl_2_, −30 °C, 1 h, 61% toward **23**, and 67% for **24**; (ii) TBAF, THF, rt, 5 h, 95% for **23**; and 94% for **24**; (b) N_3_CH_2_(CH_2_)_3_CH_2_OMs, NaH, DMSO, rt, 20 h, 92% for **25**, and 96% for **27**; (c) DDQ, CH_2_Cl_2_, phosphate buffer pH 7, 0 °C, 2.5 h, 45% for **26**, and 49% for **28**; (d) Pd(OH)_2_, H_2_, MeOH, H_2_O, AcOH, 36 h, 84% for **5**, and 78% for **9**; (e) (BnO)P(OCE)N(iPr)_2_, 5-ethylthio-1*H*-tetrazole, CH_2_Cl_2_, rt, 1 h, then *m*CPBA, −20 °C, 30 min, 79% for **29**, and 89% for **30**; (f) (i) Bu_4_NOH, THF, H_2_O, rt, and 4 h; (ii) Pd(OH)_2_, H_2_, MeOH, H_2_O, AcOH, 36 h, over two steps 75% for **6**, 78% for **8**, 76% for **10**, and 76% for **12**; and (g) (i) P(OCE)[N(iPr)_2_]_2_, tetrazole diisopropylamine, CH_2_Cl_2_, CH_3_CN, rt, 1 h, 80% toward **31**, and 90% toward **32**; (ii) 5-azidopentan-1-ol, 5-ethylthio-1*H*-tetrazole, 3 Å MS, CH_2_Cl_2_, CH_3_CN, rt, 1 h, then H_2_O, I_2_/THF, rt, 2 h, 36% for **31**, and 50% for **32**.

With the trisaccharide donor **20** in hand, we were able to construct six desired pseudotetrasaccharides (Scheme 2). First, glycosylation of donor **20** with ribitol acceptors **21** or **22** provided the desired four-unit main structures in 61 and 67% yields, respectively. In both cases, partial hydrolysis of donor **20** was observed that led to the corresponding hemiacetal and recovered the acceptors **21** and **22**. Primary TBDPS protecting groups were then removed to give pseudotetrasaccharides **23** and **24**, on which an aminopentyl linker was installed to give full-protected products **25** and **27**, respectively. These molecules without phosphate groups were fully deprotected by single-step hydrogenation, providing the simplest oligosaccharide antigens **5** and **9**. On the other hand, phosphate groups were installed at either the reducing end (Rbo residue; products **31** and **32**) or nonreducing end (terminal Glc residue; products **29** and **30**). Subsequently, the four phosphate-containing pseudotetrasaccharides went into sequential β-elimination and hydrogenation to afford synthetic antigens **6**, **8**, **10**, and **12**.

### Synthesis of Pseudotetrasaccharides with Bridging Phosphate

**Scheme 3:**
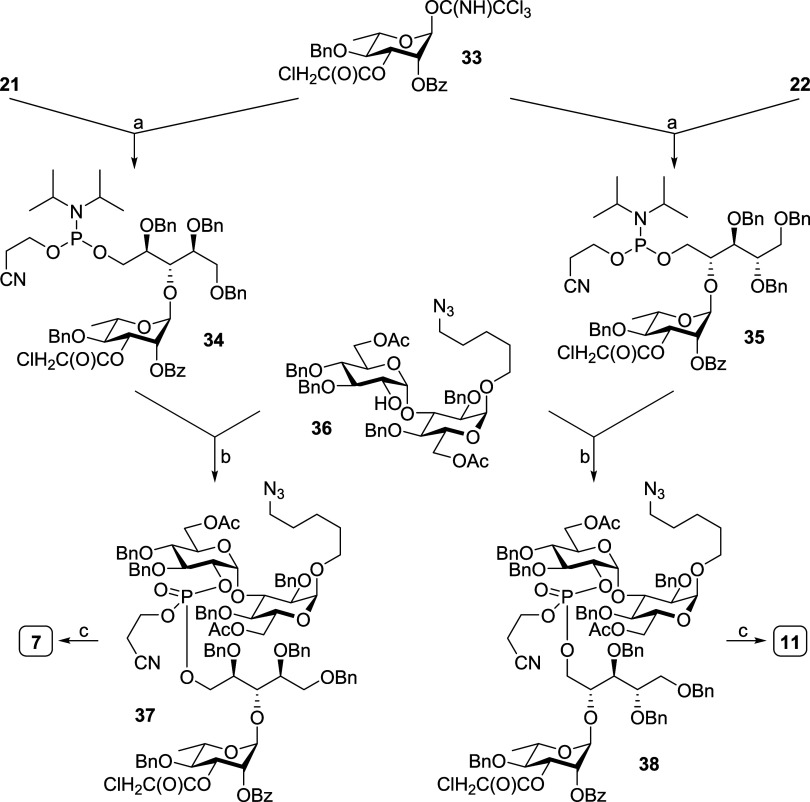
Synthesis of Phosphate-Bridged Pseudotetrasaccharides **7** and **11** Reagents and conditions: (a) (i) TMSOTf, 4 Å MS, CH_2_Cl_2_, −40 °C, 45 min, 85% from **21**, and 93% from **22**; (ii) HF-pyridine, THF, pyridine, 0 °C to rt, 8 h, 86% toward **34**, and 82% toward **35**; (iii) P(OCE)[N(iPr)_2_]_2_, tetrazole diisopropylamine, CH_2_Cl_2_, CH_3_CN, rt, 1 h, 98% for **34**, and 88% for **35**; (b) 5-ethylthio-1*H*-tetrazole, 3 Å MS, CH_3_CN, rt, 1 h, then H_2_O, I_2_/THF, rt, 2 h, 43% for **37**, and 52% for **38**; (c) (i) Bu_4_NOH, THF, H_2_O, rt, and 4 h; (ii) 0.3 M NaOMe, MeOH, CH_2_Cl_2_, and 2 h; (iii) Pd(OH)_2_, H_2_, MeOH, H_2_O, AcOH, 36 h, over three steps 72% for **7**, and 78% for **11**.

For the purpose of synthesizing phosphate-bridged pseudotetrasaccharides, Rha donor **33** was first glycosylated with Rbo acceptors **21** and **22**, and then, a phosphite was installed to provide pseudodisaccharides **34** and **35** for ST6C and ST6D (Scheme 3), respectively. Next, the pseudodisaccharides were coupled with disaccharide **36** in a [2 + 2] manner through phosphite. After iodine-mediated oxidation, fully protected phosphate-bridged pseudotetrasaccharides **37** and **38** were obtained. Lastly, sequential β-elimination, saponification, and hydrogenation gave the desired oligosaccharide antigens **7** and **11** with bridging phosphate.

**Scheme 4:**
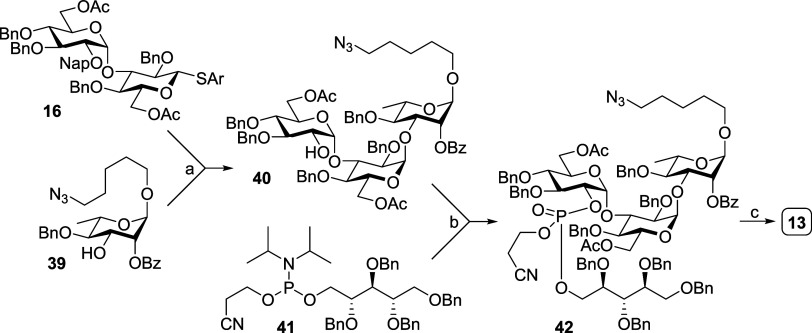
Synthesis of Common Pseudotetrasaccharide **13** Reagents and conditions: (a) (i) NIS, TfOH, 4 Å MS, CH_2_Cl_2_, −30 °C, 2 h, and 70%; (ii) DDQ, CH_2_Cl_2_, phosphate buffer pH 7, 0 °C to rt, 4 h, and 57%; (b) 5-ethylthio-1*H*-tetrazole, 3 Å MS, CH_3_CN, rt, 1 h, then H_2_O, I_2_/THF, rt, 2 h, and 57%; (c) (i) Bu_4_NOH, THF, H_2_O, rt, and 4 h; (ii) 0.3 M NaOMe, MeOH, CH_2_Cl_2_, and 24 h; (iii) Pd(OH)_2_, H_2_, MeOH, H_2_O, AcOH, 36 h, and 81% over three steps.

For the preparation of ST6C/ST6D common oligosaccharide, Glc-Glc disaccharide **16** was glycosylated with the linker-installed Rha acceptor **39**, giving trisaccharide **40** (Scheme 4). The trisaccharide was then coupled with ribitol phosphite **41** to produce phosphate-bridged pseudotetrasaccharide **42**. Subsequent three-step deprotection provided the shared antigen **13** for ST6C and ST6D.

### Glycan–Protein Incorporation and Mouse Immunization

In previous studies, the antibodies from ST6A and ST6B did not recognize the glycans that are composed of Gal-Glc-Rha-Rbo-phosphate, similar to oligosaccharides **8** and **12**.^48^ Therefore, oligosaccharides **8** and **12** are not considered to be candidate vaccine antigens in this study. Three synthetic oligosaccharide antigens each for ST6C (**5**–**7**) and ST6D (**9**–**11**) were incorporated onto the carrier protein CRM197 following the procedure described previously.^48^ The resulting glycoconjugates (**C1**–**C3** and **D1**–**D3**) were analyzed by MALDI-TOF mass spectrometry to determine the average glycan percentage (Table 1). Previously prepared glycoconjugates for ST6A (**A1** and **A2**) and ST6B (**B1** and **B2**) and their collected mouse antisera were included in this study. For immunogenicity studies of fragmentary ST6C and ST6D antigens, mice were immunized using the same conditions and timeline as those in the ST6A and ST6B study. Briefly, the glycoconjugates were mixed with Al(OH)_3_ to allow adsorption onto Al before intramuscular injection into 8 weeks old female BALB/c mice for a total of three shots of 2.2 μg of glycan with 292.7 μg of aluminum each with a 2 week interval between shots. The antisera were collected 10 days after the last shot for immunological evaluation.

**Table 1:** Analytic Results of the Glycoconjugates

glycoconjugate^a^	average glycan number	glycan percentage (%)
**A1** (CRM197-**1**)	4.31	5.78
**A2** (CRM197-**2**)	4.51	6.00
**B1** (CRM197-**3**)	6.97	9.01
**B2** (CRM197-**4**)	5.91	7.75
**C1** (CRM197-**5**)	9.15	10.7
**C2** (CRM197-**6**)	6.55	8.61
**C3** (CRM197-**7**)	6.87	9.00
**D1** (CRM197-**9**)	7.40	8.83
**D2** (CRM197-**10**)	6.19	8.19
**D3** (CRM197-**11**)	6.86	8.99

a **A1**, **A2**, **B1**, and **B2** were prepared previously.^48^

### Serological Glycan-Binding Antibody Analysis

**Figure 3:**
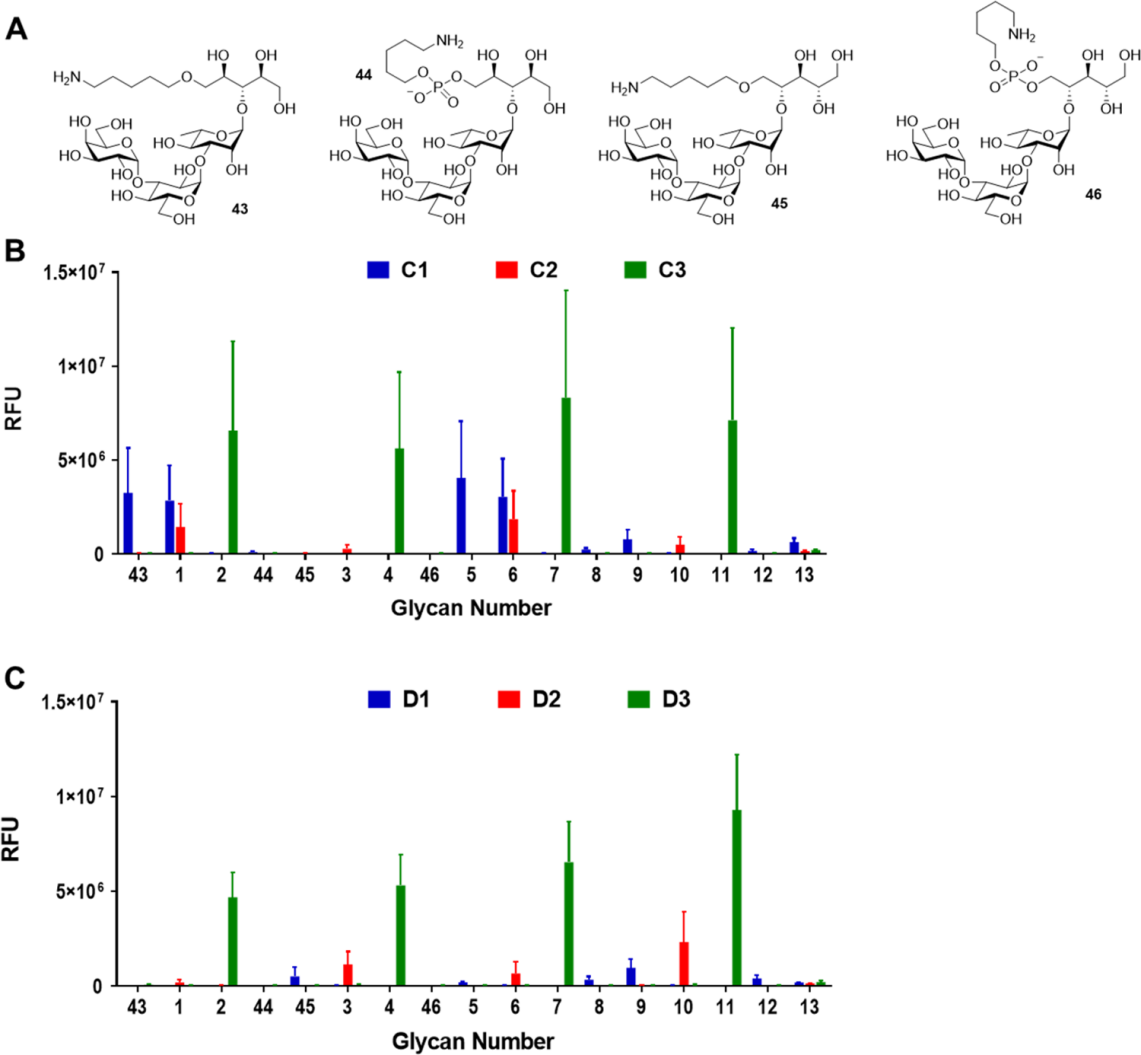
Mouse antiserum IgG analysis. Glycan microarray results of immobilized CPS **5**–**13**, also contained **1**–**4** and **43**–**46** from the previous study (A),^48^ and binding profile of antisera from immunization of ST6C (B) and ST6D (C) glycoconjugates (*n* = 5; 1:300 dilution). The bars display the mean ± SEM; RFU, relative fluorescence unit.

Synthetic oligosaccharides in this study (**5**–**13**, Figure 2) and previous study **(1–4**, **43**–**46**,^48^Figure 3A) were immobilized on *N*-hydroxylsuccinimide-coated slides for glycan microarray analysis.

Glycoconjugates C1, C2, and C3 antisera clearly recognized their corresponding oligosaccharides **5**, **6**, and **7** (Figure 3B), suggesting good immunogenicity of these glycoconjugates. Besides, C1 and C2 antisera, which were induced by vaccines containing pseudotetrasaccharides with or without the phosphate group, exhibited cross-recognition to 6A oligosaccharides (**43**, **1**) and only limited cross-recognition to 6D oligosaccharides (**9**, **10**, Figure 3B). Interestingly, C3 antiserum, which was induced by vaccines containing bridging phosphate, could recognize not only the corresponding oligosaccharide **7** but also **2**, **4**, and **11**, which were classified as 6A, 6B, and 6D, respectively (Figure 3B), indicating that all these glycoconjugate-elicited antibodies may cross-react to oligosaccharides of different serotypes, especially the C3 glycoconjugate.

The antisera induced by glycoconjugates D1, which contained pseudotetrasaccharides without the phosphate group, only showed lower recognition by oligosaccharides **9** (Figure 3C). In comparison, antisera from D2, which contained pseudotetrasaccharide phosphate, and antisera from D3, which contained pseudotetrasaccharide-bridging phosphate, could clearly recognize their corresponding oligosaccharides **10** and **11** (Figure 3C), suggesting the good immunogenicity of glycoconjugates D2 and D3. Besides, D1 and D2 antisera showed limited cross-recognition to 6B and 6C oligosaccharides. Interestingly, D3 antisera recognized not only the corresponding oligosaccharide **11** but also cross-recognized **2**, **4**, and **7**, which were classified as 6A, 6B, and 6C, respectively, indicating that these glycoconjugates are capable of generating cross-reactive antibodies to oligosaccharides of different serotypes, particularly the D3 glycoconjugate.

**Figure 4:**
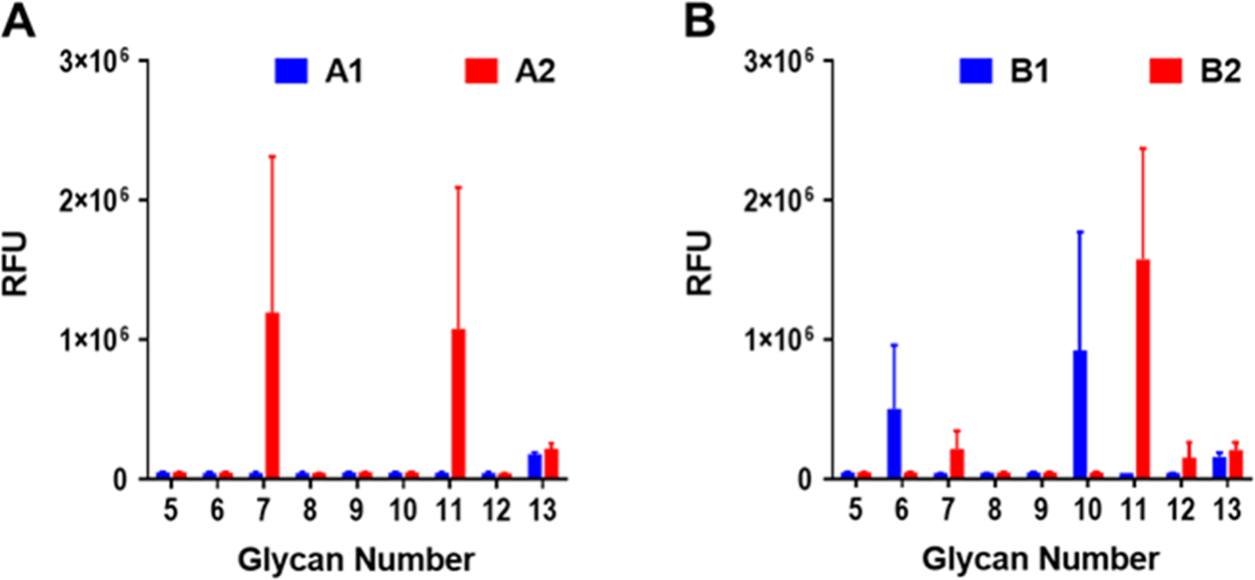
Mouse antiserum IgG analysis. Glycan microarray results of immobilized CPS compounds **5**–**13**. The binding profile of antisera from immunization of ST6A (A) and ST6B (B) glycoconjugates (*n* = 5; 1:300 dilution). The bars display the mean ± SEM; RFU, relative fluorescence unit.

Additionally, antisera induced by previous synthesized A1, which contained pseudotetrasaccharides with the phosphate group, recognized their corresponding oligosaccharides and cross-recognized 6A and 6B^48^ but could not cross-recognize 6C and 6D oligosaccharides (Figure 4A). In comparison, the antisera induced by B1, which contained pseudotetrasaccharides with the phosphate group, or antisera from A2 and B2, which also contained pseudotetrasaccharide-bridging phosphate, not only clearly recognized their corresponding oligosaccharides and cross-recognized 6A and 6B oligosaccharides^48^ but also exhibited limited cross-recognition to 6C and 6D oligosaccharides **6**, **7**, **10**, and **11** (Figure 4A,B), suggesting that the antibodies elicited from all these glycoconjugates may cross-react with oligosaccharides of different serotypes, especially the A2 and B2 glycoconjugates.

### Antiserum-Mediated Opsonophagocytosis

To validate the bactericidal efficacy of the antisera, we conducted an opsonophagocytic killing assay (OPKA) to evaluate the in vitro antibody-mediated bactericidal activity. The antisera obtained from immunization with the ST6A, ST6B, ST6C, and ST6D glycoconjugates were sequentially diluted and administered to associate bacteria SPn6A, SPn6B, SPn6C, and SPn6D. In the previous study, the OPKA assay of A2 antisera exhibited higher opsonophagocytic activity of SPn6A than that of A1 antisera, while B1 and B2 antisera showed similar responses to SPn6B. The cross-opsonic antibodies in A1 and A2 antisera were observed, however, not strong in SPn6B. B1 and B2 antisera were also observed but not strong either in SPn6A.^48^

**Figure 5:**
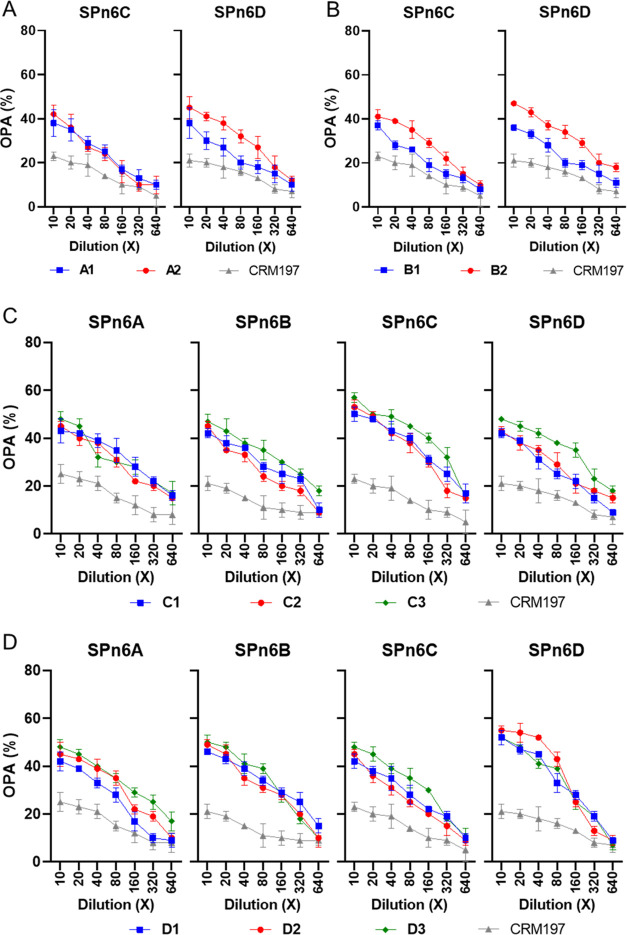
Opsonophagocytic killing assay (OPKA) of mouse antisera from immunization of ST6A (A), ST6B (B), ST6C (C), and ST6D (D) glycoconjugates. Antisera that contained glycan-recognizing antibodies were chosen and pooled for this assay. Antisera from CRM197-immunized mice were used as negative controls. The data represent the mean ± SD of three independent experiments.

In this study, we measured the cross-opsonic antibodies from ST6A and ST6B antisera with the bacteria SPn6C and SPn6D. The cross-opsonic antibodies in A1 and A2 antisera were observed, however, not strong in SPn6C; however, the cross-opsonic antibodies in A2 antisera were significantly higher than that in A1 antisera in SPn6D (Figure 5A). The cross-opsonic antibodies in B2 antisera were significantly higher than those of B1 antisera in SPn6C and SPn6D (Figure 5B). In addition, we measured the opsonophagocytic activity of SPn6C and SPn6D. The C3 antiserum was higher than the C1 and C2 antisera (Figure 5C), while the D2 antiserum was higher than the D1 and D3 antisera (Figure 5D). Besides, the cross-opsonic antibodies from ST6C antisera were observed stronger than the antisera from ST6A and ST6B, and also, the C3 antiserum was significantly higher than C1 and C2 antisera in SPn6A, SPn6B, and SPn6D (Figure 5C). Moreover, ST6D antisera were observed with stronger cross-opsonic antibodies than antisera from ST6A and ST6B too, and also, the D3 antiserum was higher than D1 and D2 antisera in SPn6A, SPn6B, and SPn6C (Figure 5D).

## Discussion

Based on our previous research,^48^ which indicated that structures featuring phosphate-terminated or phosphate-bridged pseudotetrasaccharides enhance immunogenicity and cross-reactivity, we have redirected our efforts toward synthesizing glycans of ST6C and ST6D that incorporate these structures for vaccine development. In this study, we synthesized CPS glycoconjugates and evaluated their immunogenicity and specificity against different serotypes. Our results showed that all the synthesized CPS glycoconjugates exhibited good immunogenicity, especially the serum antibodies induced by pseudotetrasaccharide phosphate B1, C2, and D2, which exhibited good specificity for recognizing CPS structures with exposed phosphates at the terminus. As phosphates are commonly found in biological organisms and serve as important charge sources in vaccine development, they often induce better immunogenicity.^51,52^

Moreover, C1 and C2 antisera not only recognized the corresponding oligosaccharides but also recognized the 6A oligosaccharide and exhibited limited binding to the 6D oligosaccharide. Interestingly, they did not bind to the 6B oligosaccharide. Similarly, serotype 6D glycoconjugates D1 and D2 antisera showed mild cross-reactivity to 6B and 6C oligosaccharides. 6A, 6B, 6C, and 6D differ in their Rha-Rbo linkage and oligosaccharide component (Gal-Glc- or Glc-Glc-). These characteristics can serve as the key epitopes for immunogenicity. Similarities between antigens may result in broadly cross-reactive antibody-binding pockets, while differences may result in lower cross-reactivity. For example, the differences in the component and linkage between 6B and 6C may lead to the C1 and C2 antisera not cross-reacting to the 6B oligosaccharide.

However, the C3 antisera recognized not only 6C but also 6A, 6B, and 6D. C3 contained bridging phosphate, which may be a more active immunogen than the linkage between Rha and Rbo for immune recognition. This may explain why the C3 antisera recognized all bridging phosphates, including those with different Rha-Rbo linkages. Interestingly, the A2, B2, and D3 glycoconjugates, which all contained bridging phosphate, induced antibodies that cross-reacted with serotypes 6A, 6B, 6C, and 6D. Furthermore, these antibodies induced by pseudotetrasaccharide-bridging phosphates did not significantly recognize the common pseudotetrasaccharide **13**. This further indicated that bridging phosphates may serve as more critical immunological recognition antigens.

In our observations, significant antibody production was detected at 1:300 dilution in the glycan array assay, yet the OPKA assay showed bacterial killing activity less than half of what was anticipated. This discrepancy is likely due to the different experimental setups. In the glycan array, high-concentration and homogeneous glycans served as clear targets, facilitating the amplification of the antibody signals through secondary antibodies. Conversely, in the OPKA assay, the heterogeneity of bacterial CPSs complicates the exposure of immunogenic fragments to antibodies, thus reducing the observed bactericidal activity despite the presence of antibodies at similar dilutions. Despite this, antibody concentrations exceeding 1:80 dilution achieved about 30–40% bactericidal effects, and even at 1:300 dilution, the bactericidal effect was significantly higher than in the negative control, which indicated positive bactericidal activity.

In the OPKA assay, A2, B2, C3, and D3 showed better cross-protection for the 6A, 6B, 6C, and 6D serotypes than other vaccine candidates that did not contain the bridging phosphate antigen structure, which is consistent with the array results. As bridging phosphate is a major component of lipoteichoic acid and wall teichoic acid found commonly in the cell wall of Gram-positive bacteria and *S. pneumoniae*,^53−55^ it is reasonable to suggest that bridging phosphate may be a key factor in achieving good cross-protection, and this finding could potentially be applied to other bacteria that have bridging phosphate.

In summary, the immunological evaluation results indicated that the linkage between Rha and Rbo and the substitution of galactose and glucose are key epitopes. Additionally, the presence of terminal phosphate groups can enhance vaccine immunogenicity. Meanwhile, the pseudotetrasaccharide bridging phosphate may serve as a potential candidate vaccine for providing cross-protection of SPn6.

## Conclusions

We synthesized a variety of SPn6C and SPn6D CPS fragments by chemical methods, which were used in glycoconjugate vaccines for mouse immunization. The glycan microarray and OPKA results showed that exposed phosphate groups could enhance the immunogenicity and cross-reactivity. Two pseudotetrasaccharide vaccine candidates with exposed bridging phosphate showed excellent cross-reactivity and may provide broad protection against major ST6 serogroup pneumococci. These findings provided an opportunity to develop synthetic pneumococcal vaccines for better protection.

## Methods

All oligosaccharides were synthesized by previously mentioned procedures, and the detailed procedures can be found in the Supporting Information. Selected oligosaccharides were immobilized on glass slides coated with *N*-hydroxysuccinimide esters by cross-linking with an aminopentyl linker. Selected oligosaccharides were incorporated onto CRM197 via succinimide 3-(bromoacetamido)propionate. The animal experiments were carried out following the protocols set forth by the Institutional Animal Care and Use Committee of the Academia Sinica. BioLASCO Taiwan provided female BALB/c mice for the immunization study. Each mouse was vaccinated three times, and each group consisted of five mice. The glycan microarray analysis was employed to examine the glycan-specific antibodies present in the antisera. Concurrently, the opsonophagocytic killing assay was conducted on HL-60 cells to measure the opsonic antibodies. Further experimental details can be found in the Supporting Information.

## Supporting Information Available

General methods, supplementary schemes, synthetic protocols, analytical data for new compounds, and glycan microarray analysis (PDF)
NMR spectra of compounds in this study (PDF)
Mass spectra of glycoconjugates and glycan binding data (PDF)
