# Supplementary material for: Chemical Synthesis of Truncated Capsular Oligosaccharide of Serotypes 6C and 6D of Streptococcus pneumoniae with Their Immunological Studies
Source: ACS Infect Dis. 2024 May 21;10(6):2161–71. doi: 10.1021/acsinfecdis.4c00147 (PMC11184553; doi:10.1021/acsinfecdis.4c00147)
Supplement: Supplementary file 1 — id4c00147_si_001.pdf [file id4c00147_si_001.pdf]

Supporting Information – 1

# Chemical Synthesis of Truncated Capsular Oligosaccharide of Serotypes 6C and 6D of *Streptococcus pneumoniae* with Their Immunological Studies

Ravinder Mettu,[1 #] Yang-Yu Cheng,[1,2 #] Hanmanth Reddy Vulupala,[1] Yu-Hsuan Lih,[1] Chiang-Yun Chen,[1] Mei-Hua Hsu,[3] Hong-Jay Lo,[1] Kuo-Shiang Liao,[1] Cheng-Hsun Chiu,[3] and Chung-Yi Wu[1]*

[1]Genomics Research Center, Academia Sinica, 128 Academia Road, Section 2, Nankang, Taipei, 11529 Taiwan

[2]Institute of Biochemistry and Molecular Biology, National Yang Ming Chiao Tung University, No. 155, Sec. 2, Linong St., Taipei 112304, Taiwan

[3]Molecular Infectious Disease Research Center, Chang Gung Memorial Hospital, Chang Gung University College of Medicine, 259 Wenhua 1st Road, Guishan, Taoyuan 33302, Taiwan

[#] These authors contributed equally.

Table of Contents

**General information** .......... **S4**
**General deprotection procedures** .......... **S4**
**Synthesis of monosaccharides** .......... **S5**
Glucose monosaccharide .......... S5
Rhamnose monosaccharide .......... S10
Ribitol .......... S13
**Synthesis of disaccharides** .......... **S14**
Glc-Glc disaccharide .......... S14
Rha-Rbo pseudo-disaccharide .......... S16
**Synthesis of trisaccharide donor 20** .......... **S20**
**Synthesis of pseudo-tetrasaccharides** .......... **S23**
Synthesis of pseudo-tetrasaccharides **5**, **6**, **7**, and **8** .......... S23
Synthesis of pseudo-tetrasaccharides **9**, **10**, **11**, and **12** .......... S32
**Synthesis of pseudo-tetrasaccharide 13** .......... **S41**
**Carrier protein incorporation of synthetic oligosaccharides** .......... **S46**
Modification of the synthetic oligosaccharides .......... S46
Oligosaccharide conjugation to carrier protein CRM197 .......... S49
**Immunization experiments** .......... **S50**
Vaccine formulation .......... S50
Mouse immunization .......... S50
**Glycan microarray** .......... **S51**
Glycan immobilization on glass slides .......... S51
Serologic assay with glycan microarray .......... S51
**Opsonophagocytic killing assay** .......... **S56**
**References** .......... **S57**

List of Figures

**Figure S1-S1.** Glycan microarray analysis of mouse antisera from ST6A and ST6B glycoconjugates immunization at different dilution ratios. ....S52
**Figure S1-S2.** Glycan microarray analysis of mouse antisera from ST6C glycoconjugates immunization at different dilution ratios. ....S53
**Figure S1-S3.** Glycan microarray analysis of mouse antisera from ST6D glycoconjugates immunization at different dilution ratios. ....S54
**Figure S1-S4.** Glycan microarray analysis of mouse antisera from CRM197 immunization at different dilution ratios....S55
**Figure S1-S5.** Glycan microarray analysis of pooled antisera from ST6A and ST6B glycoconjugates immunization. ....S55
**Figure S1-S6.** Glycan microarray analysis of pooled antisera from ST6C and ST6D glycoconjugates immunization. ....S56

List of Schemes

**Scheme S1-S1.** Synthesis of glucose building block....S5
**Scheme S1-S2.** Synthesis of rhamnose building block. ....S10
**Scheme S1-S3.** Synthesis of ribitol residue....S13
**Scheme S1-S4.** Synthesis of ribitol phosphoramidite. ....S43

## General information

All chemical reactions were carried out under an inert atmosphere unless mentioned otherwise, and standard syringe–septa techniques were followed. Solvents were purchased from Acros, Echo chemical, Merck, J. T. Baker Sigma-Aldrich, Fluka and used without further purification. The progress of all the reactions were monitored by TLC, using TLC glass plates precoated with silica gel 60 F254 (Merck). Column chromatography was performed on silica gel Geduran® Si 60 (40-63 µm, Merck). $^{1}H$ and $^{13}C$ spectra were recorded with Bruker AVANCE 600 MHz spectrometer, whereas $^{31}P$ NMR was recorded with Bruker AVANCE 500 MHz spectrometer at 25 °C and chemical shifts were measured in δ (ppm) with residual solvent peaks as internal standards ($CDCl_3$, δ 7.24 ppm, $D_2O$, δ 4.80 ppm in $^{1}H$ NMR and $CDCl_3$, δ 77.23 ppm in $^{13}C$ NMR). Coupling constants *J*, measured in Hz. Data are represented as follows: chemical shift, multiplicity (s = singlet, d = doublet, t = triplet, q = quartet, m = multiplet, br = broad). MALDI-TOF mass spectra were recorded on Bruker Ultraflex II TOF/TOF200 spectrameter using sinapinic acid as the matrix. HR ESI mass spectra were recorded on an APEX-ultra 9.4 T FTICR-MS (Bruker Daltonics).

## General deprotection procedures

**General deprotection procedure A:** To a stirred solution of starting material (1 equiv) in a mixture of MeOH/$H_2O$/$CH_2Cl_2$/AcOH (5.25 mL, 3/1/1/0.25, *v*/*v*/*v*) was added 20 wt% $Pd(OH)_2$/C (equivalent weight to starting material). The resulting mixture was stirred under hydrogen atmosphere (balloons) at room temperature for 36 h. The catalyst was filtered off, washed thoroughly with MeOH and concentrated *in vacuo*. The resulting residue was purified by Sephadex LH-20 column using distilled water as eluent. The collected appropriate fractions were concentrated *in vacuo* to afford desired compound.

**General deprotection procedure B:** To a stirred solution of phosphosugar (1 equiv) in a mixture of $CH_2Cl_2$ : $H_2O$ (2-3 mL, 1 : 1, *v*/*v*) was added tetrabutylammonium hydroxide (40% in water, 2 equiv) at room temperature. Then, it was stirred for 4-5 h at room temperature before diluted and extracted with $CH_2Cl_2$. The separated organic layer was dried over $MgSO_4$ and concentrated *in vacuo*. The obtained residue was dissolved in a mixture of MeOH/$H_2O$/AcOH (4.25 mL, 3 : 1 : 0.25, *v*/*v*/*v*), and 20 wt% $Pd(OH)_2$/C (equivalent weight to starting material) was added. Then, it was stirred under $H_2$ atmosphere for 36 h at room temperature. The catalyst was filtered off, washed thoroughly with MeOH, and concentrated *in vacuo*. The resulting residue was purified by Sephadex LH-20 column using distilled water as eluent. The collected appropriate fractions were concentrated *in vacuo* to afford desired compound.

**General deprotection procedure C:** To a stirred solution of phosphosugar (1 equiv) in a mixture of $CH_2Cl_2$:$H_2O$ (2-3 mL, 1:1, *v*/*v*) was added tetrabutylammonium hydroxide (40% in water, 2 equiv) at room temperature. Then, it was stirred for 4-5 h at room temperature before diluted and extracted

with $CH_2Cl_2$. The separated organic layer was dried over $MgSO_4$ and concentrated *in vacuo*. The resulting residue was dissolved in $CH_2Cl_2$ (1 mL), and followed NaOMe (3 mL, 0.3 M in MeOH) was added at rt. The reaction mixture continued stirring for 24 h at rt. Then, it was neutralized with IR-120, filtered and concentrated. The obtained residue was dissolved in a mixture of MeOH/$H_2O$/AcOH (4.25 mL, 3 : 1 : 0.25, *v*/*v*/*v*), and 20% $Pd(OH)_2$/C (equivalent weight to starting material) was added. Then, it was stirred under $H_2$ atmosphere for 36 h at rt. The catalyst was filtered off, washed thoroughly with MeOH and concentrated *in vacuo*. The resulting residue was purified by Sephadex LH-20 column using distilled water as eluent. The collected appropriate fractions were concentrated *in vacuo* to afford the desired compound.

## Synthesis of monosaccharides

### Glucose monosaccharide

**Scheme S1-S1.** Synthesis of glucose building block.[a]

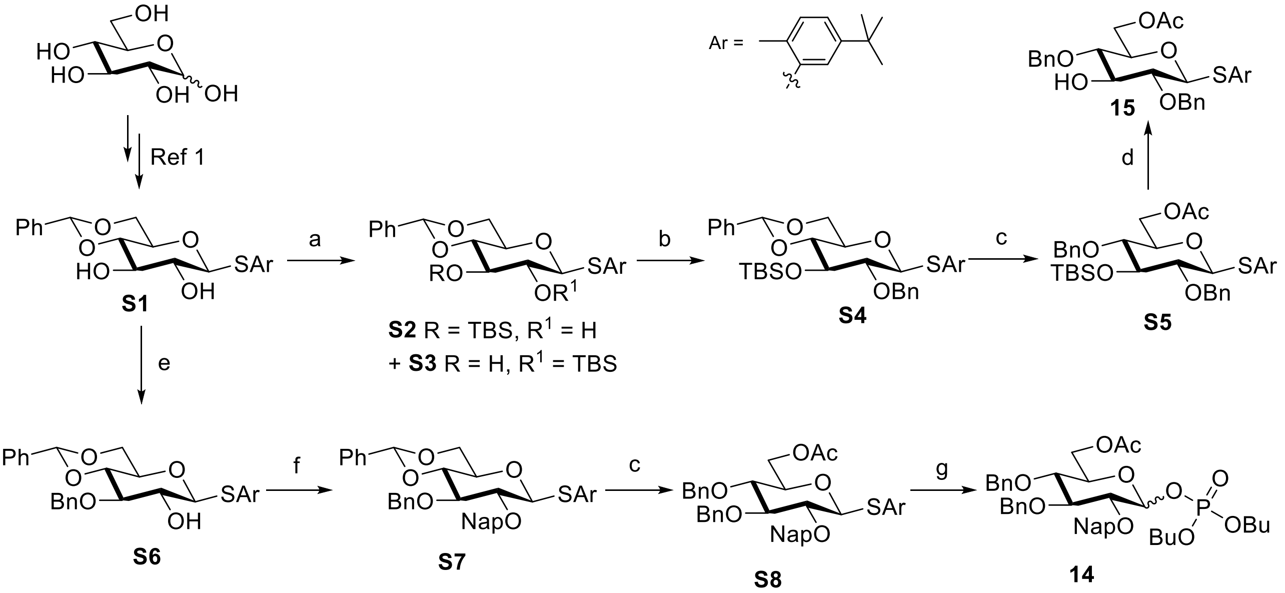


[a]Reagents and conditions: (a) TBSCl, imidazole, DMF, 0 °C to rt, 3.5 h, 88% of **S2**, 9% of **S3**; (b) NaH, BnBr, DMF, 0 °C to rt, 2 h, 99%; (c) i. $BH_3$·THF, $Cu(OTf)_2$, $CH_2Cl_2$, rt, 16 h; ii. $Ac_2O$, $NEt_3$, $CH_2Cl_2$, 0 °C to rt, 1-1.5 h, two steps 98% for **S5**, 97% for **S8**; (d) TBAF, THF, 0 °C to rt, 12 h, 98%; (e) $Bu_2SnO$, toluene, reflux, 3h, then CSF, BnBr, DMF, rt, overnight, 74%; (f) 2-napthylmethyl bromide, NaH, DMF, 0 °C to rt, 1.5 h, 91%; (g) dibutyl phosphate, NIS, TfOH, 4 Å MS, $CH_2Cl_2$, 96%.

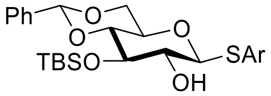

**(2-Methyl-5-*tert*-butylphenyl) 4,6-*O*-benzylidene-3-*O*-*tert*-butyldimethylsilyl-1-thio-β-D-glucopyranoside (S2).** To a stirred mixture of compound **S1**[1] (12.5 g, 29.057 mmol) and imidazole (3.956 g, 58.11 mmol) in anhydrous DMF (70 mL) was added TBSCl (5.7 g, 37.77 mmol) gradually at 0 °C. The reaction mixture was stirred 3.5 h at rt, and the solvent was removed under reduced pressure. The

resulting residue was dissolved in $CH_2Cl_2$ (150 mL) and washed with 1 N HCl, saturated aqueous $NaHCO_3$ and $H_2O$. The organic layer was dried over $MgSO_4$, filtered, and concentrated *in vacuo*. The obtained residue was purified by silica gel column chromatography (EtOAc/*n*-Hexane, 5 : 95) to afford compound **S2** (14 g, 88%) and **S3** (1.5 g, 9%) as white foam. **S2**: $^1$H NMR (600 MHz, $CDCl_3$): $\delta_H$ 7.59 (d, 1H, $J$ = 1.8 Hz), 7.46-7.40 (m, 2H), 7.35-7.32 (m, 3H), 7.23 (dd, 1H, $J$ = 1.8, 7.8 Hz), 7.13 (d, 1H, $J$ = 8.4 Hz), 5.50 (s, 1H), 4.64 (d, 1H, $J$ = 9.8 Hz ), 4.33 (dd, 1H, $J$ = 4.2, 10.8 Hz), 3.78-3.74 (m, 2H), 3.49-3.45 (m, 3H), 2.46 (brs, 1H), 2.39 (s, 3H), 1.29 (s, 9H), 0.85 (s, 9H), 0.09 (s, 3H), 0.02 (s, 3H). $^{13}$C NMR (150 MHz, $CDCl_3$): $\delta_C$ 149.7, 137.3, 137.1, 131.5, 130.2, 130.1, 129.2 128.3, 126.3, 125.4, 101.8, 89.1, 81.2, 76.2, 74.1, 70.8, 68.8, 34.6, 31.5, 26.0, 20.6, 18.5, -4.1, -4.5. HRMS (ESI-TOF) m/z: calcd for $C_{30}H_{45}O_5SSi$ [M+H]$^+$: 545.2751; found: 545.2754.

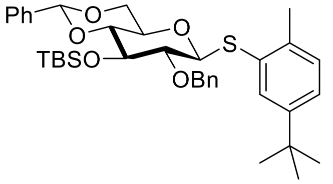


**(2-Methyl-5-*tert*-butylphenyl) 2-*O*-benzyl-4,6-*O*-benzylidene-3-*O*-*tert*-butyldimetylsilyl-1-thio-β-D-glucopyranoside (S4).** To a stirred solution of compound **S2** (8 g, 14.7 mmol) in anhydrous DMF (80 mL) were added BnBr (2.62 mL, 22.05 mmol) and NaH (1.03 g, 25.72 mmol) sequentially at 0 °C. The mixture was warm to room temperature and stirred until completion of the reaction (monitored by TLC, 2.5 h). After cooled to 0 °C, the reaction was quenched with MeOH. The solvent was removed under reduced pressure, and the obtained residue was diluted with $Et_2O$ and washed with cold water. The combined organic phase was dried over $MgSO_4$, filtered, and concentrated *in vacuo*. The resulted residue was purified by silica gel column chromatography (EtOAc/*n*-Hexane, 1 : 5) to afford compound **S4** (9.3 g, 99%) as a viscous liquid. $^1$H NMR (600 MHz, $CDCl_3$): $\delta_H$ 7.59 (d, 1H, $J$ = 1.8 Hz), 7.47-7.43 (m, 4H), 7.35-7.30 (m, 6H), 7.19 (dd, 1H, $J$ = 1.8, 7.8 Hz), 7.11 (d, 1H, $J$ = 8.0 Hz), 5.50 (s, 1H), 4.96 (d, 1H, $J$ = 10.2 Hz ), 4.85 (d, 1H, $J$ = 10.8 Hz), 4.78 (d, 1H, $J$ = 10.2 Hz), 4.32 (dd, 1H, $J$ = 4.8, 10.2 Hz), 3.93 (t, 1H, $J$ = 8.4 Hz), 3.80 (t, 1H, $J$ = 10.2 Hz), 3.56 (t, 1H, $J$ = 9.6 Hz), 3.49-3.43 (m, 2H), 2.33 (s, 3H), 1.28 (s, 9H), 0.85 (s, 9H), 0.02 (s, 3H), -0.01 (s, 3H). $^{13}$C NMR (150 MHz, $CDCl_3$): $\delta_C$ 149.7, 138.3, 137.3, 135.9, 133.3, 130.1, 129.2, 128.6, 128.5, 128.4, 128.3, 128.1 128.0, 127.8, 126.5, 124.6, 102.2, 88.6, 82.4, 81.7, 76.5, 76.0, 70.2, 68.9, 34.7, 31.5, 26.1, 20.4, 18.4, -3.9, -4.2. HRMS (ESI) m/z: calcd for $C_{37}H_{51}O_5SSi$ [M+H]$^+$: 635.3221; found 635.3220.

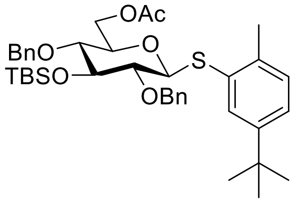


**(2-Methyl-5-*tert*-butylphenyl) 6-*O*-acetyl-2,4-di-*O*-benzyl-3-*O*-*tert*-butyldimetylsilyl-1-thio-β-D-glucopyranoside (S5).** To a stirred solution of starting material **S4** (13.45 g, 21.204 mmol) in

$BH_3$·THF complex (1 M in THF, 127 mL, 127.224 mmol) was added $Cu(OTf)_2$ (380 mg, 1.060 mmol) at room temperature under an argon atmosphere. After stirred for overnight, the reaction mixture was cooled to 0 °C and carefully quenched by sequential addition of $Et_3N$ (2.95 mL, 21.204 mmol) and MeOH (added dropwise until hydrogen gas evolution ceased). The solvent was removed under reduced pressure, and the obtained residue was purified by silica gel column chromatography (EtOAc/hexanes, 1:4 to 1:3) to give the alcohol compound as a colorless viscous liquid (13.4 g, 98%). The purified alcohol compound was dissolved in anhydrous $CH_2Cl_2$ (70 mL) and $Ac_2O$ (3 mL, 31.555 mmol, 1.5 equiv), followed by addition of $Et_3N$ (5.85 mL, 42.074 mmol, 2 equiv) and 4-(dimethylamino)pyridine (DMAP) (260 mg, 2.103 mmol, 0.1 equiv) at 0 °C. The reaction was kept stirring until TLC analysis indicated disappearance of starting material (1.5 h). Then, the reaction mixture was quenched by MeOH (3 mL) and the solvent was removed *in vacuo*. The obtained residue was purified by silica gel column chromatography (EtOAc/*n*-Hexane, 1 : 10) to give compound **S5** as a colorless viscous liquid (14.1 g, 98%). $^1$H NMR (600 MHz, $CDCl_3$): $\delta_H$ 7.54 (d, 1H, *J* = 2.0 Hz), 7.42 (d, 2H, *J* = 7.3 Hz),7.33-7.23 (m, 8H), 7.16 (dd, 1H, *J* = 2.0, 7.9 Hz), 7.07 (d, 1H, *J* = 7.9 Hz), 4.97 (d, 1H, *J* = 10.6 Hz), 4.88 (d, 1H, *J* = 11.3 Hz), 4.76 (d, 1H, *J* = 10.6 Hz), 4.68 (d, 1H, *J* = 9.7 Hz), 4.53 (d, 1H, *J* = 11.3 Hz), 4.34 (dd, 1H, *J* = 1.9, 11.9 Hz), 4.13 (dd, 1H, *J* = 5.0, 11.9 Hz), 3.78 (t, 1H, *J* = 8.6 Hz), 3.54-3.51 (m, 1H), 3.45 (t, 1H, *J* = 9.0 Hz), 3.40 (t, 1H, *J* = 8.9 Hz), 2.29 (s, 3H), 1.99 (s, 3H), 1.27 (s, 9H), 0.95 (s, 9H), 0.02 (s, 3H), -0.02 (s, 3H). $^{13}$C NMR (150 MHz, $CDCl_3$): $\delta_C$ 170.9, 149.7, 138.4, 137.8, 135.9, 133.8, 130.0 128.6, 128.3, 128.0, 127.8, 127.7, 124.5, 88.4, 81.6, 78.9, 78.7, 76.9, 75.5, 75.4, 63.8, 34.7, 31.5, 26.3, 21.1, 20.4, 18.2, -3.6, -3.7. HRMS (ESI) m/z: calcd for $C_{39}H_{54}O_6SSiNa$ $[M+Na]^+$: 701.3303; found 701.3309.

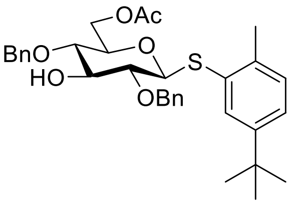


**(2-Methyl-5-*tert*-butylphenyl) 6-*O*-acetyl-2,4-di-*O*-benzyl-1-thio-β-D-glucopyranoside (15).**[2] To a stirred solution of starting material **S5** (14.1 g, 20.765 mmol) in THF (80 mL) was added TBAF (25 mL, 24.918 mmol) at 0 °C under an argon atmosphere. The mixture was warmed to room temperature and stirred until disappearnce of starting material on TLC (12 h). Then the reaction was quenched with saturated aqueous $NH_4Cl$ (15 mL) and diluted with $CH_2Cl_2$. The organic phase was separated and dried over $MgSO_4$, filtered, and concentrated *in vacuo*. The obtained residue was purified by silica gel column chromatography (EtOAc/hexanes, 1:4) to afford compound **15** as a colorless viscous liquid (14.1 g, 98%).

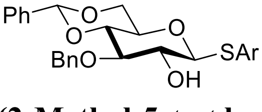


**(2-Methyl-5-*tert*-butylphenyl) 3-*O*-benzyl-4,6-*O*-benzylidene-1-thio-β-D-glucopyranoside (S6).** A mixture of starting material **S1** (15 g, 34.87 mmol) and dibutyltin oxide (11.3 g, 45.33 mmol) in

toluene (120 mL) was stirred at reflux for 3 h with azeotropic removal of water using dean-stark apparatus, during which the mixture became clear solution. The solvent was removed under reduced pressure and co-evaporated twice with toluene. The obtained residue was dissolved in *N*,*N*-dimethylformamide (120 mL) and BnBr (5.2 mL, 43.58 mmol), followed by addition of cesium fluoride (6.62 g, 43.58 mmol). The reaction mixture was stirred overnight at room temperature. The solvent was removed, and the residue was diluted with EtOAc (200 mL), washed with saturated aqueous $NaHCO_3$ (75 mL) and brine (50 mL). The organic phase was dried over $MgSO_4$, filtered, and concentrated *in vacuo*. The residue was purified by silica gel column chromatography (EtOAc/*n*-Hexane, 1:5) to give compound **S6** as white powder (13.3 g, 74%) with recoverd starting material **S1** (3.5 g, 23%). $^1$H NMR (600 MHz, $CDCl_3$): $\delta_H$ 7.64 (d, 1H, *J* = 1.8 Hz), 7.58 (dd, 2H, *J* = 2.0, 8.0 Hz), 7.43-7.26 (m, 9H), 7.18 (d, 1H, *J* = 8.0 Hz), 5.61 (s, 1H), 5.01 (d, 1H, *J* = 11.4 Hz ), 4.85 (d, 1H, *J* = 11.4 Hz), 4.68 (d, 1H, *J* = 9.7 Hz), 4.42 (dd, 1H, *J* = 4.8, 10.4 Hz), 3.85 (t, 1H, *J* = 10.2 Hz), 3.73-3.70 (m, 2H), 3.62-3.54 (m, 2H), 2.60 (brs, 1H), 2.45 (s, 3H), 1.34 (s, 9H). $^{13}$C NMR (150 MHz, $CDCl_3$): $\delta_C$ 149.7, 138.4, 137.4, 137.3, 130.9, 130.6, 130.2, 129.2, 128.6, 128.5, 128.3, 128.1, 126.2, 125.5, 101.4, 88.8, 81.9, 81.3, 74.9, 72.8, 70.8, 68.8, 34.6, 31.5, 20.6. HRMS (ESI) m/z: calcd for $C_{31}H_{36}O_5SNa$ $[M+Na]^+$: 543.2176; found: 543.2175.

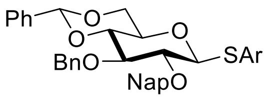


**(2-Methyl-5-*tert*-butylphenyl) 3-*O*-benzyl-4,6-*O*-benzylidene-2-*O*-(2-napthylmethyl)-1-thio-β-D-glucopyranoside (S7).** To a stirred solution of **S6** (7.3 g, 14.03 mmol) in DMF (60 mL) was added NaH (60% dispersion in mineral oil, 840 mg, 21.04 mmol) at 0 ºC under an argon atmosphere. After stirred for 5 min, NapBr (4 g, 18.24 mmol) was added. The reaction mixture was stirred at room temperature until the starting material disappeared on TLC (1.5 h). The reaction was quenched with MeOH, poured into ice water, and extracted with $Et_2O$ (75 mL × 3). The comibined organic layer was dried over $MgSO_4$ and concentrated *in vacuo*. The obtained residue was purified by silica gel column chromatography (EtOAc/*n*-Hexane, 1 : 4) to give compound **S7** as a white foam (8.51 g, 91%). $^1$H NMR (600 MHz, $CDCl_3$): $\delta_H$ 7.83-7.77 (m, 4H), 7.62 (d, 1H, *J* = 1.7 Hz), 7.54 (d, 1H, *J* = 8.3 Hz), 7.49-7.27 (m, 12H), 7.21 (dd, 1H, *J* = 1.7, 7.8 Hz), 7.13 (d, 1H, *J* = 7.9 Hz), 5.60 (s, 1H), 5.09 (d, 1H, *J* = 10.4 Hz), 5.03 (d, 1H, *J* = 10.5 Hz), 4.99 (d, 1H, *J* = 11.3 Hz), 4.83 (d, 1H, *J* = 11.3 Hz), 4.79 (d, 1H, *J* = 9.9 Hz), 4.37 (dd, 1H, *J* = 4.9, 10.4 Hz), 3.89 (t, 1H, *J* = 8.9 Hz), 3.86 (t, 1H, *J* = 10.2 Hz), 3.80 (t, 1H, *J* = 9.5 Hz), 3.66 (t, 1H, *J* = 9.7 Hz), 3.51(dt, 1H, *J* = 4.9, 9.7 Hz), 2.40 (s, 3H), 1.28 (s, 9H). $^{13}$C NMR (150 MHz, $CDCl_3$): $\delta_C$ 149.7, 138.5, 137.4, 136.3, 135.7, 133.5, 133.3, 132.9, 130.1, 129.2, 129.1, 128.6 ,128.4, 128.3 ,128.2, 128.1, 127.9, 127.8, 127.1, 126.4, 126.2, 126.1, 126.0, 124.9, 101.3, 88.7, 83.2, 81.7, 81.0, 76.2, 75.4, 70.3, 68.9, 34.6, 31.4, 20.5. HRMS (ESI) m/z: calcd for $C_{42}H_{45}O_5S$ $[M+H]^+$: 661.2982; found: 661.2990.

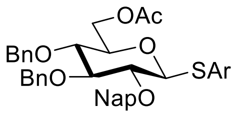


**(2-Methyl-5-*tert*-butylphenyl) 6-*O*-acetyl-3,4-*O*-dibenzyl-2-*O*-(2-napthylmethyl)-1-thio-β-D-glucopyranoside (S8).** To a stirred solution of starting material **S7** (8.51 g, 12.87 mmol) in $BH_3$·THF complex (1 M in THF, 77 mL, 77.26 mmol) was added $Cu(OTf)_2$ (233 mg, 0.643 mmol) at room temperature under an argon atmosphere. After stirred for overnight, the reaction mixture was cooled to 0 °C and carefully quenched by sequential addition of $Et_3N$ (1.79 mL, 12.87 mmol) and MeOH (added dropwise until hydrogen gas evolution ceased). The solvent was removed under reduced pressure, and the obtained residue was purified by silica gel column chromatography (EtOAc/hexanes, 1:7) to give the alcohol compound as a colorless viscous liquid (8.51 g, 100%). The purified alcohol compound was dissolved in anhydrous $CH_2Cl_2$ (60 mL) and $Ac_2O$ (1.8 mL, 19.25 mmol, 1.5 equiv), followed by addition of $Et_3N$ (3.56 mL, 25.67 mmol, 2 equiv) and 4-(dimethylamino)pyridine (DMAP) (156 mg, 1.28 mmol, 0.1 equiv) at 0 °C. The reaction was kept stirring until TLC analysis indicated disappearance of starting material (1 h). Then, the reaction mixture was quenched by MeOH (2 mL) and the solvent was removed *in vacuo*. The obtained residue was purified by silica gel column chromatography (EtOAc/*n*-Hexane, 1 : 10) to give compound **S8** as a colorless viscous liquid (8.8 g, 97%). $^1$H NMR (600 MHz, $CDCl_3$): $\delta_H$ 7.88-7.81 (m, 4H), 7.68 (s, 1H), 7.60 (d, 1H, $J$ = 8.5 Hz),7.53-7.25 (m, 13H), 7.19 (d, 1H, $J$ = 7.9 Hz), 5.22 (d, 1H, $J$ = 10.4 Hz), 5.05 (q, 2H, $J$ = 10.9 Hz), 4.96 (d, 2H, $J$ = 10.9 Hz), 4.77 (d, 1H, $J$ = 9.8 Hz), 4.67 (d, 1H, $J$ = 10.8 Hz), 4.42 (d, 1H, $J$ = 11.7 Hz), 4.33 (dd, 1H, $J$ = 4.7, 11.7), 3.84 (t, 1H, $J$ = 8.7 Hz), Hz), 3.71-3.62 (m, 3H), 2.47 (s, 3H), 2.09 (s, 3H), 1.35 (s, 9H). $^{13}$C NMR (150 MHz, $CDCl_3$): $\delta_C$ 170.9, 149.7, 138.4, 137.7, 136.3, 135.5, 133.4, 133.3, 133.2, 130.0, 128.7(2), 128.6, 128.3, 128.2, 128.1, 127.9, 127.8(2), 127.1, 126.3, 126.2, 126.1, 124.7, 88.4, 86.9, 81.3, 77.5, 75.9, 75.8, 75.3, 63.6, 34.6, 31.5, 21.1, 20.5. HRMS (ESI) m/z: calcd for $C_{44}H_{49}O_6S$ $[M+H]^+$: 705.3244; found: 705.3256.

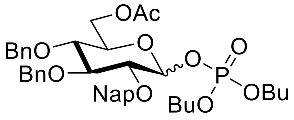


**Dibutyl 6-*O*-acetyl-3,4-*O*-dibenzyl-2-*O*-(2-napthylmethyl)-D-glucopyranosyl-1-phosphate (14).** A mixture solution of thioglucoside **S8** (9.5 g, 13.476 mmol, 1 equiv), dibutyl phosphate (8 mL, 40.430 mmol, 3 equiv) and activated pulverized 4 Å molecular sieves (15 g) in anhydrous $CH_2Cl_2$ (100 mL) was stirred under an argon atmosphere for 1 h. After cooled to 0 °C, NIS (6.06 g, 26.953 mmol, 2 equiv) and TfOH (0.5 M in $Et_2O$, 8.08 mL, 4.043 mmol, 0.3 equiv) was added. The reaction was stirred until completion (monitored by TLC, 16 h). Then, the reaction was quenched by addition of saturated aqueous $NaHCO_3$ (5 mL) and filtered through pad of celite. The filtrate was washed with saturated aqueous $Na_2S_2O_3$ (50 mL), saturated aqueous $NaHCO_3$ (50 mL), and brine (30 mL). The separated organic layer was dried over $MgSO_4$, filtered, and concentrated *in vacuo*. The resulted residue was purified by silica gel column chromatography (EtOAc/*n*-Hexane, 1 : 3 to 1 : 2) to give compound **14** as a pale yellow viscous liquid (9.5 g, 96%, α,β-mixture 1:1.1). $^1$H NMR (600 MHz,

CDCl3, α,β-mixture): $\delta_H$ 7.81-7.73 (m, 8H), 7.49-7.43 (m, 6H), 7.34-7.24 (m, 20H), 5.91 (dd, 1H, $J$ = 3.0, 7.1 Hz), 5.23 (t, 1H, $J$ = 7.4 Hz), 5.05 (d, 1H, $J$ = 11.1 Hz), 5.02 (d, 1H, $J$ = 10.8 Hz), 4.96 (d, 1H, $J$ = 11.5 Hz), 4.92-4.80 (m, 7H), 4.58 (d, 1H, $J$ = 5.0 Hz), 4.56 (d, 1H, $J$ = 4.9 Hz), 4.36 (dd, 1H, $J$ = 1.6, 12.0 Hz), 4.28 (dd, 1H, $J$ = 4.0, 12.0 Hz), 4.22-4.17 (m, 2H), 4.09-3.96 (m, 10H), 3.74 (t, 1H, $J$ = 9.1 Hz), 3.66-3.62 (m, 2H), 3.59-3.53 (m, 3H), 2.01 (s, 3H), 1.99 (s, 3H), 1.65-1.60 (m, 4H), 1.57-1.51 (m, 4H), 1.39-1.33 (m, 4H), 1.28-1.21 (m, 4H), 0.91-0.87 (m, 6H), 0.80-0.77 (m, 6H). $^{13}$C NMR (150 MHz, $CDCl_3$): $\delta_C$ 170.8, 170.7, 138.5, 138.3, 137.8, 137.7, 135.5, 135.1, 133.4(2), 133.2, 133.1, 128.7(2), 128.6, 128.4, 128.3(3), 128.2(2), 128.1, 128.0, 127.9(2), 127.8. 127.1, 126.6, 126.3(2), 126.2(2), 126.1, 126.0, 98.8(2) (C1 $^1J_{CH}$ = 164 Hz), 94.9(2) (C1 $^1J_{CH}$ = 175 Hz), 84.5(2), 82.1, 82.0, 81.3, 79.4, 79.3, 76.7, 75.9(2), 75.4, 75.2, 75.1, 73.7, 73.0, 70.8, 68.1, 68.0(2), 67.9(2), 67.7(2), 62.8(2), 32.4(2), 32.3(3), 20.9, 18.8(2), 18.7(2), 13.7(2), 13.6(2). HRMS (ESI) m/z: calcd for $C_{41}H_{52}O_{10}P$ $[M+H]^+$: 735.3293; found: 735.3301.

**Rhamnose monosaccharide**

**Scheme S1-S2.** Synthesis of rhamnose building block.[a]

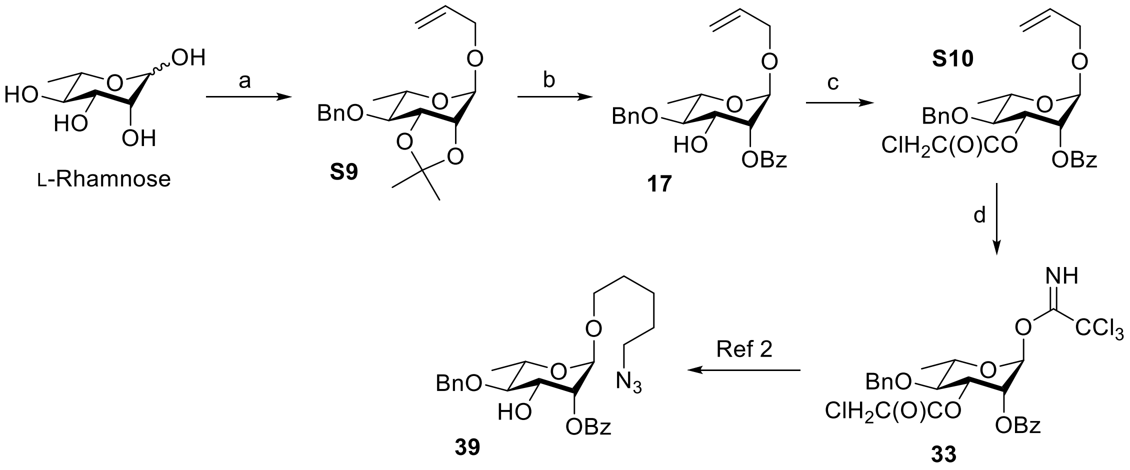


[a]Reagents and conditions: (a) i. Allyl alcohol, TfOH, rt to reflux, 3 h; ii. 2,2-dimethoxypropane, dry acetone, PTSA, rt, 1 h; iii. NaH, BnBr, DMF, 0 °C to rt, overnight, 70% over three steps; (b) i. 4% $HCl_{(aq)}$, MeOH, 45 °C, overnight, 91%; ii. Triethyl orthobenzoate, CSA, DMF, rt to 50 °C, 3 h, then 80% $CH_3COOH_{(aq)}$, 97%; (c) $ClCH_2COCl$, pyridine, $CH_2Cl_2$, 0 °C to rt, 3 h, 98%; (d) i. $PdCl_2$, MeOH/$CH_2Cl_2$ (1/1), rt, 4 h, 90%; ii. Trichloroacetonitrile, DBU, 4 Å MS, $CH_2Cl_2$, 0 °C to rt, 1 h, 79%.

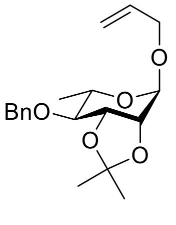


**Allyl 4-*O*-benzyl-2,3-*O*-isopropylidene-α-L-rhamnopyranoside (S9).** To a mixture of L-rhamnose (25 g) and allyl alcohol (150 mL) was added trifluoromethanesyulphonic acid (2 mL) at room temperature. The reaction mixture was stirred at reflx for 3 h. After cooled, the mixture was neutralized with triethylamine (5 mL). The reaction residue was filtered, concentrated, and co-

distilled with toluene (150 mL × 2). The resulted residue was dried under high vacuum for 30 min and redissolved in a mixtre of dry acaetone (100 mL), and 2,2-dimethoxypropane (25 mL). Then, *p*-toluene sulphonic acid (PTSA) (500 mg) was added at room temperature under an argon atmosphere. After stirred for 1 h, trietheylamine (2 mL) was added to the reaction mixture. The mixture was concentrated, co-distilled with toluene (100 mL × 2), and dried under high vacuum for 30 min to afford a syrup. The syrup was redissolved in dry DMF (70 mL), and the solution was slowly added to a stirred suspension of NaH (7 g) in DMF (50 mL) at 0 °C under an argon atmosphere. Then, benzyl bromide (15 mL) was added dropwise to the mixture. After stirred overnight at room temperature, the reaction mixture was carefully quenched with MeOH at 0 °C and poured into cold water (200 mL). The residue was extracted with ethyl acetate (100 mL × 3), and the combined organic layer was dried over $MgSO_4$ and concentrated under reduced pressure. The residue was purified by silica gel column chromatography (EtOAc/*n*-Hexane, 1 : 6) to give α-isomer compound **S9** (28.61 g) with its β-isomer (3.5 g) as pale yellow viscous comounds (70 % overall yield). $^1$H NMR (600 MHz, $CDCl_3$, α-isomer): $\delta_H$ 7.35-7.30 (m, 4H), 7.27-7.24 (m, 1H), 5.91-5.84 (m, 1H), 5.29-5.26 (m, 1H), 5.19 (dd, 1H, *J* =1.3, 10.2 Hz), 4.99 (s, 1H), 4.86 (d, 1H, *J* = 11.6 Hz), 4.62 (d, 1H, *J* = 11.6 Hz), 4.27 (t, 1H, *J* = 6.2 Hz), 4.16-4.13 (m, 2H), 3.98-3.95 (m, 1H), 3.72-53.67 (m, 1H), 3.21 (dd, 1H, *J* = 7.0 Hz), 1.49 (s, 3H), 1.35 (s, 3H), 1.27 (d, 3H, *J* = 6.3 Hz). $^{13}$C NMR (150 MHz, $CDCl_3$): $\delta_C$ 138.5, 133.8, 128.4, 128.1, 127.8, 117.8, 109.3, 96.3 (C1 $^1J_{CH}$ = 172 Hz), 81.3, 78.8, 76.2, 73.1, 68.0, 64.7, 28.1, 26.5, 18.0. HRMS (ESI) m/z: calcd for $C_{19}H_{27}O_5$ $[M+H]^+$: 335.1853; found: 335.1854.

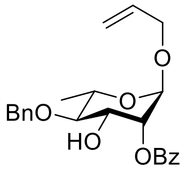


**Allyl 2-*O*-benzoyl-4-*O*-benzyl-α-L-rhamnopyranoside (17).** A solution of compound **S9** in MeOH containing 4% HCl was stirred at 45 °C overnight. The reaction mixture was cooled to room temperature and neutralized with solid $NaHCO_3$, filtered, and concentrated *in vacuo*. The obtained residue was dissolved in minimium amount of $CH_2Cl_2$ and crystalized with hexane to gave a diol compound as white prisms. The prepared diol compound (5 g, 16.987 mmol) was dissolved in DMF (50 mL) and triethyl orthobenzoate (5.8 mL, 25.480 mmol), followed by addition of camphorsulfonic acid (CSA) (0.4 g, 1.698 mmol) at room temperature under an argon atmosphere. After stirred 3 h to at 50 °C, the solvent was removed under vaccuum on a rota evaporator. The resulted residue was dissolved in 80% aqueous acetic acid (25 mL) and then stirred at room temperature for 10 min before evaporated to dryness. The obtained residue was purified by silica gel column chromatography (EtOAc/*n*-Hexane, 1 : 4) to give compound **17** (6.6 g, 97%) as a clear syrup. $^1$H NMR (600 MHz, $CDCl_3$): $\delta_H$ 8.05 (d, 2H, *J* = 7.9 Hz), 7.58 (t, 1H, *J* = 7.3 Hz), 7.45 (t, 2H, *J* = 7.6 Hz), 7.37-7.28 (m, 5H), 5.92-5.85 (m, 1H), 5.36 (s, 1H), 5.30 (d, 1H, *J* = 17.0 Hz), 5.20 (d, 1H, *J* = 10.3 Hz), 4.87 (s, 1H), 4.88 (d, 1H, *J* = 11.3 Hz), 4.74 (d, 1H, *J* = 11.1 Hz), 4.26 (p, 1H, *J* = 4.6 Hz), 4.18 (dd, 1H, *J* = 5.0, 13.0 Hz), 4.06 (dd, 1H, *J* = 6.0, 13.0 Hz), 3.89-3.81 (m, 1H), 3.49 (t, 1H, *J* = 9.3 Hz), 2.47 (d, 1H, *J* = 4.7 Hz), 1.39 (d, 3H, *J* = 6.2 Hz). $^{13}$C NMR (150 MHz, $CDCl_3$): $\delta_C$ 166.4, 138.3, 133.6, 133.4,

130.0, 129.8, 128.6, 128.5, 128.2, 128.0, 117.7, 96.7, 81.7, 75.3, 73.5, 70.6, 68.2, 67.6, 18.2. HRMS (ESI) m/z: calcd for $C_{23}H_{26}O_6Na$ $[M+Na]^+$: 421.1622; found: 421.1622.

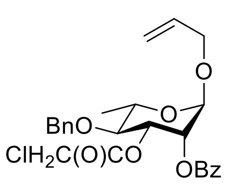


**Allyl 2-*O*-benzoyl-4-*O*-benzyl-3-*O*-chloroacetyl-α-L-rhamnopyranoside (S10).** To a solution of **17** (1.2 g, 3.011 mmol) in $CH_2Cl_2$ (10 mL) and pyridine (1.22 mL, 15.058 mmol) was added dropwise a solution of chloroacetyl chloride (480 μL, 6.023 mmol) in $CH_2Cl_2$ (1 mL) at 0 °C under an argon atmosphere. The reaction mixture was stirred at room temperature for 3 h and then quenched by careful addition of MeOH at 0 °C. The solution was evaporated, and the residue was dissolved in $CH_2Cl_2$ and washed with saturated aqueous $NaHCO_3$ and water. The organic layer was dried over $MgSO_4$, filtered, and concentrated *in vacuo*. The resulting residue was purified by silica gel column chromatography (EtOAc/*n*-Hexane, 1 : 5) to give the title compound **S10** (1.41 g, 98%) as a colorless oil. $^1H$ NMR (600 MHz, $CDCl_3$): $\delta_H$ 8.05 (d, 2H, *J* = 7.6 Hz), 7.61 (t, 1H, *J* = 7.5 Hz), 7.48 (t, 2H, *J* = 7.7 Hz), 7.33-7.26 (m, 5H), 5.92-5.85 (m, 1H), 5.51 (dd, 1H, *J* = 1.8, 3.2 Hz), 5.48 (dd, 1H, *J* = 3.4, 9.7 Hz), 5.31 (dd, 1H, *J* = 1.2, 17.2 Hz), 5.21 (dd, 1H, *J* = 1.1, 10.3 Hz), 4.88 (d, 1H, *J* = 1.5 Hz), 4.70 (d, 1H, *J* = 11.2 Hz), 4.66 (d, 1H, *J* = 11.2 Hz), 4.20 (dd, 1H, *J* = 5.2, 13.0 Hz), 4.03 (dd, 1H, *J* = 6.1, 12.8 Hz), 3.94-3.91 (m, 1H), 3.90 (d, 1H, *J* = 14.8 Hz), 3.83 (d, 1H, *J* = 14.8 Hz), 3.64 (t, 1H, *J* = 9.6 Hz), 1.39 (d, 3H, *J* = 6.2 Hz). $^{13}C$ NMR (150 MHz, $CDCl_3$): $\delta_C$ 166.5, 165.8, 138.0, 133.6, 133.4, 130.0, 129.6, 128.7, 128.6, 128.1, 128.0, 118.1, 96.6, 78.8, 75.4, 74.2, 70.7, 68.4, 67.9, 40.8, 18.2. HRMS (ESI) m/z: calcd for $C_{25}H_{27}ClO_7Na$ $[M+Na]^+$: 497.1338; found: 497.1338.

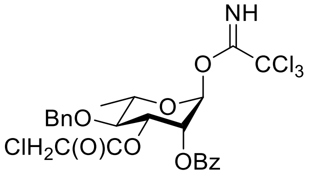


**2-*O*-Benzoyl-4-*O*-benzyl-3-*O*-chloroacetyl-α-L-rhamnopyranosyl trichloroacetimidate (33).**[2] To a solution of **S10** (1.34 g, 2.826 mmol) in a mixture of MeOH/$CH_2Cl_2$ (10 mL, 1/1) was added $PdCl_2$ (100 mg, 0.565 mmol) at room temperature under an argon atmosphere. The reaction mixture was stirred until completion (monitored by TLC, 4 h) and then filterd through a pad of celite, followed by concentration under reduced pressure. The resulted residue was purified by silica gel column chromatography (EtOAc/hexanes,1:5) to give hemiacetal as a viscous liquid (1.1 g, 90%). To a solution of the hemiacetal (1.1 g, 2.533 mmol) in anhydrous $CH_2Cl_2$ (15 mL) containing activated 4 Å MS (500 mg) were added trichloroacetonitrile (1.27 mL, 12.669 mmol) and DBU (90 μL, 0.633 mmol) at 0 °C under an argon atmosphere. After stirred for 1 h at room temperature, the reaction mixture was diluted with $CH_2Cl_2$, filtered, and concentrated *in vacuo*. The resulted residue was purified by silica gel column chromatography (EtOAc/*n*-Hexane, 1 : 4, $Et_3N$ 2%) under cold condition to afford the imidate **33** as a viscous liquid (950 mg, 79%).

**Ribitol**

**Scheme S1-S3.** Synthesis of ribitol residue.[a]

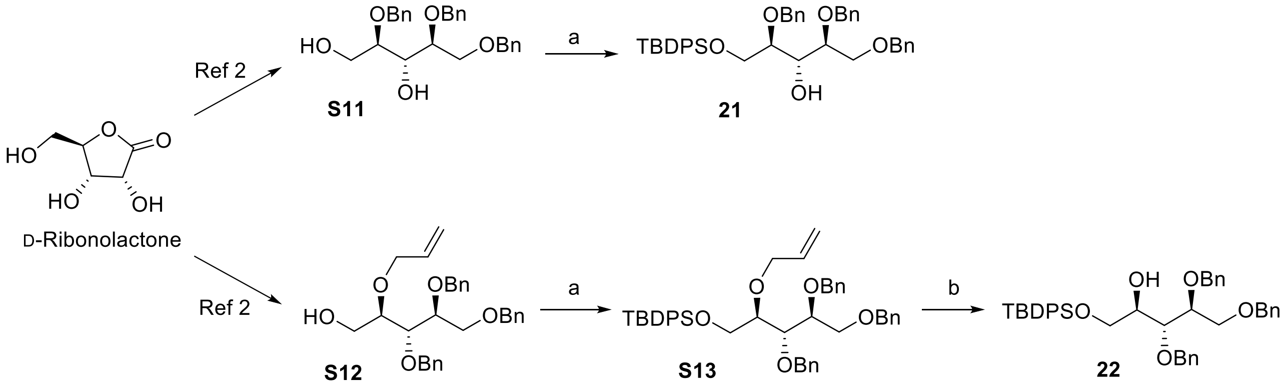

[a]Reagents and conditions: (a) TBDPSCl, imidazole, DMF, rt, 4-6 h, 88% for **21**, 98% for **S13**; (b) $PdCl_2$, $MeOH/CH_2Cl_2$, rt, 3.5 h, 85%.

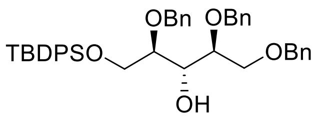

**1,2,4-Tri-*O*-benzyl-5-*O*-*tert*-butyldiphenylsilyl-D-ribitol (21).** To a well-stirred solution of diol **S11** (0.6 g, 1.42 mmol) in DMF (6 mL) were added imidazole (195 mg, 2.84 mmol) and TBDPSCl (0.5 mL, 1.85 mmol) sequentially at room temperature. The reaction mixture was stirred at 35 °C overnight, and the solvent was removed under reduced pressure. The residue was diluted with $CH_2Cl_2$, washed with 1 N HCl, saturated aqueous $NaHCO_3$, and water. The combined organic layer was dried over $MgSO_4$, filtered, and concentrated. The resulted residue was purified by silica gel column chromatography (EtOAc/*n*-Hexane, 1 : 6) to give compound **21** (828 mg, 88%) as a viscous liquid. $^1$H NMR (600 MHz, $CDCl_3$): $\delta_H$ 7.71-7.69 (m, 4H), 7.41-7.26 (m, 21H), 4.71 (t, 2H, $J$ = 11.4 Hz), 4.56-4.50 (m, 4H), 4.11 (q, 1H, $J$ = 5.4 Hz), 3.98 (dd, 1H, $J$ = 11.4, 3.1 Hz), 3.92 (dd, 1H, $J$ = 11.4, 4.8 Hz), 3.81-3.78 (m, 2H), 3.71-3.68 (m, 2H), 3.09 (d, 1H, $J$ = 5.4 Hz), 1.08 (s, 9H). $^{13}$C NMR (150 MHz, $CDCl_3$): $\delta_C$ 138.7, 138.6, 138.3, 135.8 (2), 133.4, 133.2, 129.9 (2), 128.6, 128.5, 128.4, 128.0, 127.9, 127.8 (2), 127.7, 127.6, 79.5, 78.4, 73.6, 72.3, 72.2, 72.0, 70.5, 64.4, 27.0, 19.4. HRMS (ESI-TOF) m/z : calcd for $C_{42}H_{49}O_5Si$ $[M+H]^+$: 661.3344, found: 661.3331.

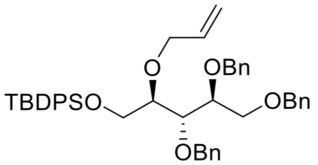

**4-*O*-Allyl-1,2,3-tri-*O*-benzyl-5-*O*-*tert*-butyldiphenylsilyl-D-ribitol (S13).** To a well-stirred solution of alcohol **S12** (1.1 g, 2.38 mmol) in DMF (10 mL) were added imidazole (325 mg, 4.76 mmol) and TBDPSCl (0.8 mL, 3.09 mmol) sequentially at room temperature. The reaction mixture was stirred at 35 °C overnight, and the solvent was removed under reduced pressure. The residue was diluted

with $CH_2Cl_2$, washed with 1 N HCl, saturated aqueous $NaHCO_3$, and water. The combined organic layer was dried over $MgSO_4$, filtered, and concentrated. The resulted residue was purified by silica gel column chromatography (EtOAc/*n*-Hexane, 1 : 6) to give compound **S13** (1.65 g, 98%) as a viscous liquid. $^1$H NMR (600 MHz, $CDCl_3$): $\delta_H$ 7.74-7.72 (m, 4H), 7.45-7.28 (m, 21H), 5.95-5.88 (m, 1H), 5.27-5.24 (m, 1H), 5.16-5.14 (m, 1H), 4.76 (d, 1H, *J* = 11.4 Hz), 4.72 (d, 1H, *J* = 10.8 Hz), 4.68 (t, 2H, *J* = 11.4 Hz), 4.59 (q, 2H, *J* = 12.2 Hz), 4.20-4.17 (m, 1H), 4.07-4.04 (m, 1H), 4.00-3.98 (m, 1H), 3.96-3.88 (m, 3H), 3.83 (dd, 1H, *J* = 3.2, 10.6 Hz), 3.76-3.73 (m, 2H), 1.10 (s, 9H). $^{13}$C NMR (150 MHz, $CDCl_3$): $\delta_C$ 139.0, 138.7 (2), 135.9, 135.8, 135.5, 135.0, 133.8, 133.6, 129.7 (2), 128.5, 128.4, 128.0, 127.9, 127.8 (2), 127.6 (2), 127.5, 116.4, 79.9, 78.9 (2), 73.8, 73.4, 72.6, 71.8, 70.7, 63.9, 27.1, 19.4. HRMS (ESI-TOF) m/z : calcd for $C_{45}H_{52}O_5SiNa$ $[M+Na]^+$: 723.3476, found: 723.3475.

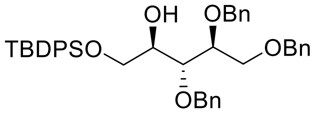


**1,2,3-Tri-*O*-benzyl-5-*O*-*tert*-butyldiphenylsilyl-D-ribitol (22).** To a stirred solution of compound **S13** (1.5 g, 2.14 mmol) in a mixture of MeOH/$CH_2Cl_2$ (10 mL, 1/1) was added $PdCl_2$ (75 mg, 0.427 mmol) at room temperature. The reaction mixture was stirred until disappereance of starting material (monitored by TLC, 3.5 h) and then filtered through a pad of celite, followed by concentration *in vacuo*. The obtained residue was purified by silica gel column chromatography (EtOAc/*n*-Hexane, 1 : 7) to afford compound **22** (1.2 g, 85%) as a viscous liquid. $^1$H NMR (600 MHz, $CDCl_3$): $\delta_H$ 7.71-7.69 (m, 4H), 7.48-7.18 (m, 21H), 4.78 (d, 1H, *J* = 11.4 Hz), 4.73 (d, 1H, *J* = 11.4 Hz), 4.73 (d, 1H, *J* = 12.0 Hz), 4.58 (s, 2H), 4.58 (d, 1H, *J* = 12.0 Hz), 4.05-4.03 (m, 1H), 3.98-3.95 (m, 1H), 3.91 (dd, 1H, *J* = 3.6, 10.8 Hz), 3.86-3.83 (m, 3H), 3.78 (dd, 1H, *J* = 6.0, 10.2 Hz), 1.12 (s, 9H). $^{13}$C NMR (150 MHz, $CDCl_3$): $\delta_C$ 138.7, 138.5, 138.4, 135.8, 135.7, 133.4, 133.3, 129.9, 128.5 (2), 128.4, 128.0, 127.9, 127.8 (2), 127.7, 127.6 (2), 79.3, 79.0, 73.7, 73.5, 72.6, 72.1, 70.1, 65.2, 27.1, 19.4. HRMS (ESI-TOF) m/z : calcd for $C_{42}H_{48}O_5SiNa$ $[M+Na]^+$: 683.3163, found: 683.3163.

**Synthesis of disaccharides**

**Glc-Glc disaccharide**

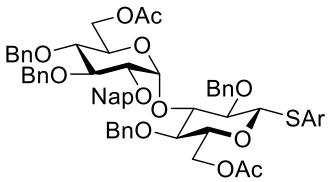


**(2-Methyl-5-*tert*-butylphenyl) 6-*O*-acetyl-3,4-di-*O*-benzyl-2-*O*-(2-napthylmethyl)-α-D-gluco-pyranosyl-(1→3)-6-*O*-acetyl-2,4-di-*O*-benzyl-1-thio-β-D-glucopyranoside (16).** A mixture of donor **14** (9.5 g, 12.928 mmol, 1 equiv), acceptor **15** (4.3 g, 7.757 mmol, 0.6 equiv), and 4 Å MS (15 g) in anhydrous $CH_2Cl_2$ (100 mL) was cooled to under an argon atmosphere before TMSOTf (4.2 mL, 23.271 mmol, 1.8 equiv) was added. The reaction was stirred at –40 °C until completion (monitored

by TLC, 6 h) and then neutralized with saturated aqueous $NaHCO_3$ (7 mL). The reaction residue was warmed to room temperature and filtered. The filtrate was washed with saturated aqueous $NaHCO_3$ and brine, dried over $MgSO_4$, and concentrated *in vacuo*. The residue was purified by silica gel column chromatography (EtOAc/*n*-Hexane, 1 : 4) to give **16** (6.4 g, 75%) as a white foam. $^1$H NMR (600 MHz, $CDCl_3$): $\delta_H$ 7.75 (dd, 1H, *J* = 2.2, 6.1 Hz), 7.63-7.60 (m, 3H), 7.55 (d, 1H, *J* = 2.0 Hz), 7.44-7.38 (m, 4H), 7.33-7.20 (m, 12H), 7.20-7.15 (m, 5H), 7.10 (d, 2H, *J* = 7.7 Hz), 7.08-7.06 (m, 2H), 5.60 (d, 1H, *J* = 3.6 Hz), 5.07 (d, 1H, *J* = 9.7 Hz), 5.00 (d, 1H, *J* = 1.8 Hz), 4.98 (d, 1H, *J* = 2.4 Hz), 4.92 (d, 1H, *J* = 10.8 Hz), 4.89 (d, 1H, *J* = 11.8 Hz), 4.86 (d, 1H, *J* = 10.8 Hz), 4.80 (d, 1H, *J* = 10.1 Hz), 4.76 (d, 1H, *J* = 11.9 Hz), 4.66 (d, 1H, *J* = 9.8 Hz), 4.50 (d, 1H, *J* = 10.8 Hz), 4.43 (d, 1H, *J* = 11.1 Hz), 4.30 (dd, 1H, *J* = 2.1, 11.9 Hz), 4.25-4.22 (m, 1H), 4.13-4.10 (m, 2H), 4.04-4.00 (m, 2H), 3.85 (dd, 1H, *J* = 3.6 12.2 Hz), 3.74 (t, 1H, *J* = 9.4 Hz), 3.61-3.57 (m, 2H), 3.54-3.48 (m, 2H), 2.35 (s, 3H), 1.98 (s, 3H), 1.93 (s, 3H), 1.98 (s, 9H). $^{13}$C NMR (150 MHz, $CDCl_3$): $\delta_C$ 170.8, 170.7, 149.8, 138.6, 138.2, 137.7, 137.6, 136.2, 135.4, 133.3, 133.1, 130.1, 128.6 (3), 128.5, 128.4, 128.3, 128.1, 128.0, 127.9, 127.8, 127.3, 126.8, 126.2, 126.1, 125.9, 124.8, 97.4 (C1 $^1J_{CH}$ = 172 Hz), 88.7 (C1 $^1J_{CH}$ = 155 Hz), 82.4, 81.0, 79.9, 79.8, 78.8, 77.9, 76.6, 75.8, 75.4 (2), 74.2, 74.1, 69.2, 63.4, 62.9, 34.7, 31.5, 21.1, 21.0, 20.5. HRMS (ESI) m/z: calcd for $C_{66}H_{72}O_{12}Na$ $[M+Na]^+$: 1111.4637; found: 1111.4647.

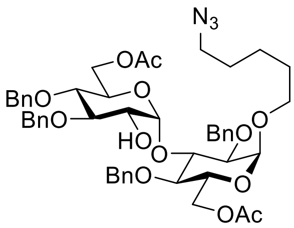


**5-Azidopentyl 6-*O*-acetyl-3,4-di-*O*-benzyl-α-D-glucopyranosyl-(1→3)-6-*O*-acetyl-2,4-di-*O*-benzyl-α-D-glucopyranoside (36).** A mixture of donor **16** (0.9 g, 0.826 mmol), 5-azidopentan-1-ol (213 mg, 1.65 mmol), and freshly activated 4 Å MS (1 g) in anhydrous $CH_2Cl_2$ (25 mL) was stirried under an argon atmosphere at room temperature for 30 min. The mixture was cooled to −30 °C before sequential addition of NIS (280 mg, 1.24 mmol, 1.5 equiv to donor) and TfOH (0.5 M in $Et_2O$, 495 μL, 0.248 mmol, 0.3 equiv to donor). The reaction was stirred at −30 °C until completion (monitored by TLC, 3 h) and then quenched by addition of saturated aqueous $NaHCO_3$ (1 mL). After warmed to room temperature, the reaction mixture was filtered though a pad of celite. The filtrate was washed with saturated aqueous $Na_2S_2O_3$, saturated aqueous $NaHCO_3$, and brine. The separated organic layer was dried over $MgSO_4$, filtered, and concentrated *in vacuo*. The resulted residue was purified by silica gel column chromatography (EtOAc/hexanes, 1:4) to afford inseparable α,β-mixture disaccharide (815 mg, 95%). The afforded α,β-mixture (800 mg, 0.771 mmol) was dissolved in a mixture of $CH_2Cl_2$ and phosphate buffer pH 7 (16.5 mL, 10:1) and cooled to 0-5 °C. DDQ (350 mg, 1.542 mmol) was added in protions over 10 min, and the reaction mixture was stirred at rt until completion (monitored by TLC, 3 h). Then, it was diluted with $CH_2Cl_2$, washed with sat. aq. $NaHCO_3$, water, dried over $MgSO_4$, and concentrated *in vacuo*. The resultant residue was purified by silica gel column chromatography (EtOAc/*n*-Hexane, 1 : 6 to 1 : 5) to afford pure α-isomer **36** (250 mg) and β-isomer

**S14** (120 mg) (α,β = 2:1, 53%). **36**: $^1$H NMR (600 MHz, $CDCl_3$): $\delta_H$ 7.35-7.23 (m, 20H, 5.43 (d, 1H, *J* = 3.9 Hz), 4.95 (d, 1H, *J* = 10.4 Hz), 4.85 (q, 2H, *J* = 11.1 Hz), 4.80 (q, 1H, *J* = 11.0 Hz), 4.77 (d, 1H, *J* = 3.6 Hz), 4.58 (d, 1H, *J* = 11.3 Hz), 4.52 (d, 1H, *J* = 6.1 Hz), 4.50 (d, 1H, *J* = 6.1 Hz), 4.48 (d, 1H, *J* = 10.4 Hz), 4.32-4.29 (2H, m), 4.26 (dd, 1H, *J* = 4.2 Hz), 4.20 (t, 1H, *J* = 9.3 Hz), 4.11 (dd, 1H, *J* = 1.6, 12.1 Hz), 3.92 (dd, 1H, *J* = 3.8, 12.1 Hz), 3.83-3.80 (1H, m), 3.76 (t, 1H, *J* = 9.2Hz), 3.70-3.66 (m, 1H), 3.63-3.56 (m, 2H), 3.48-3.44 (m, 2H), 3.33-3.29 (m, 1H), 3.27-3.24 (m, 2H), 2.45 (d, 1H, *J* = 7.3 Hz), 2.08 (s, 3H), 1.99 (s, 3H), 1.63-1.58 (m, 4H), 1.46-1.41 (m, 2H). $^{13}$C NMR (150 MHz, $CDCl_3$): $\delta_C$ 170.9, 170.8, 138.7, 138.2, 137.7, 137.5, 128.7 (2), 128.6, 128.5, 128.2, 128.1, 128.0, 127.9 (2), 98.6 ($^1J_{CH}$ = 176 Hz), 96.5 ($^1J_{CH}$ = 168 Hz), 83.5, 79.2, 78.3, 77.5, 76.6, 75.6, 74.9, 74.7, 73.0, 72.8, 69.0, 68.6, 68.2, 62.9, 51.4, 29.1, 28.8, 23.7, 21.1. HRMS (ESI) m/z: calcd for $C_{49}H_{59}N_3O_{13}Na$ $[M+Na]^+$: 920.3940, found: 920.3952.

**Rha-Rbo pseudo-disaccharide**

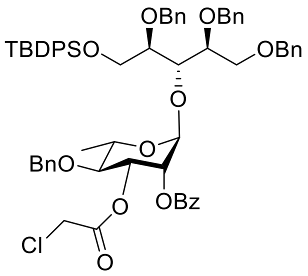


**2-*O*-Benzoyl-4-*O*-benzyl-3-*O*-chloroacetyl-α-L-rhamnopyranosyl-(1→3)-1,2,4-tri-*O*-benzyl-5-*O*-*tert*-butyldiphenylsilyl-D-ribitol (S15).** To a stirred mixture of acceptor **22** (800 mg, 1.211 mmol), donor **33** (1.049 mg, 1.817 mmol), and 4 Å MS (1.5 g) in anhydrous $CH_2Cl_2$ (35 mL) was added TMSOTf (50 µL, 0.273 mmol, 0.15 eq to donor) at –40 °C under an argon atmosphere. The reaction mixture was stirred until completion (monitored by TLC, 45 min). The mixture was then neutralized with satd. aq. $NaHCO_3$ (1 mL), slowly warmup to rt, and filtered. The filtrate was washed with satd. aq. $NaHCO_3$ and water, dried over $MgSO_4$, and concentrated *in vacuo*. The residue was purified by silica gel column chromatography (EtOAc/*n*-Hexane, 1 : 10) to give **S15** as a colorless viscous liquid (1.18 g, 90%). $^1$H NMR (600 MHz, $CDCl_3$): $\delta_H$ 8.03 (d, 2H, *J* = 7.3 Hz), 7.63-7.57 (m, 5H), 7.48 (d, 2H, *J* = 7.8 Hz), 7.32-7.12 (m, 26H), 5.46-5.45 (m, 1H), 5.36 (dd, 1H, *J* = 3.2, 9.7 Hz), 5.15 (s, 1H), 4.65-4.56 (m, 4H), 4.51 (d, 1H, *J* = 12 Hz), 4.44 (d, 1H, *J* = 11.8 Hz), 4.42 (d, 1H, *J* = 12 Hz), 4.36 (d, 1H, *J* = 11.8 Hz), 4.33 (d, 1H, *J* = 4.8 Hz), 4.12-4.09 (m, 1H), 3.93-3.81 (m, 5H), 3.74 (dd, 1H, *J* = 3.4, 10.6 Hz), 3.70 (dd, 1H, *J* = 4.6, 9.4 Hz), 3.67 (dd, 1H, *J* = 5.9, 10.6 Hz), 3.57 (t, 1H, *J* = 9.7 Hz), 1.22 (d, 3H, *J* = 6.2 Hz), 0.98 (s, 9H). $^{13}$C NMR (150 MHz, $CDCl_3$): $\delta_C$ 166.5, 165.6, 138.7, 138.6, 138.5, 138.2, 135.9, 135.8, 133.5, 133.4, 130.0, 129.8, 129.7, 128.7, 128.6, 128.5, 128.4, 128.3, 128.0, 127.9, 127.8, 127.7 (2), 127.6, 127.4, 97.3 (C1, $^1J_{CH}$ = 172 Hz), 79.2, 78.8, 77.9, 76.2, 75.1, 74.3, 73.3, 72.3, 72.2, 70.8, 70.3, 68.4, 62.8, 40.9, 27.0, 19.4, 18.1. HRMS (ESI) m/z: calcd for $C_{64}H_{69}ClO_{11}SiNa$ $[M+Na]^+$: 1099.4190, found: 1099.4200.

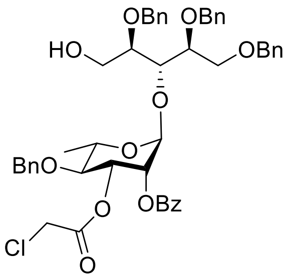


**2-*O*-Benzoyl-4-*O*-benzyl-3-*O*-chloroacetyl-α-L-rhamnopyranosyl-(1→3)-1,2,4-tri-*O*-benzyl-D-ribitol (S16).** To a Nalgene bottle containing a solution of silylether **S15** (1.1 g, 1.021 mmol) in a mixture of THF and pyridine (20 mL, 9/1 *v/v*) was added pyridine hydrofluoride (HF-py) stock solution (16 mL, 15 equiv. The stock solution was prepared from commercially available Aldrich HF-py (70%) by dissolving 15 mL in 30 mL of pyridine and 75 mL of THF.) using plastic syringe over 3 min at 0 °C. The reaction was warmed to room temperature and stirred until completion (monitored by TLC, 10 h). Then, the reaction mixtrure was poured into cold satd. aq. $NaHCO_3$ (300 mL) and extracted with $CH_2Cl_2$ (2 × 75 mL). The combined organic layer was dried over $MgSO_4$, filtered, and concentrated *in vacuo*. The resultant residue was purified by silica gel column chromatography (EtOAc/*n*-Hexane, 1 : 4 to 1 : 3) to afford pure compound **S16** as a colorless viscous liquid (737 mg, 86%). $^1H$ NMR (600 MHz, $CDCl_3$): $\delta_H$ 8.06 (d, 2H, $J$ = 7.4 Hz), 7.64 (t, 1H, $J$ = 7.7 Hz), 7.51 (t, 2H, $J$ = 7.7 Hz), 7.36-7.18 (m, 20H), 5.55 (t, 1H, $J$ = 2.3 Hz), 5.41 (dd, 1H, $J$ = 3.1, 9.6 Hz), 5.23 (d, 1H, $J$ = 1.4 Hz $Rham_{H\text{-}1}$), 4.72-4.64 (m, 4H), 4.58-4.53 (m, 3H), 4.93 (d, 1H, $J$ = 11.9 Hz), 4.25 (t, 1H, $J$ = 4.7 Hz), 4.06-4.03 (m, 1H), 3.95 (d, 1H, $J$ = 14.9 Hz), 3.89-3.82 (m, 3H), 4.11(q, 1H, $J$ = 4.4 Hz), 3.74-3.72 (m, 2H), 3.69 (dd, 1H, $J$ = 5.5, 10.3 Hz), 3.63 (t, 1H, $J$ = 9.6 Hz), 2.16 (brs, 1H), 1.27 (d, 3H, $J$ = 6.2 Hz). $^{13}C$ NMR (150 MHz, $CDCl_3$): $\delta_C$ 166.7, 165.8, 138.3, 138.2, 138.1, 138.0, 133.6, 130.1, 129.6, 129.2, 128.7, 128.6 (2), 128.5 (2), 128.4, 128.2, 128.1, 128.0, 127.9 (2), 127.8 (2), 98.1 ($^1J_{CH}$ = 172.3 Hz), 79.2, 78.7, 78.0, 76.4, 75.3, 74.2, 73.5, 72.6, 72.1, 70.7, 69.7, 68.6, 60.3, 40.9, 18.1. HRMS (ESI) m/z: calcd for $C_{48}H_{52}ClO_{11}$ $[M+H]^+$: 839.3193, found: 839.3200.

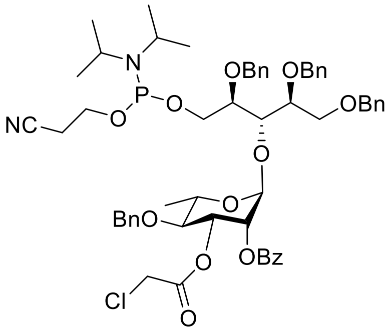


**2-*O*-Benzoyl-4-*O*-benzyl-3-*O*-chloroacetyl-α-L-rhamnopyranosyl-(1→3)-1,2,4-tri-*O*-benzyl-5-([*N*,*N*-diisopropyl]-[2-cyanoethyl]-phosphoroamidite)-D-ribitol (34).** To a soultion of alcohol **S16** (784 mg, 0.934 mmol) in a mixture of anhydrous $CH_2Cl_2$ and $CH_3CN$ (6 mL, 2:1) were added 2-cyanoethyl *N,N,N',N'*-tetraisopropylphosphorodiamidite (1.186 mL, 3.736 mmol) and *N,N*-diisopropylammonium tetrazolide (192 mg, 1.120 mmol) at room temperature under an argon atmosphere. The reaction mixture was stirred until completion (monitored by TLC, 1 h). Then, it was diluted with $CH_2Cl_2$ (15 mL), washed with satd. aq. $NaHCO_3$ (5 mL), dried over $MgSO_4$, filtered,

and concentrated *in vacuo*. The residue was purified by neutral alumina column chromatography (EtOAc/*n*-Hexane, 1 : 3) under cold condition to afford pure compound **34** as a colorless viscous liquid (955 mg, 98%). Mixture of phosphine diastereoisomers: $^1$H NMR (600 MHz, $CDCl_3$): $\delta_H$ 8.02-8.01 (m, 4H), 7.62-7.58 (m, 2H), 7.49-7-45 (m, 4H), 7.34-7.15 (m, 40H), 5.52 (dd, 1H, *J* = 2.0, 3.1 Hz), 5.55 (dd, 1H, *J* = 1.8, 2.8 Hz), 5.36-5.33 (m, 2H), 5.13 (d, 1H, *J* = 1.5 Hz), 5.12 (d, 1H, *J* = 1.5 Hz), 4.68-4.59 (m, 10H), 4.54-4.49 (m, 4H), 4.46 (d, 1H, *J* = 6.0 Hz), 4.44 (d, 1H, *J* = 6.0 Hz), 4.17-4.15 (m, 2H), 4.07-4.06 (m, 2H), 3.97-3.81 (m, 10H), 3.76-3.63 (m, 9H), 3.58-3.49 (m, 5H), 2.49-2.38 (m, 4H), 1.22 (d, 3H, *J* = 1.5 Hz), 1.21 (d, 3H, *J* = 1.4 Hz), 1.11-1.06 (m, 24H). $^{13}$C NMR (150 MHz, $CDCl_3$): $\delta_C$ 166.6, 165.6 (2), 138.6, 138.5, 138.4, 138.3, 138.2, 138.1, 133.7, 133.6, 130.0 (2), 129.7, 128.7 (2), 128.6, 128.5 (2), 128.4 (2), 128.0 (2), 127.8 (2), 127.6 (2), 117.9, 98.2 ($^1J_{CH}$ = 173.3 Hz), 97.8 ($^1J_{CH}$ = 173.3 Hz), 78.7 (2), 78.5 (2), 78.4 (2), 78.2, 78.1, 75.3, 74.3 (2), 73.4, 72.4, 72.3 (2), 70.7 (2), 70.2, 70.1, 68.6 (2), 62.7, 67.6, 62.4, 62.3, 58.8, 58.6, 58.5, 58.4, 43.4, 43.3, 40.9 (2), 24.8, 24.7, 20.3 (2), 20.2 (2), 18.1. $^{31}$P NMR (202 MHz, $CDCl_3$): δ 150.3, 150.0; HRMS (ESI) m/z: calcd for $C_{57}H_{69}ClN_2O_{12}P$ $[M+H]^+$: 1039.4271, found: 1039.4277.

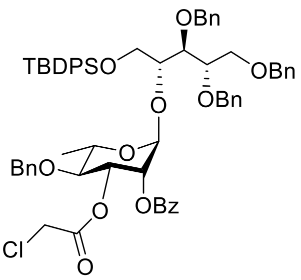


**2-*O*-Benzoyl-4-*O*-benzyl-3-*O*-chloroacetyl-α-L-rhamnopyranosyl-(1→4)-1,2,3-tri-*O*-benzyl-5-*O*-*tert*-butyldiphenylsilyl-D-ribitol (S17).** To a stirred mixture of donor **33** (510 mg, 0.838 mmol), acceptor **35** (467 mg, 0.707 mmol), and 4 Å MS (1 g) in anhydrous $CH_2Cl_2$ (15 mL) was added TMSOTf (30 µL, 0.167 mmol) at –40 °C under an argon atmosphere. The reaction mixture was stirred until completion (monitored by TLC, 45 min). The mixture was then neutralized with satd. aq. $NaHCO_3$ (250 µL), warmed to rt, and filtered. The filtrate was washed with satd. aq. $NaHCO_3$ and water, dried over $MgSO_4$, and concentrated *in vacuo*. The residue was purified by silica gel column chromatography (EtOAc/*n*-Hexane, 1 : 10) to give **S17** as a colorless viscous liquid (710 mg, 93%). $^1$H NMR (600 MHz, $CDCl_3$): $\delta_H$ 8.02 (dd, 2H, *J* = 1.0, 8.1 Hz), 7.59-7.54 (m, 5H), 7.47 (t, 2H, *J* = 8.0 Hz), 7.30-7.16 (m, 26H), 5.68 (dd, 1H, *J* = 1.6, 3.0 Hz), 5.45 (dd, 1H, *J* = 3.2, 9.7 Hz), 5.34 (d, 1H, *J* = 1.4 Hz), 4.64-4.56 (m, 4H), 4.51-4.45 (m, 4H), 4.27-4.25 (m, 1H), 4.09-4.04 (m, 1H), 3.96 (dd, 1H, *J* = 7.5, 11.4 Hz), 3.93 (d, 1H, *J* = 14.8 Hz), 3.85 (d, 1H, *J* = 14.8 Hz), 3.81 (d, 1H, *J* = 3.0, 11.4Hz), 3.77-3.74 (m, 1H), 3.73 (dd, 1H, *J* = 2.9, 6.9 Hz), 3.70 (dd, 1H, *J* = 2.7, 10.5 Hz), 3.64 (dd, 1H, *J* = 4.8, 10.5 Hz), 3.57 (t, 1H, *J* = 9.7 Hz), 1.17 (d, 3H, *J* = 6.1Hz), 0.91 (s, 9H). $^{13}$C NMR (150 MHz, $CDCl_3$): $\delta_C$ 166.5, 165.6, 138.6 (2), 138.3, 138.2, 135.8, 135.7, 133.5, 133.3, 133.1, 130.0, 129.9, 129.8, 128.7, 128.6, 128.5, 128.4, 128.1, 128.0 (2), 127.9, 127.8, 127.7, 127.6, 127.5, 97.6 (C1, $^1J_{CH}$ = 172 Hz), 78.7 (2), 78.3, 77.8, 75.2, 74.5, 73.5, 73.3, 72.8, 70.7, 70.1, 68.3, 64.8, 40.9, 26.9, 19.2, 18.1. HRMS (ESI) m/z: calcd for $C_{64}H_{69}ClO_{11}SiNa$ $[M+Na]^+$: 1099.4190, found: 1099.4176.

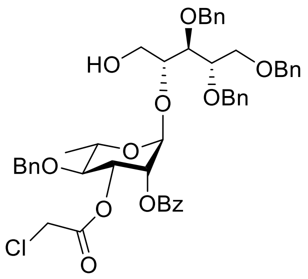


**2-*O*-Benzoyl-4-*O*-benzyl-3-*O*-chloroacetyl-α-L-rhamnopyranosyl-(1→4)-1,2,3-tri-*O*-benzyl-D-ribitol (S18).** To a Nalgene bottle containing a solution of silylether **S17** (600 mg, 0.564 mmol) in a mixture of THF and pyridine (12 mL, 9/1 *v/v*) was added pyridine hydrofluoride (HF-py) stock solution (8.75 mL, 15 equiv. The stock solution was prepared from commercially available Aldrich HF-py (70%) by dissolving 15 mL in 30 mL of pyridine and 75 mL of THF.) using plastic syringe over 3 min at 0 ºC. The reaction was warmed to room temperature and stirred until completion (monitored by TLC, 10 h). Then, the reaction mixtrure was poured into cold satd. aq. $NaHCO_3$ (150 mL) and extracted with $CH_2Cl_2$ (2 × 40 mL). The combined organic layer was dried over $MgSO_4$, filtered, and concentrated *in vacuo*. The resultant residue was purified by silica gel column chromatography (EtOAc/*n*-Hexane, 1 : 4 to 1 : 3) to afford pure compound **S18** as a colorless viscous liquid (388 mg, 82%). $^1$H NMR (600 MHz, $CDCl_3$): $\delta_H$ 8.06 (d, 2H, *J* = 7.2 Hz), 7.62 (t, 1H, *J* = 7.4 Hz), 7.49 (t, 2H, *J* = 7.7 Hz), 7.34-7.24 (m, 20H), 5.59-5.58 (m, 1H), 5.48 (dd, 1H, *J* = 3.2, 9.7 Hz), 5.16 (s, 1H, $Rham_{H-1}$), 4.76 (d, 2H, *J* = 10.6 Hz), 4.70 (d, 1H, *J* = 11.3 Hz), 4.64 (t, 3H, *J* = 12.8 Hz), 4.55 (q, 2H, *J* = 12.2 Hz), 4.11 (q, 1H, *J* = 3.9 Hz), 4.02-3.98 (m, 1H), 3.94-3.90 (m, 2H), 3.86 (d, 1H, *J* = 14.8 Hz), 3.83-3.80 (m, 2H), 3.78 (dd, 1H, *J* = 3.2, 10.4 Hz), 3.75-3.72 (m, 1H), 3.71 (dd, 1H, *J* = 4.7, 10.4 Hz), 3.64 (t, 1H, *J* = 9.6 Hz), 2.51 (brs, 1H), 1.26 (d, 3H, *J* = 6.2 Hz). $^{13}$C NMR (150 MHz, $CDCl_3$): $\delta_C$ 166.6, 165.8, 138.3, 138.2, 138.0, 133.6, 130.0, 129.6, 128.7, 128.6, 128.5, 128.2, 128.0, 127.9 (2), 127.8, 95.9 ($^1J_{CH}$ = 174 Hz), 80.0, 78.6, 78.0, 76.4, 75.3, 74.3, 74.2, 73.6, 72.7, 70.8, 69.4, 68.6, 61.0, 40.8, 18.1. HRMS (ESI) m/z: calcd for $C_{48}H_{52}ClO_{11}$ $[M+H]^+$: 839.3193, found: 839.3216.

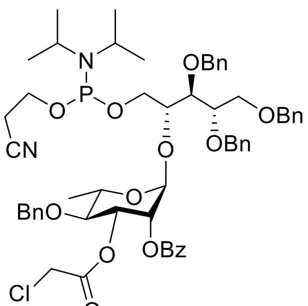


**2-*O*-Benzoyl-4-*O*-benzyl-3-*O*-chloroacetyl-α-L-rhamnopyranosyl-(1→4)-1,2,3-tri-*O*-benzyl-5-([*N*,*N*-diisopropyl]-[2-cyanoethyl]-phosphoroamidite)-D-ribitol (35).** To a soultion of alcohol **S18** (740 mg, 0.881 mmol) in a mixture of anhydrous $CH_2Cl_2$ and $CH_3CN$ (4.5 mL, 2:1) were added 2-cyanoethyl *N,N,N',N'*-tetraisopropylphosphorodiamidite (1.12 mL, 3.526 mmol) and *N,N*-diisopropylammonium tetrazolide (181 mg, 1.057 mmol) at room temperature under an argon atmosphere. The reaction mixture was stirred until completion (monitored by TLC, 1 h). Then, it was

diluted with $CH_2Cl_2$ (15 mL), washed with satd. aq. $NaHCO_3$ (5 mL), dried over $MgSO_4$, filtered, and concentrated *in vacuo*. The residue was purified by neutral alumina column chromatography (EtOAc/*n*-Hexane, 1 : 2) under cold condition to afford pure compound **35** as a colorless viscous liquid (839 mg, 90%). Mixture of phosphine diastereoisomers (1:0.9): $^1$H NMR (600 MHz, $CDCl_3$): $\delta_H$ 8.05 (d, 2H, $J$ = 6.7 Hz), 7.62 (t, 1H, $J$ = 7.4 Hz), 7.49 (t, 2H, $J$ = 7.7 Hz), 7.37 (t, 2H, $J$ = 6.9 Hz), 7.31-7.22 (m, 18H), 5.64 (dd, 1H, $J$ = 1.9, 2.9 Hz), 5.43 (t, 1H, $J$ = 3.1 Hz), 5.24 (d, 1H, $J$ = 1.4, Hz $Rham_{H-1}$), 4.75 (dd, 2H, $J$ = 3.1, 11.1 Hz), 4.66-4.63 (m, 2H), 4.62-4.59 (m, 2H), 5.56 (dd, 1H, $J$ = 3.6, 12.0 Hz), 4.53-4.50 (dd, 1H, $J$ = 3.6, 12.0 Hz), 4.28-4.25 (m, 1H), 4.06-4.02 (m, 1H), 3.92-3.77 (m, 6H), 3.76-3.64 (m, 4H), 3.59 (t, 1H, $J$ = 9.7 Hz), 3.52-3.48 (m, 1H), 3.47-3.42 (m, 1H), 2.51-2.37 (m, 2H), 1.19 (t, 3H, $J$ = 5.9 Hz), 1.10-1.02 (m, 12H). $^{13}$C NMR (150 MHz, $CDCl_3$): $\delta_C$ 166.6, 165.7, 138.6, 138.5, 138.3, 138.1, 133.6, 130.0, 129.7, 128.7, 128.6, 128.5, 128.1, 128.0, 127.9, 127.8, 127.7, 127.6, 117.8, 97.6 ($^1J_{CH}$ = 172 Hz), 78.9, 78.7, 78.4, 77.6, 76.7, 75.3, 74.4, 73.6, 73.5, 72.8, 70.7, 70.0, 68.4, 64.4, 63.7, 58.6, 58.5, 43.3, 43.2, 43.1, 40.8, 24.7, 24.6, 20.3, 20.2, 18.0. $^{31}$P NMR (202 MHz, $CDCl_3$): δ 149.47, 149.43; HRMS (ESI) m/z: calcd for $C_{57}H_{69}ClN_2O_{12}P$ $[M+H]^+$: 1039.4271, found: 1039.4277.

**Synthesis of trisaccharide donor 20**

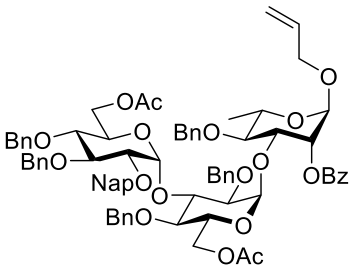


**Allyl 6-*O*-acetyl-3,4-di-*O*-benzyl-2-*O*-(2-napthylmethyl)-α-D-glucopyranosyl-(1→3)-6-*O*-acetyl-2,4-di-*O*-benzyl-α-D-glucopyranosyl-(1→3)-2-*O*-benzoyl-4-*O*-benzyl-α-L-rhamno-pyranoside (18).** A mixture of acceptor **17** (1.28 g, 3.96 mmol), donor **16** (4.4 g, 4.95 mmol), and dried 4 Å MS (8 g) in anhydrous $CH_2CL_2$ (80 mL) was stirred under an argon atmosphere at room temperature for 30 min. The mixture was cooled to −30 °C before NIS (1.34 g, 5.94 mmol, 0.25 equiv with respect to the donor) and TfOH (0.5 M in $Et_2O$, 2.5 mL, 1.24 mmol, 1.2 equiv with respect to the donor) were sequentially added. The reaction was stirred at −30 °C until TLC analysis indicated disappearance of the starting materials (4 h) and quenched with saturated aqueous $NaHCO_3$ (5 mL) before warmed to room temperature. The mixture was filtered though a pad of celite, and the filtrate was washed with saturated aqueous $Na_2S_2O_3$, saturated aqueous $NaHCO_3$, and brine. The organic layer was dried over $MgSO_4$, filtered, and concentrated *in vacuo*. The resulted residue was purified by silica gel column chromatography (EtOAc/*n*-Hexane, 1 : 4) to afford compound **18 (**2.53 g, 60%) as a white foam. $^1$H NMR (600 MHz, $CDCl_3$): $\delta_H$ 7.98 (2H, d, $J$ = 7.5 Hz), 7.71 (1H, d, $J$ = 7.8 Hz), 7.54-7.38 (m, 8H), 7.35 (d, 2H, $J$ = 7.0 Hz), 7.26-6.94 (m, 25H), 5.88-5.82 (m, 1H), 5.55 (t, 1H, $J$ = 2.7 Hz), 5.51 (d, 1H, $J$ = 3.6 Hz), 5.31 (d, 1H, $J$ = 3.5 Hz), 5.27-5.24 (m, 2H), 5.18 (dd, 1H, $J$ = 1.4, 10.3 Hz), 4.97 (d, 1H, $J$ = 10.7 Hz), 4.92-4.89 (m, 3H), 4.83-4.74 (m, 3H), 4.68 (d, 1H, $J$ = 11.5 Hz),

4.43-4.25 (m, 7H), 4.15-4.08 (m, 3H), 4.02-3.96 (m, 3H), 3.93 (dd, 1H, $J$ = 2.0, 12.4 Hz), 3.84 (dd, 1H, $J$ = 6.0, 9.5 Hz), 3.78 (dd, 1H, $J$ = 2.9, 12.3 Hz), 3.69-3.63 (m, 2H), 3.57 (dd, 1H, $J$ = 3.4, 9.6 Hz), 3.52 (dd, 1H, $J$ = 3.5, 10.0 Hz), 3.44 (t, 1H, $J$ = 3.4, 10.0 Hz), 1.99 (s, 3H), 1.90 (s, 3H), 1.37 (d, 3H, $J$ = 6.3 Hz). $^{13}$C NMR (150 MHz, $CDCl_3$): $\delta_C$ 170.9, 170.8, 166.3, 138.7, 138.5, 138.0, 137.8, 137.0, 135.3, 133.7, 133.6, 133.3, 133.1, 130.1, 129.8, 128.7, 128.6 (2), 128.5 (2), 128.4, 128.2, 128.0 (2), 127.9, 127.8, 127.7, 127.6, 127.0, 126.8, 126.1, 126.0, 118.1, 97.7 (C1 $^1J_{CH}$ = 172 Hz), 96.7 (C1 $^1J_{CH}$ = 172 Hz), 91.3 (C1 $^1J_{CH}$ = 168 Hz), 82.5, 79.6, 79.5, 79.2, 77.7, 76.2, 75.8, 75.7, 74.9, 74.2, 73.8, 72.2, 71.8, 68.6 (2), 68.5, 68.3, 63.0, 62.8, 21.1, 21.0, 18.2. HRMS (ESI) m/z: calcd for $C_{78}H_{82}O_{18}Na$ $[M+Na]^+$: 1329.5393; found: 1329.5407.

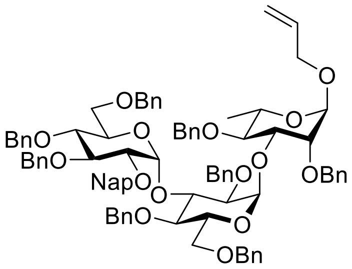


**Allyl 3,4,6-tri-*O*-benzyl-2-*O*-(2-napthylmethyl)-α-D-glucopyranosyl-(1→3)-2,4,6-tri-*O*-benzyl-α-D-glucopyranosyl-(1→3)-2,4-di-*O*-benzyl-α-L-rhamnopyranoside (19).** To a soultion of **18** (2.37 g, 1.759 mmol) in a mixture of MeOH/$CH_2Cl_2$ (12 mL, 3/1) was added NaOMe (5.4 M in MeOH, 163 μL, 0.879 mmol) at room temperature. The reaction mixture was stirred overnight and then neutralized with Amberlite IR-120 ($H^+$). The mixture residue was filtered, concentrated *in vacuo*, and co-evopated twice with toluene. The resulted residue (1.671 g, 1.400 mmol) was dissolved in DMF (15 mL), followed by addition of BnBr (1 mL, 8.401 mmol) and NaH (60%, 280 mg, 7.001 mmol) at 0 °C under an argon atmosphere. The reaction was then warmed to room temperature and kept stirring until completion (monitored by TLC, 4 h). The reaction was quenced with MeOH at 0 °C, poured into ice water, and extracted with $Et_2O$ (30 mL × 3). The combined organice layer was dried over $MgSO_4$ and concentrated *in vacuo*. The resulted residue was purified by silica gel column chromatography (EtOAc/*n*-Hexane, 1 : 10 to 1 : 5) to afford compound **19** as a colorless viscous syrup (1.822 g, 75% yield over two steps). $^1$H NMR (600 MHz, $CDCl_3$): $\delta_H$ 7.75 (d, 1H, $J$ = 7.3 Hz), 7.60 (d, 1H, $J$ = 7.3 Hz), 7.57 (d, 1H, $J$ = 8.5 Hz), 7.52 (s, 1H), 7.54-7.41 (m, 2H), 7.35 (d, 1H, $J$ = 7.3 Hz), 7.29-7.14 (m, 31H), 7.13-7.09 (m, 5H), 7.05 (t, 2H, $J$ = 7.7 Hz), 6.92 (d, 2H, $J$ = 7.7 Hz), 5.88-5.82 (m, 1H), 5.71 (d, 1H, $J$ = 3.4 Hz), 5.27 (d, 1H, $J$ = 3.3Hz), 5.25 (dd, 1H, $J$ = 1.6, 17.5 Hz), 5.16 (dd, 1H, $J$ = 1.5, 10.6 Hz), 4.97 (d, 1H, $J$ = 10.8 Hz), 4.93-4.86 (m, 3H), 4.82-4.77 (m, 4H), 4.71 (d, 1H, $J$ = 12.0 Hz), 4.65 (q, 1H, $J$ = 10.8 Hz), 4.56 (d, 1H, $J$ = 8.0 Hz), 4.54 (d, 1H, $J$ = 6.6 Hz), 4.55-4.53 (m, 2H), 4.43-4.33 (m, 4H), 4.28 (d, 1H, $J$ = 12.0 Hz), 4.15-4.09 (m, 4H), 5.71 (d, 1H, $J$ = 10.0 Hz), 3.94-3.90 (m, 3H), 3.74-3.69 (m, 3H), 3.764-3.61 (m, 2H), 3.46 (dd, 1H, $J$ = 2.5, 11.0 Hz), 3.42 (dd, 1H, $J$ = 1.7, 11.0 Hz), 3.36 (dd, 1H, $J$ = 2.3, 11.0 Hz), 3.30 (dd, 1H, $J$ = 1.7, 10.6, Hz), 1.35(d, 1H, $J$ = 6.2 Hz). $^{13}$C NMR (150 MHz, $CDCl_3$): $\delta_C$ 139.0 (2), 138.7, 138.6, 138.2, 138.1, 138.0, 137.5, 135.6, 134.1, 133.3, 133.1, 129.1, 128.6, 128.5, 128.4 (2), 128.3 (2), 128.2 (2), 128.1, 127.9, 127.8 (3), 127.7, 127.6 (2), 127.5, 127.2, 126.8, 126.7, 126.0, 125.8, 117.2, 97.5 (C1 $^1J_{CH}$ = 172 Hz), 97.2

(C1 $^{1}J_{CH}$ = 172 Hz), 94.5 (C1 $^{1}J_{CH}$ = 171 Hz), 82.5, 80.0, 79.7, 79.1, 78.7, 78.3, 76.4, 75.9, 75.6 (2), 75.0, 73.9, 73.6, 73.5, 73.4, 73.3, 70.5, 70.1, 68.6, 68.3, 68.2, 68.0, 18.1. HRMS (ESI) m/z: calcd for $C_{88}H_{92}O_{15}Na$ [M+Na]$^+$: 1411.6328; found: 1411.6350.

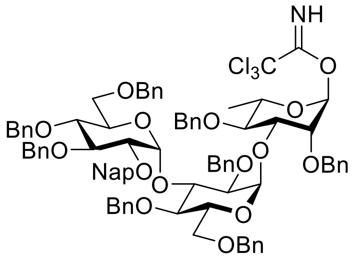


**3,4,6-Tri-*O*-benzyl-2-*O*-(2-napthylmethyl)-α-D-glucopyranosyl-(1→3)-2,4,6-tri-*O*-benzyl-α-D-glucopyranosyl-(1→3)-2,4-di-*O*-benzyl-α-L-rhamnopyranosyl-trichloroacetimidate (20).** To a solution of fully protected trisaccharide **19** (1.79 g, 1.288 mmol) in a mixture of MeOH/$CH_2Cl_2$ (10 mL, 1/1) was added $PdCl_2$ (68.5 mg, 0.386 mmol) at room temperature under an argon atmosphere. The reaction mixture was stirred until completion (monitored by TLC, 5 h), diluted with $CH_2Cl_2$ (15 mL), and filterd through a pad of celite, followed by concentration under reduced pressure. The resulted residue was purified by silica gel column chromatography (EtOAc/hexanes, 1:3 to 1:2) to give hemiacetal as a white foam (1.478 g, 85%). To a solution of the hemiacetal in anhydrous $CH_2Cl_2$ (15 mL) were added trichloroacetonitrile (0.5 mL, 4.380 mmol) and $Cs_2CO_3$ (178 mg, 0.547 mmol) at room temperature under an argon atmosphere. After stirred overnight at room temperature, the reaction mixture was diluted with $CH_2Cl_2$ (10 mL), filtered, and concentrated *in vacuo.* The resulted residue was purified by silica gel column chromatography (EtOAc/*n*-Hexane, 1 : 3 to 1 : 2) to afford the imidate **20** as a white foam (1.25 g, 76%). $^1$H NMR (600 MHz, $CDCl_3$): $\delta_H$ 8.59 (s, 1H, N*H*), 7.77 (d, 1H, *J* = 8.9 Hz), 7.63 (d, 1H, *J* = 8.9 Hz), 7.59 (d, 1H, *J* = 8.4 Hz), 7.54 (s, 1H), 7.48-7.43 (m, 2H), 7.38 (d, 1H, *J* = 8.4 Hz), 7.31-7.20 (m, 25H), 7.19-7.14 (m, 7H), 7.13-7.10 (m, 2H),7.06 (t, 2H, *J* = 7.7 Hz), 6.95 (d, 2H, *J* = 7.2 Hz), 6.32 (d, 1H, *J* = 2.2 Hz), 5.72 (d, 2H, *J* = 3.3 Hz), 5.32 (d, 2H, *J* = 3.5 Hz), 5.00-4.90 (m, 4H), 4.85-4.74 (m, 4H), 4.65-4.55 (m, 5H), 4.52 (t, 1H, *J* = 9.0 Hz), 4.44-4.38 (m, 4H), 4.33 (d, 1H, *J* = 12.0 Hz), 4.20 (dd, 1H, *J* = 3.0, 9.4 Hz), 4.16 (t, 1H, *J* = 2.6 Hz), 4.13 (t, 1H, *J* = 9.4 Hz), 4.09 (d, 1H, *J* = 12.2 Hz), 4.01-3.94 (m, 3H), 3.77-3.70 (m, 3H), 3.65 (dd, 1H, *J* = 3.6, 9.9 Hz), 3.54 (dd, 1H, *J* = 1.9, 10.7 Hz), 3.43 (dd, 1H, *J* = 1.5, 10.7 Hz), 3.35 (dd, 1H, *J* = 2.4, 10.8 Hz), 3.28 (dd, 1H, *J* = 1.6, 10.8 Hz), 1.39 (d, 3H, *J* = 6.2 Hz). $^{13}$C NMR (150 MHz, $CDCl_3$): $\delta_C$ 160.8, 139.0 (2), 138.5, 138.2, 138.0, 137.9, 137.8, 137.5, 135.5, 133.3, 133.1, 129.0, 128.9, 128.7, 128.6, 128.5 (2), 128.4, 128.3, 128.2 (2), 128.1, 128.0, 127.9 (2), 127.8 (2), 127.7, 127.6, 127.5, 127.3, 126.8, 126.7, 126.1, 126.0, 125.9, 97.5, 95.9, 93.6, 91.3, 82.5, 79.6, 78.9, 78.3, 78.2, 75.8, 75.7, 75.6, 75.0, 74.9, 74.0, 73.8, 73.7, 73.5, 73.4, 73.1, 71.4, 70.6, 70.2, 68.3, 68.1, 18.2. HRMS (ESI) m/z: calcd for $C_{87}H_{89}Cl_3NO_{15}$ [M+H]$^+$: 1492.5292; found: 1492.5202.

## Synthesis of pseudo-tetrasaccharides

### Synthesis of pseudo-tetrasaccharides 5, 6, 7, and 8

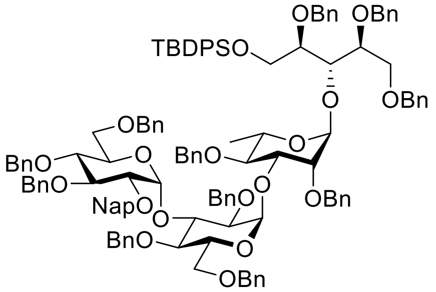


**3,4,6-Tri-*O*-benzyl-2-*O*-(2-napthylmethyl)-α-D-glucopyranosyl-(1→3)-2,4,6-tri-*O*-benzyl-α-D-glucopyranosyl-(1→3)-2,4-di-*O*-benzyl-α-L-rhamnopyranosyl-(1→3)-1,2,4-tri-*O*-benzyl-5-*O*-*tert*-butyldiphenylsilyl-D-ribitol (S19).** To a stirred mixture of imidate donor **20** (995 mg, 0.666 mmol), acceptor **21** (400 mg, 0.605 mmol), and 4 Å MS ( 1.3 g) in anhydrous $CH_2Cl_2$ (15 mL) was added TMSOTf (36 μL, 0.199 mmol ) at –30 °C under an argon atmosphere. The reaction mixture was stirred until completion (monitored by TLC, 1 h). The mixture was then neutralized with satd. aq. $NaHCO_3$ (250 μL), warmed to rt, filtered, and concentrated *in vacuo*. The residue was purified by silica gel column chromatography (EtOAc/*n*-Hexane, 1 : 6) to give **S19** as a white foam (737 mg, 61%) and recovered acceptor **21** (80 mg, 20%). $^1$H NMR (600 MHz, $CDCl_3$): $\delta_H$ 7.77 (d, 1H, $J$ = 7.6 Hz), 7.70 (d, 2H, $J$ = 6.8 Hz), 7.67 (d, 2H, $J$ = 6.8 Hz), 7.61 (d, 1H, $J$ = 7.6 Hz), 7.57 (d, 1H, $J$ = 8.4 Hz), 7.52 (s, 1H), 7.47-7.44 (m, 2H), 7.37-7.33 (m, 4H), 7.29-7.17 (m, 47H), 7.12-7.06 (m, 7H), 6.99 (t, 2H, $J$ = 7.8 Hz), 6.93 (d, 2H, $J$ = 7.4 Hz), 5.72 (d, 1H, $J$ = 3.5 Hz), 5.17 (d, 1H, $J$ = 3.3 Hz), 5.15 (d, 1H, $J$ = 1.3 Hz), 4.98 (d, 1H, $J$ = 10.8 Hz), 4.93-4.86 (m, 3H), 4.83 (d, 1H, $J$ = 9.8 Hz), 4.83 (d, 1H, $J$ = 8.9 Hz), 4.73 (d, 1H, $J$ = 11.9 Hz), 4.63 (t, 2H, $J$ = 11.2 Hz), 4.56-4.50 (m, 7H), 4.49-4.34 (m, 7H), 4.25-4.421 (m, 2H), 4.17 (d, 1H, $J$ = 11.9 Hz), 4.13-4.09 (m, 2H), 4.05-4.01 (m, 2H), 3.95-3.92 (m, 2H), 3.89-3.82 (m, 4H), 3.75-3.69 (m, 4H), 3.65-3.59 (m, 3H), 3.39-3.29 (m, 4H), 1.26 (d, 3H, $J$ = 6.1 Hz), 1.08 (s, 9H). $^{13}$C NMR (150 MHz, $CDCl_3$): $\delta_C$ 139.0, 138.8, 138.7, 138.6, 138.3, 138.2, 138.0, 137.5, 135.9, 135.8, 135.6, 133.6, 133.5, 133.3, 133.1, 129.9, 129.8, 128.9, 128.6, 128.5 (2), 128.4 (2), 128.3 (3), 128.2 (2), 128.1, 128.0, 127.9 (2), 127.8 (3), 127.7 (2), 127.6 (2), 127.5 (2), 127.4, 127.2, 126.8, 126.7, 126.1, 126.0, 125.9, 97.5 (two signals overlapped, $^1J_{CH}$ = 173, 172 Hz), 93.1 (C1, $^1J_{CH}$ = 172 Hz), 82.5, 80.2, 79.6, 79.4, 79.1, 78.4, 78.3, 78.1, 76.0, 75.7, 75.5, 75.4, 74.9, 74.0, 73.5, 73.4 (2), 73.3, 73.2, 72.9, 72.7, 72.3, 70.4, 70.3, 70.1, 69.3, 68.3, 68.3, 64.3, 27.1, 19.4, 18.2. HRMS (ESI) m/z: calcd for $C_{127}H_{135}O_{19}Si$ $[M+H]^+$: 1991.9361, found: 1991.9393.

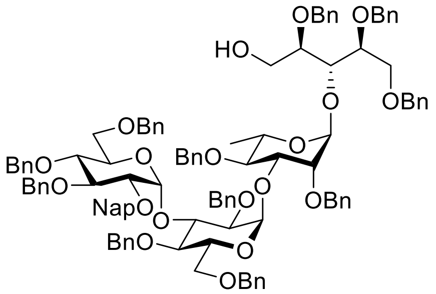

**3,4,6-Tri-*O*-benzyl-2-*O*-(2-napthylmethyl)-α-D-glucopyranosyl-(1→3)-2,4,6-tri-*O*-benzyl-α-D-glucopyranosyl-(1→3)-2,4-di-*O*-benzyl-α-L-rhamnopyranosyl-(1→3)-1,2,4-tri-*O*-benzyl-D-ribitol (23).** To a stirred solution of **S19** (720 mg, 0.361 mmol) in THF (7 mL) was added tetrabutyl ammonium fluoride (TBAF) in THF (1 M, 1.084 mL, 1.084 mmol) at room temperature. The reaction mixture was stirred until completion (monitored by TLC, 5 h). Then, it was diluted with EtOAc (15 mL), washed with satd. aq. $NH_4Cl$ (2 × 10 mL), dried over $MgSO_4$, filtered, and concentrated *in vacuo*. The residue was purified by silica gel column chromatography (EtOAc/*n*-Hexane, 1 : 3) to give **23** as a white foam (600 mg, 95%). $^1$H NMR (600 MHz, $CDCl_3$): $\delta_H$ 7.74 (d, 1H, *J* = 7.5 Hz), 7.59 (d, 1H, *J* = 7.8 Hz), 7.55 (d, 1H, *J* = 8.3 Hz), 7.55 (s, 1H), 7.44-7.39 (m, 2H), 7.35 (d, 2H, *J* = 7.7 Hz), 7.31-7.07 (m, 50H), 7.00 (t, 2H, *J* = 7.8 Hz), 6.91 (d, 2H, *J* = 7.8 Hz), 5.69 (d, 1H, *J* = 3.5 Hz), 5.24 (d, 1H, *J* = 3.1 Hz), 5.19 (s, 1H), 4.96 (d, 1H, *J* = 11.0 Hz), 4.91-4.88 (m, 3H), 4.80 (d, 1H, *J* = 11.9 Hz), 4.78 (d, 1H, *J* = 11.0 Hz), 4.71 (t, 2H, *J* = 11.9 Hz), 4.61 (d, 1H, *J* = 11.5 Hz), 4.55-4.36 (m, 14H), 4.33 (d, 1H, *J* = 11.8 Hz), 4.20-4.16 (m, 2H), 4.10-4.04 (m, 3H), 4.00 (d, 1H, *J* = 9.9 Hz), 3.94 (s, 1H), 3.90 (t, 1H, *J* = 9.5 Hz), 3.83-3.80 (m, 1H), 3.77 (q, 1H, *J* = 5.6 Hz), 3.74-3.67 (m, 4H), 3.65-3.56 (m, 5H), 3.42 (d, 1H, *J* = 10.9 Hz), 3.34-3.31 (m, 2H), 3.27 (d, 1H, *J* = 10.9 Hz), 2.23 (brs, 1H), 1.27 (d, 3H, *J* = 6.1 Hz). $^{13}$C NMR (150 MHz, $CDCl_3$): $\delta_C$ 139.0 (2), 138.6, 138.5, 138.6, 138.3, 138.2 (2), 138.1, 137.9, 137.6, 135.5, 133.3, 133.1, 128.9, 128.7, 128.6 (2), 128.5 (2), 128.4 (2), 128.3 (3), 128.2, 128.1 (2), 128.0, 127.9, 127.8 (2), 127.7 (2), 127.6 (2), 127.5, 127.2, 126.7, 126.0 (2), 125.8, 98.9, 97.5, 93.1, 82.5, 79.7, 79.6, 79.4, 79.0, 78.4, 78.3, 76.8, 75.6, 75.1, 74.9, 74.8, 73.9, 73.5, 73.4 (2), 73.1, 72.9, 72.5, 72.1, 70.3, 70.1, 69.9, 68.2, 60.6, 18.2. HRMS (ESI) m/z: calcd for $C_{111}H_{117}O_{19}$ $[M+H]^+$: 1753.8184, found: 1753.8240.

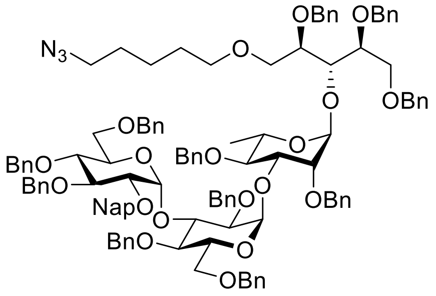


**3,4,6-Tri-*O*-benzyl-2-*O*-(2-napthylmethyl)-α-D-glucopyranosyl-(1→3)-2,4,6-tri-*O*-benzyl-α-D-glucopyranosyl-(1→3)-2,4-di-*O*-benzyl-α-L-rhamnopyranosyl-(1→3)-5-*O*-(5-azidopentyl)-1,2,4-tri-*O*-benzyl-D-ribitol (25).** To a stirred mixture of alcohol **23** (500 mg, 0.285 mmol), 5-azidopentyl methanesulfonate (120 mg, 0.57 mmol) in DMSO (5 mL) was added NaH (60%, 34 mg, 0.855 mmol ) at 0 °C under an argon atmosphere. The ice bath was removed, and the reaction mixture was stirred ar rt until completion (monitored by TLC, 20 h). Then, it was quenched with satd. aq. $NH_4Cl$ (3 mL), poured into ice cold water, and extracted with $Et_2O$ (3 × 15 mL). The combined organic layer was dried over $MgSO_4$, filtered, and concentrated *in vacuo*. The obtained residue was purified by silica gel column chromatography (EtOAc/*n*-Hexane, 1 : 3) to afford pure compound **25** as a colorless viscous liquid (489 mg, 92%). $^1$H NMR (600 MHz, $CDCl_3$): $\delta_H$ 7.70 (d, 1H, *J* = 7.5 Hz), 7.59 (d, 1H, *J* = 7.6 Hz), 7.55 (d, 1H, *J* = 8.5 Hz), 7.50 (s, 1H), 7.44-7.39 (m, 2H), 7.33-7.06 (m,

52H), 6.99 (t, 2H, $J$ = 7.6 Hz), 6.90 (d, 2H, $J$ = 7.3 Hz), 5.69 (d, 1H, $J$ = 3.5 Hz), 5.22 (d, 1H, $J$ = 3.5 Hz), 5.17 (d, 1H, $J$ = 1.7 Hz), 4.96 (d, 1H, $J$ = 10.9 Hz), 4.91-4.87 (m, 3H), 4.80 (d, 1H, $J$ = 12.5 Hz), 4.78 (d, 1H, $J$ = 8.0 Hz), 4.71 (d, 1H, $J$ = 8.3 Hz), 4.69 (d, 1H, $J$ = 8.1 Hz), 4.64 (d, 1H, $J$ = 11.5 Hz), 4.59-4.52 (m, 7H), 4.49-4.43 (m, 3H), 4.40-4.30 (m, 5H), 4.20 (d, 1H, $J$ = 12.0 Hz), 4.12 (quin, 1H, $J$ = 3.8, 6.0 Hz), 4.09 (d, 1H, $J$ = 2.9 Hz), 4.07 (s, 1H), 4.05 (dd, 1H, $J$ = 2.6, 9.6 Hz), 4.01 (td, 1H, $J$ = 2.0, 4.1 Hz), 3.93-3.88 (m, 3H), 3.85-3.80 (m, 1H), 3.74-3.67 (m, 4H), 3.64-3.56 (m, 4H), 3.53 (dd, 1H, $J$ = 5.9, 10.6 Hz), 3.42 (dd, 1H, $J$ = 2.4, 11.0 Hz), 3.35-3.31 (m, 4H), 3.28 (dd, 1H, $J$ = 1.6, 10.6 Hz), 3.18 (t, 2H, $J$ = 7.0 Hz), 1.56-1.51 (m, 4H), 1.39-1.36 (m, 2H), 1.25 (d, 3H, $J$ = 6.1 Hz). $^{13}$C NMR (150 MHz, $CDCl_3$): $\delta_C$ 139.0, 138.9, 138.7, 138.6 (2), 138.5, 138.2, 137.9, 137.6, 135.5, 133.3, 133.0, 128.9, 128.7, 128.5 (2), 128.4 (3), 128.3 (3), 128.1 (2), 128.0 (3), 127.9, 127.8, 127.7 (3), 127.6 (2), 127.5, 127.4, 127.2, 126.7 (2), 126.0 (2), 125.8, 98.7 (C1, $^1J_{CH}$ = 172 Hz), 97.5 (C1, $^1J_{CH}$ = 172 Hz), 93.3 (C1, $^1J_{CH}$ = 172 Hz), 82.5, 79.6, 79.4, 79.0, 78.5, 78.4, 78.3, 78.2, 77.6, 75.7, 75.6, 75.5, 75.4, 75.1, 74.9, 79.9, 73.5, 73.5, 73.4, 73.2, 72.6 (2), 72.5, 71.3, 70.8, 70.4, 70.3, 70.1, 69.3, 68.3, 68.2, 51.5, 29.5, 28.8, 23.6, 18.2. HRMS (ESI) m/z: calcd for $C_{116}H_{125}N_3O_{19}Na$ [M+Na]$^+$: 1886.8799, found: 1886.8800.

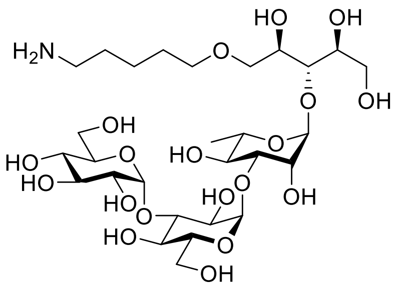


**α-D-Glucopyranosyl-(1→3)-α-D-glucopyranosyl-α-L-rhamnopyranosyl-(1→3)-5-*O*-(5-aminopentyl)-D-ribitol (5).** *Prepared according to general deprotection procedure A* using **25** (100 mg, 0.054 mmol), mixture of solvents $MeOH/H_2O/CH_2Cl_2/AcOH$ (5.25 mL) and $Pd(OH)_2/C$ (100 mg). Reaction time was 36 h. Compound **5** was obtained as an amorphous white solid (36 mg, 84%) after Sephadex LH-20 chromatography. $^1$H NMR (600 MHz, $D_2O$): $\delta_H$ 5.39 (d, 1H, $J$ = 3.8 Hz), 5.11 (d, 1H, $J$ = 3.7 Hz), 5.04 (d, 1H, $J$ = 1.5 Hz), 4.22 (t, 1H, $J$ = 2.2 Hz), 4.11-4.09 (m, 1H), 4.04 (ddd, 2H, $J$ = 3.4. 7.2, 10.1 Hz), 3.96-3.93 (m, 2H), 3.90-3.75 (m, 9H), 3.73-3.63 (m, 4H), 3.62-3.54 (m, 5H), 3.50 (t, 1H, $J$ = 9.7 Hz), 3.04 (t, 2H, $J$ = 7.4 Hz), 1.74-1.64 (m, 4H), 1.49-1.44 (m, 2H), 1.34 (d, 3H, $J$ = 6.2 Hz). $^{13}$C NMR (150 MHz, $D_2O$): $\delta_C$ 100.1 (C1, $^1J_{CH}$ = 171 Hz), 99.2 (C1, $^1J_{CH}$ = 171 Hz), 95.6 (C1, $^1J_{CH}$ = 171 Hz), 79.8, 79.6, 75.3, 72.8, 71.7, 71.6 (2), 71.4, 71.0, 70.8, 70.1, 69.9, 69.8, 69.4, 69.3, 69.0, 66.9, 62.6, 60.2, 59.9, 39.3, 28.1, 26.5, 22.3, 16.6. HRMS (ESI) m/z: calcd for $C_{28}H_{53}NO_{19}Na$ [M+Na]$^+$: 730.3104, found: 730.3091.

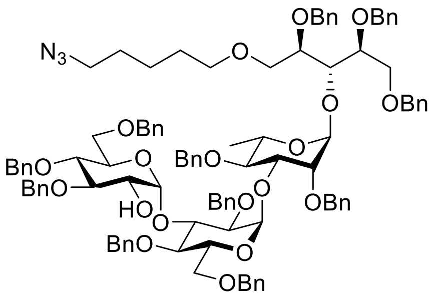


**3,4,6-Tri-*O*-benzyl-α-D-glucopyranosyl-(1→3)-2,4,6-tri-*O*-benzyl-α-D-glucopyranosyl-(1→3)-2,4-di-*O*-benzyl-α-L-rhamnopyranosyl-(1→3)-5-*O*-(5-azidopentyl)-1,2,4-tri-*O*-benzyl-D-ribitol (26).** To a solution of substrate **25** (240 mg, 0.128 mmol) in a mixture of $CH_2Cl_2$ and $H_2O$ (6.3 mL, 20:1) were added 2,6-ditertiary butyl pyridine (179 µL, 0.772 mmol) and DDQ (992 mg, 0.386 mmol) at 0-5 °C. The reaction mixture was stirred for 2.5 h, and then diluted with $CH_2Cl_2$ (25 mL), washed with satd. aq. $NaHCO_3$, water, dried over $MgSO_4$, filtered, and concentrated *in vacuo.* The obtained residue was purified by silica gel column chromatography (EtOAc/*n*-Hexane, 1 : 5 to 1 : 4) to give **26** as a colorless viscous liquid (100 mg, 45%) and recovered starting material **25** (23 mg, 10%). $^1H$ NMR (600 MHz, $CDCl_3$): $\delta_H$ 7.31-7.21 (m, 33H), 7.20-7.16 (m, 9H), 7.15-7.11 (m, 4H), 7.10-7.07 (m, 7H), 7.04 (t, 2H, *J* = 7.6 Hz), 5.54 (d, 1H, *J* = 3.3 Hz), 5.18 (d, 1H, *J* = 3.4 Hz), 5.15 (d, 1H, *J* = 1.6 Hz), 4.85 (d, 1H, *J* = 10.8 Hz), 4.82 (d, 1H, *J* = 10.8 Hz), 4.79 (s, 2H), 4.75 (d, 1H, *J* = 11.1 Hz), 4.70 (d, 1H, *J* = 11.6 Hz), 4.64 (d, 1H, *J* = 11.6 Hz), 4.59-4.49 (m, 8H), 4.45-4.34 (m, 6H), 4.26 (d, 1H, *J* = 11.9 Hz), 4.17 (td, 1H, *J* = 2.0, 10.2 Hz), 4.11-4.08 (m, 2H), 4.02 (dd, 1H, *J* = 2.6, 9.5 Hz), 3.98 (td, 1H, *J* = 2.0, 10.2 Hz), 3.90-3.87 (m, 2H), 3.84-3.78 (m, 2H), 3.75-3.69 (m, 4H), 3.66-3.59 (m, 3H), 3.58-3.55 (m, 2H), 3.52 (dd, 1H, *J* = 5.9, 10.5 Hz), 3.48 (dd, 1H, *J* = 2.4, 11.1 Hz), 3.37-3.32 (m, 4H), 3.26 (dd, 1H, *J* = 1.6, 10.7 Hz), 3.18 (t, 2H, *J* = 6.9 Hz), 2.48 (d, 1H, *J* = 7.3 Hz), 1.56-1.51 (m, 4H), 1.39-1.36 (m, 2H), 1.25 (d, 3H, *J* = 6.2 Hz). $^{13}C$ NMR (150 MHz, $CDCl_3$): $\delta_C$ 139.0, 138.9, 138.7, 138.6, 138.5, 138.2, 138.1, 137.7, 137.6, 128.8, 128.5 (2), 128.4 (3), 128.3, 128.2, 128.1, 128.0 (2), 127.9, 127.8 (3), 127.7 (2), 127.6 (2), 127.5 (2), 98.9, 98.7, 93.5, 83.6, 79.4, 78.8, 78.6, 78.3, 78.2, 77.7, 77.6, 76.7, 75.6, 75.4, 75.3, 74.7, 74.3, 73.6, 73.5 (2), 73.2, 73.1, 72.7, 72.6, 72.5, 71.3, 70.9, 70.6, 70.4, 70.3, 69.3, 68.3, 68.2, 51.5, 29.5, 28.8, 23.6, 18.2. HRMS (ESI) m/z: calcd for $C_{105}H_{118}N_3O_{19}$ $[M+H]^+$: 1724.8354, found: 1724.8409.

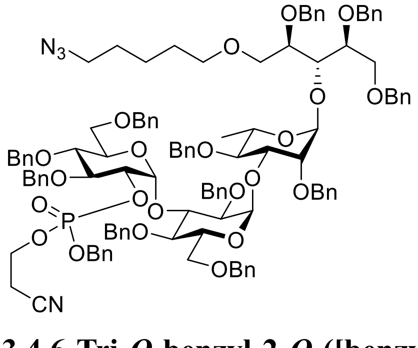


**3,4,6-Tri-*O*-benzyl-2-*O*-([benzyloxy]-[2-cyanoethoxy]-phosphono)-α-D-glucopyranosyl-(1→3)-2,4,6-tri-*O*-benzyl-α-D-glucopyranosyl-(1→3)-2,4-di-*O*-benzyl-α-L-rhamnopyranosyl-(1→3)-5-*O*-(5-azidopentyl)-1,2,4-tri-*O*-benzyl-D-ribitol (29).** To a stirred mixture of alcohol **26** (159 mg,

0.092 mmol) and 5-ethylthio-1*H*-tetrazole (145 mg, 1.10 mmol) in anhydrous $CH_3CN$ (6 mL) under an argon atmosphere was added freshly activated 3 Å MS (300 mg) at rt. After being stirred for 5-10 min, benzyl 2-cyanoethyl *N*,*N*-diisopropylphosphoramidite (113 mg, 0.37 mmol) in anhydrous $CH_3CN$ (2 mL) was added at rt, and the reaction mixture was stirred for 2 h (monitored by TLC). Then, it was cooled to –20 °C, followed by *m*-CPBA (318 mg, 1.84 mmol) addition. After being stirred for 1 h, the reaction mixture was warmed to room temperature, diluted with $CH_2Cl_2$, washed with satd. aq. $NaHCO_3$ (10 mL), satd. aq. $Na_2S_2O_3$ (10 mL), and water. The organic layer was dried over $MgSO_4$, filtered, and concentrated *in vacuo*. The residue was purified by silica gel column chromatography (EtOAc/*n*-Hexane, 1 : 4 to 1 : 2) to give **29** as a colorless viscous liquid (142 mg, 79%, mixture of diastereomers 1:0.9). $^1$H NMR (600 MHz, $CDCl_3$): $\delta_H$ 7.40-7.04 (m, 120H), 5.79 (d, 1H, *J* = 3.4 Hz), 5.77 (d, 1H, *J* = 3.4 Hz), 5.23 (d, 1H, *J* = 3.4 Hz), 5.22 (d, 1H, *J* = 3.4 Hz), 5.19 (s, 2H), 5.02-4.93 (m, 5H), 4.89-4.79 (m, 8H), 4.74-4.71 (m, 3H), 4.68-4.52 (m, 18H), 4.51-4.36 (m, 16H), 4.28 (d, 1H, *J* = 2.8 Hz), 4.26 (d, 1H, *J* = 2.9 Hz), 4.20 (d, 1H, *J* = 5.7 Hz), 4.18 (d, 1H, *J* = 5.8 Hz), 4.15-4.13 (m, 2H), 4.11-4.02 (m, 6H), 3.94-3.92 (m, 2H), 3.90-3.89 (m, 2H), 3.88-3.82 (m, 4H), 3.81-3.971 (m, 8H), 3.70-3.59 (m, 10H), 3.56-3.48 (m, 6H), 3.42-3.37 (m, 8H), 3.22 (t, 4H, *J* = 6.8 Hz), 2.06-1.91 (m, 4H), 1.61-1.56 (m, 8H), 1.44-1.39(m, 4H), 1.28 (t, 6H, *J* = 6.2 Hz). $^{13}$C NMR (150 MHz, $CDCl_3$): $\delta_C$ 138.7, 138.6 (4), 138.5 (2), 138.4, 138.2, 138.1, 137.9, 137.8, 137.6, 135.6 (2), 135.5, 128.8, 128.7 (2), 128.6 (3), 128.5 (3), 128.4 (2), 128.3, 128.2 (3), 128.0 (4), 127.9 (2), 127.8, 127.7 (2), 127.6, 127.5 (2), 127.4, 116.5, 116.4, 98.6 (2), 96.6 (2), 93.2, 80.2 (2), 79.6, 79.5, 78.8, 78.7, 78.6, 78.5, 78.4, 78.3, 78.1, 77.8, 77.7, 77.6, 76.3, 75.8, 75.7, 75.6, 75.5 (2), 75.4, 75.2, 75.1, 73.7 (2), 73.6 (2), 73.3, 73.2, 72.6 (2), 72.5, 72.4, 71.4, 70.8, 70.6, 70.5, 70.4, 70.1 (2), 70.0, 69.9 (2), 69.2, 68.3 (2), 68.2, 61.8 (2), 51.5, 29.5, 28.8, 23.6, 18.9, 18.8, 18.1. $^{31}$P NMR (202 MHz, $CDCl_3$): δ -0.441, -0.523. HRMS (ESI) m/z: calcd for $C_{115}H_{128}N_4O_{22}$ $[M+H]^+$: 1947.8752, found: 1947.8754.

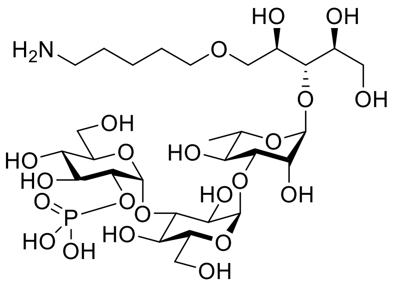


**2-*O*-Phosphono-α-D-glucopyranosyl-(1→3)-α-D-glucopyranosyl-(1→3)-α-L-rhamnopyranosyl-(1→3)-5-*O*-(5-aminopentyl)-D-ribitol (6).** *Prepared according to general deprotection procedure B* using phosphosugar **29** (142 mg, 0.073 mmol), mixture of solvents $CH_2Cl_2/H_2O$ (2 mL), and tetrabutylammonium hydroxide (95 µL, 0.146 mmol). Reaction time was 5 h. Second step with mixture of solvents $MeOH/H_2O/AcOH$ (4.25 mL) and $Pd(OH)_2/C$ (142 mg). Reaction time was 36 h. Compound **6** was obtained as an amorphous white solid (43 mg, 75% over two steps) after Sephadex LH-20 chromatography. $^1$H NMR (600 MHz, $D_2O$): $\delta_H$ 5.62 (s, 1H), 5.10 (d, 1H, *J* = 3.7 Hz), 5.03 (d, 1H, *J* = 1.2 Hz), 4.21 (t, 1H, *J* = 2.1 Hz), 4.10-4.08 (m, 1H), 4.06 (ddd, 1H, *J* =2.9, 5.7, 10.1 Hz), 4.01-3.93 (m, 4H), 3.90-3.75 (m, 10H), 3.72-3.65 (m, 3H), 3.63-3.54 (m, 5H), 3.04 (t, 2H, *J* = 7.5 Hz), 1.74-1.64 (m, 4H), 1.48-1.43 (m, 2H), 1.33 (d, 3H, *J* = 6.4 Hz). $^{13}$C NMR (150 MHz, $D_2O$): $\delta_C$

100.2 (C1, $^{1}J_{CH}$ = 172 Hz), 97.5 (C1, $^{1}J_{CH}$ = 172 Hz), 95.6 (C1, $^{1}J_{CH}$ = 172 Hz), 80.1, 79.5, 75.3, 74.6, 72.1 (2), 71.7, 71.3, 71.2, 71.0, 70.8, 70.0, 69.6, 69.5, 69.4, 69.0, 68.9, 66.9, 62.6, 60.0 (2), 39.3, 28.0, 26.5, 22.3, 16.6. $^{31}$P NMR (202 MHz, $D_2O$): δ 1.06. HRMS (ESI) m/z: calcd for $C_{28}H_{55}NO_{22}P$ [M+H]$^+$: 788.2948, found: 788.2978.

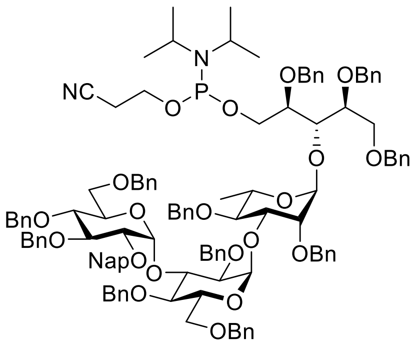


**3,4,6-Tri-*O*-benzyl-2-*O*-(2-napthylmethyl)-α–D-glucopyranosyl-(1→3)-2,4,6-tri-*O*-benzyl-α-D-glucopyranosyl-(1→3)-2,4-di-*O*-benzyl-α-L-rhamnopyranosyl-(1→3)-1,2,4-tri-*O*-benzyl-5-([*N*,*N*-diisopropyl]-[2-cyanoethyl]-phosphoroamidite)-D-ribitol (S20).** To a soultion of alcohol **23** (175 mg, 0.099 mmol) in a mixture of anhydrous $CH_2Cl_2$ and $CH_3CN$ (3 mL, 2:1) were added 2-cyanoethyl *N,N,N',N'*-tetraisopropylphosphorodiamidite (127 μL, 0.399 mmol) and *N,N*-diisopropyl-ammonium tetrazolide (190 mg, 0.197 mmol) at room temperature under an argon atmosphere. The reaction mixture was stirred until completion (monitored by TLC, 1 h). Then, it was diluted with $CH_2Cl_2$ (15 mL), washed with satd. aq. $NaHCO_3$ (5 mL), dried over $MgSO_4$, filtered, and concentrated *in vacuo*. The obtained residue was purified by neutral alumina ($Al_2O_3$) column chromatography (EtOAc/*n*-Hexane, 1 : 3) under cold condition to afford pure compound **S20** as a pale yellow viscous liquid (155 mg, 80%, mixture of diastereomers). $^{1}$H NMR (600 MHz, $CDCl_3$): $\delta_H$ 7.72 (d, 1H, *J* = 7.7 Hz), 7.57 (d, 1H, *J* = 7.7 Hz), 7.53 (d, 1H, *J* = 8.4 Hz), 7.48 (s, 1H), 7.43-7.38 (m, 2H), 7.32-7.03 (m, 52H), 6.98-6.95 (m, 2H), 6.88 (dd, 2H, *J* = 2.4, 7.2 Hz), 5.68 (t, 1H, *J* = 3.2 Hz), 5.23 (dd, 1H, *J* = 3.3, 13.3 Hz), 5.16 (s, 1H), 4.94-4.82 (m, 4H), 4.78 (t, 2H, *J* = 11.8 Hz), 4.69-4.63 (m, 3H), 4.60-4.56 (m, 4H), 4.54-4.43 (m, 5H), 4.42-4.28 (m, 5H), 4.19-4.11 (m, 2H), 4.08-3.99 (m, 4H), 3.96-3.76 (m, 6H), 3.72-3.64 (m, 4H), 3.63-3.53 (m, 4H), 3.42-3.24 (m, 4H), 2.44-2.30 (m, 2H), 1.23-1.21 (m, 3H), 1.19-1.12 (m, 12H). $^{13}$C NMR (150 MHz, $CDCl_3$): $\delta_C$ 139.0, 138.7, 138.6 (3), 138.5, 138.2, 138.0, 137.9, 137.6, 135.5, 133.3, 133.0, 129.0, 128.9 (2), 128.7, 128.6, 128.5, 128.4 (2), 128.3 (2), 128.1, 128.0 (2), 127.9 (2), 127.8 (2), 127.7, 127.6 (2), 127.5 (2), 127.4, 127.2, 126.7, 126.0 (2), 125.8, 117.9, 98.3 (2), 97.5, 93.3, 93.2, 82.5, 79.6, 79.4, 79.3, 79.2 (2), 79.0, 78.4 (2), 78.3, 76.8, 76.7, 75.7, 75.6, 75.5 (2), 75.4, 75.3, 75.2, 74.9, 74.0, 73.5, 73.4, 73.3, 72.7, 72.6, 72.5, 72.4, 70.3 (3), 70.1, 69.4, 69.3, 68.3 (2), 68.2, 63.5, 63.4, 63.3, 60.2, 58.5, 58.4, 58.3, 43.4, 43.3, 24.9 (2), 24.8 (2), 20.4 (2), 18.2. $^{31}$P NMR (202 MHz, $CDCl_3$): δ 150.12. HRMS (ESI) m/z: calcd for $C_{120}H_{134}N_2O_{20}$ [M+H]$^+$: 1953.9262, found: 1953.9278.

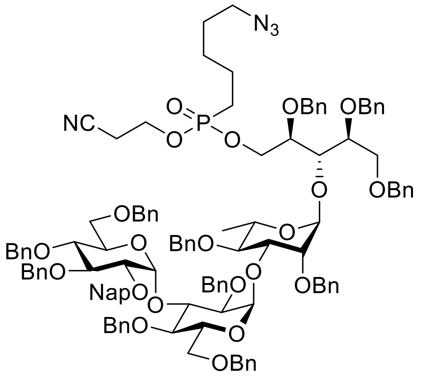


**3,4,6-Tri-*O*-benzyl-2-*O*-(2-napthylmethyl)-α-D-glucopyranosyl-(1→3)-2,4,6-tri-*O*-benzyl-α-D-glucopyranosyl-(1→3)-2,4-di-*O*-benzyl-α-L-rhamnopyranosyl-(1→3)-1,2,4-tri-*O*-benzyl-5-*O*-([5-azidopentyloxy]-[2-cyanoethoxy]-phosphono)-D-ribitol (31).** To a stirred mixture of 5-azidopentan-1-ol (52 μL, 0.396 mmol) and 5-ethylthio-1*H*-tetrazole (103 mg, 0.793 mmol) in anhydrous $CH_3CN$ (4 mL) was added freshly activated 3 Å MS (250 mg) at rt under an argon atmosphere. After being stirred for 10 min, tetrasaccharide phosphoramidite **S20** (155 mg, 0.079 mmol) in anhydrous $CH_3CN$ (2 mL) was added, and the reaction mixture was stirred for 1 h (monitored by TLC). Then, $H_2O$ (1 mL) and $I_2$ (302 mg, 1.189 mmol) in THF (1.5 mL) were added respectively. The mixture was stirred for further 2 h at rt before diluted with EtOAc, filtered through a pad of Celite, washed with satd. aq. $Na_2S_2O_3$ (7 mL) and satd. aq. $NaHCO_3$ (5 mL). The organic layer was dried over $MgSO_4$, filtered, and concentrated *in vacuo*. The residue was purified by silica gel column chromatography (EtOAc/*n*-Hexane, 1 : 2 to 1 : 1) to give **31** as a colorless viscous liquid (73 mg, 46%, mixture of diastereomers). Lower diastereoisomer: $^1$H NMR (600 MHz, $CDCl_3$,): $\delta_H$ 7.73 (d, 1H, $J$ = 7.2 Hz), 7.58 (d, 1H, $J$ = 7.6 Hz), 7.54 (d, 1H, $J$ = 8.4 Hz), 7.50 (s, 1H), 7.44-7.39 (m, 2H), 7.33-6.89 (m, 56H), 5.70 (d, 1H, $J$ = 3.5 Hz), 5.28 (t, 1H, $J$ = 2.9 Hz), 5.16 (s, 1H), 4.96 (d, 1H, $J$ = 10.8 Hz), 4.93 (dd, 1H, $J$ = 3.7, 11.9 Hz), 4.88 (d, 1H, $J$ = 10.4 Hz), 4.86 (d, 1H, $J$ = 10.4 Hz), 4.81 (d, 1H, $J$ = 12.1 Hz), 4.78 (dd, 1H, $J$ = 3.0, 11.1 Hz), 4.71-4.66 (m, 3H), 4.61-4.30 (m, 15H), 4.20-3.88 (m, 14H), 3.81-3.76 (m, 2H), 3.71-3.57 (m, 6H), 3.41-3.26 (m, 5H), 3.17 (q, 2H, $J$ = 6.8 Hz), 2.42-2.33 (m, 2H), 1.60-1.55 (m, 2H), 1.52-1.47 (m, 2H), 1.35-1.31 (m, 2H), 1.27 (d, 3H, $J$ = 5.8 Hz). $^{13}$C NMR (150 MHz, $CDCl_3$): $\delta_C$ 138.9 (2), 138.7, 138.6, 138.5, 138.4, 138.3, 138.2 (2), 138.1, 137.9 (3), 137.5, 135.5, 133.3, 133.0, 128.9, 128.7, 128.5 (2), 128.4 (2), 128.3 (2), 128.2, 128.1 (2), 128.0 (2), 127.9 (2), 127.8 (2), 127.7 (2), 127.6 (2), 127.5, 127.4, 127.2, 126.7, 126.0 (2), 125.8, 116.8, 116.7, 98.7 (2), 97.5, 93.2, 93.1, 82.5, 79.6, 79.3, 79.0, 78.3 (2), 78.2, 78.1, 78.0 (2), 77.9 (2), 79.3 (2), 75.7, 75.6, 75.5 (2), 75.2, 75.1, 72.0, 74.9, 74.0, 73.6, 73.4 (2), 73.3 (2), 73.2, 72.7 (2), 72.6, 72.5, 70.3, 70.2, 70.1, 70.0, 69.6 (2), 69.6, 69.5 (2), 68.3, 68.2 (2), 68.1 (2), 67.5 (3), 65.6, 65.5, 61.9, 61.8, 51.4, 51.3, 51.2 (2), 29.8, 29.7, 28.4 (2), 22.7 (2), 19.5 (3), 18.2. $^{31}$P NMR (202 MHz, $CDCl_3$): δ 0.215. HRMS (ESI) m/z: calcd for $C_{119}H_{129}N_4O_{22}PNa$ $[M+Na]^+$: 2019.8728, found: 2019.8726.

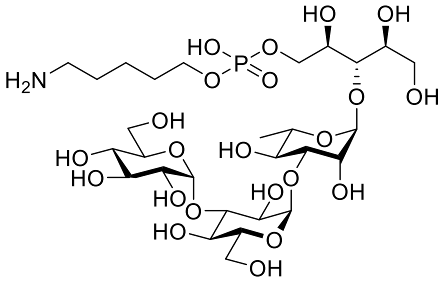


**α-D-Glucopyranosyl-(1→3)-α-D-glucopyranosyl-α-L-rhamnopyranosyl-(1→3)-5-*O*-(5-aminopentyl)-phosphono-D-ribitol (8).** *Prepared according to general deprotection procedure B* using phosphosugar **31** (57 mg, 0.028 mmol), mixture of solvents $CH_2Cl_2/H_2O$ (1 mL), and tetrabutylammonium hydroxide (37 µL, 0.057 mmol). Reaction time was 4 h. Second step with mixture of solvents $MeOH/H_2O/AcOH$ (4.25 mL) and $Pd(OH)_2/C$ (57 mg). Reaction time was 36 h. Compound **8** was obtained as an amorphous white solid (17.5 mg, 78% over two steps) after Sephadex LH-20 chromatography. $^1H$ NMR (600 MHz, $D_2O$): $\delta_H$ 5.39 (d, 1H, $J$ = 3.8 Hz), 5.13 (d, 1H, $J$ = 3.8 Hz), 5.05 (d, 1H, $J$ = 1.4 Hz), 4.25 (t, 1H, $J$ = 2.4 Hz), 4.11-4.09 (m, 1H), 4.06-3.97 (m, 5H), 3.96-3.85 (m, 5H), 3.84-3.76 (m, 7), 3.74-3.66 (m, 3H), 3.62-3.57 (m, 2H), 3.50 (t, 1H, $J$ = 9.7 Hz), 3.05 (t, 2H, $J$ = 7.4 Hz), 1.76-1.69 (m, 4H), 1.53-1.48 (m, 2H), 1.34 (d, 3H, $J$ = 6.2 Hz). $^{13}C$ NMR (150 MHz, $D_2O$): $\delta_C$ 103.1 (C1, $^1J_{CH}$ = 172 Hz), 101.7 (C1, $^1J_{CH}$ = 172 Hz), 98.0 (C1, $^1J_{CH}$ = 172 Hz), 82.2 (2), 77.7, 75.4, 74.5, 74.2, 73.9, 72.6, 72.5, 72.4, 72.0, 71.9, 71.8, 69.4, 69.0 (2), 68.3 (2), 65.1, 62.7, 62.5, 41.9, 31.6 (2), 28.8, 24.5, 19.2. $^{31}P$ NMR (202 MHz, $D_2O$): δ 2.29. HRMS (ESI) m/z: calcd for $C_{28}H_{55}NO_{22}P$ $[M+H]^+$: 788.2948, found: 788.2955.

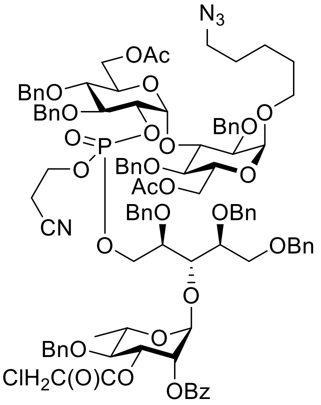


**5-Azidopentyl 2-*O*-benzoyl-4-*O*-benzyl-3-*O*-chloroacetyl-α-L-rhamnopyranosyl-(1→3)-1,2,4-tri-*O*-benzyl-D-ribityl-(1→[2-cyanoethyl]-phosphate→2)-6-*O*-acetyl-3,4-di-*O*-benzyl-α-D-glucopyranosyl-(1→3)-6-*O*-acetyl-2,4-di-*O*-benzyl-α-D-glucopyranoside (37).** To a stirred mixture of alcohol **36** (100 mg, 0.111 mmol) and 5-ethylthio-1*H*-tetrazole (145 mg, 1.113 mmol) in anhydrous $CH_3CN$ (5 mL) under an argon atmosphere was added freshly activated 3 Å MS (300 mg) at rt. After being stirred for 10 min, disaccharide phosphoramidite **34** (202 mg, 0.194 mmol) in anhydrous $CH_3CN$ (3 mL) was added, and the reaction mixture was stirred until completion (monitored by TLC, 1 h). Then, $H_2O$ (1 mL) and $I_2$ (423 mg, 1.670 mmol) in THF were added respectively. The mixture was stirred for further 2 h at rt before diluted with EtOAc, filtered through a pad of Celite, and washed with satd. aq. $Na_2S_2O_3$ (8 mL) and satd. aq. $NaHCO_3$ (5 mL). The organic

layer was dried over $MgSO_4$, filtered, and concentrated *in vacuo*. The residue was purified by silica gel column chromatography (EtOAc/*n*-Hexane, 1 : 2) to give **37** as a colourless viscous liquid (95 mg, 46%, mixture of phosphate diastereomers, 1:0.68) and recovered starting material **36** (30 mg, 30%). $^1$H NMR (600 MHz, $CDCl_3$): $\delta_H$ 8.01-7.99 (m, 4H), 7.60-7.58 (m, 2H), 7.47-7.43 (m, 4H), 7.38-7.09 (m, 80H), 5.62 (d, 1H, *J* = 3.4 Hz), 5.59 (d, 1H, *J* = 3.5 Hz), 5.47 (m, 1H), 5.45 (m, 1H), 5.33-5.30 (m, 1H), 5.07 (d, 1H, *J* = 1.1 Hz), 5.03-4.98 (m, 3H), 4.89-4.74 (m, 6H), 4.72-4.69 (m, 2H), 4.67-4.56 (m, 8H), 4.52-4.35 (m, 19H), 4.33-4.18 (m, 10H), 4.14-3.87 (m, 15H), 3.82-3.63 (m, 9H), 3.62-3.43 (m, 16H), 3.27-3.18 (m, 6H), 2.23-2.08 (m, 2H), 2.01 (s, 3H), 1.99 (s, 3H), 1.97 (s, 3H), 1.96 (s, 3H), 1.94 (t, 2H, *J* = 6.8 Hz), 1.58-1.53 (m, 8H), 1.40-1.36 (m, 4H), 1.24-1.22 (m, 6H). $^{13}$C NMR (150 MHz, $CDCl_3$): $\delta_C$ 170.9, 170.8, 166.7, 166.6, 165.6, 138.6, 138.4, 138.3, 138.2 (2), 138.1, 138.0 (2), 137.9 (2), 137.8, 137.7, 133.6, 130.1, 130.0, 129.6, 129.5, 128.7, 128.6 (2), 128.5 (3), 128.4 (2), 128.3, 128.2 (2), 128.1 (2), 128.0 (4), 127.9 (3), 127.8, 127.7, 116.6, 116.5, 98.1, 98.0, 96.8, 96.7 (2), 96.3, 80.2 (3), 79.3 (2), 78.8, 78.7, 78.5, 78.4, 78.3, 78.2, 77.9 (2), 77.8 (2), 77.7 (2), 77.6, 77.5, 76.8, 76.5, 76.2, 75.4 (2), 75.3 (2), 75.2 75.1, 74.5, 74.3, 74.2, 73.4, 73.0, 72.9, 72.6, 72.3, 72.2 (2), 70.6, 70.5, 69.2 (3), 69.0, 68.7, 68.4, 68.2, 68.1 (2), 67.8 (2), 67.7, 67.6, 63.0, 62.8, 62.2 (2), 62.1 (2), 51.4, 40.8 (2), 29.1 (2), 28.8, 23.6, 21.0, 18.9, 18.8, 18.7, 18.6, 18.1. $^{31}$P NMR (202 MHz, $CDCl_3$): δ 0.130, -0.065. HRMS (ESI) m/z: calcd for $C_{100}H_{113}ClN_4O_{26}P$ $[M+H]^+$: 1851.7064, found: 1851.7109.

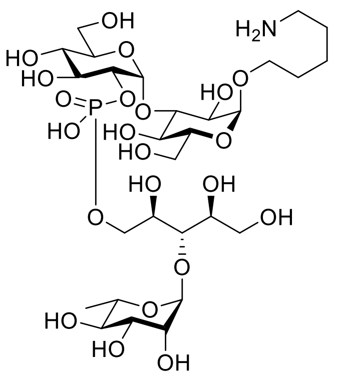

**5-Aminopentyl α-L-rhamnopyranosyl-(1→3)-D-ribityl-(1→hydrogen phosphate→2)-α-D-glucopyranosyl-(1→3)-α-D-glucopyranoside (7).** *Prepared according to general deprotection procedure C* using phosphosugar **37** (89 mg, 0.056 mmol), mixture of solvents $CH_2Cl_2/H_2O$ (2 mL), and tetrabutylammonium hydroxide (73 µL, 0.112 mmol). Reaction time was 4 h. Second step with $CH_2Cl_2$ (1 mL) and NaOMe (3 mL, 0.3 M in MeOH). Reaction time was 24 h. Third step with mixture of solvents $MeOH/H_2O/AcOH$ (4.25 mL) and $Pd(OH)_2/C$ (89 mg). Reaction time was 36 h. Compound **7** was obtained as an amorphous white solid (32 mg, 72% over 3 steps) after Sephadex LH-20 chromatography. $^1$H NMR (600 MHz, $D_2O$): $\delta_H$ 5.62 (d, 1H, *J* = 3.8 Hz), 5.00 (d, 1H, *J* = 1.3 Hz), 4.93 (d, 1H, *J* = 3.6 Hz), 4.16-4.11 (m, 2H), 4.09-4.01 (m, 4H), 4.00 (td, 1H, *J* = 3.7, 7.5 Hz), 3.92-3.73 (m, 11H), 3.71-3.65 (m, 4H), 3.58-3.55 (m, 1H), 3.52 (t, 1H, *J* = 9.6 Hz), 3.47 (t, 1H, *J* = 9.6 Hz), 3.03 (t, 2H, *J* = 7.5 Hz), 1.77-1.65 (m, 4H), 1.56-1.44 (m, 2H), 1.31 (d, 3H, *J* = 6.3 Hz). $^{13}$C NMR (150 MHz, $D_2O$): $\delta_C$ 101.0 (C1, $^1J_{CH}$ = 172 Hz), 98.2 (C1, $^1J_{CH}$ = 172 Hz), 97.2 (C1, $^1J_{CH}$ = 173 Hz), 79.7, 79.4, 75.3 (2), 71.9, 71.8, 71.6 (2), 71.5, 71.4, 70.2, 70.0, 69.9, 69.5, 69.4 (2), 69.3, 69.2, 67.6, 66.9 (2), 62.6, 60.5, 60.3, 39.4, 28.1, 26.5, 22.5, 16.5. $^{31}$P NMR (202 MHz, $D_2O$): δ 1.10.

HRMS (ESI) m/z: calcd for $C_{28}H_{55}NO_{22}P$ $[M+H]^+$: 788.2948, found: 788.2951.

**Synthesis of pseudo-tetrasaccharides 9, 10, 11, and 12**

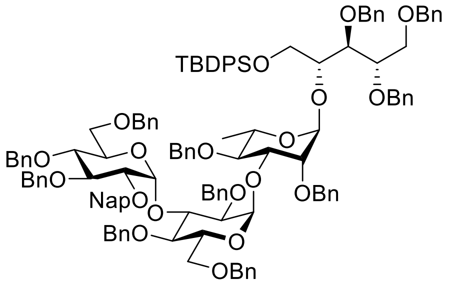


**3,4,6-Tri-*O*-benzyl-2-*O*-(2-napthylmethyl)-α-D-glucopyranosyl-(1→3)-2,4,6-tri-*O*-benzyl-α-D-glucopyranosyl-(1→3)-2,4-di-*O*-benzyl-α-L-rhamnopyranosyl-(1→4)-1,2,3-tri-*O*-benzyl-5-*O*-*tert*-butyldiphenylsilyl-D-ribitol (S21).** To a stirred mixture of donor **20** (750 mg, 0.502 mmol), acceptor **22** (331 mg, 0.502 mmol), and 4 Å MS (1 g) in anhydrous $CH_2Cl_2$ (15 mL) was added TMSOTf (27 µL, 0.150 mmol) at –30 °C under an argon atmosphere. The reaction mixture was stirred until completion (monitored by TLC, 1 h). The mixture was then neutralized with satd. aq. $NaHCO_3$ (250 µL), warmed to rt, filtered, and concentrated *in vacuo*. The residue was purified by silica gel column chromatography (EtOAc/*n*-Hexane, 1 : 7) to give **S21** as a colorless viscous syrup (674 mg, 67%) and recovered acceptor **22** (91 mg, 27%). $^1$H NMR (600 MHz, $CDCl_3$): $\delta_H$ 7.77 (d, 1H, $J$ = 7.7 Hz), 7.71 (td, 2H, $J$ = 1.5, 2.9 Hz), 7.68 (d, 2H, $J$ = 1.5 Hz), 7.62 (d, 2H, $J$ = 7.5 Hz), 7.57 (d, 2H, $J$ = 8.4 Hz), 7.56 (s,1H), 7.48-7.42 (m, 2H), 7.41-7.35 (m, 6H), 7.33-7.16 (m, 43H), 7.12-7.05 (m, 9H), 6.96-6.92 (m, 4H), 5.74 (d, 1H, $J$ = 3.5 Hz, H-1), 5.36 (d, 1H, $J$ = 1.4 Hz, H-1), 5.29 (d, 1H, $J$ = 3.3 Hz, H-1), 4.99 (d, 1H, $J$ = 11.0 Hz), 4.93-4.89 (m, 3H), 4.82 (d, 2H, $J$ = 11.0 Hz), 4.79-4.68 (m, 4H), 4.61-4.47 (m, 8H), 4.44-4.39 (m, 3H), 4.36-4.32 (m, 4H), 4.24 (dd, 1H, $J$ = 2.5, 9.6 Hz), 4.13-4.10 (m, 2H), 4.08 (d, 1H, $J$ = 10.1 Hz), 4.04 (t, 1H, $J$ = 2.1 Hz), 4.00-3.93 (m, 5H), 3.82 (dd, 1H, $J$ = 2.6, 6.6 Hz), 3.77-3.68 (m, 5H), 3.65-3.61 (m, 2H), 3.39-3.36 (m, 2H), 3.31-3.28 (m, 2H), 1.25 (d, 3H, $J$ = 6.1 Hz), 1.08 (s, 9H). $^{13}$C NMR (150 MHz, $CDCl_3$): $\delta_C$ 139.04, 138.8, 138.7, 138.6, 138.4, 138.3, 138.2, 138.1, 137.5, 135.8, 135.7, 135.6, 133.6, 133.4, 133.3, 133.1, 129.9, 128.9, 128.8, 128.5 (2), 128.4 (3), 128.3 (2), 128.2 (2), 128.1 (2), 127.9 (2), 127.8 (4), 128.7 (2), 127.6 (3), 127.5 (2), 127.3, 127.2, 126.7, 126.1, 126.0, 125.8, 98.1 (C1, $^1J_{CH}$ = 173 Hz), 97.5(C1, $^1J_{CH}$ = 172 Hz), 93.2 (C1, $^1J_{CH}$ = 172 Hz), 82.5, 79.7, 79.6, 79.4, 79.1, 78.7, 78.5, 78.3, 77.8, 77.4, 75.8 ($C$H$_2$), 75.7 (2), 75.4 (2), 75.0 ($C$H$_2$), 73.9 ($C$H$_2$), 73.6 ($C$H$_2$), 73.5 (4,$C$H$_2$), 73.4 ($C$H$_2$), 73.2 ($C$H$_2$), 72.9 ($C$H$_2$), 72.7 ($C$H$_2$), 70.5, 70.4 ($C$H$_2$), 70.1, 69.3, 68.4 ($C$H$_2$), 68.3 ($C$H$_2$), 64.5 ($C$H$_2$), 27.1, 19.4, 18.2. HRMS (ESI) m/z: calcd for $C_{127}H_{135}O_{19}Si$ $[M+H]^+$: 1991.9361; found: 1991.9406.

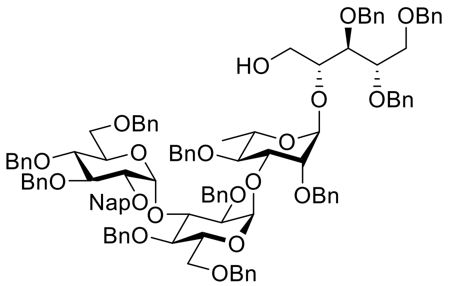

**3,4,6-Tri-*O*-benzyl-2-*O*-(2-napthylmethyl)-α-D-glucopyranosyl-(1→3)-2,4,6-tri-*O*-benzyl-α-D-glucopyranosyl-(1→3)-2,4-di-*O*-benzyl-α-L-rhamnopyranosyl-(1→4)-1,2,3-tri-*O*-benzyl-D-ribitol (24).** To a stirred solution of **S21** (777 mg, 0.389 mmol) in THF (7 mL) was added tetrabutyl ammonium fluoride (TBAF) in THF (1 M, 1.17 mL, 1.17 mmol) at room temperature. The reaction mixture was stirred until completion (monitored by TLC, 5 h). Then, it was diluted with EtOAc (15 mL), washed with satd. aq. $NH_4Cl$ (2 × 10 mL), dried over $MgSO_4$, filtered, and concentrated *in vacuo*. The residue was purified by silica gel column chromatography (EtOAc/*n*-Hexane, 1 : 3) to give **24** as a white foam (640 mg, 94%). $^1H$ NMR (600 MHz, $CDCl_3$): $\delta_H$ 7.45 (d, 1H, *J* = 7.5 Hz), 7.64 (d, 1H, *J* = 7.6 Hz), 7.60 (d, 1H, *J* = 8.3 Hz), 7.55 (s, 1H), 7.49-7.44 (m, 2H), 7.39 (d, 2H, *J* = 7.4 Hz), 7.35-7.12 (m, 50H), 7.07 (t, 2H, *J* = 7.6 Hz), 6.94 (d, 2H, *J* = 7.4 Hz), 5.74 (d, 1H, *J* = 3.3 Hz), 5.33 (d, 1H, *J* = 3.2 Hz), 5.08 (d, 1H, *J* = 1.8 Hz), 5.00-4.73 (m, 10H), 4.65-4.51 (m, 9H), 4.46-4.34 (m, 5H), 4.21 (dd, 1H, *J* = 2.5, 8.8 Hz), 4.16-4.09 (m, 3H), 4.06 (d, 1H, *J* = 9.7 Hz), 4.00 (s, 2H), 3.94-43.90 (m, 2H), 3.85-3.82 (m, 2H), 3.79-3.64 (m, 8H), 3.45 (dd, 1H, *J* = 1.9, 10.9 Hz), 3.39 (dd, 1H, *J* = 2.3, 10.9 Hz), 3.34-3.32 (m, 2H), 1.30 (d, 3H, *J* = 6.4 Hz). $^{13}C$ NMR (150 MHz, $CDCl_3$): $\delta_C$ 139.1, 138.9, 138.5, 138.4, 138.3 (2), 138.2, 138.1, 137.9, 137.6, 135.6, 133.3, 133.1, 128.9, 128.7, 128.6 (2), 128.5 (3), 128.4 (3), 128.3 (2), 128.2 (2), 128.1 (2), 128.0, 127.9 (3), 127.8 (3), 127.7 (3), 127.6 (2), 127.5, 127.2, 126.8, 126.7, 126.0, 125.8, 97.5, 96.6, 93.7, 82.5, 80.6, 79.6, 79.0, 78.5, 78.4, 78.3, 76.2, 75.6 (2), 75.4, 74.9, 74.4, 73.9, 73.6, 73.5, 73.4, 73.3, 73.1, 72.7, 70.4, 70.1, 69.7, 69.3, 68.3, 68.2, 60.8, 18.2. HRMS (ESI) m/z: calcd for $C_{111}H_{117}O_{19}$ $[M+H]^+$: 1753.8184, found: 1753.8207.

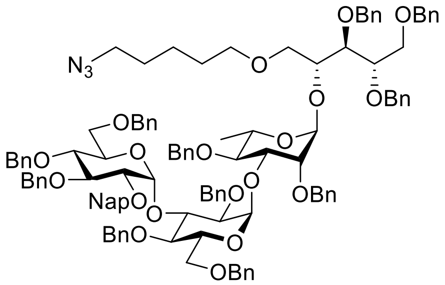


**3,4,6-Tri-*O*-benzyl-2-*O*-(2-napthylmethyl)-α-D-glucopyranosyl-(1→3)-2,4,6-tri-*O*-benzyl-α-D-glucopyranosyl-(1→3)-2,4-di-*O*-benzyl-α-L-rhamnopyranosyl-(1→4)-5-*O*-(5-azidopentyl)-1,2,3-tri-*O*-benzyl-D-ribitol (27).** To a stirred mixture of alcohol **24** (300 mg, 0.171 mmol), 5-azidopentyl methanesulfonate (70 mg, 0.342 mmol) in DMSO (2.5 mL) was added NaH (60%, 20 mg, 0.513 mmol) at 0 °C under an argon atmosphere. The ice bath was removed, and the reaction mixture was stirred ar rt until completion (monitored by TLC, 18 h). Then, it was quenched with satd. aq. $NH_4Cl$ (3 mL), poured into ice cold water, and extracted with $Et_2O$ (3 × 15 mL). The combined organic layer was dried over $MgSO_4$, filtered, and concentrated *in vacuo*. The obtained residue was purified by silica gel column chromatography (EtOAc/*n*-Hexane, 1 : 3) to afford pure compound **27** as a colorless viscous liquid (310 mg, 96%). $^1H$ NMR (600 MHz, $CDCl_3$): $\delta_H$ 7.70 (d, 1H, *J* = 7.6 Hz), 7.55 (d, 1H, *J* = 7.6 Hz), 7.50 (d, 1H, *J* = 8.5 Hz), 7.46 (s, 1H), 7.40-7.35 (m, 2H), 7.31-7.29 (m, 4H), 7.26-7.09 (m, 41H), 7.07-7.03 (m, 7H), 6.96 (t, 2H, *J* = 7.6 Hz), 6.84 (d, 2H, *J* = 7.3 Hz), 5.65 (d, 1H, *J* = 3.4 Hz), 5.25 (d, 1H, *J* = 3.2 Hz), 5.21 (d, 1H, *J* = 1.2 Hz), 4.92 (d, 1H, *J* = 10.9 Hz), 4.84 (t, 3H, *J* = 12.3 Hz), 4.74 (t, 2H, *J* = 11.4 Hz), 4.70-4.64 (m, 4H), 4.57-4.45 (m, 9H), 4.37-4.32 (m, 3H), 4.26

(dd, 2H, $J$ = 4.5, 11.6 Hz), 4.20 (q, 1H, $J$ = 4.7 Hz), 4.12 (dd, 1H, $J$ = 2.6, 9.5 Hz), 4.05-4.03 (m, 2H), 3.97-3.86 (m, 4H), 3.75-3.71 (m, 3H), 3.67-3.59 (m, 4H), 3.57 (dd, 1H, $J$ = 3.6, 9.8 Hz), 3.54 (d, 2H, $J$ = 5.0 Hz), 3.34 (dd, 1H, $J$ = 1.6, 10.8 Hz), 3.28-3.26 (m, 3H), 3.23 (d, 1H, $J$ = 10.6 Hz), 3.11 (t, 2H, $J$ = 6.9 Hz), 1.50-1.45 (m, 4H), 1.34-1.29 (m, 2H), 1.21 (d, 3H, $J$ = 6.1 Hz). $^{13}$C NMR (150 MHz, $CDCl_3$): $\delta_C$ 139.0, 138.9, 138.7, 138.6 (2), 138.5, 138.3, 138.2, 137.9, 137.6, 135.5, 133.3, 133.0, 128.9, 128.8, 128.5 (2), 128.4 (2), 128.3 (2), 128.2, 128.1 (2), 127.9 (2), 127.8 (3), 127.7, 127.6 (2), 127.5 (3), 127.2, 126.7 (2), 126.0 (2), 125.8, 97.5 (C1, $^1J_{CH}$ = 172 Hz), 97.4 (C1, $^1J_{CH}$ = 172 Hz), 93.0 (C1, $^1J_{CH}$ = 172 Hz), 82.5, 79.6 (2), 78.9, 78.5, 78.3 (2), 75.7 (2), 75.6 (2), 75.4, 75.2, 74.9, 73.9, 73.8, 73.5 (2), 73.4, 73.2, 73.1, 72.7, 72.6, 71.3, 71.2, 70.4, 70.3, 70.1, 69.0, 68.3, 51.4, 29.5, 28.8, 23.6, 18.2. HRMS (ESI) m/z: calcd for $C_{116}H_{125}N_3O_{19}Na$ [M+Na]$^+$: 1886.8799, found: 1886.8802.

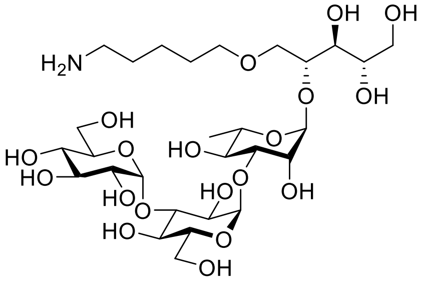


**α-D-Glucopyranosyl-(1→3)-α-D-glucopyranosyl-α-L-rhamnopyranosyl-(1→4)-5-*O*-(5-aminopentyl)-D-ribitol (9).** *Prepared according to general deprotection procedure A* using **27** (75 mg, 0.04 mmol), mixture of solvents MeOH/$H_2O$/$CH_2Cl_2$/AcOH (5.25 mL) and $Pd(OH)_2$/C (75 mg). Reaction time was 36 h. Compound **9** was obtained as an amorphous white solid (25 mg, 78%) after Sephadex LH-20 chromatography. $^1$H NMR (600 MHz, $D_2O$): $\delta_H$ 5.37 (d, 1H, $J$ = 3.6 Hz), 5.1 (s, 1H), 5.09 (s, 1H), 4.19 (t, 1H, $J$ = 2.4 Hz), 4.09-4.06 (m, 1H), 4.03-3.99 (m, 2H), 3.94 (t, 1H, $J$ = 9.4 Hz), 3.88 (dd, 1H, $J$ = 2.8, 9.6 Hz), 3.84-3.74 (m, 9H), 3.72-3.67(m, 4H), 3.65-3.54 (m, 5H), 3.48 (t, 1H, $J$ = 9.7 Hz), 3.02 (t, 2H, $J$ = 7.7 Hz), 1.72-1.62 (m, 4H), 1.46-1.41 (m, 2H), 1.32 (d, 3H, $J$ = 6.2 Hz). $^{13}$C NMR (150 MHz, $D_2O$): $\delta_C$ 99.4 (C1, $^1J_{CH}$ = 172 Hz), 99.2 (C1, $^1J_{CH}$ = 172 Hz), 95.6 (C1, $^1J_{CH}$ = 171 Hz), 79.5, 76.3, 75.4, 72.8, 72.1, 71.7, 71.6, 71.4, 71.3, 70.8, 70.1, 69.9, 69.7, 69.3, 69.2, 68.9, 67.1, 62.5, 60.1, 59.8, 39.3, 28.0, 26.4, 22.3, 16.6. HRMS (ESI) m/z: calcd for $C_{28}H_{54}NO_{19}$ [M+H]$^+$: 708.3212, found: 708.3311.

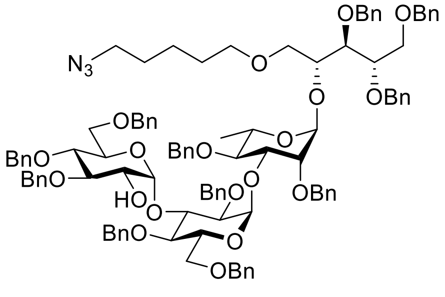


**3,4,6-Tri-*O*-benzyl-α-D-glucopyranosyl-(1→3)-2,4,6-tri-*O*-benzyl-α-D-glucopyranosyl-(1→3)-2,4-di-*O*-benzyl-α-L-rhamnopyranosyl-(1→4)-5-*O*-(5-azidopentyl)-1,2,3-tri-*O*-benzyl-D-ribitol (28).** To a solution of substrate **27** (379 mg, 0.203 mmol) in a mixture of $CH_2Cl_2$ and $H_2O$ (6.3 mL, 20:1) were added 2,6-ditertiary butyl pyridine (273 μL, 1.22 mmol) and DDQ (156 mg, 0.609 mmol)

at 0-5 °C. The reaction mixture was stirred for 2.5 h, and then diluted with $CH_2Cl_2$ (25 mL), washed with satd. aq. $NaHCO_3$, water, dried over $MgSO_4$, filtered, and concentrated *in vacuo.* The obtained residue was purified by silica gel column chromatography (EtOAc/*n*-Hexane, 1 : 5 to 1 : 4) to give **28** as a colorless viscous liquid (175 mg, 49%) and recovered starting material **27** (33 mg, 9%). $^1$H NMR (600 MHz, $CDCl_3$): $\delta_H$ 7.34-7.17 (m, 42H), 7.16-7.07 (m, 11H), 7.04 (t, 2H, *J* = 7.6 Hz), 5.54 (d, 1H, *J* = 3.2 Hz), 5.25 (d, 1H, *J* = 3.3 Hz), 5.24 (d, 1H, *J* = 1.6 Hz), 4.85 (d, 2H, *J* = 10.3 Hz), 4.82 (s, 2H), 4.75-4.72 (m, 4H), 4.59-4.49 (m, 8H), 4.43 (d, 1H, *J* = 10.7 Hz), 4.39-4.36 (m, 3H), 4.33 (d, 1H, *J* = 11.5 Hz), 4.25-4.23 (m, 1H), 4.18 (td, 1H, *J* = 2.0, 10.2 Hz), 4.14 (dd, 1H, *J* = 2.7, 9.4 Hz), 4.18 (d, 1H, *J* = 12.2 Hz), 4.04 (d, 1H, *J* = 11.8 Hz), 3.98-3.97 (m, 2H), 3.87-3.84 (m, 1H), 3.81-3.75 (m, 4H), 3.73-3.69 (m, 2H), 3.66-3.63 (m, 3H), 3.58-3.56 (m, 3H), 3.45 (dd, 1H, *J* = 1.9, 10.9 Hz), 3.33-3.28 (m, 4H), 3.25 (dd, 1H, *J* = 1.4, 10.7 Hz), 3.15 (t, 2H, *J* = 6.9 Hz), 2.47 (d, 1H, *J* = 7.3 Hz), 1.58-1.48 (m, 4H), 1.38-1.33 (m, 2H), 1.25 (d, 3H, *J* = 6.1 Hz). $^{13}$C NMR (150 MHz, $CDCl_3$): $\delta_C$ 139.0, 138.8, 138.7, 138.6 (2), 138.5, 138.2, 138.1, 137.7, 137.6, 128.8, 128.7, 128.5 (3), 128.4 (3), 128.3, 128.2, 128.1 (2), 128.0, 127.9, 127.8 (4), 127.7 (3), 127.6 (3), 98.9, 97.4, 93.3, 83.6, 79.6, 78.7, 78.6, 78.3, 77.6, 76.7, 75.6 (2), 75.5, 75.4, 74.7, 74.2, 73.8, 73.5, 73.4, 73.2, 73.0, 72.8, 72.6, 71.2, 71.1, 70.5, 70.4, 70.3, 69.1, 68.2 (2), 51.4, 29.5, 28.8, 23.6, 18.2. HRMS (ESI) m/z: calcd for $C_{105}H_{117}N_3O_{19}Na$ [M+Na]$^+$: 1746.8173, found: 1746.8173.

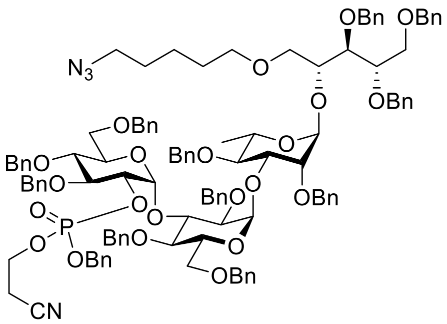


**3,4,6-Tri-*O*-benzyl-2-*O*-([benzyloxy]-[2-cyanoethoxy]-phosphono)-α-D-glucopyranosyl-(1→3)-2,4,6-tri-*O*-benzyl-α-D-glucopyranosyl-(1→3)-2,4-di-*O*-benzyl-α-L-rhamnopyranosyl-(1→4)-5-*O*-(5-azidopentyl)-1,2,3-tri-*O*-benzyl-D-ribitol (30).** To a stirred mixture of alcohol **28** (172 mg, 0.1 mmol) and 5-ethylthio-1*H*-tetrazole (155 mg, 1.2 mmol) in anhydrous $CH_3CN$ (6 mL) under an argon atmosphere was added freshly activated 3 Å MS (300 mg) at rt. After being stirred for 5-10 min, benzyl 2-cyanoethyl *N*,*N*-diisopropylphosphoramidite (123 mg, 0.4 mmol) in anhydrous $CH_3CN$ (2 mL) was added at rt, and the reaction mixture was stirred for 2 h (monitored by TLC). Then, it was cooled to –20 °C, followed by *m*-CPBA (345 mg, 2 mmol) addition. After being stirred for 1 h, the reaction mixture was warmed to room temperature, diluted with $CH_2Cl_2$, washed with satd. aq. $NaHCO_3$ (10 mL), satd. aq. $Na_2S_2O_3$ (10 mL), and water. The organic layer was dried over $MgSO_4$, filtered, and concentrated *in vacuo*. The residue was purified by silica gel column chromatography (EtOAc/*n*-Hexane, 1 : 4 to 1 : 2) to give **30** as a white foam (174 mg, 89%, mixture of diastereomers 1:5). Major isomer: $^1$H NMR (600 MHz, $CDCl_3$): $\delta_H$ 7.45 (t, 2H, *J* = 7.8 Hz), 7.38-7.19 (m, 46H), 7.16-7.07 (m, 12H), 5.77 (d, 1H, *J* = 3.2 Hz), 5.32 (d, 1H, *J* = 2.7 Hz), 5.28 (s, 1H), 5.00-4.87 (m, 4H), 4.83-4.72 (m, 6H), 4.63-4.33(m, 15H), 4.27 (s, 1H), 4.19-4.00 (m, 6H), 3.90-3.88 (m, 2H), 3.83-

3.74 (m, 5H), 3.72-3.66 (m, 4H), 3.61 (d, 2H, $J$ = 5.0 Hz), 3.48 (t, 2H, $J$ = 12.2 Hz), 3.41 (d, 1H, $J$ = 10.6 Hz), 3.36-3.31 (m, 3H), 3.19 (t, 2H, $J$ = 6.9 Hz), 2.06-1.95 (m, 2H), 1.57-1.52 (m, 4H), 1.42-1.35 (m, 2H), 1.27 (d, 3H, $J$ = 6.1 Hz). $^{13}$C NMR (150 MHz, $CDCl_3$): $\delta_C$ 138.6, 138.5 (2), 138.4, 138.2, 138.1, 137.8, 137.6, 135.5, 135.4, 128.7 (2), 128.6, 128.5, 128.4, 128.3 (2), 128.2 (2), 128.1, 128.0, 127.9, 127.8 (3), 127.7 (2), 127.6 (2), 127.5, 127.4 (2), 116.0, 97.3, 96.6, 92.9, 80.2 (2), 79.8, 79.5, 78.6, 78.5, 78.4, 78.3, 77.8 (2), 76.3, 75.6, 75.5, 75.3, 75.2, 75.1, 73.8, 73.6, 73.5 (2), 73.4, 73.0, 72.7, 72.6, 71.2, 71.1, 70.5, 70.2, 70.1 (2), 70.0, 69.0, 68.2, 61.9 (2), 51.4, 29.5, 28.8, 23.6, 18.8 (2), 18.2. $^{31}$P NMR (162 MHz, $CDCl_3$): δ -0.492. HRMS (ESI) m/z: calcd for $C_{115}H_{128}N_4O_{22}P$ $[M+H]^+$: 1947.8752, found: 1947.8788.

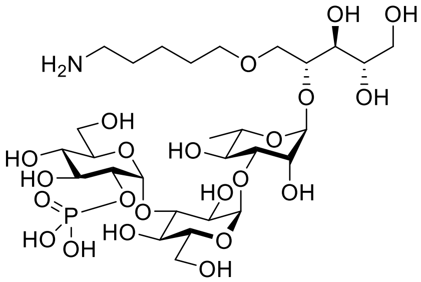


**2-*O*-Phosphono-α-D-glucopyranosyl-(1→3)-α-D-glucopyranosyl-(1→3)-α-L-rhamnopyranosyl-(1→4)-5-*O*-(5-aminopentyl)-D-ribitol (10).** *Prepared according to general deprotection procedure B* using phosphosugar **30** (170 mg, 0.087 mmol), mixture of solvents $CH_2Cl_2/H_2O$ (2 mL), and tetrabutylammonium hydroxide (113 μL, 0.175 mmol). Reaction time was 5 h. Second step with mixture of solvents $MeOH/H_2O/AcOH$ (4.25 mL) and $Pd(OH)_2/C$ (170 mg). Reaction time was 36 h. Compound **10** was obtained as an amorphous white solid (52 mg, 76% over 2 steps) after Sephadex LH-20 chromatography. $^1$H NMR (600 MHz, $D_2O$): $\delta_H$ 5.62 (d, 1H, $J$ = 3.8 Hz), 5.11-5.10 (m, 2H, two anomeric signals overlapped), 4.21 (t, 1H, $J$ = 2.4 Hz), 4.10-4.01 (m, 4H), 3.99 (t, 1H, $J$ = 8.9 Hz), 3.90 (dd, 1H, $J$ = 2.9, 9.6 Hz), 3.86-3.78 (m, 9H), 3.77-3.68 (m, 4H), 3.67-3.61 (m, 3H), 3.59-3.55 (m, 2H), 3.02 (t, 2H, $J$ = 7.4 Hz), 1.74-1.63 (m, 4H), 1.48-1.44 (m, 2H), 1.34 (d, 3H, $J$ = 6.2 Hz). $^{13}$C NMR (150 MHz, $D_2O$): $\delta_C$ 99.5 (C1, $^1J_{CH}$ = 172 Hz), 97.4 (C1, $^1J_{CH}$ = 172 Hz), 95.6 (C1, $^1J_{CH}$ = 172 Hz), 79.4, 76.5, 75.5, 75.1, 75.0, 72.1, 71.8 (2), 71.5, 71.4, 71.2, 70.9, 70.1, 69.6 (2), 69.3, 68.9 (2), 67.1, 62.6, 60.0 (2), 39.3, 28.0, 26.5, 22.3, 16.6. $^{31}$P NMR (202 MHz, $D_2O$): δ 1.06. HRMS (ESI) m/z: calcd for $C_{28}H_{55}NO_{22}P$ $[M+H]^+$: 788.2948, found: 788.2959.

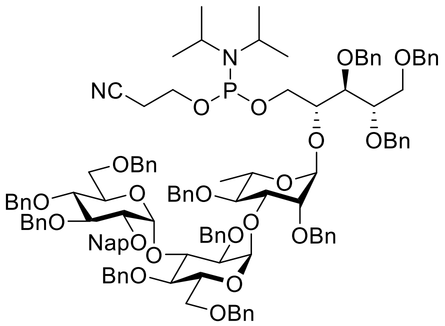


**3,4,6-Tri-*O*-benzyl-2-*O*-(2-napthylmethyl)-α–D-glucopyranosyl-(1→3)-2,4,6-tri-*O*-benzyl-α-D-glucopyranosyl-(1→3)-2,4-di-*O*-benzyl-α-L-rhamnopyranosyl-(1→4)-1,2,3-tri-*O*-benzyl-5-**

**([$N$,$N$-diisopropyl]-[2-cyanoethyl]-phosphoroamidite)-D-ribitol (S22).** To a soultion of alcohol **24** (325 mg, 0.185 mmol) in a mixture of anhydrous $CH_2Cl_2$ and $CH_3CN$ (3 mL, 2:1) were added 2-cyanoethyl *N,N,N',N'*-tetraisopropylphosphorodiamidite (235 µL, 0.741 mmol) and *N,N*-diisopropyl-ammonium tetrazolide (35 mg, 0.204 mmol) at room temperature under an argon atmosphere. The reaction mixture was stirred until completion (monitored by TLC, 1 h). Then, it was diluted with $CH_2Cl_2$ (15 mL), washed with satd. aq. $NaHCO_3$ (5 mL), dried over $MgSO_4$, filtered, and concentrated *in vacuo*. The obtained residue was purified by neutral alumina ($Al_2O_3$) column chromatography (EtOAc/*n*-Hexane, 1 : 3) under cold condition to afford pure compound **S22** as a colorless viscous liquid (325 mg, 90%, mixture of diastereomers). $^1$H NMR (600 MHz, $CDCl_3$): $\delta_H$ 7.72 (d, 1H, $J$ = 7.6 Hz), 7.57 (d, 1H, $J$ = 7.5 Hz), 7.52 (d, 1H, $J$ = 8.4 Hz), 7.50 (s, 1H), 7.43-7.37 (m, 2H), 7.32-7.30 (m, 4H), 7.28-7.20 (m, 22H), 7.19-7.10 (m, 19H), 7.06-7.04 (m, 7H), 6.97 (dt, 2H, $J$ = 2.5, 7.6 Hz), 6.87 (dd, 2H, $J$ = 3.0, 7.1 Hz), 5.69 (t, 1H, $J$ = 3.0 Hz), 5.27-5.23 (m, 2H), 4.94 (dd, 1H, $J$ = 3.5, 10.9 Hz), 4.88-4.84 (m, 3H), 4.81-4.79 (m, 3H), 4.72-4.66 (m, 3H), 4.60-4.55 (m, 4H), 4.53-4.44 (m, 5H), 4.39-4.33 (m, 3H), 4.29 (t, 2H, $J$ = 12.0 Hz), 4.23-4.21 (m, 1H), 4.15 (dd, 2H, $J$ = 1.4, 9.5 Hz), 4.08-4.03 (m, 2H), 4.01-3.97 (m, 2H), 3.94-3.88 (m, 2H), 3.87-3.72 (m, 6H), 3.69-3.62 (m, 6H), 3.59-3.52 (m, 3H), 3.34-3.29 (m, 2H), 3.26-3.23 (m, 2H), 2.38-2.27 (m, 2H), 1.20 (d, 3H, $J$ = 6.1 Hz). 1.15-1.08 (m, 12H). $^{13}$C NMR (150 MHz, $CDCl_3$): $\delta_C$ 139.0 (2), 138.8 (3), 138.6 (3), 138.5, 138.2, 138.0, 137.9, 137.5, 135.5, 133.3, 133.0, 129.1, 129.0, 128.9, 128.8, 128.7, 128.5, 128.4 (2), 128.3 (2), 128.2, 128.1, 128.0, 127.9 (3), 127.8 (2), 127.7, 127.6 (3), 127.5 (3), 127.4 (2), 127.3, 127.2, 126.7, 126.0 (2), 125.8, 117.8 (2), 97.4, 97.3 (d), 93.3 (d), 82.5, 79.6, 79.5, 79.3, 79.0, 78.9 (2), 78.8, 78.6, 78.4, 78.3, 76.5, 76.4 (2), 76.3, 75.8 (2), 75.6 (2), 75.5, 75.4, 75.3, 75.0, 74.9, 73.9, 73.8, 73.6, 73.5, 73.4 (2), 73.3, 73.2, 73.0, 72.9, 72.7, 70.4, 70.3, 70.0, 69.3, 68.2, 58.7, 58.6, 58.5, 43.4, 43.3 (2), 43.2, 24.9 (2), 24.8 (2), 20.3 (3), 18.2. $^{31}$P NMR (202 MHz, $CDCl_3$): δ 150, 149, HRMS (ESI) m/z: calcd for $C_{120}H_{134}N_2O_{20}P$ $[M+H]^+$: 1953.9262, found: 1953.9263.

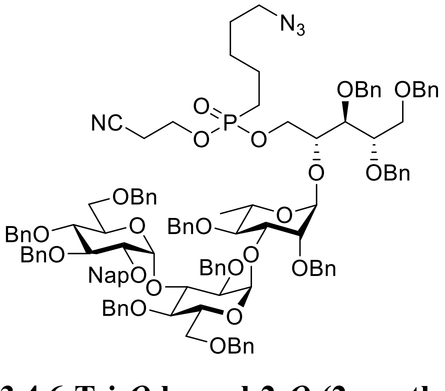


**3,4,6-Tri-*O*-benzyl-2-*O*-(2-napthylmethyl)-α-D-glucopyranosyl-(1→3)-2,4,6-tri-*O*-benzyl-α-D-glucopyranosyl-(1→3)-2,4-di-*O*-benzyl-α-L-rhamnopyranosyl-(1→4)-1,2,3-tri-*O*-benzyl-5-*O*-([5-azidopentyloxy]-[2-cyanoethoxy]-phosphono)-D-ribitol (32).** To a stirred mixture of 5-azidopentan-1-ol (36.5 µL, 0.284 mmol) and 5-ethylthio-1*H*-tetrazole (123 mg, 0.946 mmol) in anhydrous $CH_3CN$ (3 mL) was added freshly activated 3 Å MS (250 mg) at rt under an argon atmosphere. After being stirred for 10 min, tetrasaccharide phosphoramidite **S22** (185 mg, 0.094 mmol) in anhydrous $CH_3CN$ (2 mL) was added, and the reaction mixture was stirred for 1 h

(monitored by TLC). Then, $H_2O$ (1 mL) and $I_2$ (360 mg, 1.41 mmol) in THF (1.5 mL) were added respectively. The mixture was stirred for further 2 h at rt before diluted with EtOAc, filtered through a pad of Celite, washed with satd. aq. $Na_2S_2O_3$ (5 mL) and satd. aq. $NaHCO_3$ (5 mL). The organic layer was dried over $MgSO_4$, filtered, and concentrated *in vacuo*. The residue was purified by silica gel column chromatography (EtOAc/*n*-Hexane, 1 : 2 to 1 : 1) to give **32** as a colorless viscous liquid (94 mg, 50%, mixture of diastereomers). Upper diastereomer: $^1$H NMR (600 MHz, $CDCl_3$): $\delta_H$ 7.72 (d, 1H, *J* = 7.7 Hz), 7.57 (d, 1H, *J* = 7.5 Hz), 7.52 (d, 1H, *J* = 8.4 Hz), 7.50 (s, 1H), 7.42-7.37 (m, 2H), 7.32-7.11 (m, 45H), 7.07-7.05 (m, 7H), 6.99 (t, 2H, *J* = 7.6 Hz), 6.87 (d, 2H, *J* = 7.2 Hz), 5.67 (d, 1H, *J* = 3.5 Hz), 5.28 (d, 1H, *J* = 3.3 Hz), 5.13 (d, 1H, *J* = 1.6 Hz), 4.94 (d, 1H, *J* = 10.9 Hz), 4.89-4.85 (m, 3H), 4.78 (m, 3H), 4.70-4.66 (m, 3H), 4.62-4.55 (m, 4H), 4.52-4.45 (m, 5H), 4.39-4.33 (m, 4H), 4.28-4.25 (m, 3H), 4.22-4.18 (m, 1H), 4.12 (dd, 1H, *J* = 2.6, 9.5 Hz), 4.08-3.94 (m, 9H), 3.90 (t, 1H, *J* = 9.3 Hz), 3.81-3.79 (m, 2H), 3.78-3.76 (m, 1H), 3.72-3.57 (m, 6H), 3.36 (dd, 1H, *J* = 2.4, 10.9 Hz), 3.31 (dd, 1H, *J* = 2.4, 10.9 Hz), 3.26-3.23 (m, 2H), 3.17 (t, 2H, *J* = 6.8 Hz), 2.51-2.39 (m, 2H), 1.61-1.56 (m, 2H), 1.52-1.47 (m, 2H), 1.36-1.33 (m, 2H), 1.21 (d, 3H, *J* = 6.2 Hz). $^{13}$C NMR (150 MHz, $CDCl_3$): $\delta_C$ 139.0, 138.9, 138.7, 138.6, 138.5, 138.4, 138.2 (2), 138.1, 137.8, 137.5, 135.5, 133.3, 133.0, 129.0, 128.8, 128.6, 128.5 (3), 128.4 (3), 128.3 (2), 128.1 (2), 128.0, 127.9 (3), 127.8 (2), 127.7 (2), 127.6 (2), 127.5 (2), 127.2, 126.7 (2), 126.0 (2), 125.8, 116.7, 97.8, 97.5, 93.0, 82.5, 79.6, 79.4, 79.0, 78.9, 78.5, 78.4, 78.3, 75.8 (2), 75.7 (2), 75.6, 75.1, 74.9, 74.0, 73.8, 73.5, 73.4, 73.2, 72.9, 72.7, 70.3, 70.1, 69.6, 69.5, 68.3, 68.2, 68.1, 68.0, 62.1, 62.0, 51.3, 29.9, 29.8, 28.5, 22.8, 19.6, 19.5, 18.1. $^{31}$P NMR (202 MHz, $CDCl_3$): δ 0.095. HRMS (ESI) m/z: calcd for $C_{119}H_{130}N_4O_{22}P$ $[M+H]^+$: 1997.8909, found: 1997.8898. Lower diastereomer: $^1$H NMR (600 MHz, $CDCl_3$): $\delta_H$ 7.72 (d, 1H, *J* = 7.8 Hz), 7.57 (d, 1H, *J* = 7.5 Hz), 7.52 (d, 1H, *J* = 8.5 Hz), 7.50 (s, 1H), 7.42-7.38 (m, 2H), 7.32-7.21 (m, 27H), 7.32-7.21 (m, 27H), 7.20-7.10 (m, 18H), 7.08-7.05 (m, 7H), 6.99 (t, 2H, *J* = 7.6 Hz), 6.87 (d, 2H, *J* = 3.0, 7.3 Hz), 5.68 (d, 1H, *J* = 3.5 Hz), 5.29 (d, 1H, *J* = 3.5 Hz), 5.15 (d, 1H, *J* = 1.6 Hz), 4.94 (d, 1H, *J* = 10.8 Hz), 4.89-4.85 (m, 3H), 4.79 (m, 3H), 4.71-4.66 (m, 3H), 4.61-4.44 (m, 9H), 4.38-4.32 (m, 4H), 4.30-4.26 (m, 3H), 4.23-4.19 (m, 1H), 4.13 (dd, 1H, *J* = 2.6, 9.5 Hz), 4.08-3.95 (m, 9H), 3.91 (t, 1H, *J* = 9.5 Hz), 3.84-3.79 (m, 2H), 3.76-3.74 (m, 1H), 3.71-3.64 (m, 4H), 3.62-3.57 (m, 2H), 3.36 (dd, 1H, *J* = 2.4, 11.1 Hz), 3.30 (dd, 1H, *J* = 2.4, 11.1 Hz), 3.26-3.22 (m, 2H), 3.15 (t, 2H, *J* = 6.8 Hz), 2.44-2.42 (m, 2H), 1.60-1.55 (m, 2H), 1.51-1.46 (m, 2H), 1.35-1.29 (m, 2H), 1.21 (d, 3H, *J* = 6.1 Hz). $^{13}$C NMR (150 MHz, $CDCl_3$): $\delta_C$ 139.0, 138.9, 138.7, 138.6, 138.5, 138.4, 138.2, 138.1, 137.9, 137.6, 135.5, 133.3, 133.0, 128.9, 128.8, 128.6, 128.5 (3), 128.4 (3), 128.3 (2), 128.1 (2), 128.0, 127.9 (3), 127.8 (2), 127.7 (2), 127.6 (2), 127.5, 127.4, 127.2, 126.7, 126.0 (2), 125.8, 116.6, 97.8, 97.5, 93.0, 82.5, 79.6, 79.4, 79.1, 78.9, 78.5, 78.4 (2), 78.3, 75.8 (2), 75.6 (2), 75.2 (2), 74.9, 73.9, 73.8, 73.5, 73.4, 73.2, 72.9, 72.6, 70.3, 70.1, 69.6, 69.5, 68.4, 68.3 (3), 68.2, 61.9 (2), 51.2, 29.9, 29.8, 28.5, 22.7, 19.6, 19.5, 18.1. $^{31}$P NMR (202 MHz, $CDCl_3$): δ 0.095. HRMS (ESI) m/z: calcd for $C_{119}H_{130}N_4O_{22}P$ $[M+H]^+$: 1997.8909, found: 1997.8909.

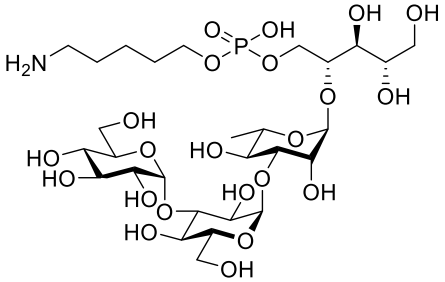

**α-D-Glucopyranosyl-(1→3)-α-D-glucopyranosyl-α-L-rhamnopyranosyl-(1→4)-5-*O*-(5-aminopentyl)-phosphono-D-ribitol (12).** *Prepared according to general deprotection procedure B* using phosphosugar **32** (94 mg, 0.047 mmol), mixture of solvents $CH_2Cl_2/H_2O$ (2 mL), and tetrabutylammonium hydroxide (61 μL, 0.094 mmol). Reaction time was 4 h. Second step with mixture of solvents $MeOH/H_2O/AcOH$ (4.25 mL) and $Pd(OH)_2/C$ (94 mg). Reaction time was 36 h. Compound **12** was obtained as an amorphous white solid (29 mg, 76% over two steps) after Sephadex LH-20 chromatography. $^1$H NMR (600 MHz, $D_2O$): $\delta_H$ 5.39 (d, 1H, $J$ = 3.8 Hz), 5.16 (s, 1H), 5.14 (d, 1H, $J$ =3.6 Hz), 4.28 (s, 1H), 4.19-4.16 (m, 1H), 4.14-4.12 (m, 1H), 4.07-4.03 (m, 3H), 3.96-3.90 (m, 4H), 3.88-3.77 (m, 9H), 3.74-3.65 (m, 3H), 3.63-3.58 (m, 2H), 3.51 (t, 1H, $J$ = 9.6 Hz), 3.06 (t, 2H, $J$ = 7.5 Hz), 1.76-1.69 (m, 4H), 1.54-1.48 (m, 2H), 1.35 (d, 3H, $J$ = 6.2 Hz). $^{13}$C NMR (150 MHz, $D_2O$): $\delta_C$ 99.8 (C1, $^1J_{CH}$ = 172 Hz), 99.2 (C1, $^1J_{CH}$ = 172 Hz), 95.6 (C1, $^1J_{CH}$ = 172 Hz), 79.6, 77.0 (2), 75.3, 72.8, 71.9, 71.6, 71.4, 71.3, 70.2, 70.0, 69.8, 69.3, 69.2, 66.9, 65.8 (2), 64.2 (2), 62.5, 60.1, 59.9, 39.3, 29.1 (2), 26.3, 22.0, 16.6. $^{31}$P NMR (202 MHz, $D_2O$): δ 2.18; HRMS (ESI) m/z: calcd for $C_{28}H_{55}NO_{22}P$ $[M+H]^+$: 788.2948, found: 788.2952.

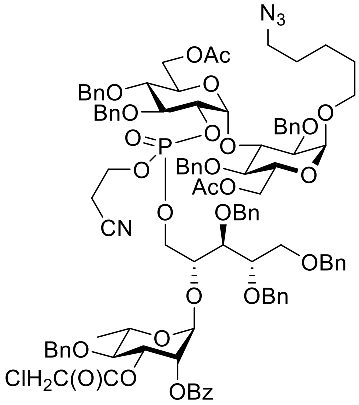

**5-Azidopentyl 2-*O*-benzoyl-4-*O*-benzyl-3-*O*-chloroacetyl-α-L-rhamnopyranosyl-(1→4)-1,2,3-tri-*O*-benzyl-D-ribityl-(1→[2-cyanoethyl]-phosphate→2)-6-*O*-acetyl-3,4-di-*O*-benzyl-α-D-glucopyranosyl-(1→3)-6-*O*-acetyl-2,4-di-*O*-benzyl-α-D-glucopyranoside (38).** To a stirred mixture of alcohol **36** (120 mg, 0.133 mmol) and 5-ethylthio-1*H*-tetrazole (174 mg, 1.336 mmol) in anhydrous $CH_3CN$ (6 mL) under an argon atmosphere was added freshly activated 3 Å MS (300 mg) at rt. After being stirred for 10 min, disaccharide phosphoramidite **35** (276 mg, 0.276 mmol) in anhydrous $CH_3CN$ (3 mL) was added, and the reaction mixture was stirred until completion (monitored by TLC, 1 h). Then, $H_2O$ (1 mL) and $I_2$ (508 mg, 2 mmol) in THF were added respectively. The mixture was stirred for further 2 h at rt before diluted with EtOAc, filtered through a pad of Celite, and washed with satd. aq. $Na_2S_2O_3$ (10 mL) and satd. aq. $NaHCO_3$ (5 mL). The organic layer was

dried over $MgSO_4$, filtered, and concentrated *in vacuo*. The residue was purified by silica gel column chromatography (EtOAc/*n*-Hexane, 1 : 2) to give **38** as a colourless viscous liquid (128 mg, 52%, mixture of phosphate diastereomers). Upper diastereomer: $^1$H NMR (600 MHz, $CDCl_3$): $\delta_H$ 8.03 (d, 2H, *J* = 7.0 Hz), 7.62 (t, 1H, *J* = 7.2 Hz), 7.47 (t, 2H, *J* = 7.7 Hz), 7.30-7.12 (m, 40H), 5.55 (d, 1H, *J* = 3.7 Hz), 5.54 (dd, 1H, *J* = 2.0, 3.1 Hz), 5.28 (dd, 1H, *J* = 3.1, 9.8 Hz), 4.96-4.95 (m, 2H), 4.81 (q, 2H, *J* = 11.1 Hz), 4.74 (d, 1H, *J* = 10.8 Hz), 4.70 (d, 1H, *J* = 3.6 Hz), 4.64 (d, 1H, *J* = 11.4 Hz), 4.60-4.49 (m, 5H), 4.47-4.38 (m, 6H), 4.32 (dt, 1H, *J* = 2.7, 11.4 Hz), 4.28 (dt, 1H, *J* = 3.6, 9.6 Hz), 4.20-4.16 (m, 3H), 4.06-4.03 (m, 2H), 4.01-3.96 (m, 2H), 3.94-3.88 (m, 4H), 3.82 (d, 1H, *J* = 14.6 Hz), 3.75-3.71 (m, 4H), 3.66-3.60 (m, 2H), 3.58-3.47 (m, 4H), 3.42 (dd, 1H, *J* = 3.6, 9.6 Hz), 3.26-3.19 (m, 3H), 2.11-2.00 (m, 2H), 1.98 (s, 3H), 1.97 (s, 3H), 1.56-1.52 (m, 4H), 1.39-1.34 (m, 2H), 1.13 (d, 3H, *J* = 6.1 Hz). $^{13}$C NMR (150 MHz, $CDCl_3$): $\delta_C$ 170.9, 170.8, 166.7, 165.6, 138.6, 138.5, 138.4, 138.2, 138.1, 137.9, 133.7, 130.1, 129.6, 128.8, 128.7, 128.6 (3), 128.5, 128.1, 128.0 (2), 127.9, 127.8, 127.7 (2), 116.7, 96.7 ($^1J_{CH}$ = 172 Hz), 96.4, 96.3, 80.1 (2), 78.8, 78.6, 78.3 (2), 78.2, 78.1, 77.7 (2), 76.6, 75.4, 75.3, 75.2, 74.4, 74.1, 73.7, 73.5, 72.9, 72.7, 70.4, 69.9, 69.1, 68.9, 68.3, 68.1, 67.1, 67.0, 63.0, 62.8, 62.4, 62.3, 51.4, 40.8, 29.1, 28.8, 21.1, 21.0, 18.9, 18.8, 18.0. $^{31}$P NMR (202 MHz, $CDCl_3$): δ 0.118. HRMS (ESI) m/z: calcd for $C_{100}H_{112}ClN_4O_{26}PNa$ $[M+Na]^+$: 1873.6883, found: 1873.6931. Lower diastereomer: $^1$H NMR (600 MHz, $CDCl_3$): $\delta_H$ 8.00 (d, 2H, *J* = 7.3 Hz), 7.60 (t, 1H, *J* = 7.6 Hz), 7.45 (t, 2H, *J* = 7.5 Hz), 7.30-7.12 (m, 40H), 5.58 (d, 1H, *J* = 3.3 Hz), 5.49 (dd, 1H, *J* = 1.6, 2.6 Hz), 5.36 (dd, 1H, *J* = 3.1, 9.8 Hz), 4.95 (d, 1H, *J* = 11.5 Hz), 4.88 (d, 1H, *J* = 1.2 Hz), 4.84 (d, 1H, *J* = 11.1 Hz), 4.72-4.67 (m, 3H), 4.64-4.62 (m, 3H), 4.59-4.49 (m, 3H), 4.47-4.39 (m, 5H), 4.36-4.30 (m, 2H), 4.27-4.24 (m, 2H), 4.18-4.15 (m, 2H), 4.10-4.07 (m, 2H), 4.00-3.93 (m, 3H), 3.90-3.82 (m, 2H), 3.780-3.74 (m, 2H), 3.70-3.67 (m, 2H), 3.64-3.50 (m, 6H), 3.48 (t, 2H, *J* = 9.4 Hz), 3.41 (dd, 1H, *J* = 3.8, 9.4 Hz), 3.25-3.16 (m, 3H), 2.20-2.14 (m, 2H), 1.99 (s, 3H), 1.97 (s, 3H), 1.56-1.52 (m, 4H), 1.39-1.34 (m, 2H), 1.11 (d, 3H, *J* = 6.2 Hz). $^{13}$C NMR (150 MHz, $CDCl_3$): $\delta_C$ 170.9, 170.8, 166.6, 165.7, 138.4 (2), 138.2, 138.1, 138.0, 137.9 (2), 137.8, 133.7, 130.1, 129.5, 128.8, 128.7, 128.6 (3), 128.5 (2), 128.3, 128.2, 128.1 (2), 128.0, 127.9, 127.8 (4), 116.7, 96.2 ($^1J_{CH}$ = 172 Hz), 96.7, 96.3, 80.0 (2), 79.4, 78.5, 78.4, 78.1, 77.9 (2), 75.4, 75.3, 75.2, 74.2, 73.5, 73.2, 73.1, 72.6, 70.6, 69.3 (2), 68.6 (2), 68.2, 68.0, 67.1, 63.1, 63.0, 62.3, 62.2, 51.4, 40.8, 29.1, 28.8, 23.6, 21.0, 18.8 (2), 18.0. $^{31}$P NMR (202 MHz, $CDCl_3$): δ 0.189. HRMS (ESI) m/z: calcd for $C_{100}H_{113}ClN_4O_{26}P$ $[M+H]^+$: 1851.7064, found: 1851.7080.

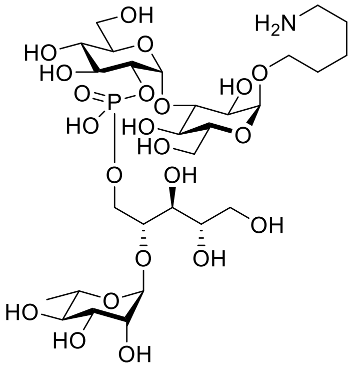


**5-Aminopentyl α-L-rhamnopyranosyl-(1→4)-D-ribityl-(1→hydrogen phosphate→2)-α-D-**

**glucopyranosyl-(1→3)-α-D-glucopyranoside (11).** *Prepared according to general deprotection procedure C* using phosphosugar **38** (115 mg, 0.062 mmol), mixture of solvents $CH_2Cl_2/H_2O$ (2 mL), and tetrabutylammonium hydroxide (57 µL, 0.124 mmol). Reaction time was 4 h. Second step with $CH_2Cl_2$ (1 mL) and NaOMe (3 mL, 0.3 M in MeOH). Reaction time was 24 h. Third step with mixture of solvents MeOH/$H_2O$/AcOH (4.25 mL) and $Pd(OH)_2/C$ (115 mg). Reaction time was 36 h. Compound **11** was obtained as an amorphous white solid (38 mg, 78% over 3 steps) after Sephadex LH-20 chromatography. $^1$H NMR (600 MHz, $D_2O$): $\delta_H$ 5.60 (d, 1H, $J$ = 3.8 Hz), 5.08 (d, 1H, $J$ = 1.1 Hz), 4.90 (d, 1H, $J$ = 3.7 Hz), 4.25-4.21 (m, 1H), 4.12-4.05 (m, 4H), 4.00-3.96 (m, 1H), 3.89-3.71 (m, 12H), 3.69-3.62 (m, 4H), 3.56-3.44 (m, 3H), 3.02 (t, 2H, $J$ = 7.6 Hz), 1.75-1.63 (m, 4H), 1.54-1.39 (m, 2H), 1.29 (d, 3H, $J$ = 6.3 Hz). $^{13}$C NMR (150 MHz, $D_2O$): $\delta_C$ 100.2 (C1, $^1J_{CH}$ = 172 Hz), 98.1 (C1, $^1J_{CH}$ = 172 Hz), 97.1 (C1, $^1J_{CH}$ = 171 Hz), 79.4, 77.0, 76.9, 75.4 (2), 71.9 (2), 71.5 (2), 71.4 (2), 71.2, 70.2, 70.0, 69.9, 69.4, 69.2 (2), 67.6, 64.4 (2), 62.5, 60.4, 60.3, 39.3, 28.0, 26.5, 22.5, 16.5. $^{31}$P NMR (202 MHz, $D_2O$): δ 0.91. HRMS (ESI) m/z: calcd for $C_{28}H_{55}NO_{22}P$ $[M+H]^+$: 788.2948, found: 788.2963.

**Synthesis of pseudo-tetrasaccharide 13**

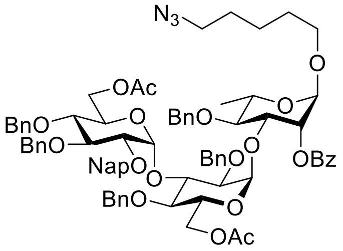


**5-Azidopentyl 6-*O*-acetyl-3,4-di-*O*-benzyl-2-*O*-(2-napthylmethyl)-α-D-glucopyranosyl-(1→3)-6-*O*-acetyl-2,4-di-*O*-benzyl-α-D-glucopyranosyl-(1→3)-2-*O*-benzoyl-4-*O*-benzyl-α-L-rhamnopyranoside (S23).** A mixture of donor **16** (430 mg, 0.399 mmol), acceptor **39** (150 mg, 0.319 mmol), and freshly activated 4 Å MS (500 mg) in anhydrous $CH_2Cl_2$ (12 mL) were stirred under an argon atmosphere at rt for 30 min. Then, it was cooled to −30 °C, followed by addition of NIS (100 mg, 0.479 mmol, 1.2 equiv. to donor) and TfOH (0.5 M in $Et_2O$, 200 µL, 0.099 mmol, 0.25 equiv. to donor). The reaction mixture was stirred until completion (monitored by TLC, 2 h). Then, it was quenched by addition of satd. aq. $NaHCO_3$ (1 mL) and warmed to room temperature. The mixture was filtered through a pad of Celite, and the filtrate was washed with satd. aq. $Na_2S_2O_3$, satd. aq. $NaHCO_3$, and brine. The separated organic layer was dried over $MgSO_4$, filtered, and concentrated *in vacuo*. The resultant residue was purified by silica gel column chromatography (EtOAc/*n*-Hexane, 1 : 5) to afford pure α-isomer **S23** as a colorless viscous liquid (312 mg, 70%). $^1$H NMR (600 MHz, $CDCl_3$): $\delta_H$ 7.99 (dd, 2H, $J$ = 1.0, 8.5 Hz), 7.71 (d, 1H, $J$ = 7.6 Hz), 7.55-7.50 (m, 3H), 7.46 (s, 1H), 7.42-7.37 (m, 4H), 7.34 (d, 2H, $J$ = 7.2 Hz), 7.27-7.20 (m, 9H), 7.17-7.13 (m, 6H), 7.08-6.94 (m, 9H), 5.53 (d, 1H, $J$ = 3.5 Hz, anomeric), 5.50 (t, 1H, $J$ = 2.4 Hz), 5.29 (d, 1H, $J$ = 3.6 Hz, anomeric), 4.95-4.91 (m, 3H), 4.84-4.74 (m, 3H, anomeric-H overlapped), 4.76 (1H, d, $J$ = 11.1 Hz), 4.69 (1H, d, $J$ = 8.6 Hz), 4.67 (1H, d, $J$ = 7.2 Hz), 4.44 (1H, d, $J$ = 11.1 Hz), 4.40-4.33 (m, 3H), 4.31 (1H, d, $J$ = 11.6

Hz), 4.27-4.25 (m, 2H), 4.15 (dd, 1H, $J$ = 2.0, 12.2 Hz), 4.09 (td, 1H, $J$ = 2.7, 10.3 Hz), 4.00-3.96 (m, 2H), 3.92 (dd, 1H, $J$ = 1.6, 12.2 Hz), 3.80-3.77 (m, 2H), 3.70-3.62 (m, 3H), 3.57 (dd, 1H, $J$ = 3.5, 9.7 Hz), 3.53 (dd, 1H, $J$ = 3.5, 9.7 Hz), 3.45-3.38 (m, 2H), 3.27 (t, 2H, $J$ = 6.8 Hz), 1.99 (s, 3H), 1.91 (s, 3H), 1.62-1.56 (m, 4H), 1.43-1.39 (m, 2H), 1.37 (d, 3H, $J$ = 6.2 Hz). $^{13}$C NMR (150 MHz, $CDCl_3$): $\delta_C$ 170.8, 170.7, 166.4, 138.7, 138.4, 137.9, 137.7, 136.9, 135.2, 133.6, 133.2, 133.0 130.0, 129.7, 128.7, 128.6 (2), 128.5 (2), 128.4 (2), 128.3, 128.2, 128.1, 128.0, 127.9 (2), 127.8 (2), 127.6, 127.0, 126.9, 126.1, 126.0, 97.5 (C1, $^1J_{CH}$ = 173 Hz), 97.4 (C1, $^1J_{CH}$ = 172 Hz), 91.4 (C1, $^1J_{CH}$ = 172 Hz) , 82.4, 79.6, 79.4, 78.9, 77.7, 76.8, 76.2, 75.8, 75.7, 74.9, 74.3, 73.9, 72.3, 71.8, 68.7, 68.5, 68.3, 67.9, 63.0, 62.6, 51.4, 29.1, 28.8, 23.5, 21.1, 21.0, 18.2. HRMS (ESI) m/z: calcd for $C_{80}H_{87}N_3O_{18}Na$ [M+Na]$^+$: 1400.5877, found: 1400.5895.

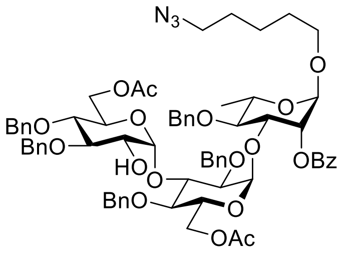


**5-Azidopentyl 6-*O*-acetyl-3,4-di-*O*-benzyl-α–D-glucopyranosyl-(1→3)-6-*O*-acetyl-2,4-di-*O*-benzyl-α-D-glucopyranosyl-(1→3)-2-*O*-benzoyl-4-*O*-benzyl-α-L-rhamnopyranoside (40).** A solution of trisaccharide **S23** (800 mg, 0.771 mmol) in a mixture of $CH_2Cl_2$ and phosphate buffer pH 7 (3.3 ml, 10:1) was cooled to 0-5 °C. DDQ (350 mg, 1.542 mmol) was added in protions over 10 min, and the reaction mixture was stirred at room temperature until completion (monitored by TLC, 4 h). Then, it was diluted with $CH_2Cl_2$, washed with sat. aq. $NaHCO_3$, water, dried over $MgSO_4$, and concentrated *in vacuo*. The resultant residue was purified by silica gel column chromatography (EtOAc/*n*-Hexane, 1 : 4) to afford pure compound **40** as a white foam (124 mg, 57%). $^1$H NMR (600 MHz, $CDCl_3$): $\delta_H$ 7.99 (d, 2H, $J$ = 7.1 Hz), 7.71 (t, 1H, $J$ = 7.4 Hz), 7.42 (t, 2H, $J$ = 7.8 Hz), 7.29-7.23 (m, 13H), 7.19-7.11 (m, 5H), 7.06 (t, 3H, $J$ = 7.6 Hz), 7.00-6.95 (m, 4H), 5.49 (t, 1H, $J$ = 2.2 Hz), 5.41 (d, 1H, $J$ = 3.0 Hz), 5.28 (d, 1H, $J$ = 3.4 Hz), 4.89 (d, 2H, $J$ = 10.5 Hz), 4.82 (1H, d), 4.79 (q, 2H, $J$ = 11.3 Hz), 4.72 (d, 1H, $J$ = 11.0 Hz), 4.66 (d, 1H, $J$ = 10.5 Hz), 4.42 (1H, d, $J$ = 11.0 Hz), 4.37-4.35 (m, 2H), 4.26-4.16 (m, 5H), 4.09-4.04 (m, 2H), 3.89 (dd, 1H, $J$ = 1.8, 12.2 Hz), 3.80-3.77 (m, 1H), 3.73 (dd, 1H, $J$ = 3.0, 12.2 Hz), 3.66-3.58 (m, 5H), 3.47 (dd, 1H, $J$ = 3.4, 9.7 Hz), 3.42-3.37 (m, 2H), 3.27 (t, 2H, $J$ = 6.9 Hz), 2.35 (d, 1H, $J$ = 6.7 Hz), 2.04 (s, 3H), 1.87 (s, 3H), 1.62-1.57 (m, 4H), 1.43-1.39 (m, 2H), 1.37 (d, 3H, $J$ = 6.2 Hz). $^{13}$C NMR (150 MHz, $CDCl_3$): $\delta_C$ 170.8, 170.7, 166.4, 138.7, 138.3, 137.6, 136.9, 133.7, 130.0, 129.8, 128.7 (2), 128.6 (2), 128.5 (2), 128.4, 128.1, 128.0, 127.9 (3), 127.8, 98.5 (C1, $^1J_{CH}$ = 172 Hz), 97.5 (C1, $^1J_{CH}$ = 172 Hz), 91.5 (C1, $^1J_{CH}$ = 172 Hz), 83.4, 79.6, 79.0, 78.8, 76.2, 75.6, 74.7, 74.6, 72.9, 72.3, 71.9, 68.8, 68.7, 68.6, 68.3, 67.9, 62.9, 62.6, 51.4, 29.1, 28.8, 23.5, 21.0, 21.0, 18.2. HRMS (ESI) m/z: calcd for $C_{64}H_{77}N_3O_{18}Na$ [M+Na]$^+$: 1198.5094, found: 1198.4177.

**Scheme S1-S4.** Synthesis of ribitol phosphoramidite.[a]

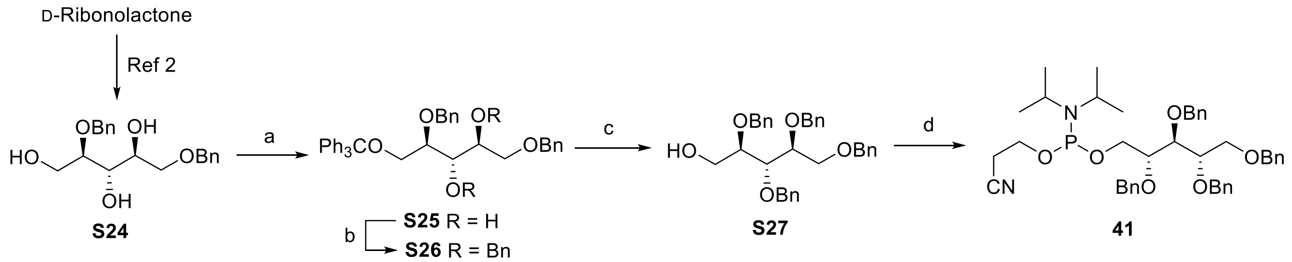


[a]Reagents and conditions: (a) $CPh_3Cl$, pyridine, DMAP, 50 °C, overnight, 81%; (b) NaH, BnBr, DMF, 0 °C to rt, 2 h, 90%; (c) PTSA, MeOH, $CH_2Cl_2$, rt, 1.5 h, 86%; (d) *N*,*N*-diisopropylammonium tetrazolide, 2-cyanoethyl-*N*,*N*,*N'*,*N'*-tetraisoproylphosphordiamidite, $CH_2Cl_2/CH_3CN$ (2/1), rt, 1 h, 88%.

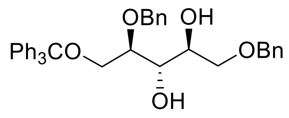

**1,4-Di-*O*-benzyl-5-*O*-(triphenylmethyl)-D-ribitol (S25).** To a stirred solution of triol **S24** (2.4 g, 7.22 mmol) in pyridine (30 mL) was added DMAP (88 mg, 0.722 mmol), followed by addition of triphenylmethyl chloride (2.42 g, 8.67 mmol) at room temparture under an argon atmosphere. The reaction mixture was stirred at 55 °C overnight. Then, it was cooled to room temperature and concentrated *in vacuo.* The resulting residue was dissolved in $CH_2Cl_2$ and washed with 1% HCl, satd. aq. $NaHCO_3$, and brine. The combined organic layer was dried over $MgSO_4$, filtered, concentrated *in vacuo*. The obtained residue was purified by silica gel column chromatography (EtOAc/*n*-Hexane, 1 : 3) to give compound **S25** as a viscous liquid (2.2 g, 81%). $^1H$ NMR (600 MHz, $CDCl_3$): $\delta_H$ 7.38-7.28 (m, 25H), 4.73 (d, 1H, $J$ = 11.4 Hz), 4.55-4.49 (m, 3H), 3.97 (t, 1H, $J$ = 6.3 Hz), 3.85-3.82 (m, 1H), 3.75-3.73 (m, 1H), 3.67-3.58 (m, 3H), 3.39 (dd, 1H, $J$ = 4.6, 10.4 Hz), 2.93 (brs, 2H). $^{13}C$ NMR (150 MHz, $CDCl_3$): $\delta_C$ 143.9, 138.2, 137.9, 128.8, 128.6 (2), 128.0, 127.9, 127.3, 87.4, 79.3, 73.7, 72.7, 72.3, 71.9, 70.9, 63.3. HRMS (ESI) m/z: calcd for $C_{38}H_{38}O_5Na$ [M+Na]$^+$: 597.2611, found: 597.2619.

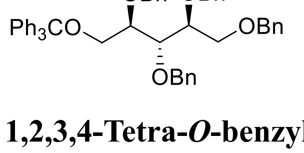

**1,2,3,4-Tetra-*O*-benzyl-5-*O*-(triphenylmethyl)-D-ribitol (S26).** To a suspension of NaH (60%, 95 mg, 2.348 mmol) in dry DMF (6 mL) was added a solution of diol **S25** (0.45 g, 0.782 mmol) in dry DMF (4 mL) at 0 °C under argon atmosphere. The mixture solution was stirred for 10 min, followed by addition of benzyl bromide (240 μL, 2.03 mmol). The reaction mixture was allowed to stir at room temperature until the starting material disappeared (monitored by TLC, 3 h). Then, it was carefully quenched with MeOH at 0 °C and concentrated *in vacuo*. The resulting residue was poured into ice-cold water and extracted with EtOAc (3 × 15 mL). The combined organic layer was dried over $MgSO_4$, filtered, and concentrated *in vacuo*. The obtained residue was purified by silica gel column chromatography (EtOAc/*n*-Hexane, 1 : 10) to give compound **S26** (0.54 g, 90%) as a viscous liquid. $^1H$ NMR (600 MHz, $CDCl_3$): $\delta_H$ 7.53-7.13 (m, 35H), 4.85 (d, 1H, $J$ = 11.4 Hz), 4.75 (d, 1H, $J$ = 11.6

Hz), 4.68 (d, 2H, $J$ = 11.6 Hz), 4.62 (dd, 2H, $J$ = 9.5, 11.6 Hz), 4.57 (q, 2H, $J$ = 12.0 Hz), 4.01-3.95 (m, 3H), 3.80 (dd, 1H, $J$ = 3.1, 10.7 Hz), 3.76 (dd, 1H, $J$ = 5.6, 10.4 Hz), 3.52 (dd, 1H, $J$ = 2.4, 10.3 Hz), 3.46 (dd, 1H, $J$ = 5.8, 10.3 Hz). $^{13}$C NMR (150 MHz, $CDCl_3$): $\delta_C$ 144.3, 138.9 (2), 138.7, 138.6, 129.0, 128.5, 128.4 (2), 128.3, 128.1, 127.9 (2), 127.8, 127.6, 127.5 (2), 127.1, 86.8, 79.2, 79.1, 78.9, 73.8, 73.4, 72.8, 72.6, 70.6, 64.2. HRMS (ESI) m/z: calcd for $C_{52}H_{50}O_5Na$ $[M+Na]^+$: 777.3584, found: 777.3560.

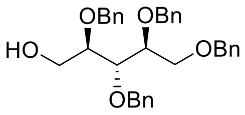


**1,2,3,4-Tetra-*O*-benzyl-D-ribitol (S27).** To a stirred solution of compound **S26** (0.51 g, 0.676 mmol) in a mixture of MeOH and $CH_2Cl_2$ (3 mL, 2:1) was added PTSA (167 mg, 0.878 mmol) at room temperature. The reaction mixture was stirred until disappearance of the starting material (monitored by TLC, 1.5 h). Then, it was concentrated *in vacuo*, diluted with $CH_2Cl_2$, and washed with satd. aq. $NaHCO_3$, and brine. The combined organic layer was dried over $MgSO_4$, filtered, and concentrated *in vacuo*. The resulting residue was purified by silica gel column chromatography (EtOAc/*n*-Hexane, 1 : 3) to give pure compound **S27** as a viscous liquid (295 mg, 86%). $^1$H NMR (600 MHz, $CDCl_3$): $\delta_H$ 7.36-7.28 (m, 20H), 4.78 (d, 1H, $J$ = 11.8 Hz), 4.73 (q, 2H, $J$ = 11.4 Hz), 4.68 (d, 1H, $J$ = 11.8 Hz), 4.61 (s, 2H), 4.56 (q, 2H, $J$ = 11.8 Hz), 3.99 (t, 1H, $J$ = 4.8 Hz), 3.94 (q, 2H, $J$ = 4.8 Hz), 3.79-3.71 (m, 5H), 2.32 (brs, 1H). $^{13}$C NMR (150 MHz, $CDCl_3$): $\delta_C$ 138.5, 138.4, 138.3 (2), 128.6 (2), 128.5 (2), 128.3, 128.1, 128.0, 127.9 (3), 127.8 (2), 79.3, 79.0, 78.4, 74.2, 73.5, 72.6, 72.1, 69.9, 61.6. HRMS (ESI) m/z: calcd for $C_{33}H_{36}O_5Na$ $[M+Na]^+$: 535.2455, found: 535.2457.

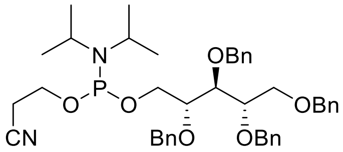


**1,2,3,4-Tetra-*O*-benzyl-5-([*N,N*-diisopropyl]-[2-cyanoethyl]-phosphoramidite)-D-ribitol (41).** To a soultion of alcohol **S27** (0.25 g, 0.488 mmol) in a mixture of anhydrous $CH_2Cl_2$ and $CH_3CN$ (6 mL, 2:1) were added 2-cyanoethyl *N,N,N',N'*-tetraisopropylphosphorodiamidite (620 µL, 1.95 mmol) and diisopropyl-ammonium tetrazolide (100 mg, 0.585 mmol) at room temperature under an argon atmosphere. The reaction mixture was stirred until completion (monitored by TLC, 1 h). Then, it was diluted with $CH_2Cl_2$ (5 mL), washed with satd. aq. $NaHCO_3$ (5 mL), dried over $MgSO_4$, filtered, and concentrated *in vacuo*. The obtained residue was purified by neutral alumina ($Al_2O_3$) column chromatography (EtOAc/*n*-Hexane, 1 : 4) under cold condition to afford pure compound **41** (300 mg, 88%) as a viscous liquid. Diastereomeic mixture: $^1$H NMR (600 MHz, $CDCl_3$): $\delta_H$ 7.38-7.28 (m, 35H), 4.77-4.58 (m, 10.5H), 4.55-4.49 (m, 3.5H), 4.02-3.97 (m, 2.5H), 3.93-3.85 (m, 6H), 3.83-3.70 (m, 8H), 3.66-3.60 (m, 4H), 2.69-2.67 (m, 0.7H), 2.53-2.46 (m, 3H), 1.21 (d, 12H, $J$ = 6.8 Hz), 1.18 (d, 4.5H, $J$ = 6.6 Hz), 1.17 (d, 4.5H, $J$ = 6.6 Hz). $^{13}$C NMR (150 MHz, $CDCl_3$): $\delta_C$ 138.9 (2), 138.8, 138.7 (2), 138.6 (2), 128.5, 128.4 (2), 128.1 (2), 127.9 (3), 127.8, 127.7 (2), 127.6 (2), 117.9, 117.8, 79.4,

79.3 (2), 79.2, 78.9, 78.8 (2), 78.7, 73.9 (2), 73.4, 72.6, 72.5 (2), 70.5, 70.4, 63.6, 63.5, 63.3, 63.2, 58.7, 58.6, 58.5 (2), 58.4, 43.4, 43.3 (3), 43.2, 24.8 (2), 20.5, 20.4 (3). HRMS (ESI) m/z: calcd for $C_{42}H_{53}N_2O_6PNa$ $[M+Na]^+$: 735.3533, found: 735.3545.

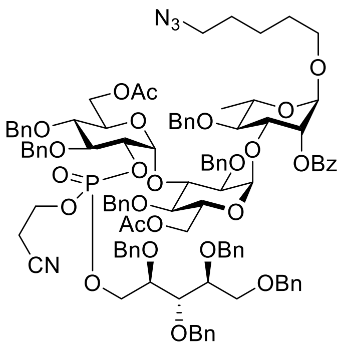


**5-Azidopentyl 1,2,3,4-tetra-*O*-benzyl-D-ribityl-(1→[2-cyanoethyl]-phosphate→2)-6-*O*-acetyl-3,4-di-*O*-benzyl-α-D-glucopyranosyl-(1→3)-6-*O*-acetyl-2,4-di-*O*-benzyl-α-D-glucopyranosyl-(1→3)-2-*O*-benzoyl-4-*O*-benzyl-α-L-rhamnopyranoside (42).** To a stirred mixture of alcohol **40** (126 mg, 0.102 mmol) and 5-ethylthio-1*H*-tetrazole (132 mg, 1.018 mmol) in anhydrous $CH_3CN$ (3 mL) under an argon atmosphere was added freshly activated 3 Å MS (250 mg) at rt. After being stirred for 10 min, ribitol phosphoramidite **41** (145 mg, 0.203 mmol) in anhydrous $CH_3CN$ (2 mL) was added. The reaction mixture was stirred for 1 h (monitored by TLC). Then, $H_2O$ (1 mL) and $I_2$ (387 mg, 1.527 mmol) in THF (3 mL) were added respectively. The mixture was stirred for further 2 h at rt before diluted with EtOAc. The reaction residue was filtered through a pad of Celite, washed with satd. aq. $Na_2S_2O_3$ (15 mL), and satd. aq. $NaHCO_3$ (5 mL). The organic layer was dried over $MgSO_4$, filtered, and concentrated *in vacuo*. The residue was purified by silica gel column chromatography (EtOAc/*n*-Hexane, 1 : 2) to give **42** as a colourless viscous liquid (108 mg, 57%, mixture of diastereomers) and recoverd acceptor **40** (47 mg). Upper diastereomer: $^1$H NMR (600 MHz, $CDCl_3$): $\delta_H$ 7.94 (d, 2H, $J$ = 7.3 Hz), 7.50 (t, 1H, $J$ = 7.3 Hz), 7.37 (t, 2H, $J$ = 7.8 Hz), 7.30-7.13 (m, 38H), 7.06-7.04 (m, 3H), 7.01-6.98 (m, 2H), 6.94-6.91 (m, 2H), 5.59 (d, 1H, $J$ = 3.4 Hz), 5.48 (t, 1H, $J$ = 1.9 Hz), 5.27 (d, 1H, $J$ = 3.4 Hz), 4.95 (d, 1H, $J$ = 11.3 Hz), 4.92 (d, 1H, $J$ = 10.6 Hz), 4.83-4.78 (m, 2H), 4.73 (d, 1H, $J$ = 11.1 Hz), 4.65-4.58 (m, 3H), 4.50 (dd, 2H, $J$ = 3.4, 11.2 Hz), 4.46-4.28 (m, 12H), 4.26-4.21 (m, 3H), 4.16-4.08 (m, 3H), 4.06-4.01 (m, 2H), 3.93 (t, 1H, $J$ = 9.4 Hz), 3.83 (dd, 1H, $J$ = 3.3, 12.2 Hz), 3.77 (dd, 1H, $J$ = 6.0, 9.4 Hz), 3.72-3.69 (m, 2H), 3.65-3.55 (m, 7H), 3.49 (dd, 1H, $J$ = 3.4, 9.5 Hz), 3.43-3.38 (m, 2H), 3.27 (t, 2H, $J$ = 6.8 Hz), 1.98 (s, 3H), 1.89 (s, 3H), 1.80-1.77 (m, 2H), 1.62-1.58 (m, 4H), 1.41-1.38 (m, 2H), 1.35 (d, 3H, $J$ = 6.2 Hz). $^{13}$C NMR (150 MHz, $CDCl_3$): $\delta_C$ 170.8 (2), 166.3, 138.5, 138.4 (2), 138.3, 138.1, 137.9, 137.7, 137.1, 133.5, 130.0, 129.5, 128.7, 128.6 (2), 128.5 (2), 128.3, 128.2, 128.1, 128.0, 127.9, 127.8 (3), 127.7 (2), 116.4, 97.5 (C1, $^1J_{CH}$ = 172 Hz), 96.5 (C1, $^1J_{CH}$ = 173 Hz), 91.4 (C1, $^1J_{CH}$ = 172 Hz), 80.1 (2), 79.7, 78.6, 78.2, 78.2, 78.1, 78.0, 77.7, 76.2, 76.1, 75.4, 75.1, 74.1, 73.6, 73.5, 72.5, 72.4, 72.1, 69.7, 68.8, 68.6 (2), 68.5, 68.4, 68.3, 67.9, 62.8, 62.7, 61.9 (2), 51.5, 29.1, 28.8, 23.5, 21.0 (2), 18.8, 18.7, 18.2. $^{31}$P NMR (202 MHz, $CDCl_3$): δ 0.215. HRMS (ESI) m/z: calcd for $C_{105}H_{118}N_4O_{25}P$ $[M+H]^+$: 1865.7817, found: 1865.7859.

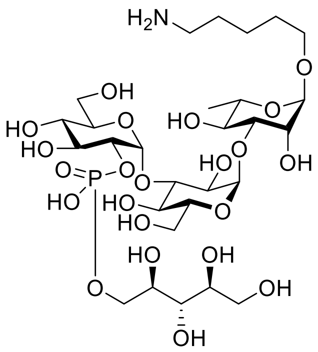

**5-Aminopentyl D-ribityl-(1→hydrogen phosphate→2)-α-D-glucopyranosyl-(1→3)-α-D-glucopyranosyl-α-L-rhamnopyranoide (13).** *Prepared according to general deprotection procedure C* using phosphosugar **42** (85 mg, 0.044 mmol), mixture of solvents $CH_2Cl_2/H_2O$ (2 mL), and tetrabutylammonium hydroxide (80 µL, 0.087 mmol). Reaction time was 4 h. Second step with $CH_2Cl_2$ (1 mL) and NaOMe (3 mL, 0.3 M in MeOH). Reaction time was 24 h. Third step with mixture of solvents MeOH/$H_2O$/AcOH (4.25 mL) and $Pd(OH)_2/C$ (85 mg). Reaction time was 36 h. Compound **13** was obtained as an amorphous white solid (31 mg, 81% over 3 steps) after Sephadex LH-20 chromatography. $^1H$ NMR (600 MHz, $D_2O$): $\delta_H$ 5.63 (d, 1H, $J$ = 3.8 Hz), 5.09 (d, 1H, $J$ = 3.8 Hz), 4.86 (d, 1H, $J$ = 1.5 Hz), 4.17-4.14 (m, 2H), 4.08-4.00 (m, 4H), 3.99-3.96 (m, 2H), 3.89-3.80 (m, 8H), 3.78-3.72 (m, 4H), 3.70-3.66 (m, 2H), 3.61-3.55 (m, 3H), 3.02 (t, 2H, $J$ = 7.7 Hz), 1.74-1.64 (m, 4H), 1.54-1.42 (m, 2H), 1.34 (d, 3H, $J$ = 6.2 Hz). $^{13}C$ NMR (150 MHz, $D_2O$): $\delta_C$ 99.2 (C1, $^1J_{CH}$ = 172 Hz), 97.4 (C1, $^1J_{CH}$ = 172 Hz), 95.4 (C1, $^1J_{CH}$ = 171 Hz), 79.3, 75.4 (2), 71.9, 71.7 (2), 71.4, 71.2, 70.9, 70.8, 70.1, 69.7 (2), 68.9, 68.7, 67.4, 66.7 (2), 66.6, 62.3, 60.1, 60.0, 39.3, 28.0, 26.5, 22.4, 16.6. $^{31}P$ NMR (202 MHz, $D_2O$): δ 1.27. HRMS (ESI) m/z: calcd for $C_{28}H_{55}NO_{22}P$ $[M+H]^+$: 788.2948, found: 788.2949.

## Carrier protein incorporation of synthetic oligosaccharides

### Modification of the synthetic oligosaccharides

3,3′-Dithiobis(sulfosuccinimidylpropionate (DTSSP) (2 equiv) was added to the solution of free amine glycan (1 equiv) in PBS buffer having pH 8 (0.75 mL) at room temperature and the pH was adjusted to 7-8 by adding 1 N $NaOH_{(aq)}$ dropwise every 30 min up to 2 h before allowed to stir overnight. Thereafter, dithiothreitol (DTT) (4 equiv) was added to the reaction mixture. The reaction mixture was stirred at 40 °C for further 1.5 h. The crude reaction mixture was loaded on Bio-gel P-2 column directly and eluted with distilled $H_2O$. Fracations containing the product were pooled and lyophilized to give desired thiolated compound.

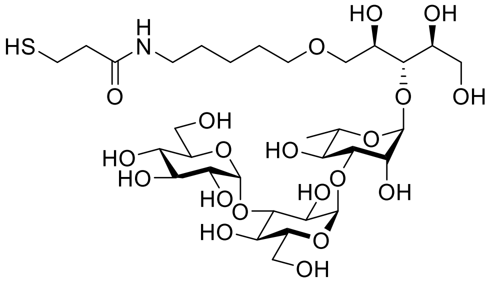


**Compound 5-SH.** *Prepared according to the above procedure* using DTSSP (13.7 mg, 0.0226 mmol), glycan **5** (8 mg, 0.0113 mmol) and DTT (7 mg, 0.045 mmol). Compound **5-SH** was obtained as a white solid (5.5 mg, 61%) after P-2 gel column chromatography. $^{1}$H NMR (600 MHz, $D_2O$): $\delta_H$ 5.37 (d, 1H, $J$ = 3.6 Hz), 5.09 (d, 1H, $J$ = 3.8 Hz), 5.01 (d, 1H, $J$ = 1.5 Hz), 4.19 (t, 1H, $J$ = 2.4 Hz), 4.08-4.06 (m, 1H), 4.01 (ddd, 2H, $J$ = 3.4. 6.3, 10.1 Hz), 3.94-3.91 (m, 2H), 3.87-3.74 (m, 9H), 3.72-3.62 (m, 4H), 3.59-3.52 (m, 5H), 3.48 (t, 1H, $J$ = 9.6 Hz), 3.23 (t, 2H, $J$ = 6.7 Hz), 2.78 (t, 2H, $J$ = 6.7 Hz), 2.54 (t, 2H, $J$ = 6.7 Hz), 1.64-1.52 (m, 4H), 1.41-1.36 (m, 2H), 1.31 (d, 3H, $J$ = 6.2 Hz). $^{13}$C NMR (150 MHz, $D_2O$): $\delta_C$ 174.2, 100.1, 99.2, 99.5, 79.9, 79.6, 75.3, 72.8, 71.6, 71.3, 71.1, 70.90, 70.6, 70.1, 69.9, 69.8, 69.4, 69.2, 69.0, 66.9, 62.6, 60.1, 59.9, 39.4, 39.2, 28.2, 28.0, 22.6, 20.0, 16.6. HRMS (ESI) m/z: calcd for $C_{31}H_{57}NO_{20}SNa$ $[M+Na]^+$: 818.3087, found: 818.3090.

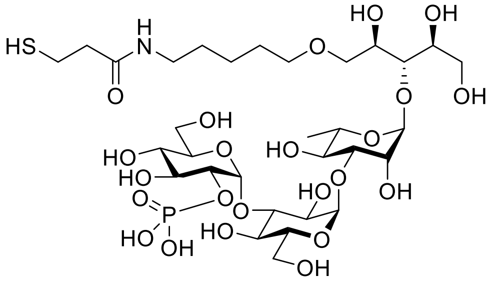


**Compound 6-SH.** *Prepared according to the above procedure* using DTSSP (12.3 mg, 0.0203 mmol), glycan **6** (8 mg, 0.0101 mmol) and DTT (6.3 mg, 0.0406 mmol). Compound **6-SH** was obtained as a white foam (8.1 mg, 91%) after P-2 gel column chromatography. $^{1}$H NMR (600 MHz, $D_2O$): $\delta_H$ 5.60 (d, 1H, $J$ = 3.4 Hz), 5.07 (d, 1H, $J$ = 3.5 Hz), 5.01 (s, 1H,), 4.18 (s, 1H), 4.08 (t, 1H, $J$ = 6.5 Hz), 4.03 (ddd, 1H, $J$ = 3.1, 6.0, 13.2 Hz), 3.98-3.90 (m, 4H), 3.86-3.73 (m, 10H), 3.69-3.62 (m, 3H), 3.60-3.52 (m, 5H), 3.22 (t, 2H, $J$ = 6.7 Hz), 2.78 (t, 2H, $J$ = 6.7 Hz), 2.54 (t, 2H, $J$ = 6.7 Hz), 1.65-1.52 (m, 4H), 1.48-1.43 (m, 2H), 1.33 (d, 3H, $J$ = 6.4 Hz). $^{13}$C NMR (150 MHz, $D_2O$): $\delta_C$ 174.2, 100.1, 97.8, 95.6, 79.9, 79.6, 75.3, 74.1 (2), 72.3 (2), 71.6, 71.2, 71.1, 70.9, 69.9, 69.6, 69.4 (2), 69.0, 68.9, 66.9, 62.6, 60.0, 39.5, 39.2, 28.2, 28.0, 22.6, 20.0, 16.6. HRMS (ESI) m/z: calcd for $C_{31}H_{59}NO_{23}PS$ $[M+H]^+$: 876.2931, found: 876.2945.

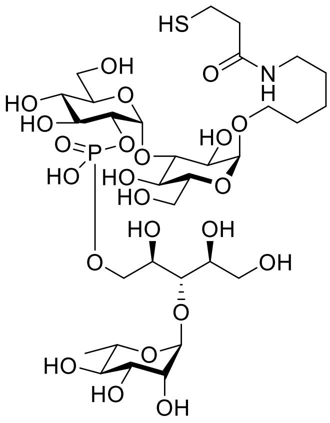

**Compound 7-SH.** *Prepared according to the above procedure* using DTSSP (12.3 mg, 0.0203 mmol), glycan **7** (8 mg, 0.0101 mmol) and DTT (6.25 mg, 0.0406 mmol). Compound **7-SH** was obtained as a white solid (7.9 mg, 89%) after P-2 gel column chromatography. $^{1}$H NMR (600 MHz, $D_2O$): $\delta_H$ 5.61 (d, 1H, $J$ = 3.9 Hz), 4.97 (d, 1H, $J$ = 1.5 Hz), 4.90 (d, 1H, $J$ = 3.7 Hz), 4.14-4.09 (m, 2H), 4.08-3.99 (m, 4H), 3.66 (td, 1H, $J$ = 4.1, 7.8 Hz), 3.90-3.75 (m, 10H), 3.73-3.63 (m, 5H), 3.55-3.49 (m, 2H), 3.47 (t, 1H, $J$ = 9.6 Hz), 3.23 (t, 1H, $J$ = 6.8 Hz), 2.78 (t, 2H, $J$ = 6.5 Hz), 2.55 (t, 2H, $J$ = 6.8 Hz), 1.68-1.61 (m, 2H), 1.58-1.54 (m, 2H), 1.47-1.37 (m, 2H), 1.29 (d, 3H, $J$ = 6.3 Hz). $^{13}$C NMR (150 MHz, $D_2O$): $\delta_C$ 174.1, 101.0, 98.21, 97.1, 79.7, 79.3, 75.3 (2), 71.9, 71.8, 71.6 (2), 71.4 (2), 70.2, 70.0, 69.9, 69.5, 69.4, 69.3, 69.1, 67.9, 66.8 (2), 62.6, 60.4, 60.3, 39.4, 39.2, 28.2, 28.0, 22.8, 19.9, 16.5. HRMS (ESI) m/z: calcd for $C_{31}H_{59}NO_{23}PS$ $[M+H]^+$: 876.2931, found: 876.2951.

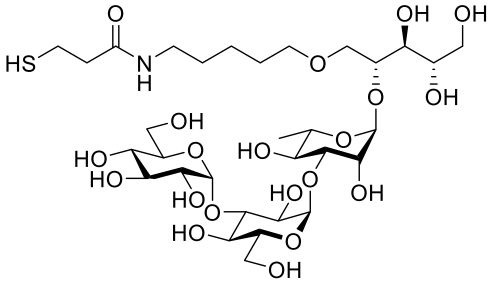

**Compound 9-SH.** *Prepared according to the above procedure* using DTSSP (13.7 mg, 0.0226 mmol), glycan **9** (8 mg, 0.0113 mmol) and DTT (7 mg, 0.045 mmol). Compound **9-SH** was obtained as a white solid (5.6 mg, 63%) after P-2 gel column chromatography. $^{1}$H NMR (600 MHz, $D_2O$): $\delta_H$ 5.37 (d, 1H, $J$ = 3.9 Hz), 5.09 (m, 2H, two anomeric signals overlapped), 4.19 (t, 1H, $J$ = 2.5 Hz), 4.07-4.06 (m, 1H), 4.02-3.99 (m, 2H), 3.94 (t, 1H, $J$ = 9.4 Hz), 3.88 (dd, 1H, $J$ = 2.9, 9.6 Hz), 3.84-3.74 (m, 9H), 3.73-3.66 (m, 4H), 3.65-3.53 (m, 5H), 3.48 (t, 1H, $J$ = 9.7 Hz), 3.22 (t, 2H, $J$ = 6.8 Hz), 2.78 (t, 2H, $J$ = 6.7 Hz), 2.54 (t, 2H, $J$ = 6.7 Hz), 1.63-1.58 (m, 2H), 1.57-1.52 (m, 2H), 1.41-1.36 (m, 2H), 1.32 (d, 3H, $J$ = 6.2 Hz). $^{13}$C NMR (150 MHz, $D_2O$): $\delta_C$ 174.2, 99.6, 99.2, 95.7, 79.6, 76.5, 75.5, 72.8, 72.1, 71.6, 71.4, 71.3, 71.2, 70.1, 69.9, 69.7, 69.3, 69.2, 68.8, 67.1, 62.5, 60.1, 59.9, 39.5, 39.2, 28.1, 27.9, 22.6, 20.0, 16.6. HRMS (ESI) m/z: calcd for $C_{31}H_{57}NO_{20}SNa$ $[M+Na]^+$: 818.3087, found: 818.3089.

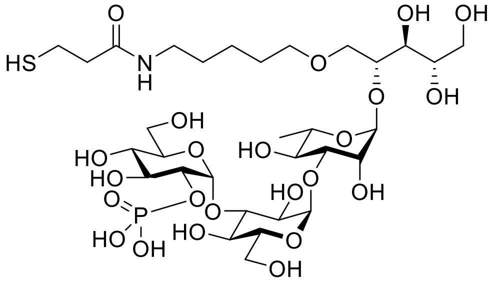


**Compound 10-SH.** *Prepared according to the above procedure* using DTSSP (10.8 mg, 0.0177 mmol), glycan **10** (7 mg, 0.0089 mmol) and DTT (5.5 mg, 0.0355 mmol). Compound **10-SH** was obtained as a white solid (6.7 mg, 86%) after P-2 gel column chromatography. $^{1}$H NMR (600 MHz, $D_2O$): $\delta_H$ 5.59 (d, 1H, $J$ = 3.8 Hz), 5.08-5.07 (m, 2H, two anomeric signals overlapped), 4.19 (t, 1H, $J$ = 2.3 Hz), 4.07-3.97 (m, 4H), 3.95 (t, 1H, $J$ = 9.1 Hz), 3.88-3.77 (m, 10H), 3.75-3.53 (m, 10H), 3.22 (t, 2H, $J$ = 6.8 Hz), 2.78 (t, 2H, $J$ = 6.6 Hz), 2.55 (t, 2H, $J$ = 6.8 Hz), 1.63-1.52 (m, 4H), 1.41-1.36 (m, 2H), 1.32 (d, 3H, $J$ = 6.2 Hz). $^{13}$C NMR (150 MHz, $D_2O$): $\delta_C$ 174.1, 99.6, 97.6, 95.7, 79.5, 76.5, 75.5, 74.7 (2), 72.1, 71.9 (2), 71.4, 71.3, 71.2, 71.1, 70.9, 70.1, 69.6, 69.5, 69.3, 68.8, 67.1, 62.6, 60.0, 59.9, 39.3, 39.2, 28.2, 27.9, 22.6, 19.9, 16.6. HRMS (ESI) m/z: calcd for $C_{31}H_{59}NO_{23}PS$ $[M+H]^+$: 876.2931, found: 876.2940.

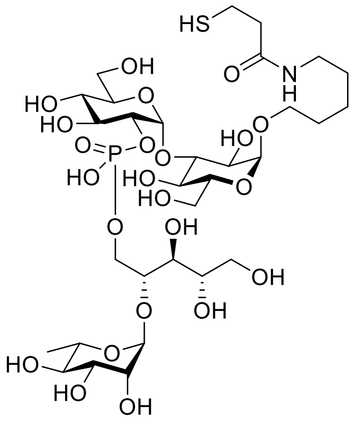


**Compound 11-SH.** *Prepared according to the above procedure* using DTSSP (12.3 mg, 0.0203 mmol), glycan **11** (8 mg, 0.0101 mmol) and DTT (6.25 mg, 0.0406 mmol). Compound **11-SH** was obtained as a white solid (7.5 mg, 84%) after P-2 gel column chromatography. $^{1}$H NMR (600 MHz, $D_2O$): $\delta_H$ 5.61 (d, 1H, $J$ = 3.6 Hz), 5.08 (s, 1H), 4.90 (d, 1H, $J$ = 3.6 Hz), 4.25-4.21 (m, 1H), 4.12-4.06 (m, 4H), 4.00-3.97 (m, 1H), 3.90-3.70 (m, 12H), 3.69-3.62 (m, 4H), 3.55-3.44 (m, 3H), 3.23 (t, 2H, $J$ = 6.8 Hz), 2.78 (t, 2H, $J$ = 6.8 Hz), 2.54 (t, 2H, $J$ = 6.8 Hz), 1.72-1.60 (m, 2H), 1.58-1.53 (m, 2H), 1.48-1.35 (m, 2H), 1.29 (d, 3H, $J$ = 6.4 Hz). $^{13}$C NMR (150 MHz, $D_2O$): $\delta_C$ 174.2, 100.2, 98.1, 97.1, 79.2, 77.0, 76.9, 75.4 (2), 71.9 (2), 71.6 (2), 71.4, 71.3, 71.2, 70.2, 70.0, 69.9, 69.5, 69.2, 69.1, 67.9, 64.4 (2), 62.5, 60.4, 60.3, 39.5, 39.2, 28.2, 28.0, 22.8, 20.0, 16.5. HRMS (ESI) m/z: calcd for $C_{31}H_{59}NO_{23}PS$ $[M+H]^+$: 876.2931, found: 876.2949.

**Oligosaccharide conjugation to carrier protein CRM197**

Water washed CRM197 (cross reacting material 197, 1 equiv) was dissolved in PBS buffer pH 8.0 before cross-linker SBAP (succinimidyl 3-(bromoacetamido)propionate; 60 equiv) was added. The

mixture was stirred at room temperature for 2 h. The reaction was washed by PBS buffer pH 9.0 through centrifugation by Amicon. The resulting activated CRM197 (in PBS buffer pH 9.0) and dissolved sulfhydryl oligosaccharides (in PBS buffer pH 9.0; prepared as described above) were mixed at equal weights and stirred 2 h at room temperature. The reactions were quenched by cysteine and washed by PBS buffer pH 7.4 through centrifugation by Amicon. The resulting glycoconjugates (**C1**–**C3** and **D1**–**D3**) were analyzed by MALDI-TOF (positive mode, sinapinic acid) for determination of incorporation characteristics. The concentration of CRM197 and glycoconjugates were determined by NanoDrop (NanoDrop Lite Spectrophotometer, Thermo Scientific).

**Table S1.** MALDI-TOF analysis for glycoconjugates.

| Conjugates | m/z | Average glycan number |
|---|---|---|
| CRM197 | 58367 | – |
| **C1** (CRM197-**5**) | 68178 | 9.15 |
| **C2** (CRM197-**6**) | 66628 | 6.55 |
| **C3** (CRM197-**7**) | 66909 | 6.87 |
| **D1** (CRM197-**9**) | 66780 | 7.40 |
| **D2** (CRM197-**10**) | 66314 | 6.19 |
| **D3** (CRM197-**11**) | 66900 | 6.86 |

## Immunization experiments

### Vaccine formulation

Each immunogen (glycoconjugates **C1**–**C3** and **D1**–**D3**, and CRM197) was mixed with 161 μL of aluminium hydroxide gel (Alhydrogel adjuvant 2%, InvivoGen) in phosphate buffer (PBS pH 7.4, gibco), making a total volume of 550 μL. The mixture was rotated gently at 4 $^{o}$C overnight to formulate the vaccine. The vaccine was then centrifuged at 3000 × g for 10 min for determination of the adsorption results. The adsorption of the proteins on alum was determined according to the non-adsorbed proteins in the supernatants. The non-adsorbed protein concentrations were determined by NanoDrop (NanoDrop Lite Spectrophotometer, Thermo Scientific). All the proteins were totally adsorbed onto alum. The vaccines were resuspended adequately before injection.

### Mouse immunization

Seven groups of five 8-week-old female BALB/c mice (BioLASCO, Taiwan) were immunized with glycoconjugates (**C1**–**C3** and **D1**–**D3**) and CRM197 (control) through intramuscular injection. Each dose contained 2.2 μg glycan in 100 μL vaccine suspension (prepared as described above). Each mouse was given three doses in two-week intervals. The mice were bled 10 days after the third immunization. The antisera were obtained by centrifugation at 1500 × g for 10 min.

## Glycan microarray

### Glycan immobilization on glass slides

Seventeen oligosaccharides (**1–13, 42-45**) with aminopentyl linkers were dissolved in printing buffer (300 mM sodium phosphate buffer, 0.005% Triton X-100, pH 8.5) to make 100 μM solutions and added into wells of a 384-well plate. The glycans were printed (BioDot, Cartesian Technologies) by robotic pin (SMP3, TeleChem International) deposition of ~0.6 nL of glycan solutions from the 384-well plate onto NHS-coated glass slides (Nexterion H slide, SCHOTT North America). The microarray was designed as 16 grids in one slide and 14 columns × 10 rows (5 spots for each glycan) in one grid. The printing slides were allowed to react in an atmosphere of 80% humidity for one hour, followed by desiccation overnight. The printed slides were stored in dry box at room temperature before use.

### Serologic assay with glycan microarray

The printed glass slides (prepared as described above) were treated with SuperBlock blocking buffer (Thermo Scientific) for 1 h at room temperature and then washed three times with PBST buffer (PBS buffer pH 7.4 with 0.05% Tween 20). The diluted mouse sera (diluted with 3% BSA/PBST into 1 : 300, 1 : 900 and 1 : 2700 dilutions) were introduced onto the slides and incubated at room temperature for 1 h. The excess sera were removed, and then the slides were treated with goat anti-mouse IgG antibody (Alexa Fluor 647-conjugated, Jackson ImmunoResearch) as secondary antibody for 1 h at room temperature in dark. The reacted slides were washed three times with PBST buffer and then scanned at wavelength 635 nm by microarray scanner (GenePix 4300A, Molecular Devices). The scanned images were analyzed by GenePix 7 software.

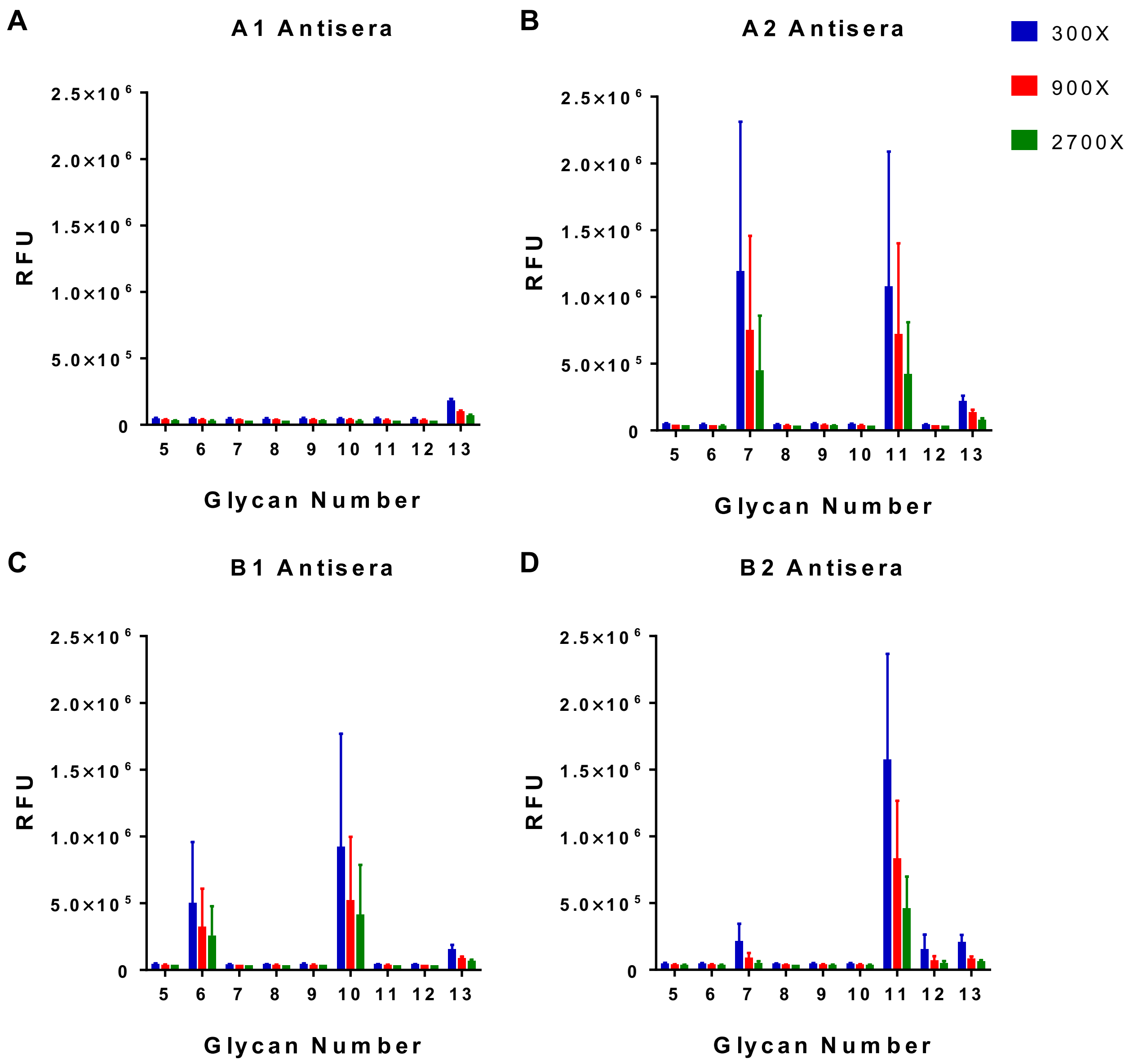


**Figure S1-S1.** Glycan microarray analysis of mouse antisera from ST6A and ST6B glycoconjugates immunization at different dilution ratios (1 : 300, 1 : 900, 1 : 2700). The binding profiles of the antisera with CPS **5-13** were examined. The data presented the mean ± SEM for each group of five mice. (A) A1 antisera. (B) A2 antisera. (C) B1 antisera. (D) B2 antisera. RFU, relative fluorescence unit.

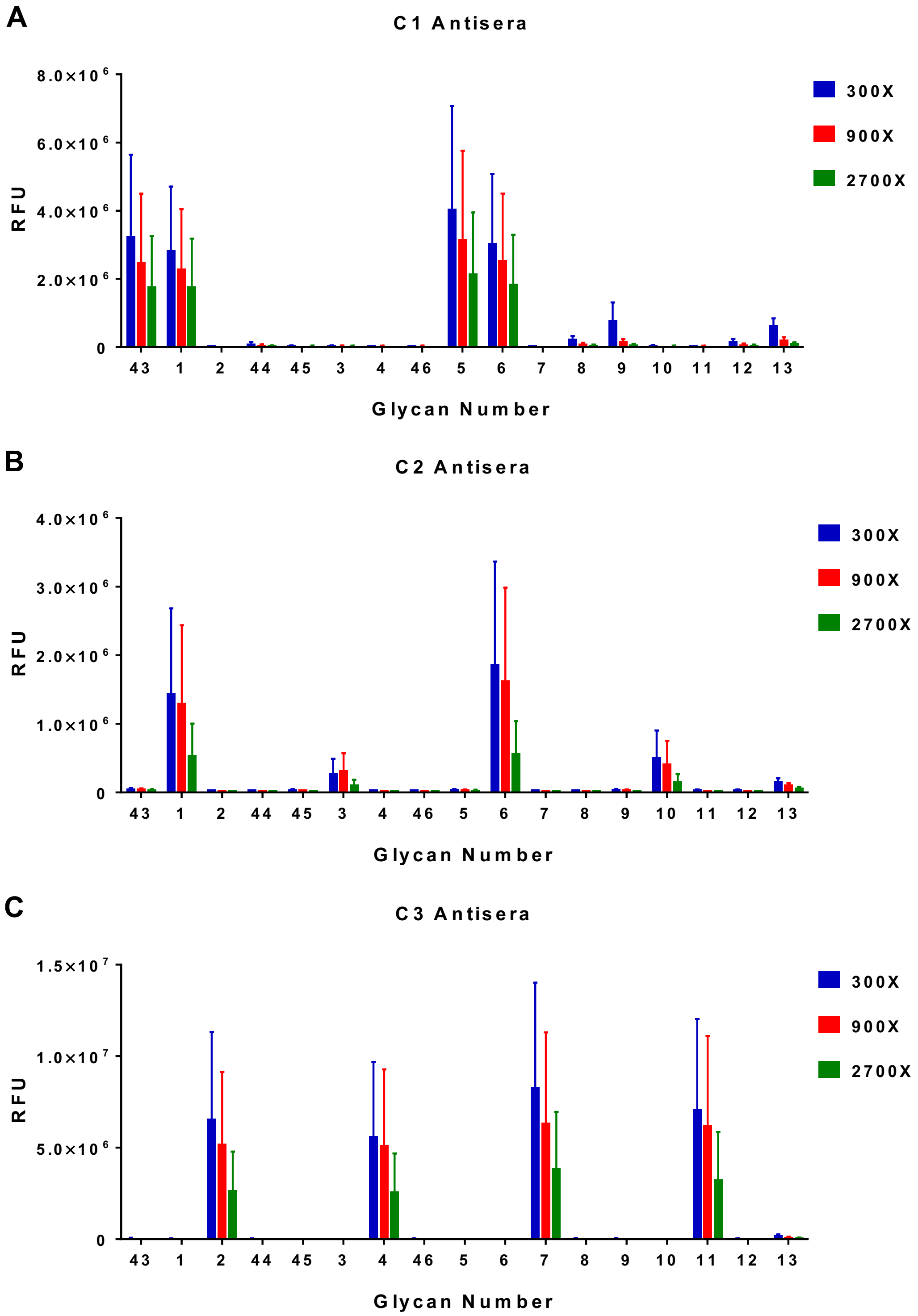


**Figure S1-S2.** Glycan microarray analysis of mouse antisera from ST6C glycoconjugates immunization at different dilution ratios (1 : 300, 1 : 900, 1 : 2700). Used glycan array with immobilized CPS **1-13** and also contained **43-46**. The data presented the mean ± SEM for each group of five mice. (A) C1 antisera. (B) C2 antisera. (C) C3 antisera. RFU, relative fluorescence unit.

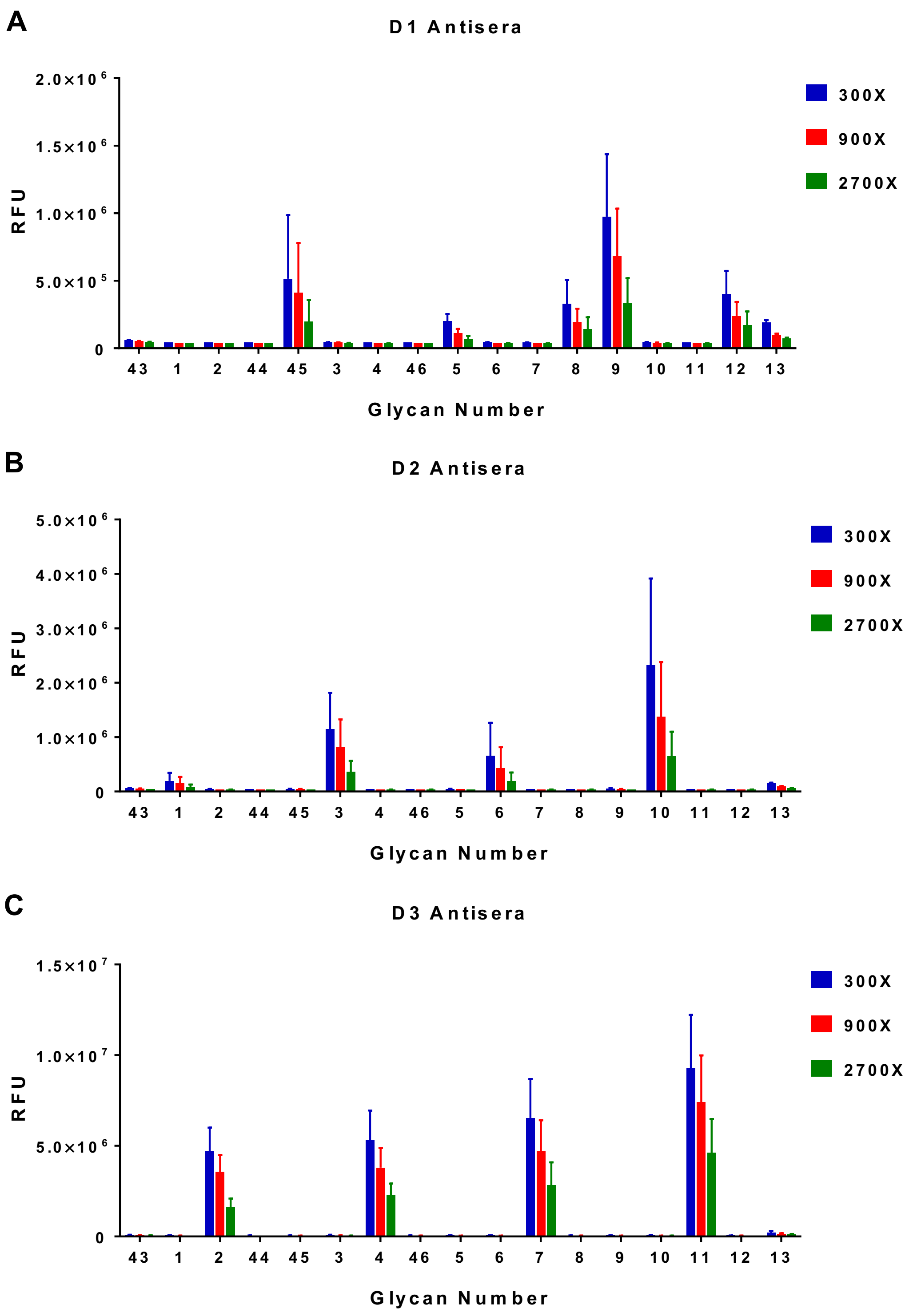


**Figure S1-S3.** Glycan microarray analysis of mouse antisera from ST6D glycoconjugates immunization at different dilution ratios (1 : 300, 1 : 900, 1 : 2700). Used glycan array with immobilized CPS **1-13** and also contained **43-46**. The data presented the mean ± SEM for each group of five mice. (A) D1 antisera. (B) D2 antisera. (C) D3 antisera. RFU, relative fluorescence unit.

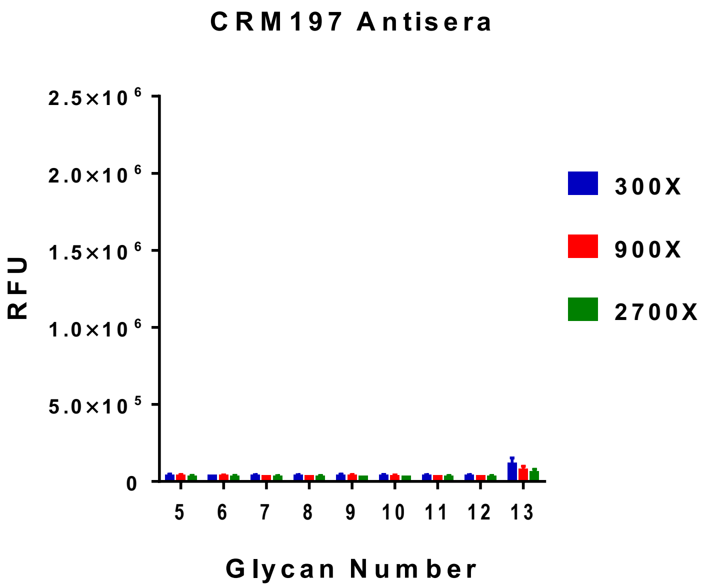


**Figure S1-S4.** Glycan microarray analysis of mouse antisera from CRM197 immunization at different dilution ratios (1 : 300, 1 : 900, 1 : 2700). The binding profiles of the antisera with CPS **5**-**13** were examined. The data presented the mean ± SEM for each group of five mice.

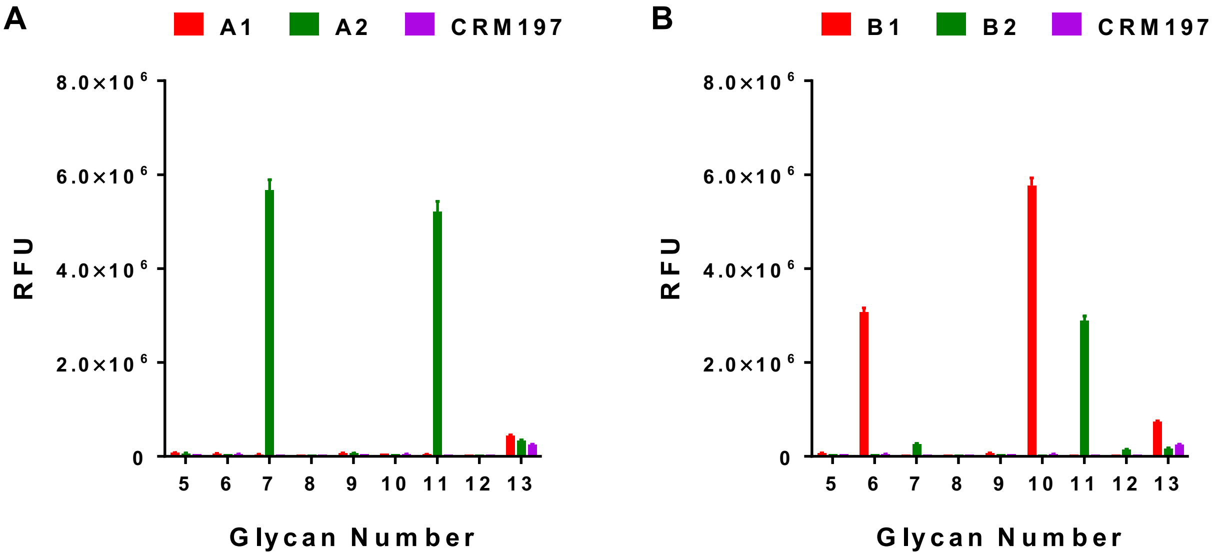


**Figure S1-S5.** Glycan microarray analysis of pooled antisera from ST6A and ST6B glycoconjugates immunization. The pooled antisera at 1 : 300 dilutions and examined the binding profiles of CPS 5-13. The presented data represents the mean ± SEM of five replicated spots for each glycan. (A) A1-A2 antisera. (B) B1-B2 antisera. RFU, relative fluorescence unit.

A

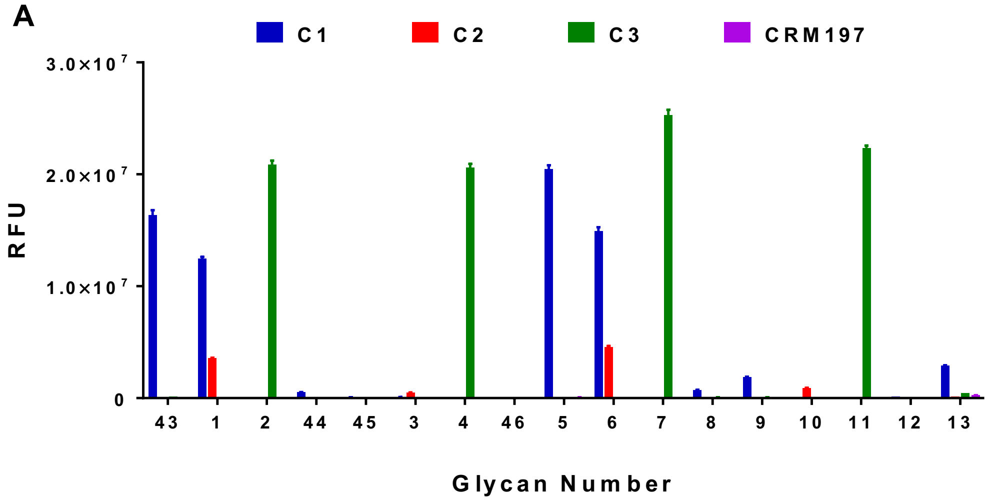


B

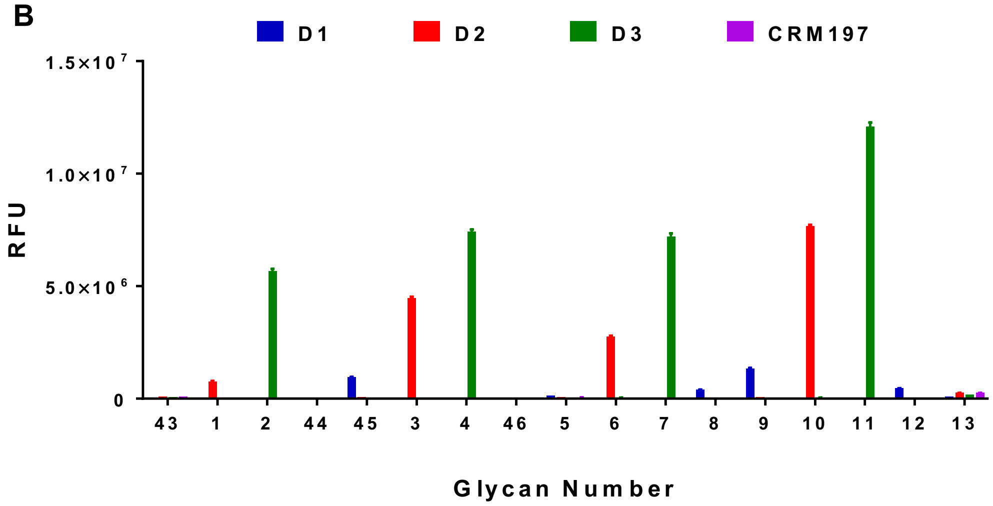


**Figure S1-S6.** Glycan microarray analysis of pooled antisera from ST6C and ST6D glycoconjugates immunization. The pooled antisera at 1 : 300 dilutions. The glycan array utilized immobilized CPS **1-13** and also contained **43-46**. The presented data represents the mean ± SEM of five replicated spots for each glycan. (A) C1-C3 antisera. (B) D1-D3 antisera. RFU, relative fluorescence unit.

## Opsonophagocytic killing assay

The in vitro opsonophagocytic killing assay was performed following the standard procedure.[3] Briefly, heat-inactivated mouse antisera (**A1**–**A2**, **B1**–**B2**, **C1**–**C3**, **D1**–**D3**, and CRM197 antisera) were diluted in two-fold series (from 1 : 10 to 1 : 640) with opsonization buffer (Hanks' balanced salt solution [HBSS] with Mg and Ca, 0.1% gelatin and 10% FBS) into a 96-well microtiter plate. Ten microliters of bacterial suspension (SPn6A, SPn6B, SPn6C, and SPn6D; ~$10^4$ CFU/well) was added to each well, and the mixtures were incubated at room temperature for 1 h with shaking on an orbital shaker at 250 rpm. New born rabbit complement (baby rabbit serum, final concentration of 12.5%) and differentiated effector HL-60 cells were added to each well (effector:bacterium ratio 400:1, final volume 80 µL/well). The plate was incubated in tissue culture incubator at 37 °C in 5% $CO_2$. After 90 min of incubation, each well was diluted with 80 µL of 0.9% NaCl (in opsonization buffer). Then,

aliquots of 10 μL from each well were applied onto tilted blood agar plates. The plates were incubated at 37 °C in 5% $CO_2$ overnight. Negative controls lacked antisera only. The percentages of killing were determined by CFU reduction relative to negative controls. Three repeated were performed independently.

## References

(1) Martin, C. E.; Weishaupt, M. W.; Seeberger, P. H. Progress toward developing a carbohydrate-conjugate vaccine against *Clostridium difficile* ribotype 027: synthesis of the cell-surface polysaccharide PS-I repeating unit. *Chem. Commun.* **2011**, *47*, 10260-10262.

(2) Mettu, R.; Lih, Y.-H.; Vulupala, H. R.; Chen, C.-Y.; Hsu, M.-H.; Lo, H.-J.; Liao, K.-S.; Cheng, Y.-Y.; Chiu, C.-H.; Wu, C.-Y. Synthetic Library of Oligosaccharides Derived from the Capsular Polysaccharide of *Streptococcus pneumoniae* Serotypes 6A and 6B and Their Immunological Studies. *ACS Infect. Dis.* **2022**, *8*, 626-634.

(3) Burton, R. L.; Nahm, M. H. Development and Validation of a Fourfold Multiplexed Opsonization Assay (MOPA4) for Pneumococcal Antibodies. *Clin. Vaccine Immunol.* **2006**, *13*, 1004-1009.
