# Supplementary material for: Chemical Synthesis of Truncated Capsular Oligosaccharide of Serotypes 6C and 6D of Streptococcus pneumoniae with Their Immunological Studies
Source: ACS Infect Dis. 2024 May 21;10(6):2161–71. doi: 10.1021/acsinfecdis.4c00147 (PMC11184553; doi:10.1021/acsinfecdis.4c00147)

Supporting Information – 2

Chemical Synthesis of Truncated Capsular Oligosaccharide of Serotypes 6C and 6D of *Streptococcus pneumoniae* with Their Immunological Studies

Ravinder Mettu,[1 #] Yang-Yu Cheng,[1,2 #] Hanmanth Reddy Vulupala,[1] Yu-Hsuan Lih,[1] Chiang-Yun Chen,[1] Mei-Hua Hsu,[3] Hong-Jay Lo,[1] Kuo-Shiang Liao,[1] Cheng-Hsun Chiu,[3] and Chung-Yi Wu[1]*

[1]Genomics Research Center, Academia Sinica, 128 Academia Road, Section 2, Nankang, Taipei, 11529 Taiwan

[2]Institute of Biochemistry and Molecular Biology, National Yang Ming Chiao Tung University, No. 155, Sec. 2, Linong St., Taipei 112304, Taiwan

[3]Molecular Infectious Disease Research Center, Chang Gung Memorial Hospital, Chang Gung University College of Medicine, 259 Wenhua 1st Road, Guishan, Taoyuan 33302, Taiwan

[#] These authors contributed equally.

Table of Contents

NMR spectra of glucose monosaccharides S3
NMR spectra of rhamnose monosaccharides S15
NMR spectra of ribitol S21
NMR spectra of Glc-Glc disaccharides S25
NMR spectra of Rha-Rbo pseudo-disaccharide S28
NMR spectra of trisaccharides S38
NMR spectra of pseudo-tetrasaccharides for ST6C S42
NMR spectra of pseudo-tetrasaccharides for ST6D S56
NMR spectra of common pseudo-tetrasaccharide synthesis S74
NMR spectra of ST6C and ST6D pseudo-tetrasaccharides S84
NMR spectra of thiolated pseudo-tetrasaccharides S101

NMR spectra of glucose monosaccharides

S2: ^{1}H NMR (600 MHz, $CDCl_3$)

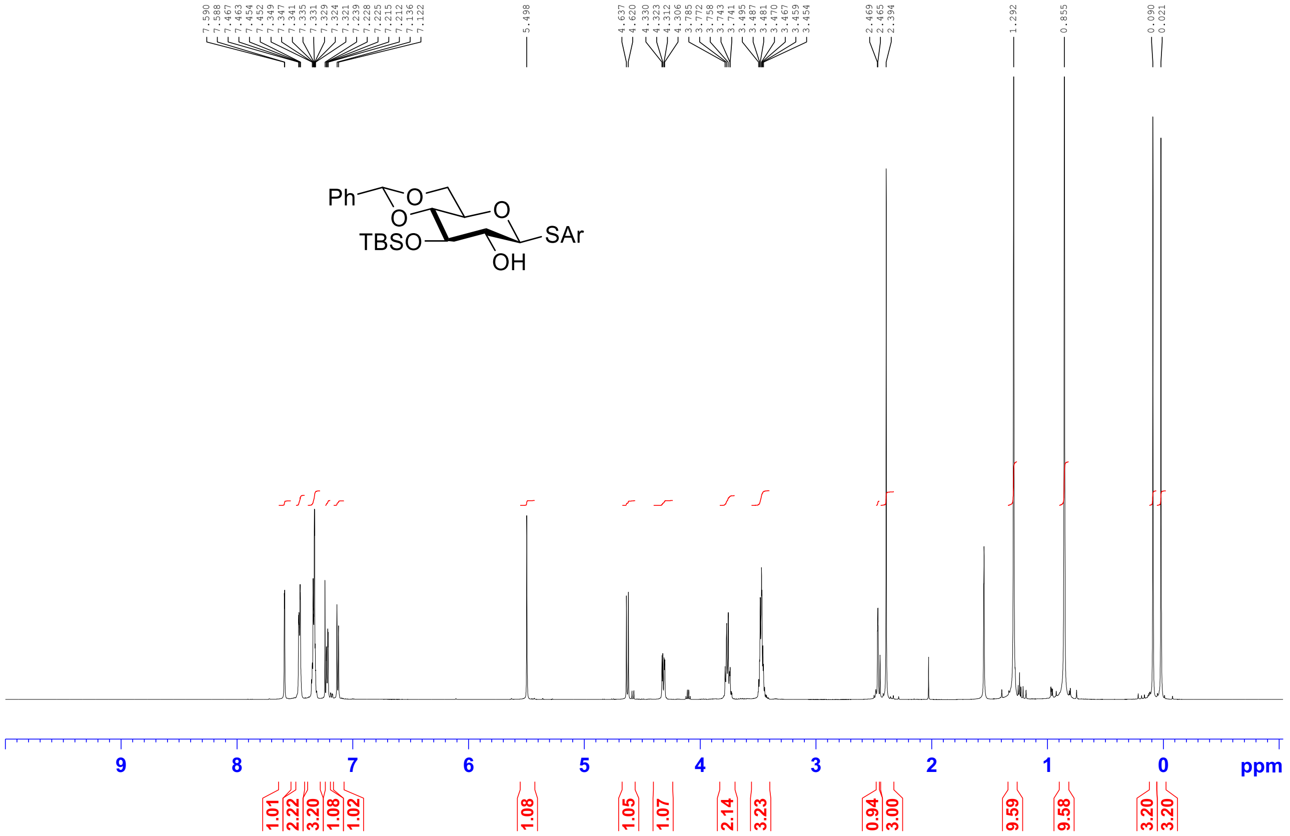

S2: ^{13}C NMR (150 MHz, $CDCl_3$)

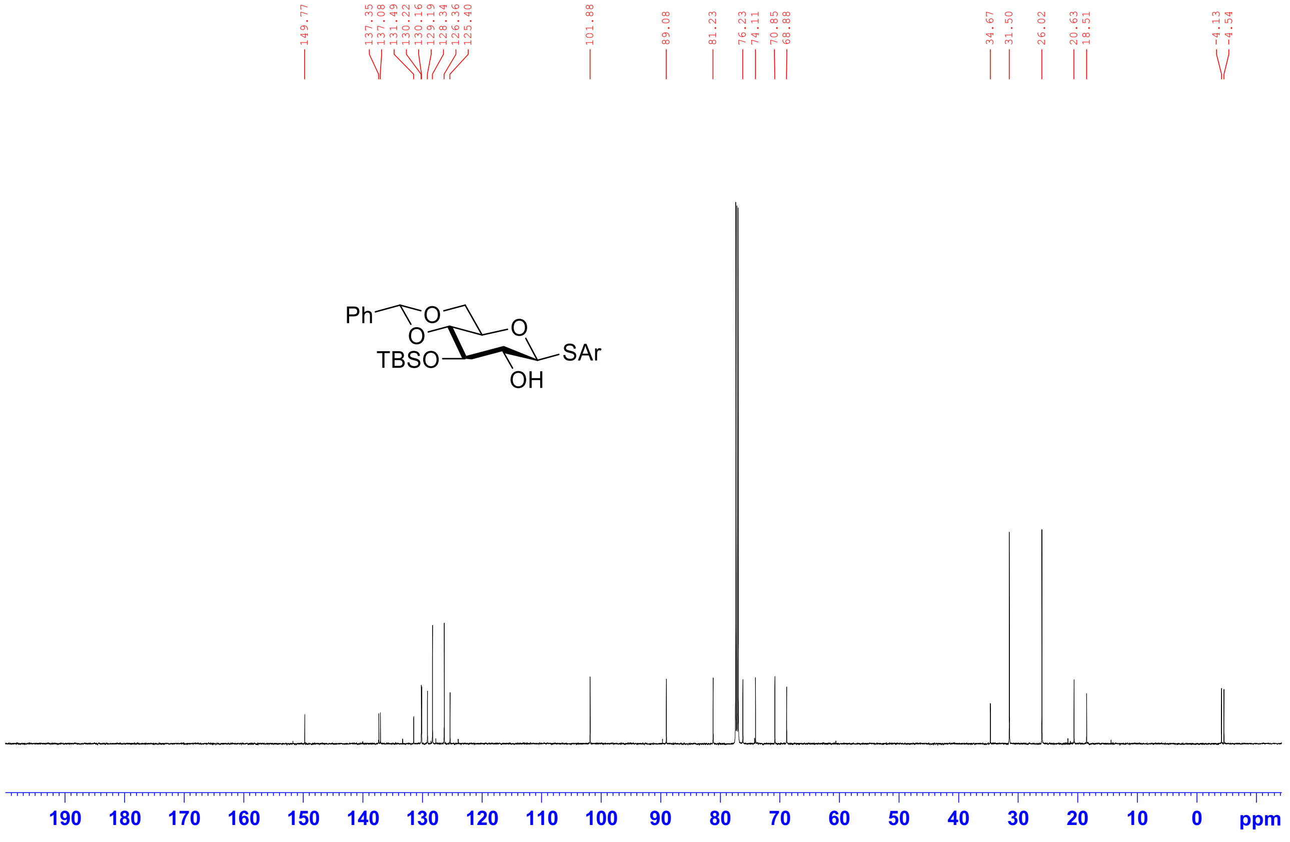

S2: DEPT 135 (150 MHz, $CDCl_3$)

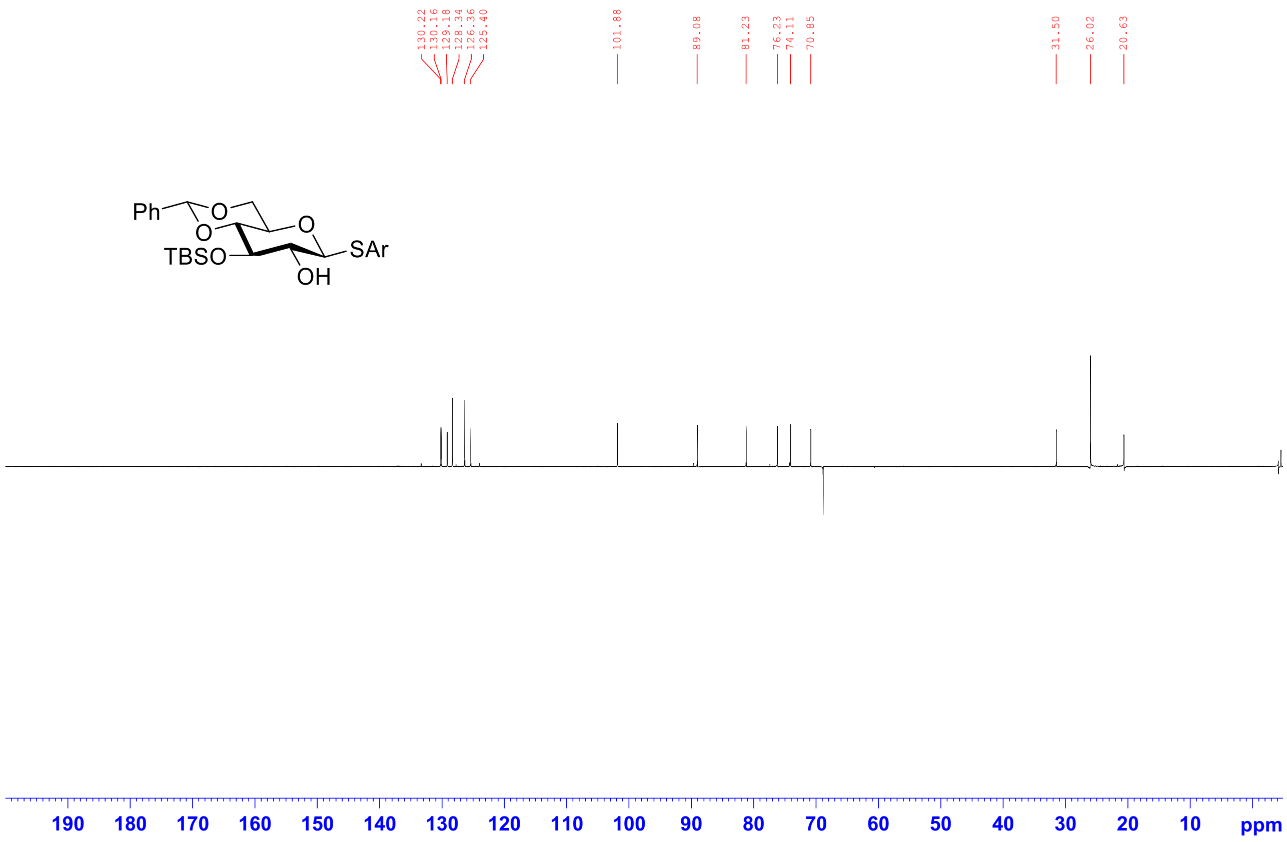

S4: ^{1}H NMR (600 MHz, $CDCl_3$)

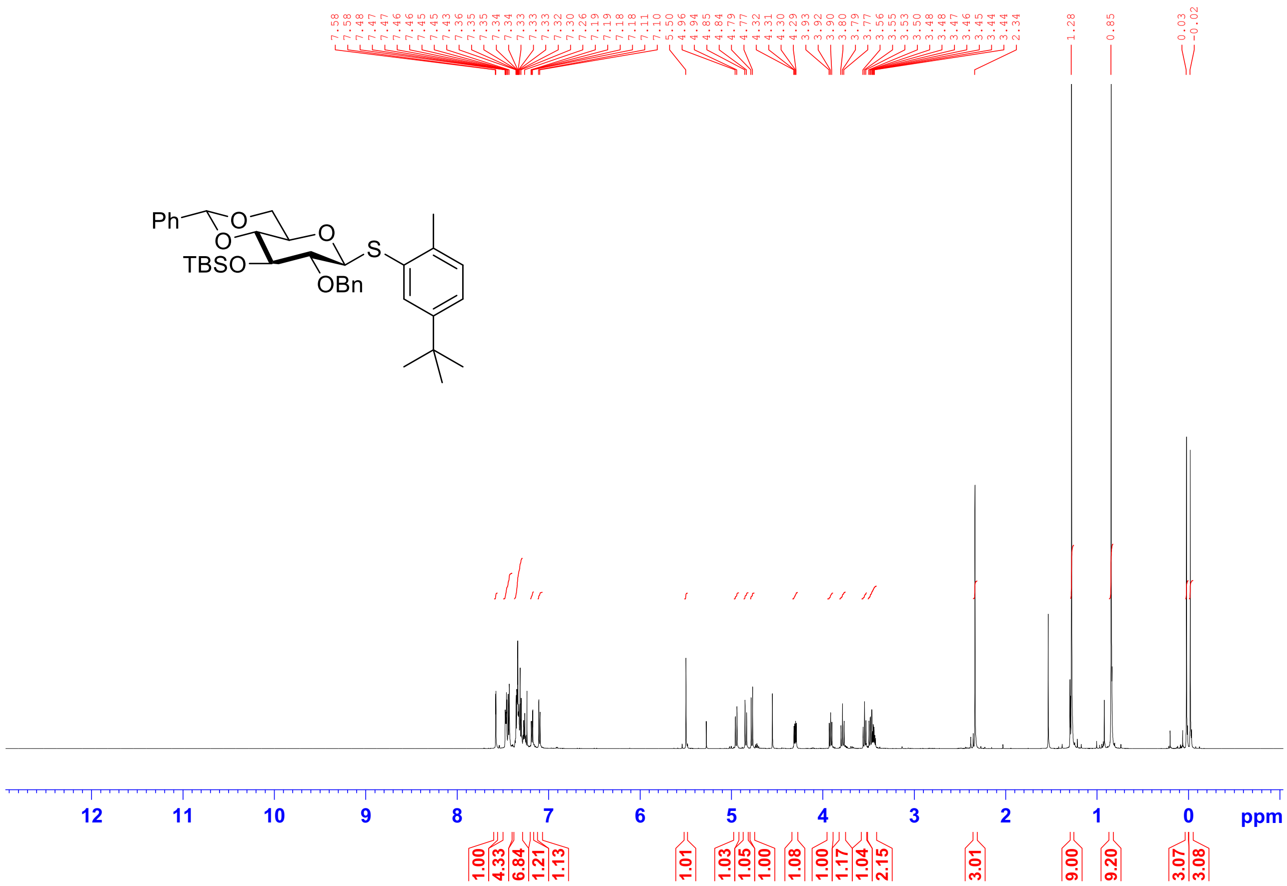

S4: ^{13}C NMR (150 MHz, $CDCl_3$)

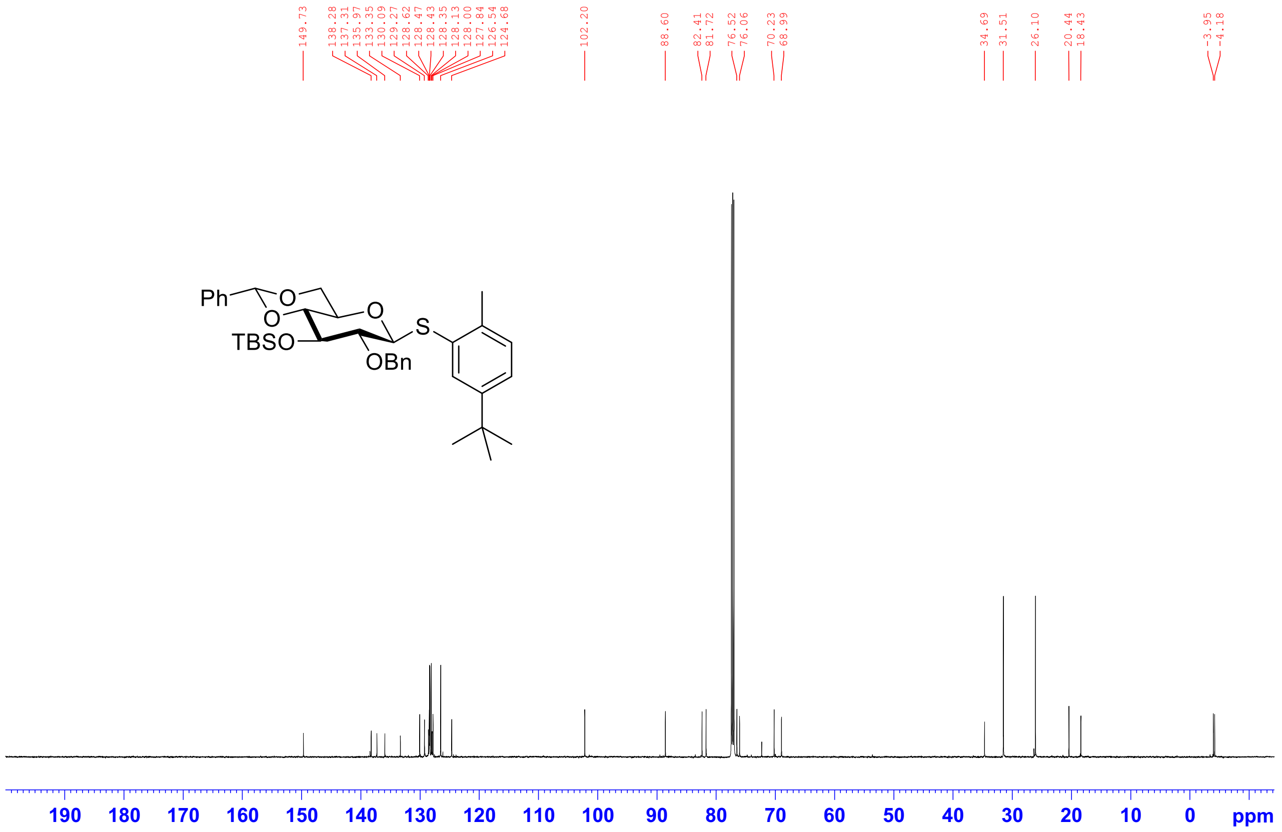

S4: DEPT 135 (150 MHz, $CDCl_3$)

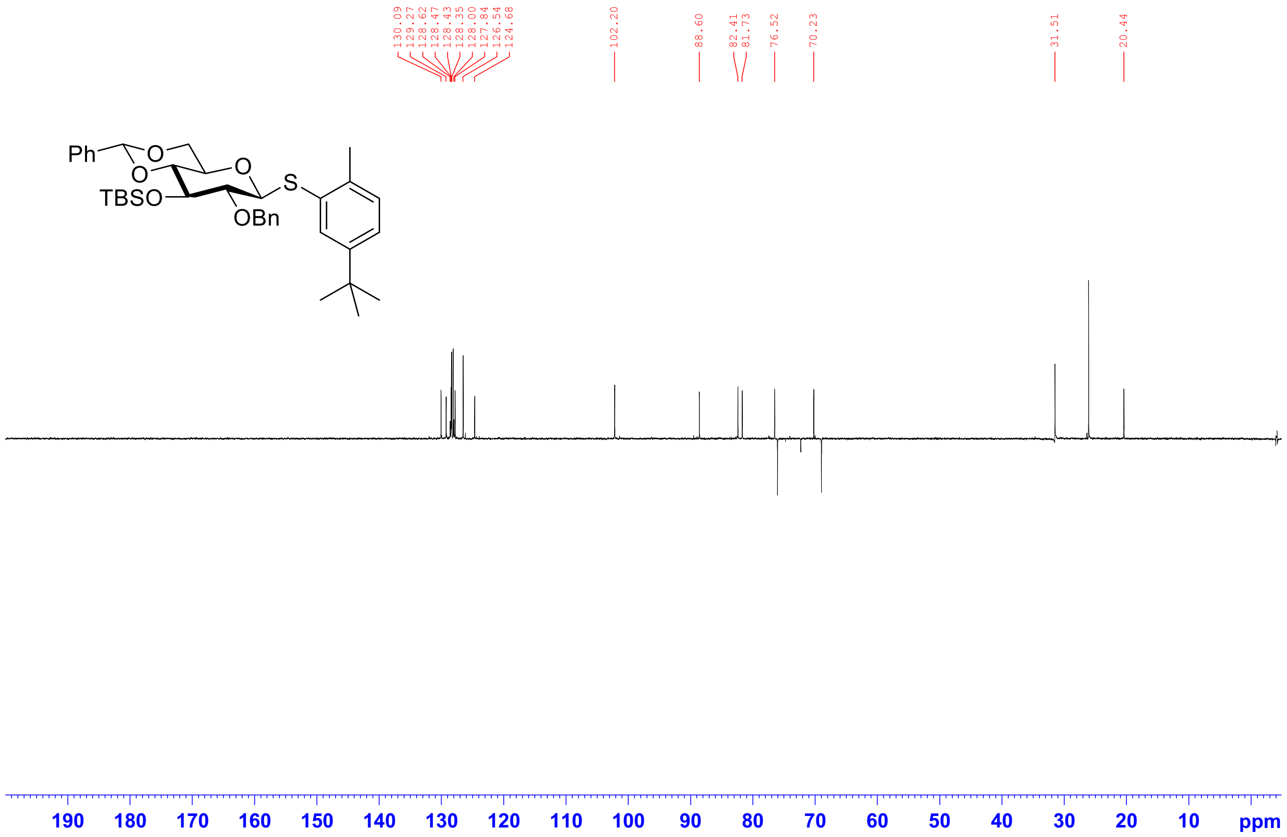

S5: ^{1}H NMR (600 MHz, $CDCl_3$)

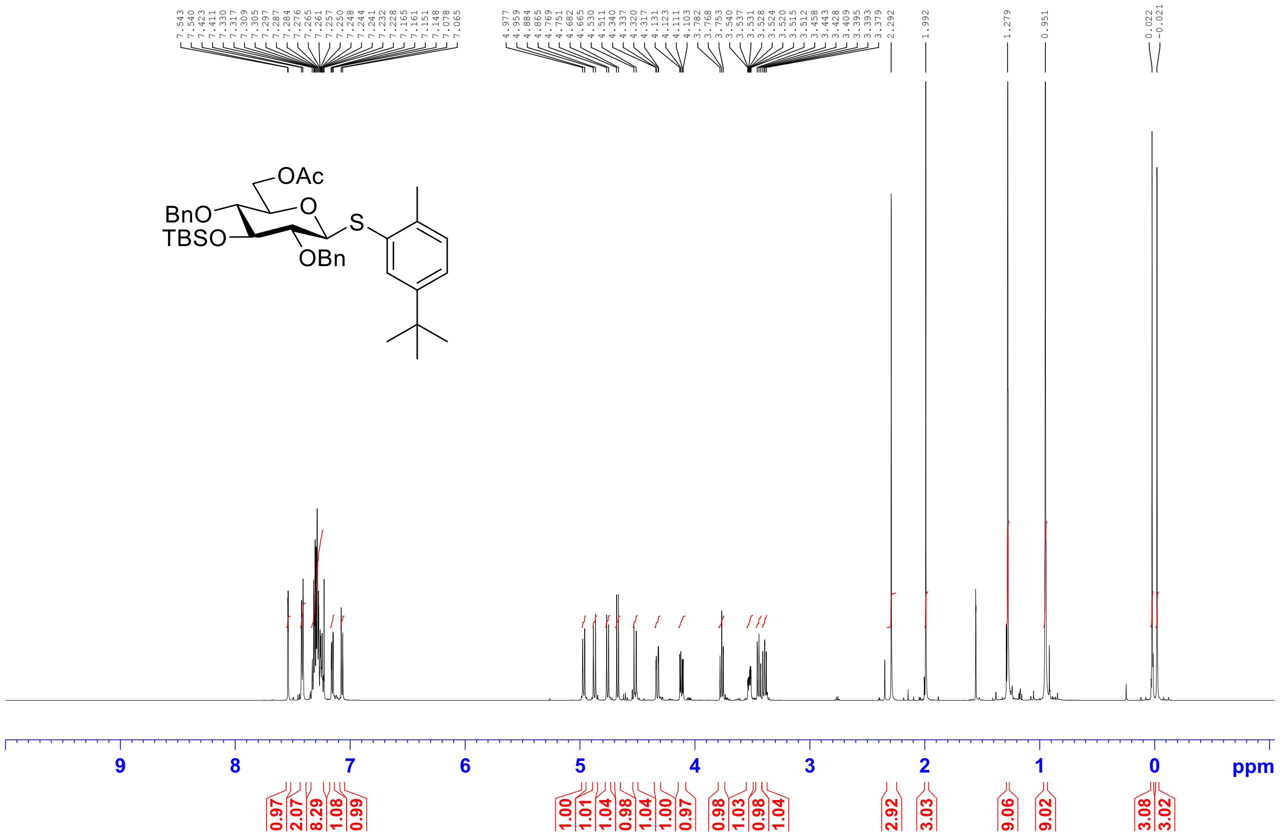

S5: ^{13}C NMR (150 MHz, $CDCl_3$)

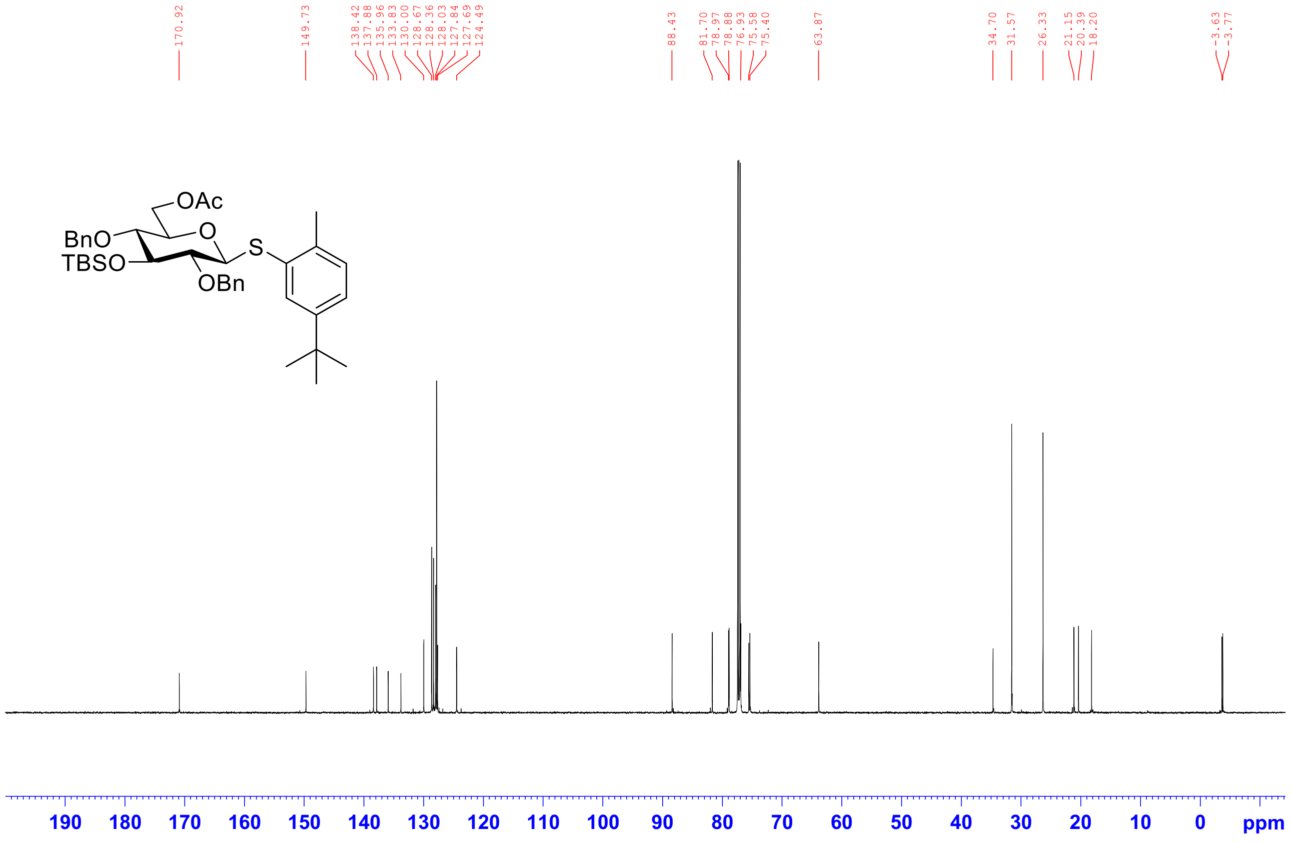

S5: DEPT 135 (150 MHz, CDCl₃)

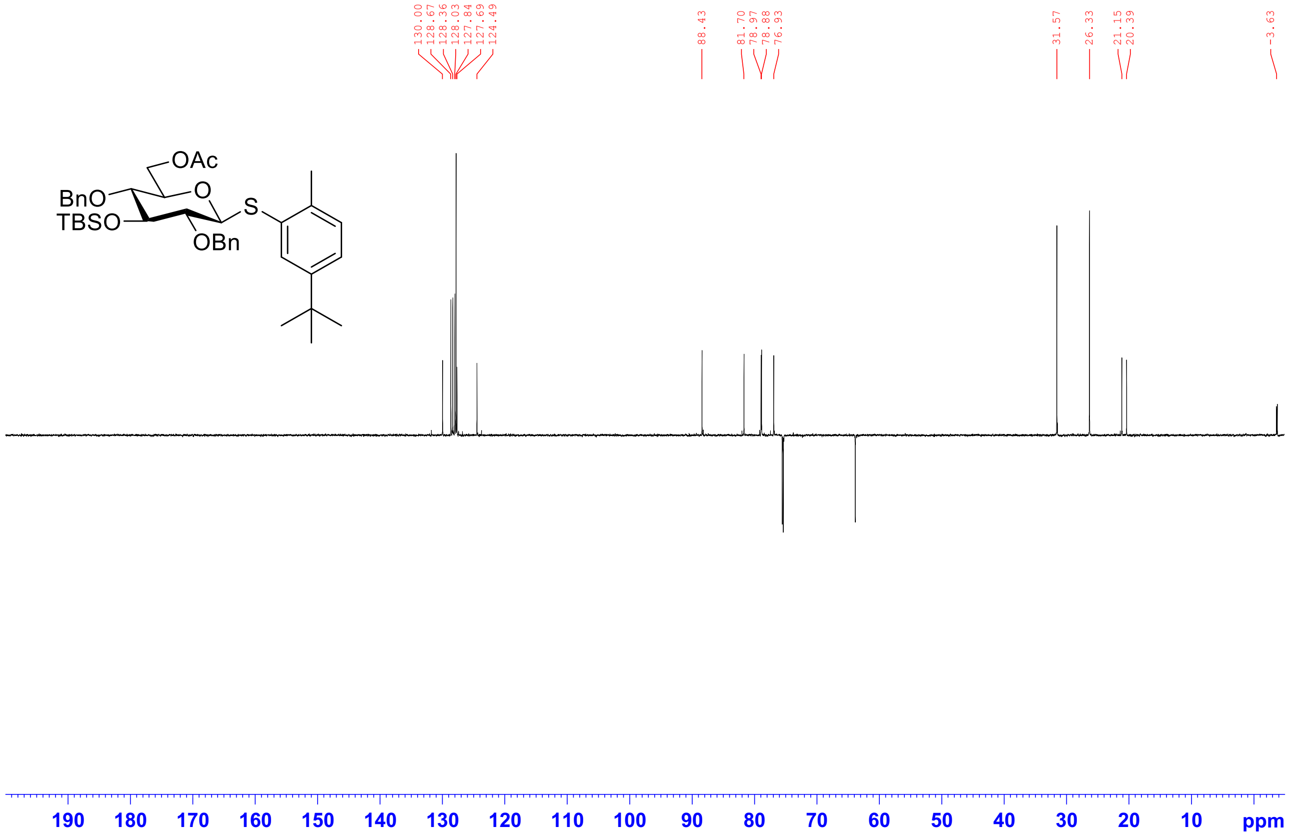

15: ^{1}H NMR (600 MHz, CDCl₃)

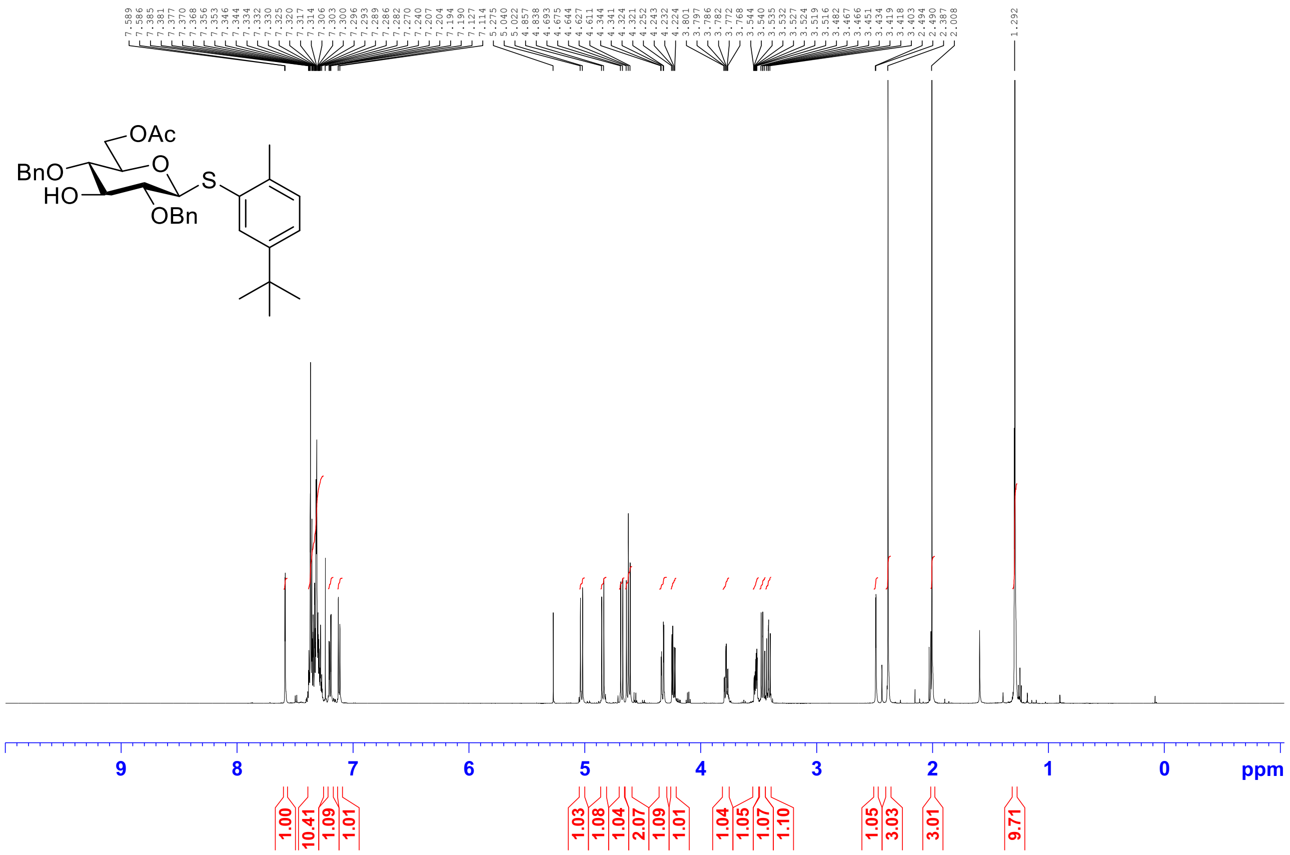

15: ^{13}C NMR (150 MHz, $CDCl_3$)

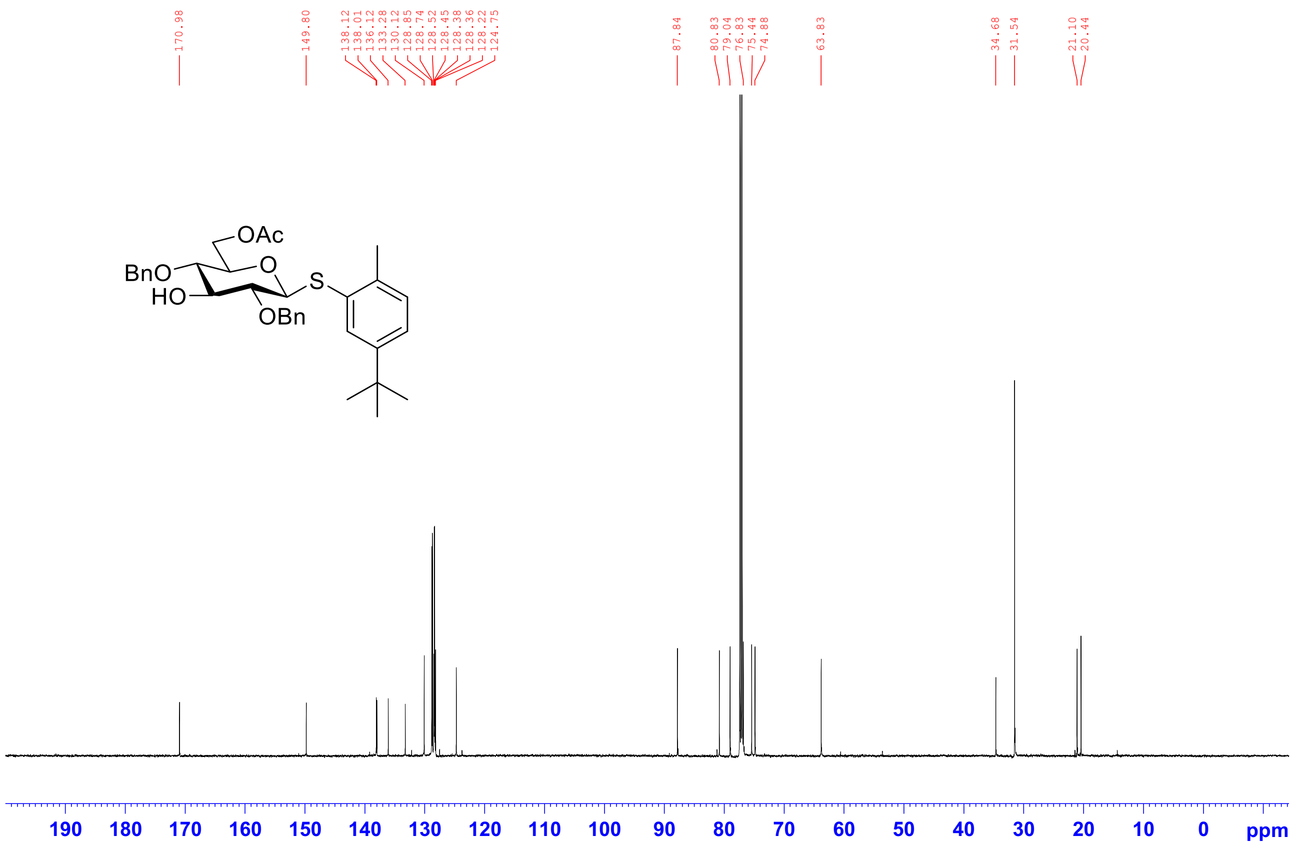

15: DEPT 135 (150 MHz, $CDCl_3$)

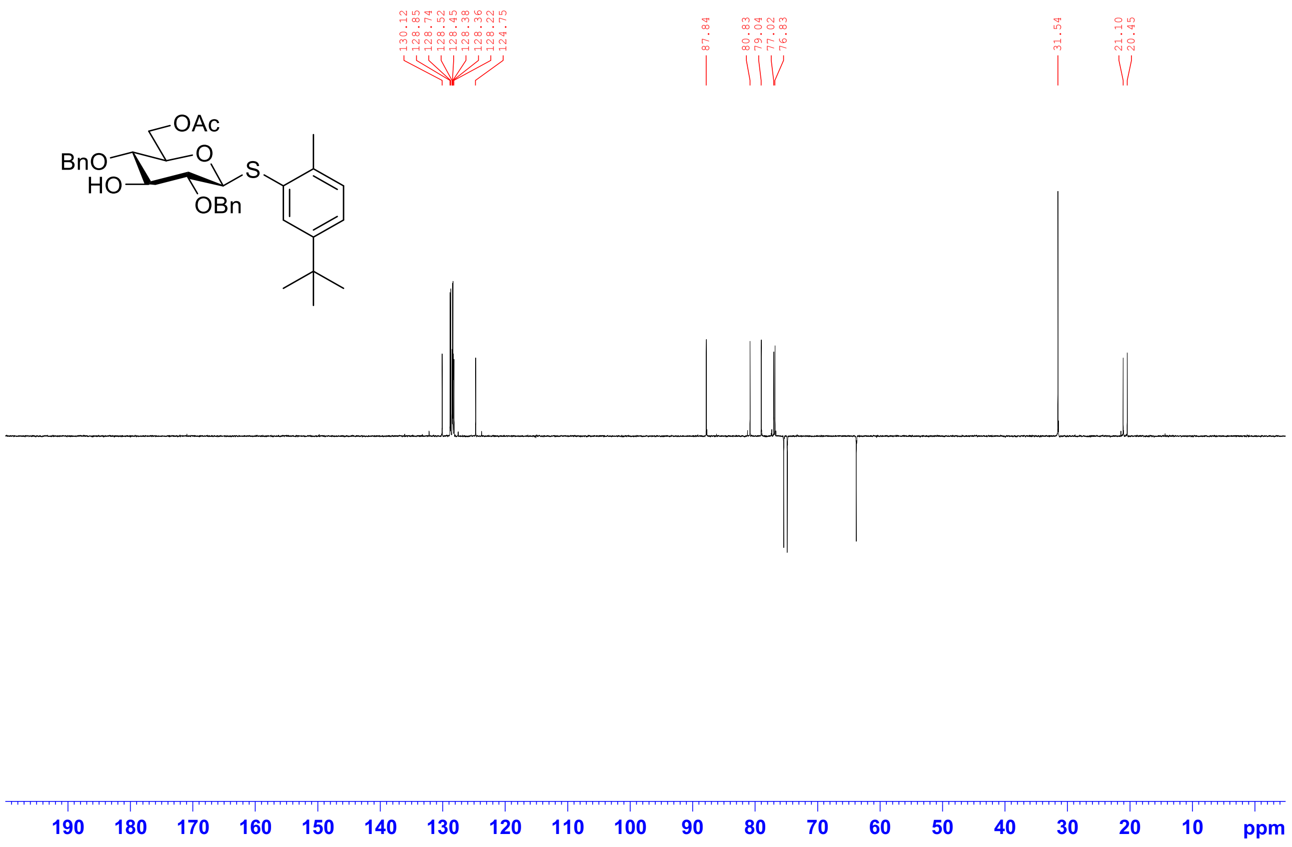

S6: ^{1}H NMR (600 MHz, $CDCl_3$)

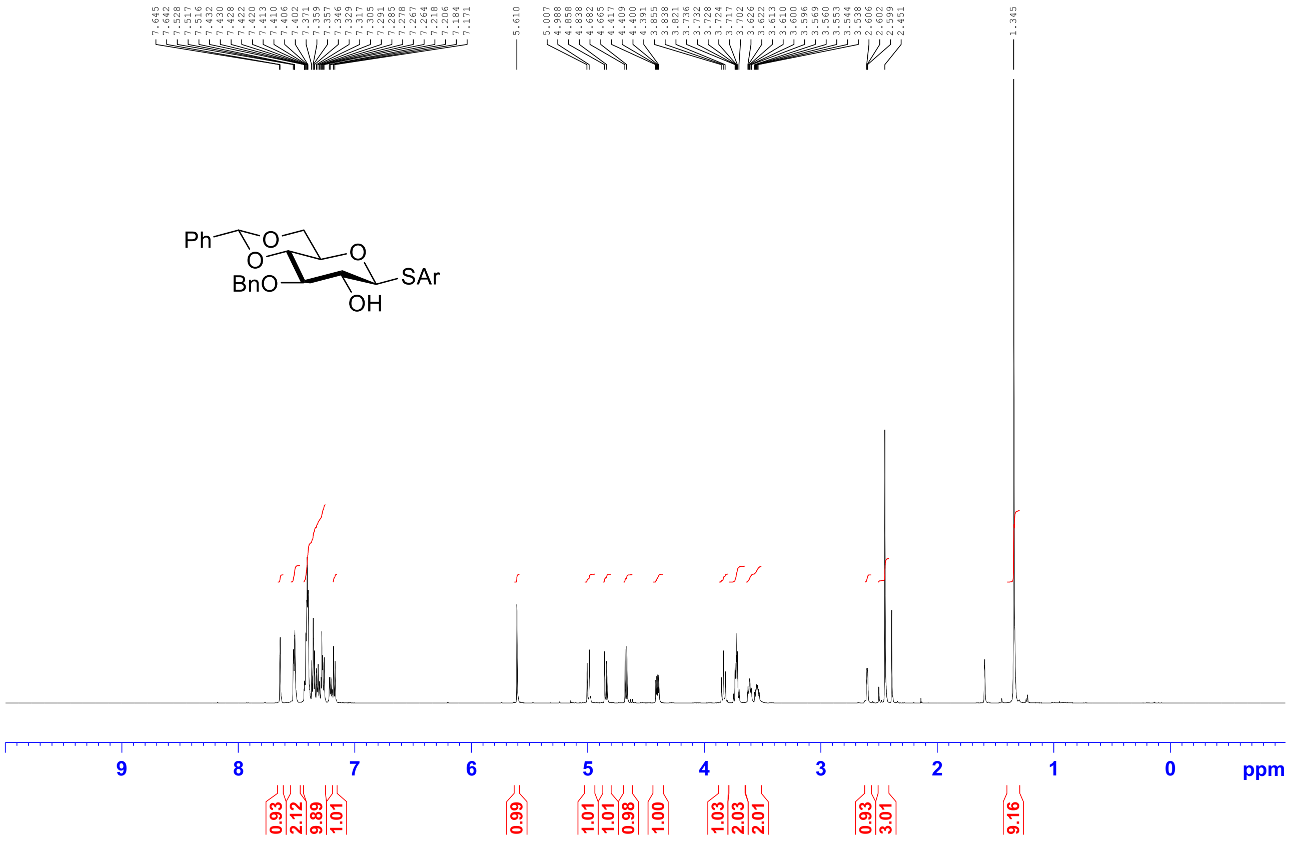

S6: ^{13}C NMR (150 MHz, $CDCl_3$)

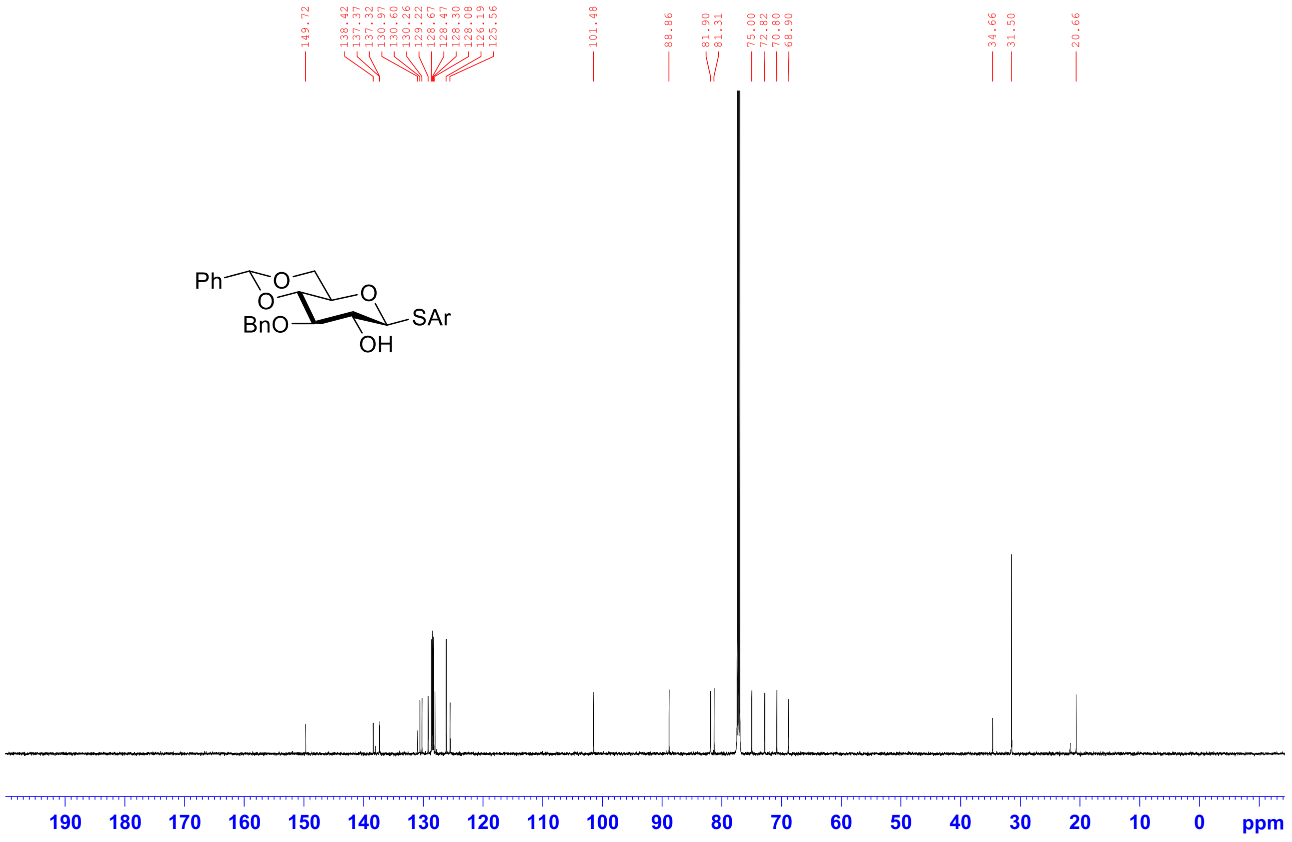

S6: DEPT 135 (150 MHz, $CDCl_3$)

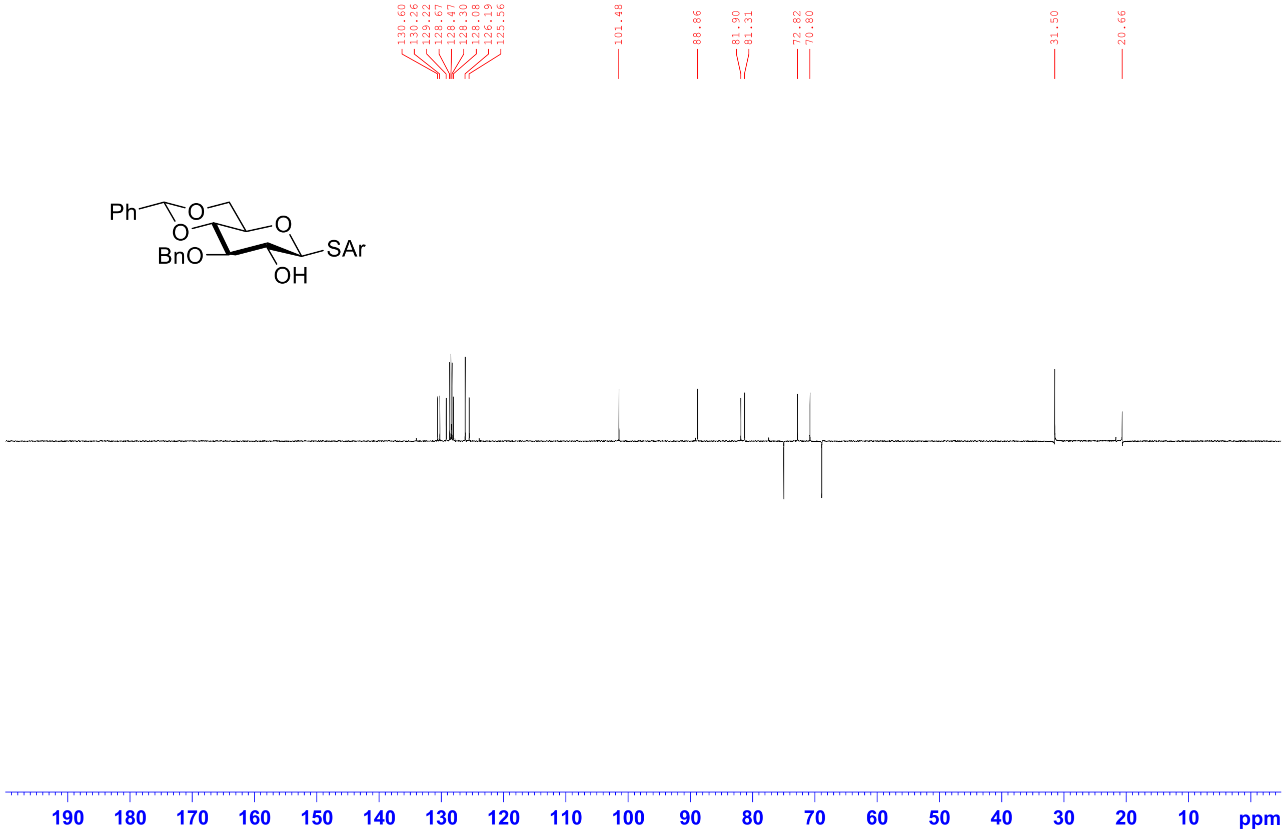

S7: ^{1}H NMR (600 MHz, $CDCl_3$)

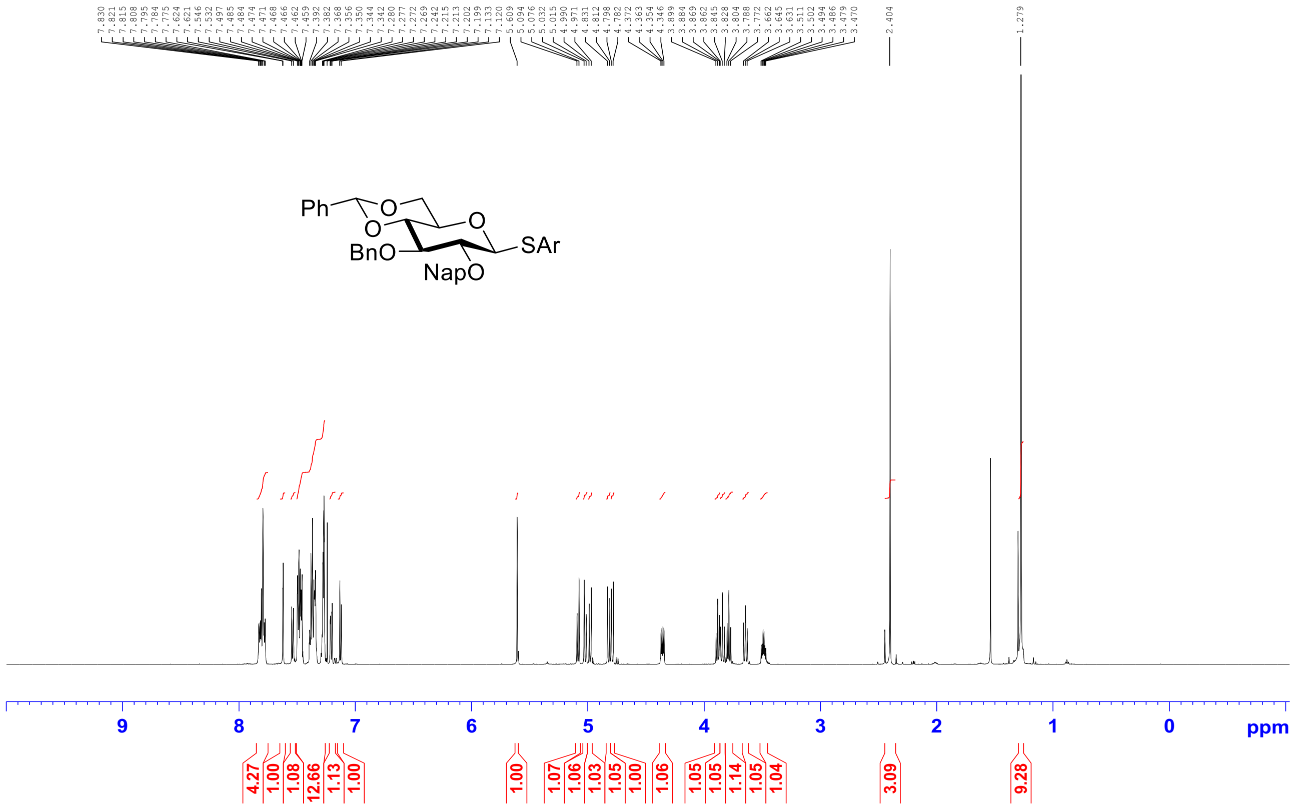

S7: ^{13}C NMR (150 MHz, $CDCl_3$)

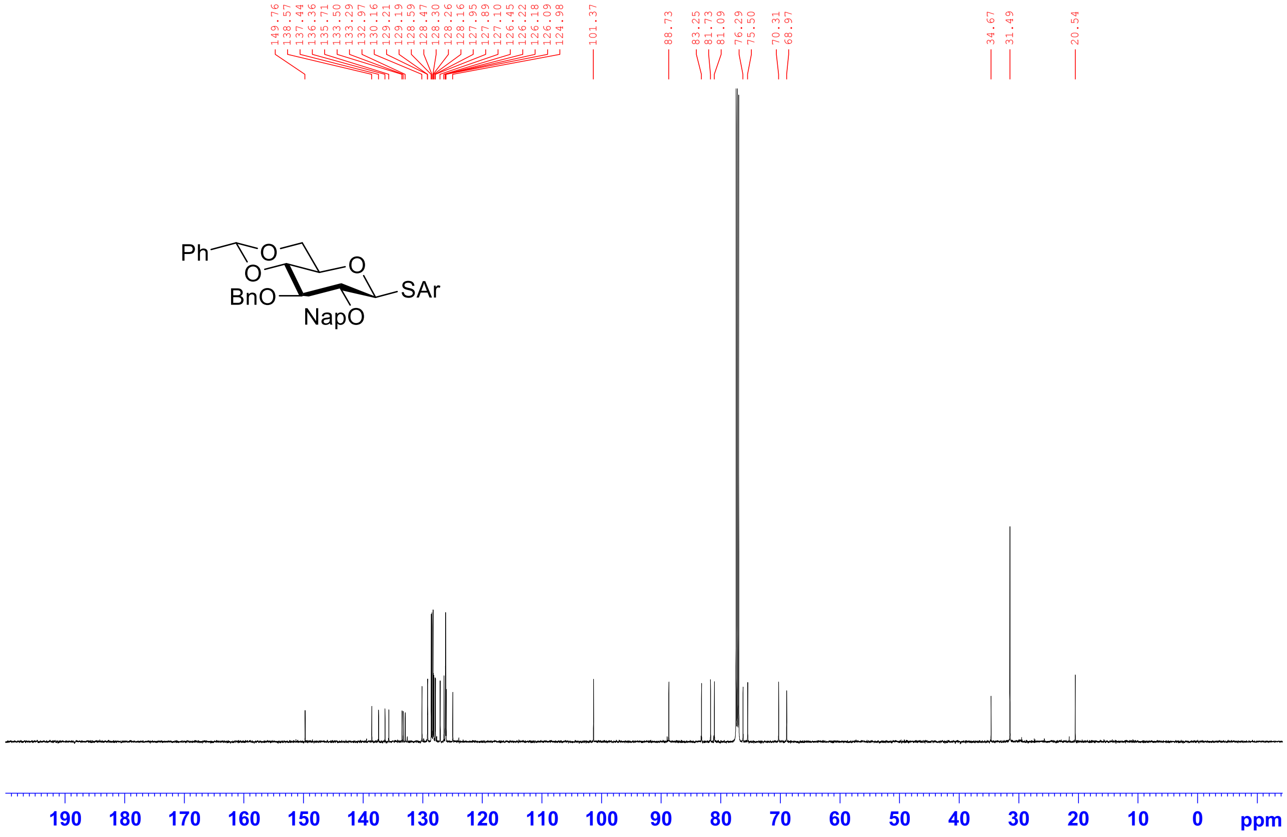

S7: DEPT 135 (150 MHz, $CDCl_3$)

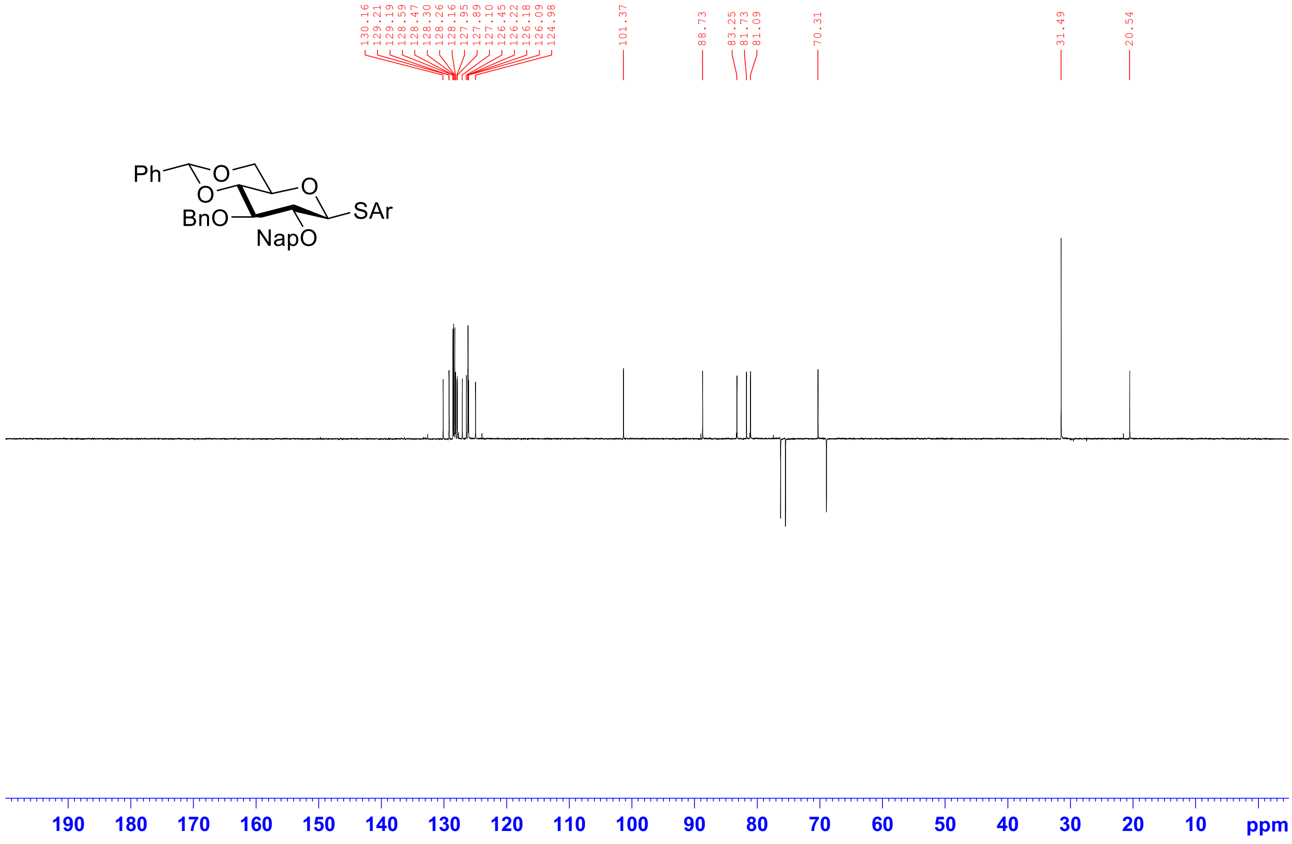

S8: ^{1}H NMR (600 MHz, $CDCl_3$)

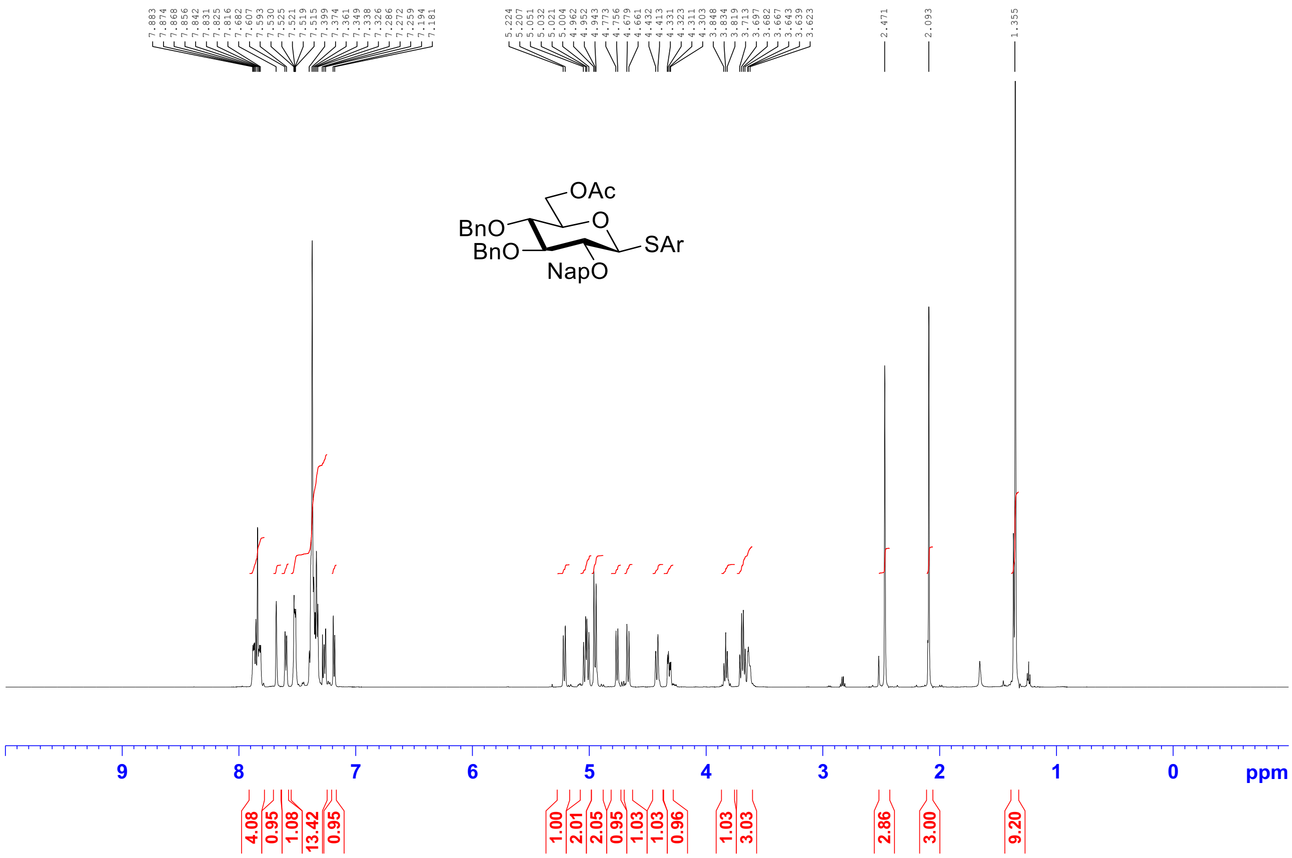

S8: ^{13}C NMR (150 MHz, $CDCl_3$)

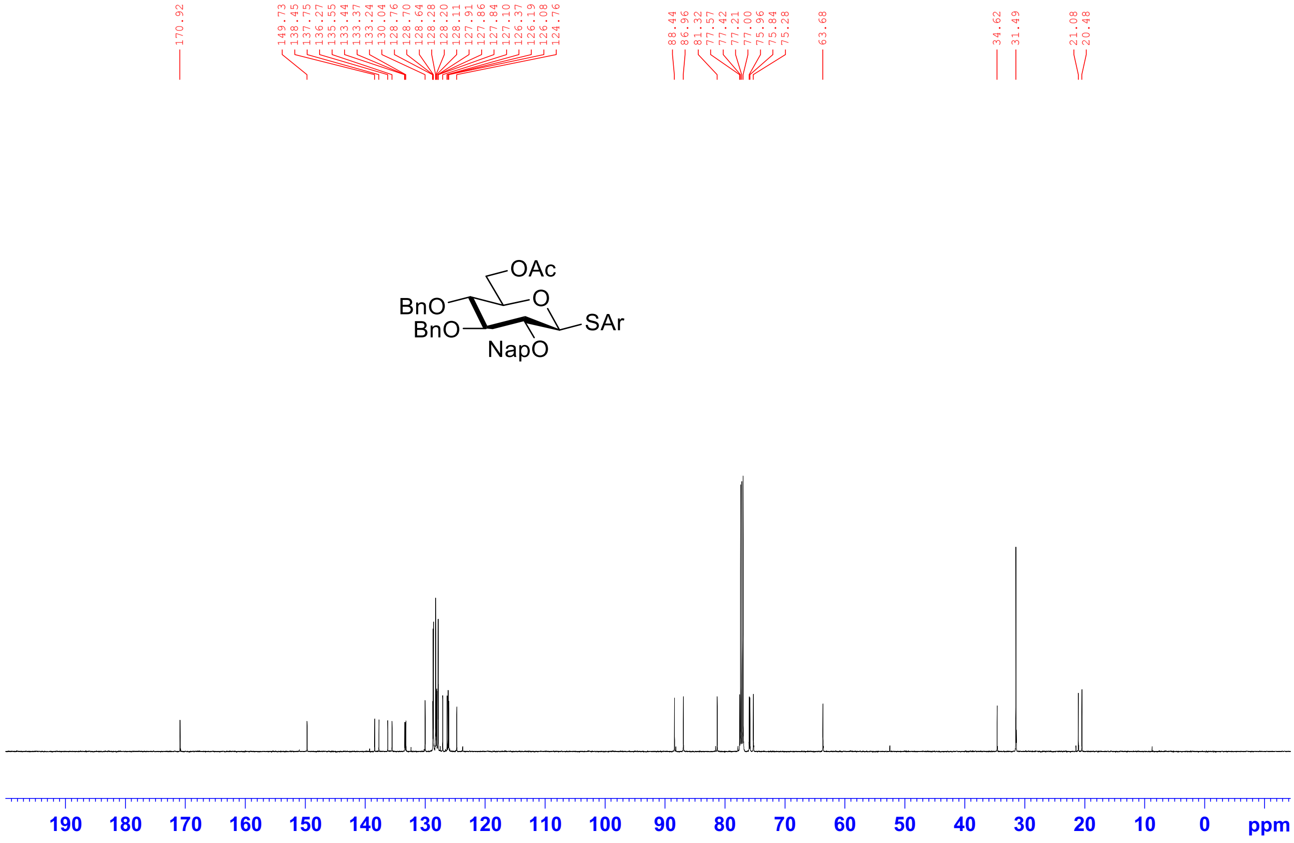

S8: DEPT 135 (150 MHz, $CDCl_3$)

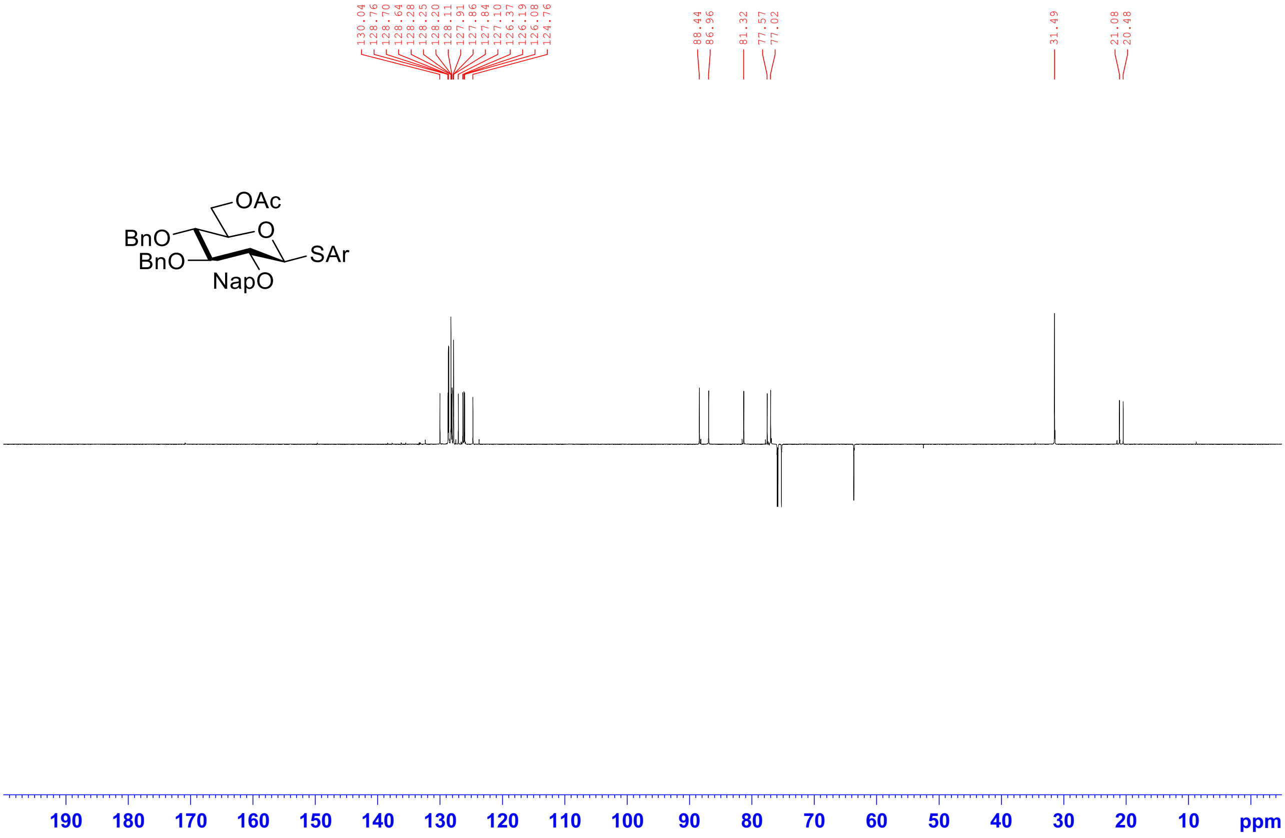

14: ^{1}H NMR (600 MHz, $CDCl_3$)

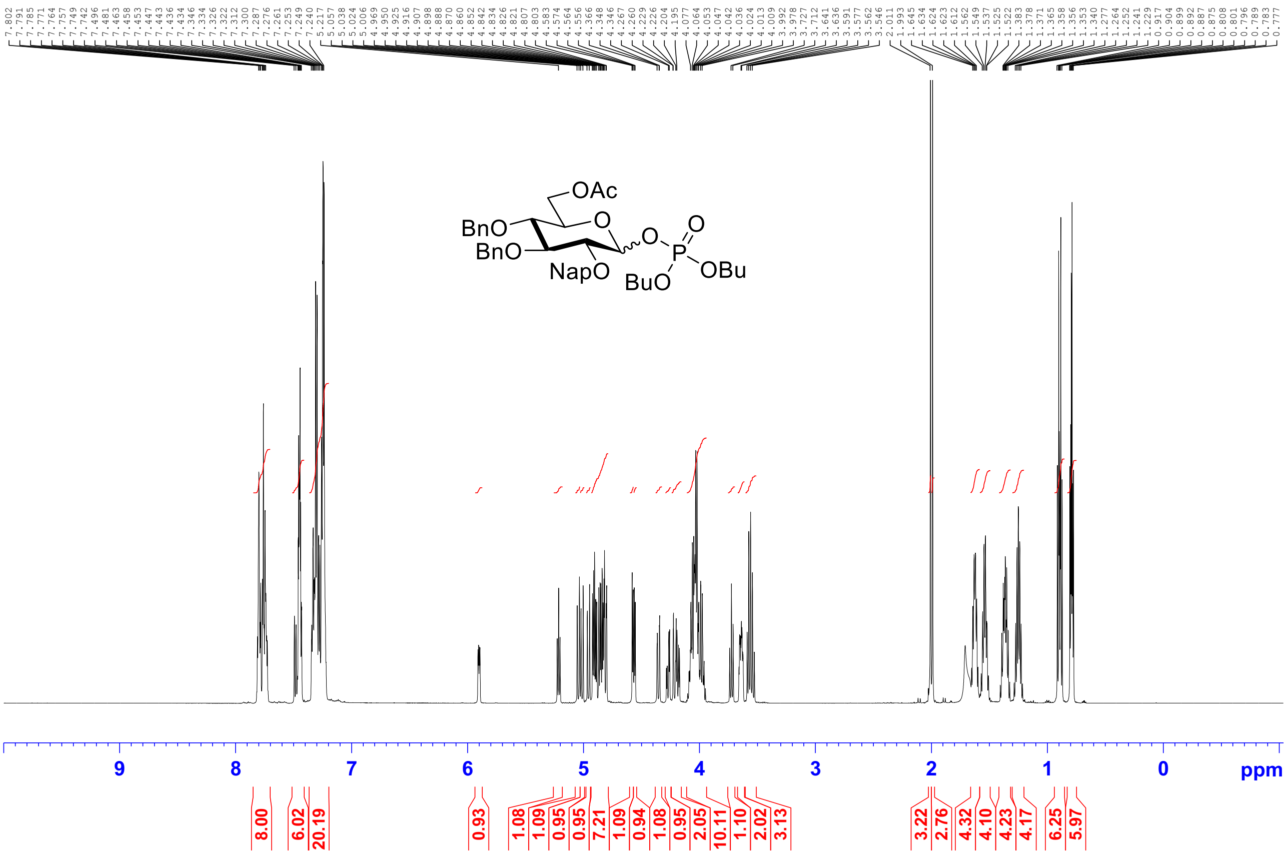

14: ^{13}C NMR (150 MHz, $CDCl_3$)

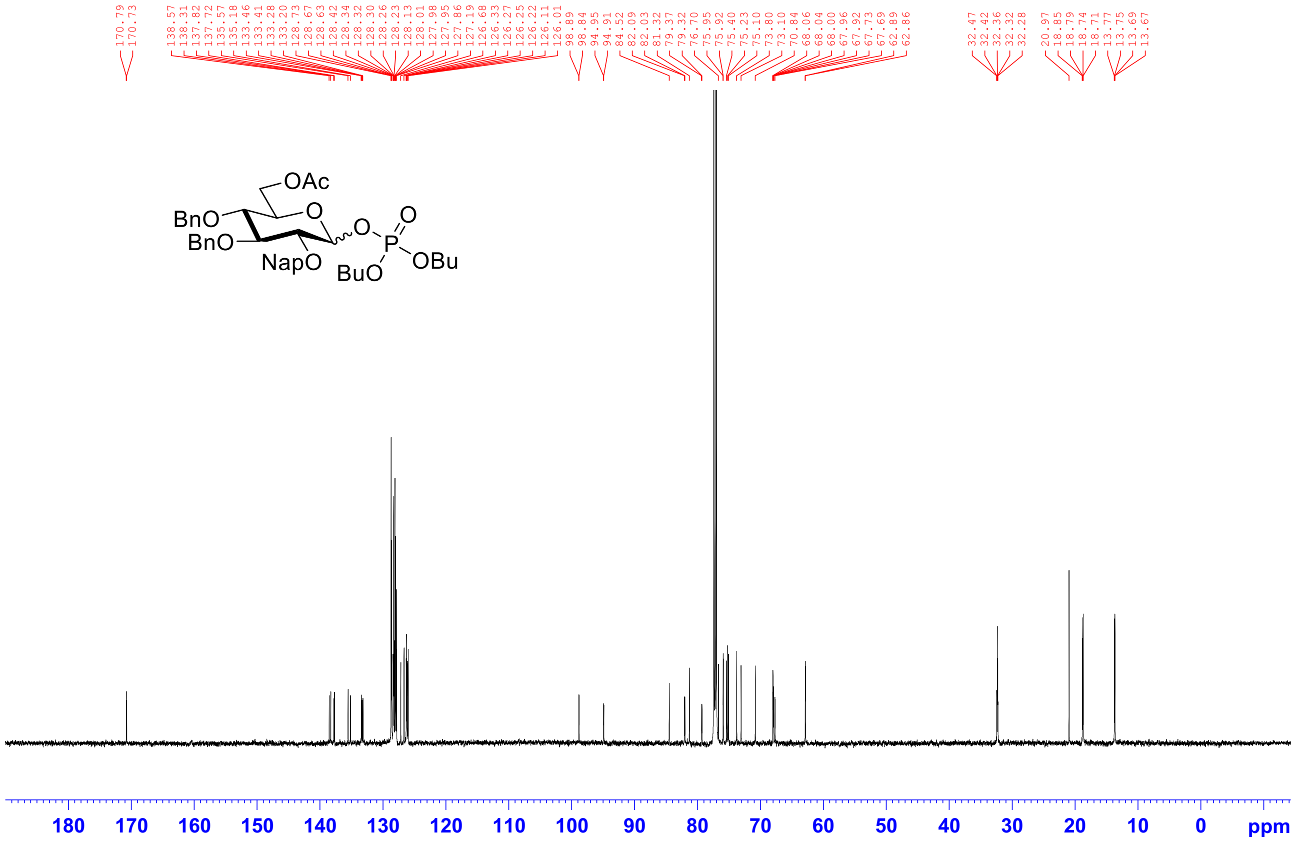

14: DEPT 135 (150 MHz, $CDCl_3$)

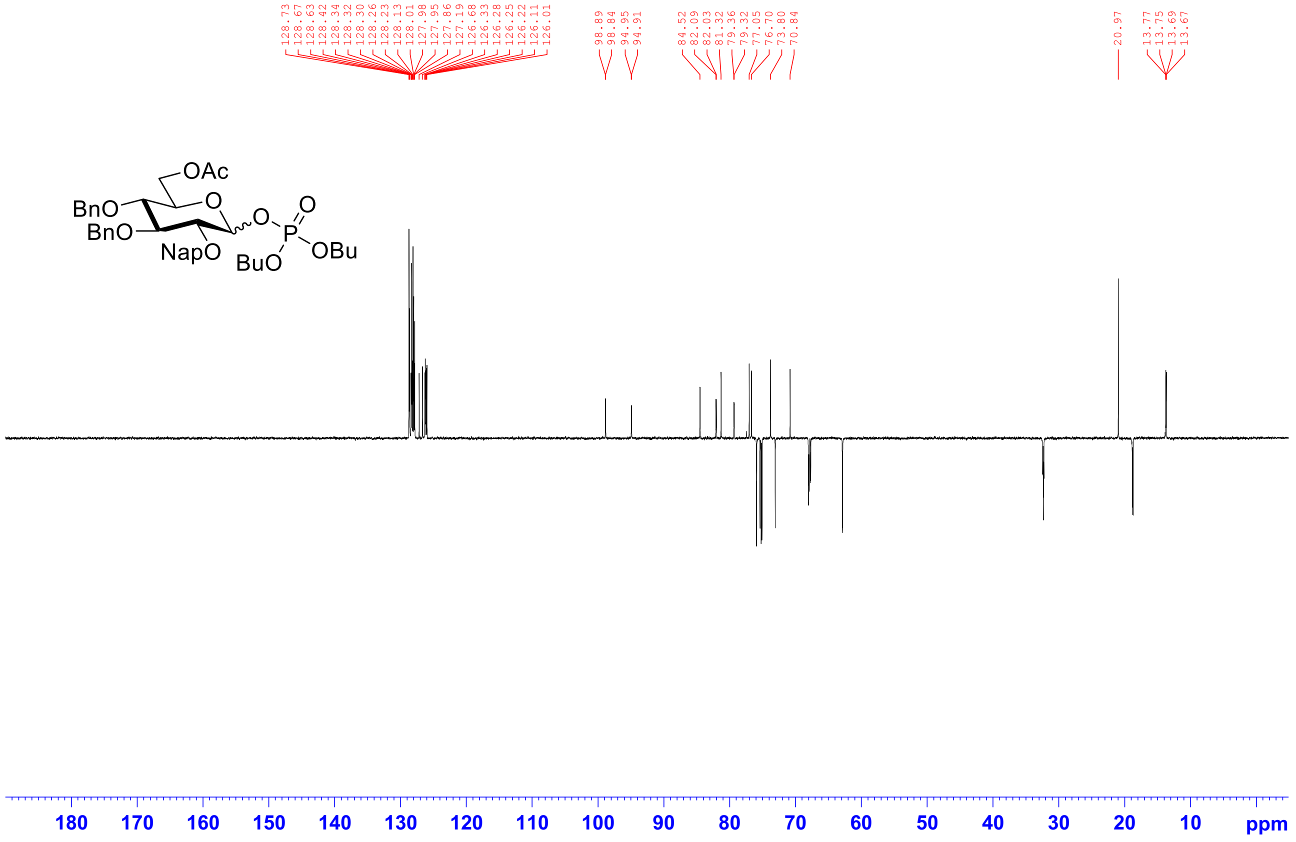

NMR spectra of rhamnose monosaccharides

S9: ^{1}H NMR (600 MHz, $CDCl_3$)

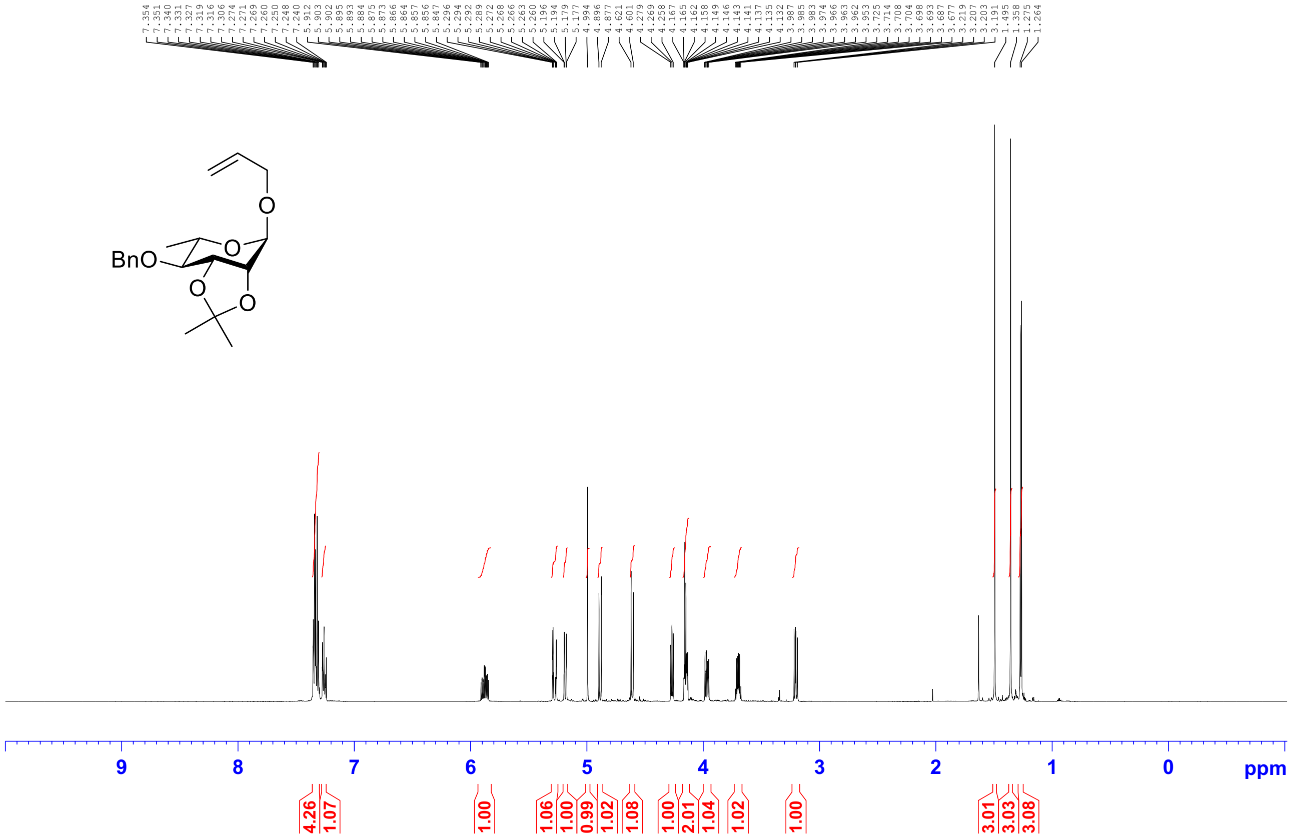

S9: ^{13}C NMR (150 MHz, $CDCl_3$)

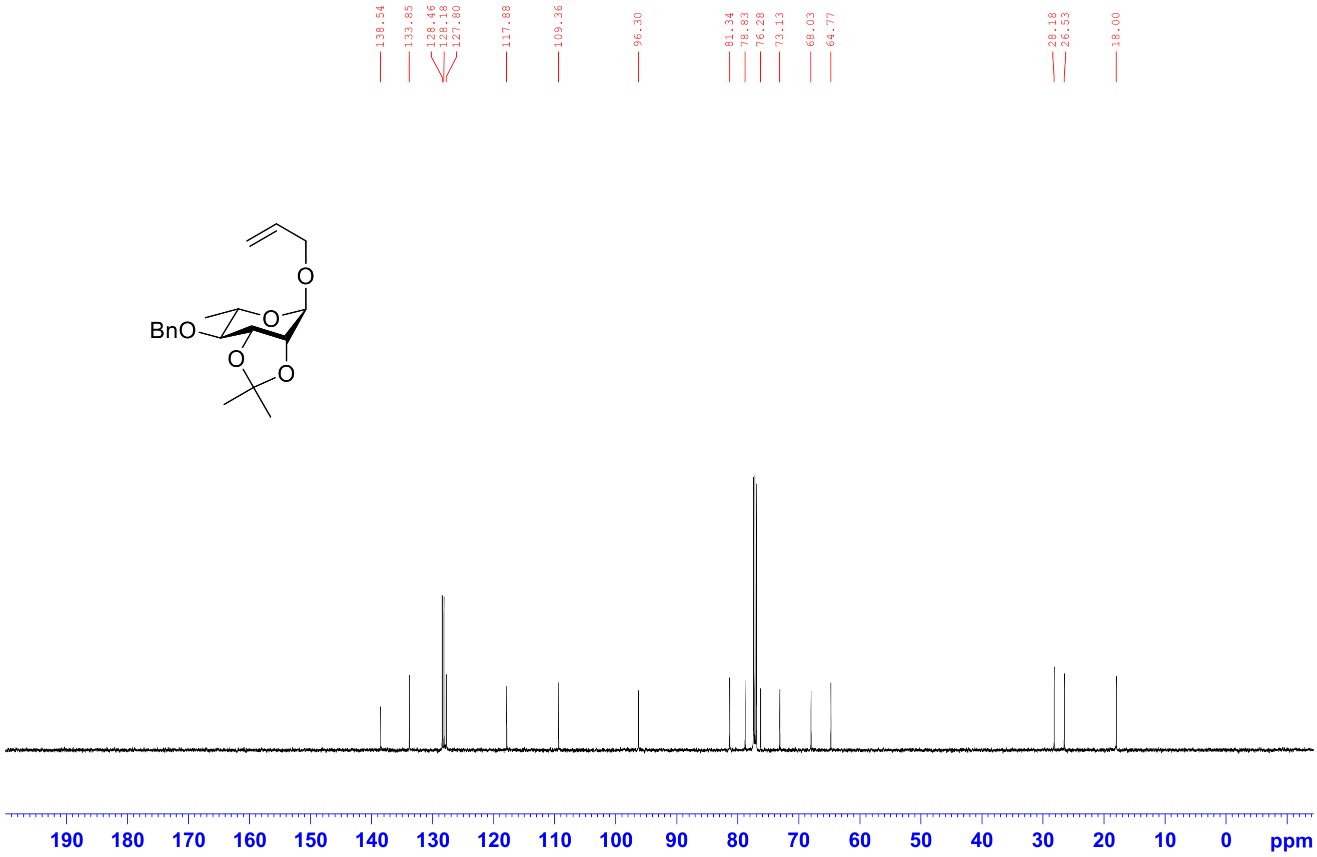

S9: DEPT 135 (150 MHz, CDCl$_3$)

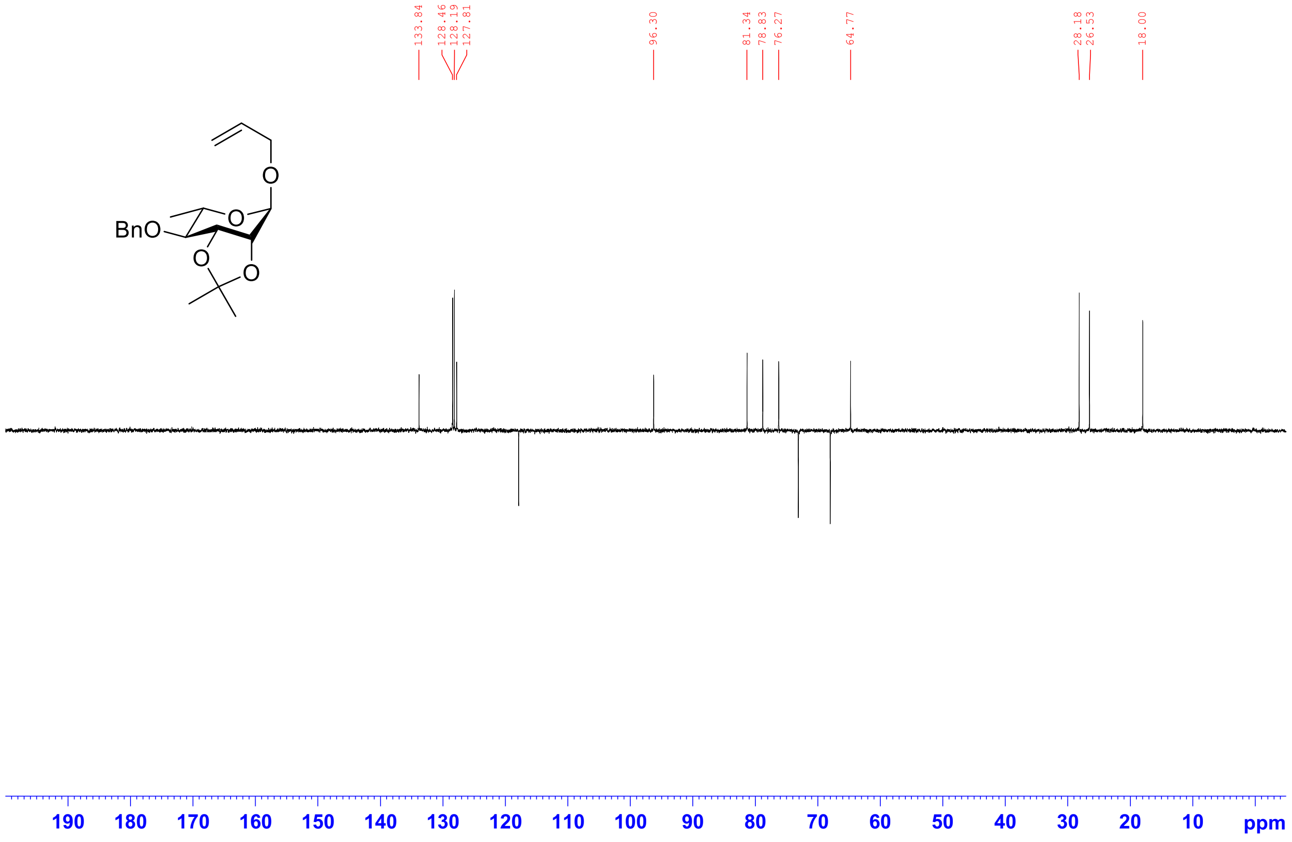

17: ^{1}H NMR (600 MHz, CDCl$_3$)

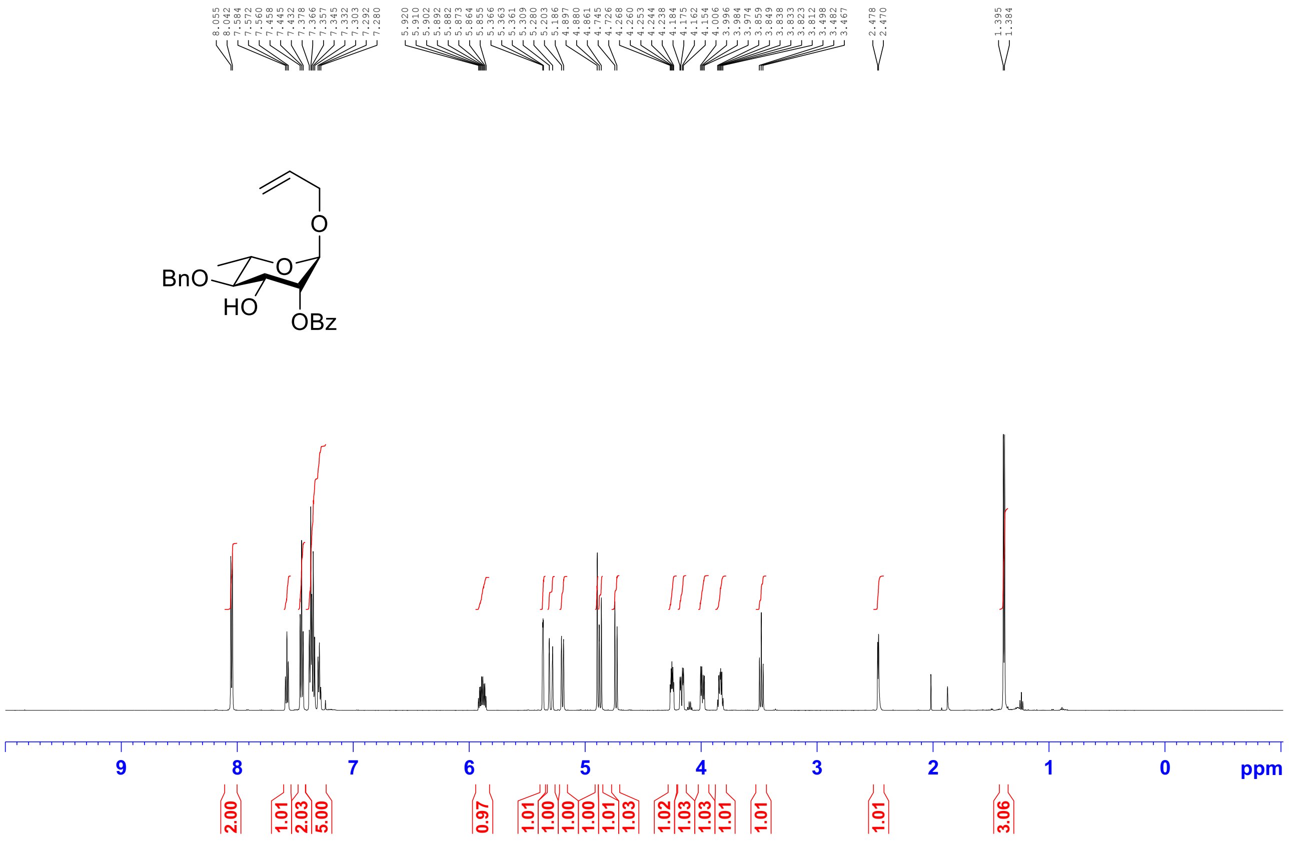

17: ^{13}C NMR (150 MHz, CDCl$_3$)

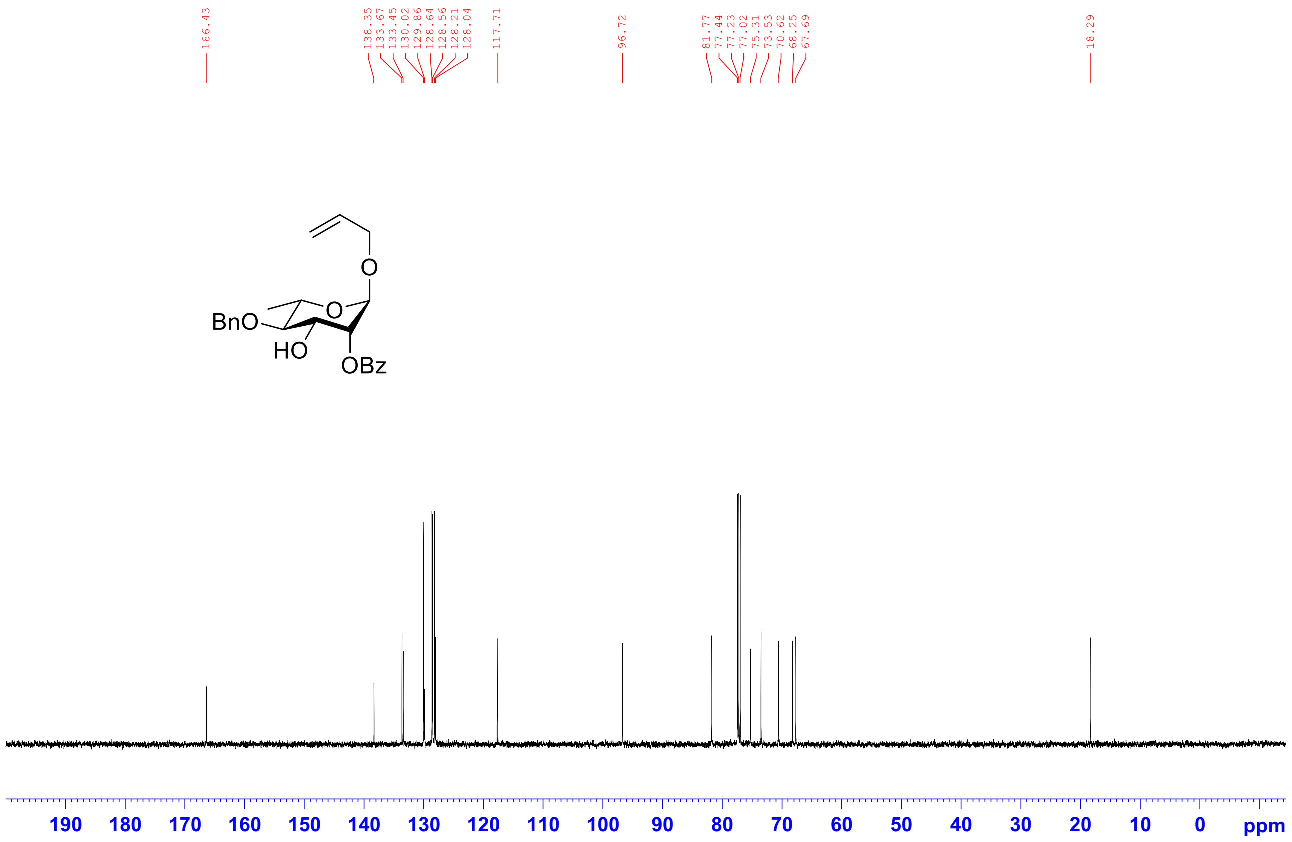

17: DEPT 135 (150 MHz, CDCl$_3$)

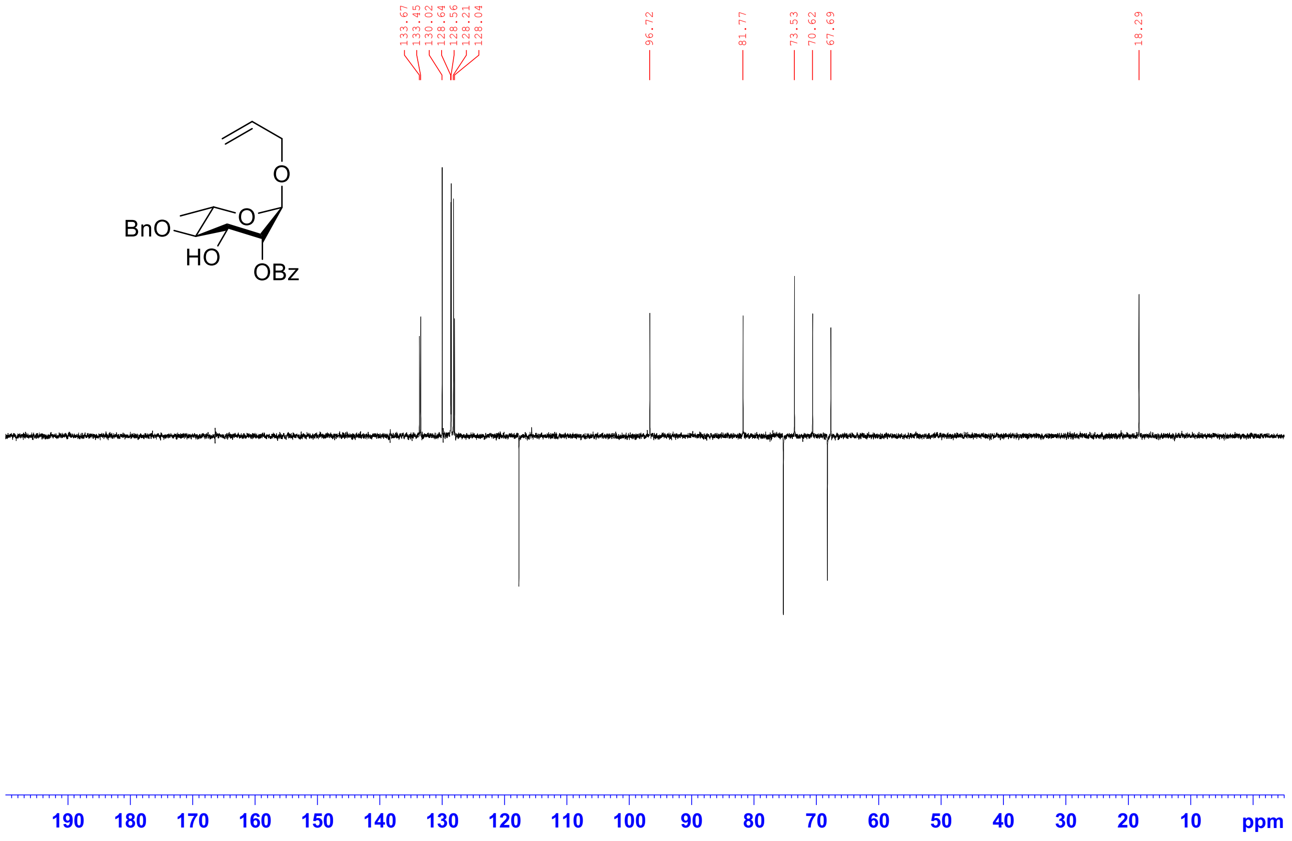

S10: ^{1}H NMR (600 MHz, CDCl$_3$)

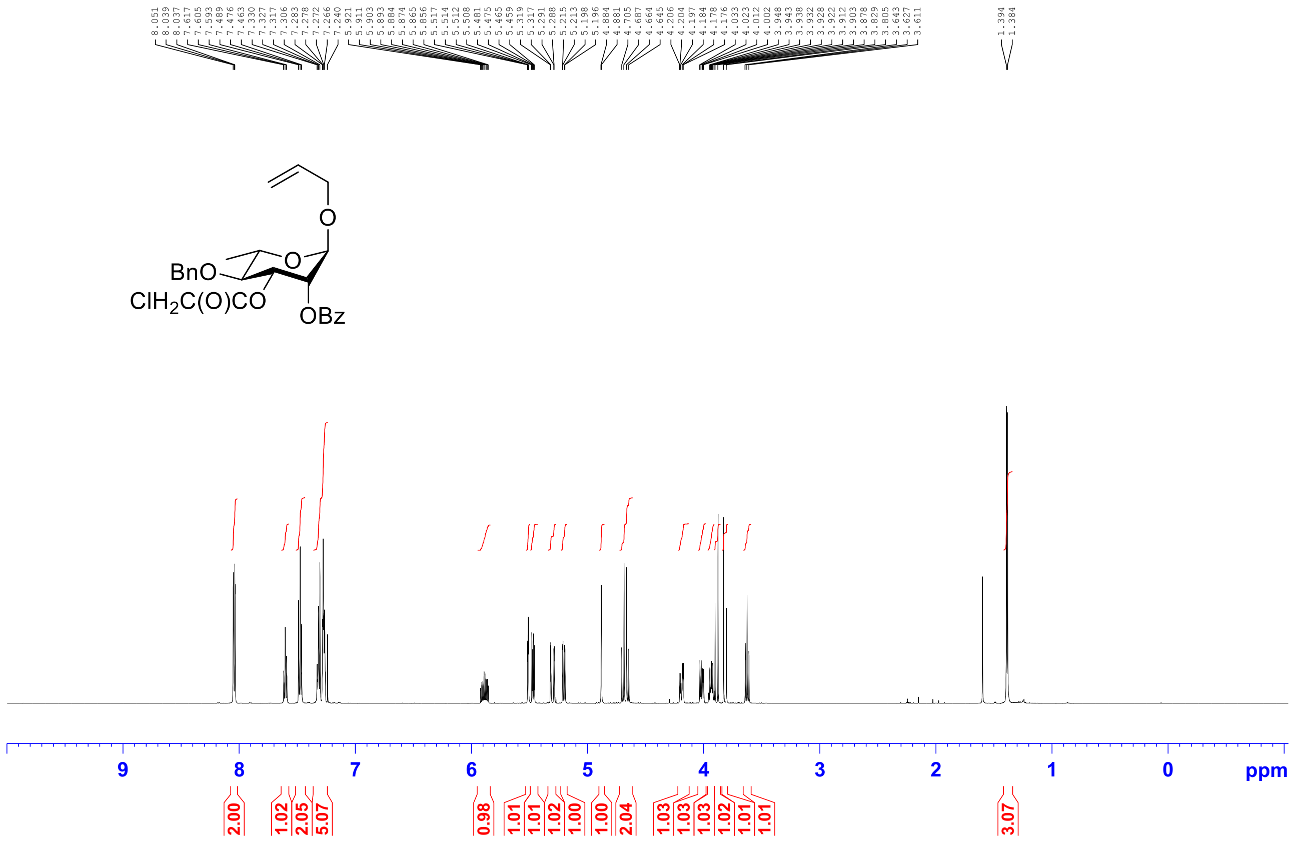

S10: ^{13}C NMR (150 MHz, CDCl$_3$)

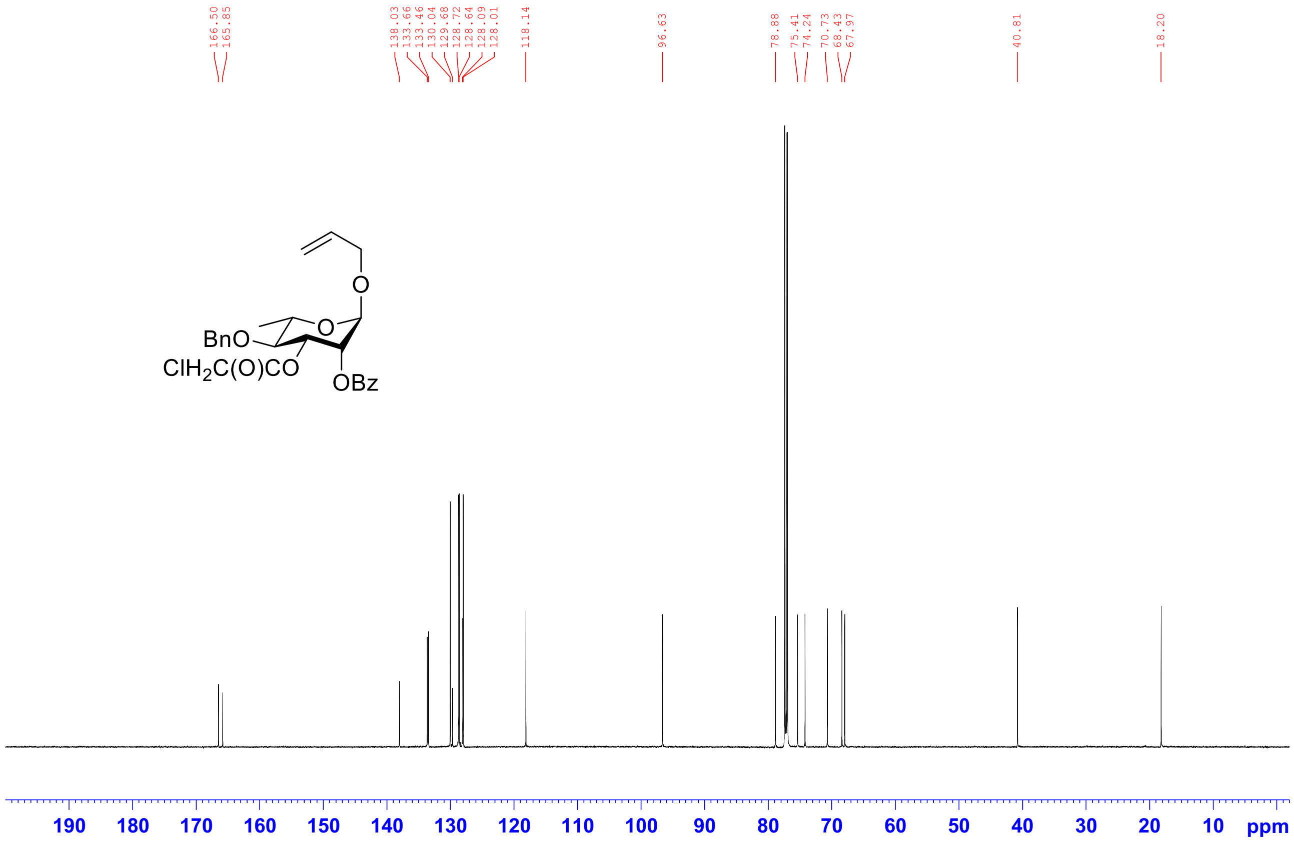

S10: DEPT 135 (150 MHz, $CDCl_3$)

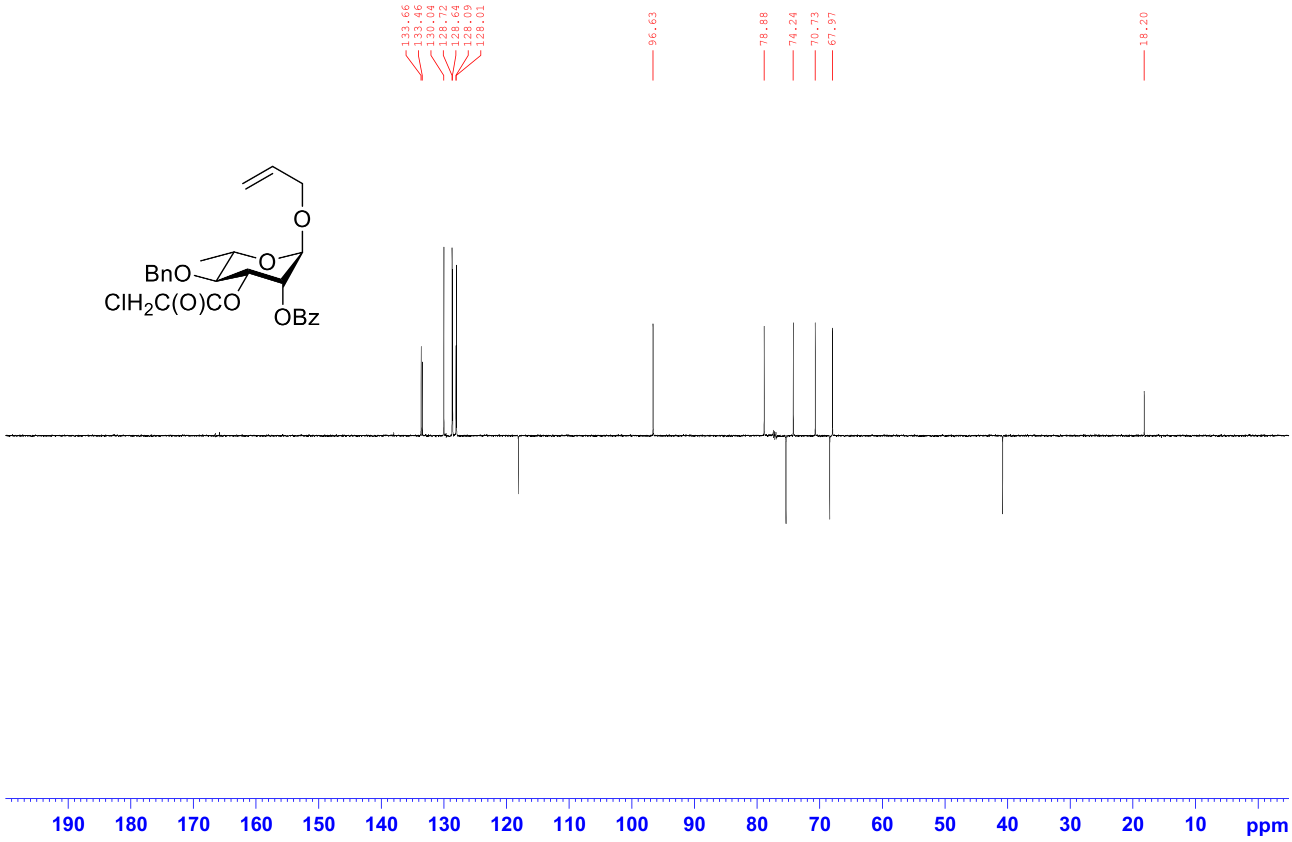

33: ^{1}H NMR (600 MHz, $CDCl_3$)

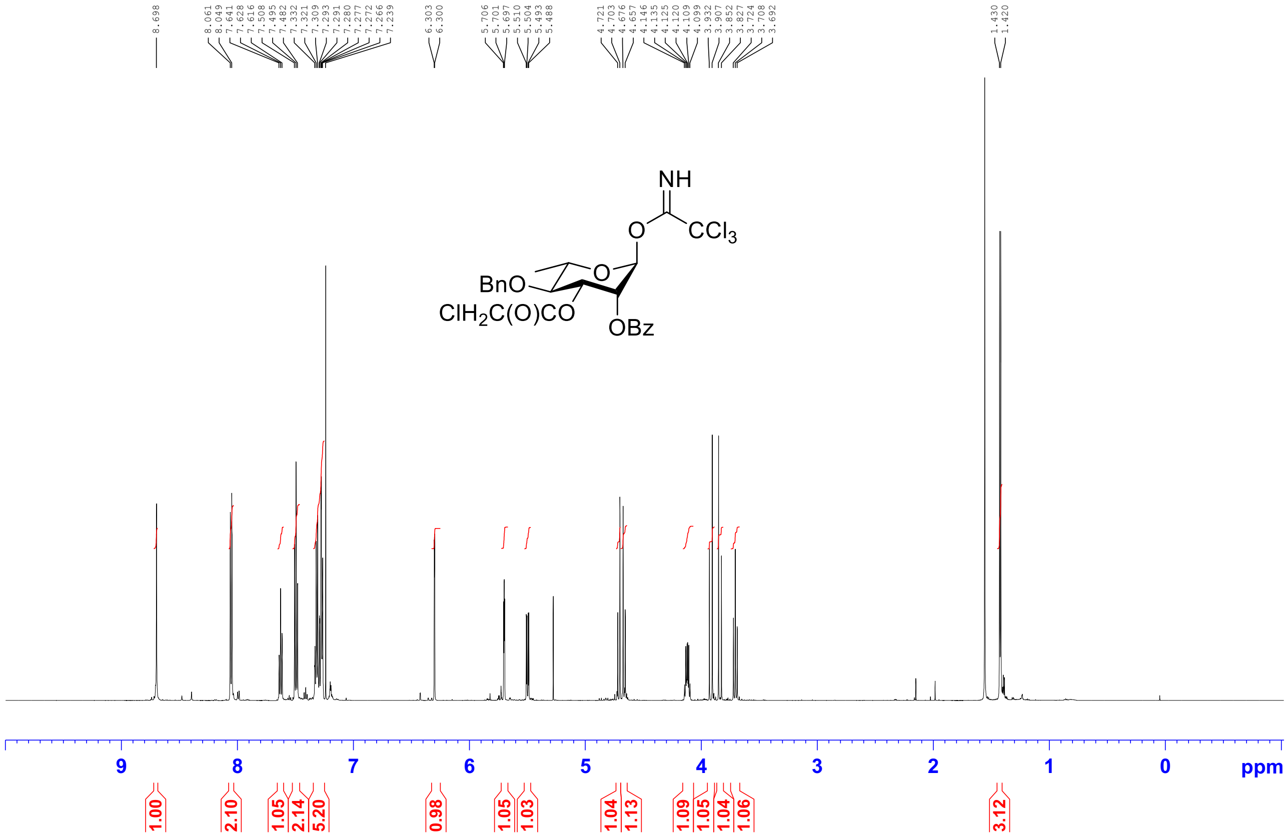

33: ^{13}C NMR (150 MHz, CDCl$_3$)

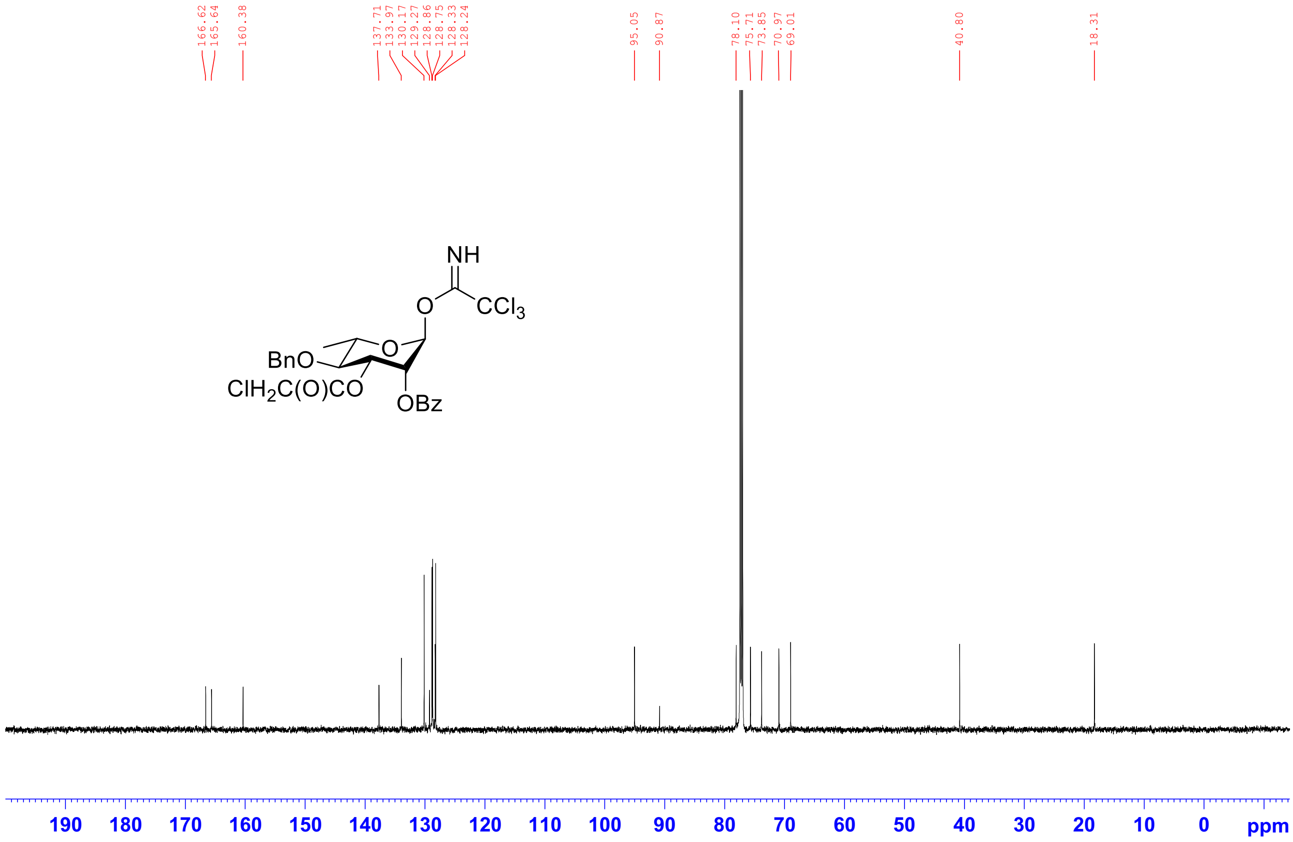

33: DEPT 135 (150 MHz, CDCl$_3$)

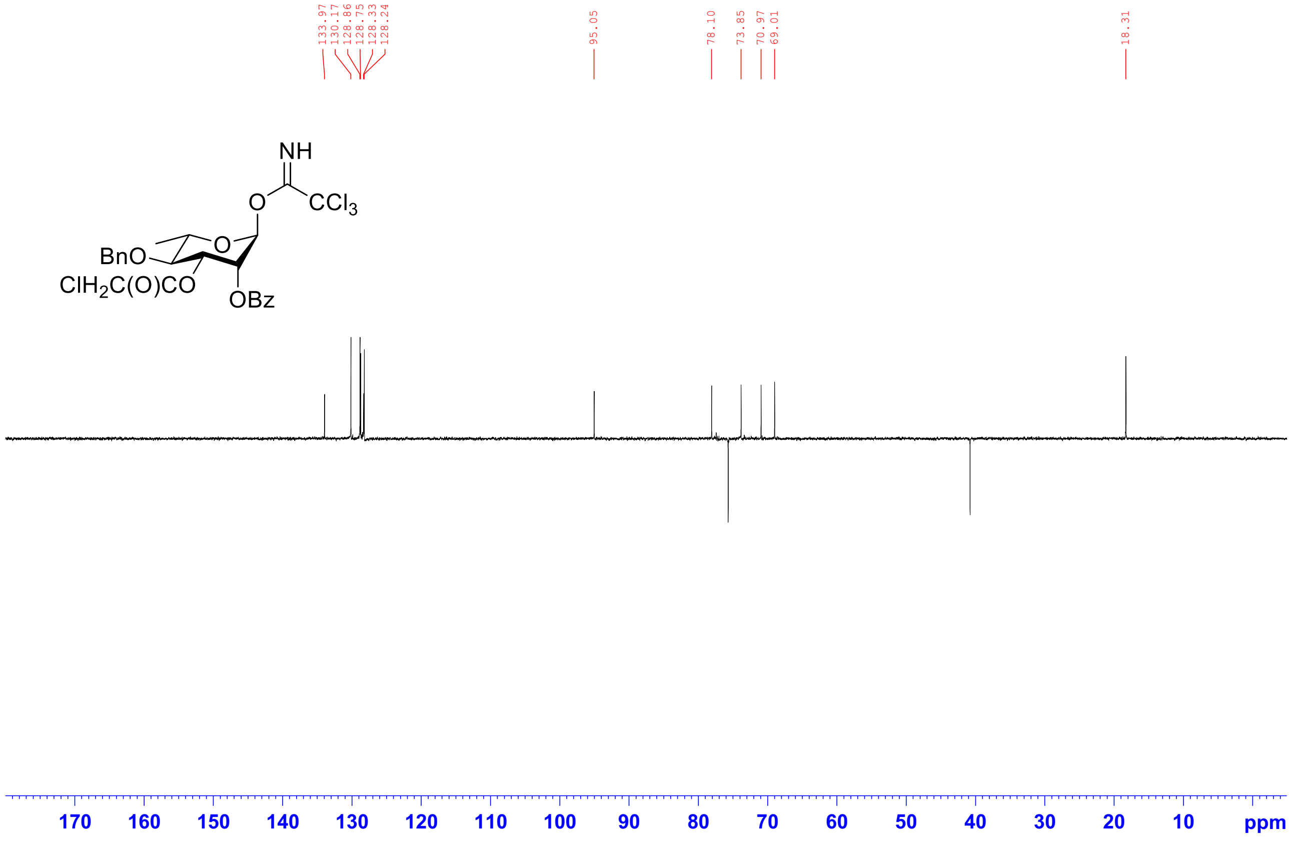

NMR spectra of ribitol

21: ^{1}H NMR (600 MHz, $CDCl_3$)

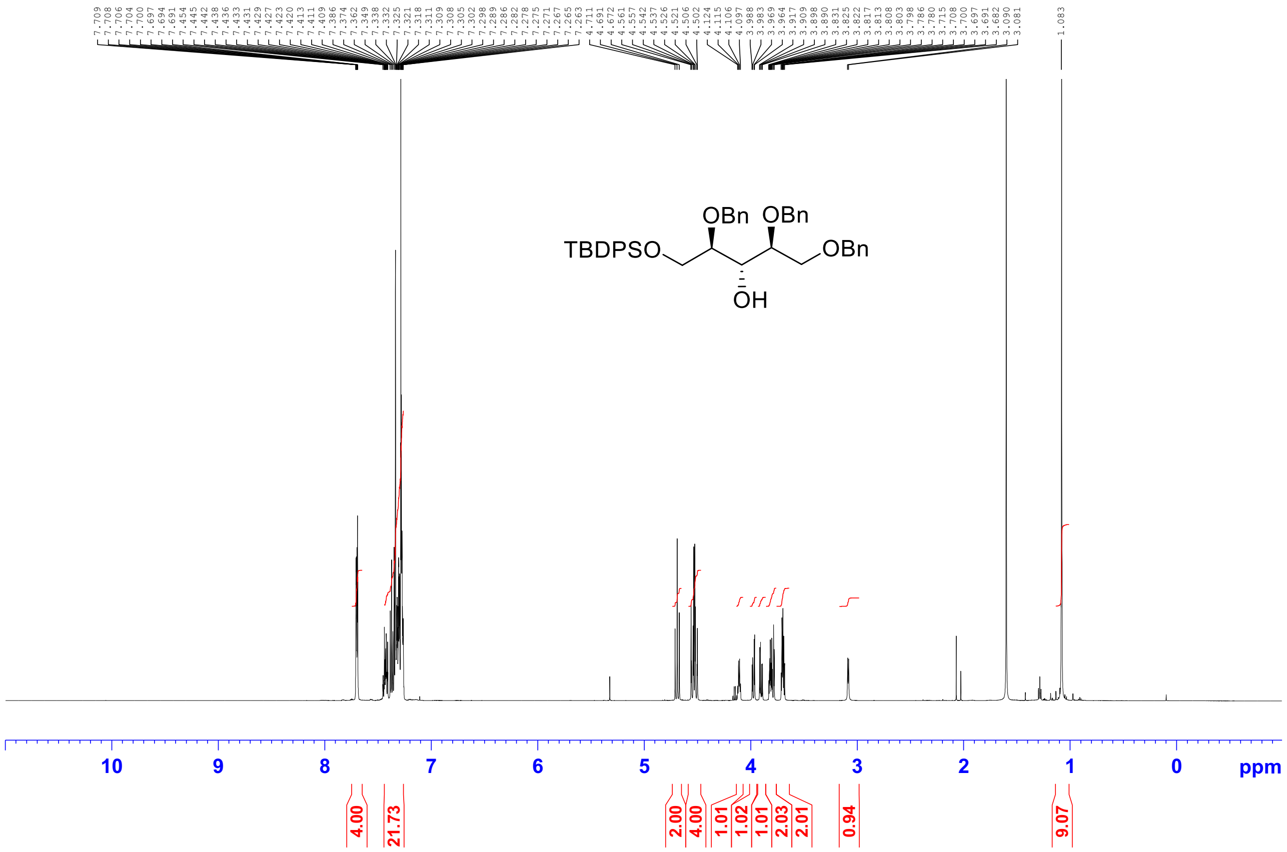

21: ^{13}C NMR (150 MHz, $CDCl_3$)

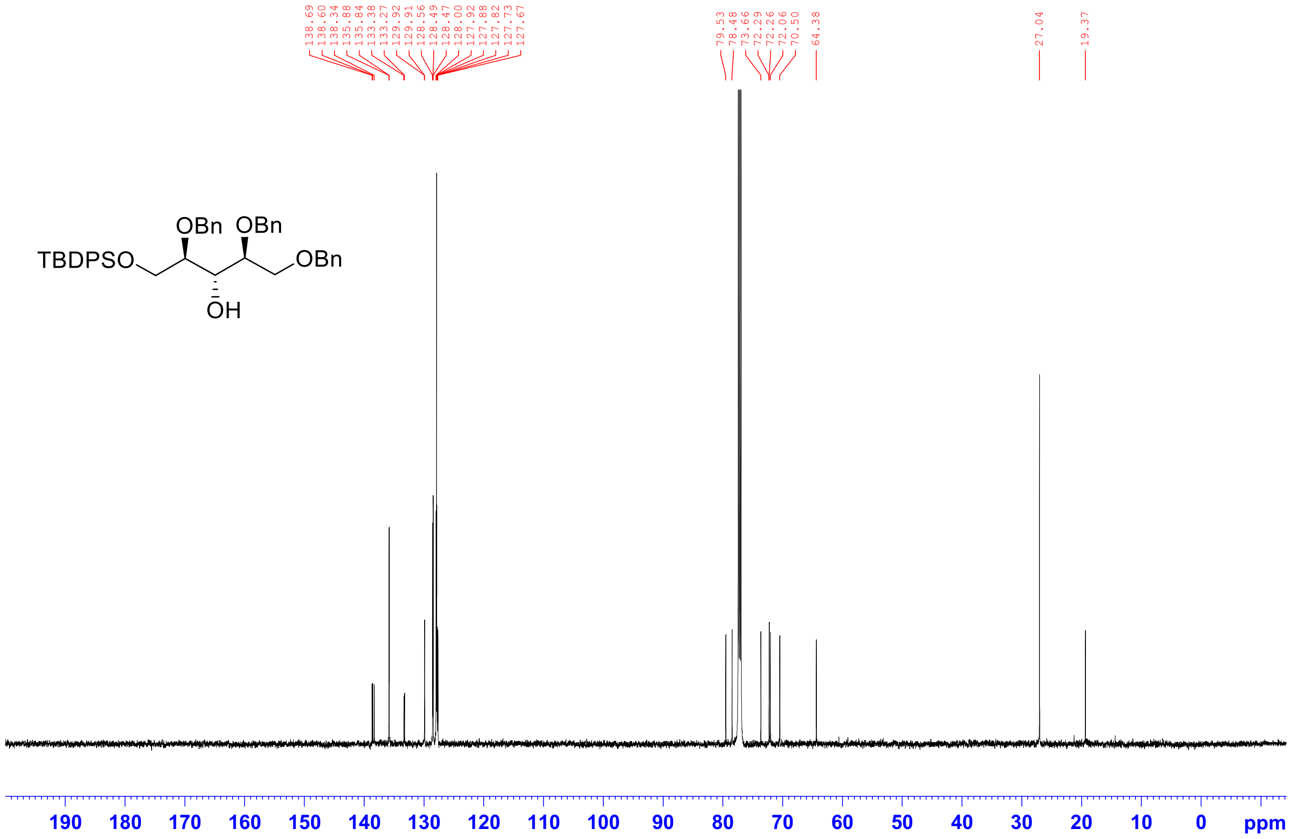

21: DEPT 135 (150 MHz, CDCl$_3$)

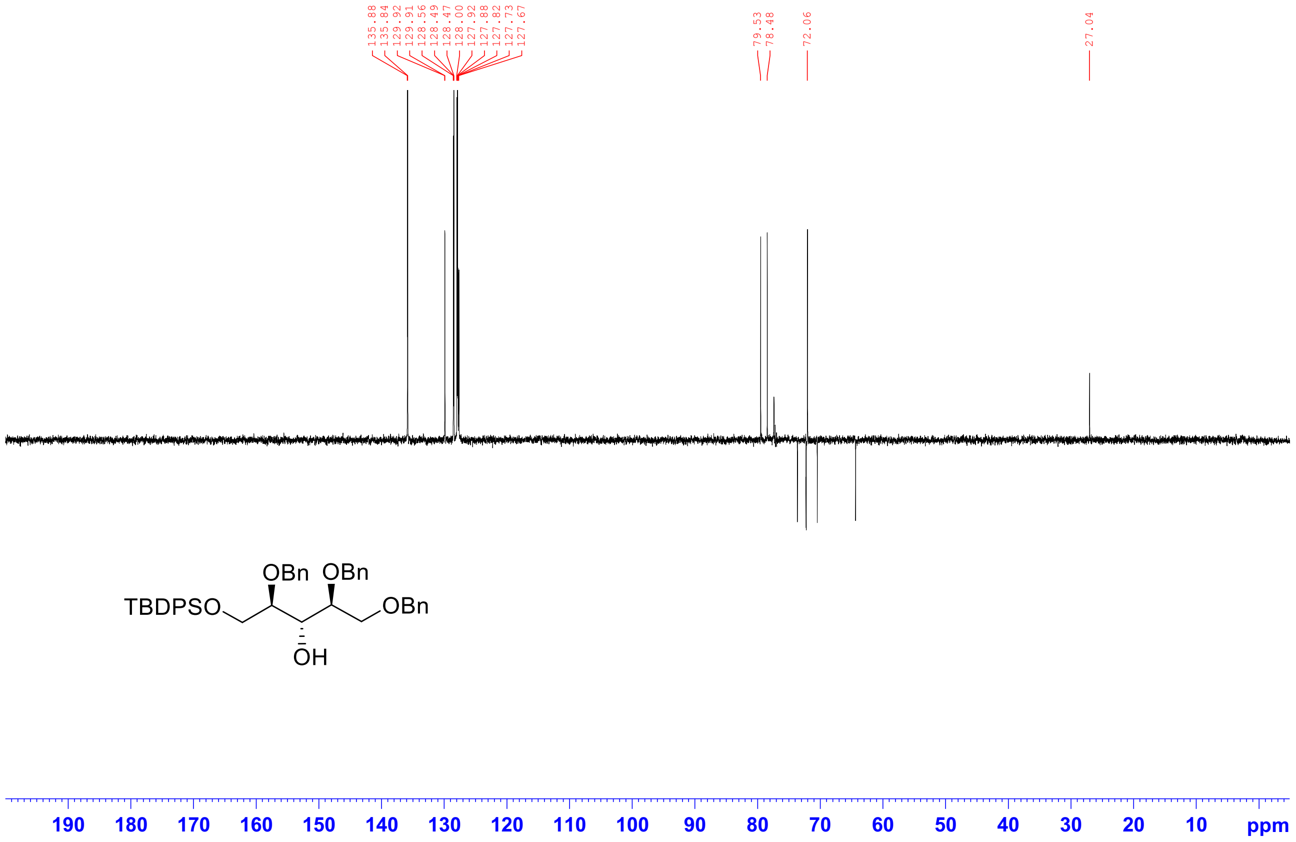

S13: ^{1}H NMR (600 MHz, CDCl$_3$)

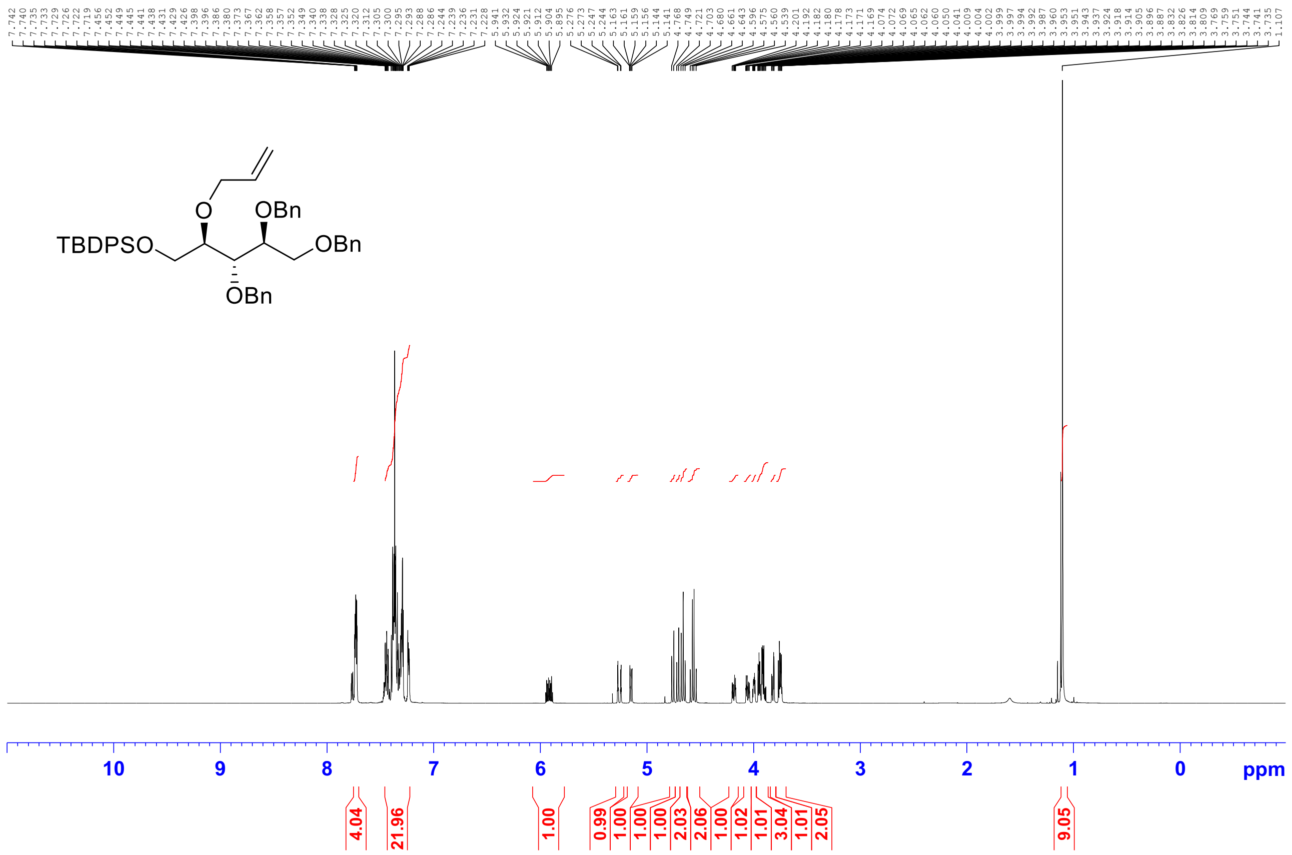

S13: ^{13}C NMR (150 MHz, CDCl$_3$)

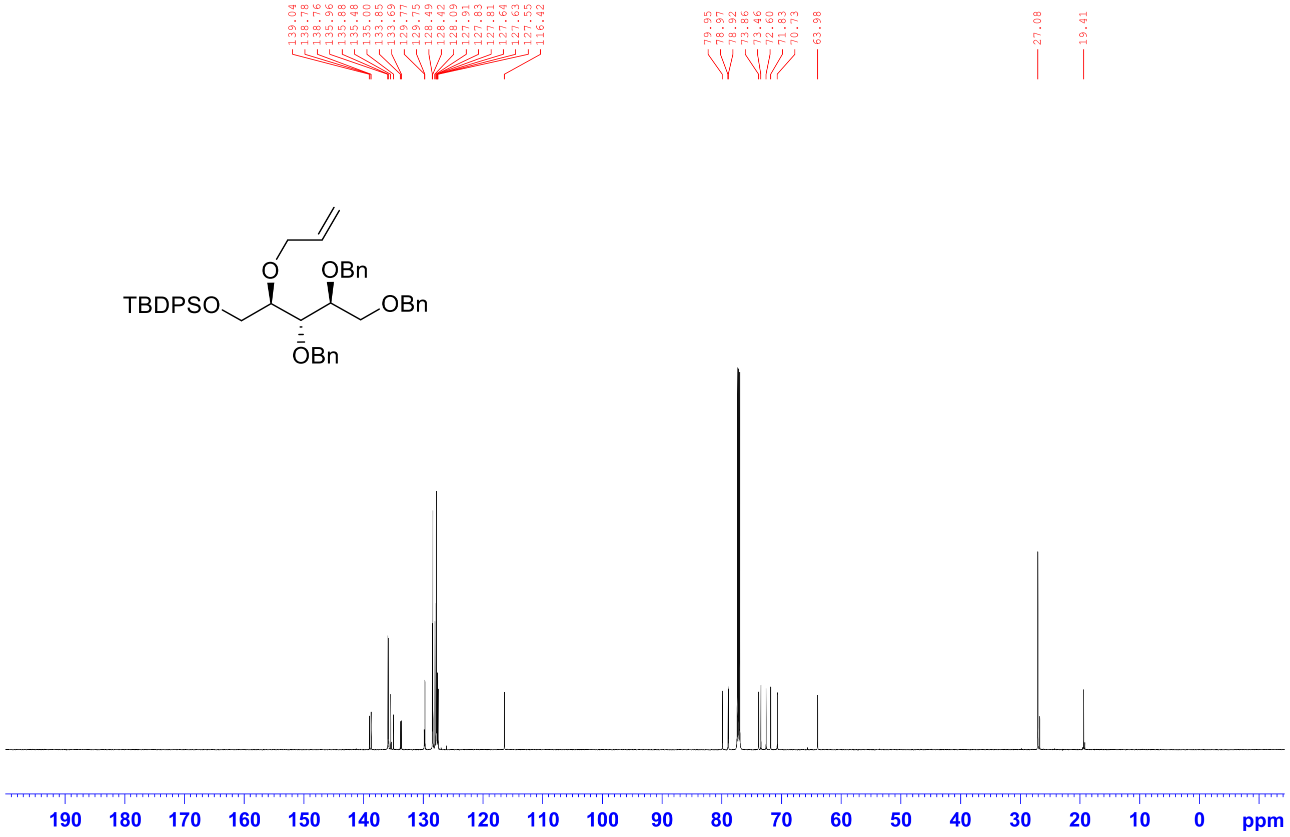

S13: DEPT 135 (150 MHz, CDCl$_3$)

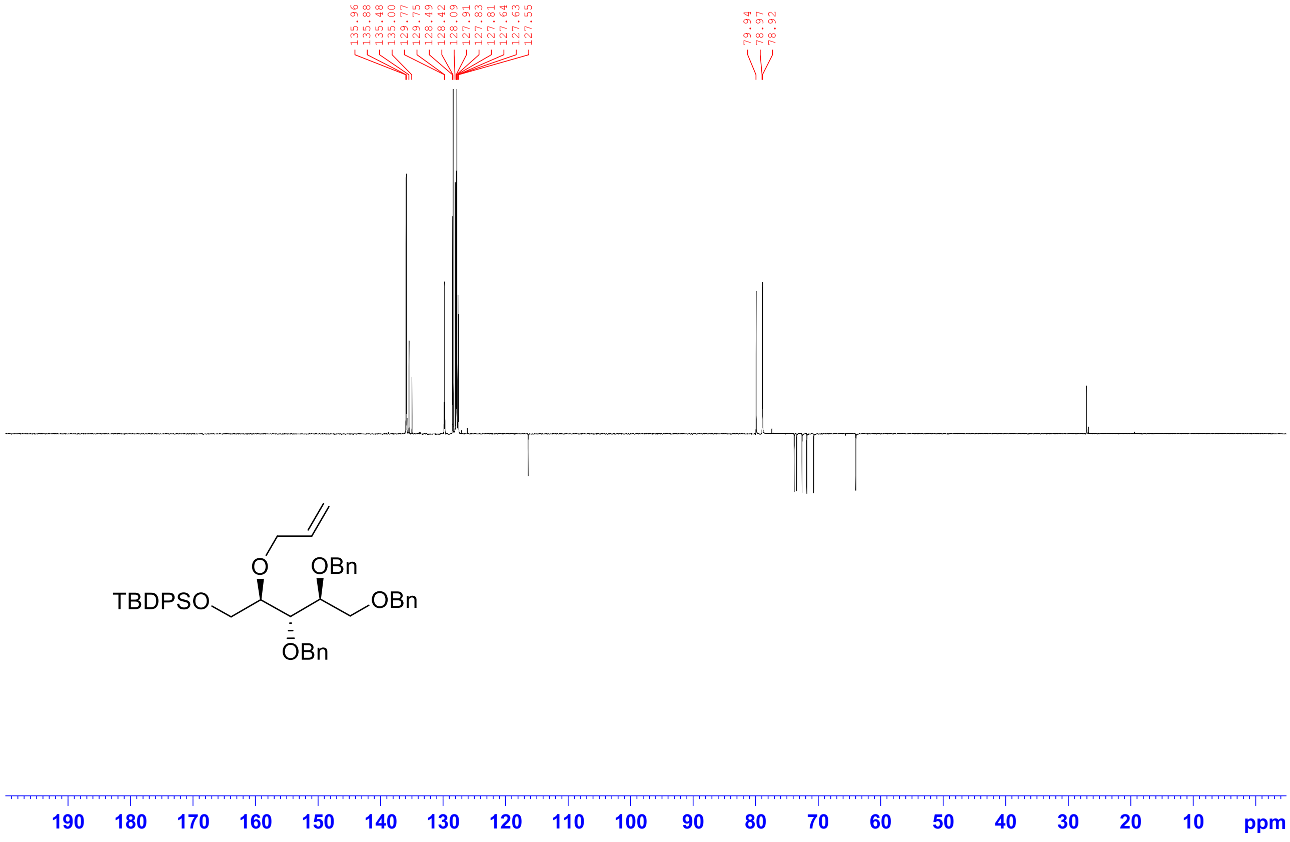

22: ^{1}H NMR (600 MHz, $CDCl_3$)

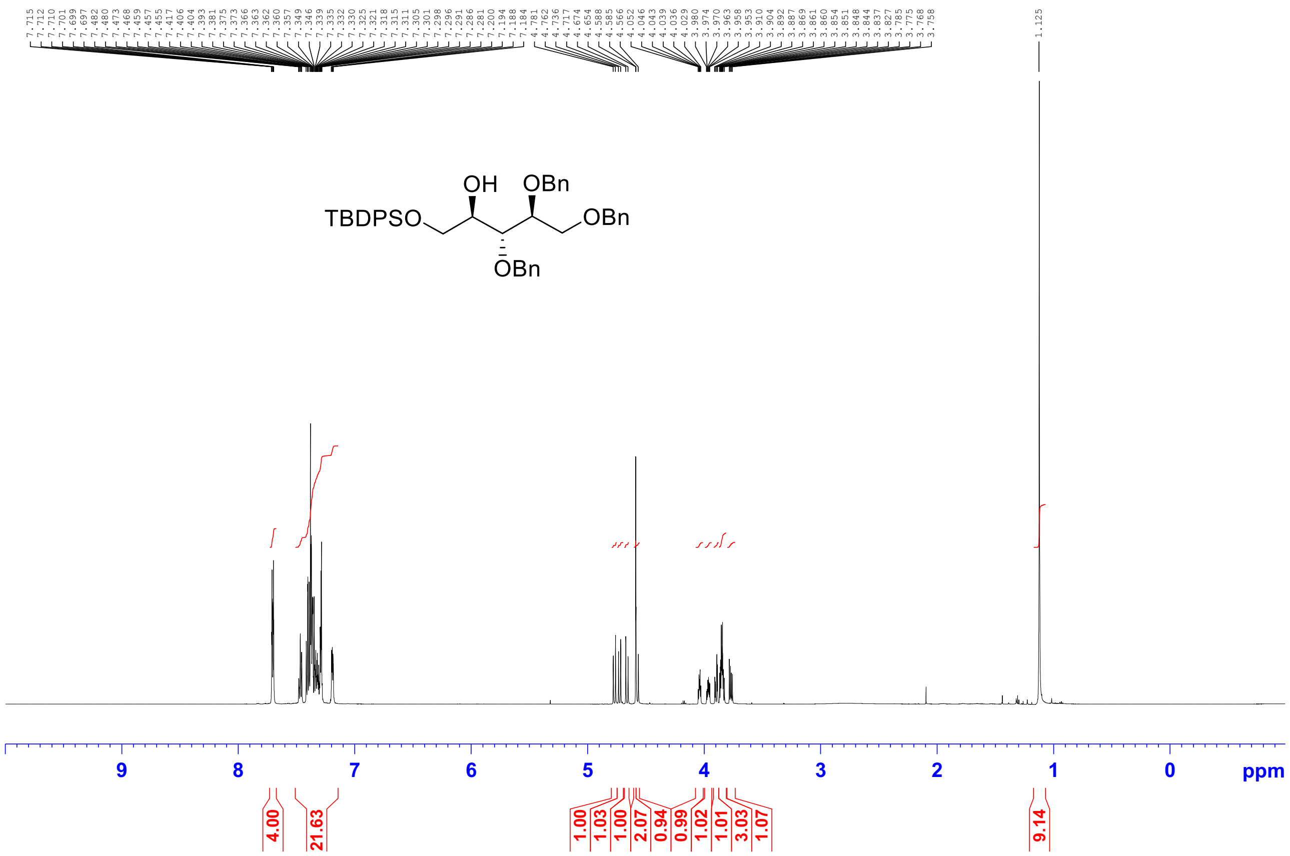

22: ^{13}C NMR (150 MHz, $CDCl_3$)

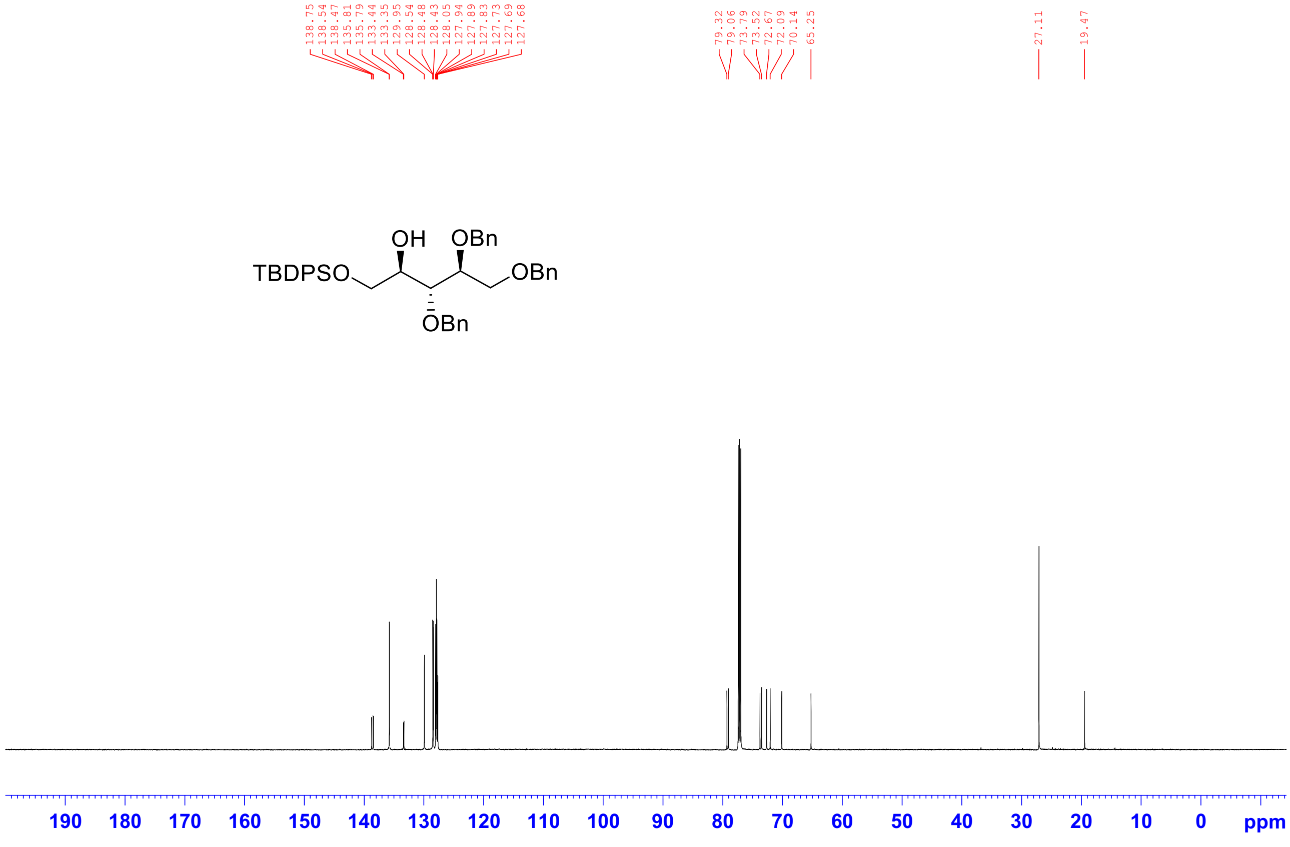

22: DEPT 135 (150 MHz, $CDCl_3$)

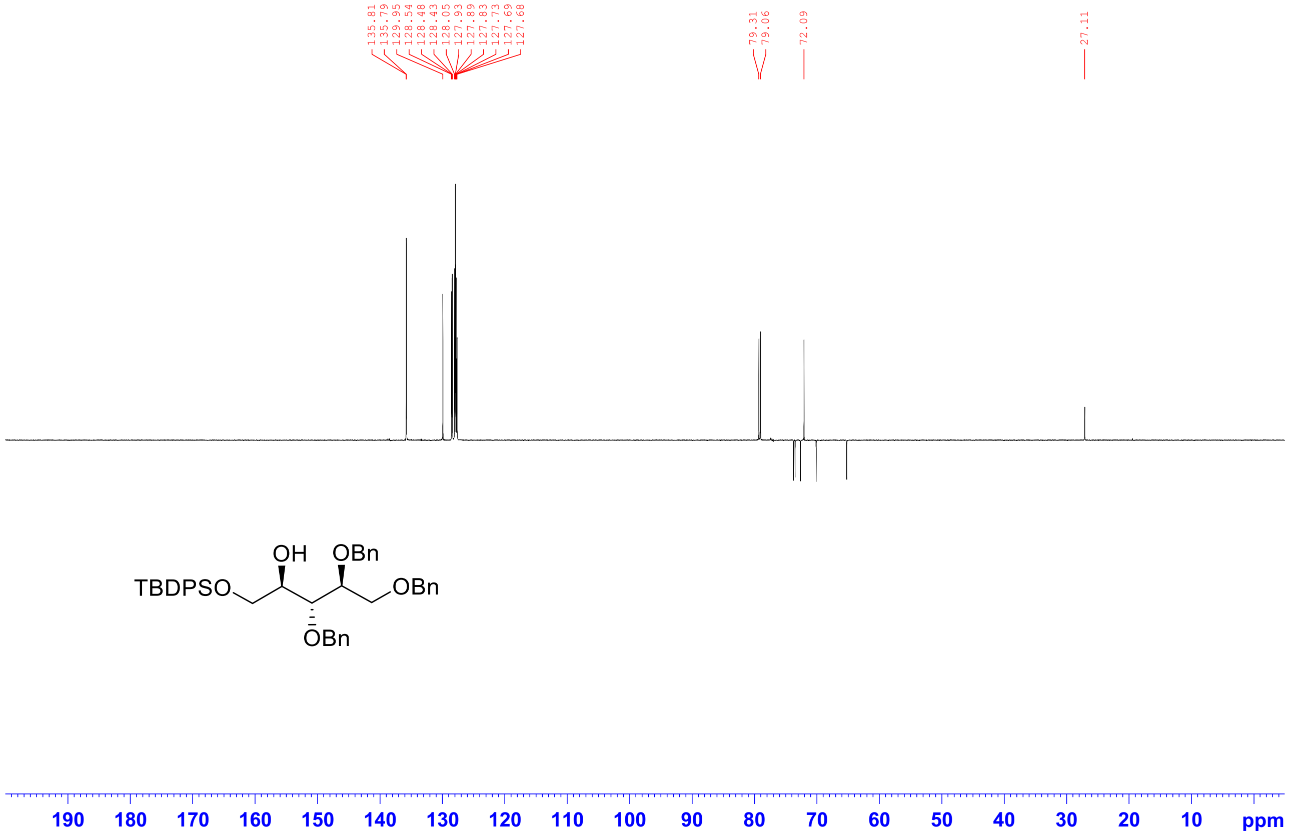

NMR spectra of Glc-Glc disaccharides

16: ^{1}H NMR (600 MHz, $CDCl_3$)

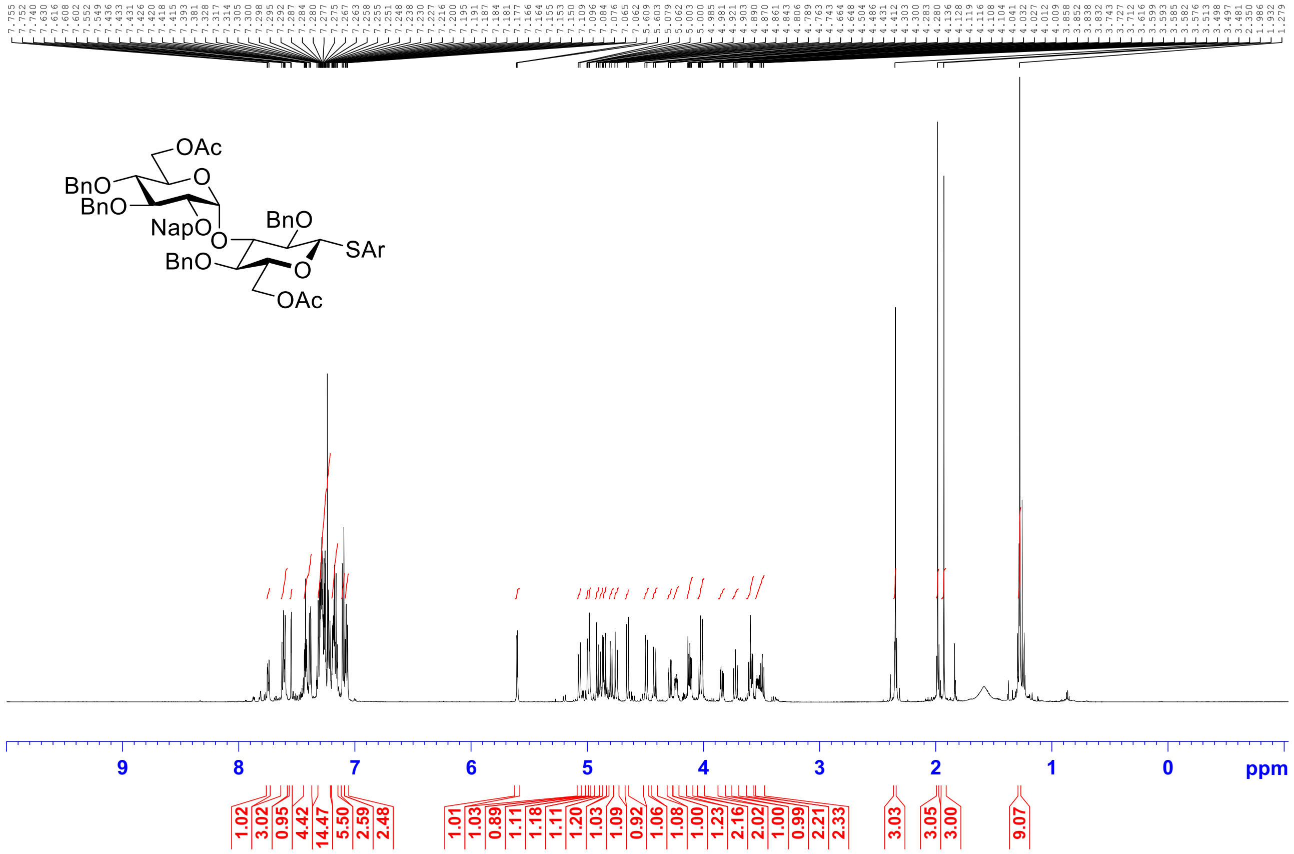

16: ^{13}C NMR (150 MHz, CDCl$_3$)

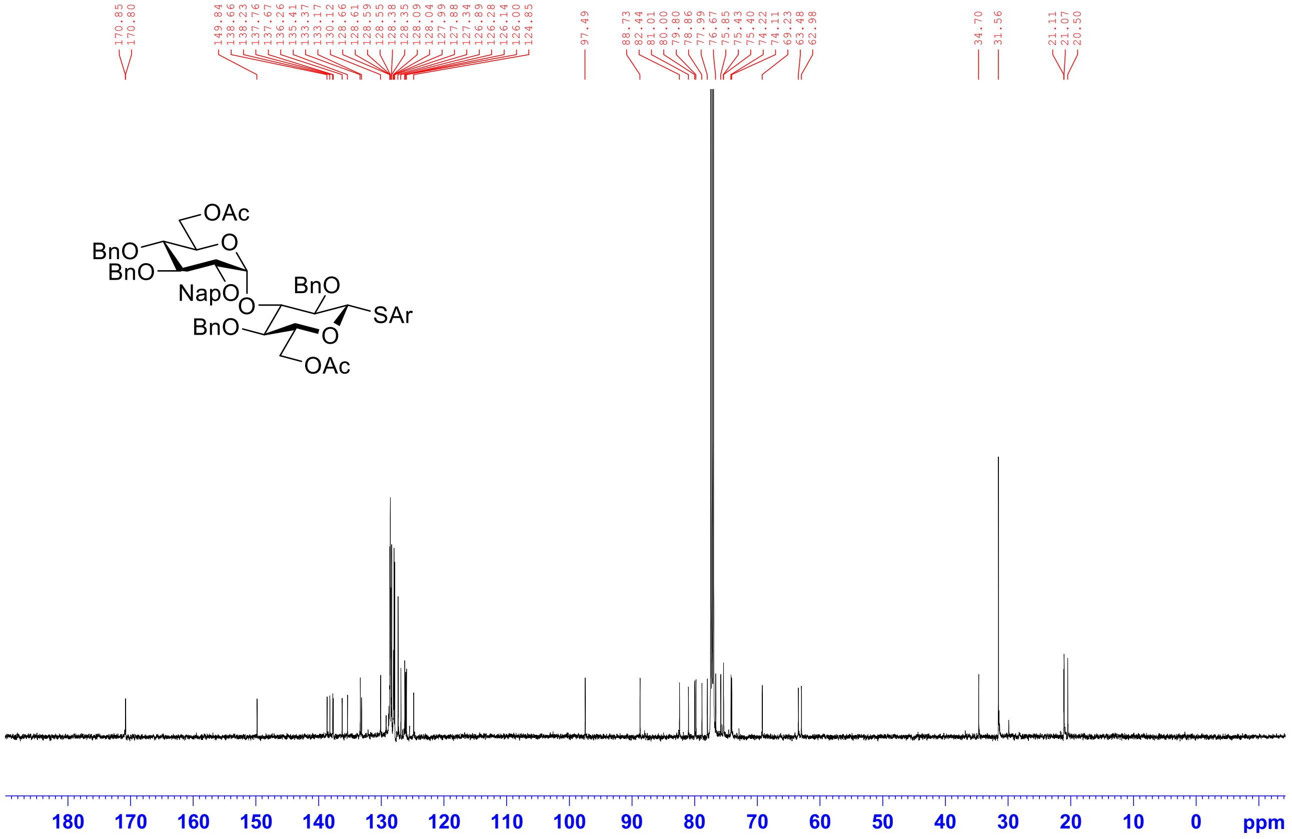

16: DEPT 135 (150 MHz, CDCl$_3$)

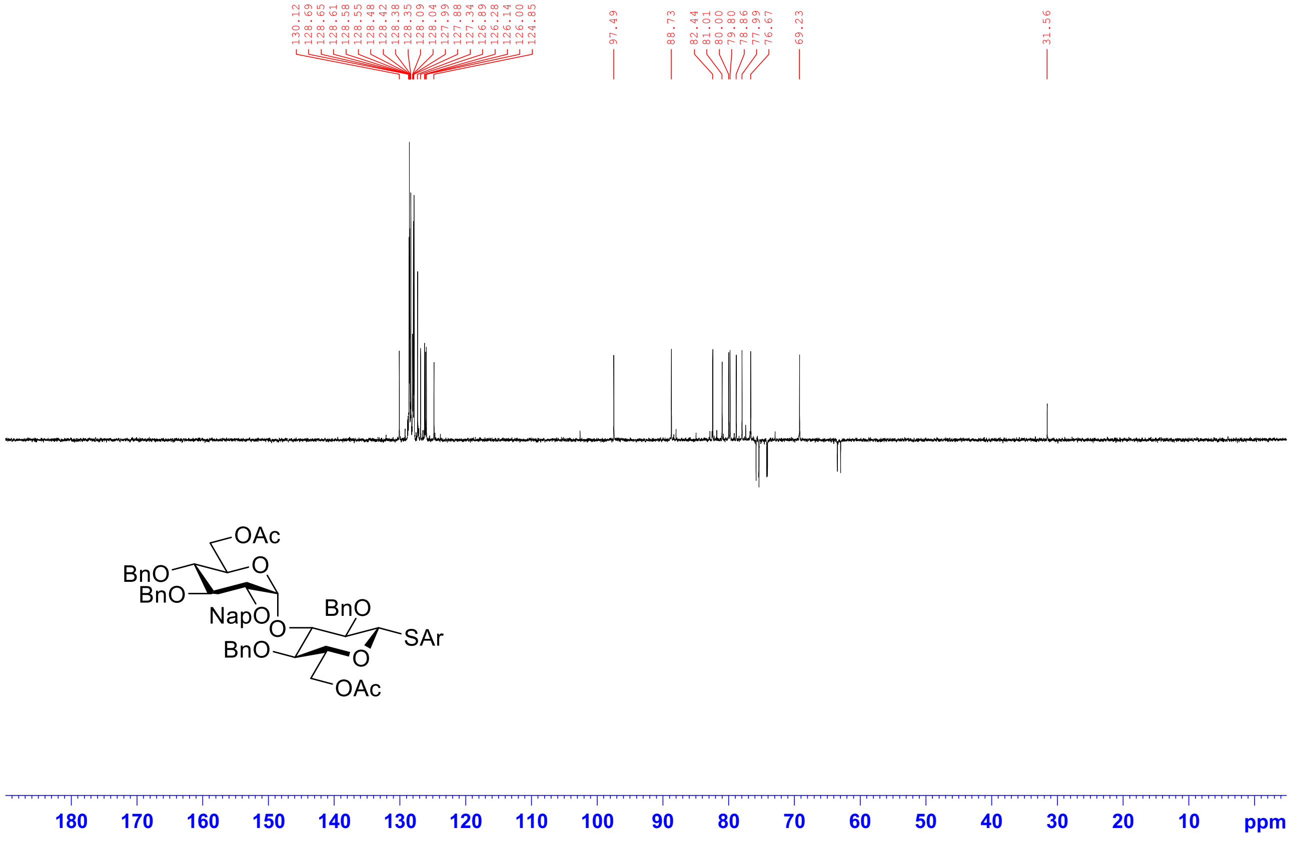

36: ^{1}H NMR (600 MHz, $CDCl_3$)

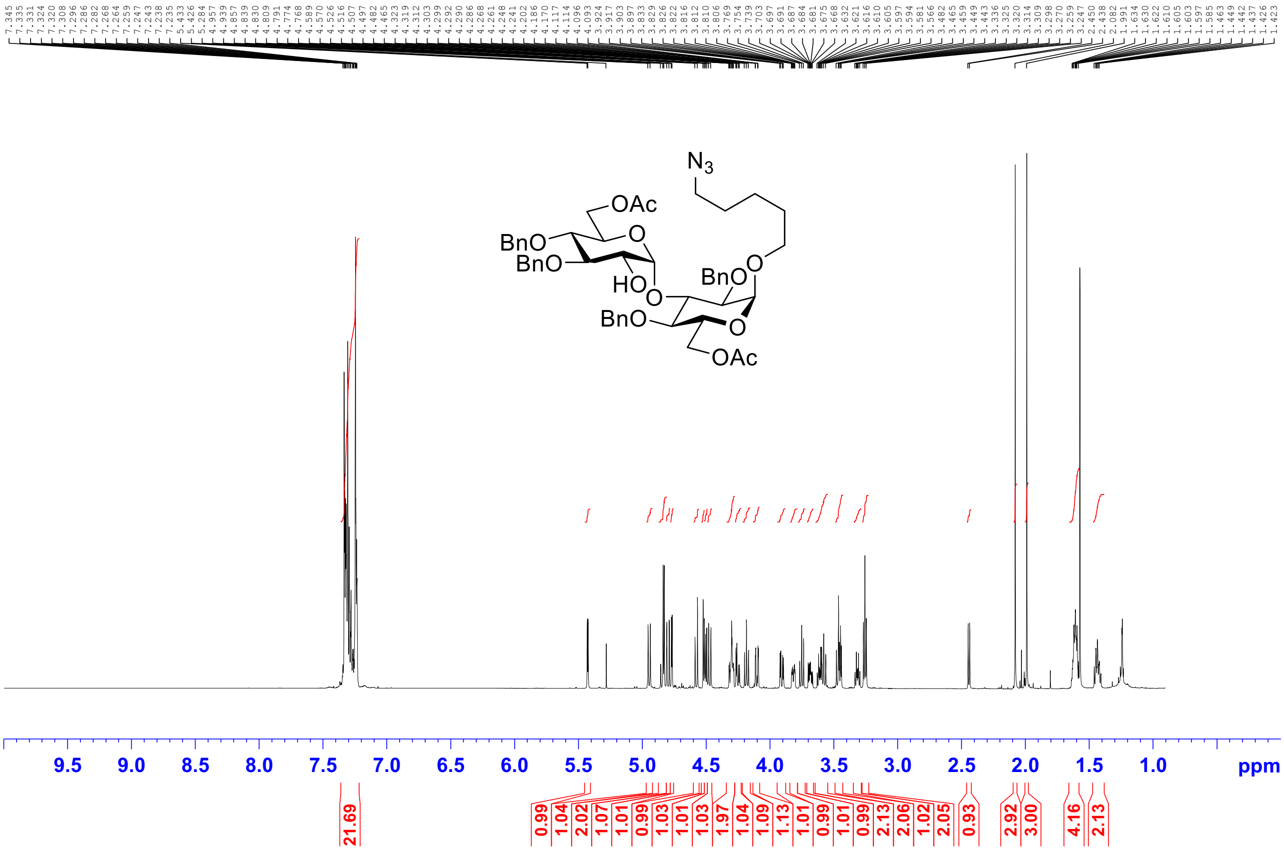

36: ^{13}C NMR (150 MHz, $CDCl_3$)

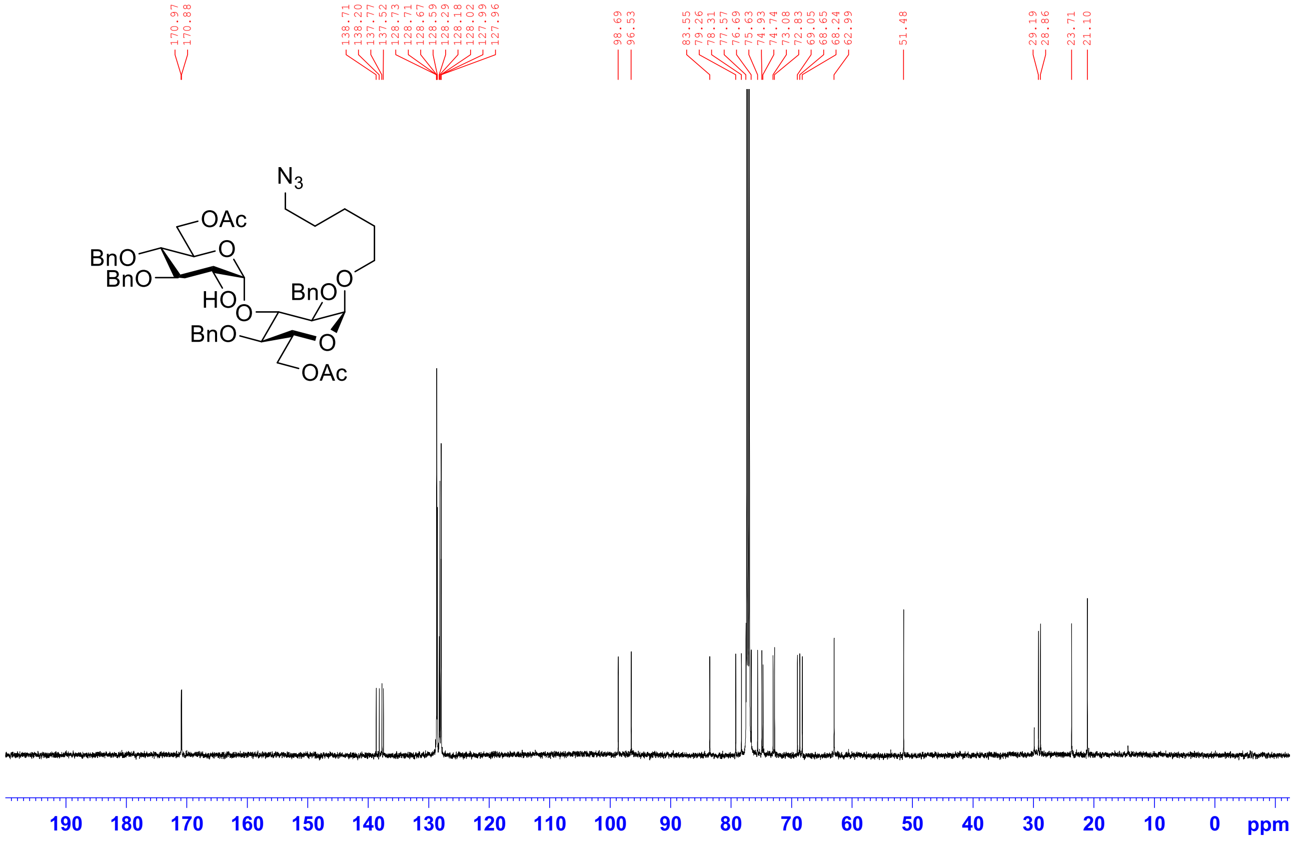

36: DEPT 135 (150 MHz, $CDCl_3$)

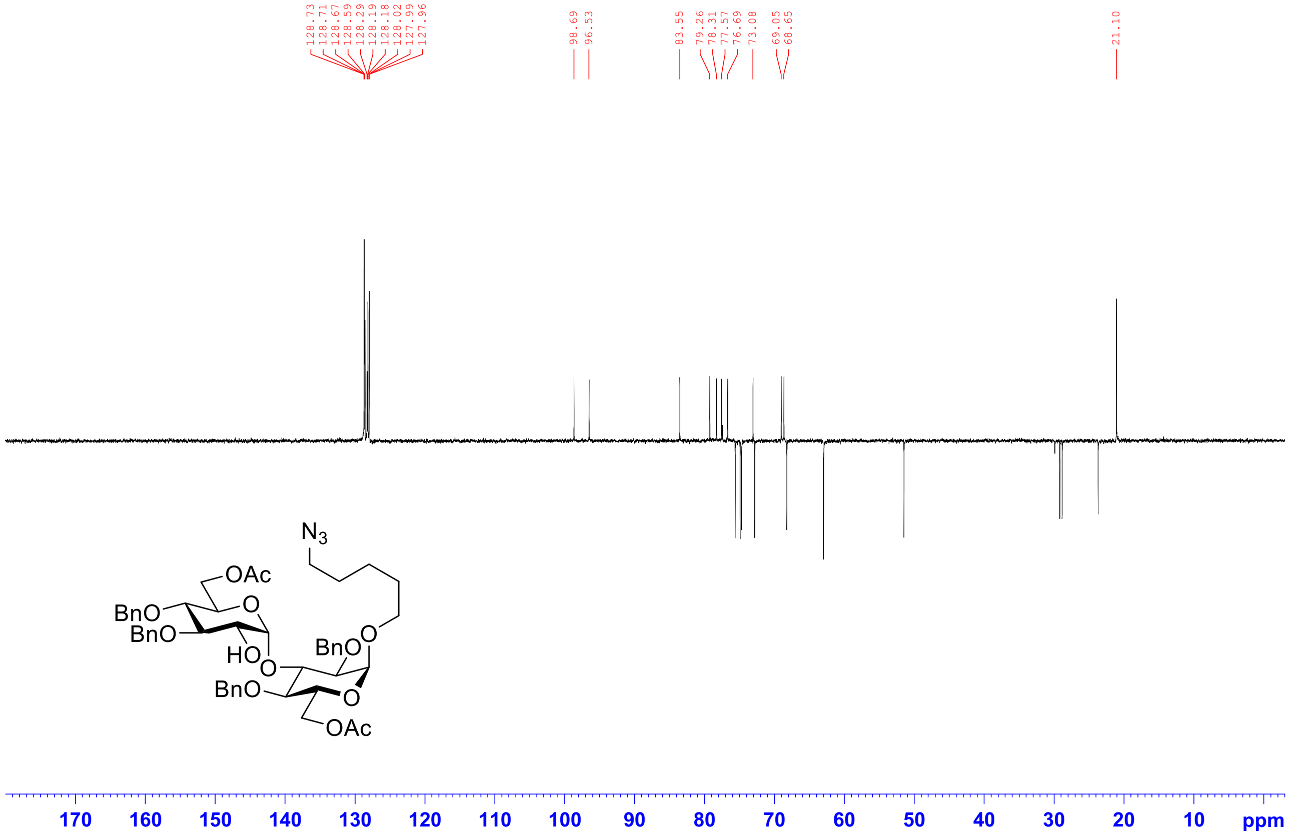

NMR spectra of Rha-Rbo pseudo-disaccharide

S17: ^{1}H NMR (600 MHz, $CDCl_3$)

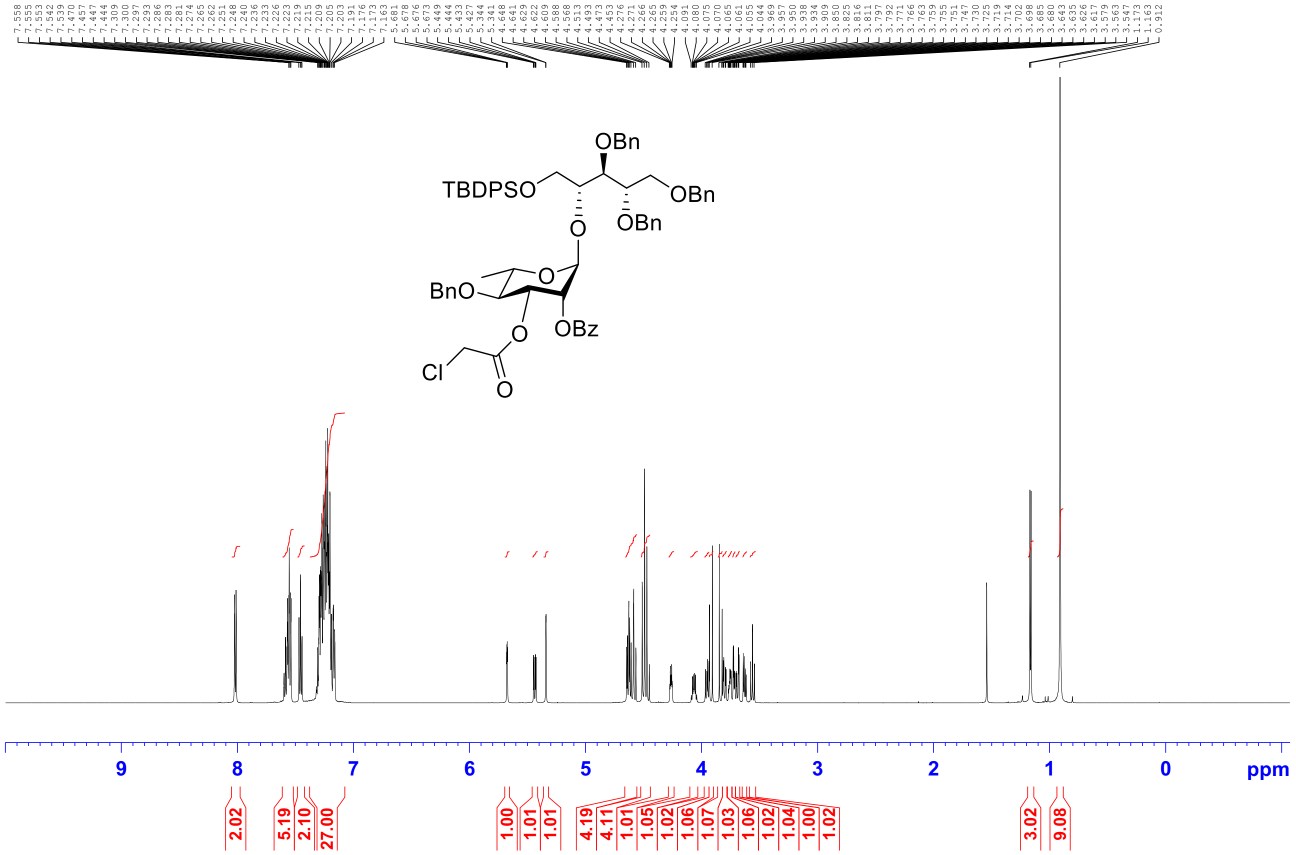

S17: ^{13}C NMR (150 MHz, $CDCl_3$)

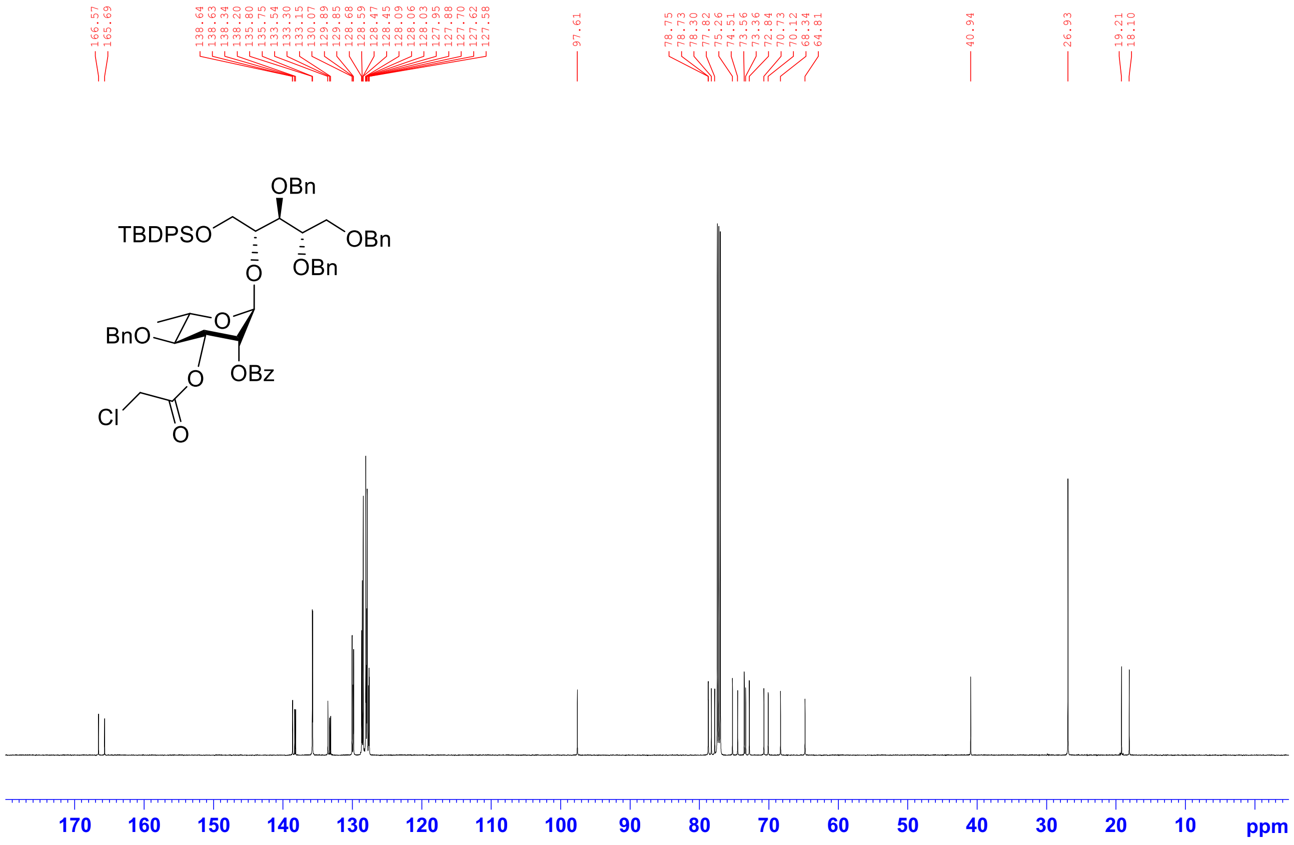

S17: DEPT 135 (150 MHz, $CDCl_3$)

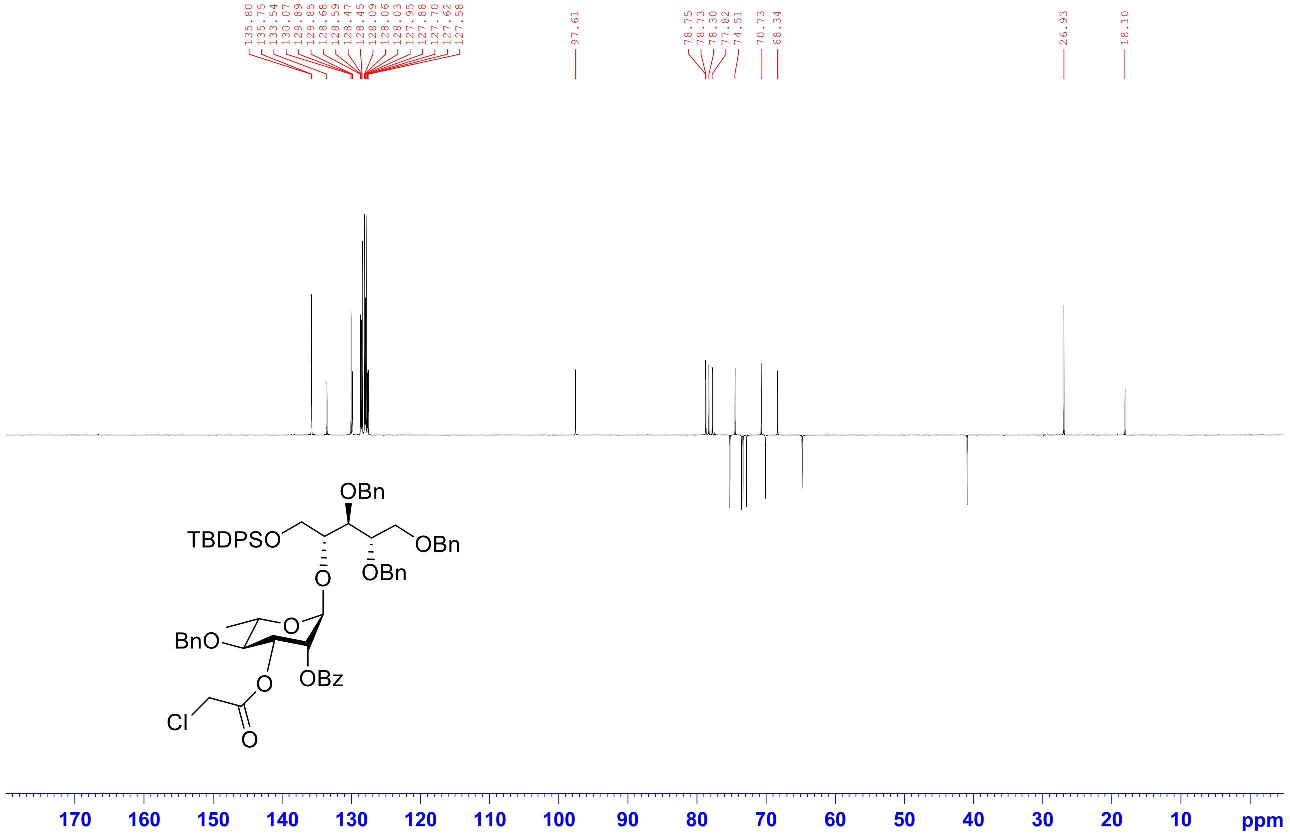

S18: ^{1}H NMR (600 MHz, $CDCl_3$)

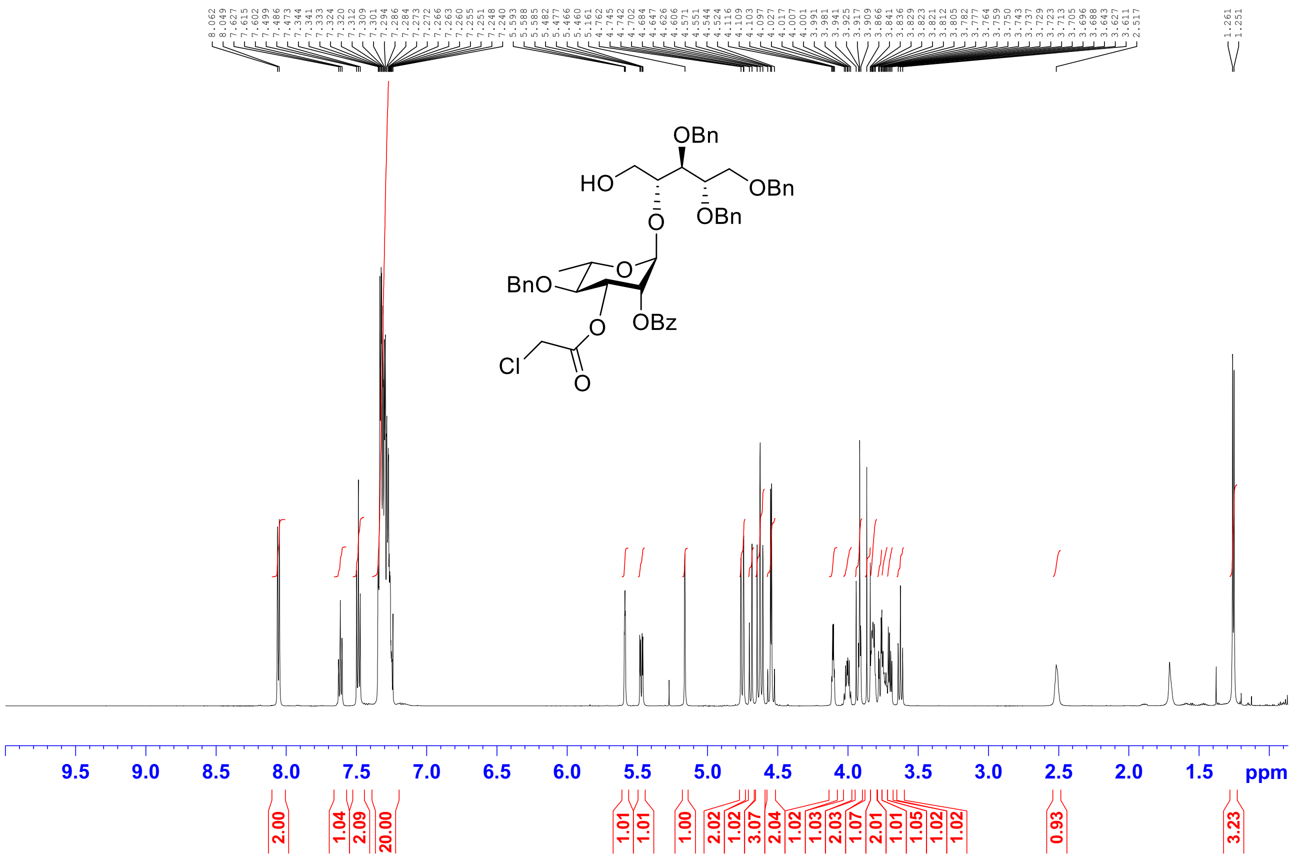

S18: ^{13}C NMR (150 MHz, $CDCl_3$)

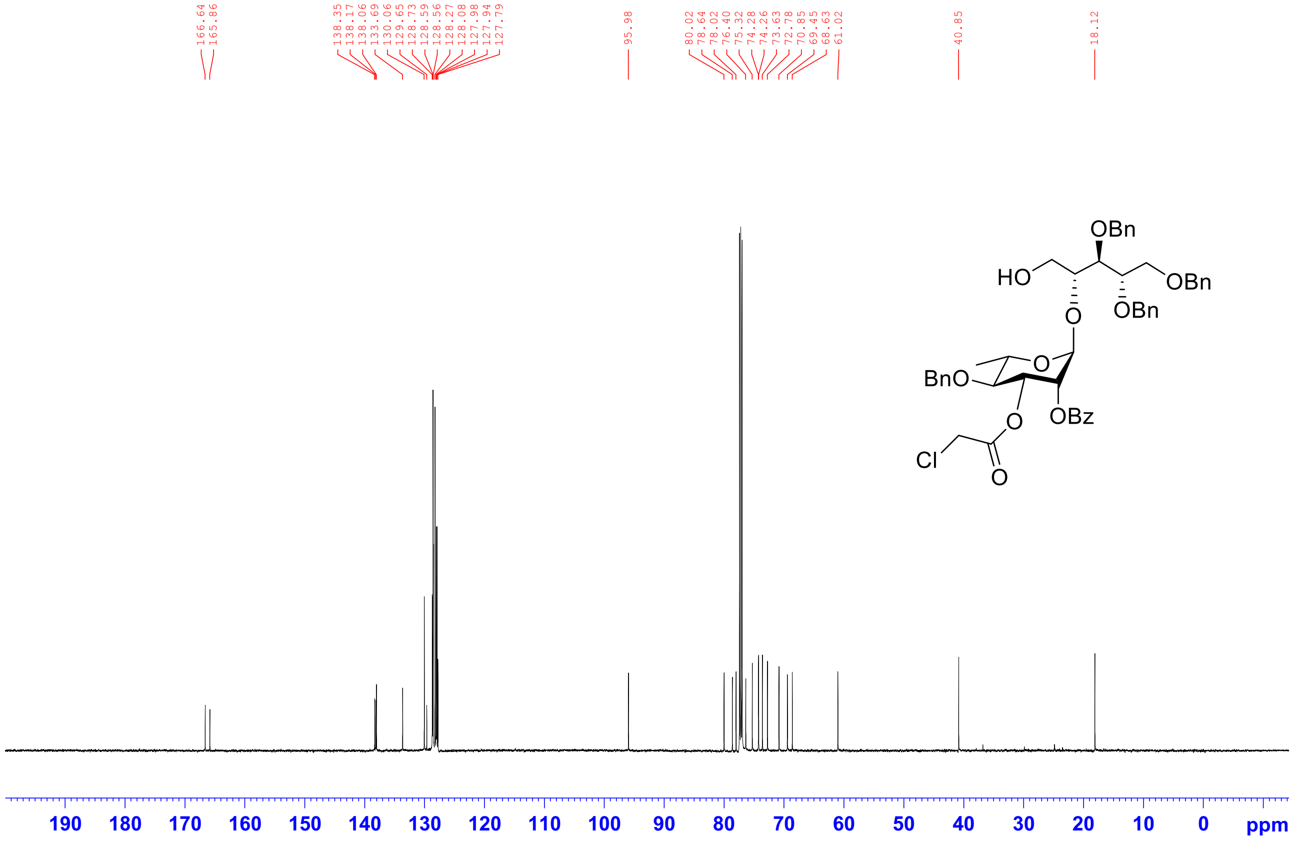

S18: DEPT 135 (150 MHz, $CDCl_3$)

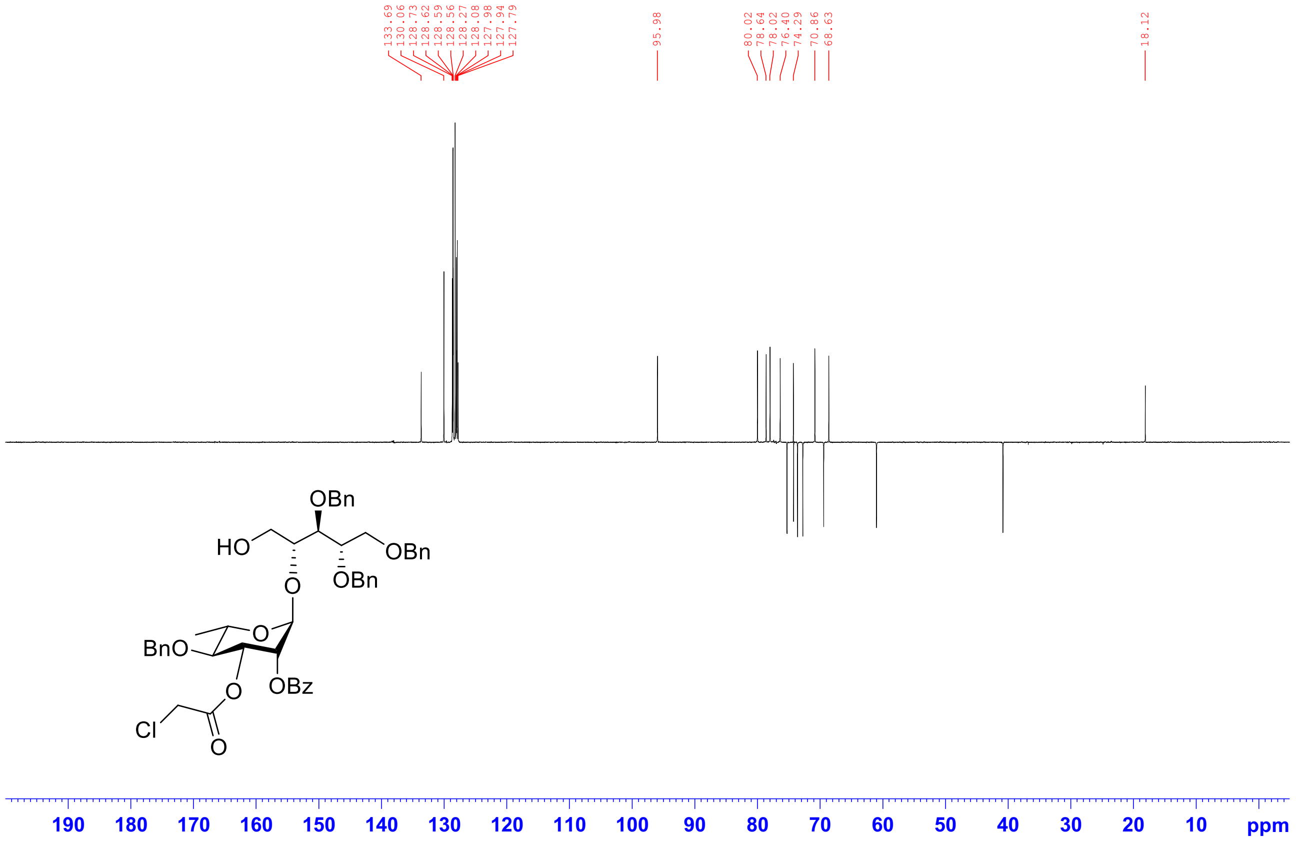

35: ^{1}H NMR (600 MHz, $CDCl_3$)

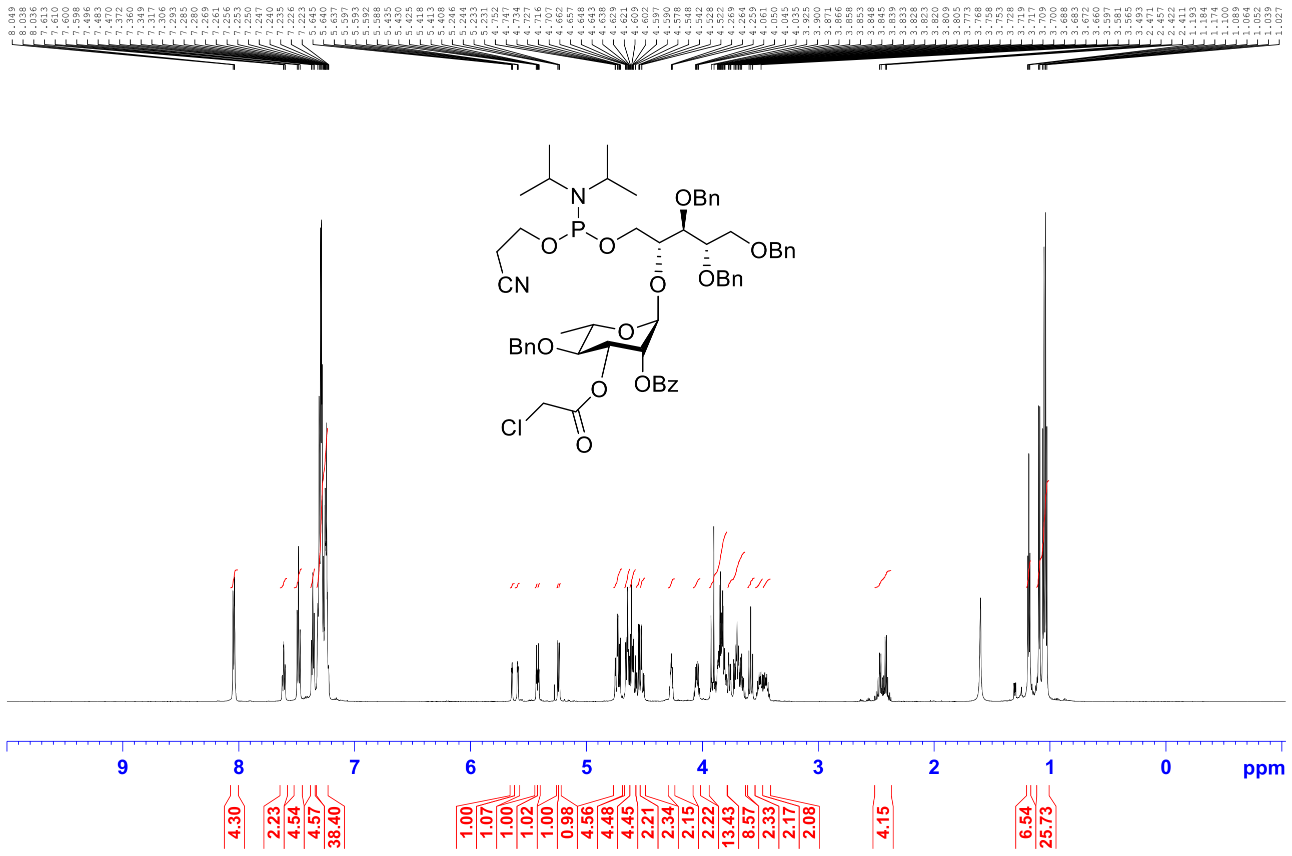

35: ^{13}C NMR (150 MHz, $CDCl_3$)

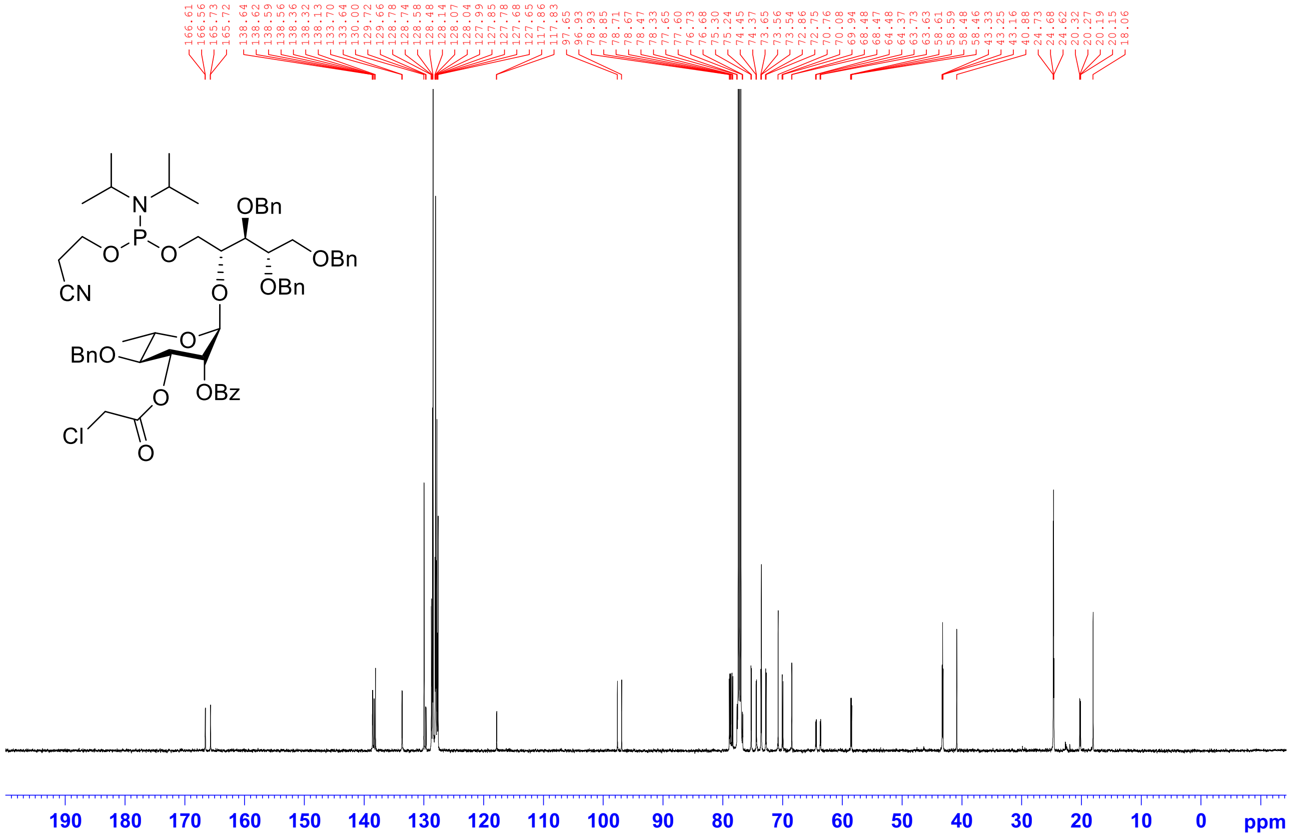

35: DEPT 135 (150 MHz, $CDCl_3$)

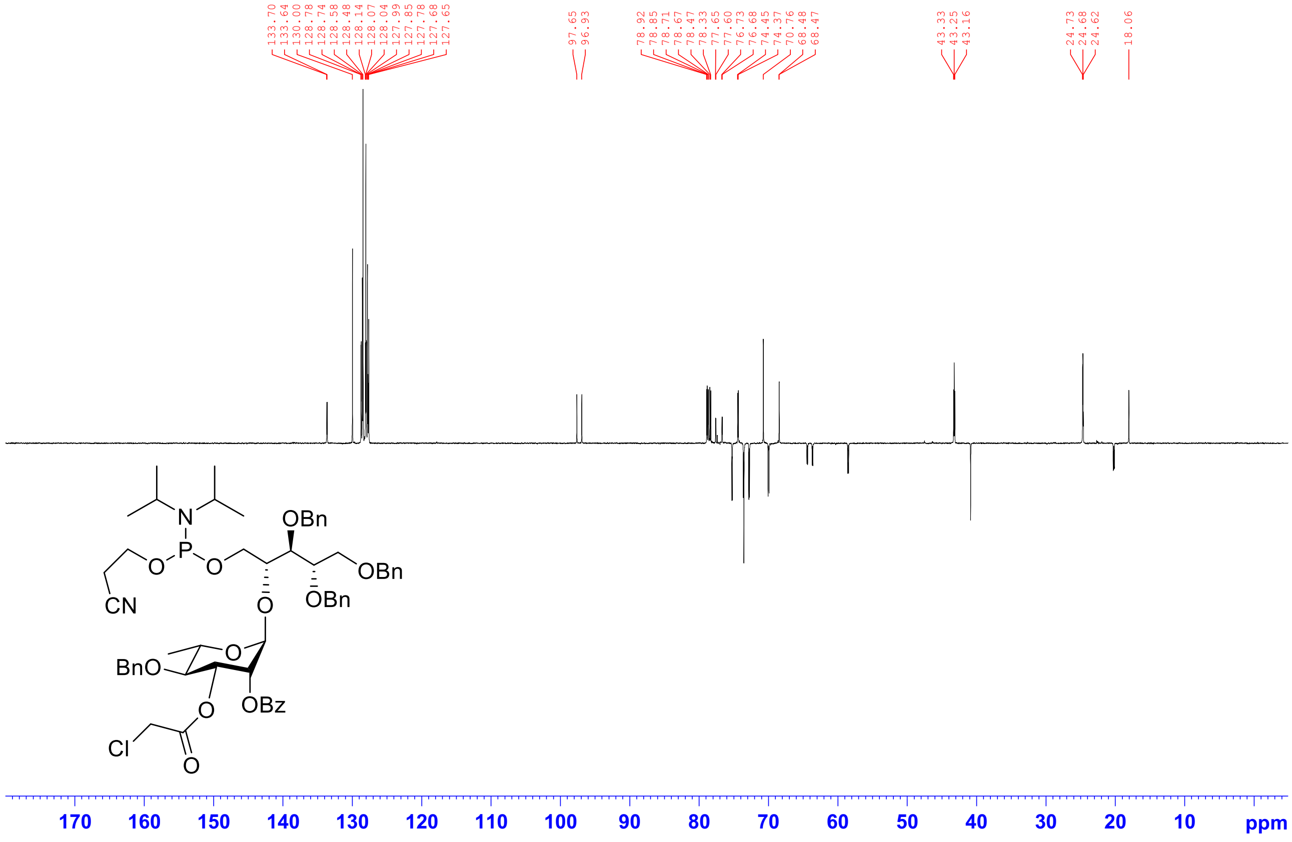

35: ^{31}P NMR (202 MHz, $CDCl_3$)

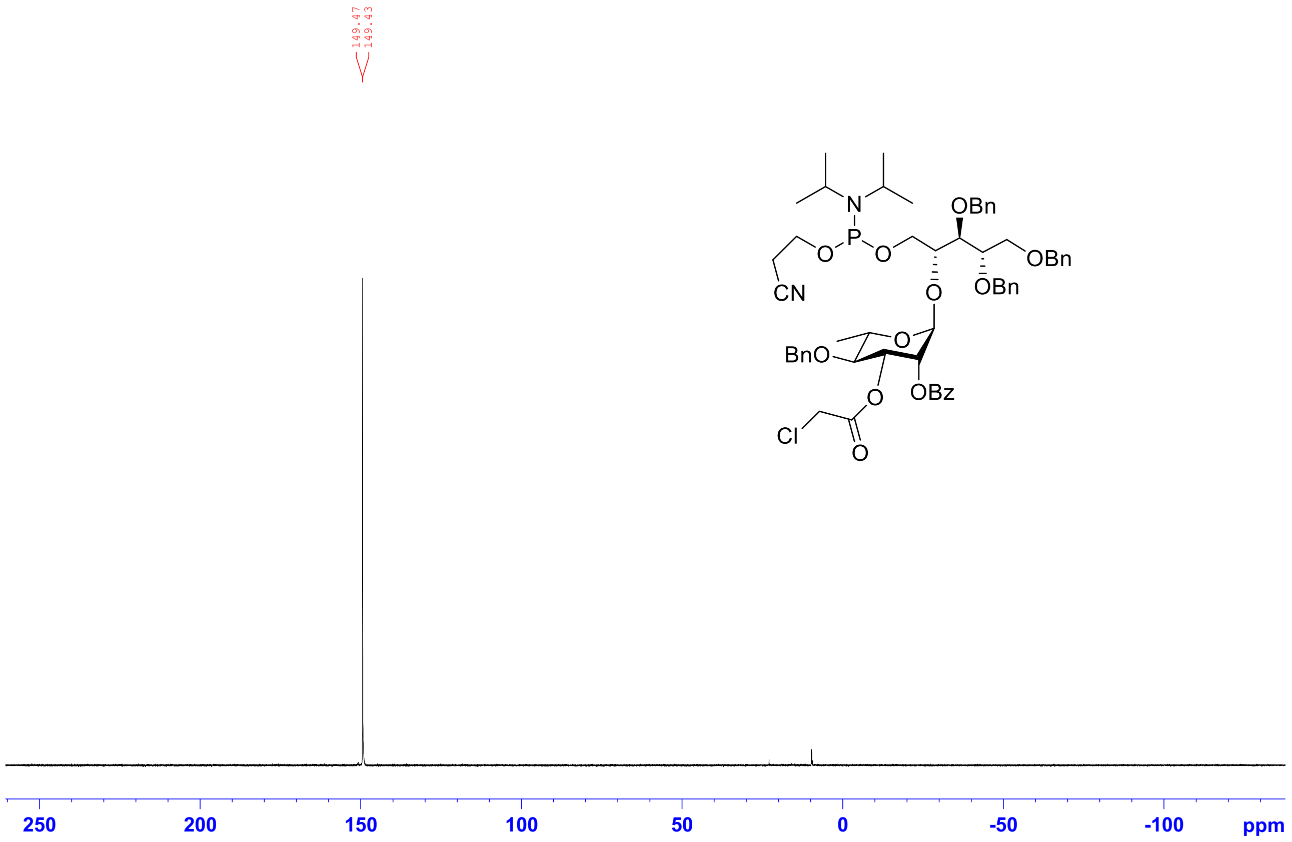

S15: ^{1}H NMR (600 MHz, $CDCl_3$)

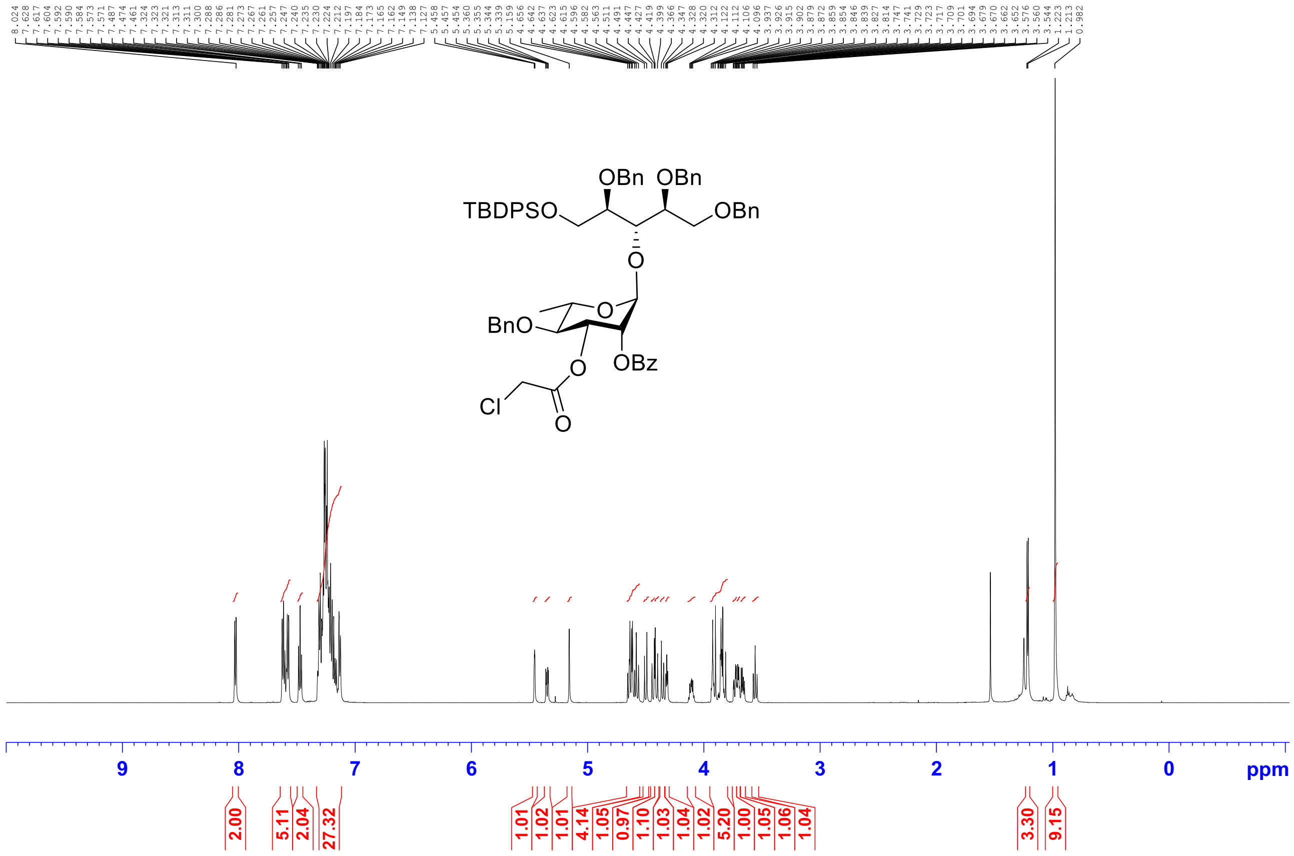

S15: ^{13}C NMR (150 MHz, CDCl$_3$)

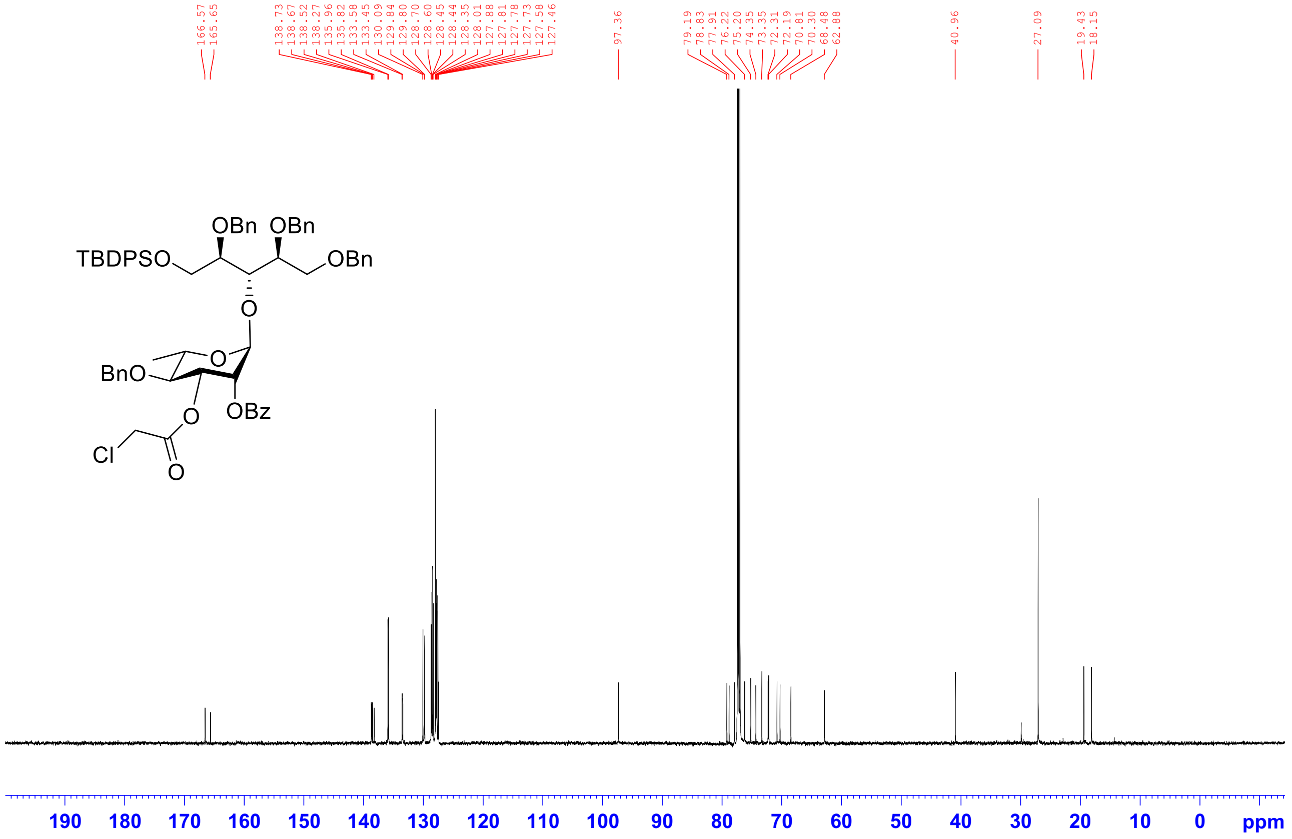

S15: DEPT 135 (150 MHz, CDCl$_3$)

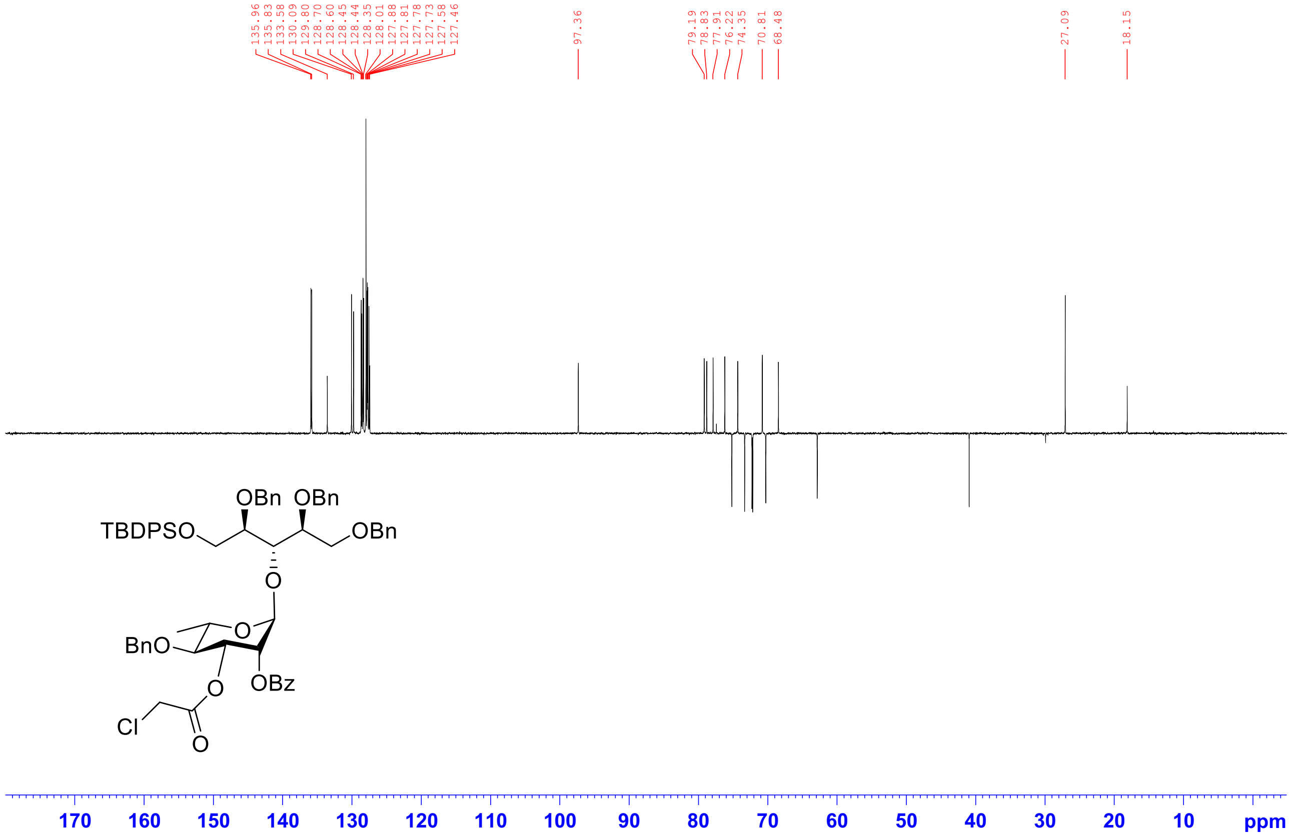

S16: ^{1}H NMR (600 MHz, $CDCl_3$)

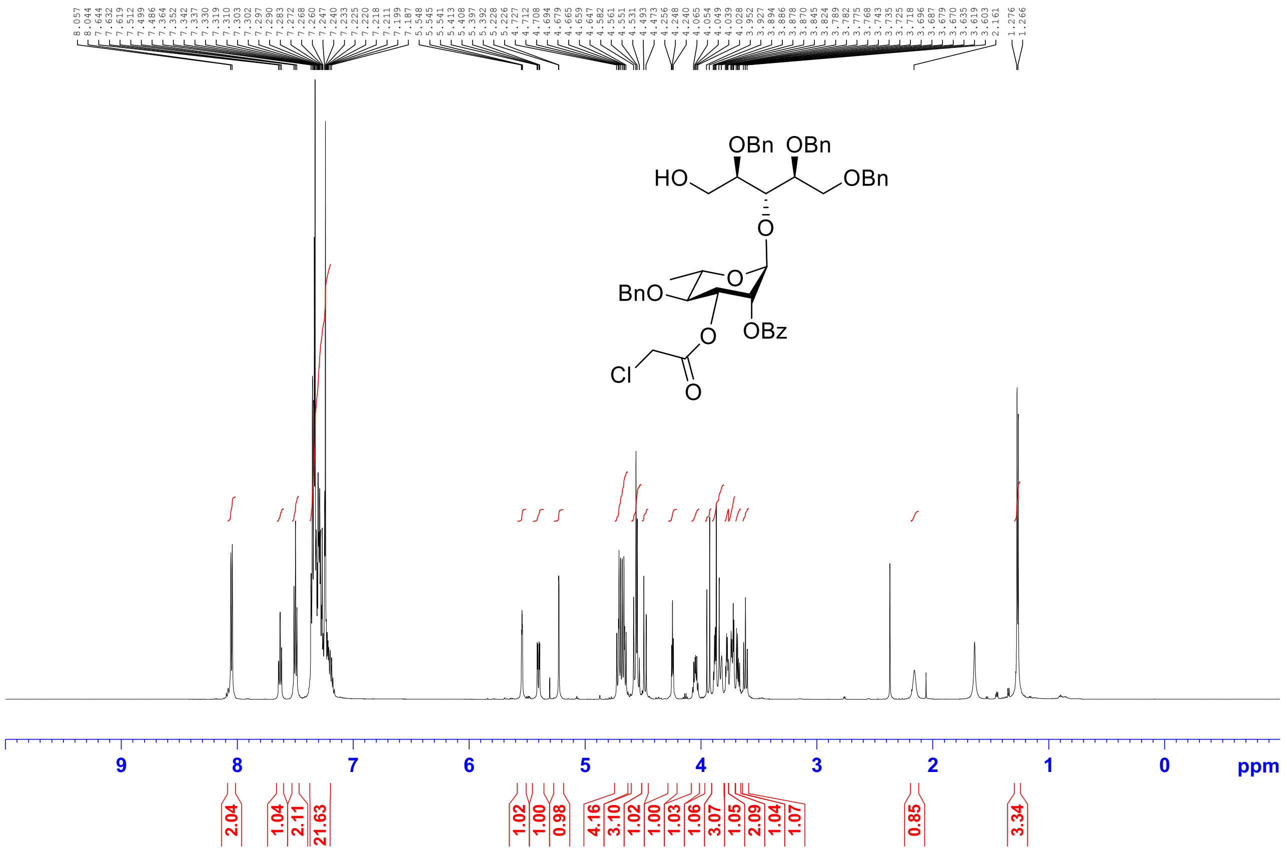

S16: ^{13}C NMR (150 MHz, $CDCl_3$)

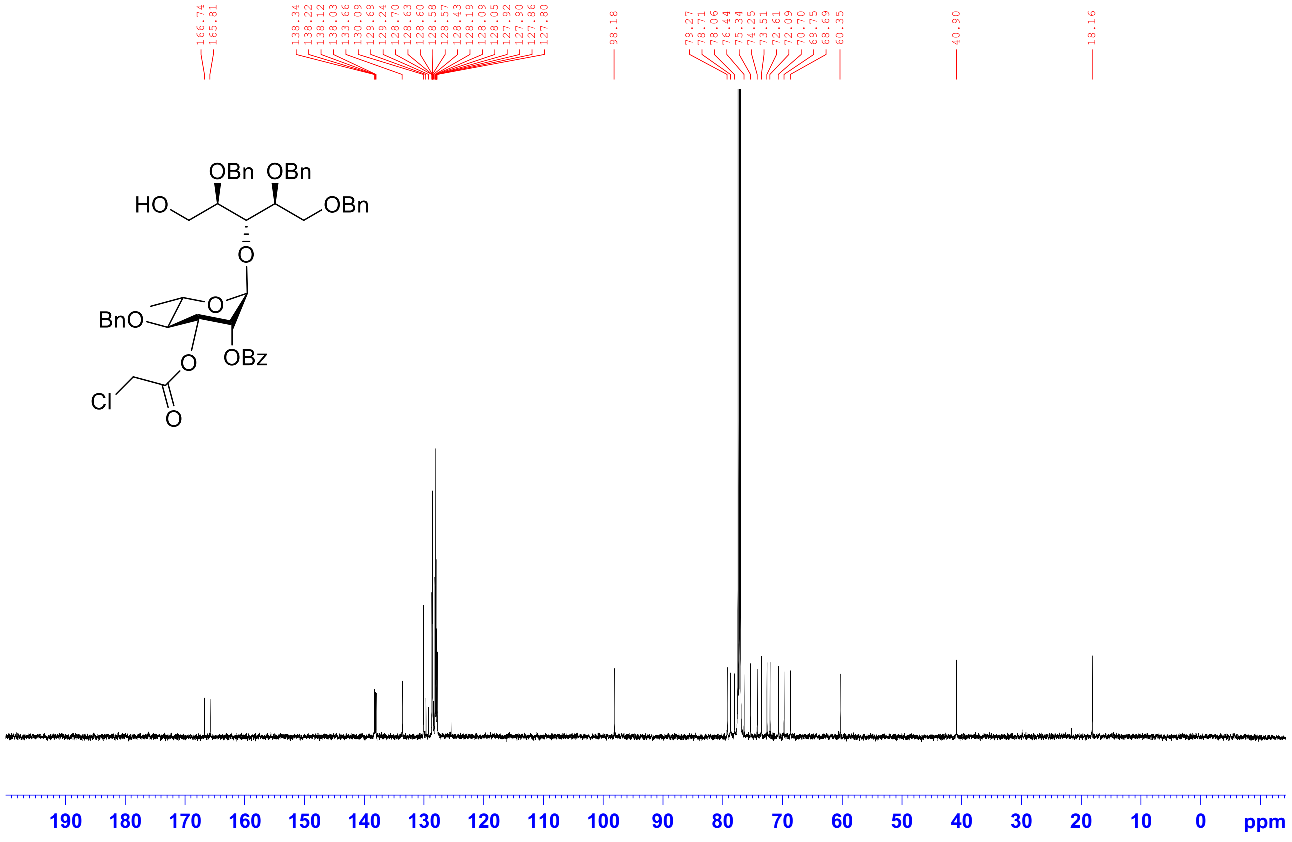

S16: DEPT 135 (150 MHz, $CDCl_3$)

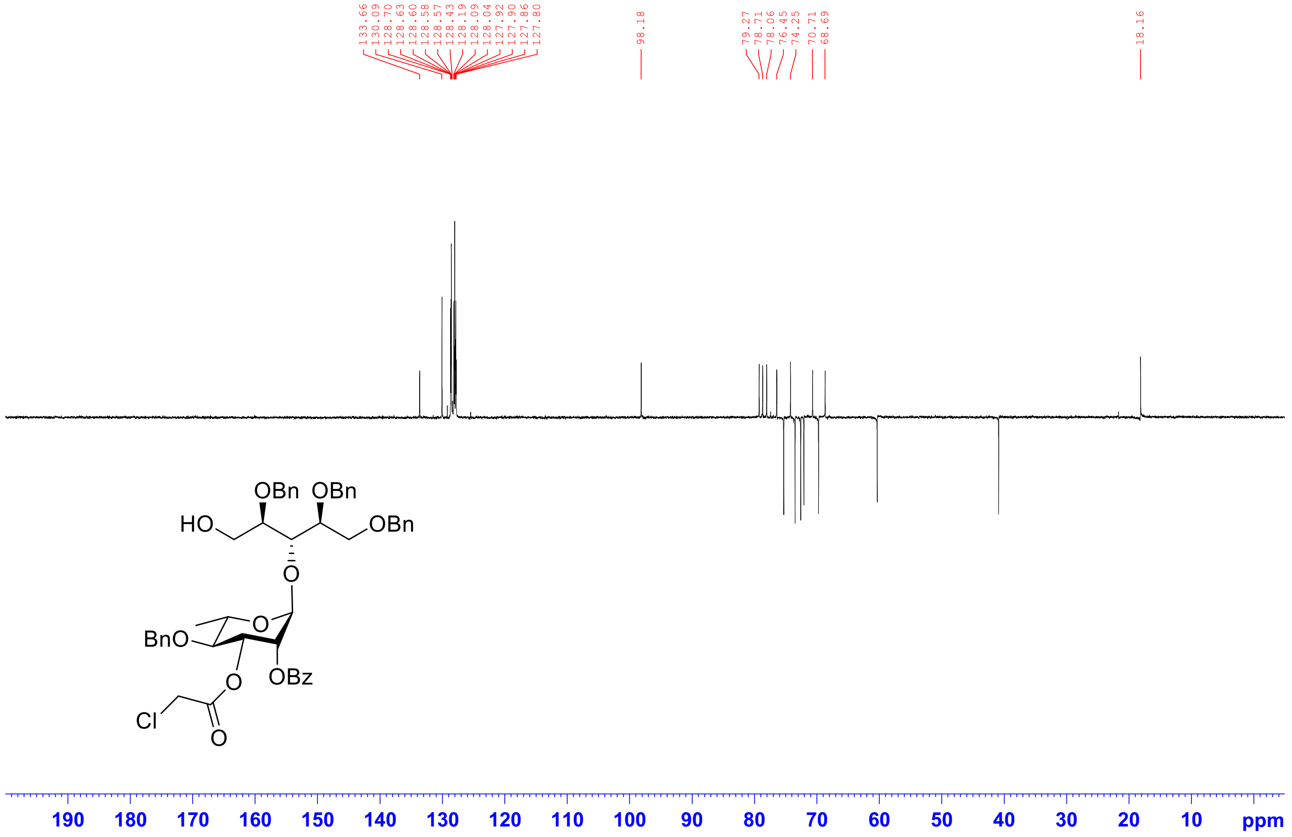

34: ^1H NMR (600 MHz, $CDCl_3$)

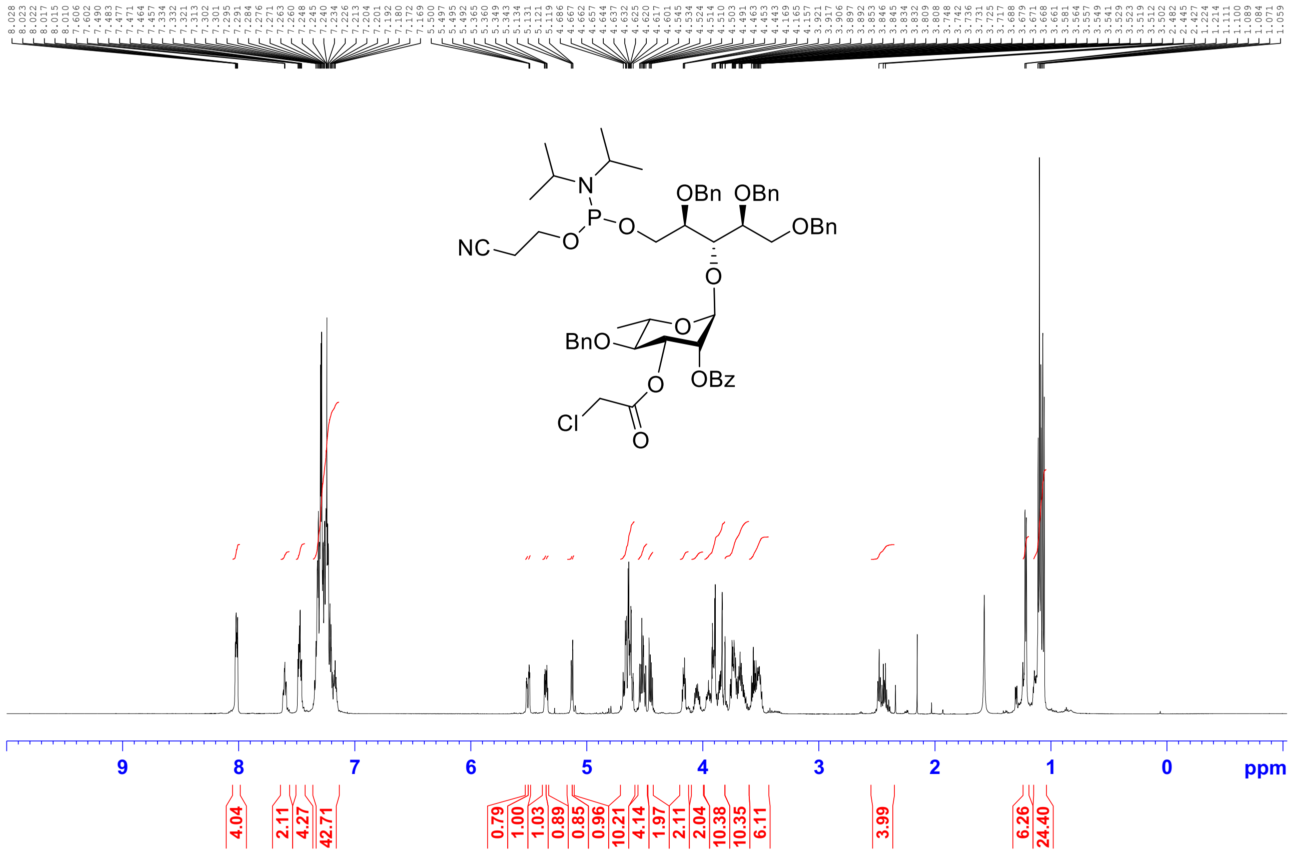

34: ^{13}C NMR (150 MHz, $CDCl_3$)

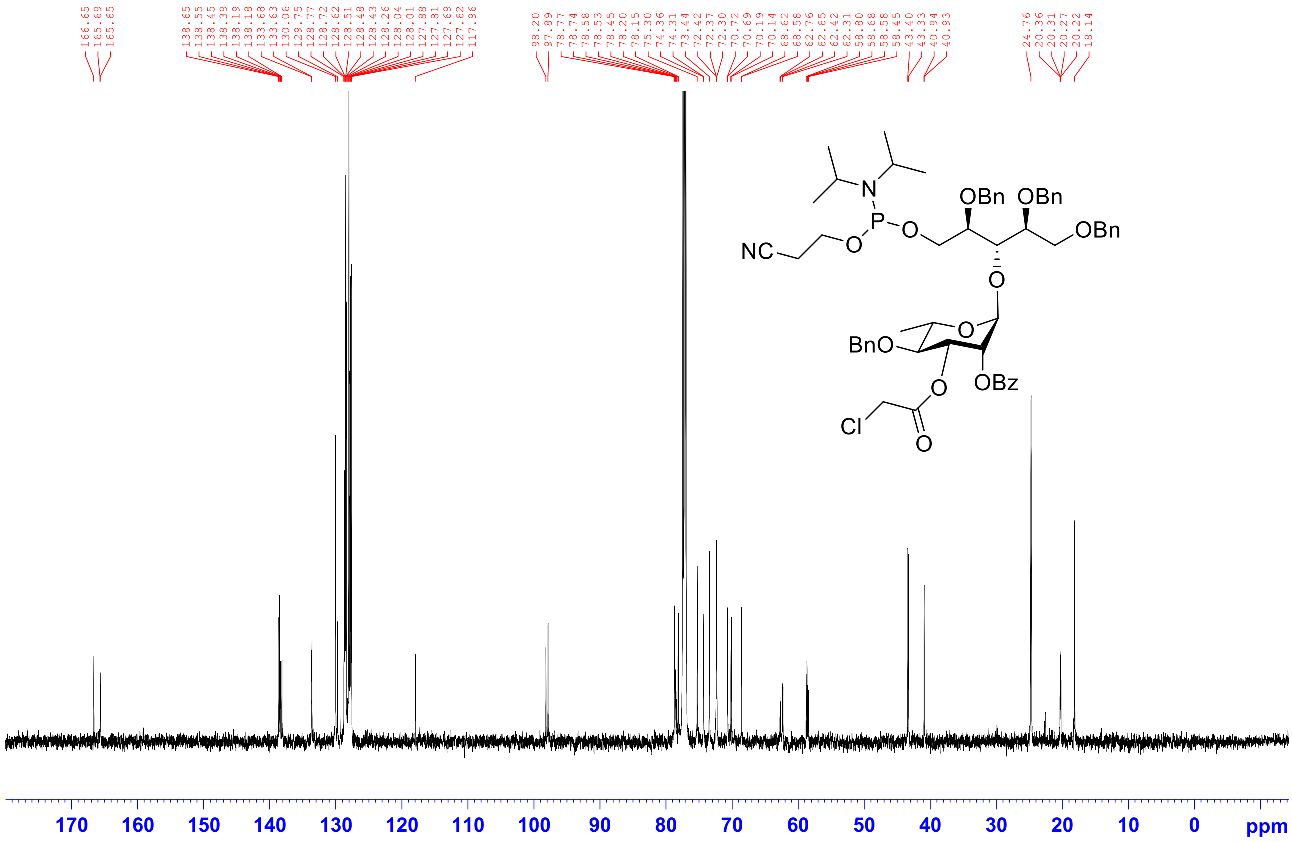

34: DEPT 135 (150 MHz, $CDCl_3$)

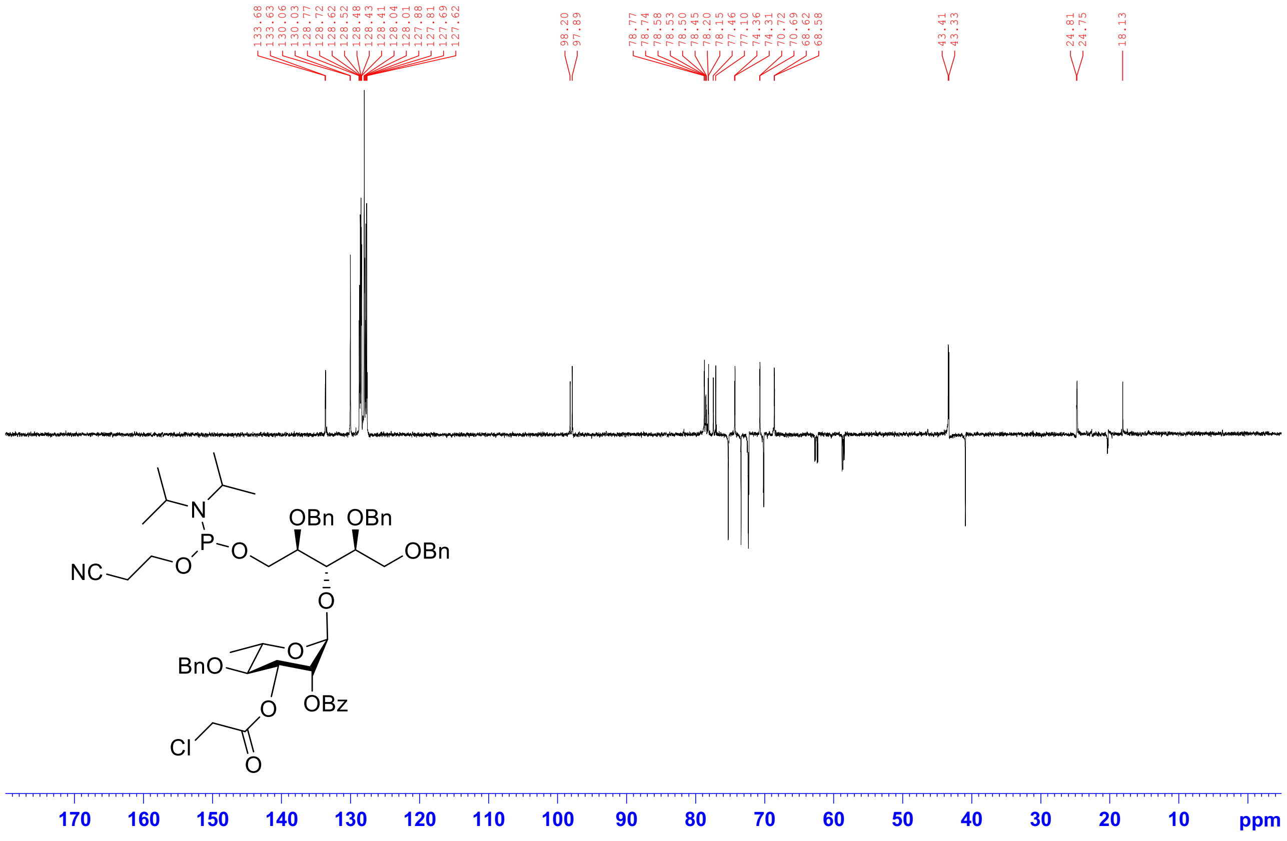

NMR spectra of trisaccharides

18: ^{1}H NMR (600 MHz, CDCl$_3$)

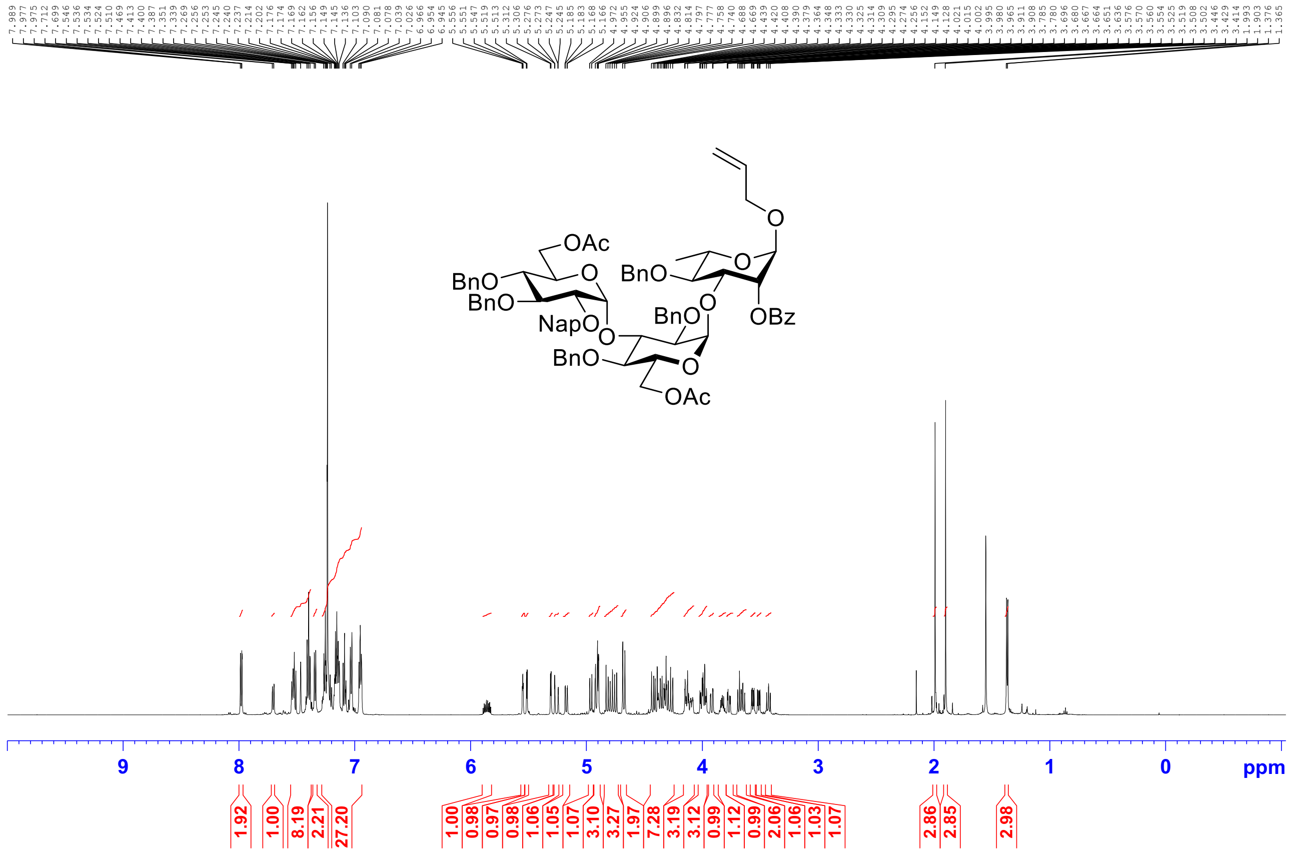

18: ^{13}C NMR (150 MHz, CDCl$_3$)

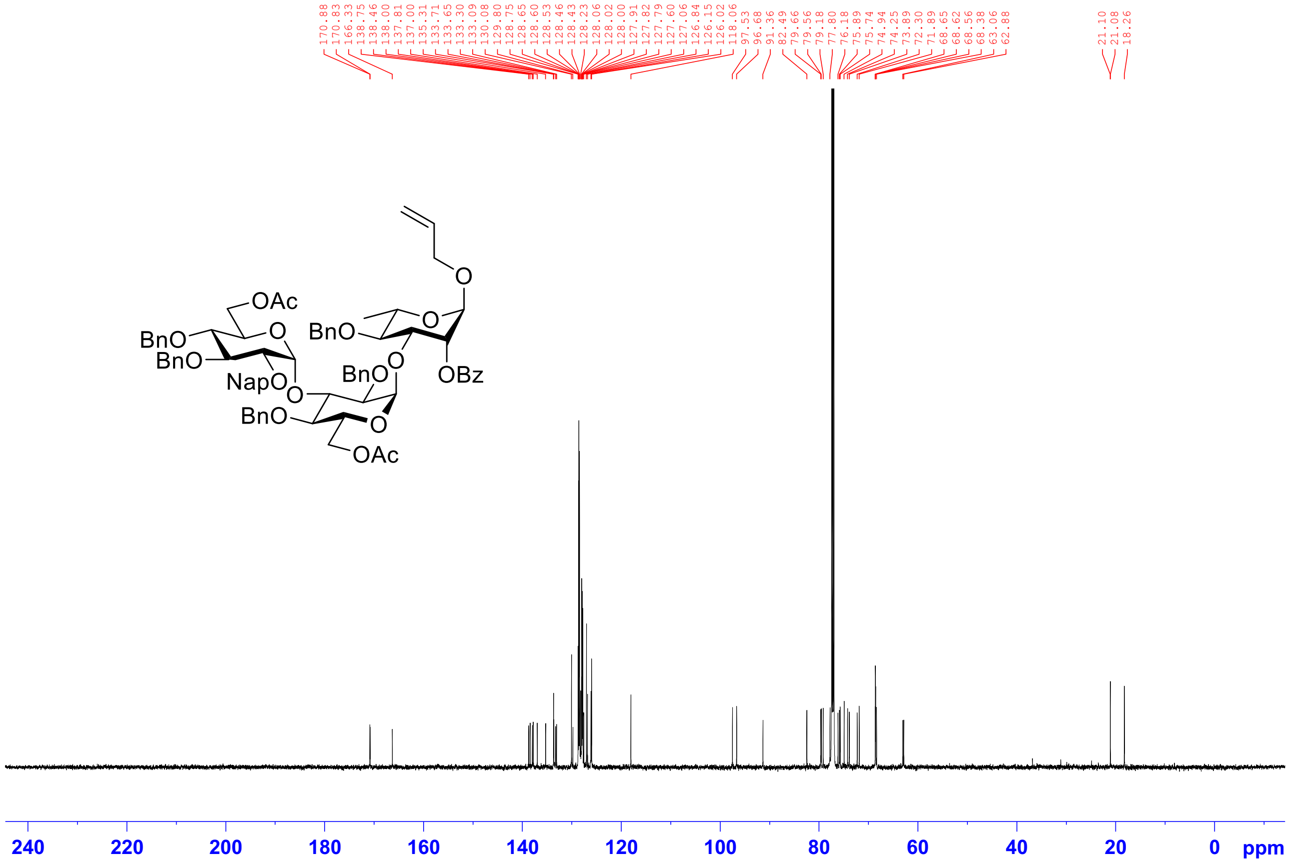

18: DEPT 135 (150 MHz, $CDCl_3$)

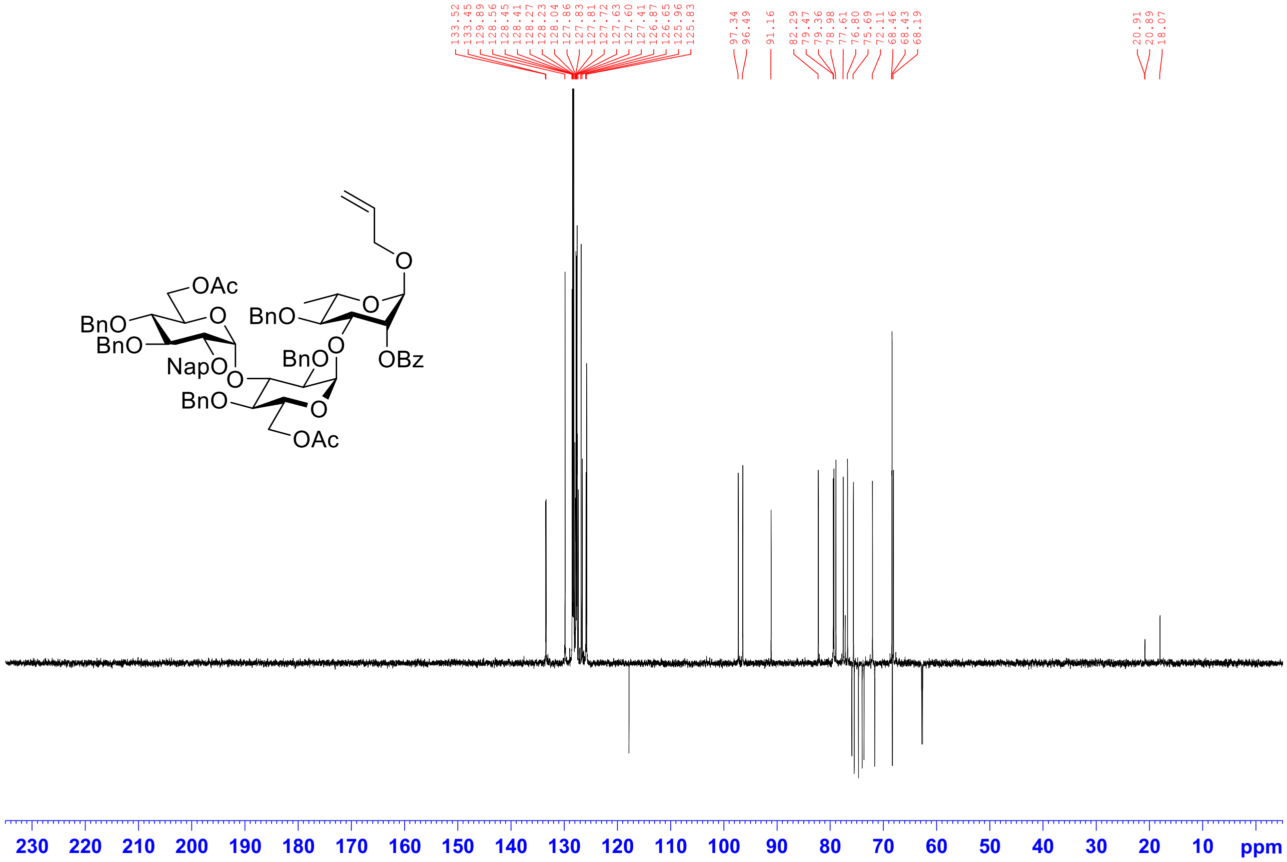

19: ^1H NMR (600 MHz, $CDCl_3$)

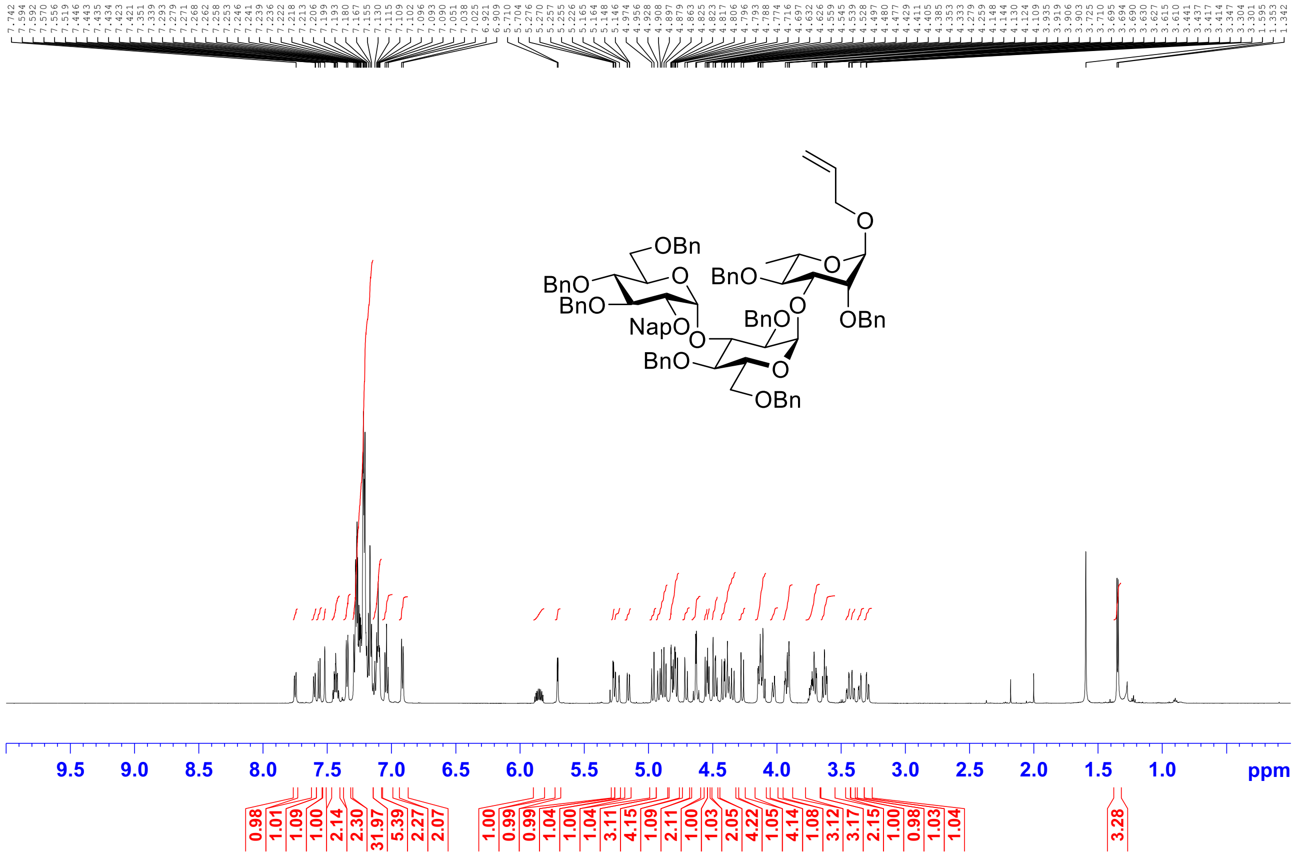

19: ^{13}C NMR (150 MHz, $CDCl_3$)

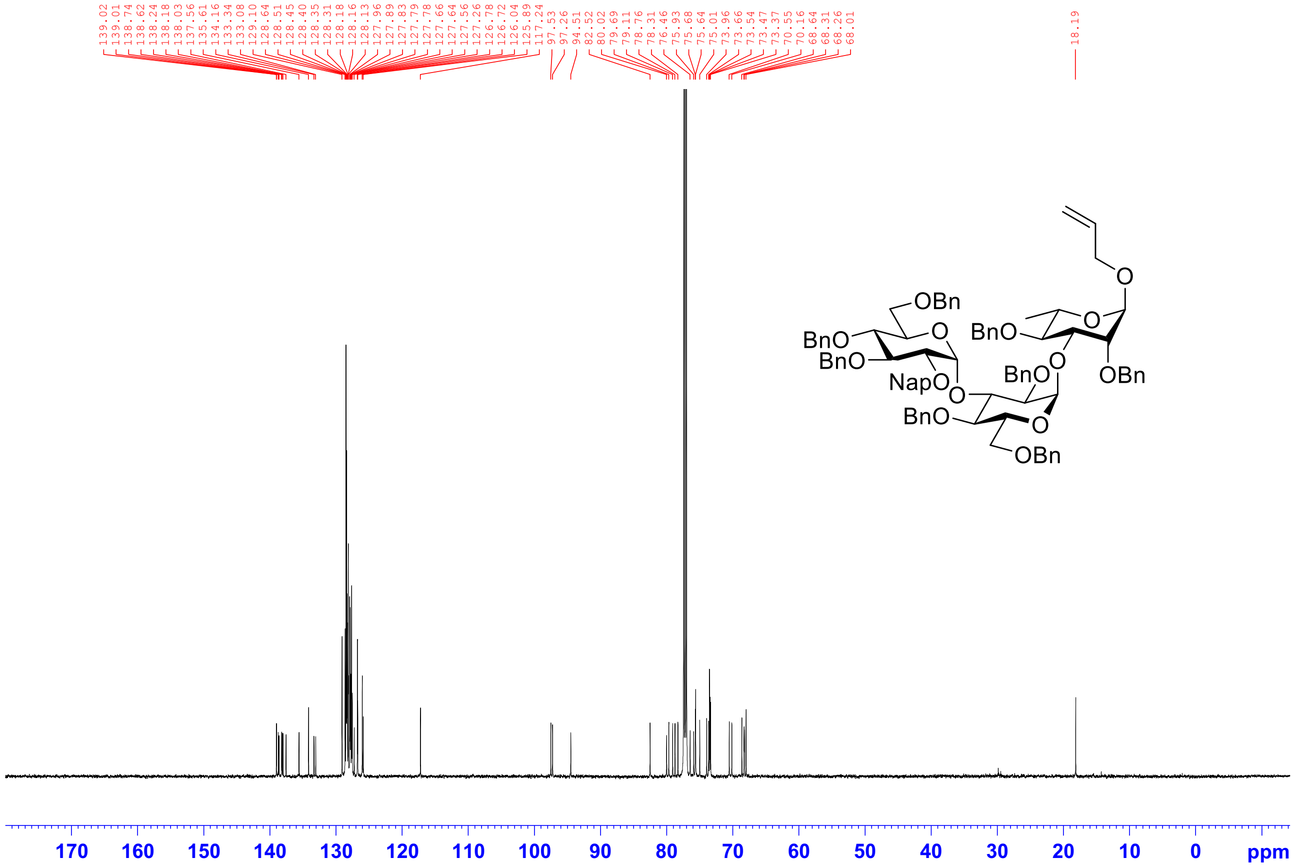

19: DEPT 135 (150 MHz, $CDCl_3$)

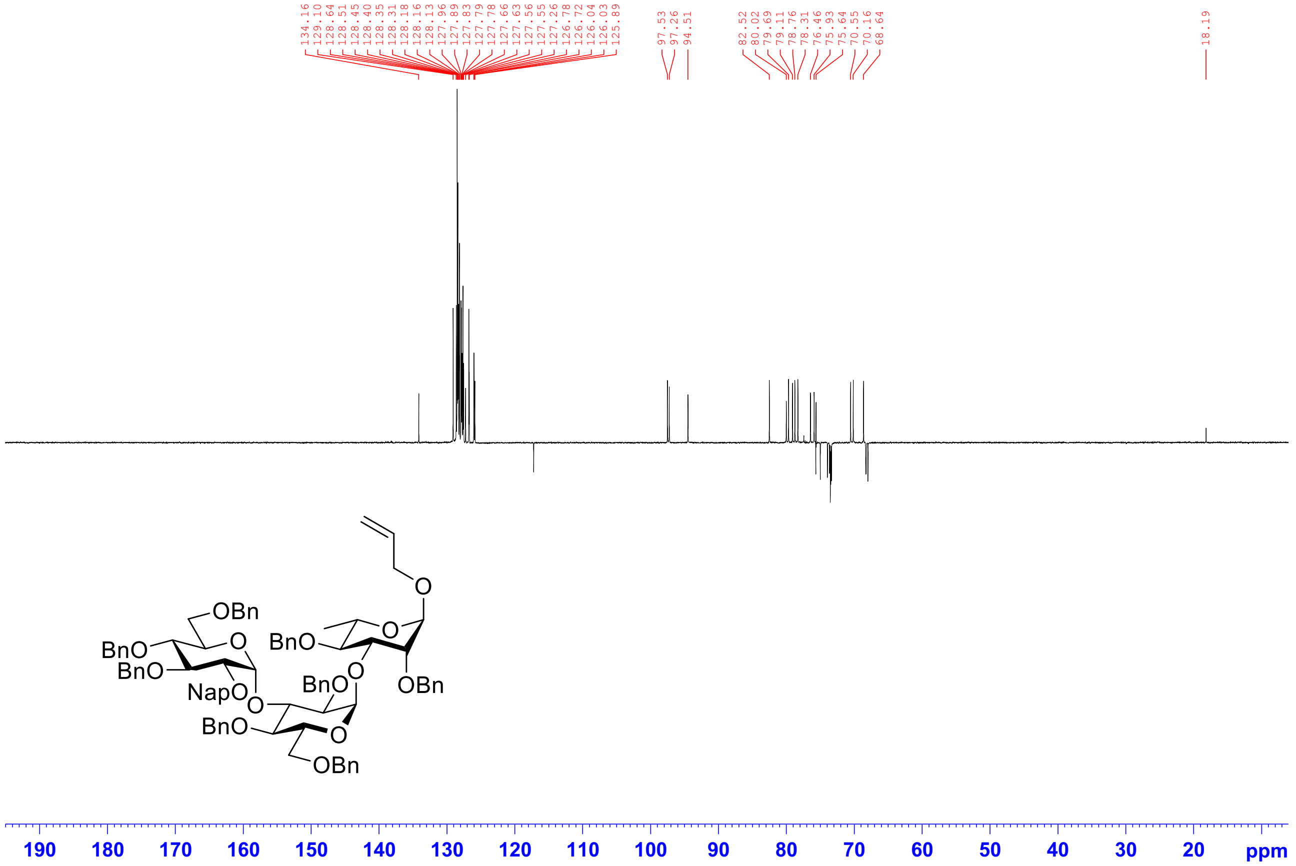

20: ^{1}H NMR (600 MHz, $CDCl_3$)

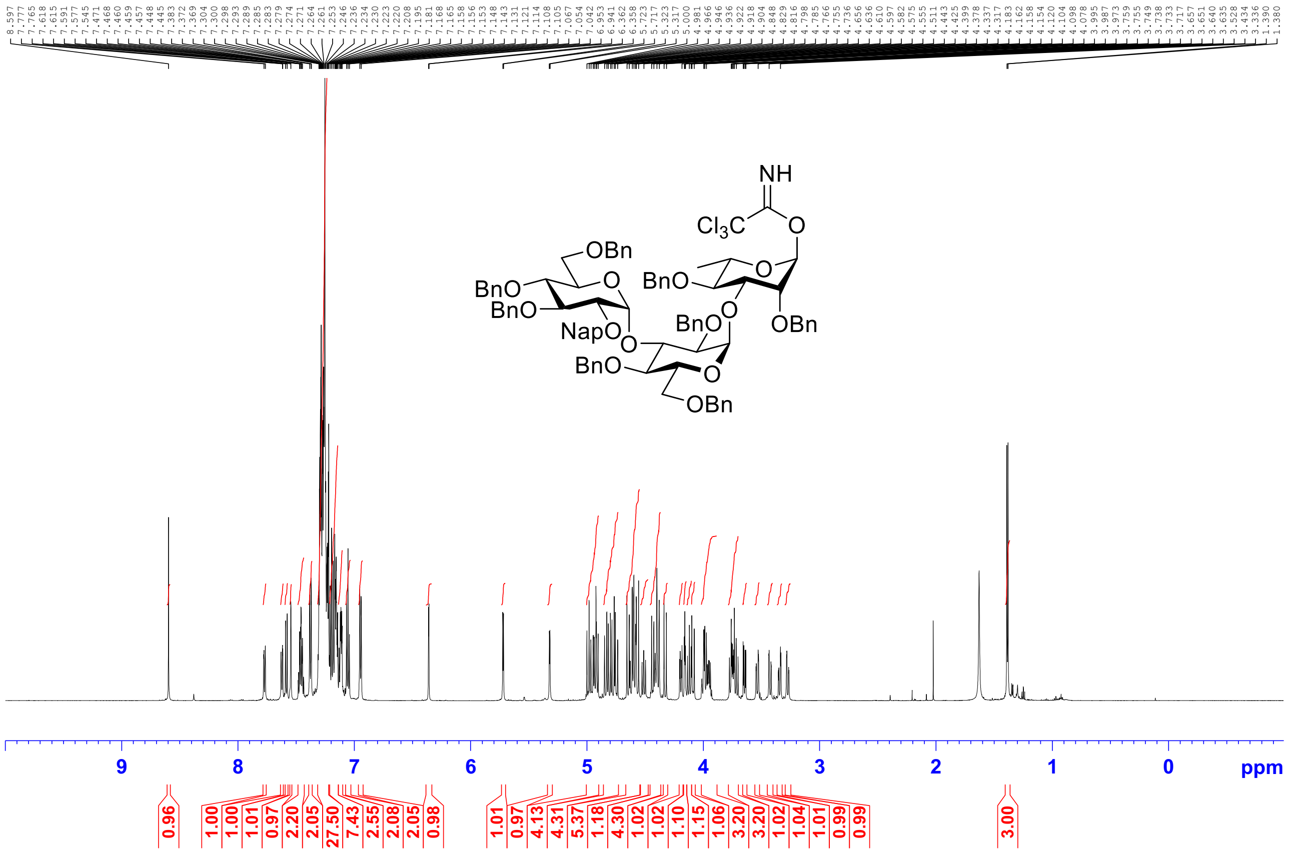

20: ^{13}C NMR (150 MHz, $CDCl_3$)

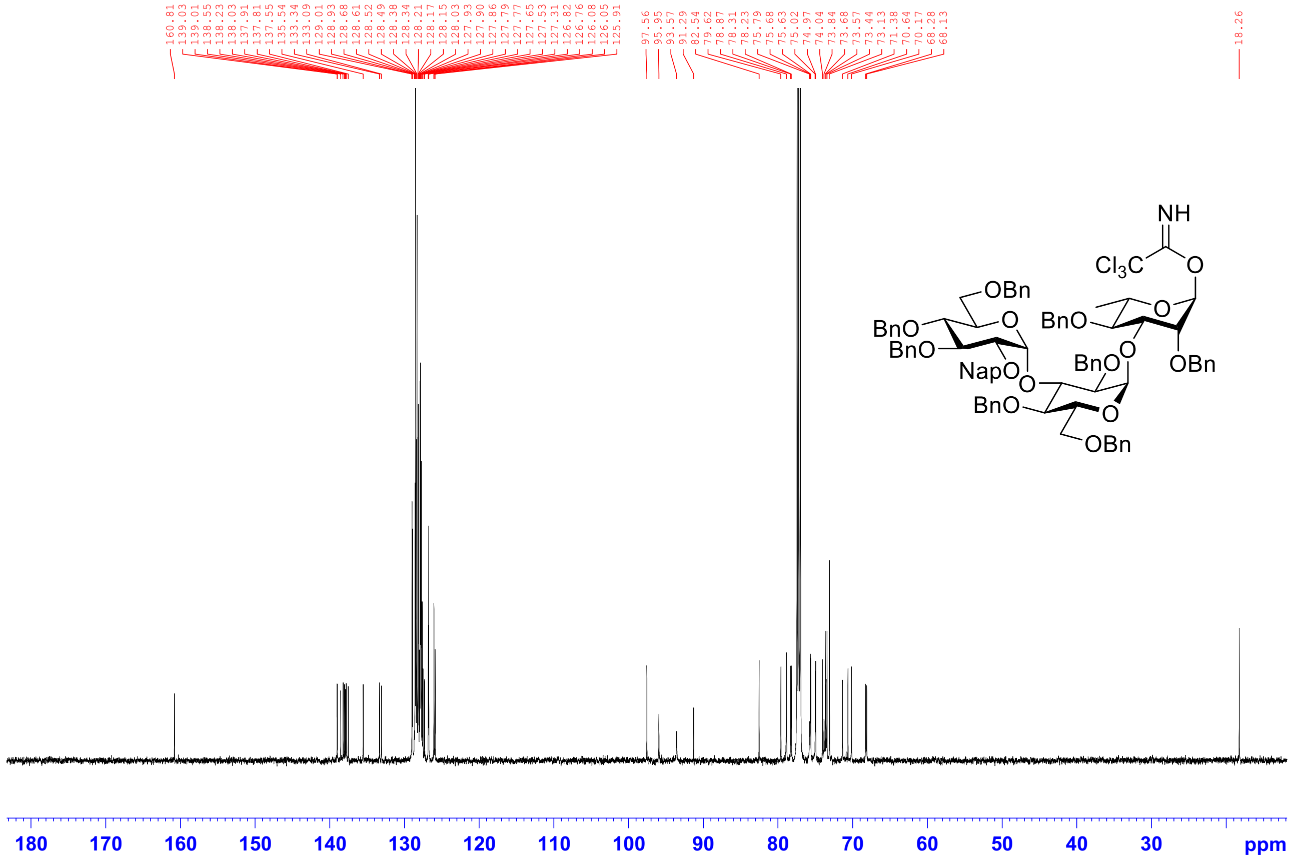

20: DEPT 135 (150 MHz, $CDCl_3$)

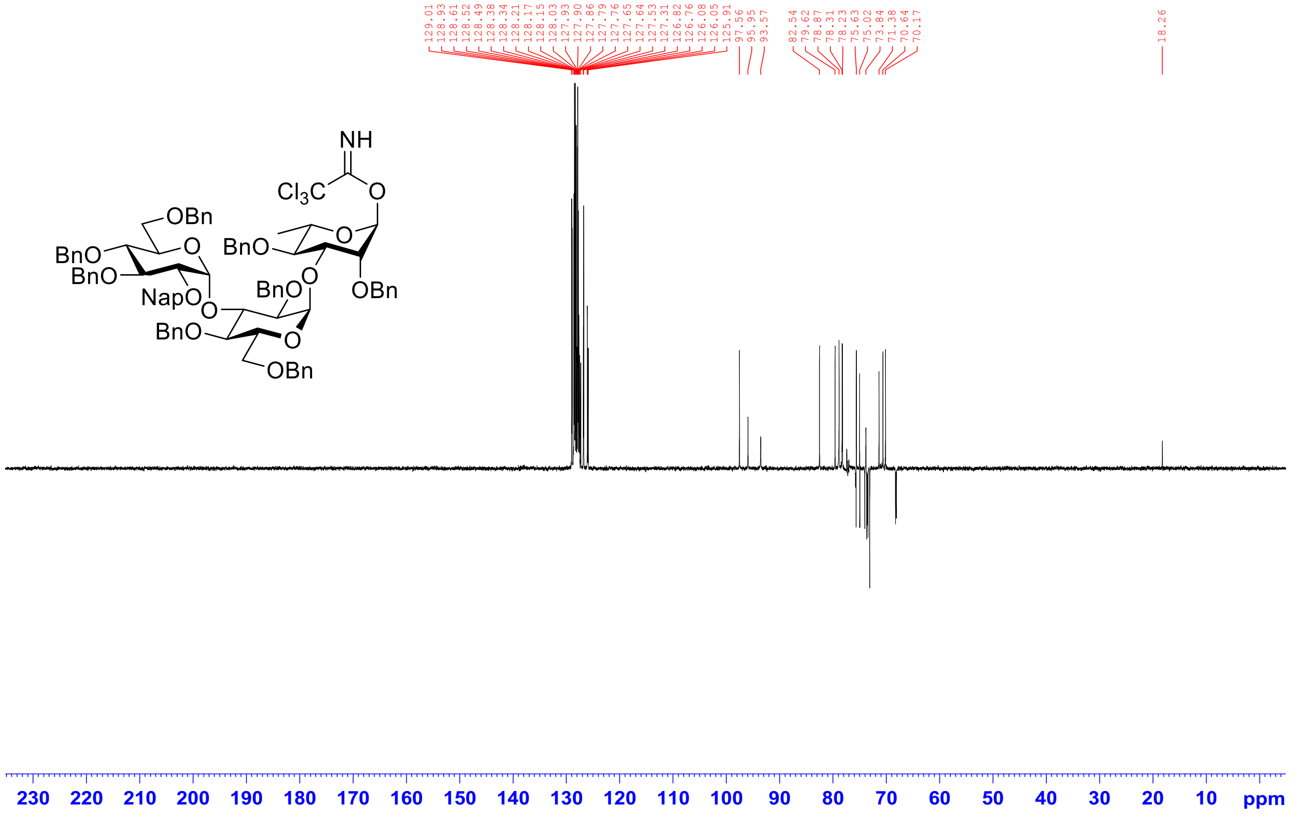

NMR spectra of pseudo-tetrasaccharides for ST6C

S19: ^{1}H NMR (600 MHz, $CDCl_3$)

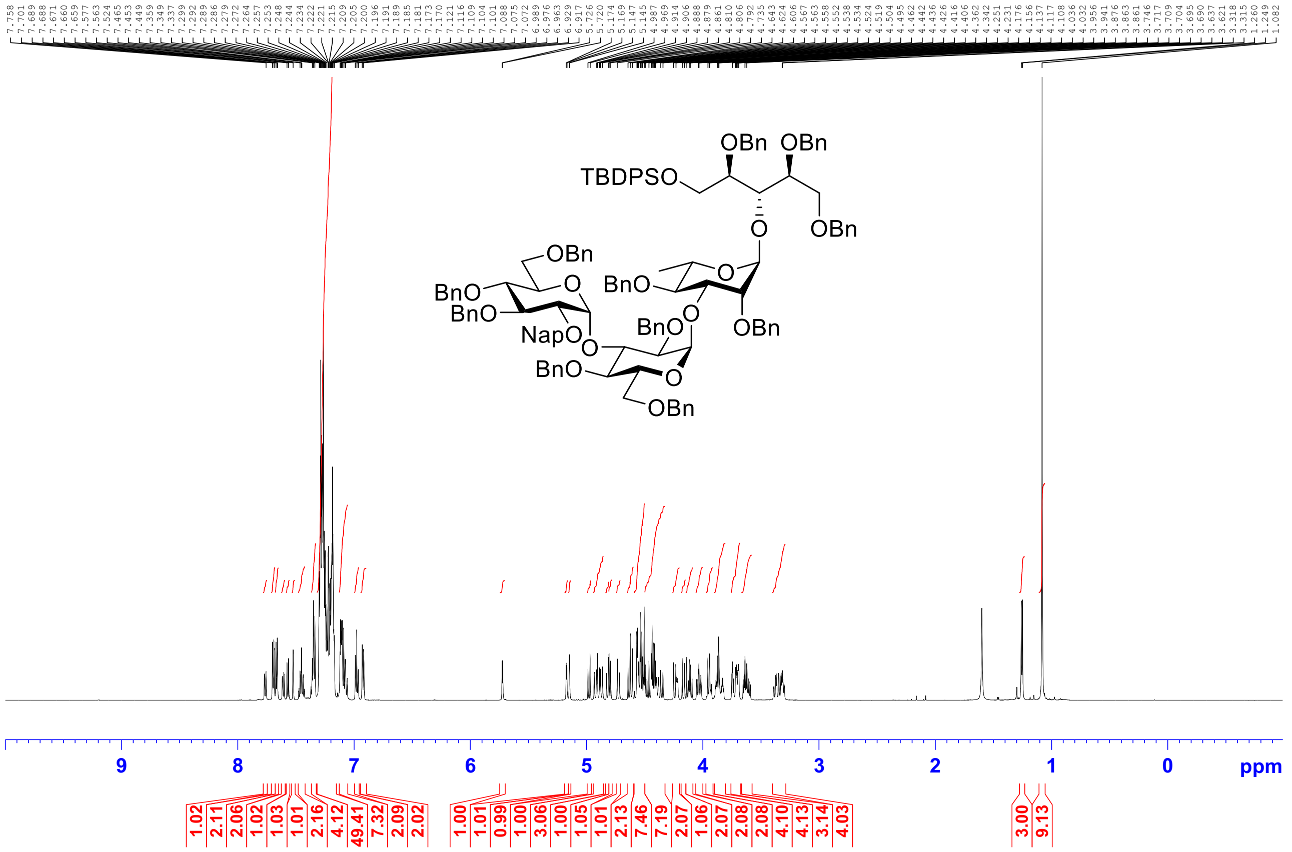

S19: ^{13}C NMR (150 MHz, CDCl$_3$)

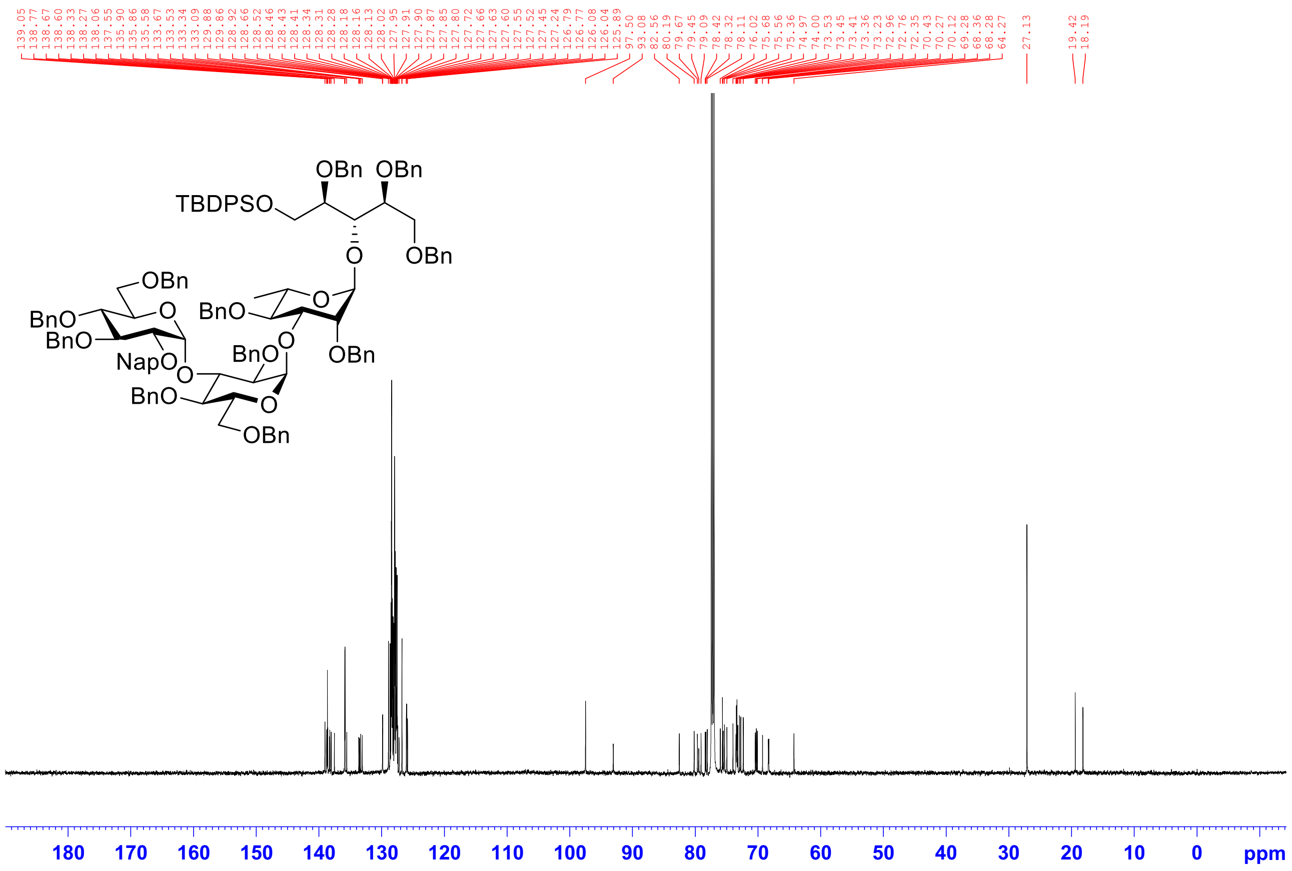

S19: DEPT 135 (150 MHz, CDCl$_3$)

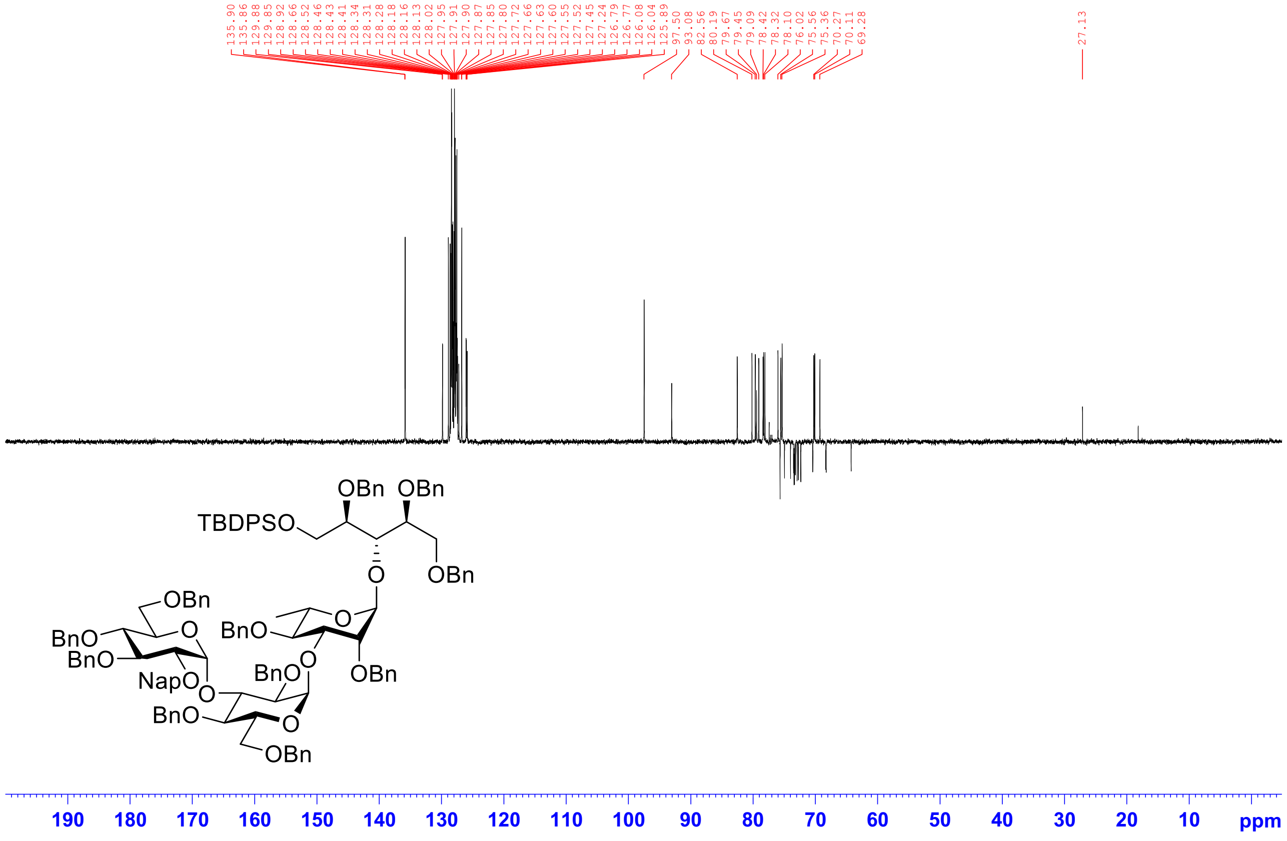

23: ^{1}H NMR (600 MHz, $CDCl_3$)

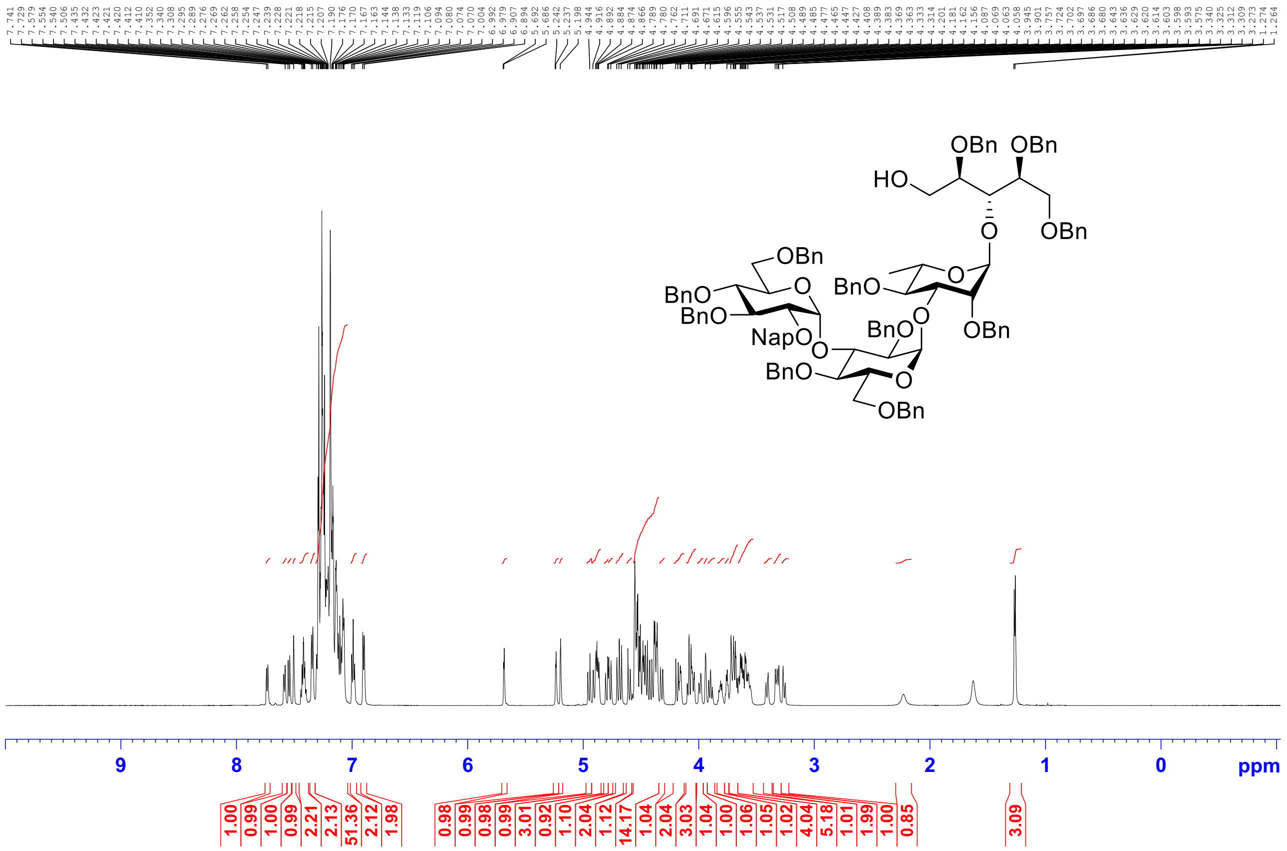

23: ^{13}C NMR (150 MHz, $CDCl_3$)

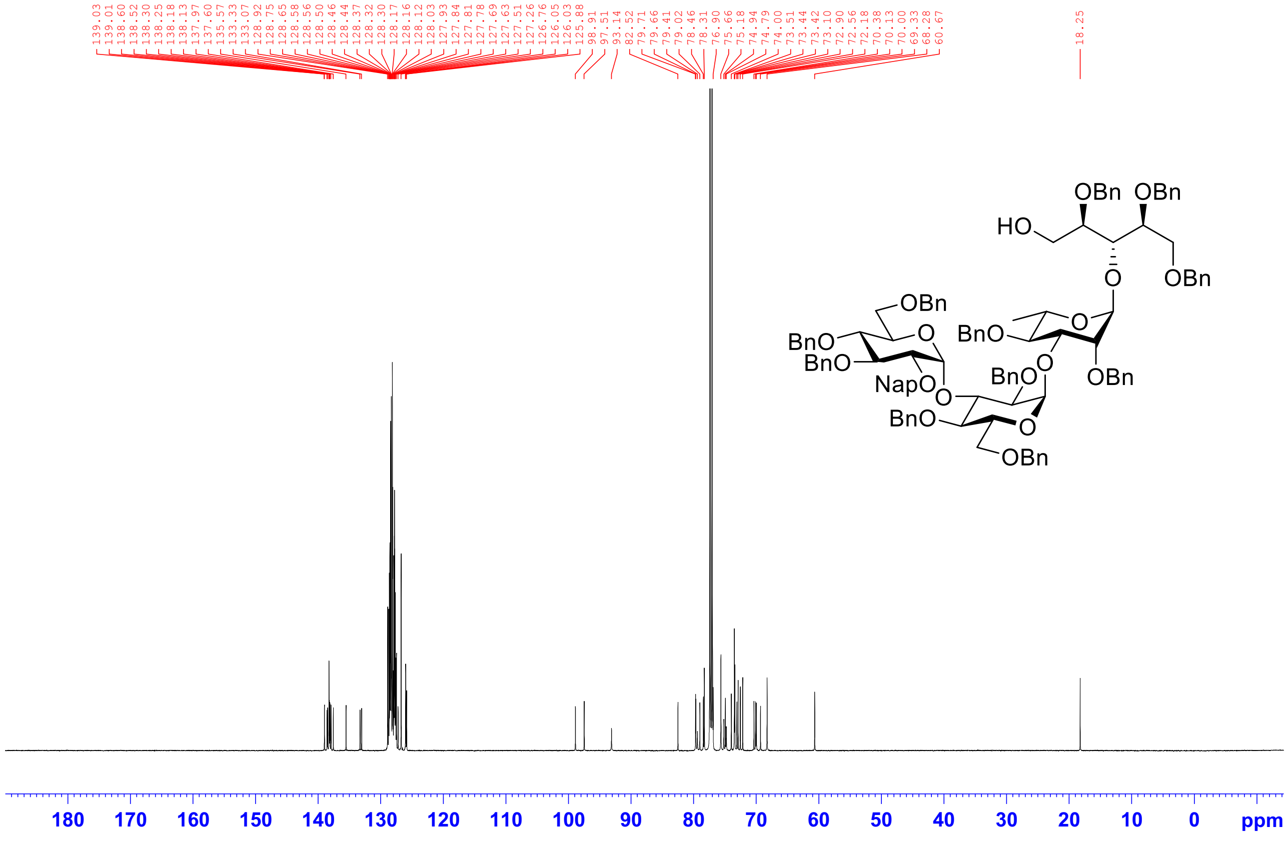

23: DEPT 135 (150 MHz, $CDCl_3$)

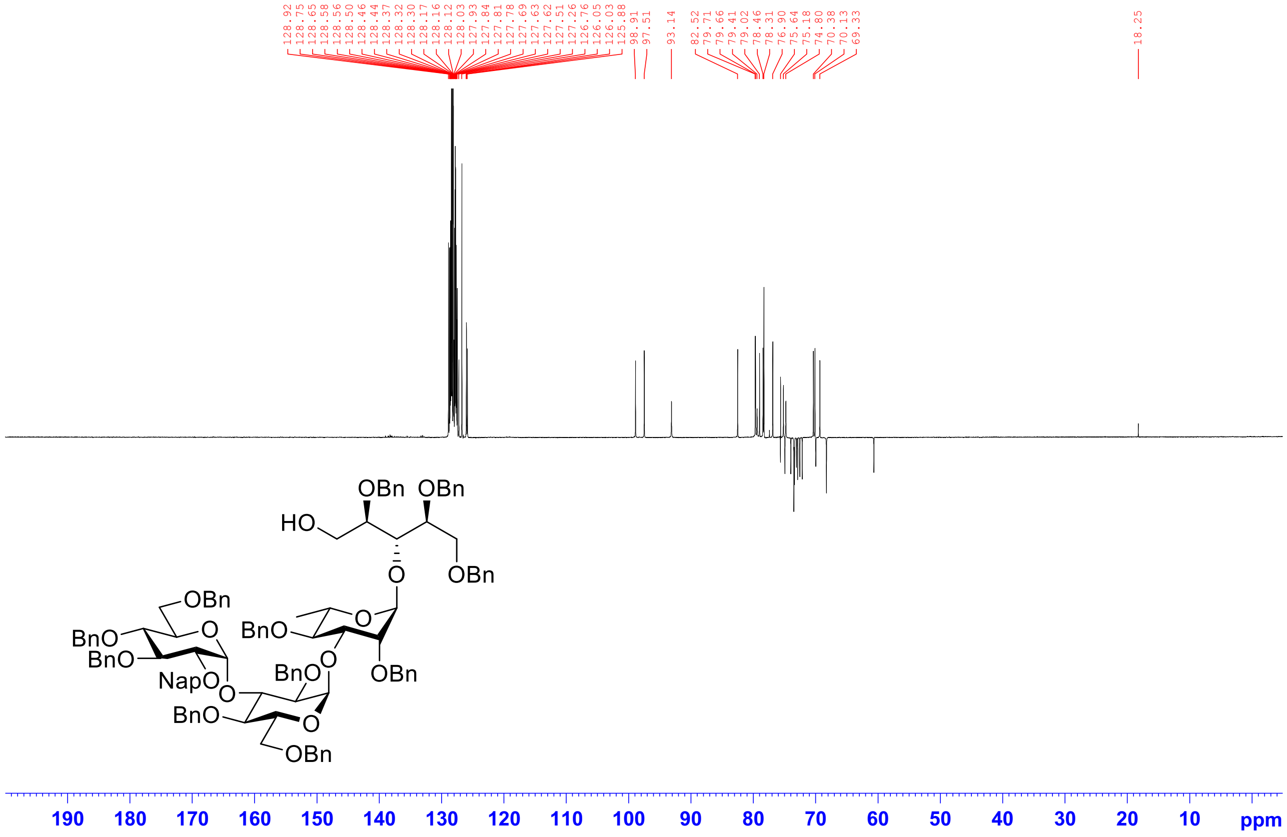

25: ^{1}H NMR (600 MHz, $CDCl_3$)

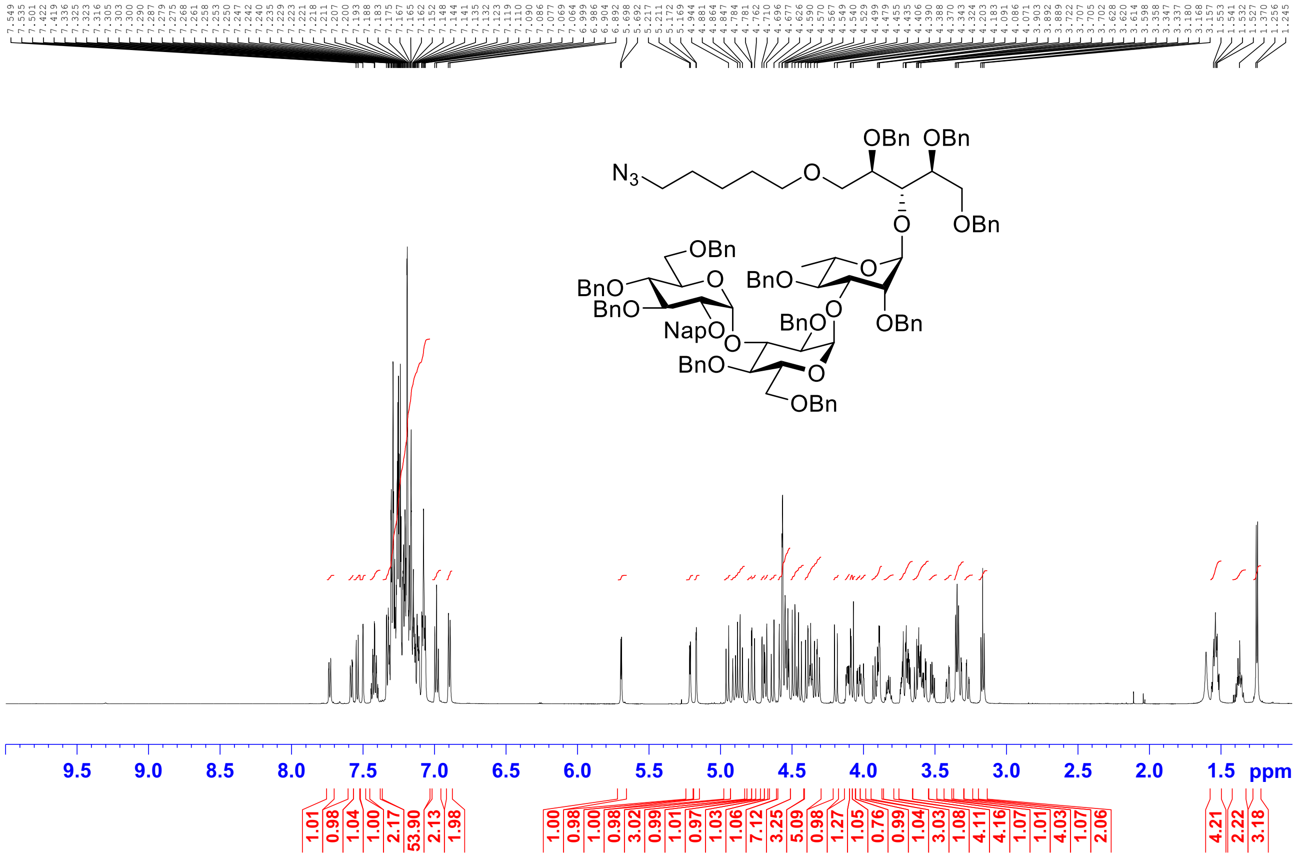

25: ^{13}C NMR (150 MHz, CDCl$_3$)

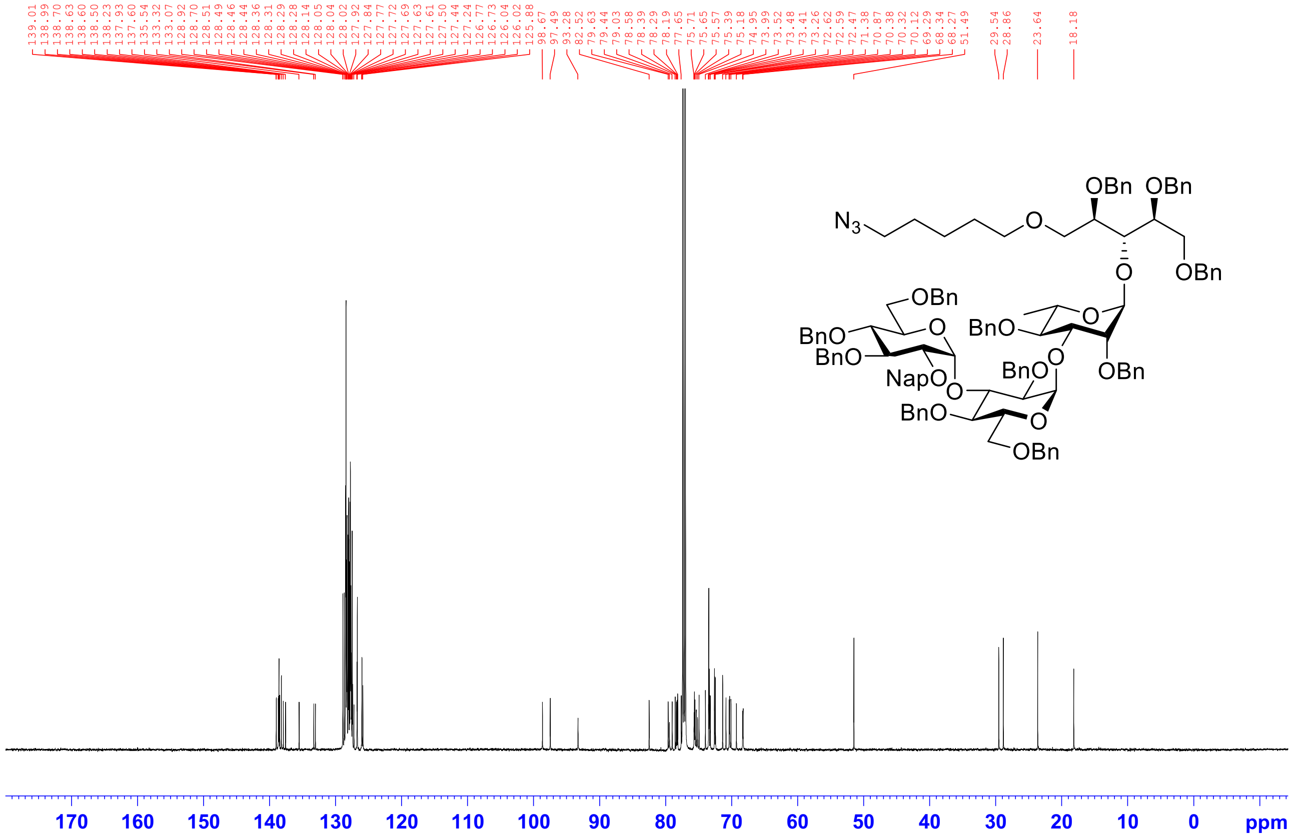

25: DEPT 135 (150 MHz, CDCl$_3$)

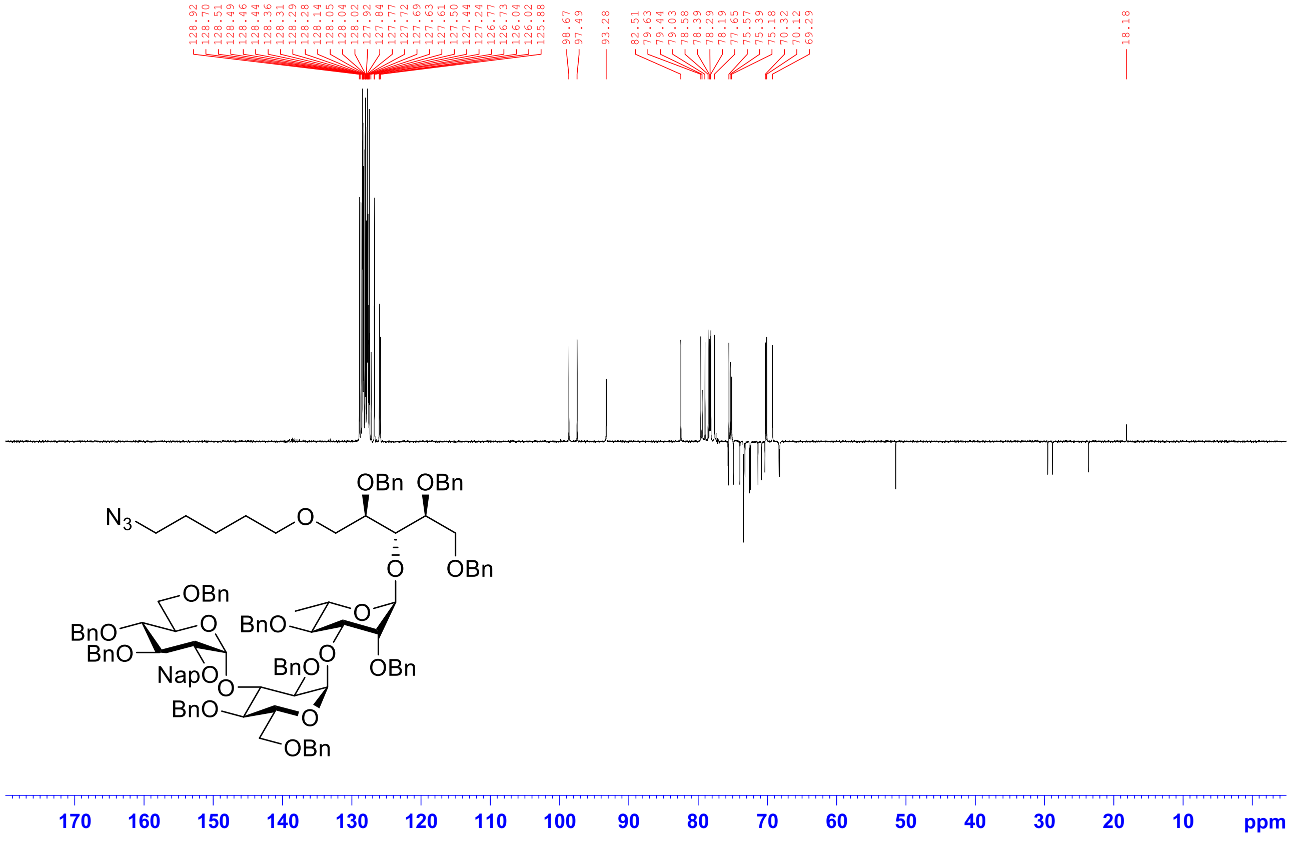

26: ^{1}H NMR (600 MHz, CDCl$_3$)

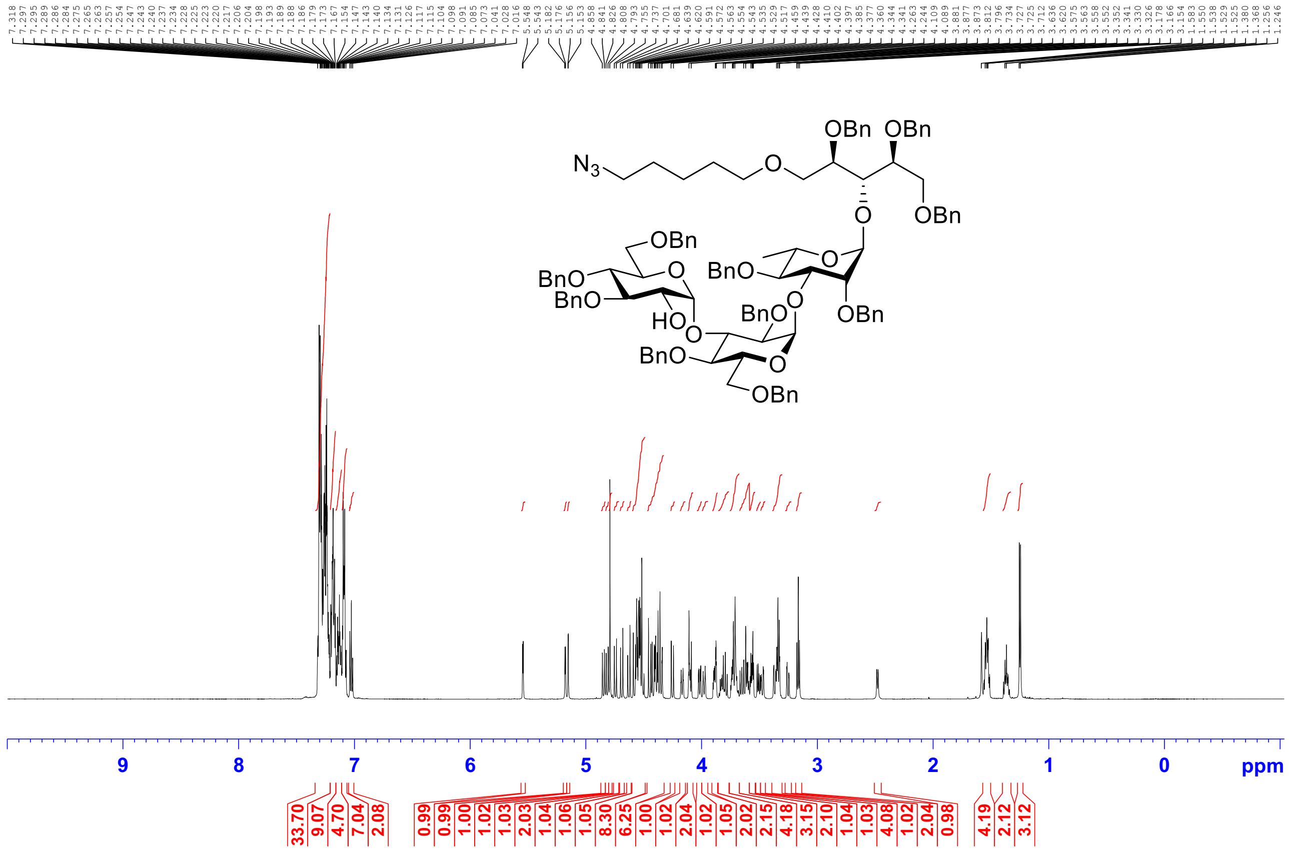

26: ^{13}C NMR (150 MHz, CDCl$_3$)

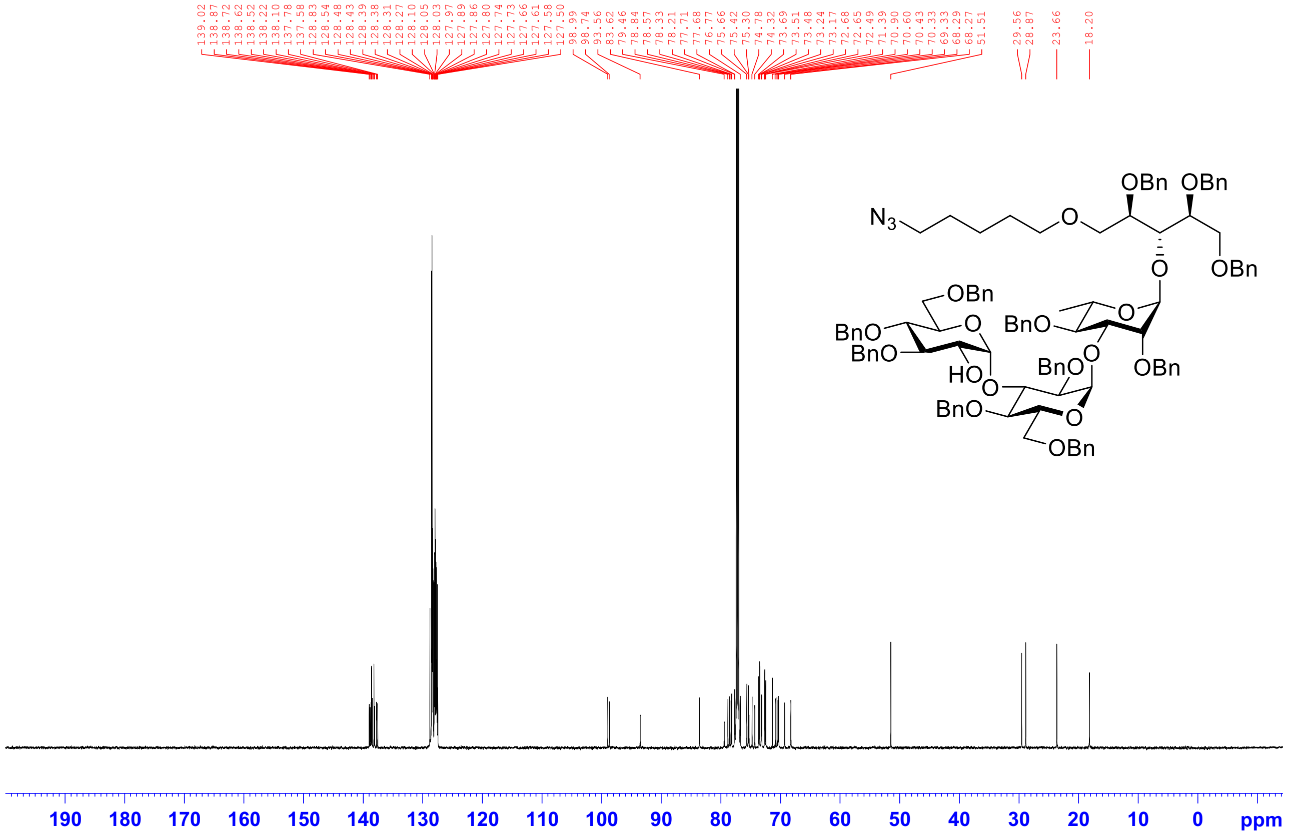

26: DEPT 135 (150 MHz, $CDCl_3$)

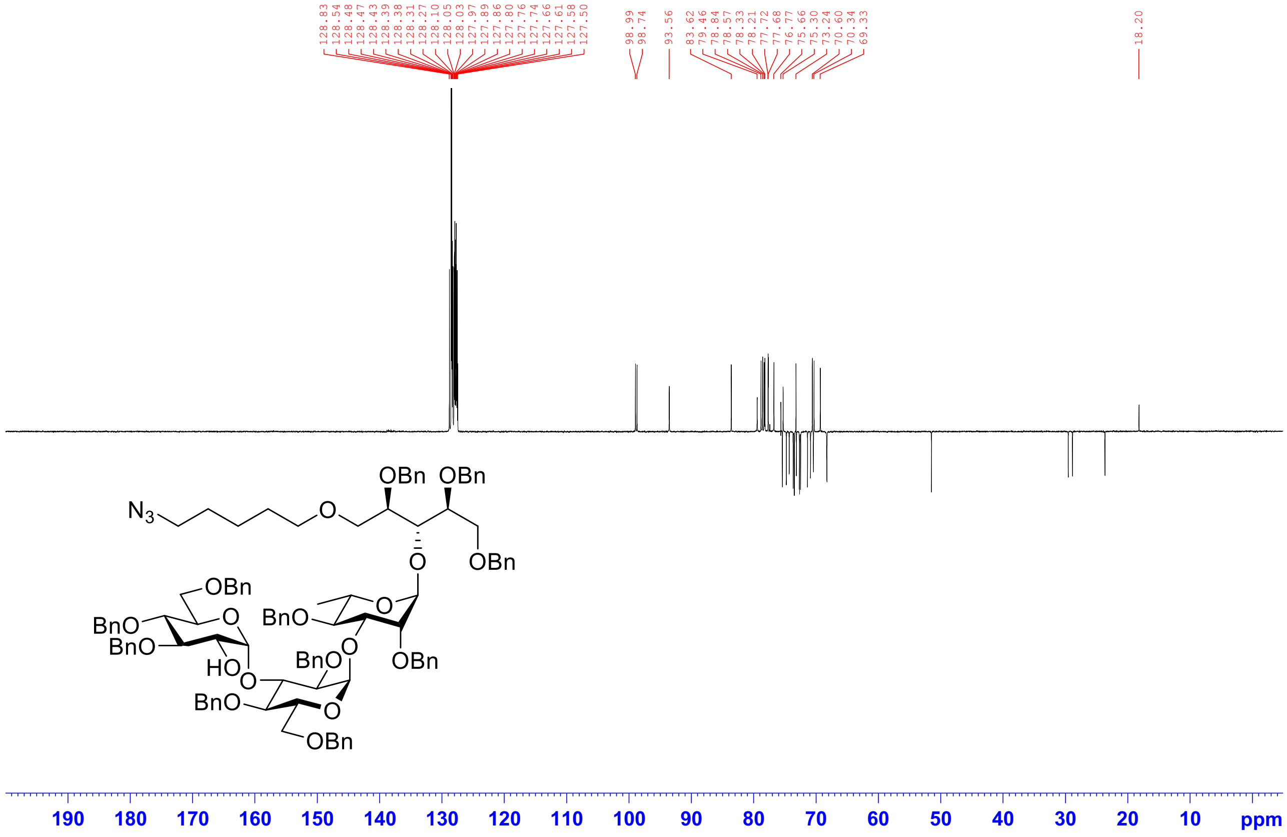

29: ^1H NMR (600 MHz, $CDCl_3$)

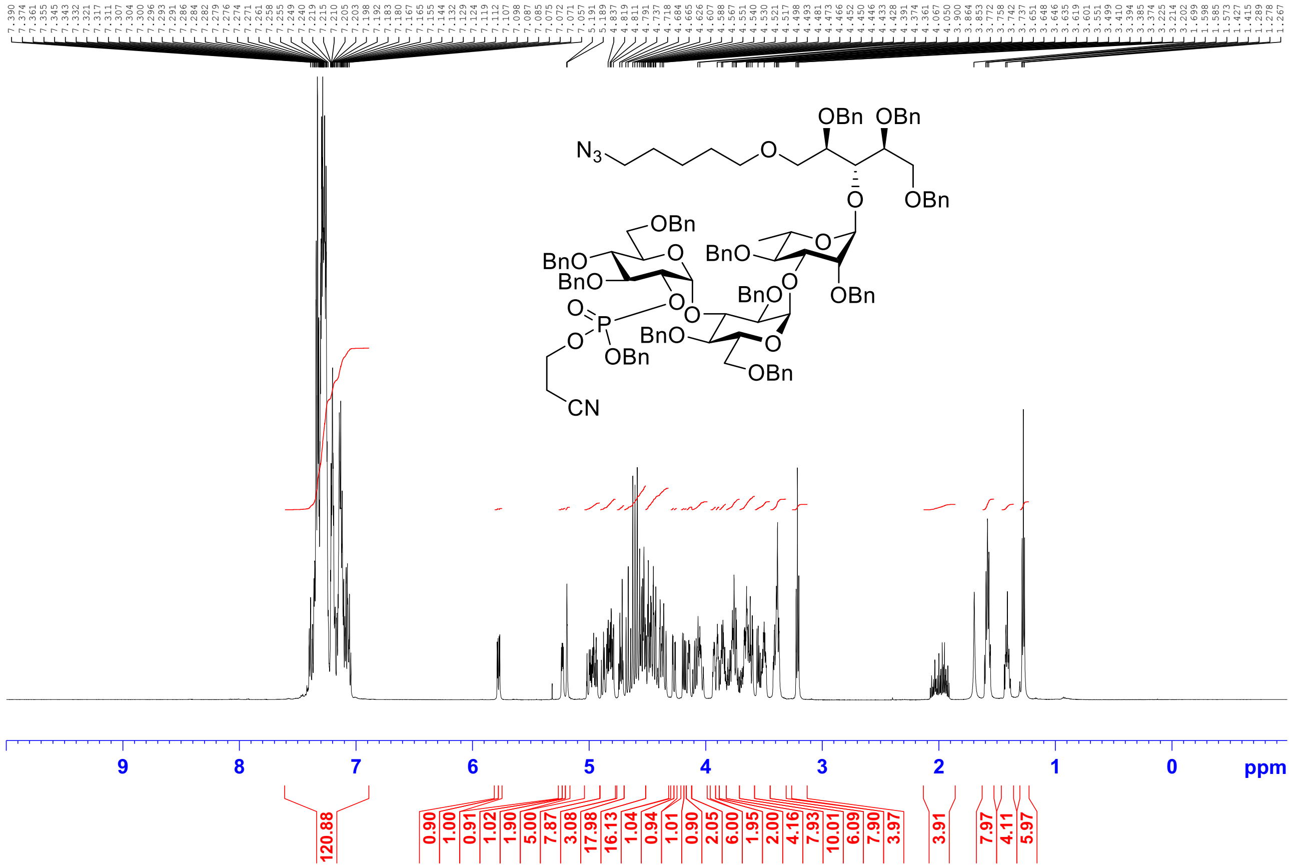

29: ^{13}C NMR (150 MHz, CDCl$_3$)

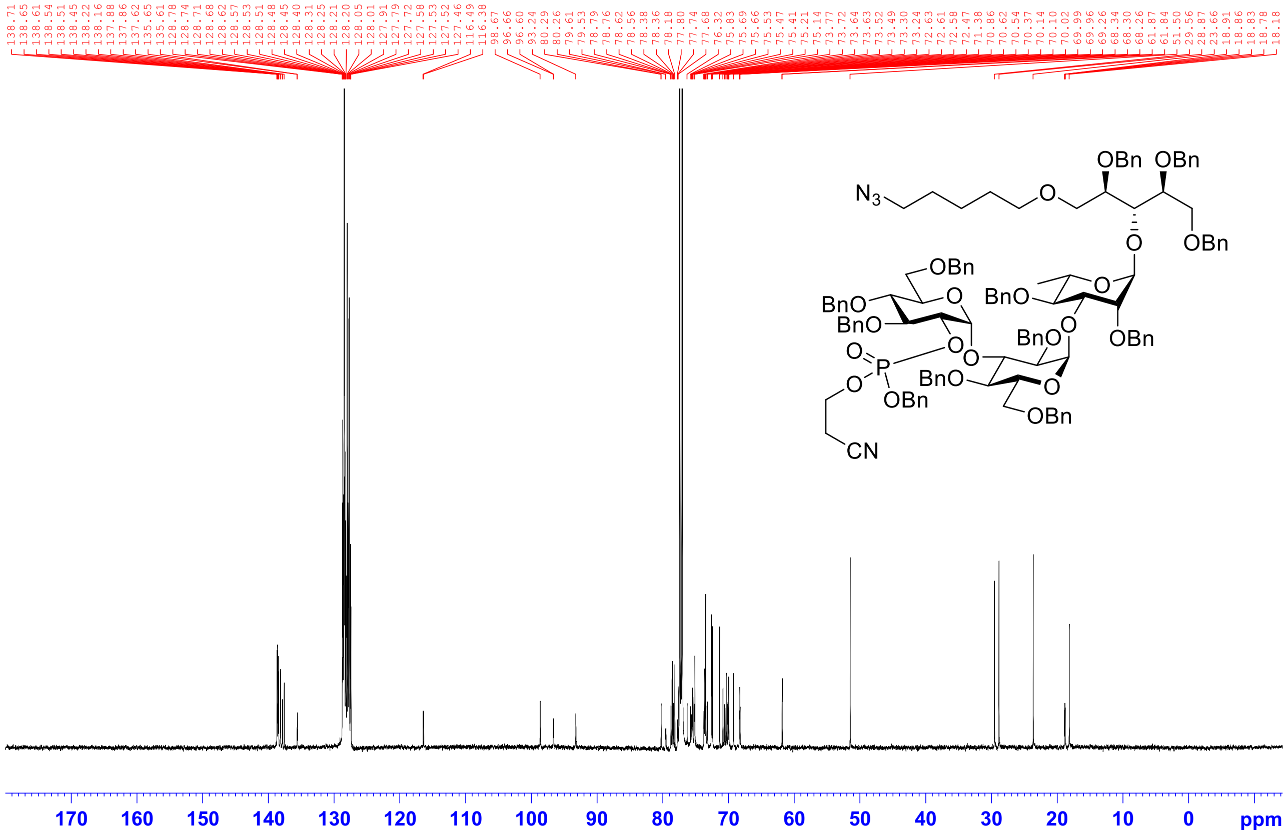

29: DEPT 135 (150 MHz, CDCl$_3$)

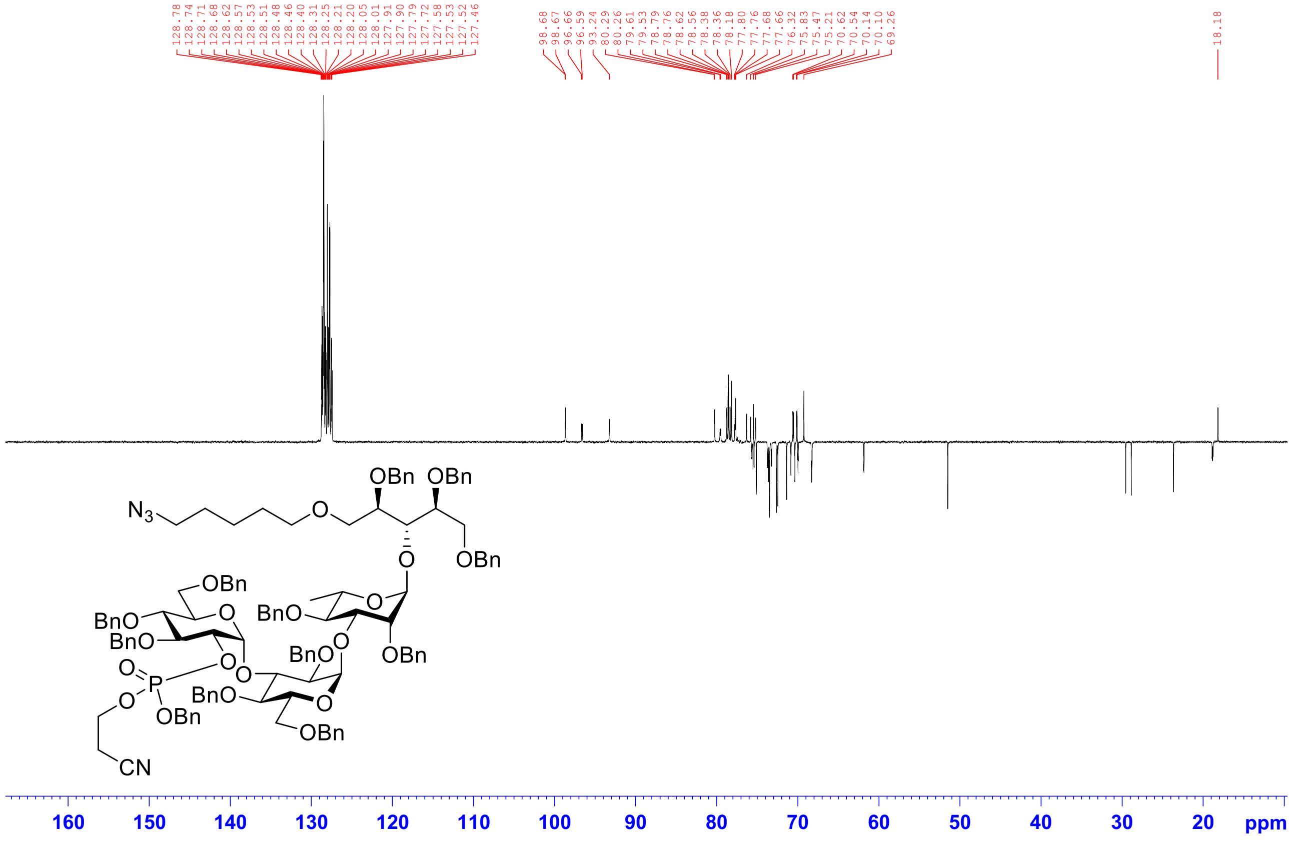

29: ^{31}P NMR (202 MHz, $CDCl_3$)

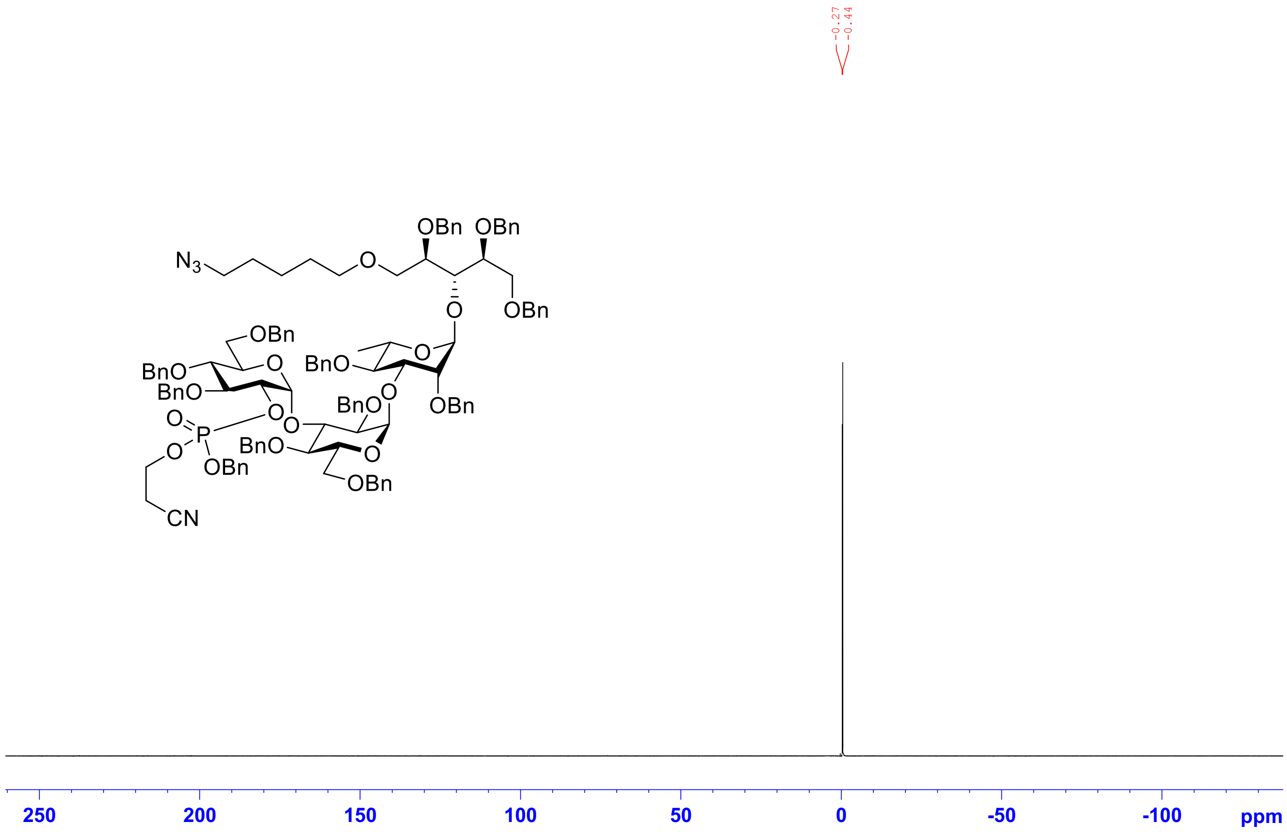

S20: ^{1}H NMR (600 MHz, $CDCl_3$)

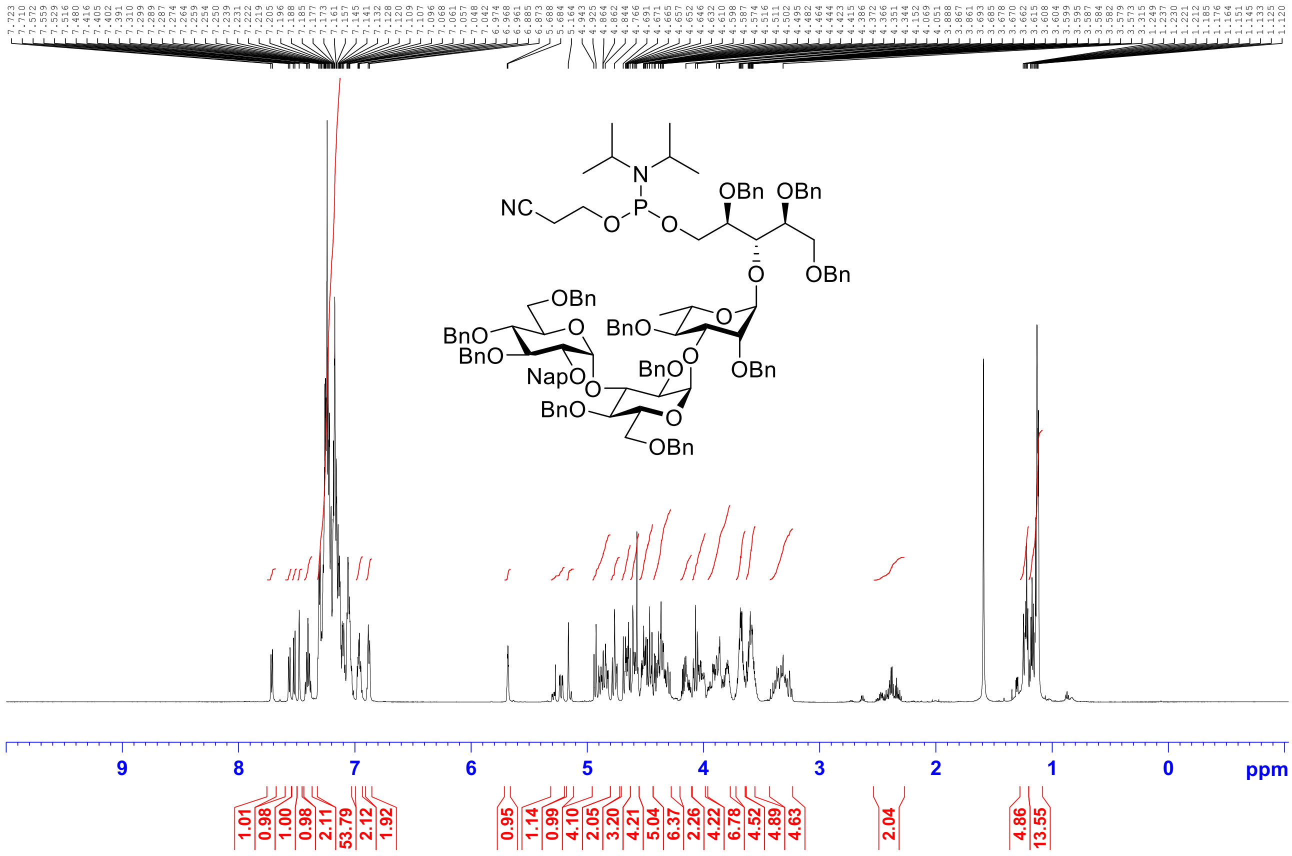

S20: ^{13}C NMR (150 MHz, $CDCl_3$)

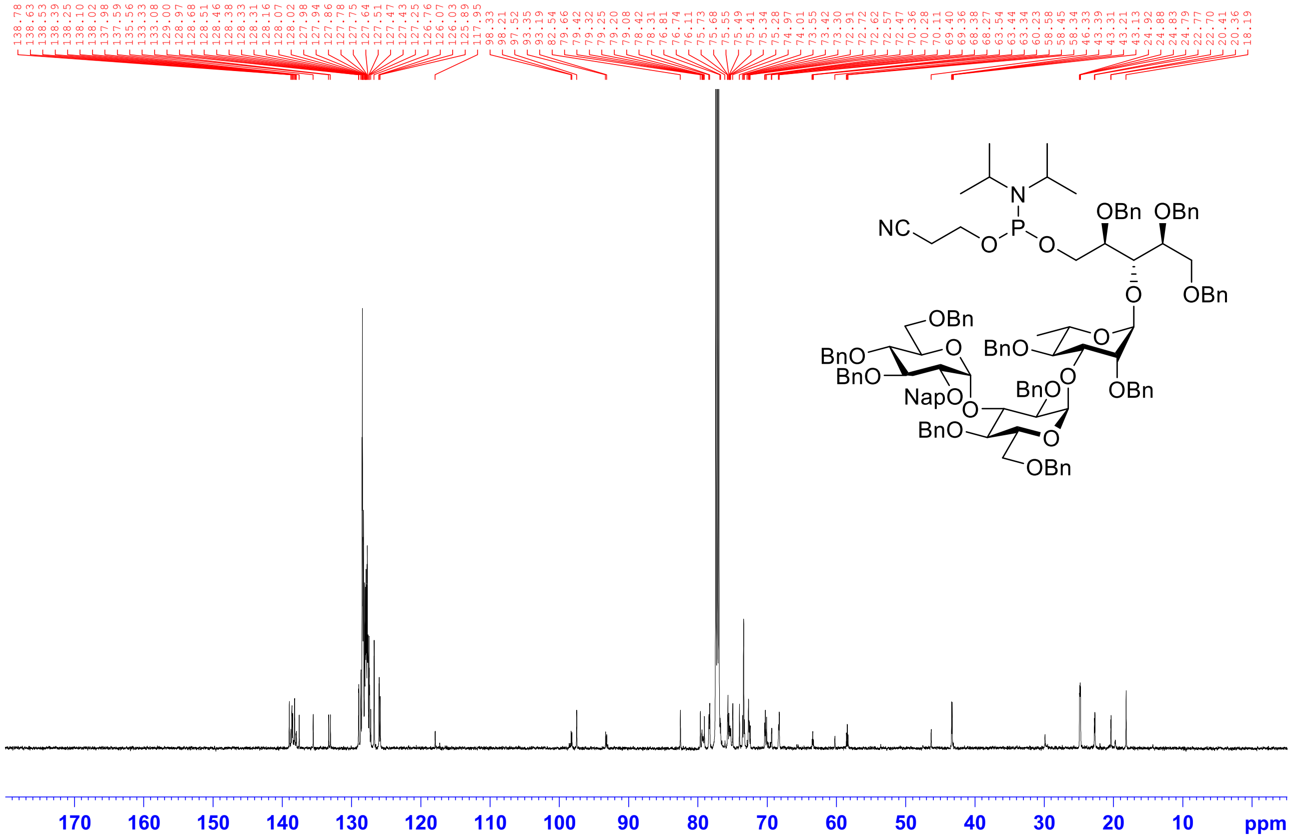

S20: DEPT 135 (150 MHz, $CDCl_3$)

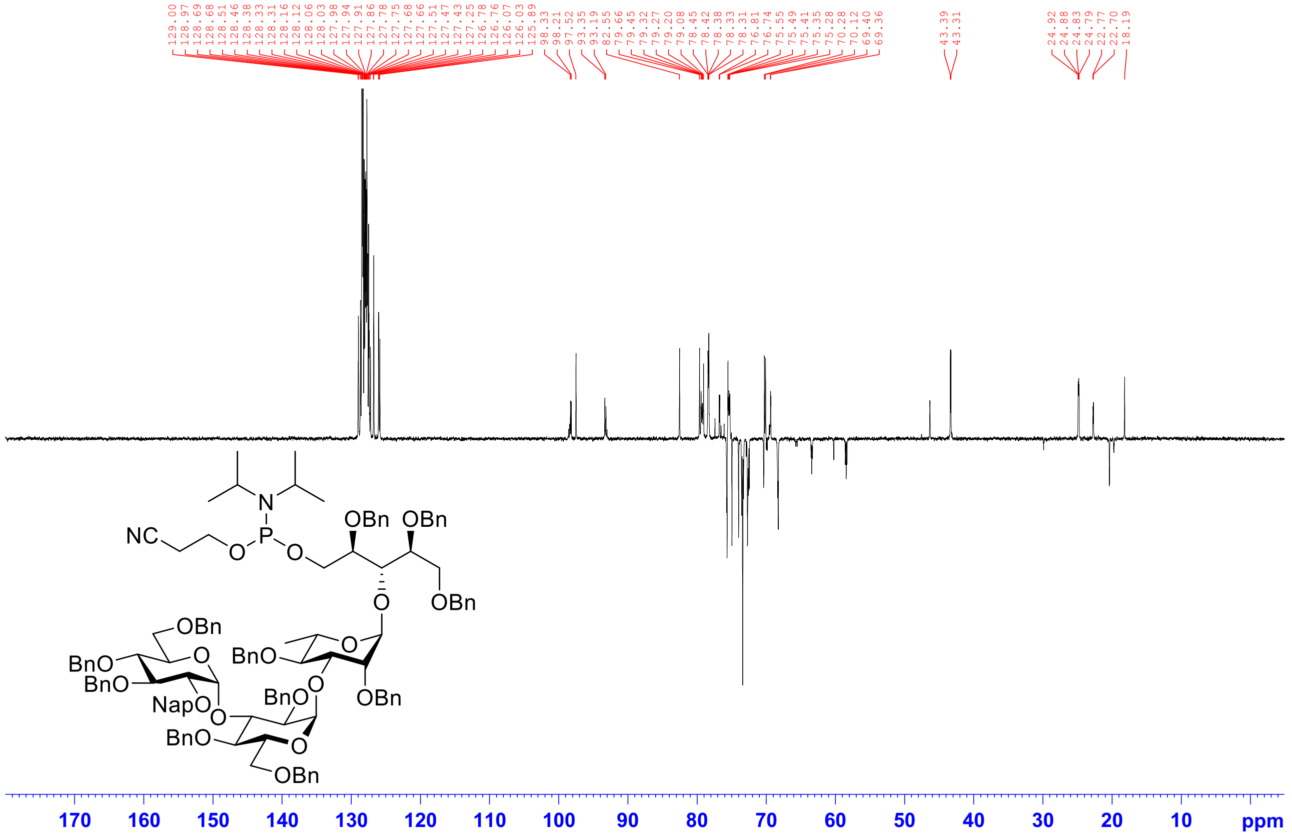

S20: ^{31}P NMR (202 MHz, $CDCl_3$)

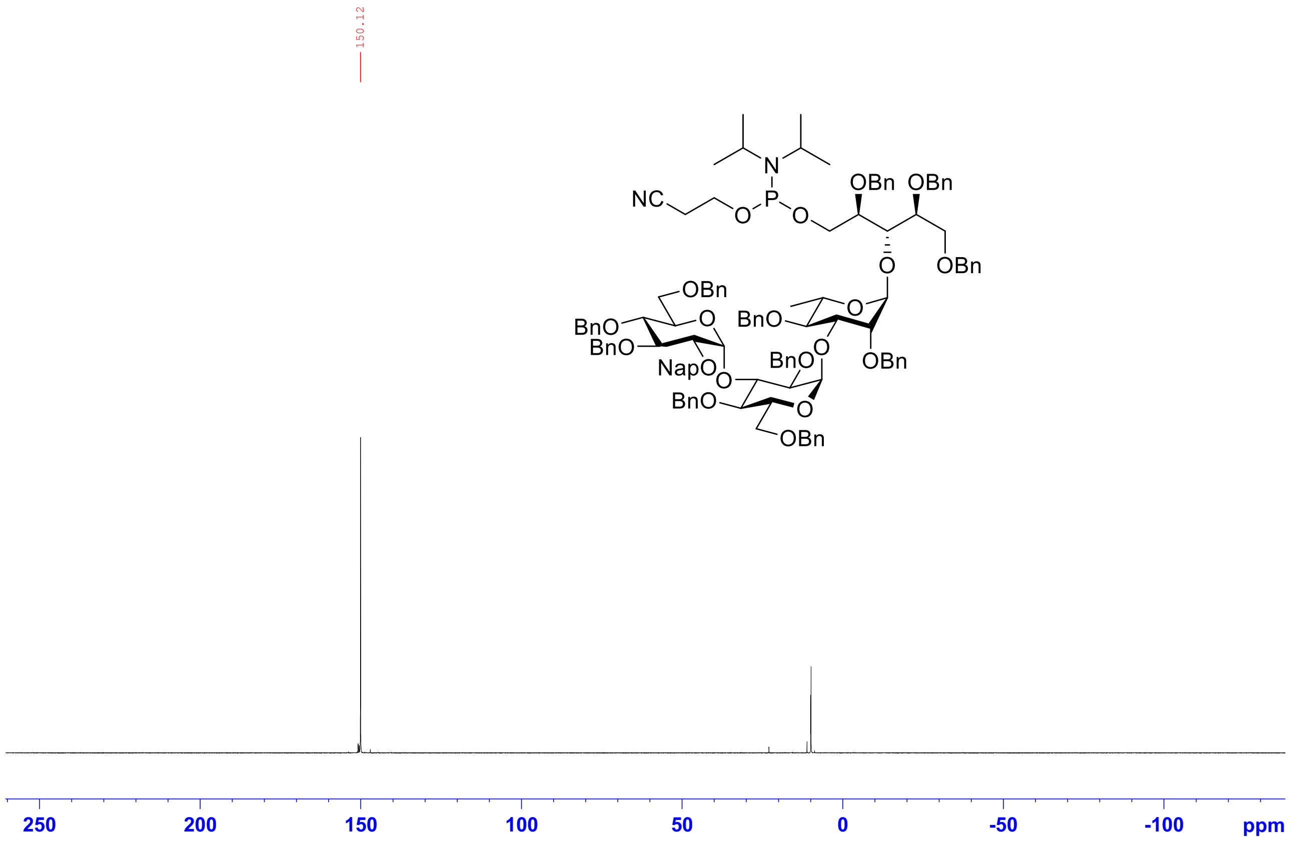

31: ^{1}H NMR (600 MHz, $CDCl_3$)

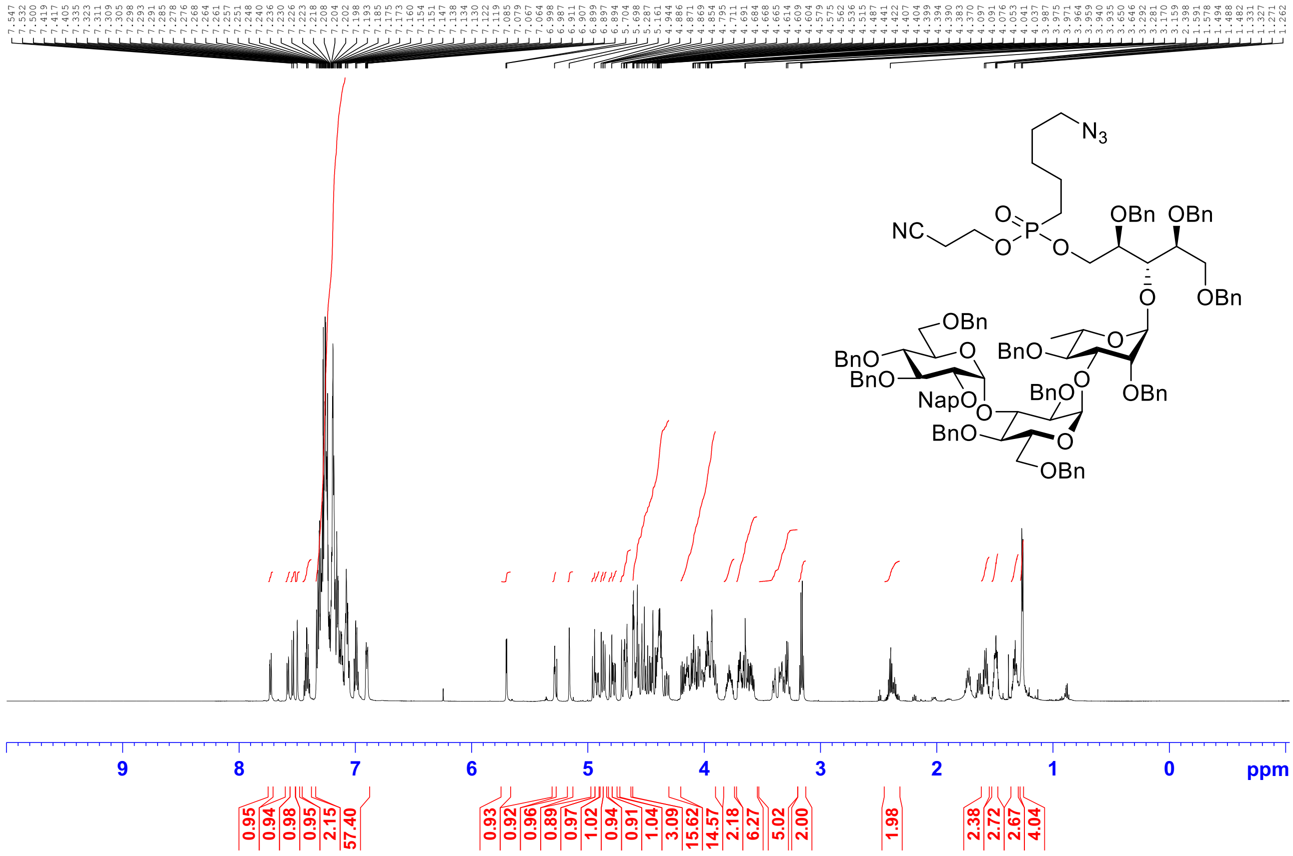

31: ^{13}C NMR (150 MHz, $CDCl_3$)

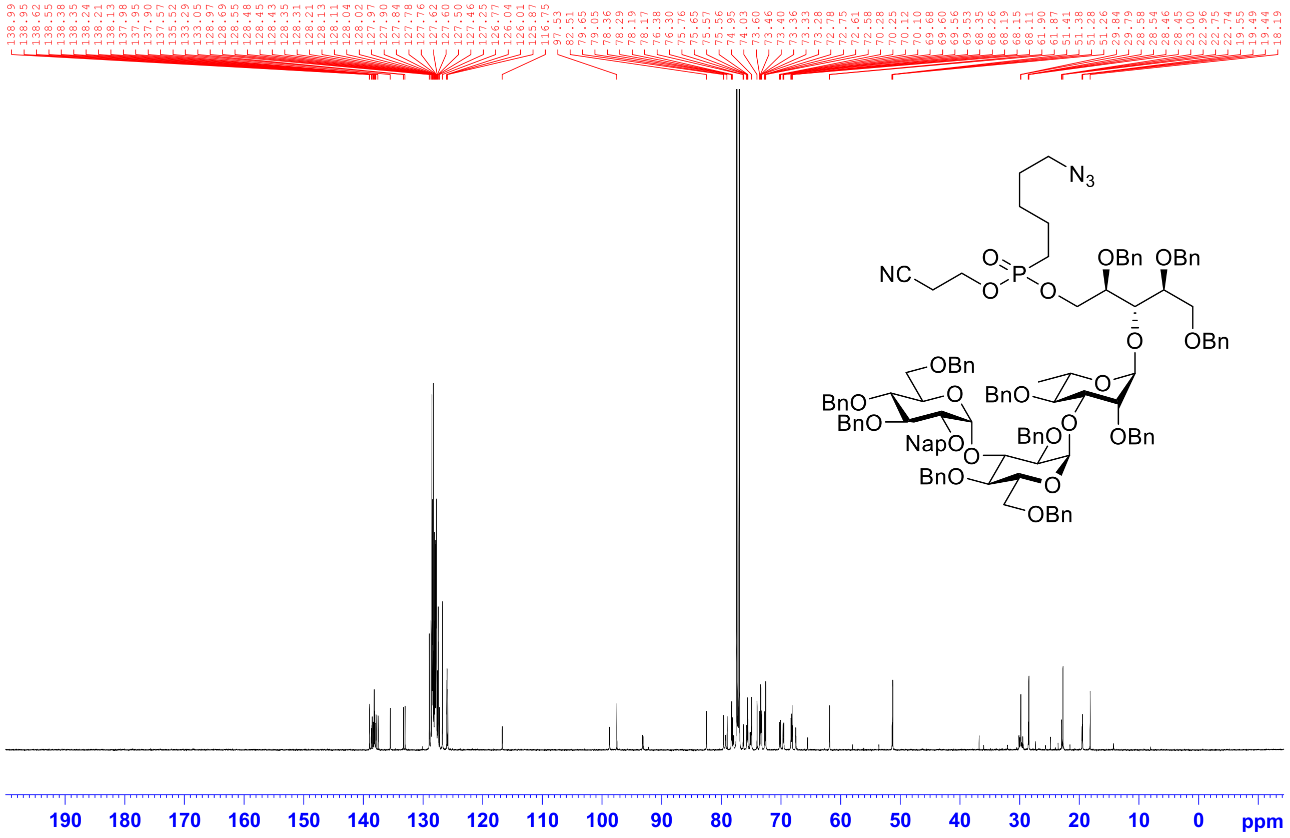

31: DEPT 135 (150 MHz, $CDCl_3$)

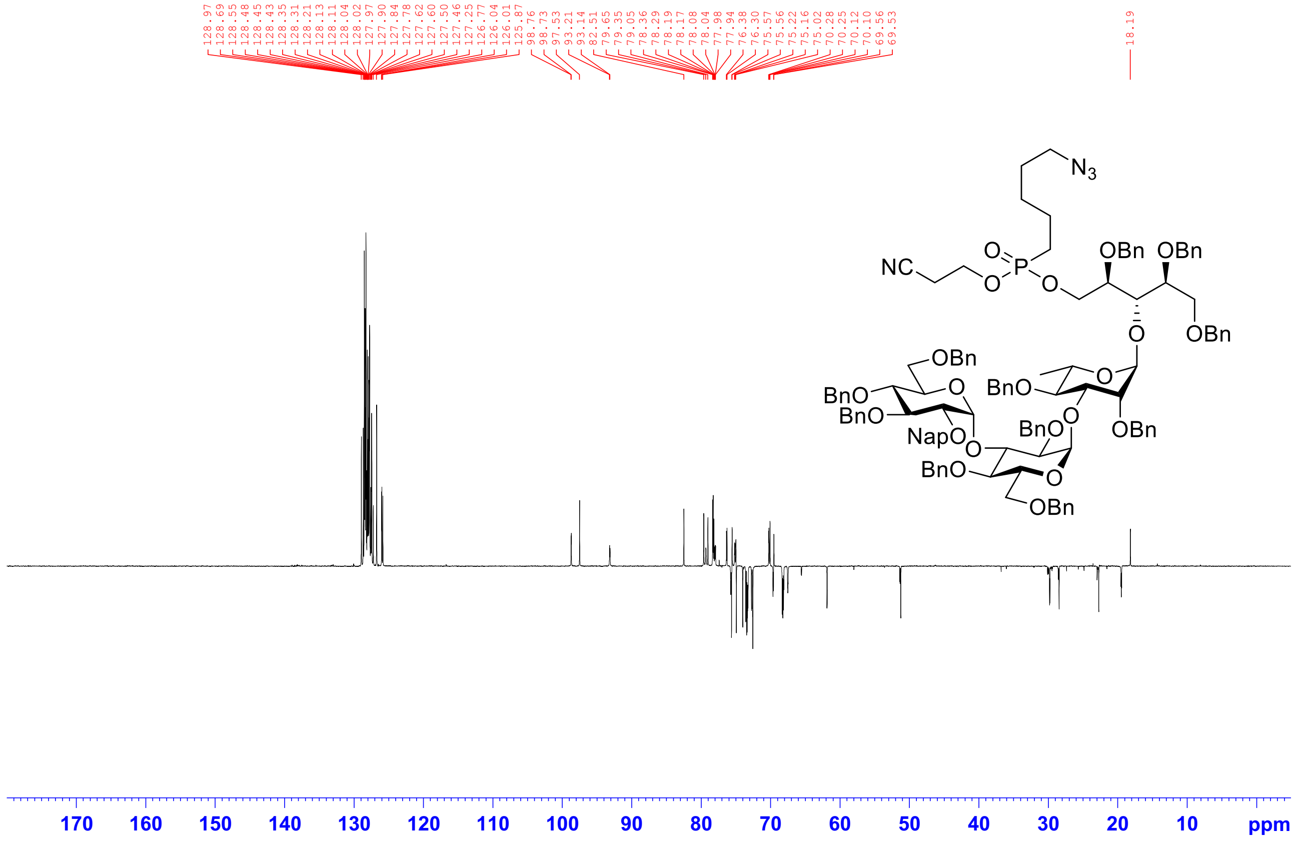

37: ^{1}H NMR (600 MHz, $CDCl_3$)

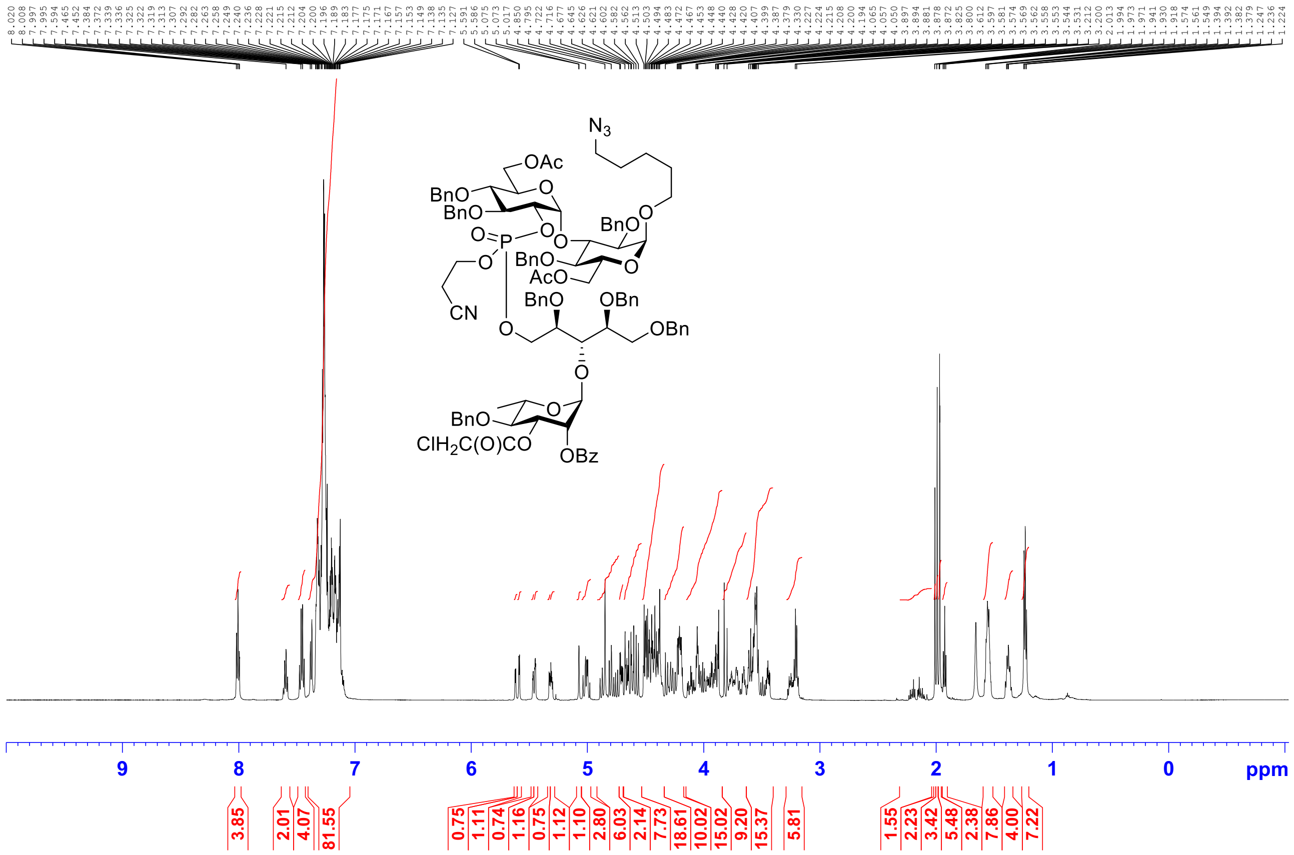

37: ^{13}C NMR (150 MHz, $CDCl_3$)

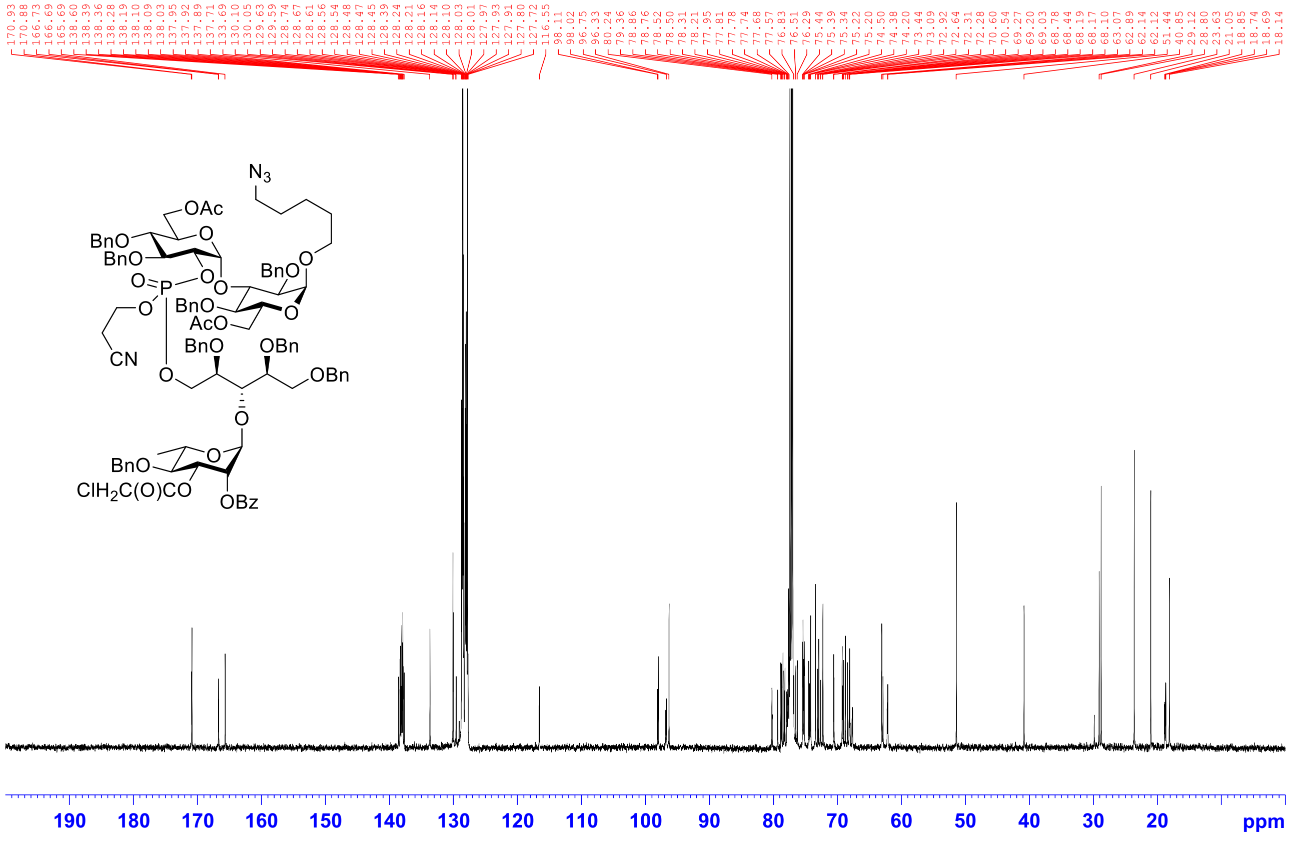

37: DEPT 135 (150 MHz, $CDCl_3$)

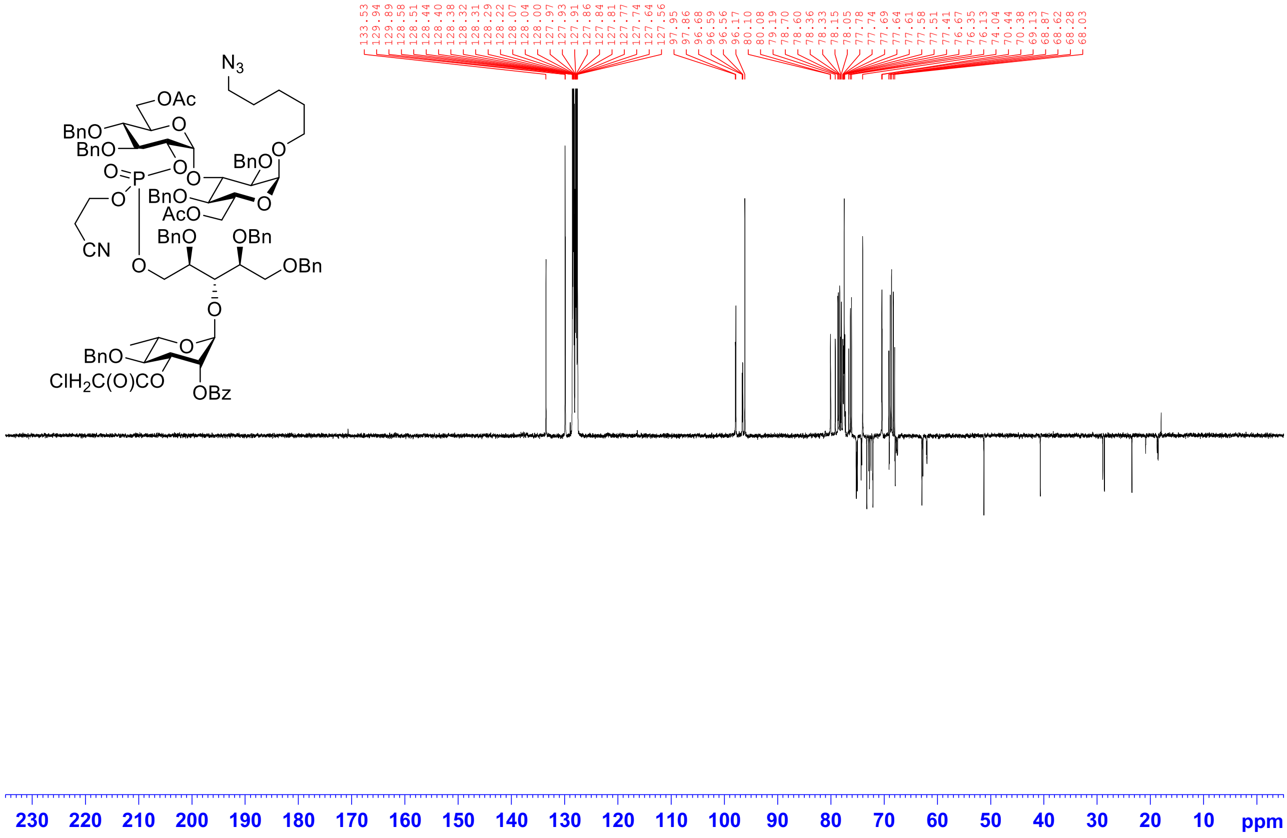

37: ^{31}P NMR (202 MHz, $CDCl_3$)

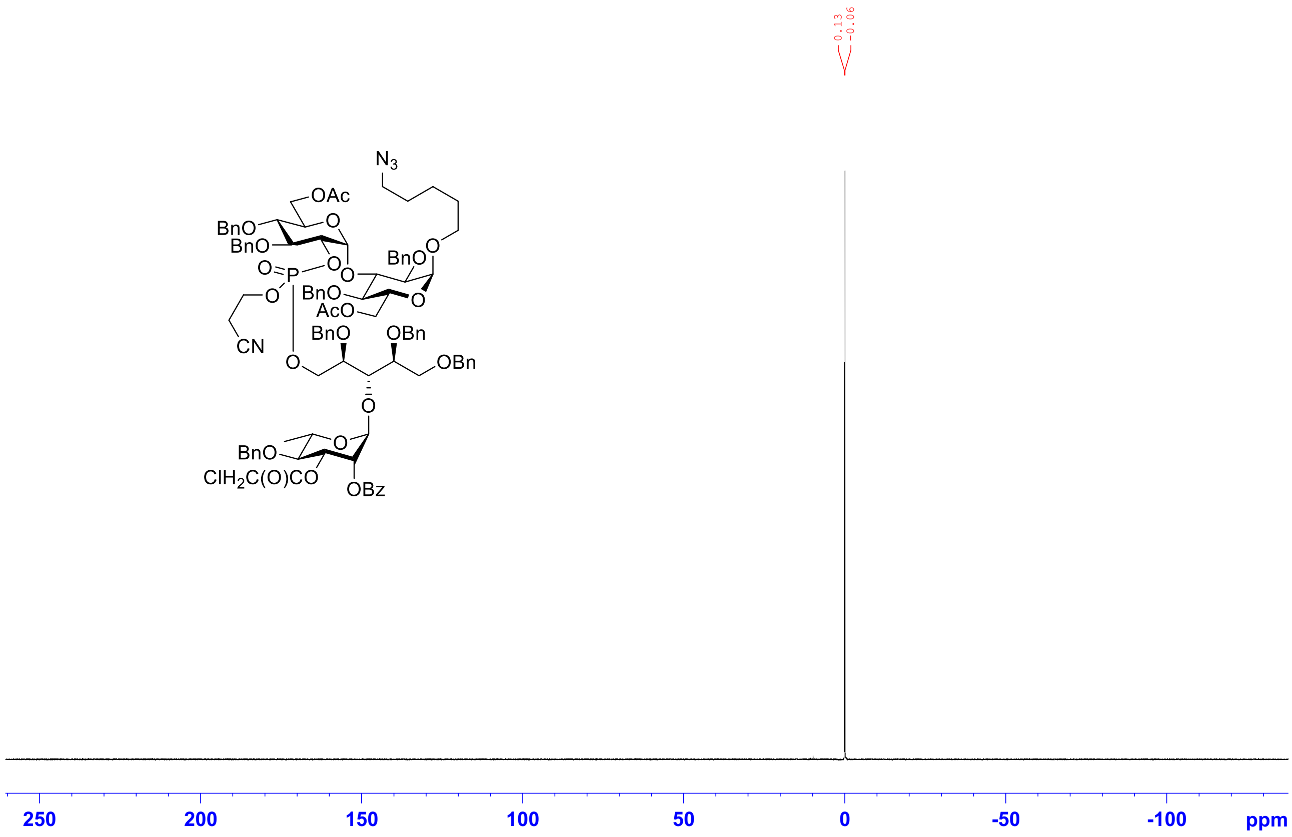

NMR spectra of pseudo-tetrasaccharides for ST6D

S21: ^{1}H NMR (600 MHz, $CDCl_3$)

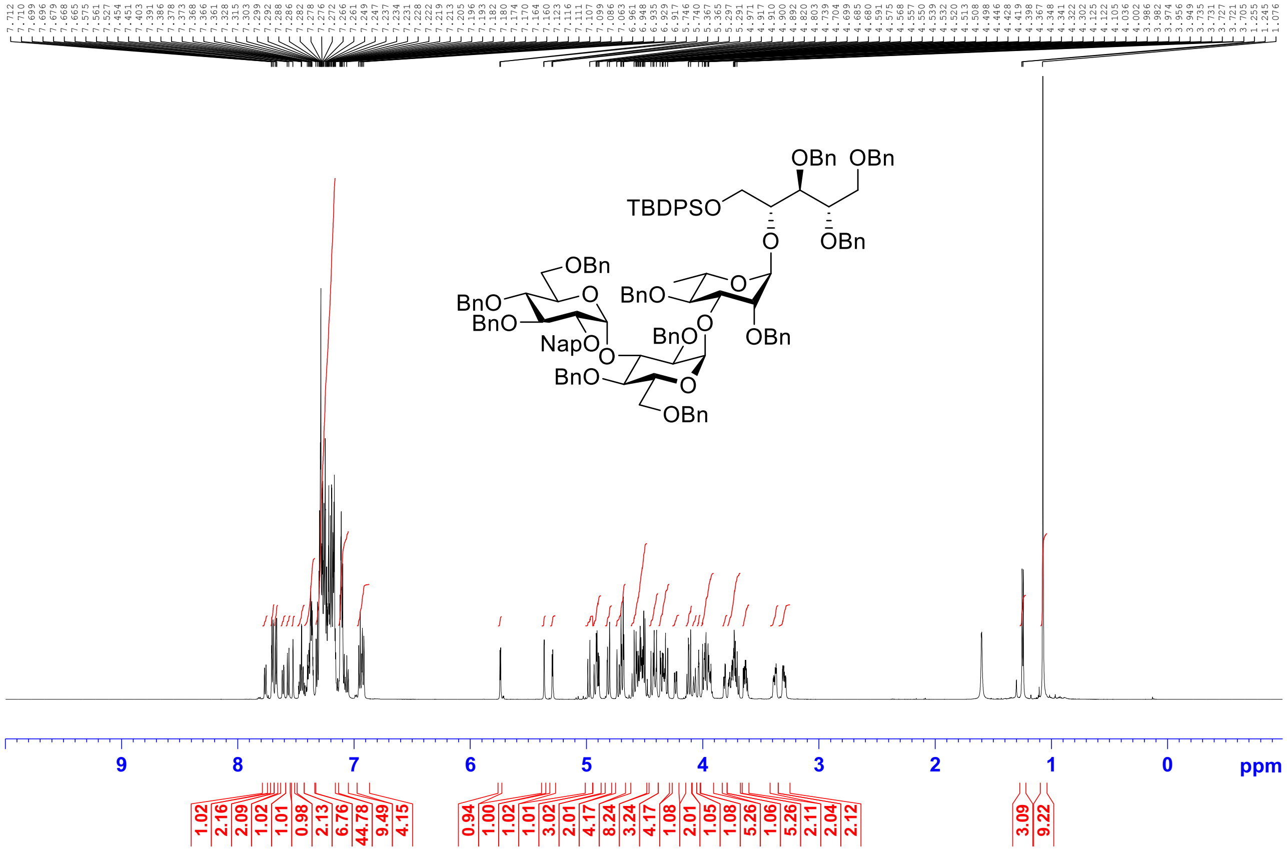

S21: ^{13}C NMR (150 MHz, $CDCl_3$)

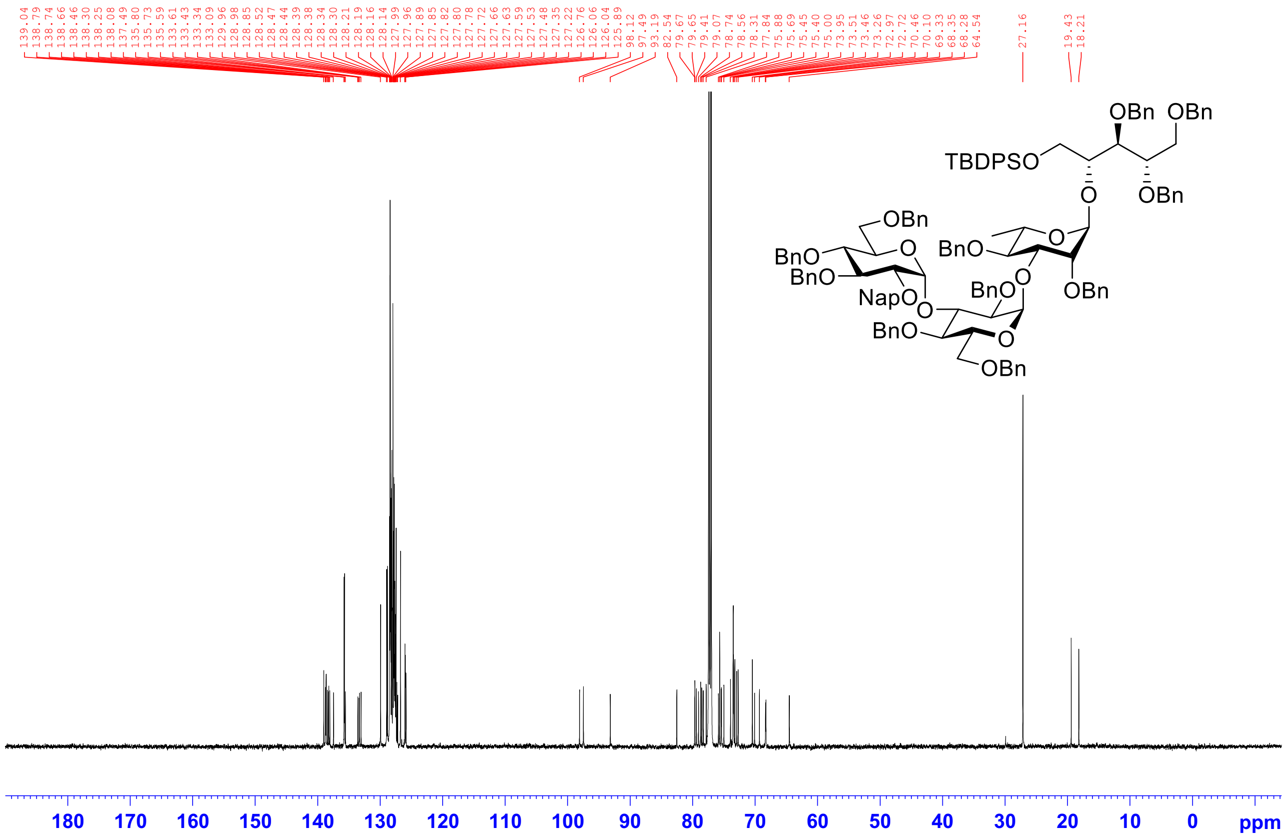

S21: DEPT 135 (150 MHz, $CDCl_3$)

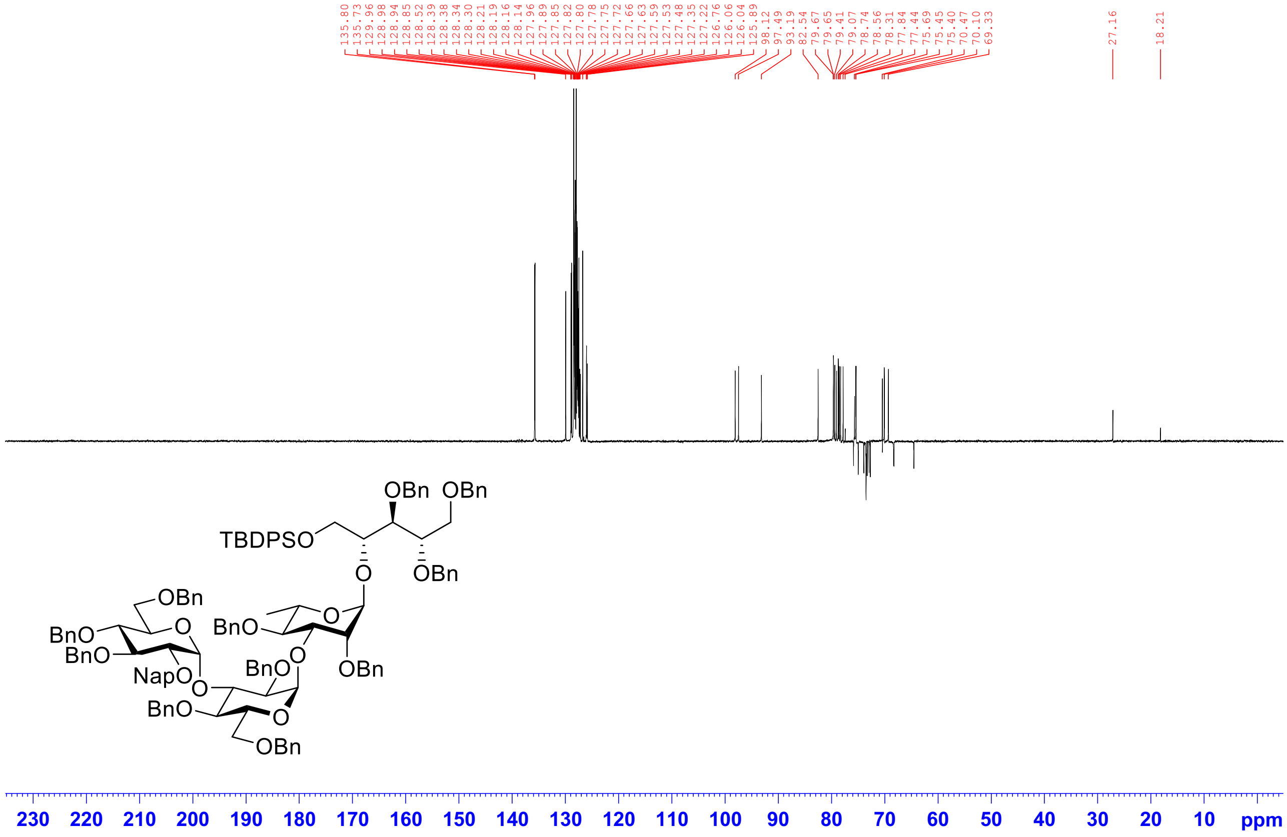

24: ^{1}H NMR (600 MHz, $CDCl_3$)

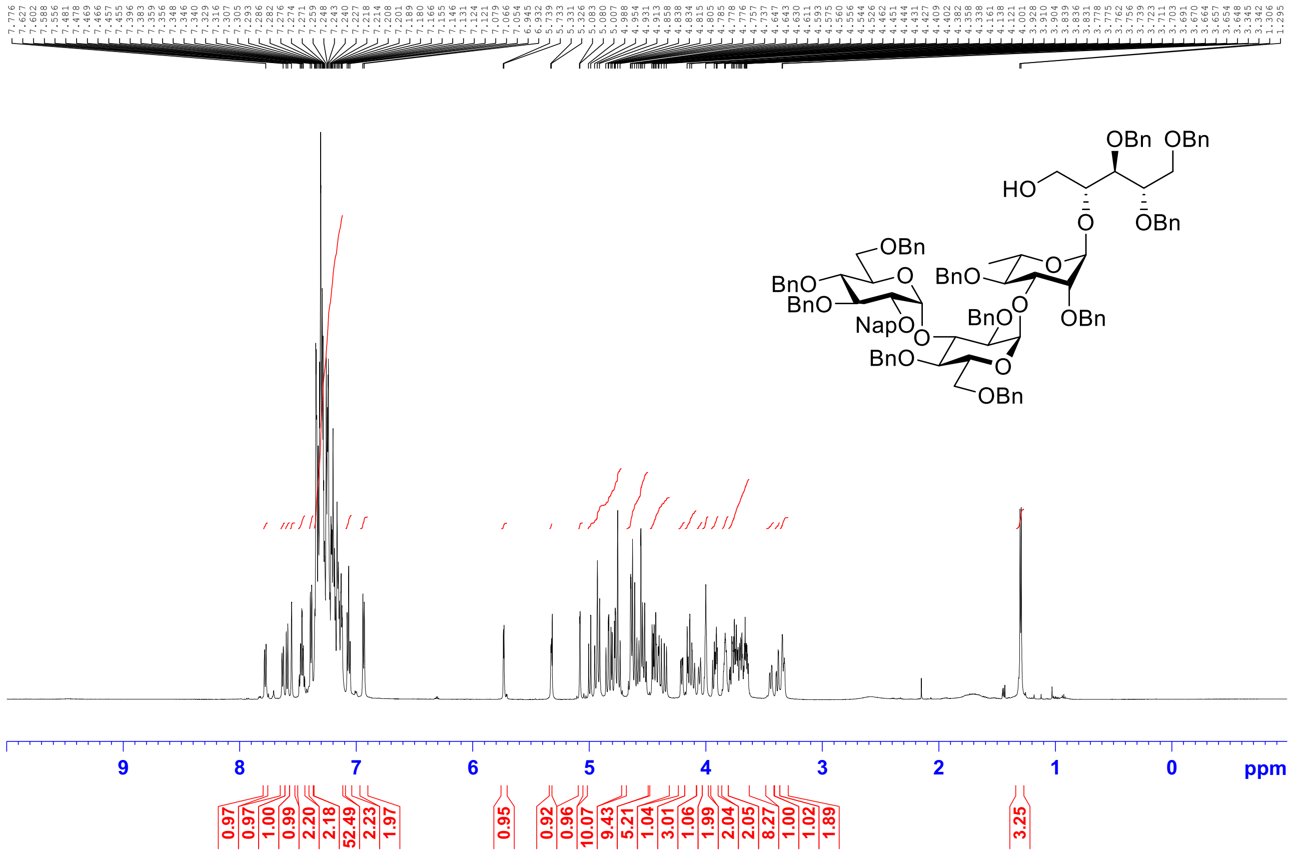

24: ^{13}C NMR (150 MHz, $CDCl_3$)

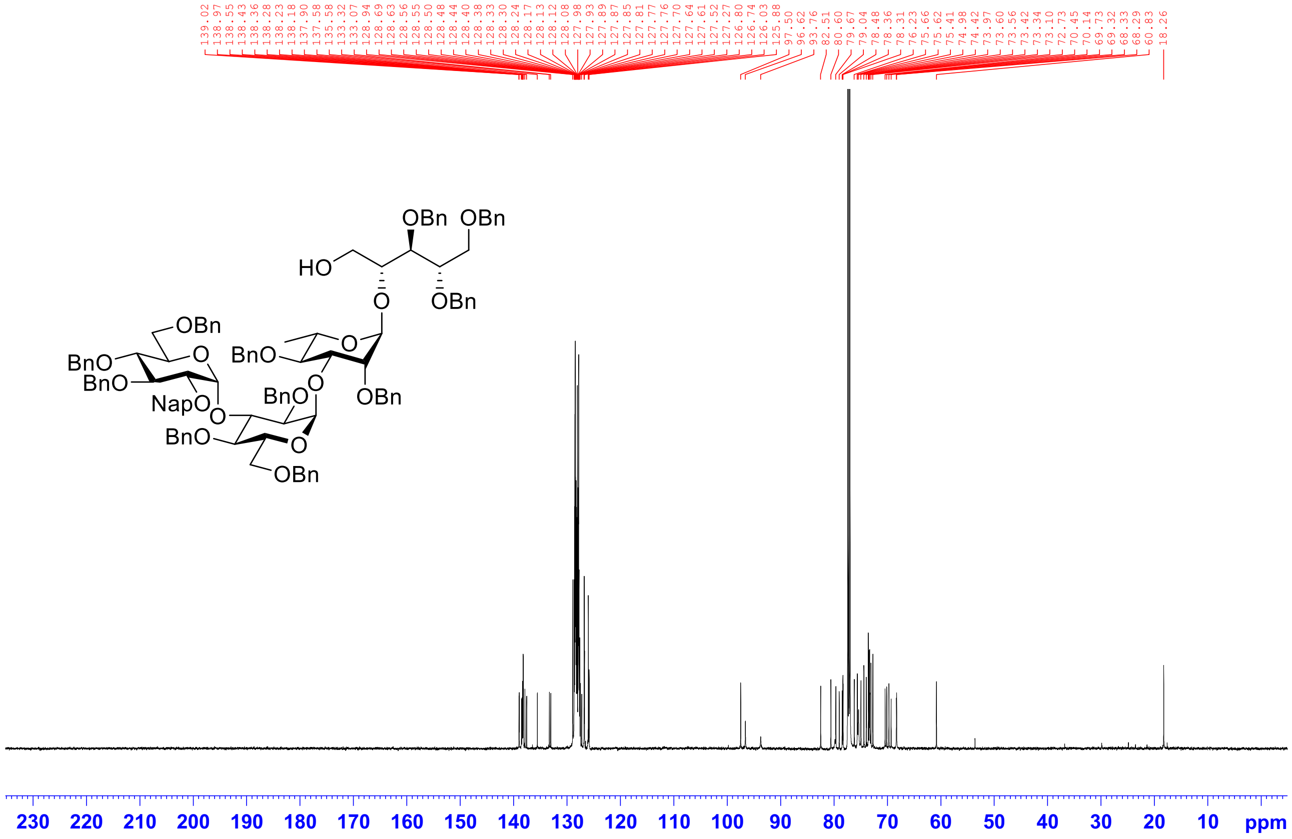

24: DEPT 135 (150 MHz, $CDCl_3$)

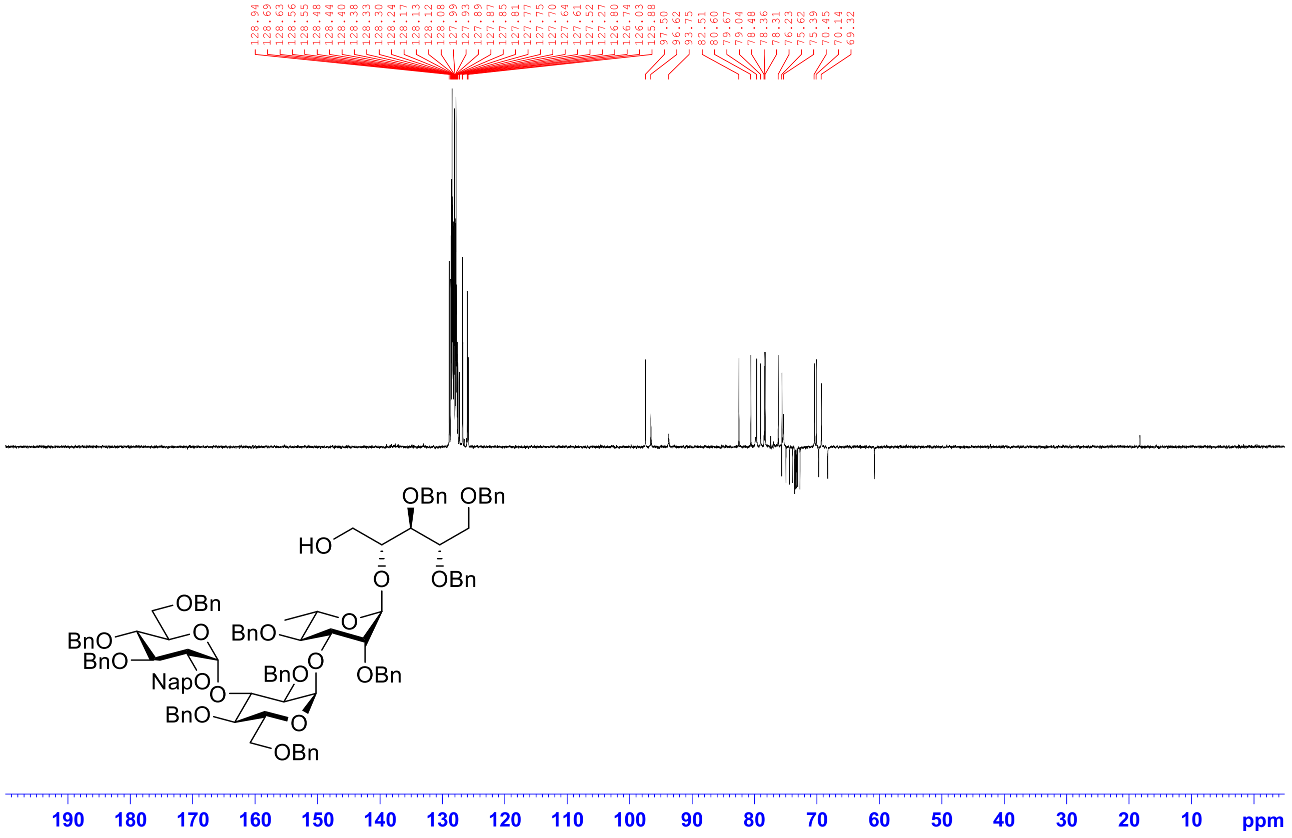

27: ^{1}H NMR (600 MHz, $CDCl_3$)

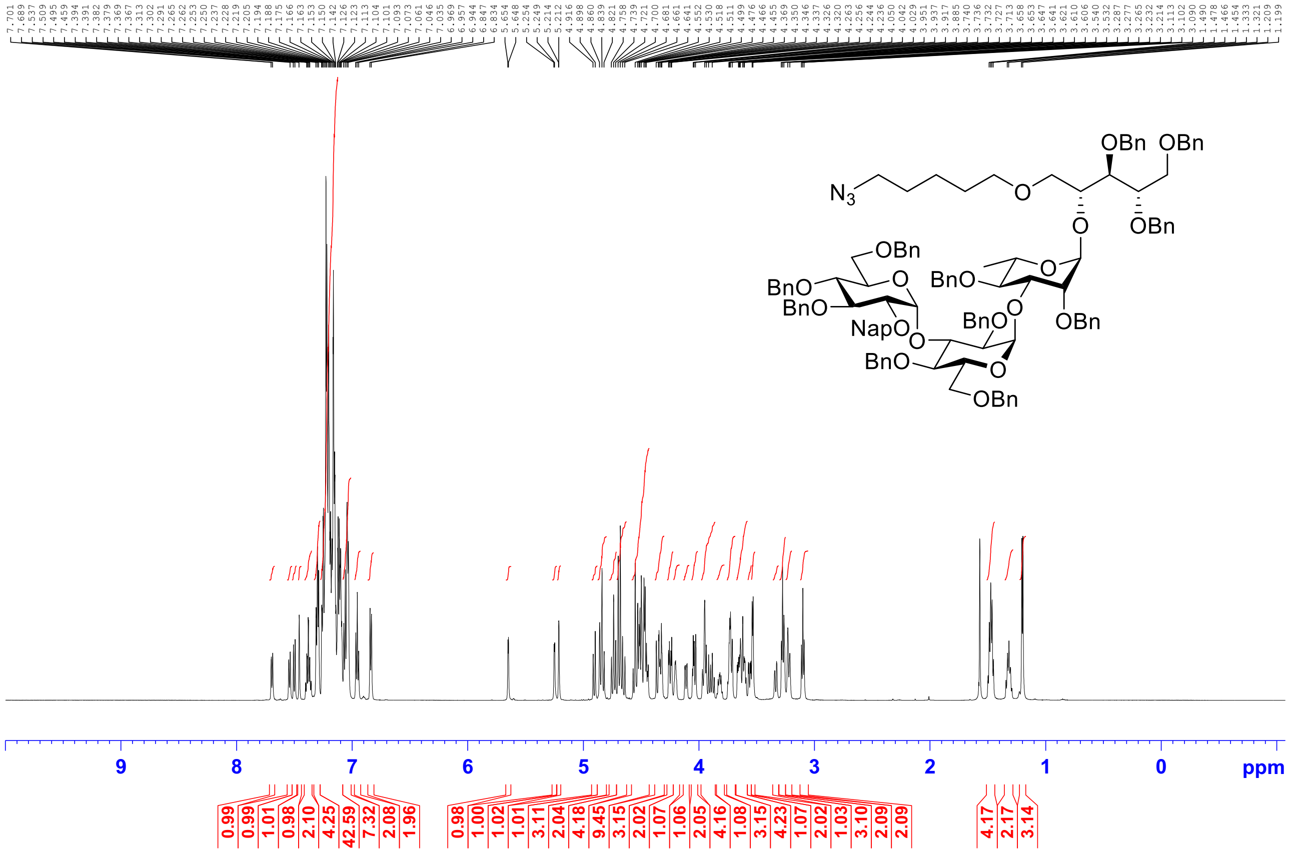

27: ^{13}C NMR (150 MHz, $CDCl_3$)

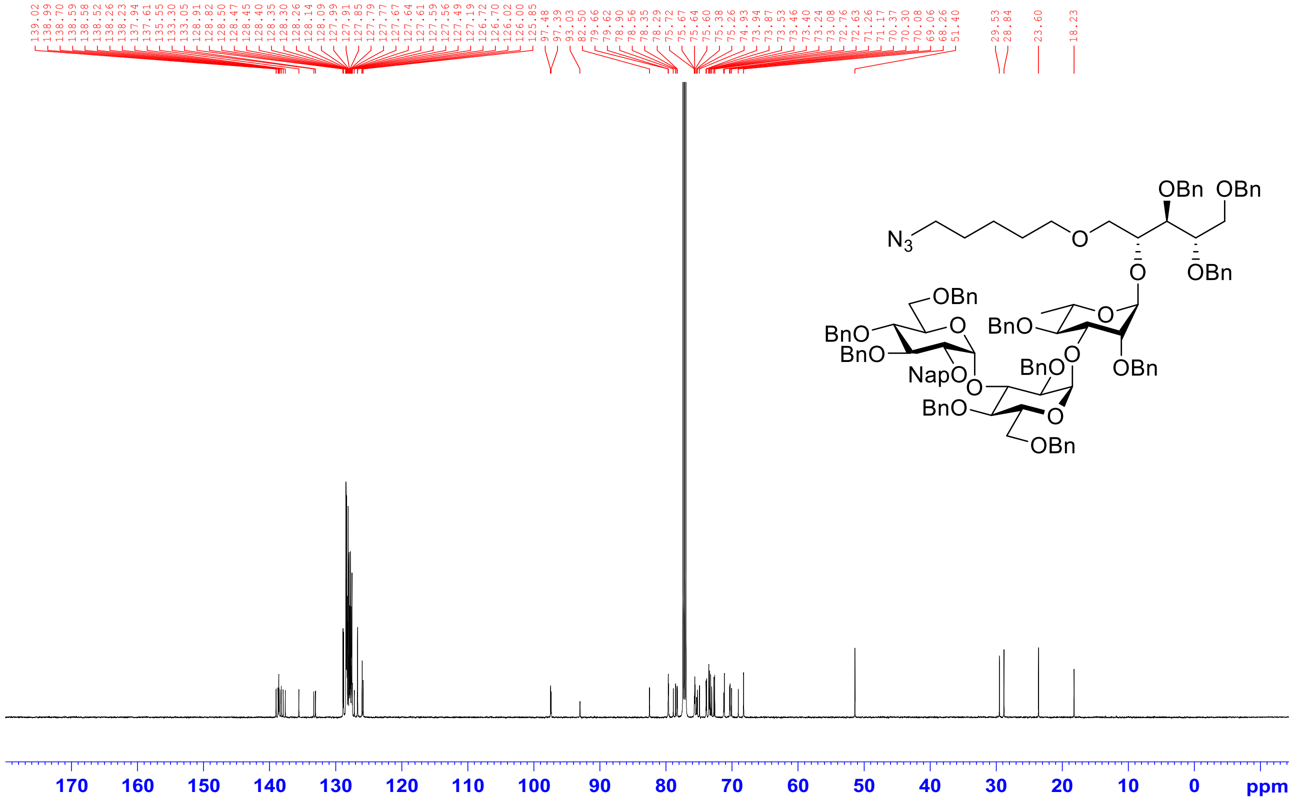

27: DEPT 135 (150 MHz, $CDCl_3$)

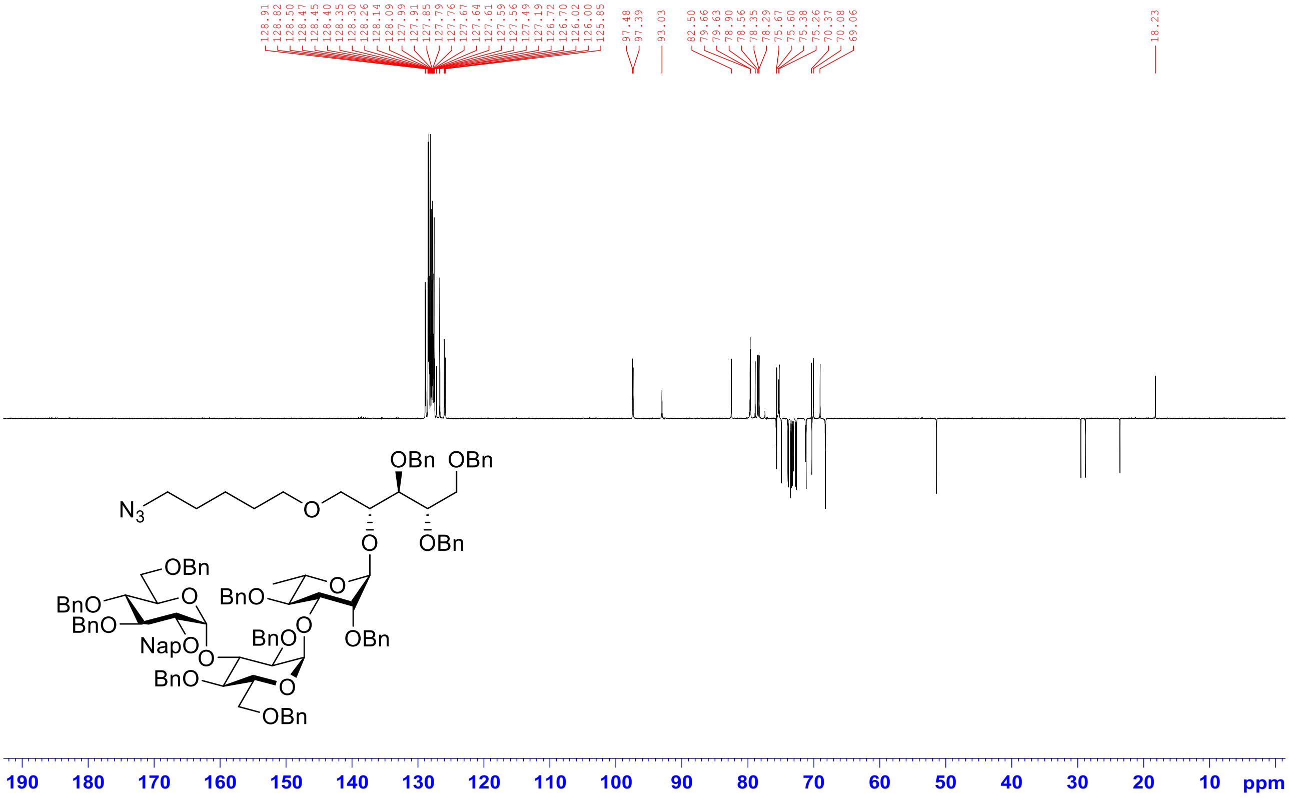

28: ^1H NMR (600 MHz, $CDCl_3$)

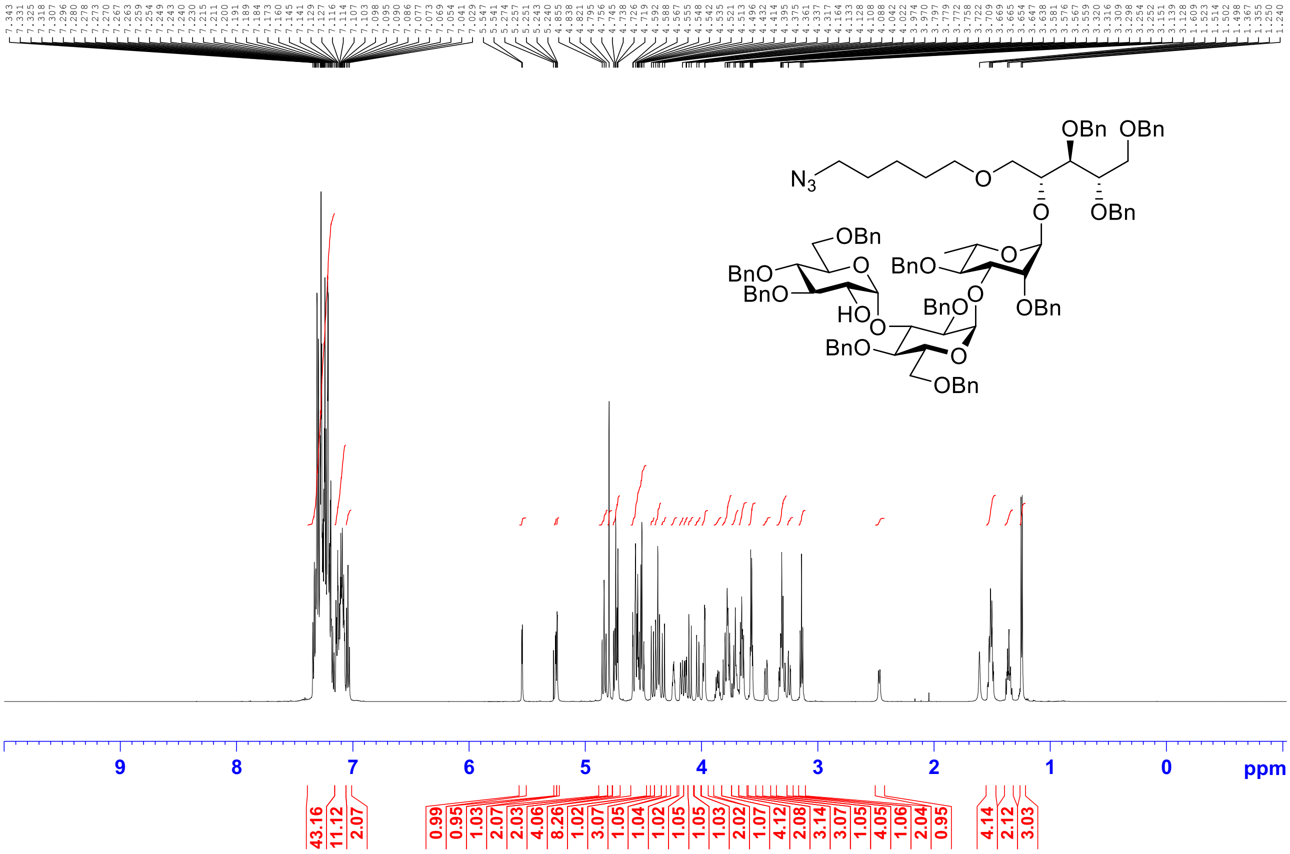

28: ^{13}C NMR (150 MHz, $CDCl_3$)

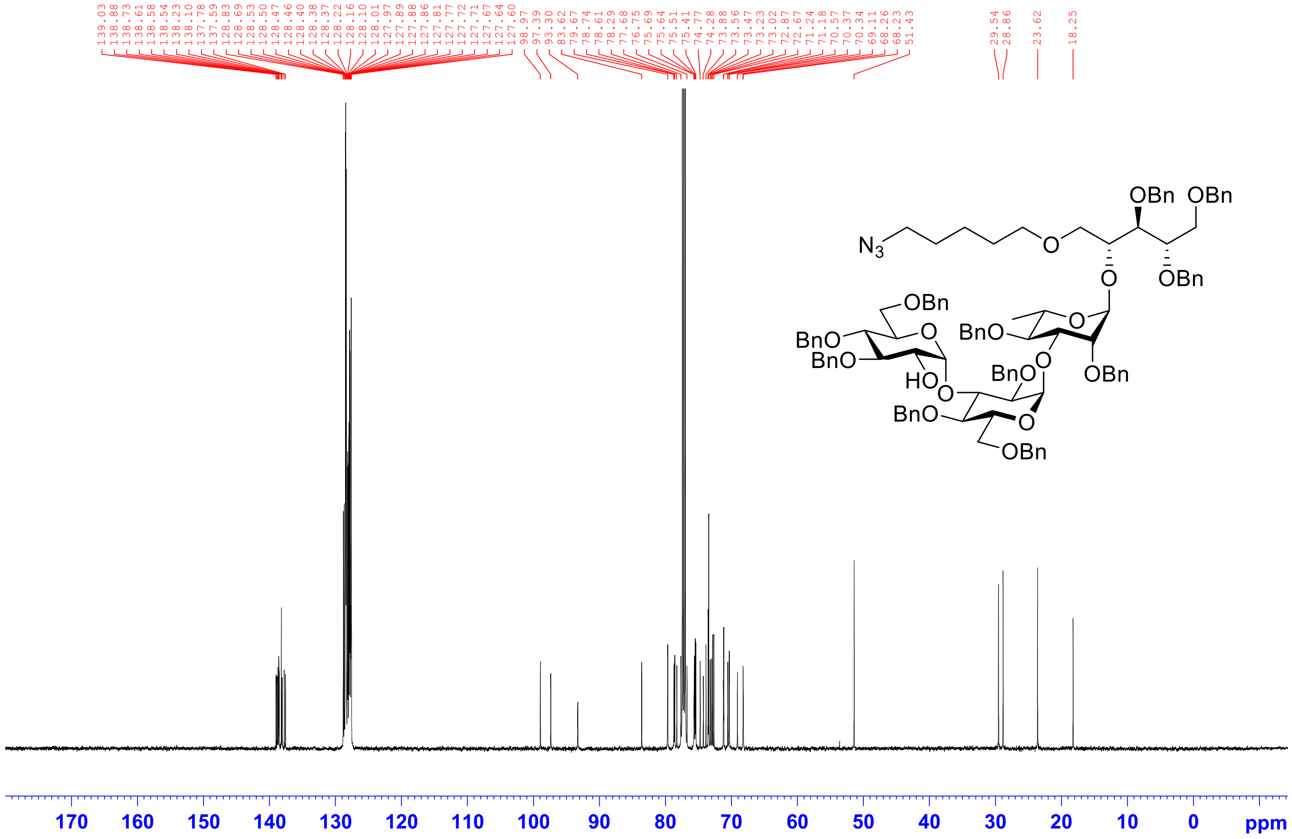

28: DEPT 135 (150 MHz, $CDCl_3$)

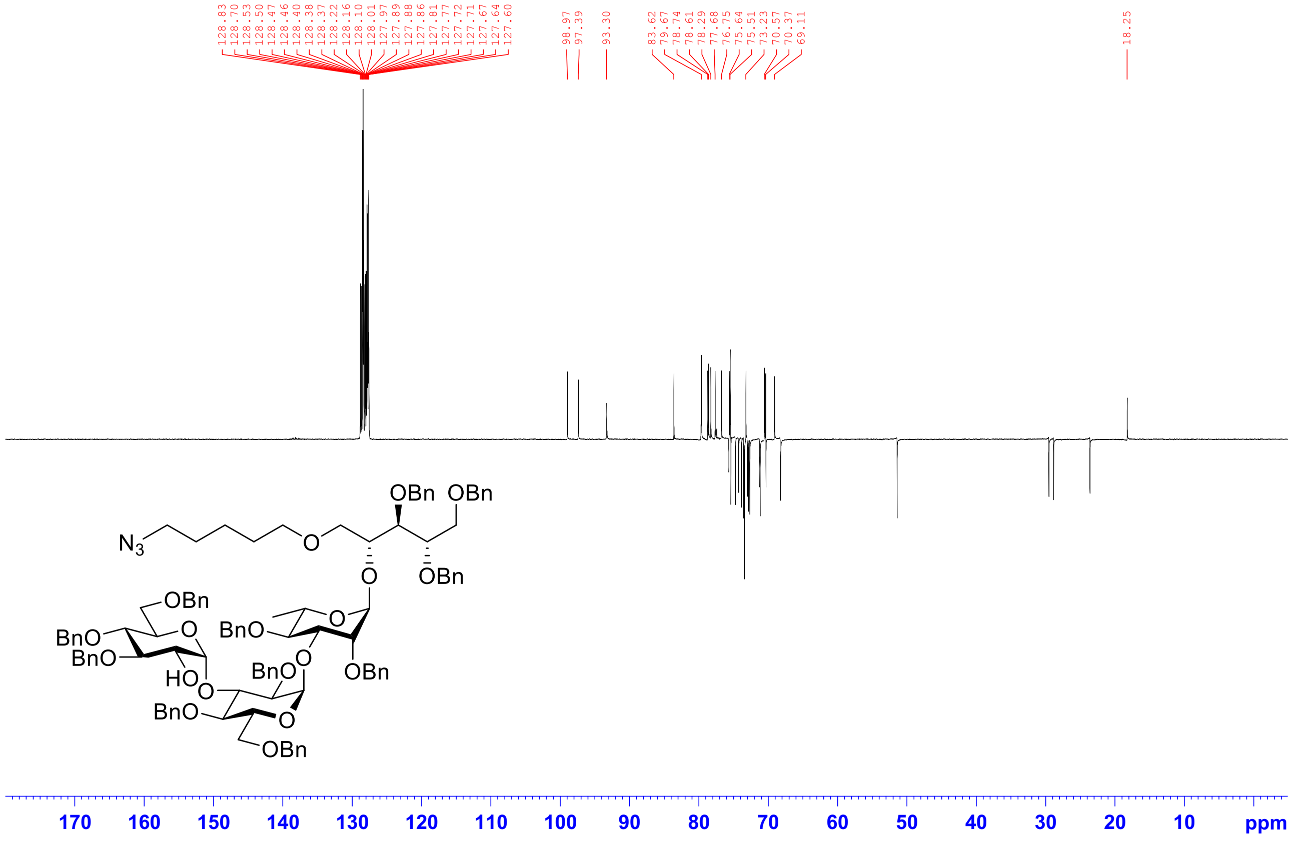

30: ^{1}H NMR (600 MHz, $CDCl_3$)

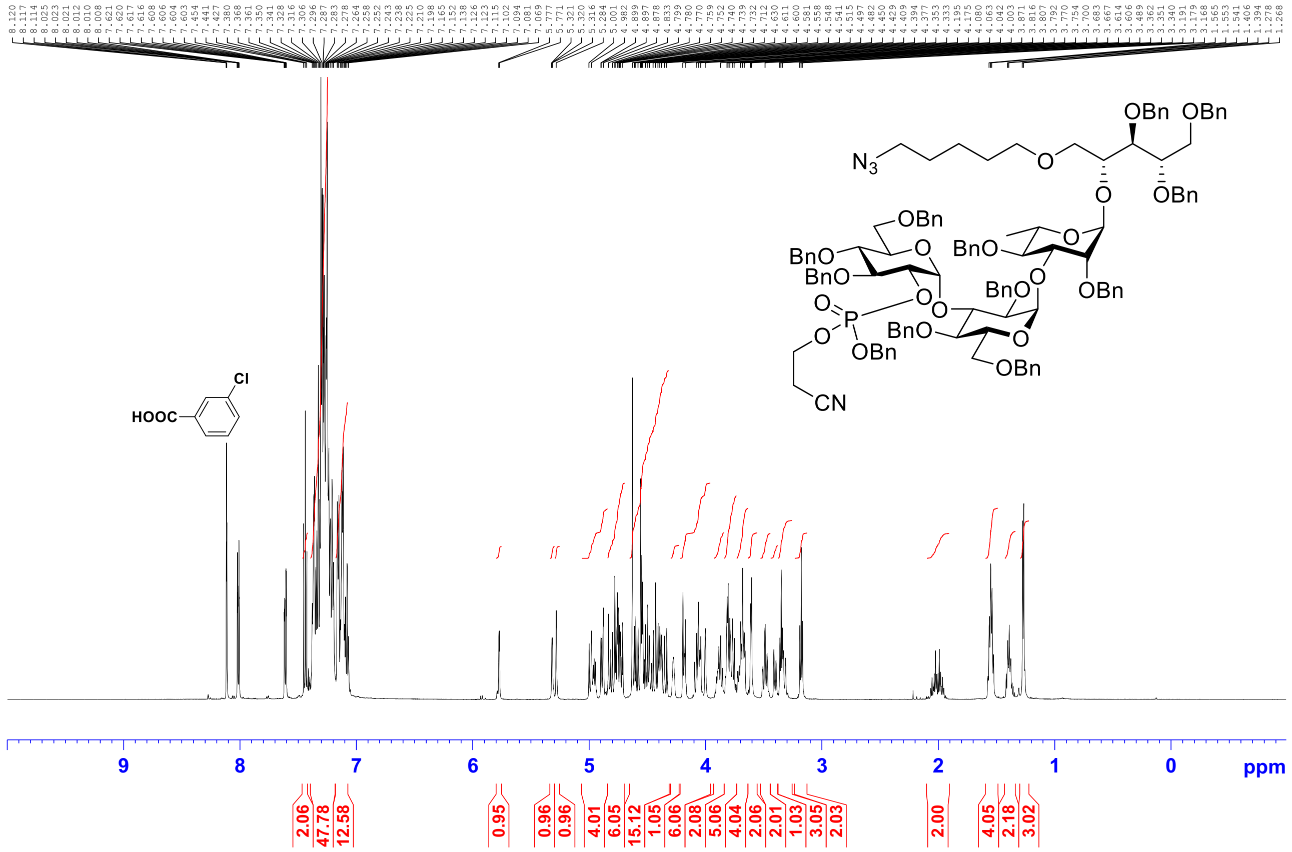

30: ^{13}C NMR (150 MHz, $CDCl_3$)

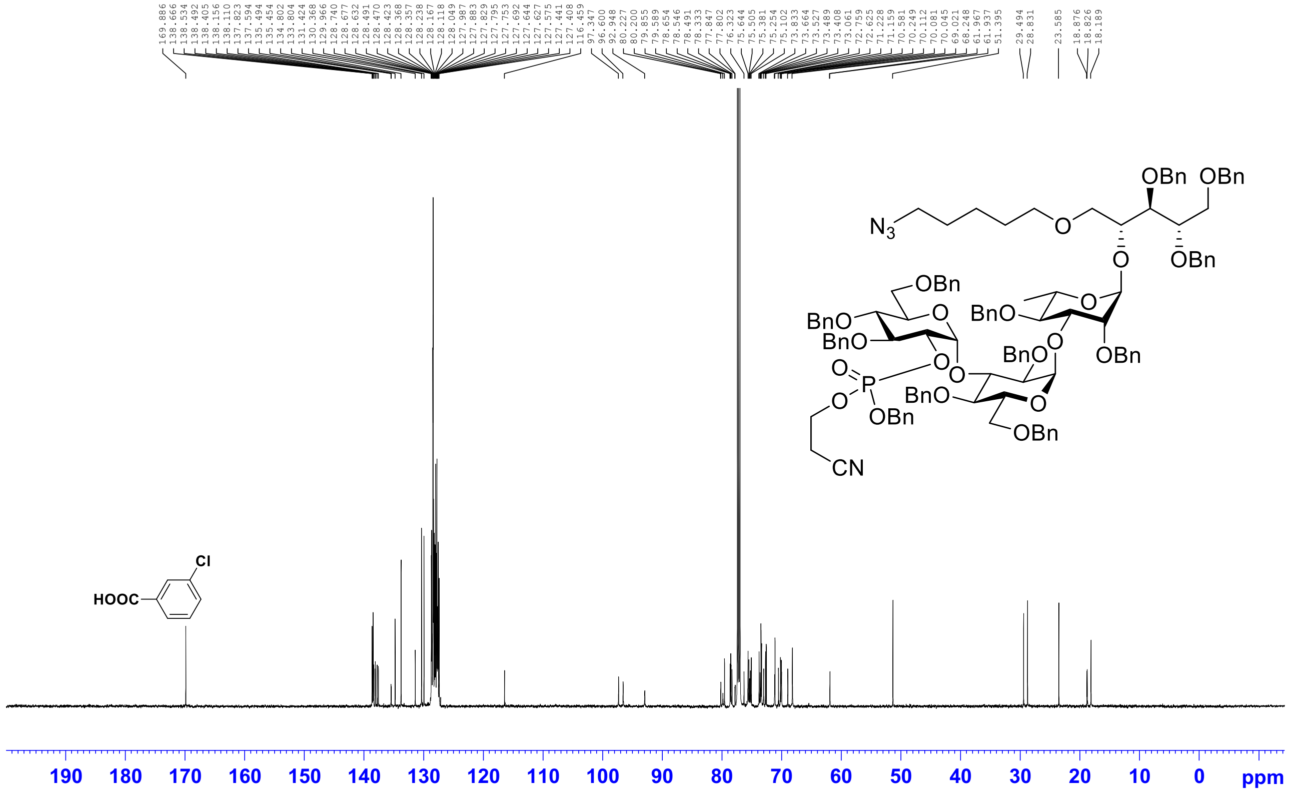

30: DEPT 135 (150 MHz, $CDCl_3$)

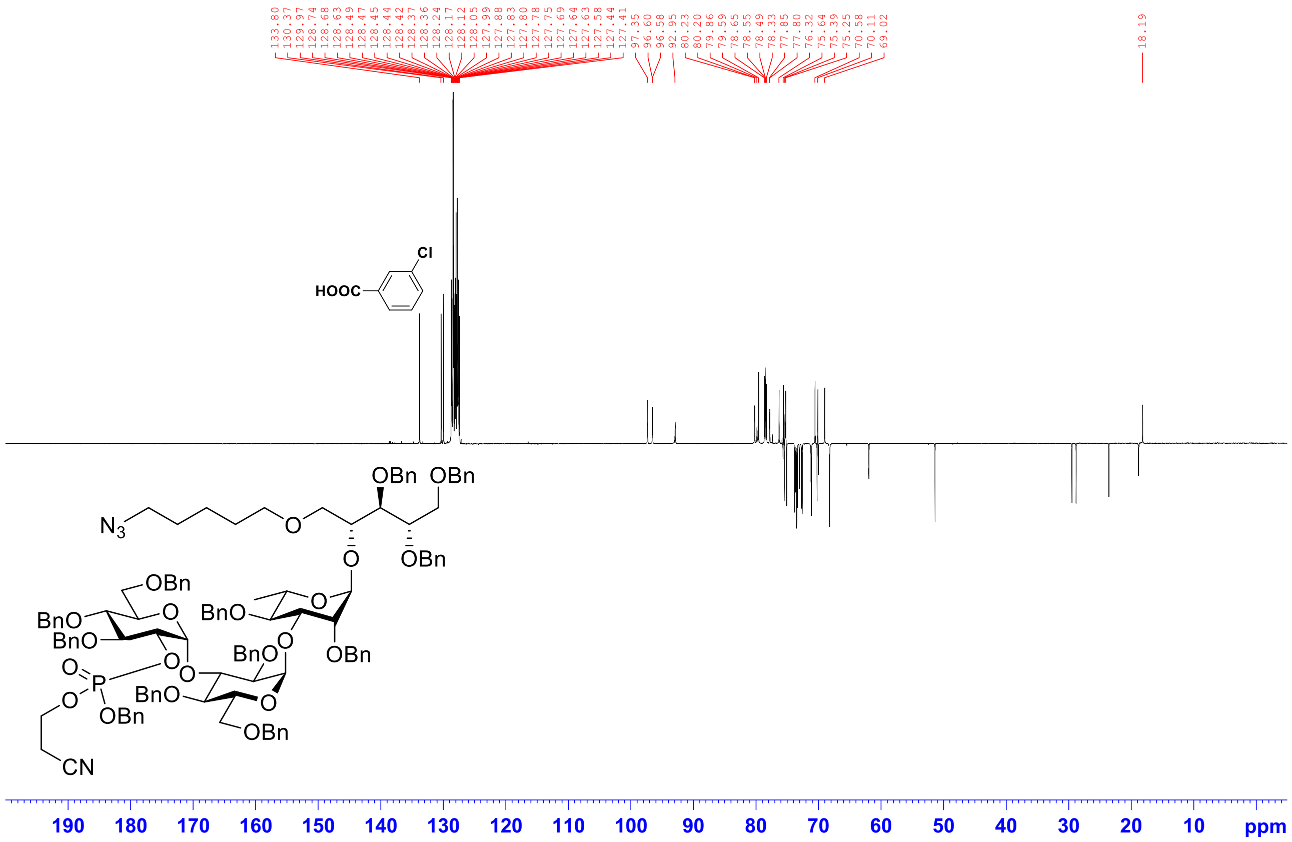

30: ^{31}P NMR (202 MHz, $CDCl_3$)

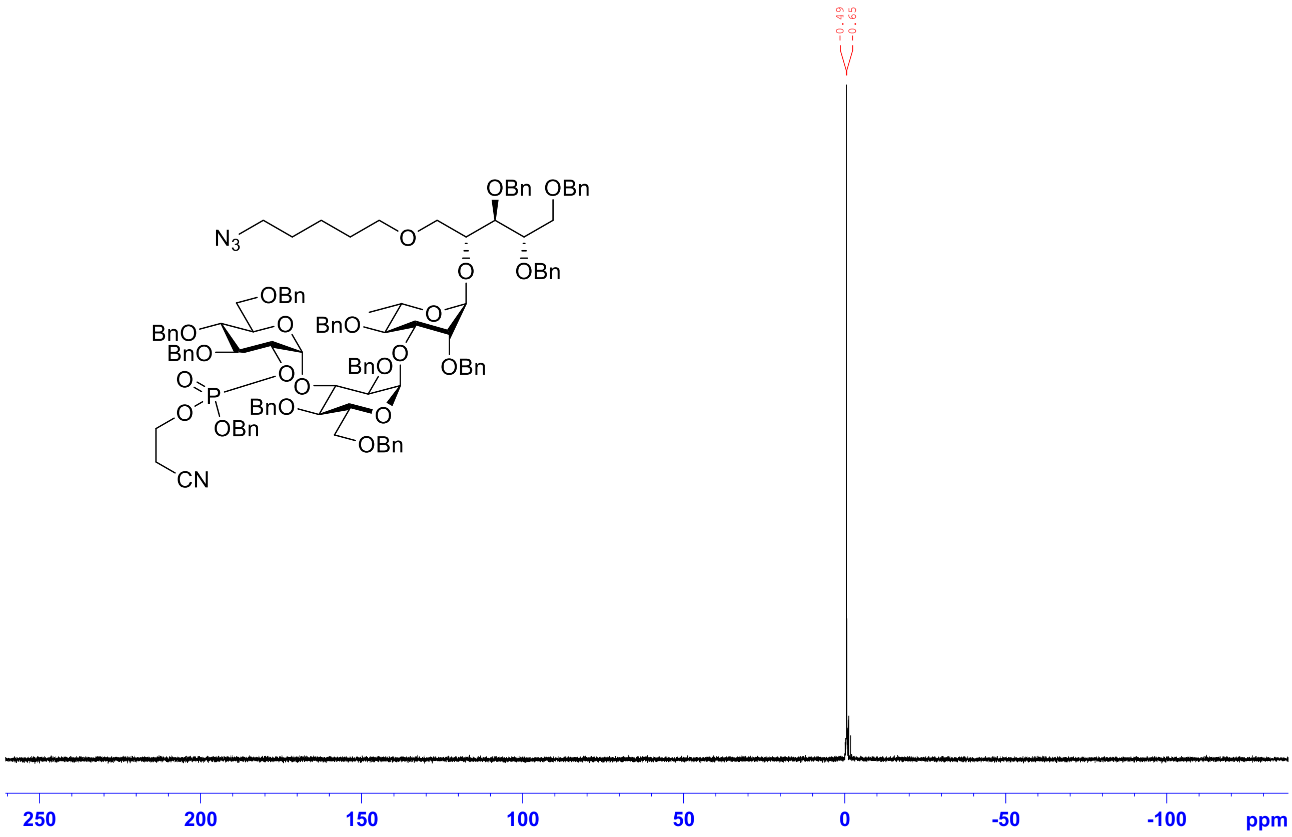

S22: ^{1}H NMR (600 MHz, $CDCl_3$)

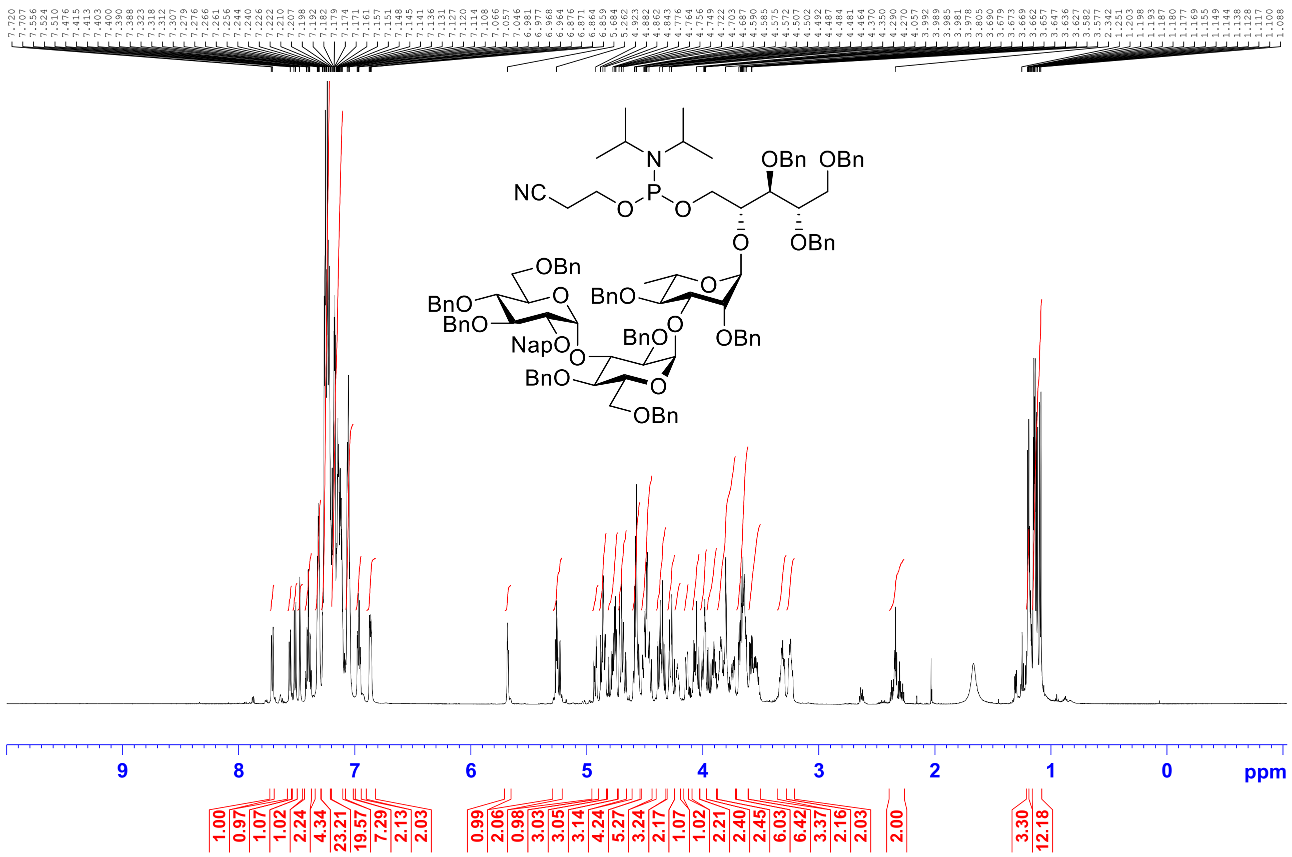

S22: ^{13}C NMR (150 MHz, $CDCl_3$)

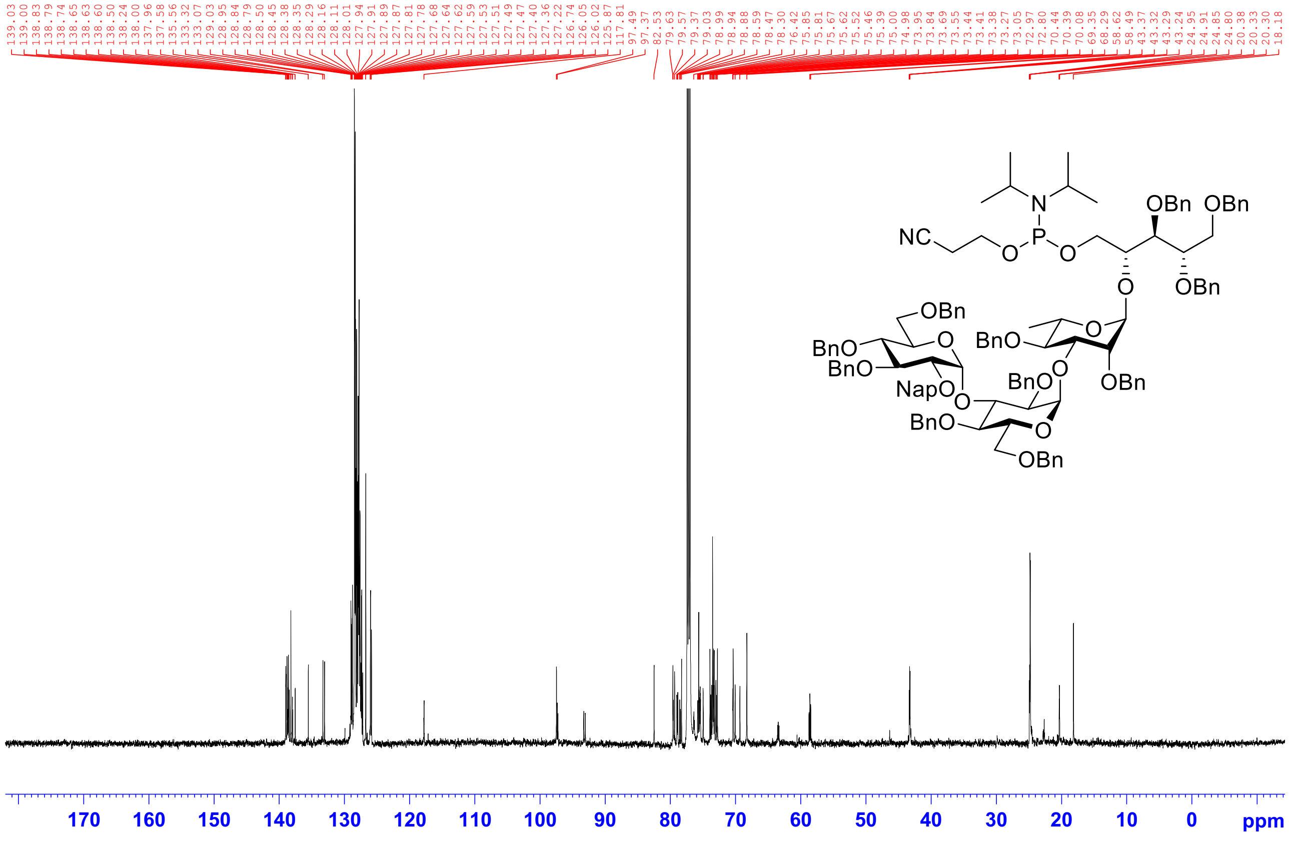

S22: DEPT 135 (150 MHz, $CDCl_3$)

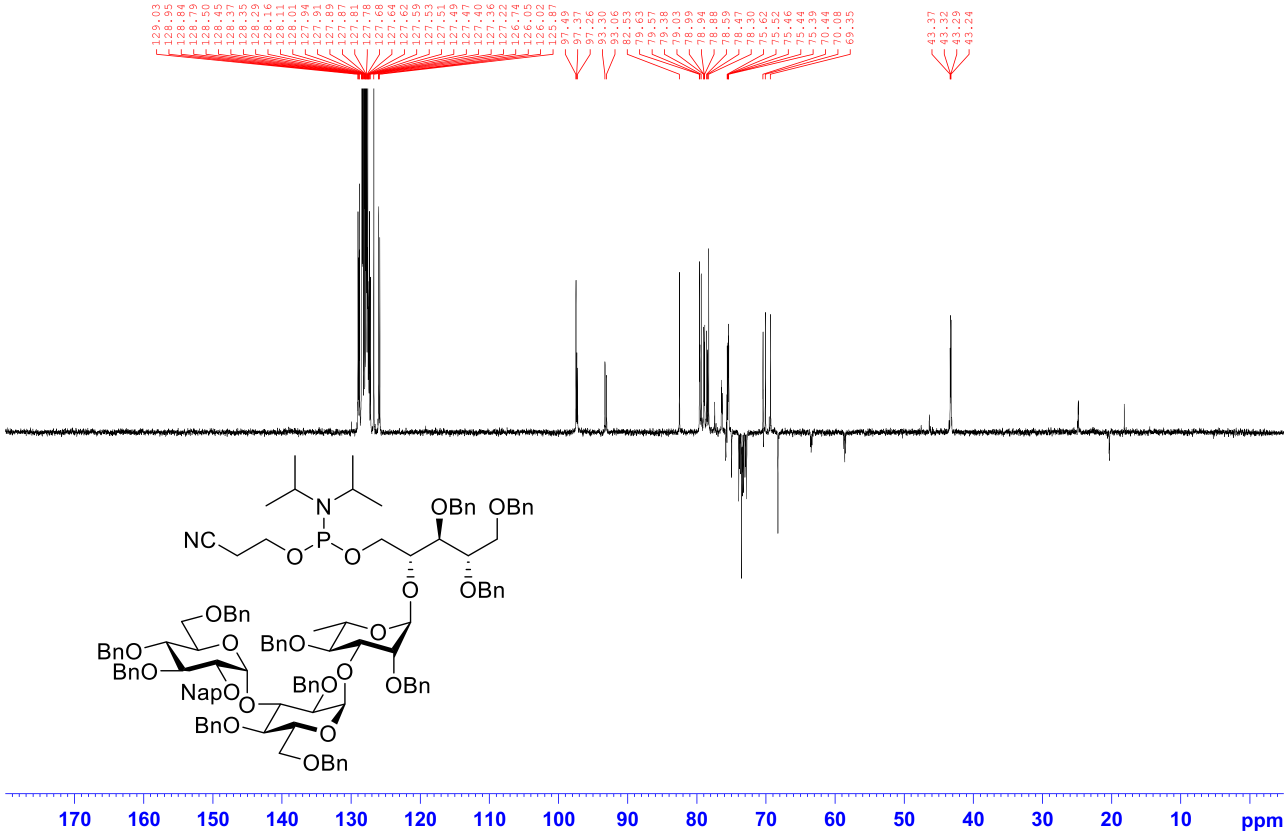

S22: ^{31}P NMR (202 MHz, $CDCl_3$)

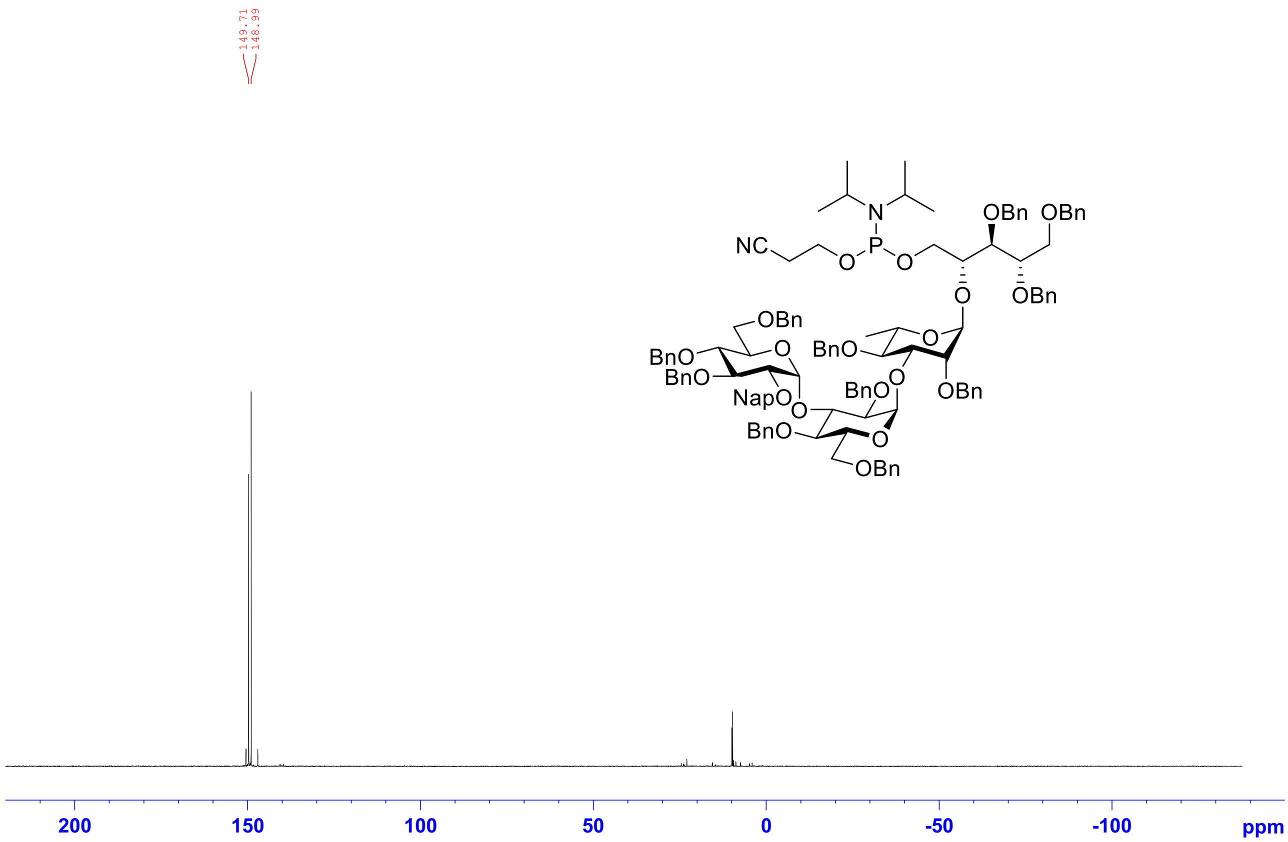

32 (Upper isomer): ^{1}H NMR (600 MHz, $CDCl_3$) (Upper isomer)

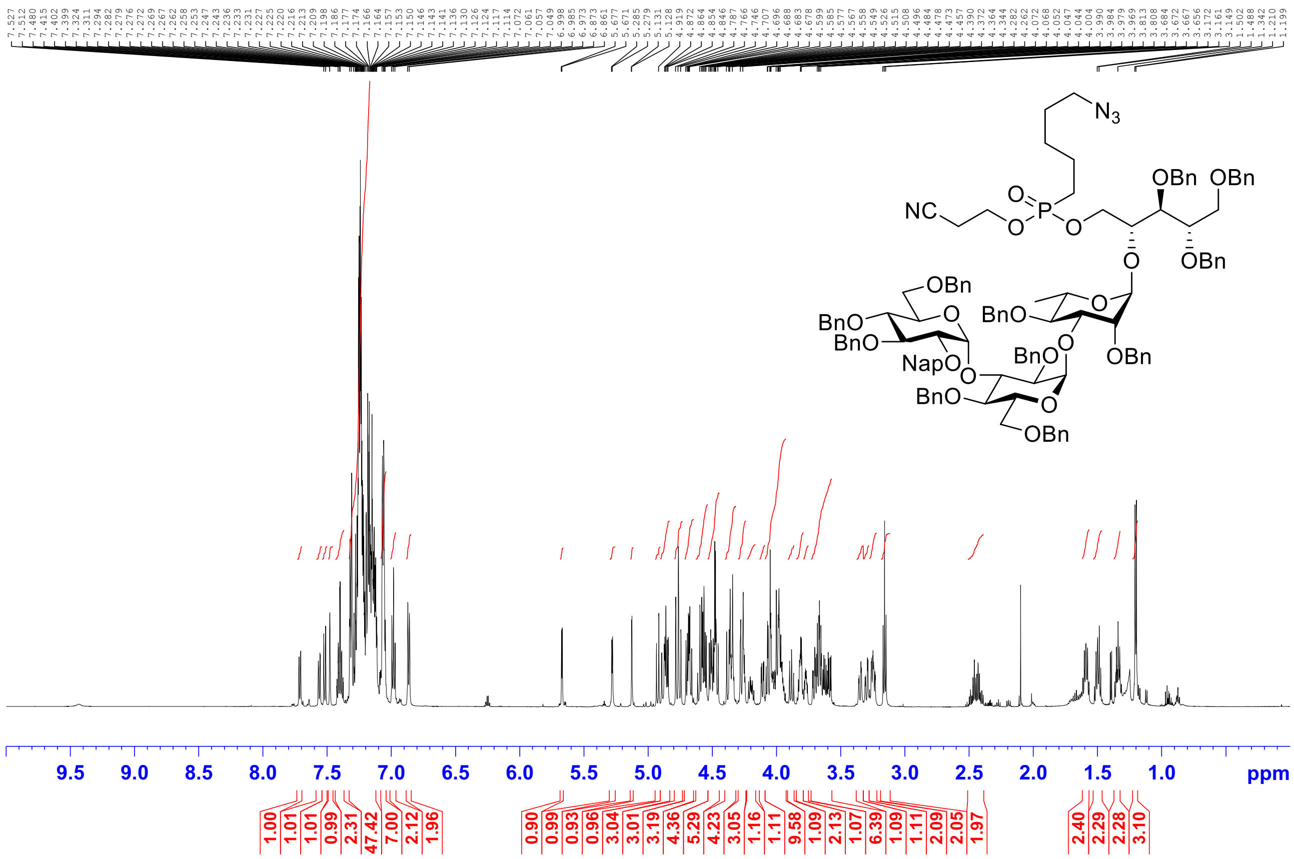

32 (Upper isomer): ^{13}C NMR (150 MHz, $CDCl_3$)

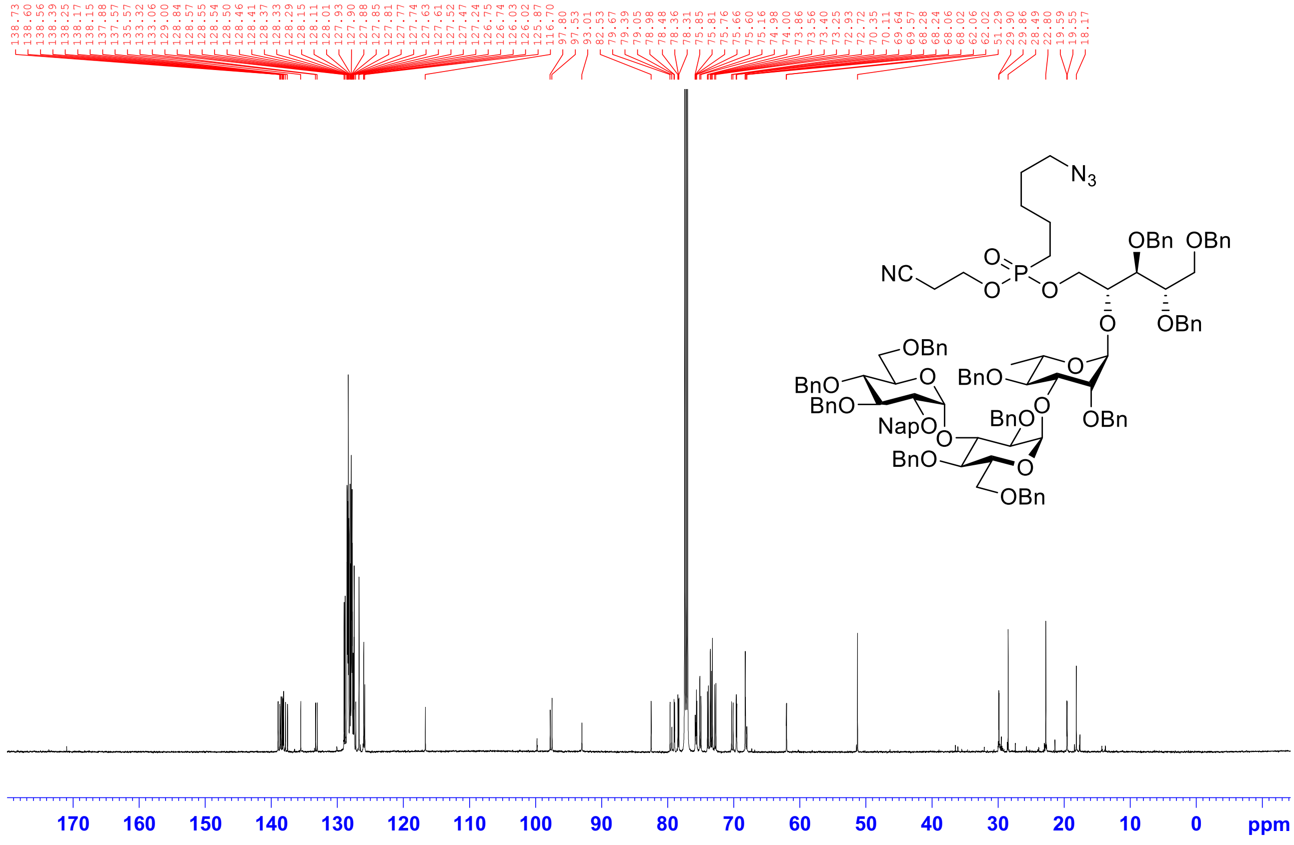

32 (Upper isomer): DEPT 135 (150 MHz, $CDCl_3$)

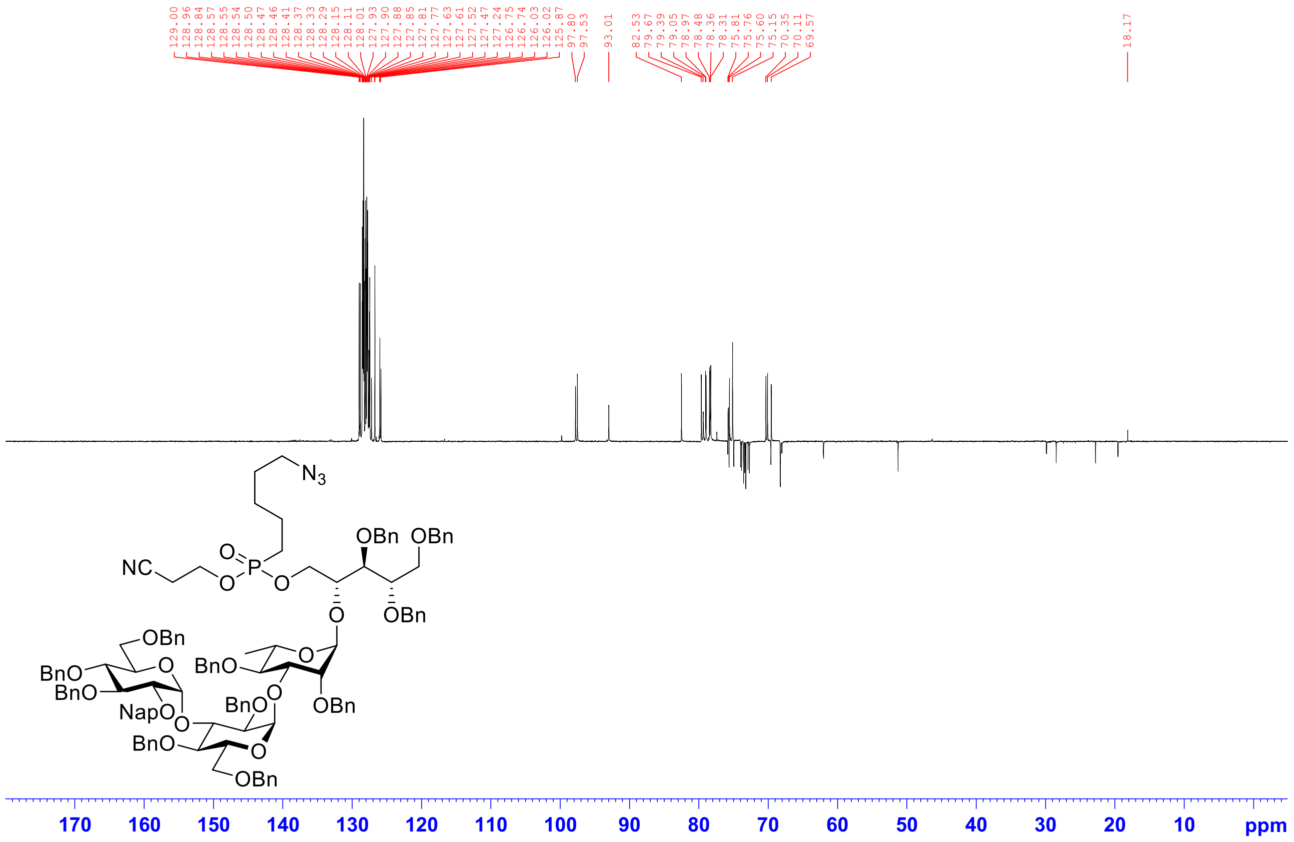

32 (Upper isomer): ^{31}P NMR (202 MHz, $CDCl_3$)

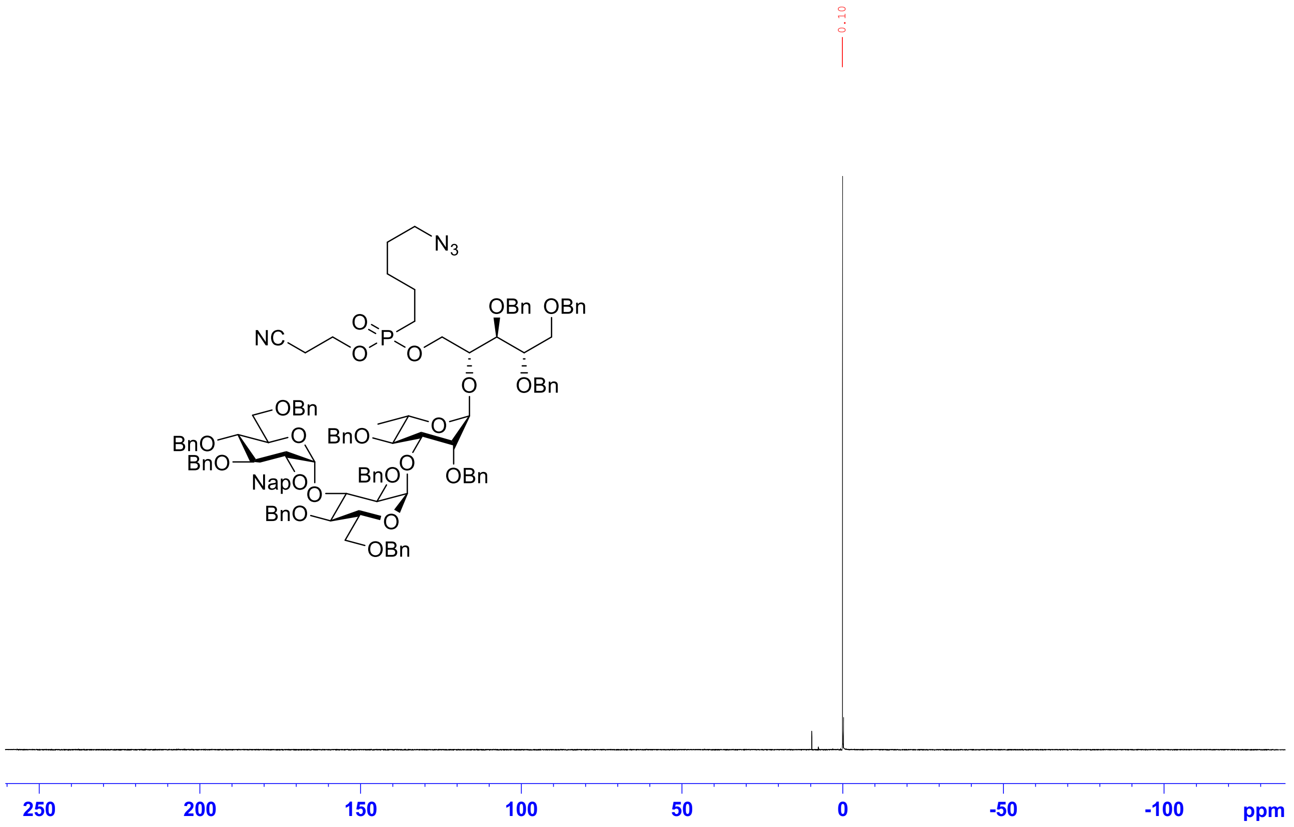

32 (Lower isomer): ^{1}H NMR (600 MHz, $CDCl_3$)

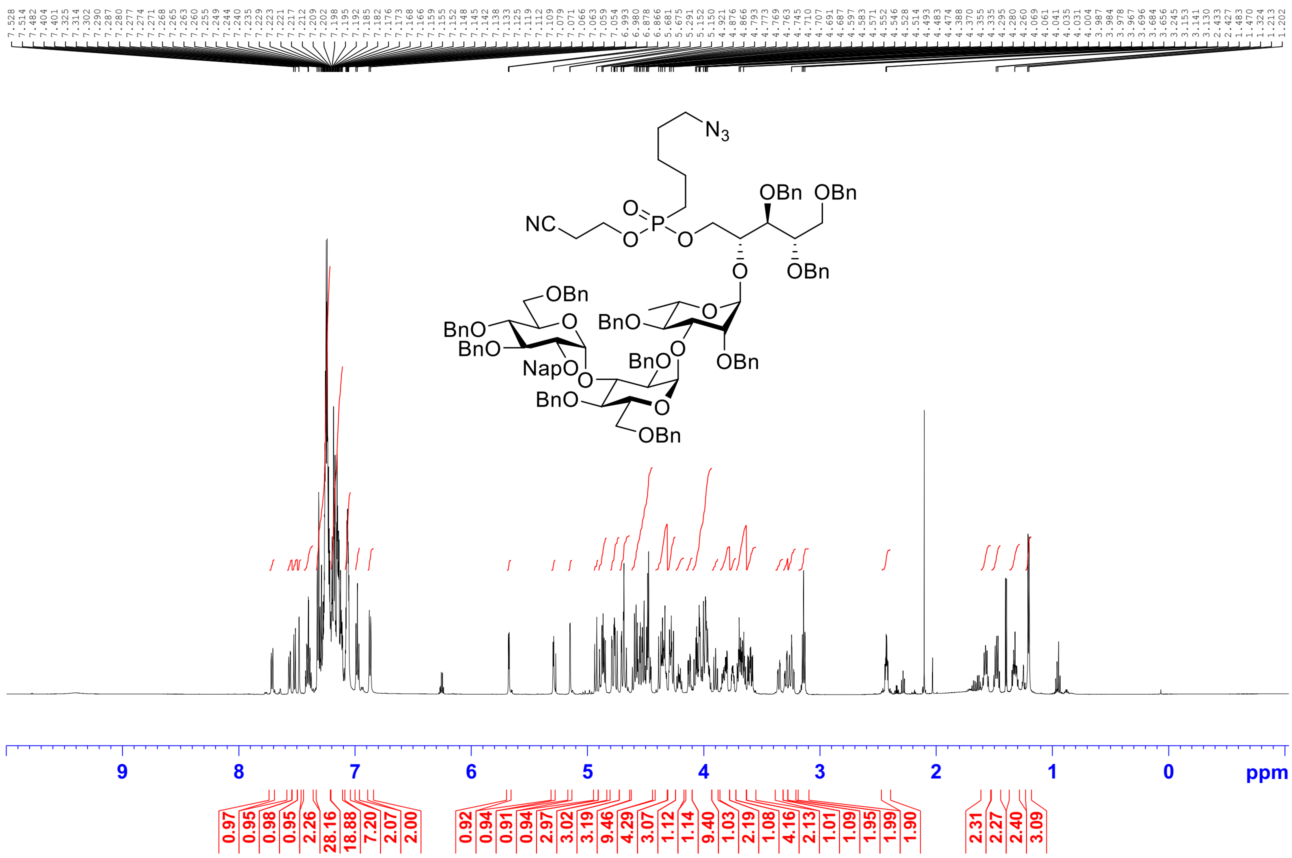

32 (Lower isomer): ^{13}C NMR (150 MHz, $CDCl_3$)

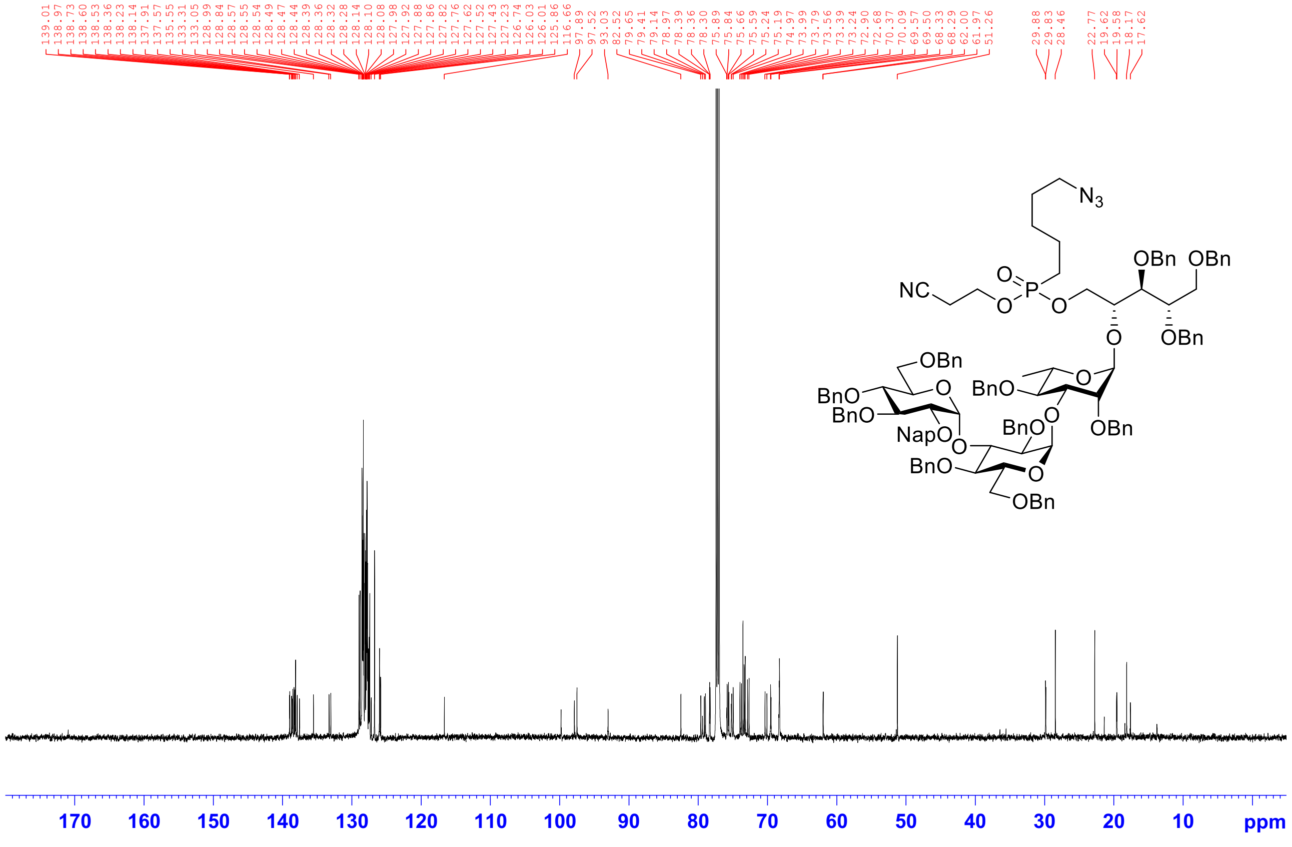

32 (Lower isomer): DEPT 135 (150 MHz, CDCl3)

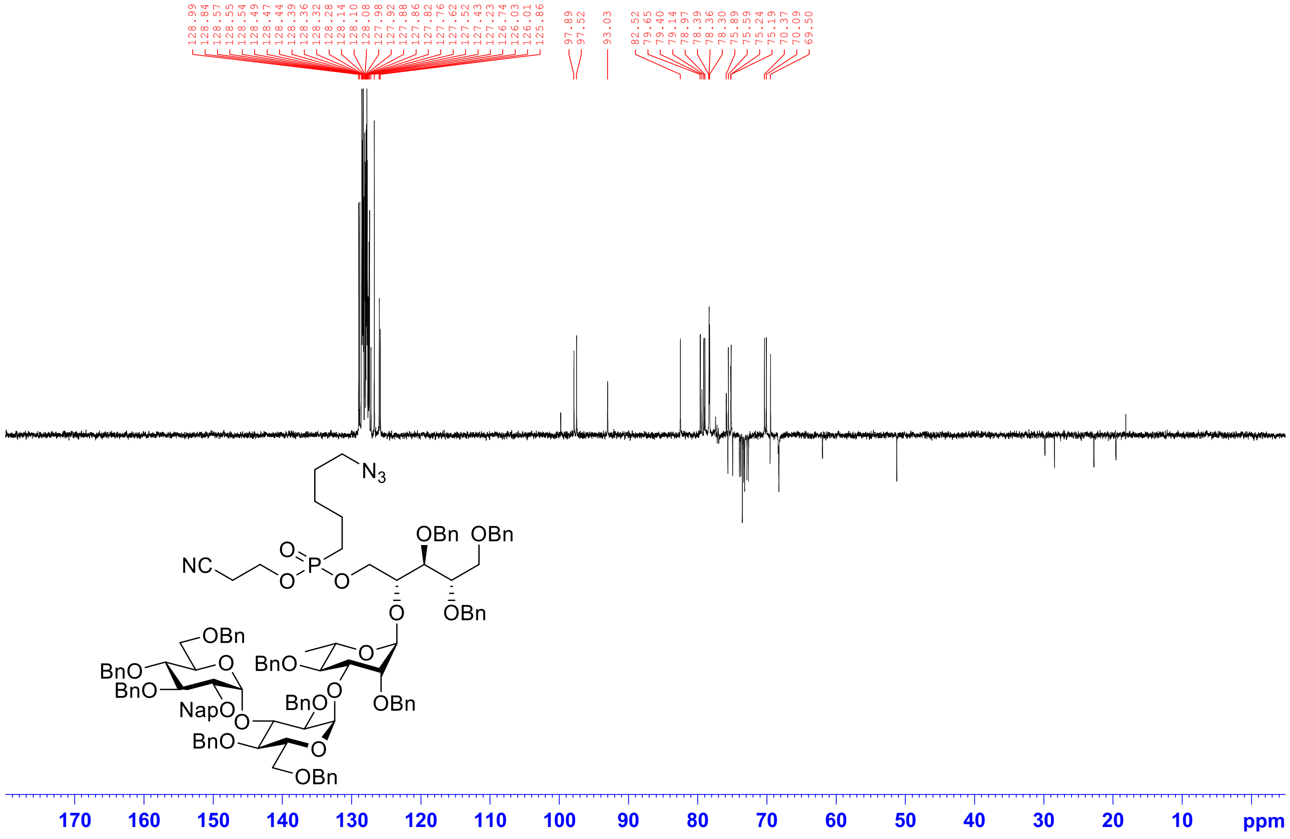

32 (Lower isomer): ^{31}P NMR (202 MHz, CDCl3)

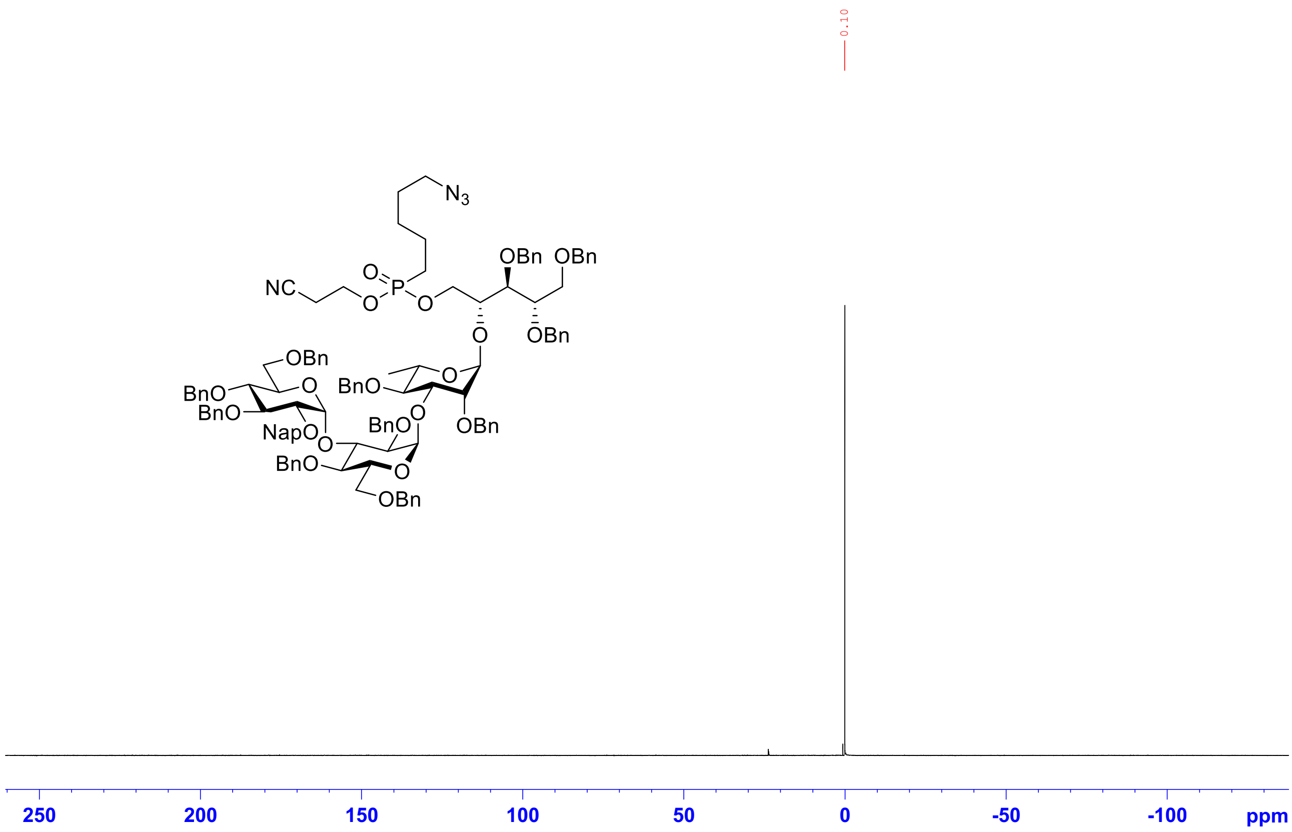

38 (Upper isomer): ^{1}H NMR (600 MHz, $CDCl_3$)

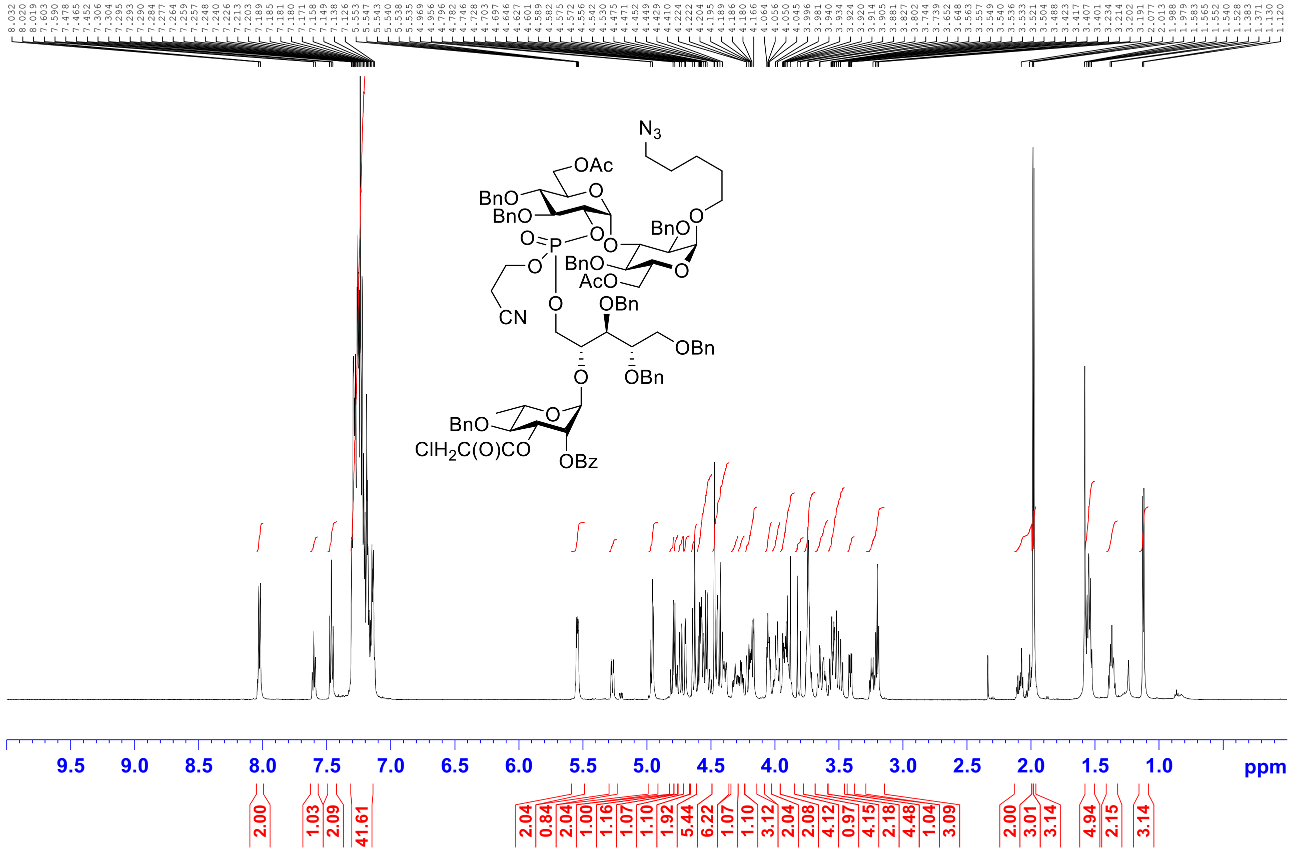

38 (Upper isomer): ^{13}C NMR (150 MHz, $CDCl_3$)

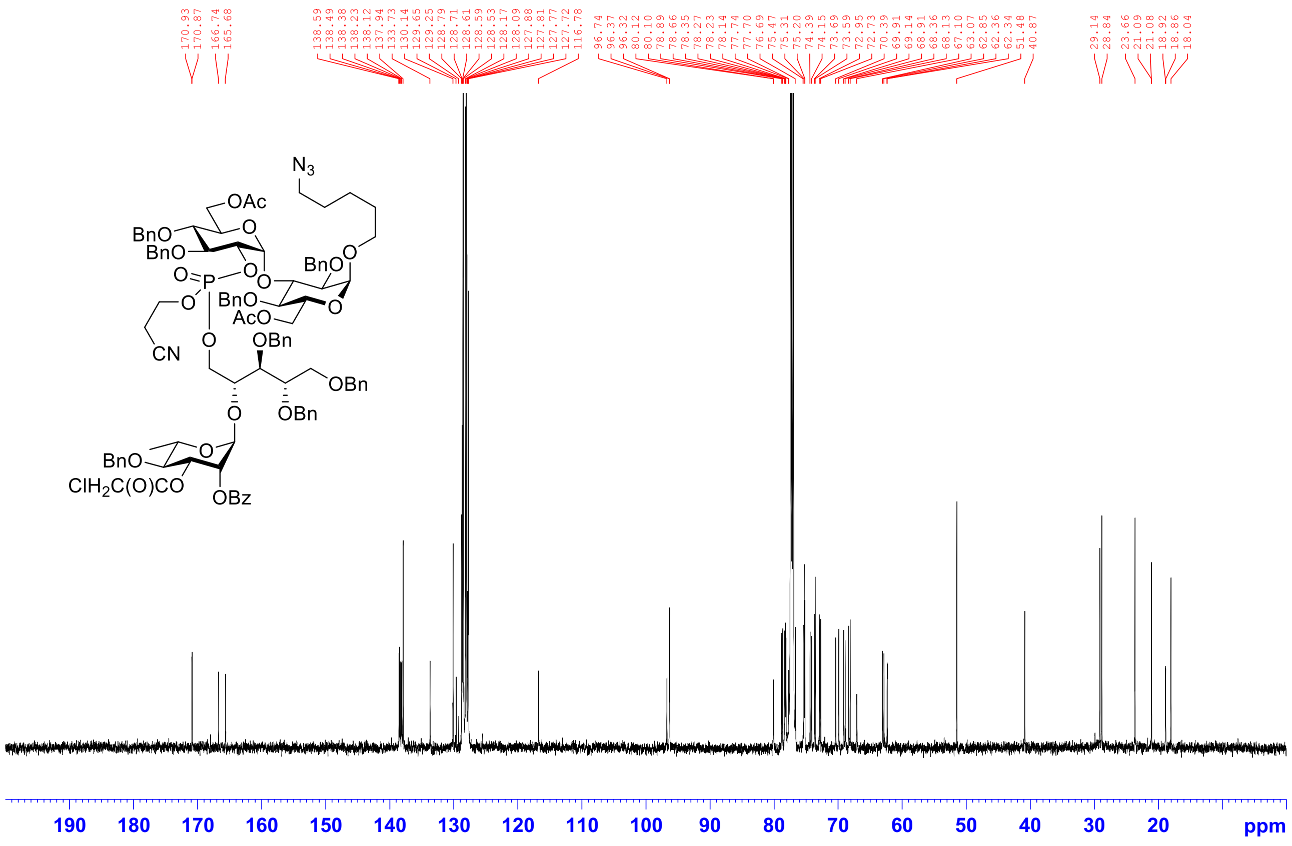

38 (Upper isomer): DEPT 135 (150 MHz, $CDCl_3$)

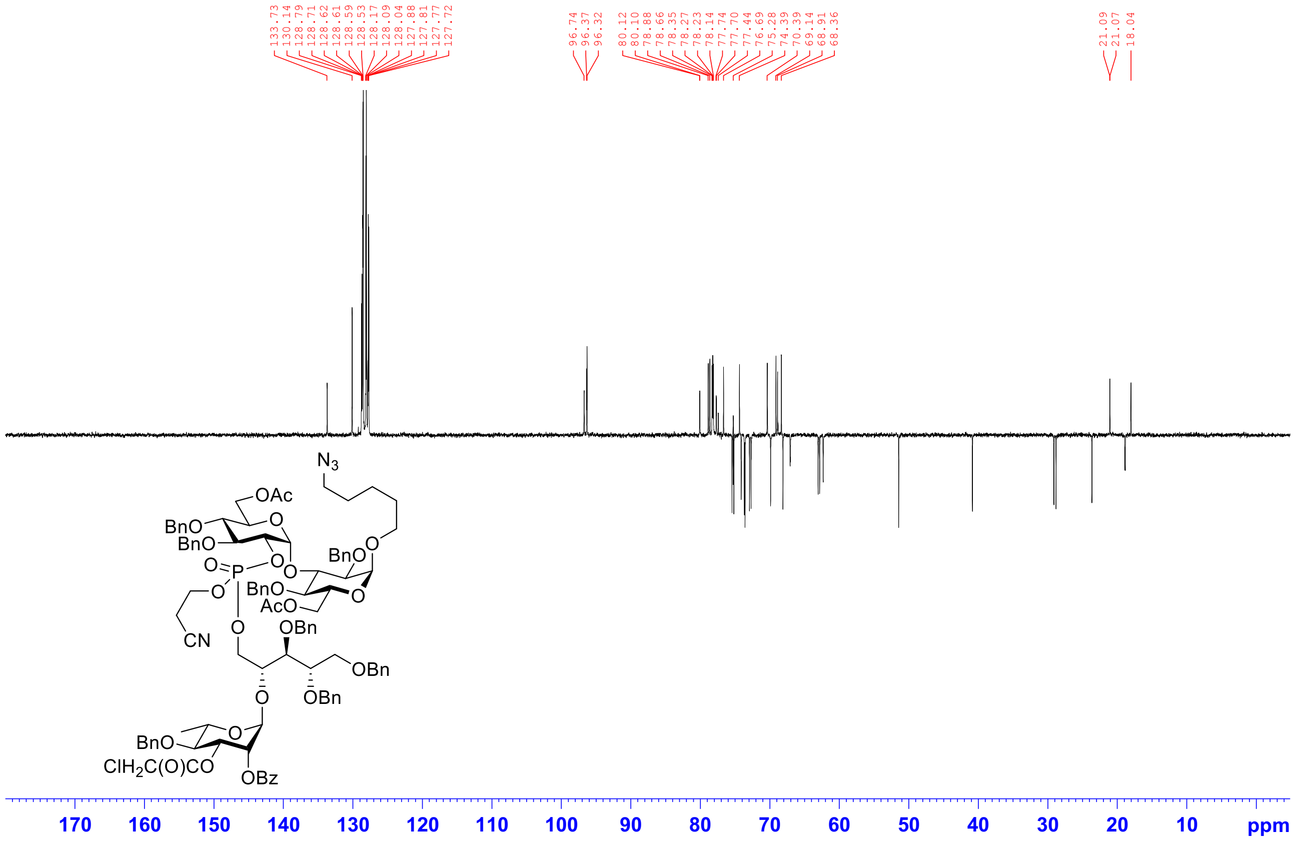

38 (Upper isomer): ^{31}P NMR (202 MHz, $CDCl_3$)

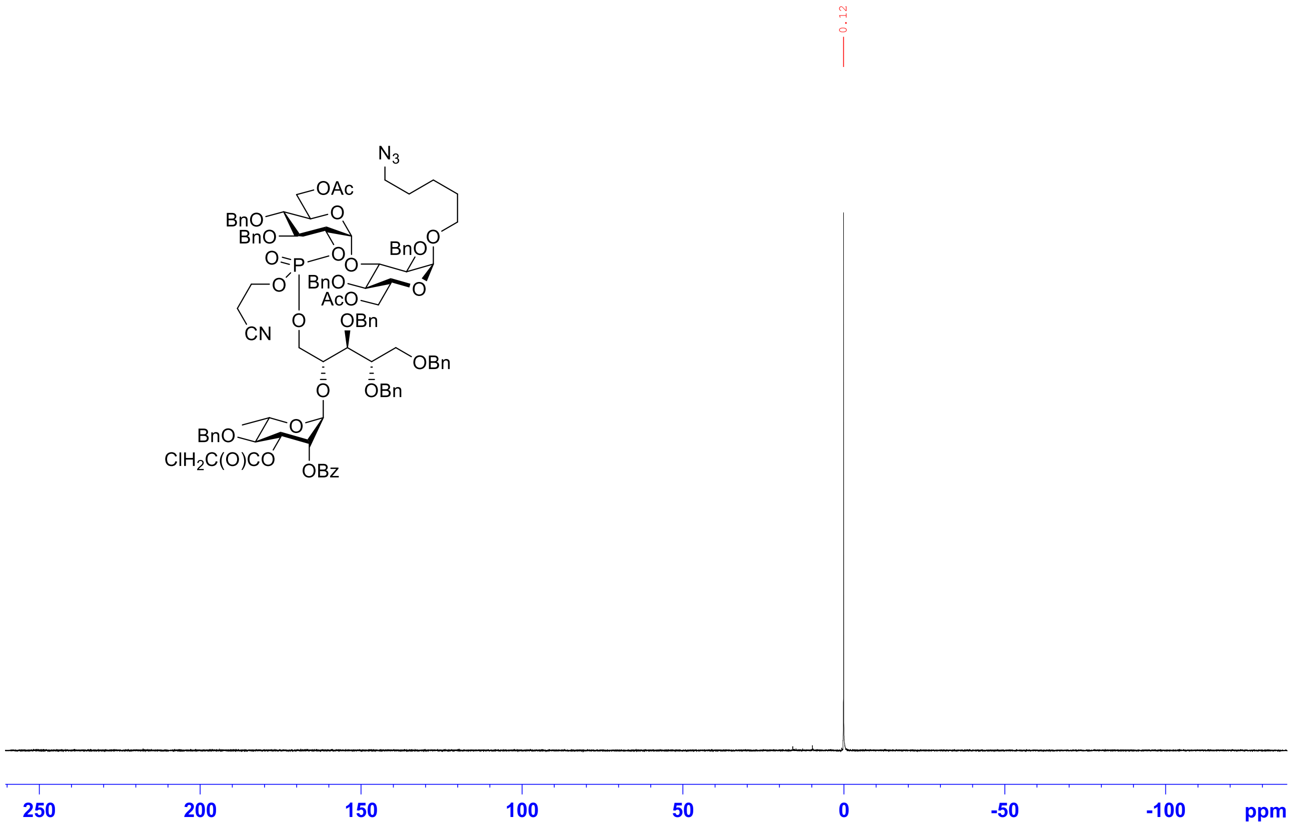

38 (Lower isomer): ^{1}H NMR (600 MHz, CDCl$_3$)

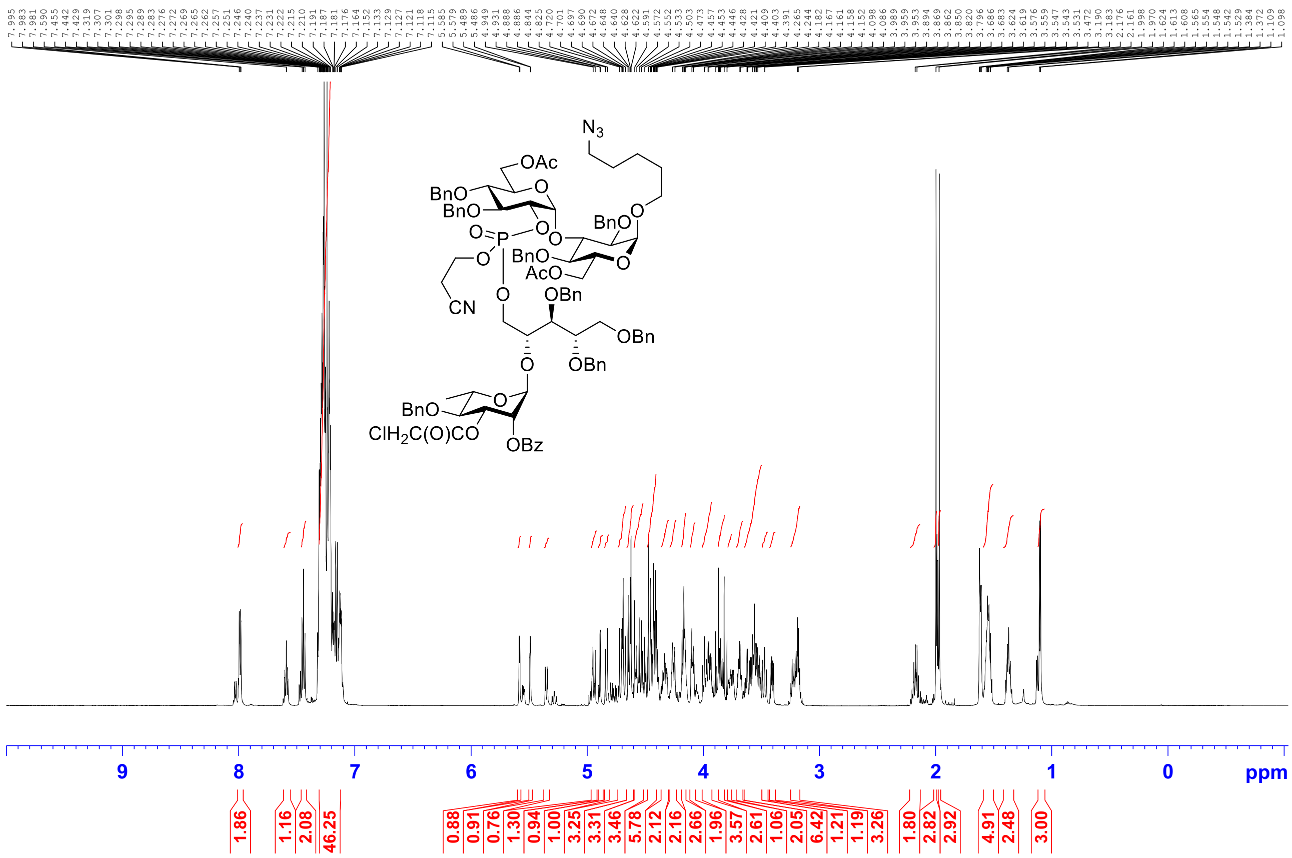

38 (Lower isomer): ^{13}C NMR (150 MHz, CDCl$_3$)

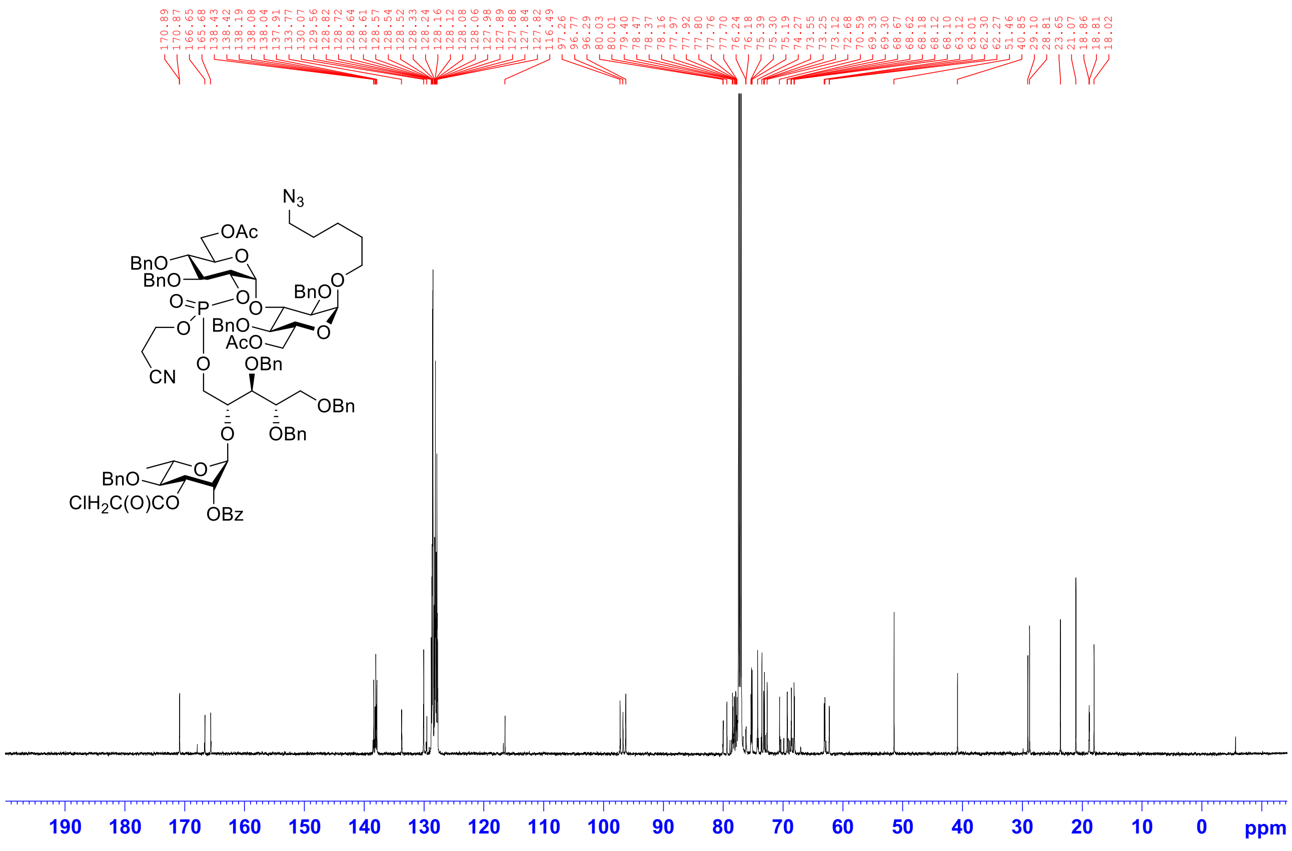

38 (Lower isomer): DEPT 135 (150 MHz, $CDCl_3$)

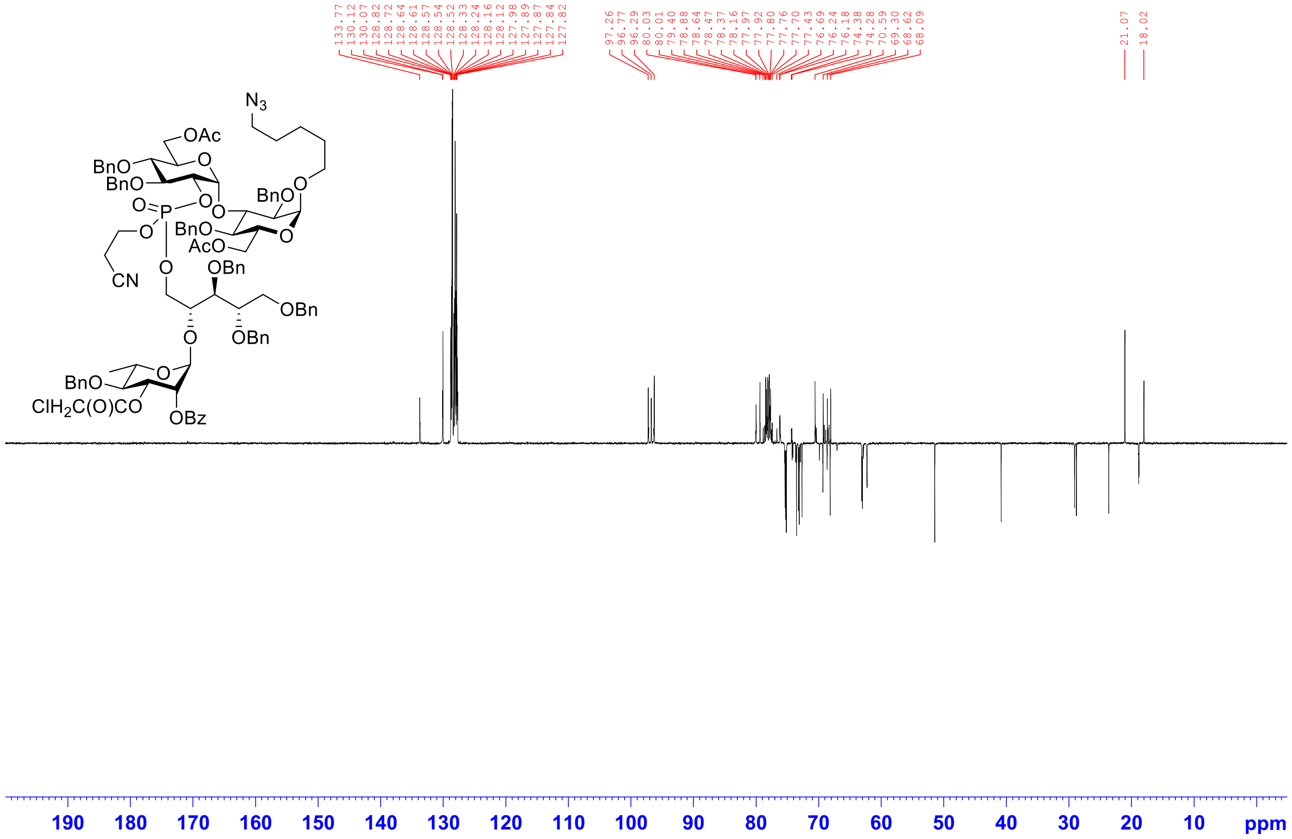

38 (Lower isomer): ^{31}P NMR (202 MHz, $CDCl_3$)

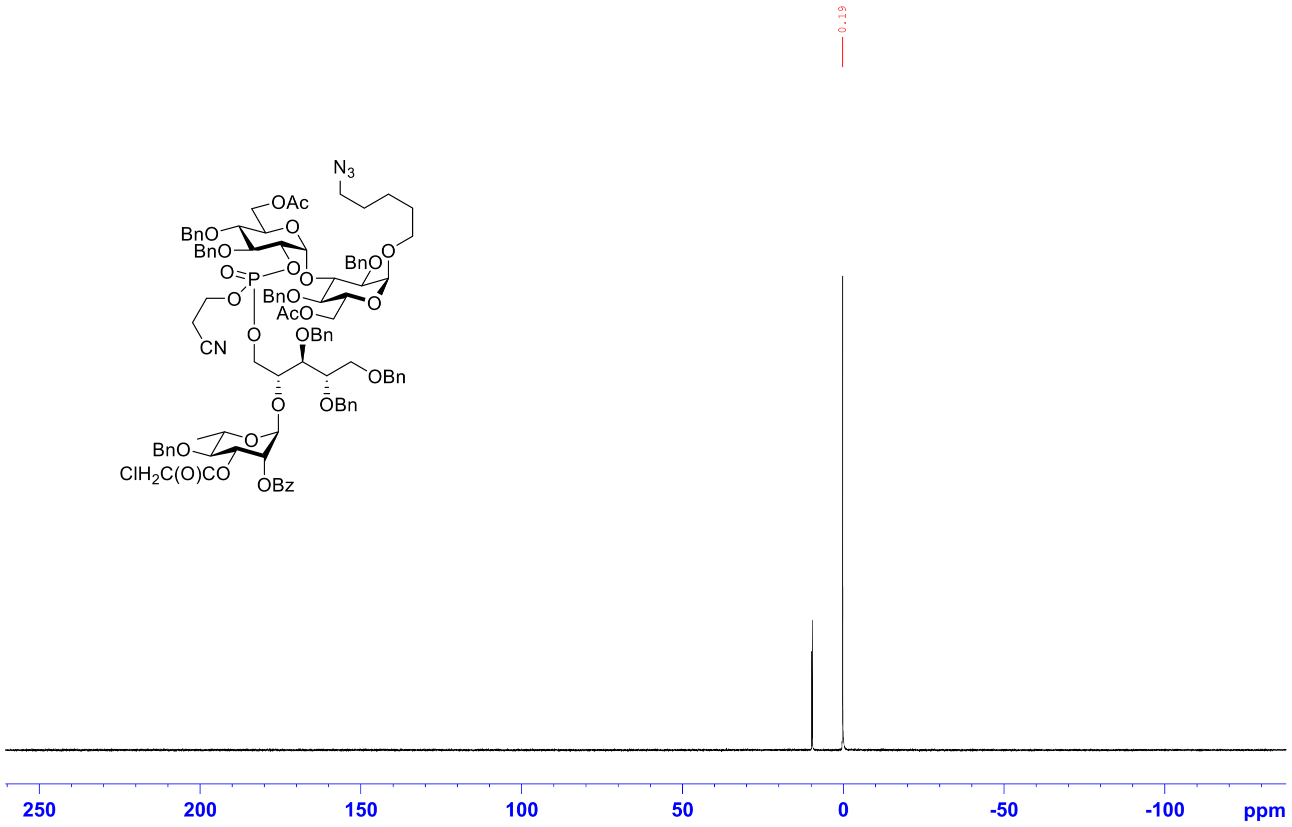

NMR spectra of common pseudo-tetrasaccharide synthesis

S23: ^{1}H NMR (600 MHz, $CDCl_3$)

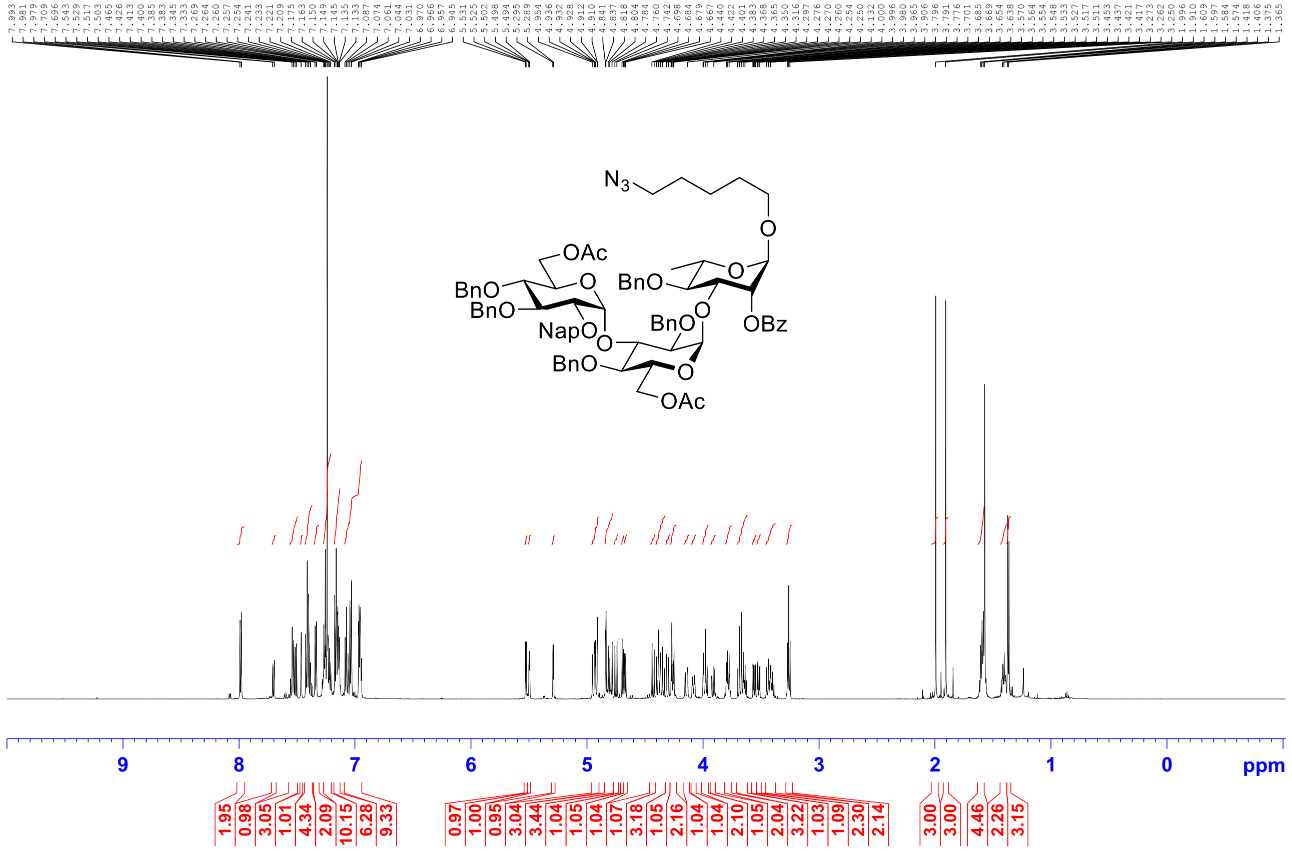

S23: ^{13}C NMR (150 MHz, $CDCl_3$)

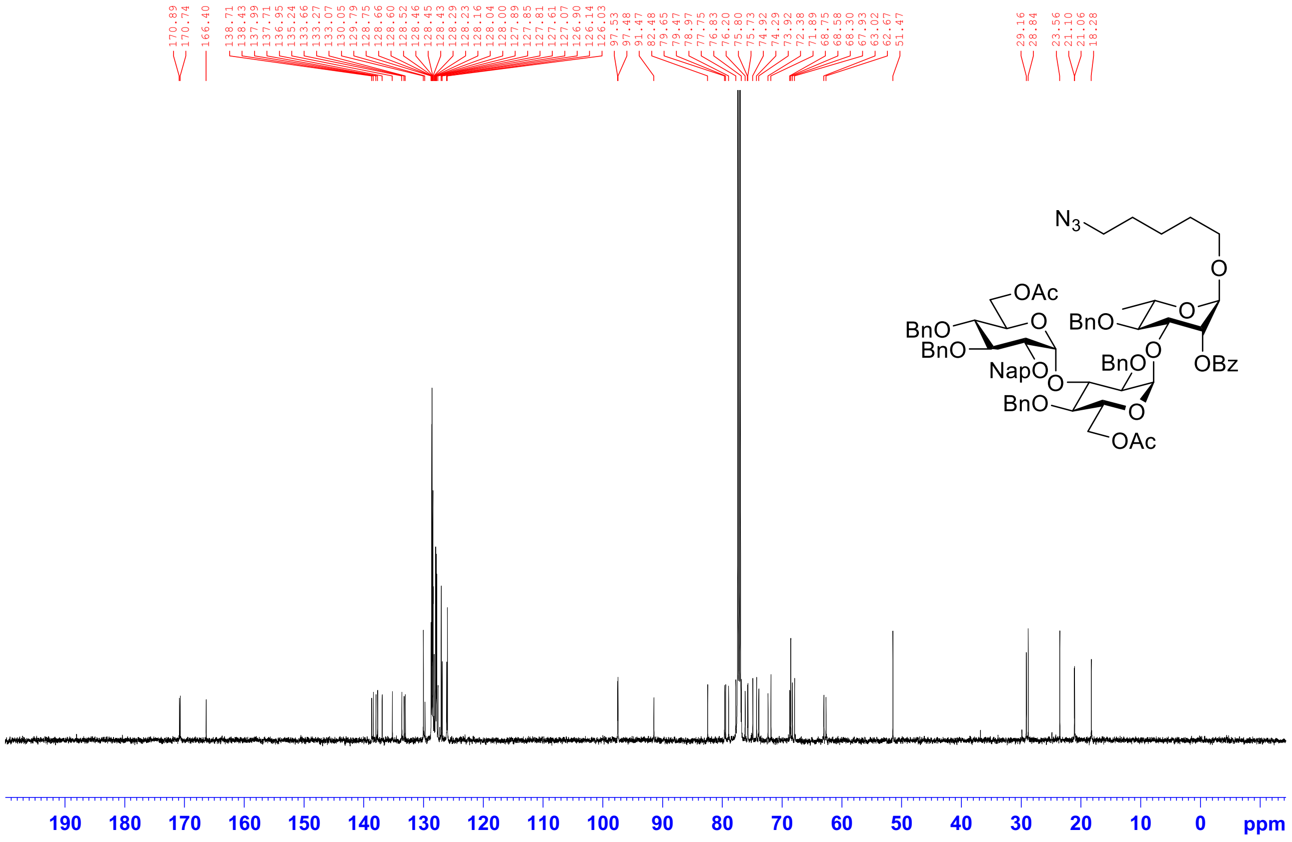

S23: DEPT 135 (150 MHz, $CDCl_3$)

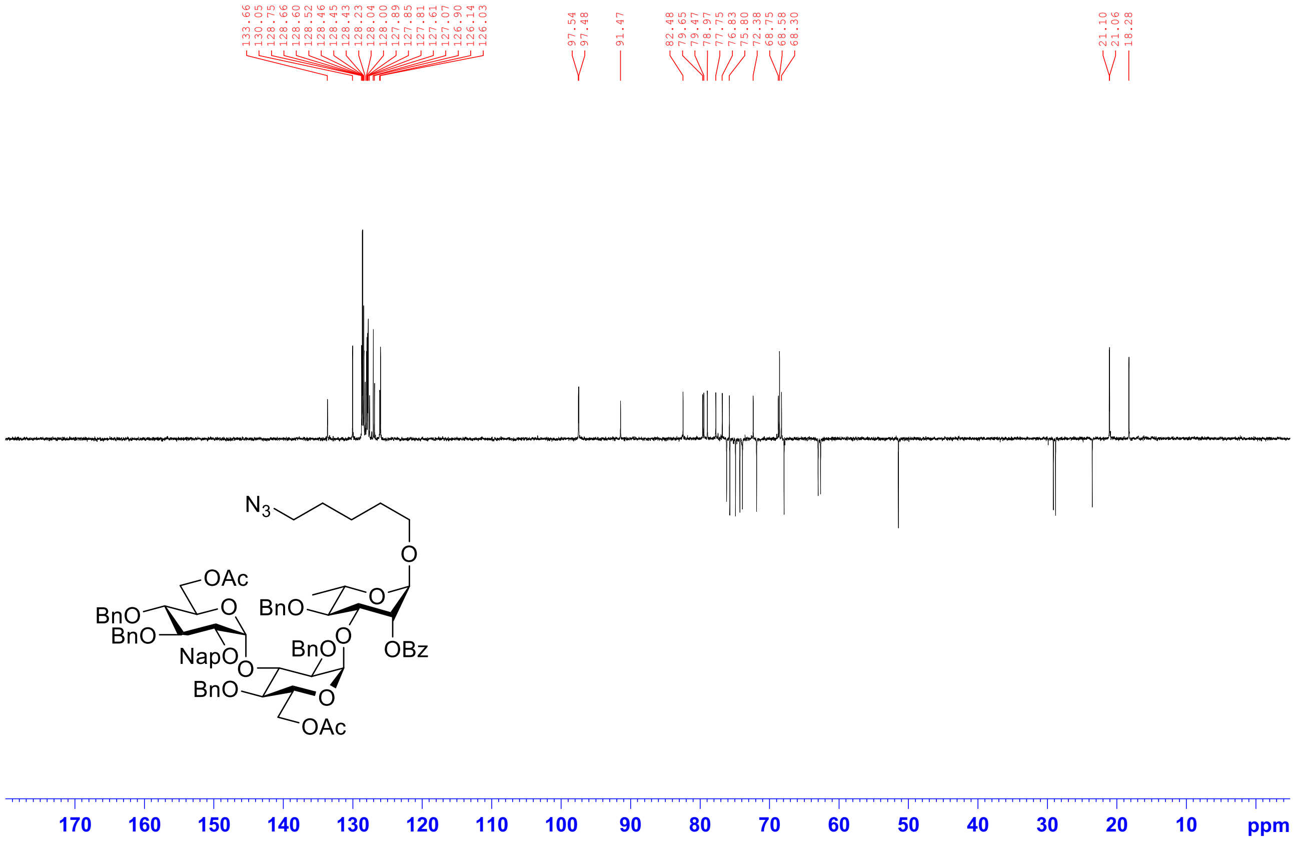

40: ^{1}H NMR (600 MHz, $CDCl_3$)

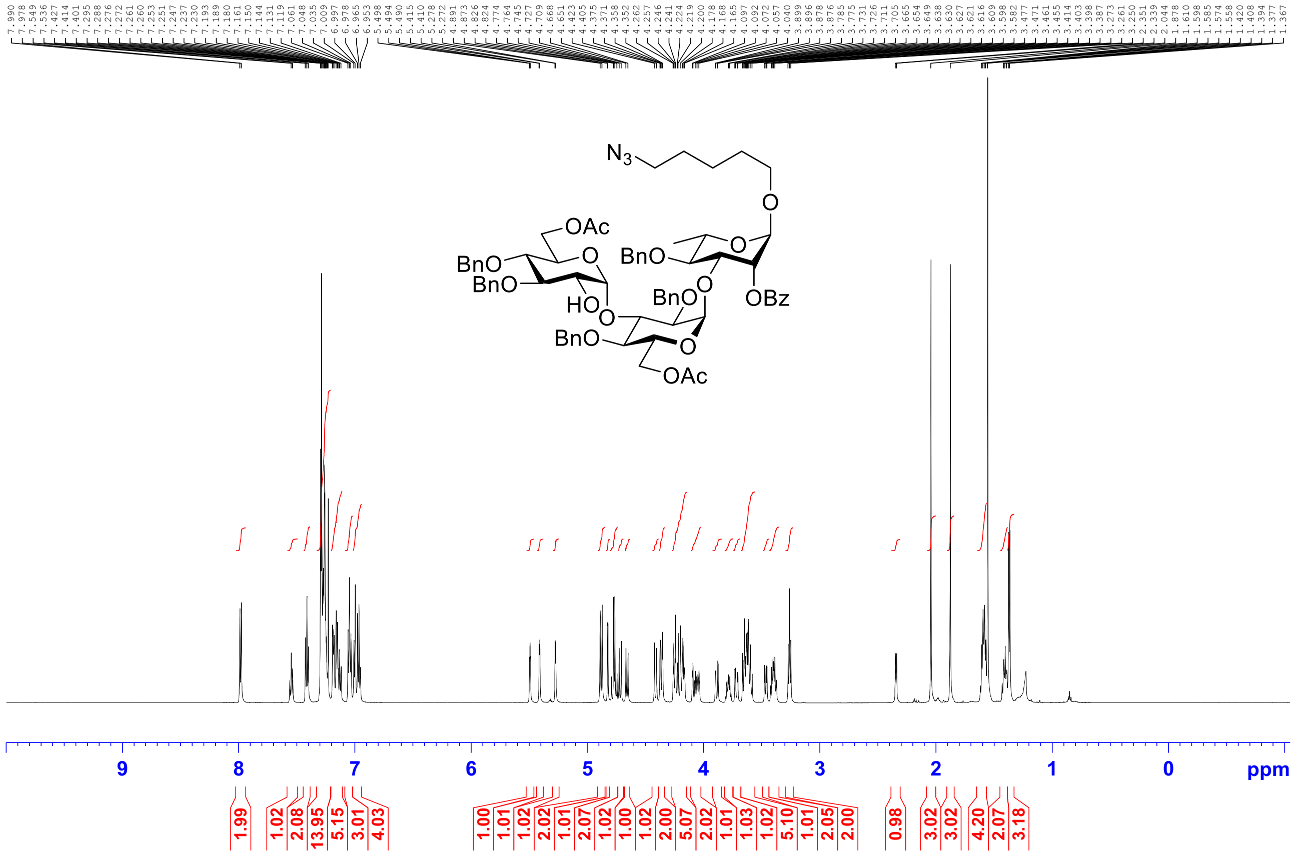

40: ^{13}C NMR (150 MHz, $CDCl_3$)

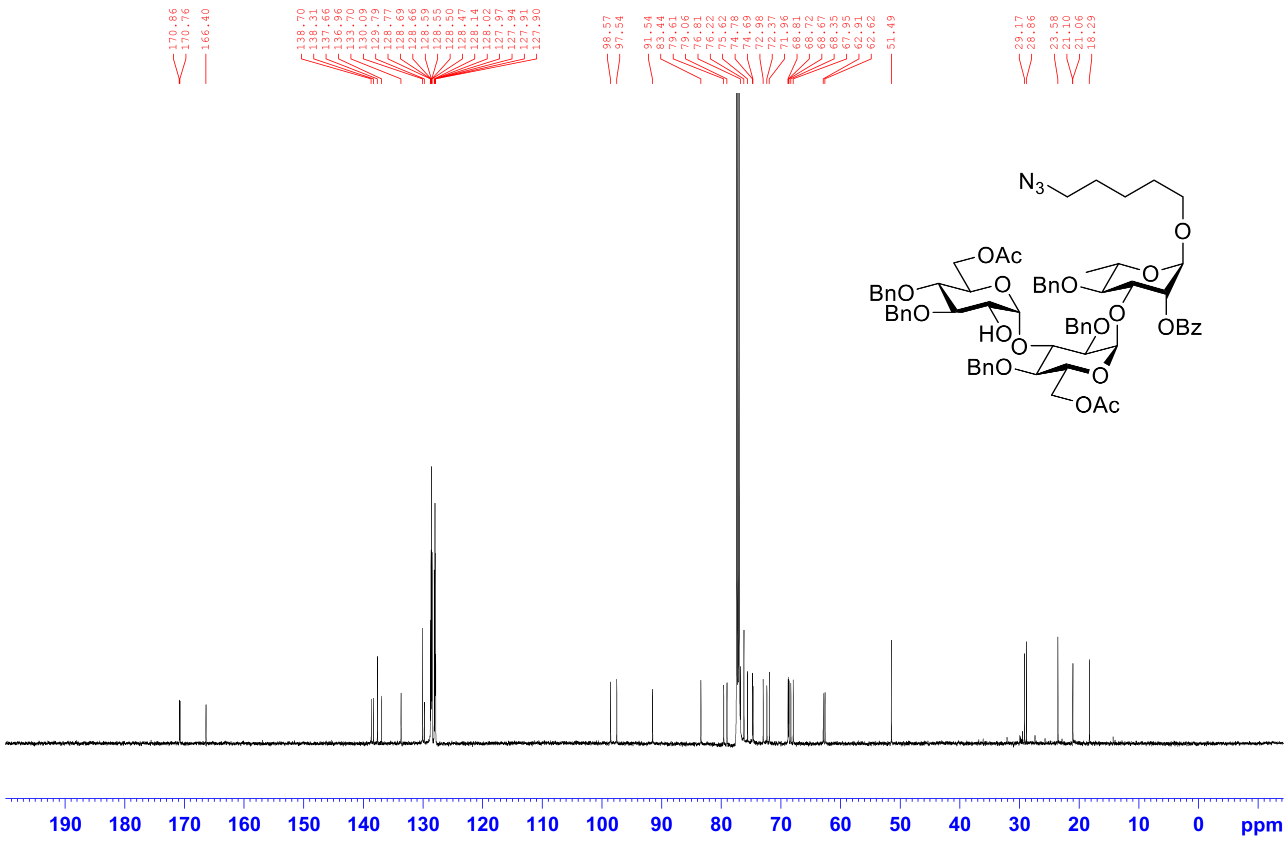

40: DEPT 135 (150 MHz, $CDCl_3$)

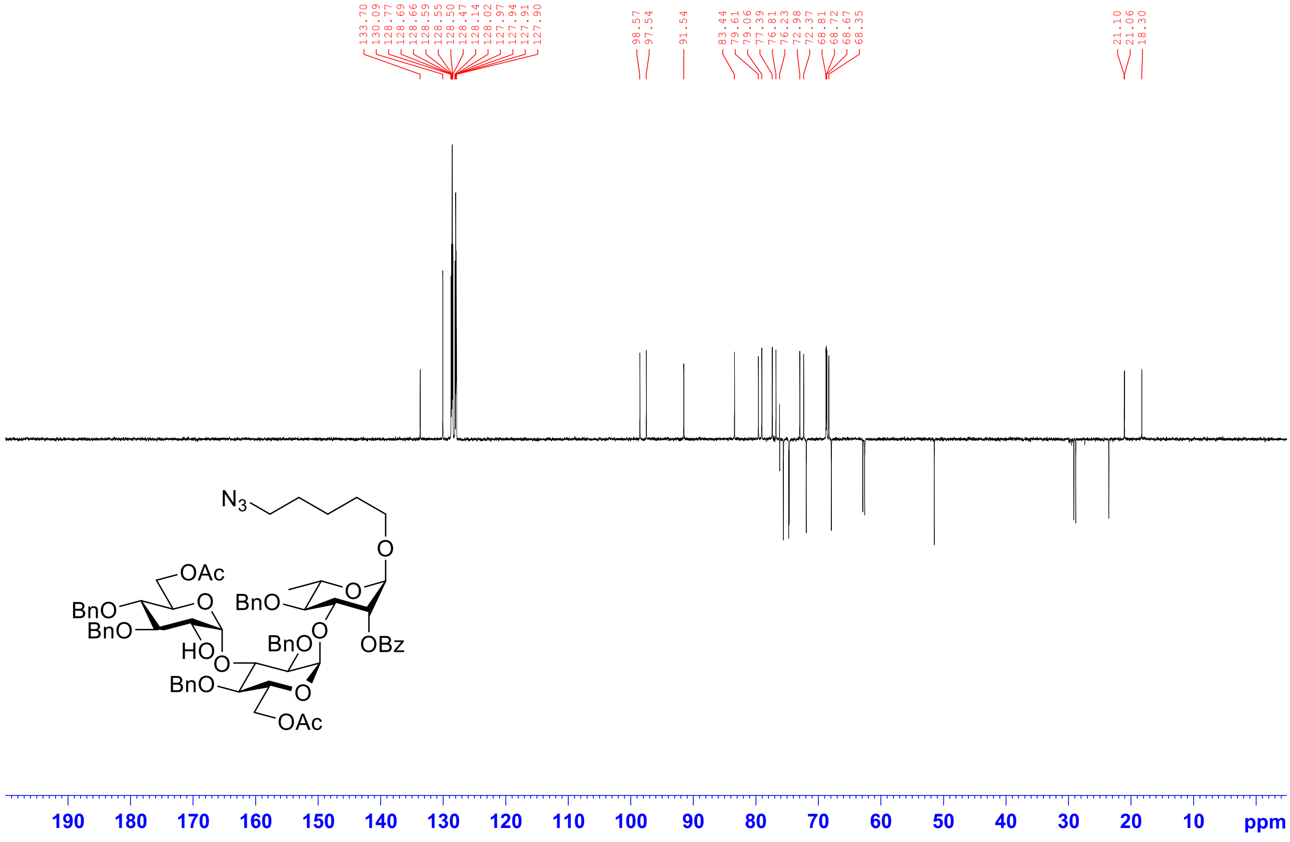

S25: ^{1}H NMR (600 MHz, $CDCl_3$)

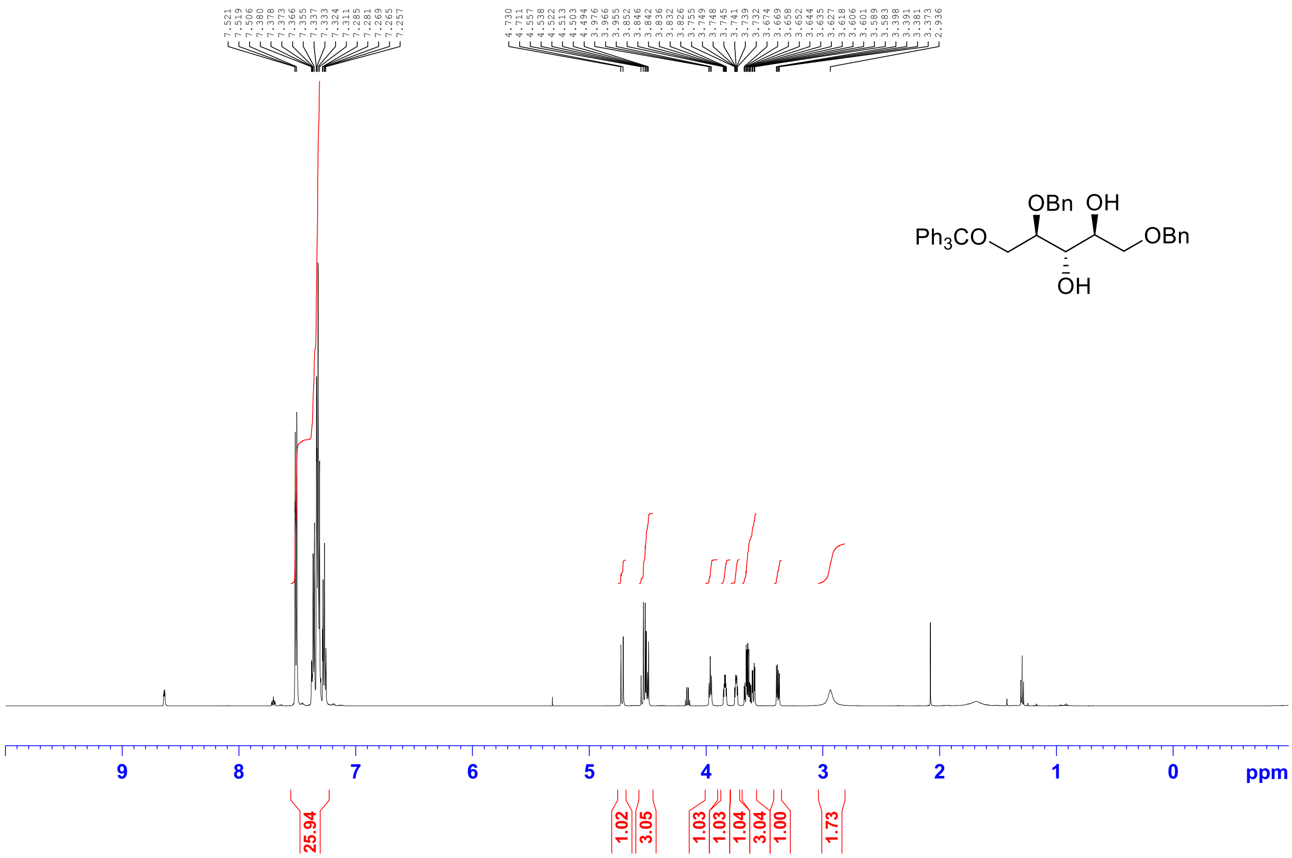

S25: ^{13}C NMR (150 MHz, $CDCl_3$)

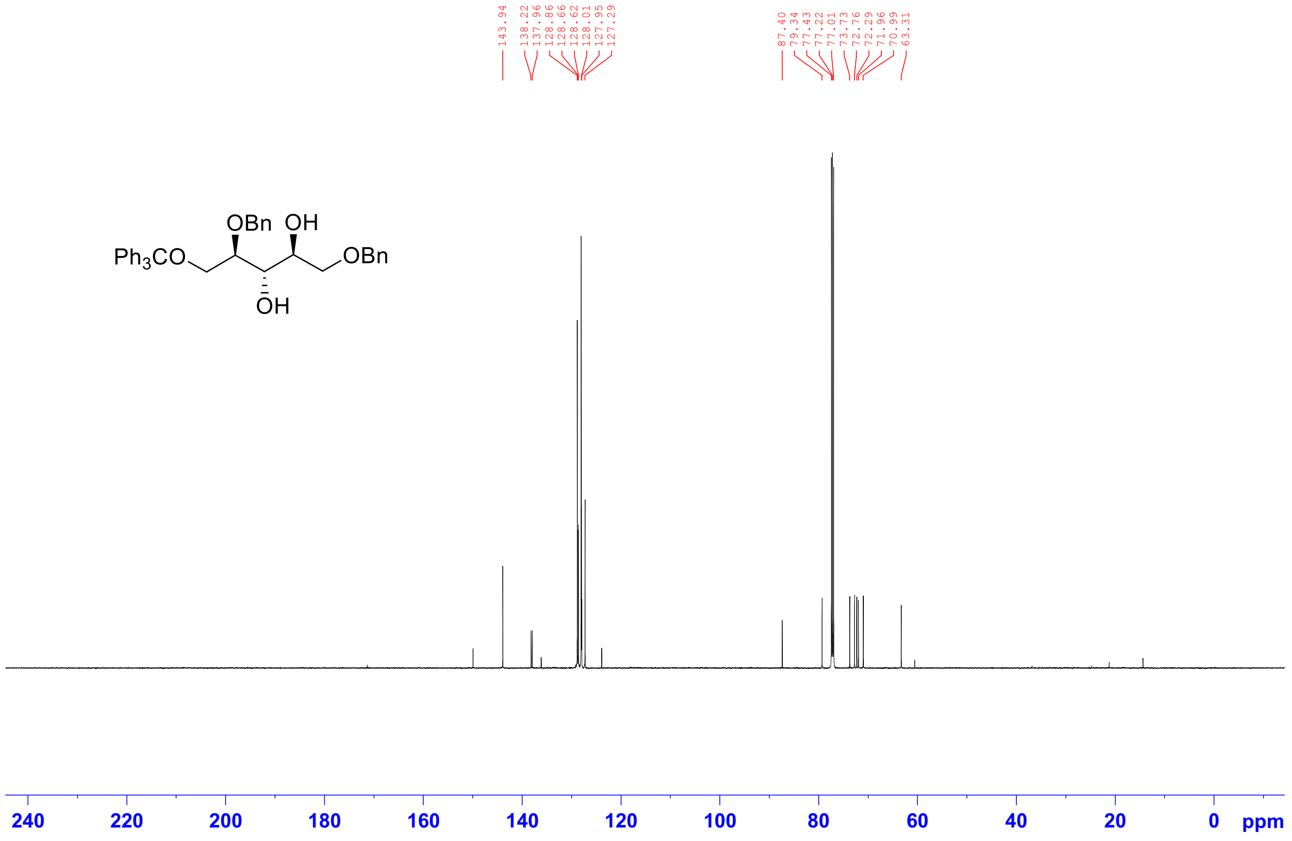

S25: DEPT 135 (150 MHz, $CDCl_3$)

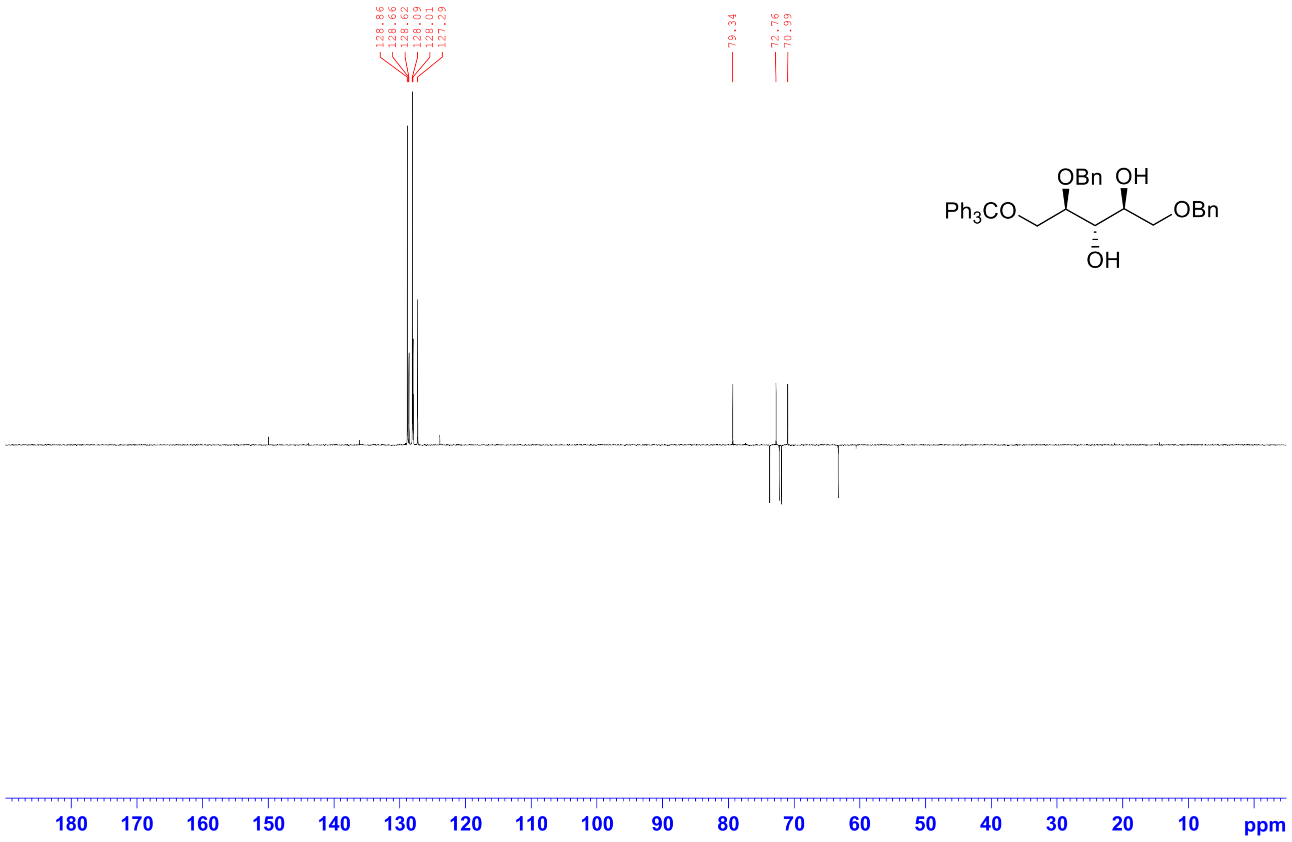

S26: ^{1}H NMR (600 MHz, $CDCl_3$)

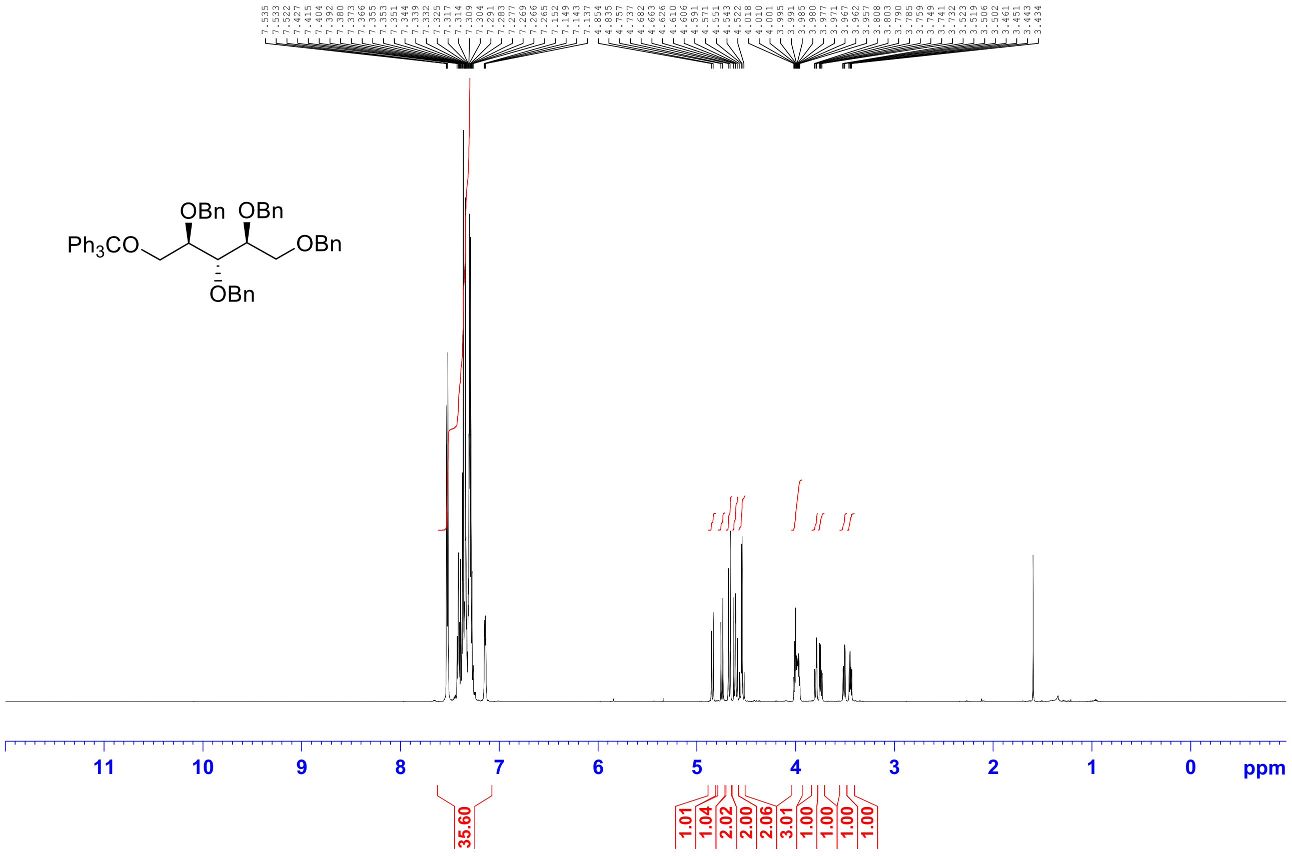

S26: ^{13}C NMR (150 MHz, CDCl$_3$)

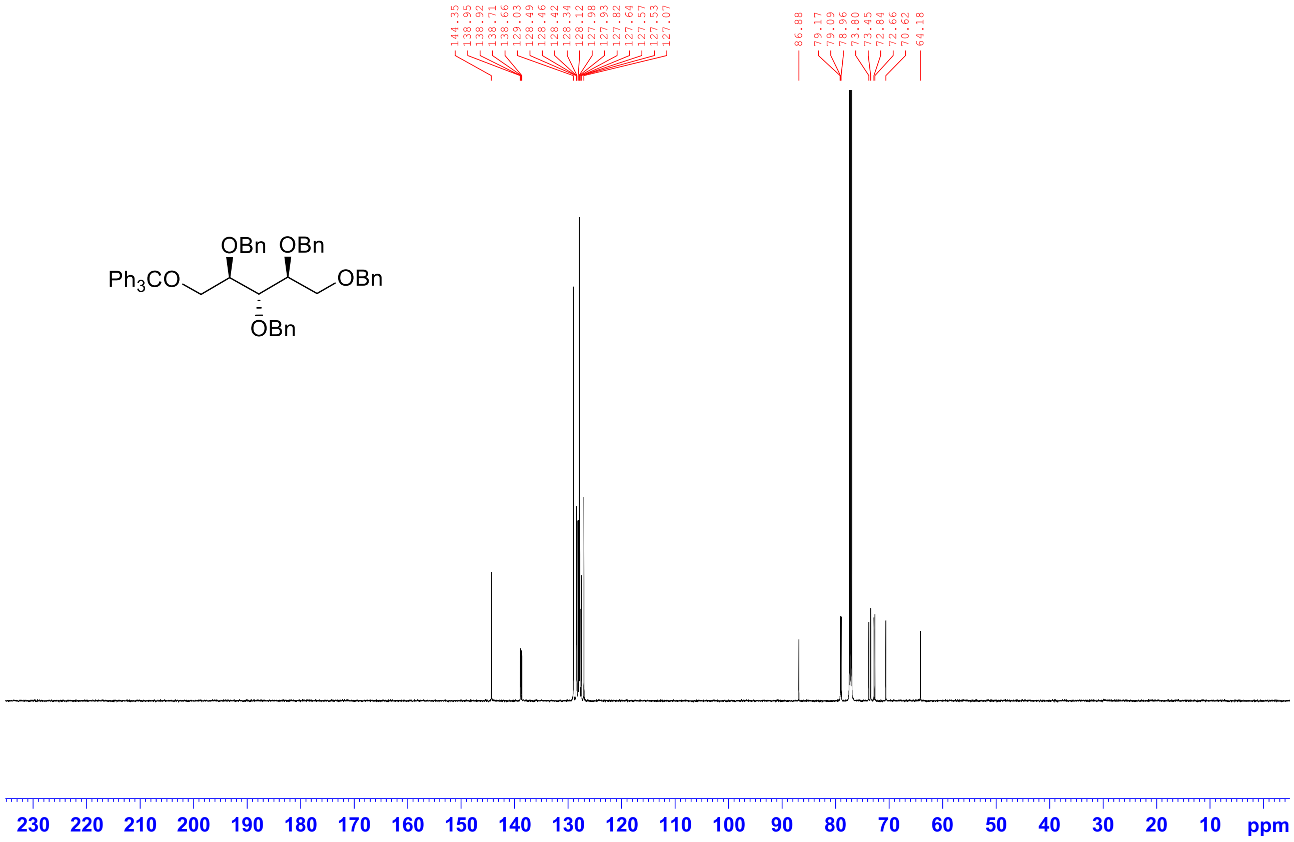

S26: DEPT 135 (150 MHz, CDCl$_3$)

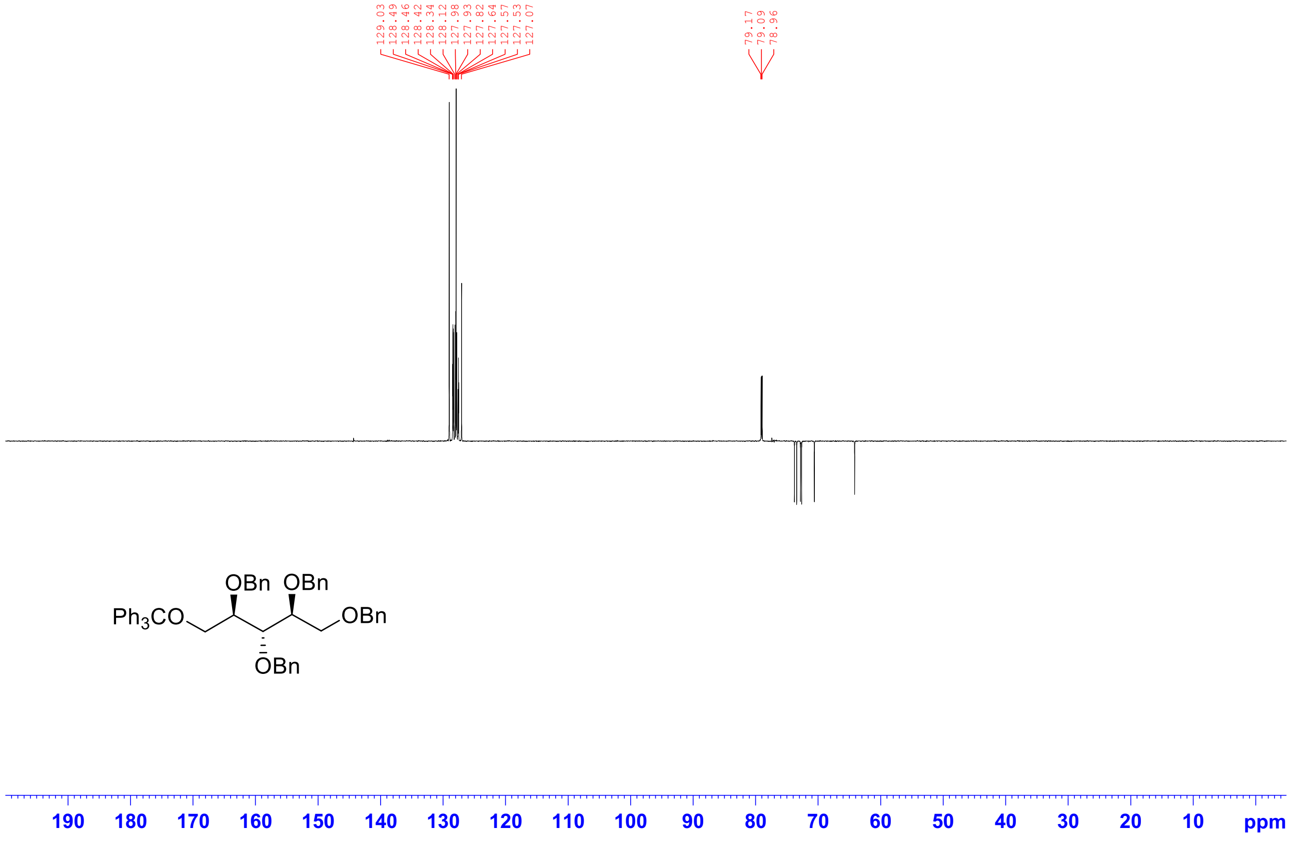

S27: ^{1}H NMR (600 MHz, CDCl$_3$)

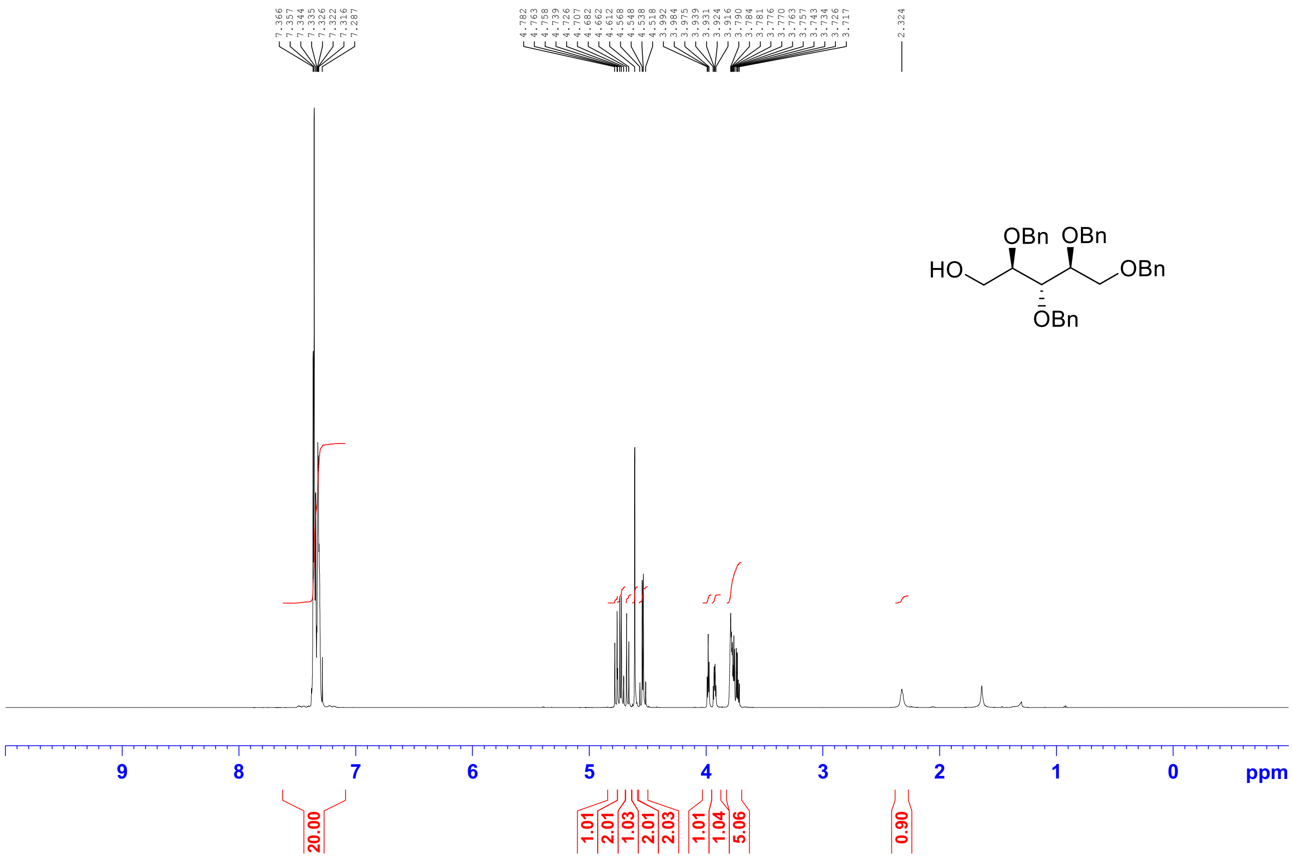

S27: ^{13}C NMR (150 MHz, CDCl$_3$)

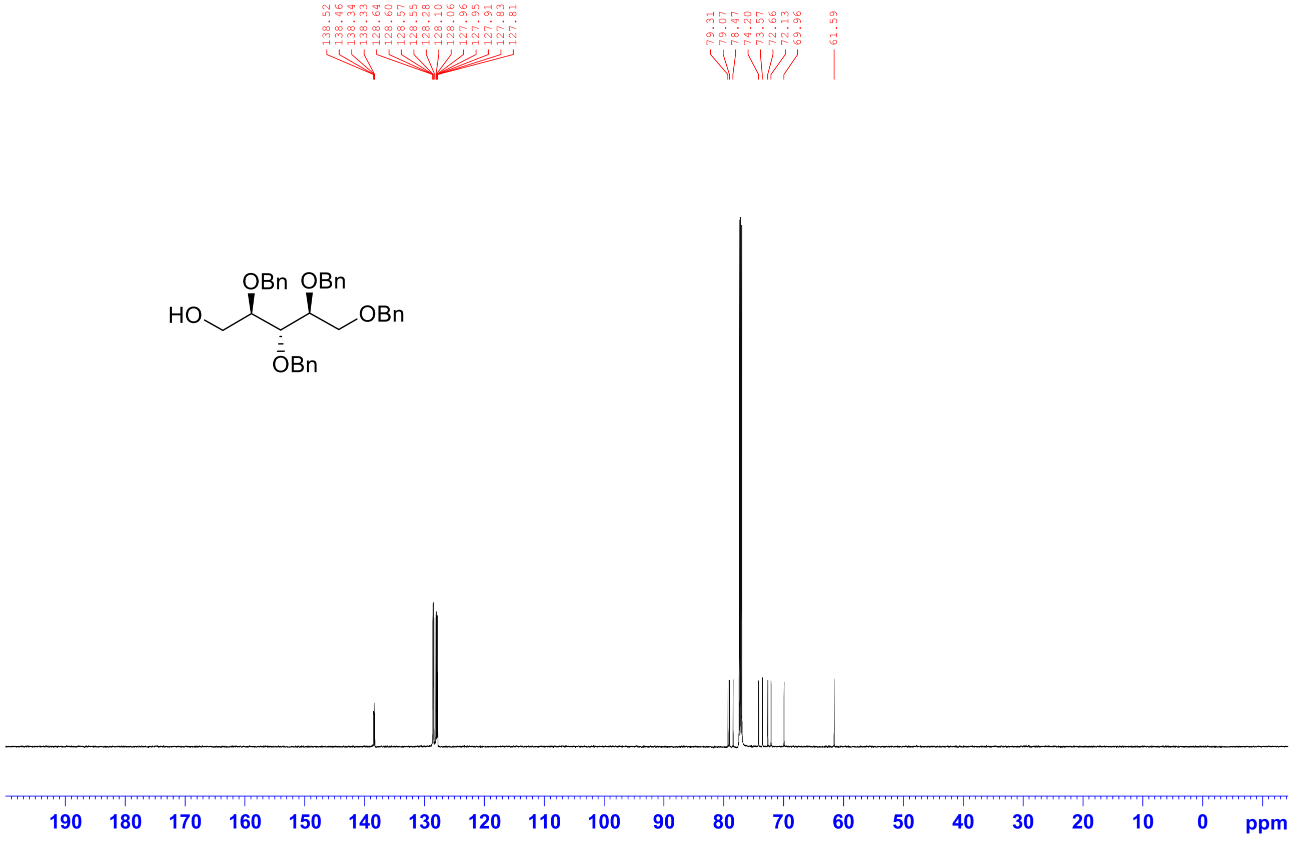

41: ^{1}H NMR (600 MHz, $CDCl_3$)

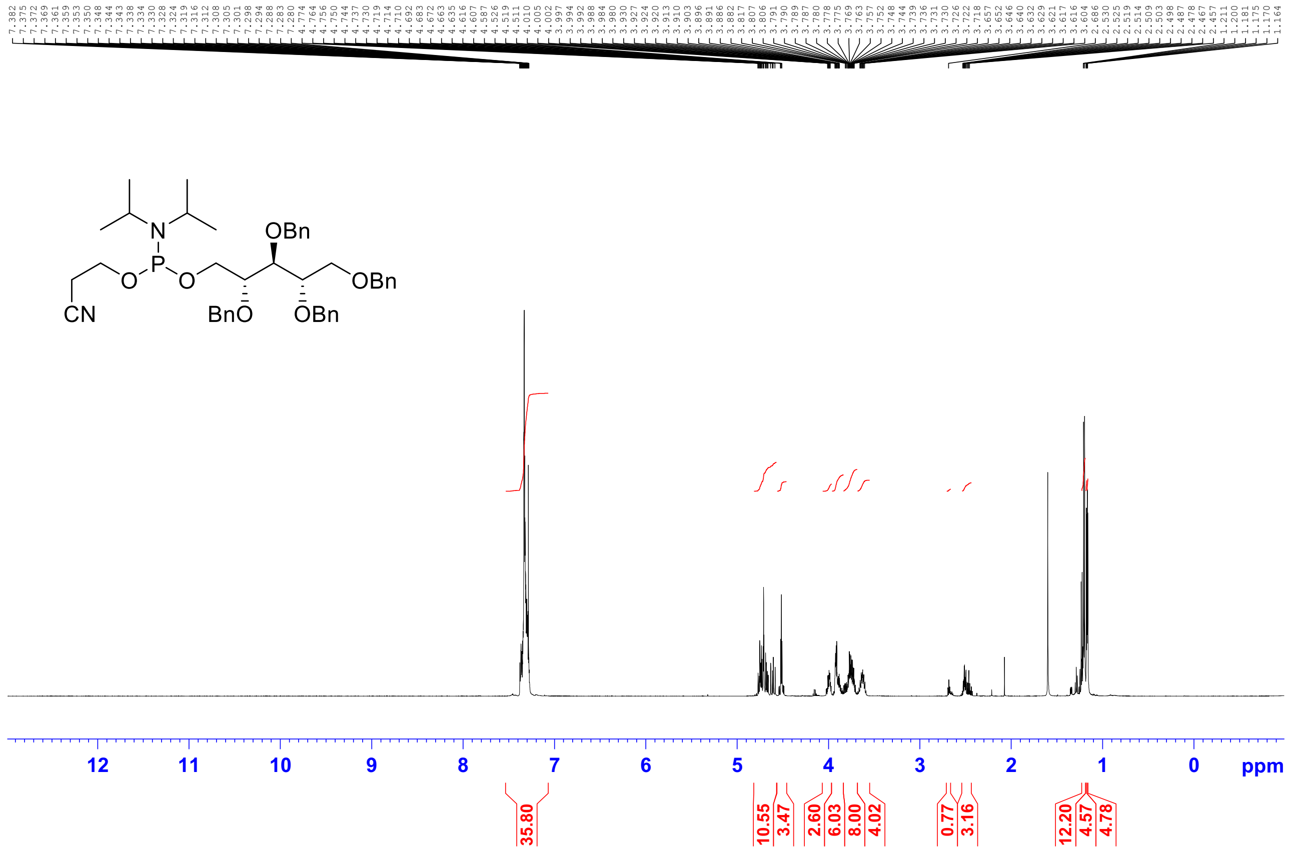

41: ^{13}C NMR (150 MHz, $CDCl_3$)

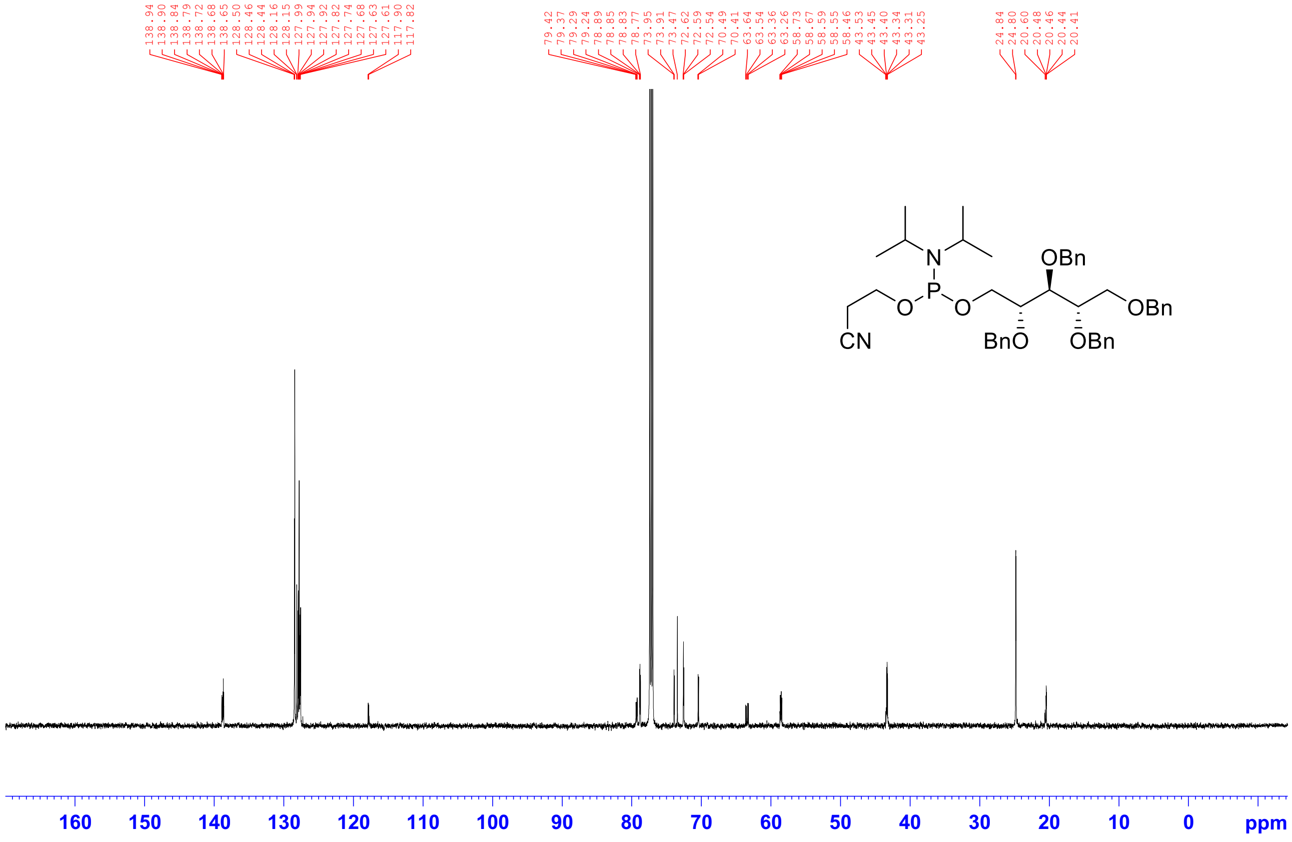

41: DEPT 135 (150 MHz, $CDCl_3$)

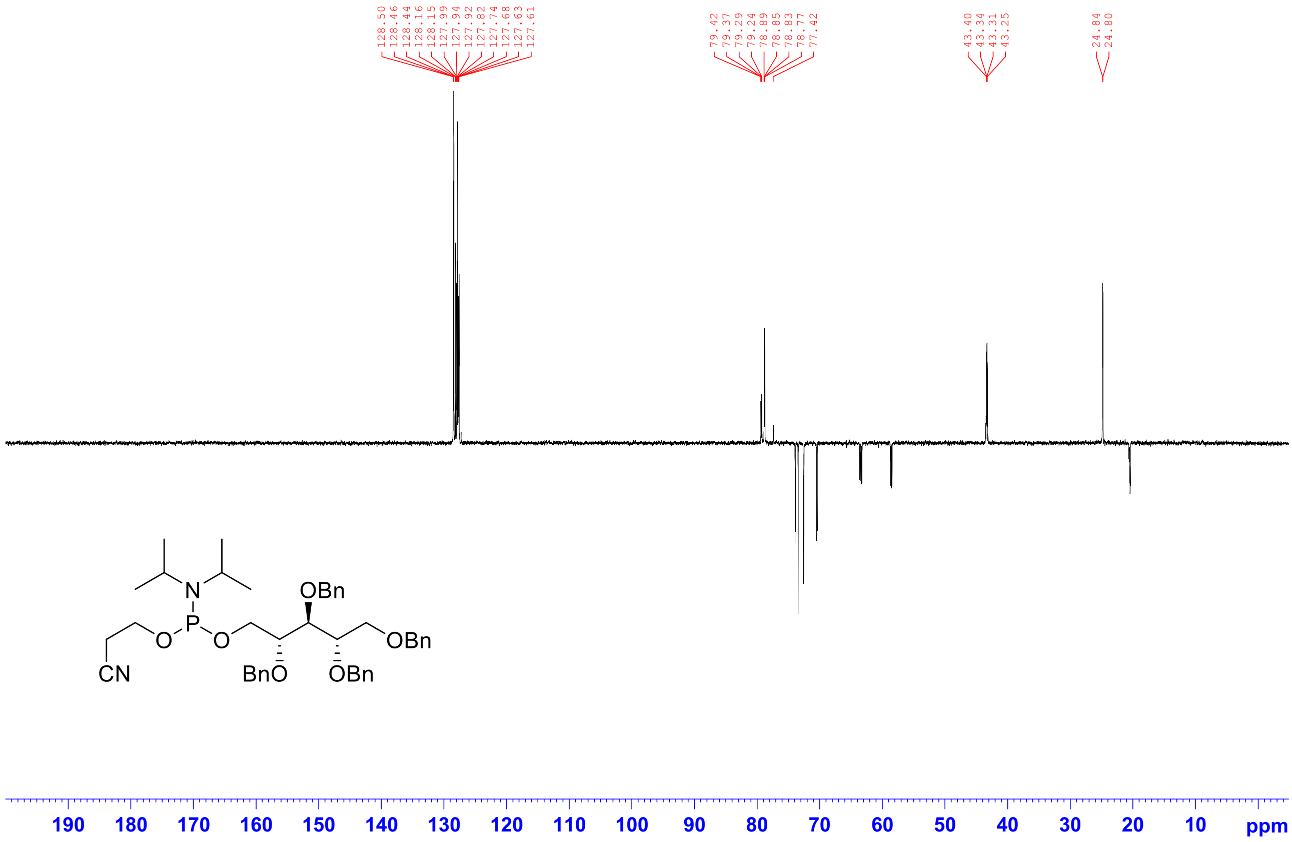

42: ^{1}H NMR (600 MHz, $CDCl_3$)

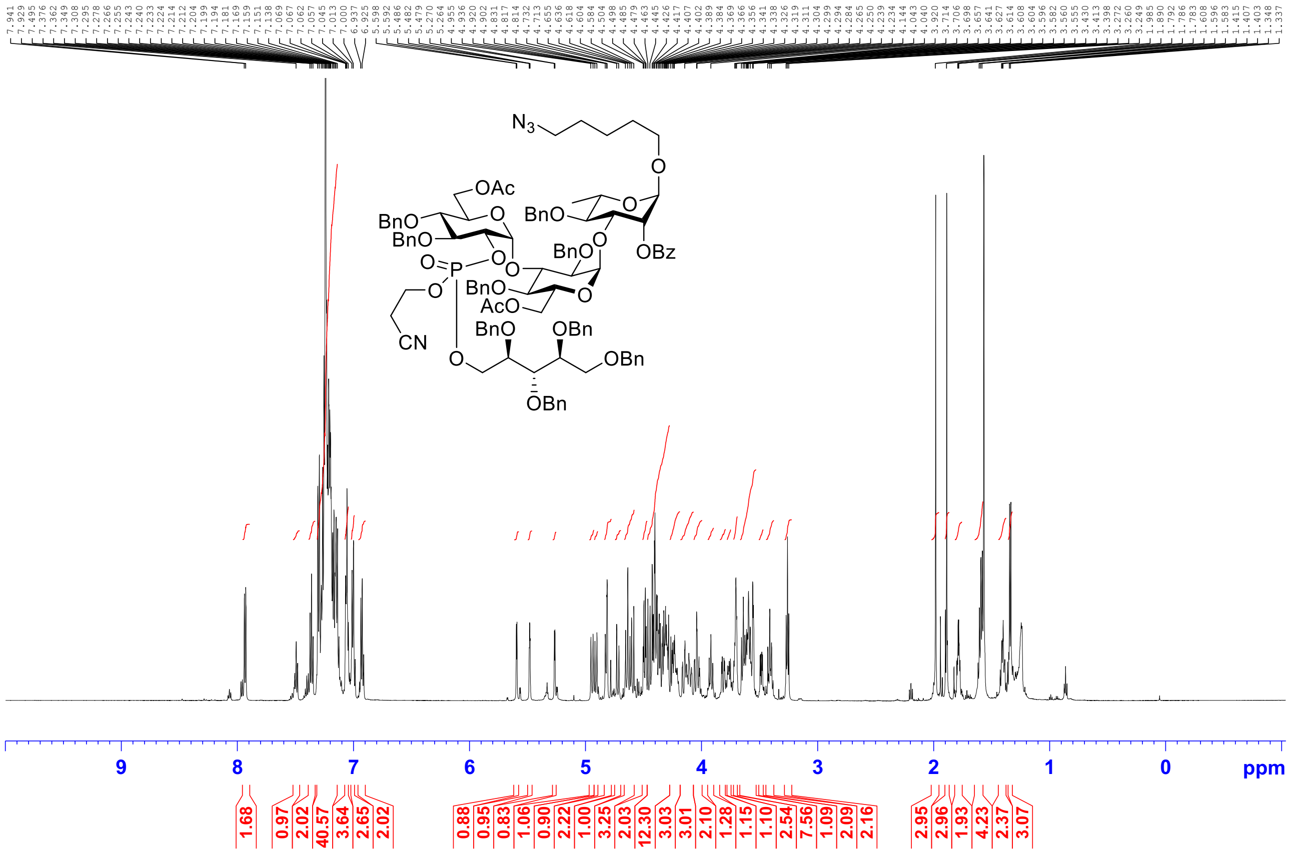

42: ^{13}C NMR (150 MHz, $CDCl_3$)

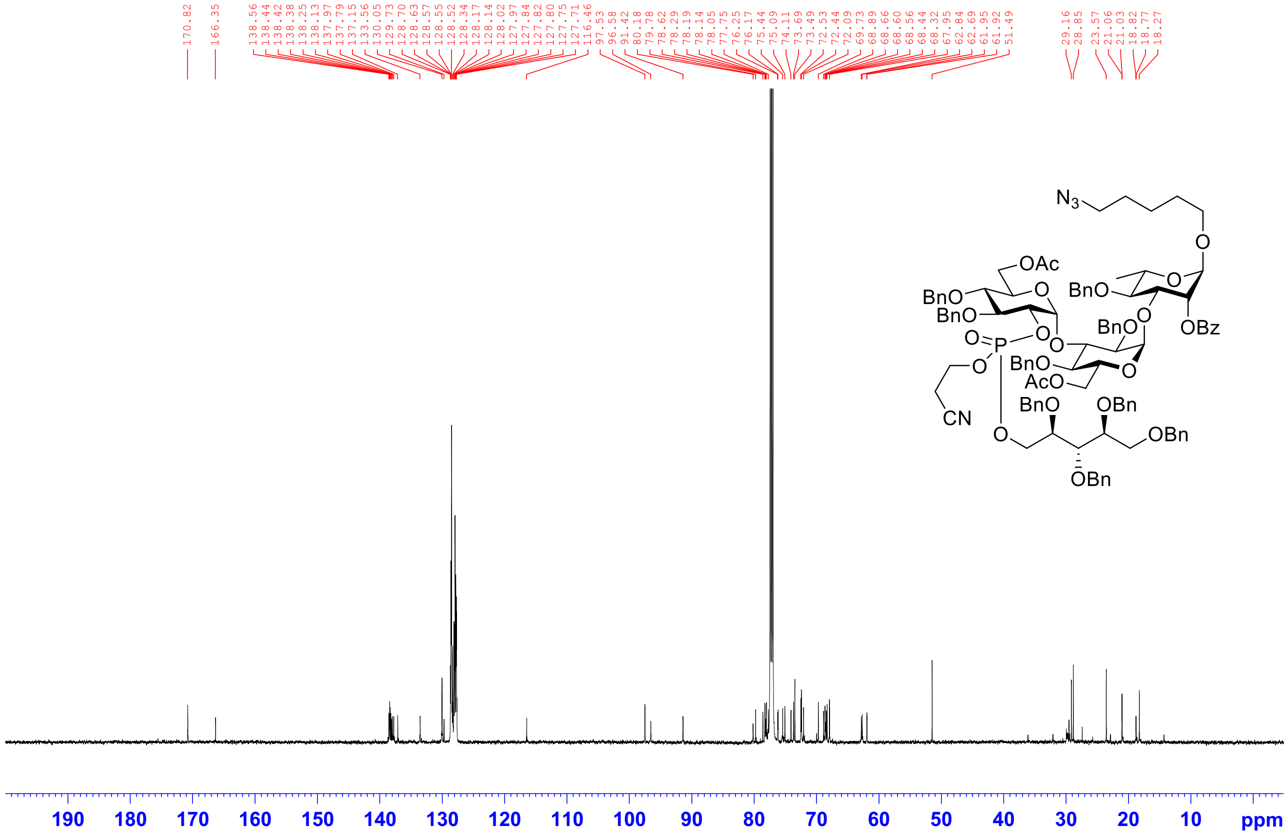

42: DEPT 135 (150 MHz, $CDCl_3$)

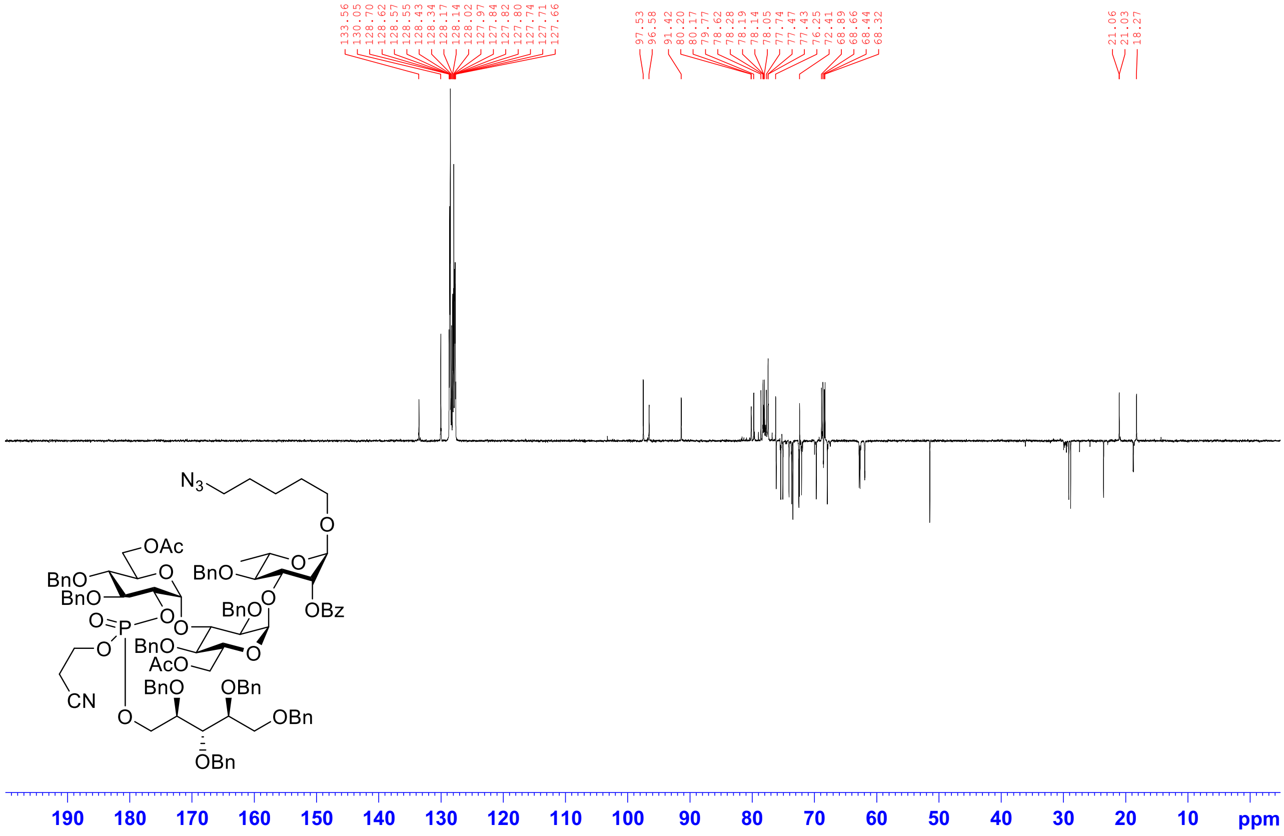

42: ^{31}P NMR (202 MHz, $CDCl_3$)

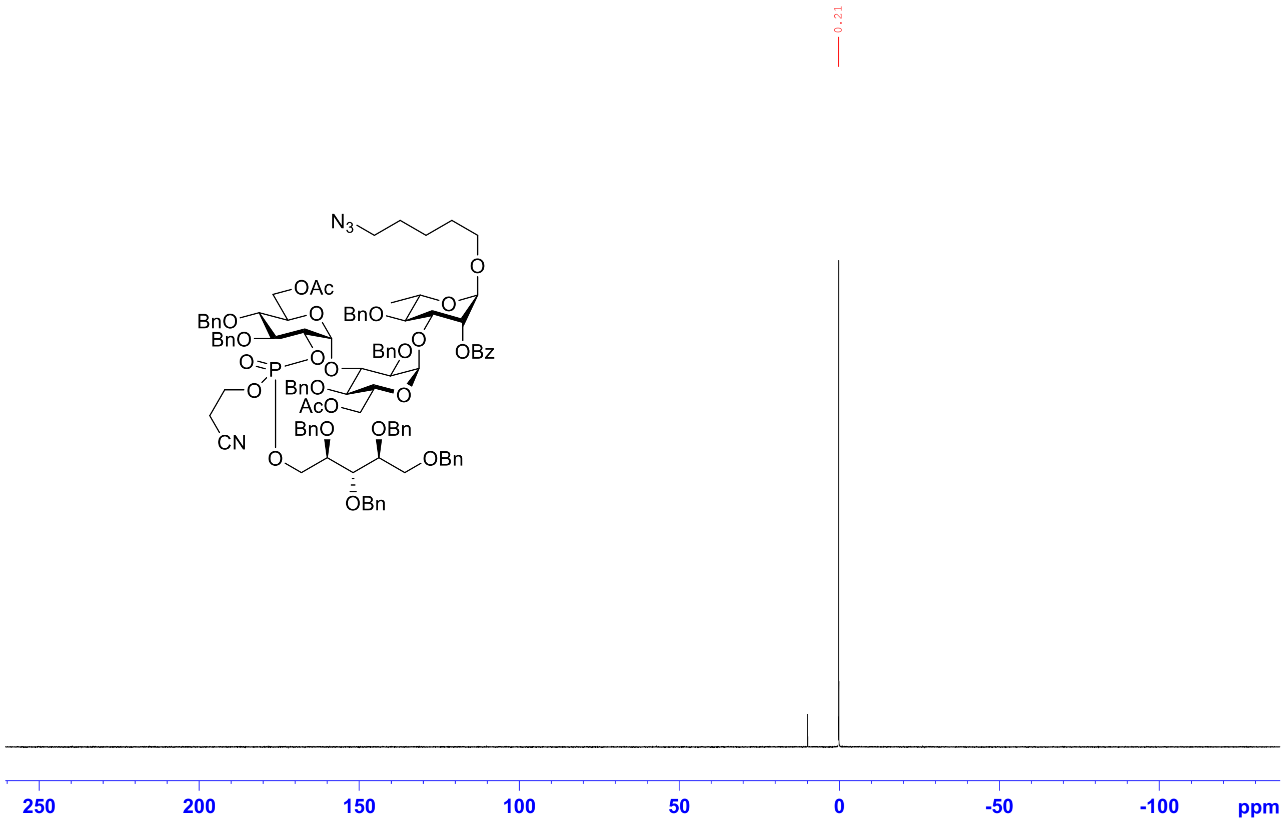

NMR spectra of ST6C and ST6D pseudo-tetrasaccharides

5: ^{1}H NMR (600 MHz, D_2O)

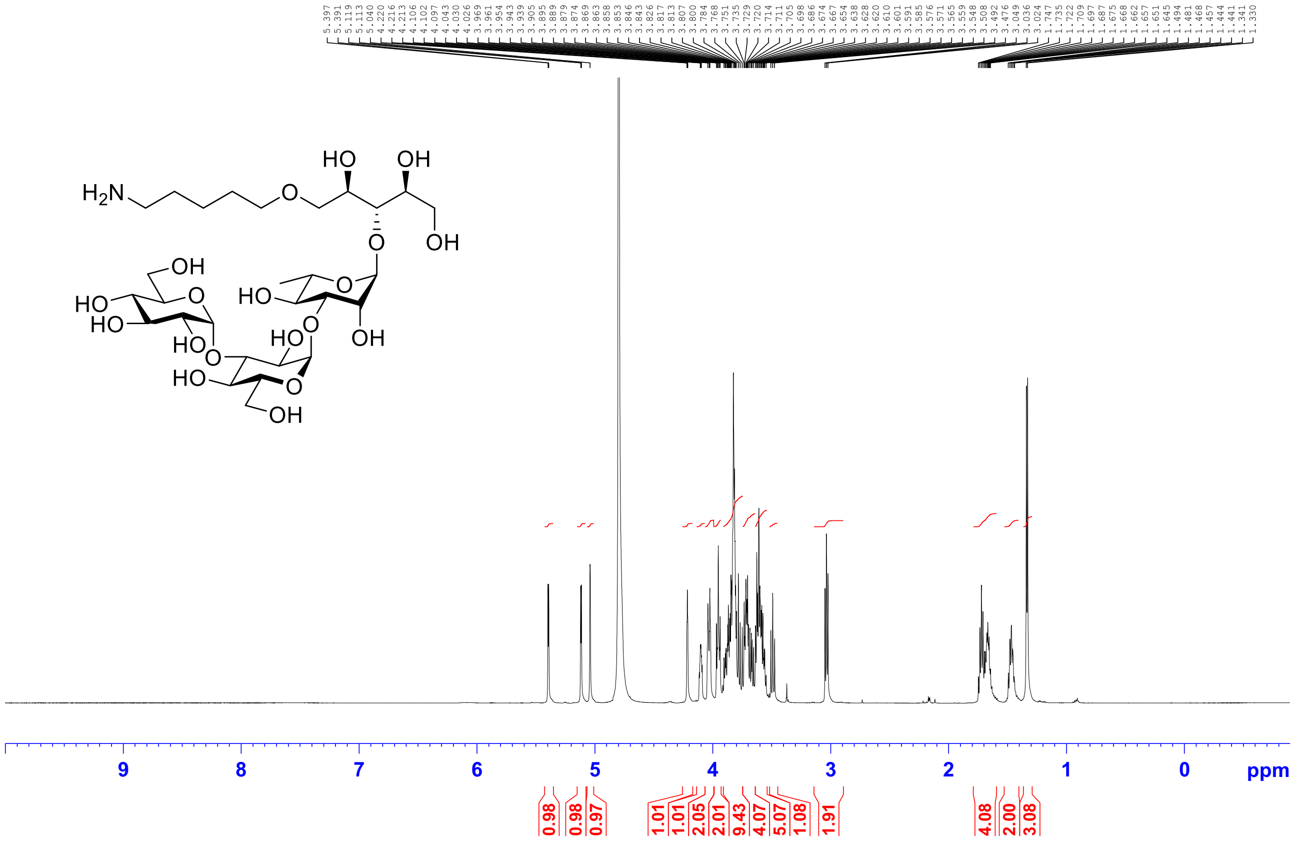

5: ^{13}C NMR (150 MHz, D_2O)

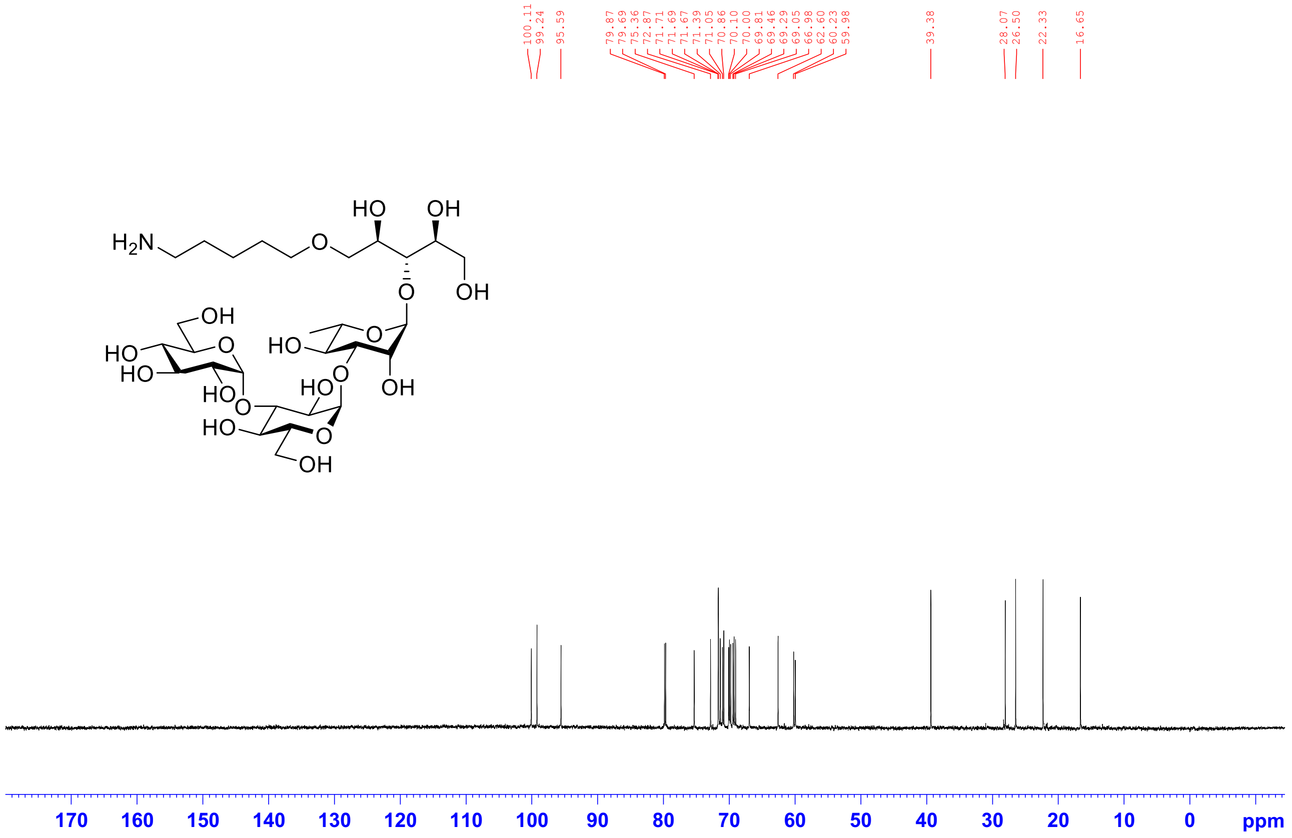

5: DEPT 135 (150 MHz, D_2O)

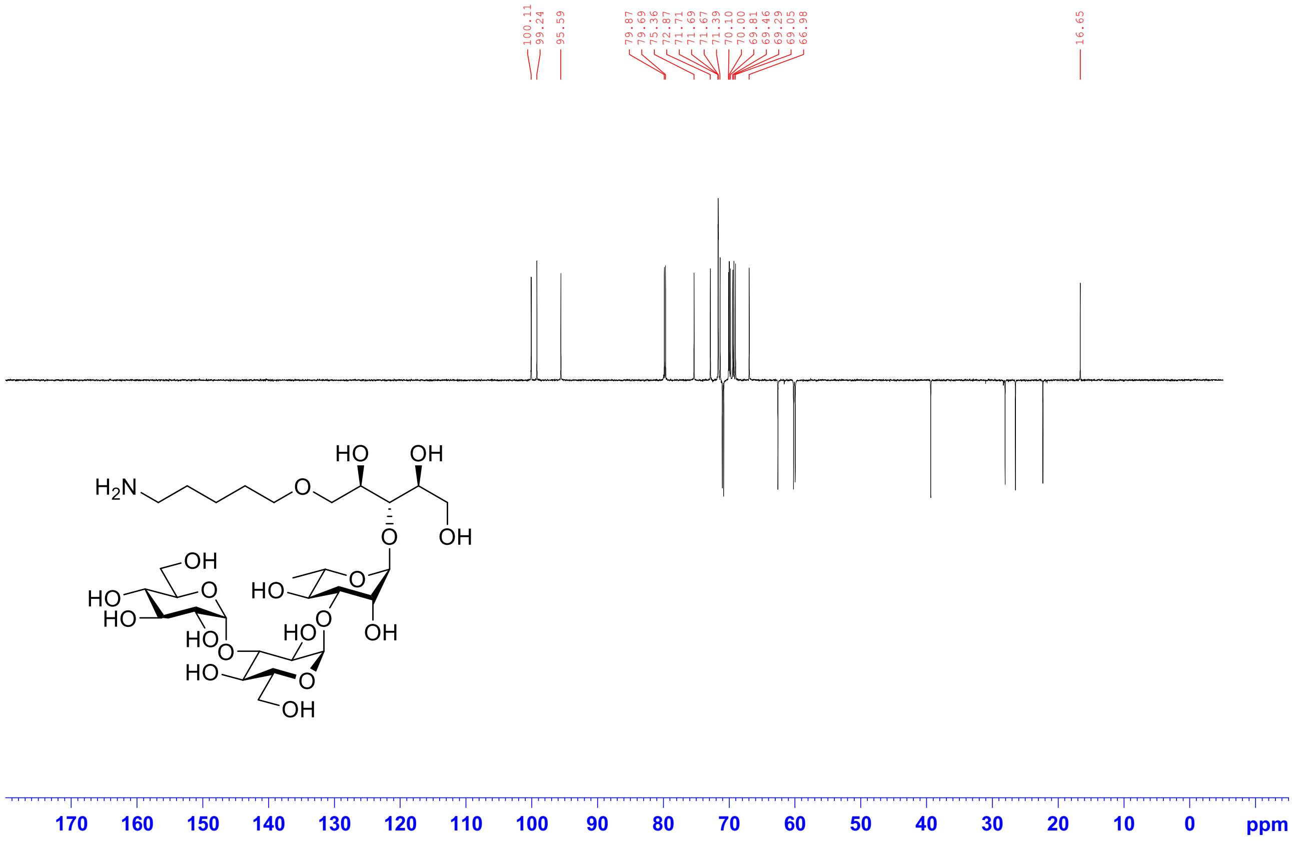

6: ^{1}H NMR (600 MHz, D_2O)

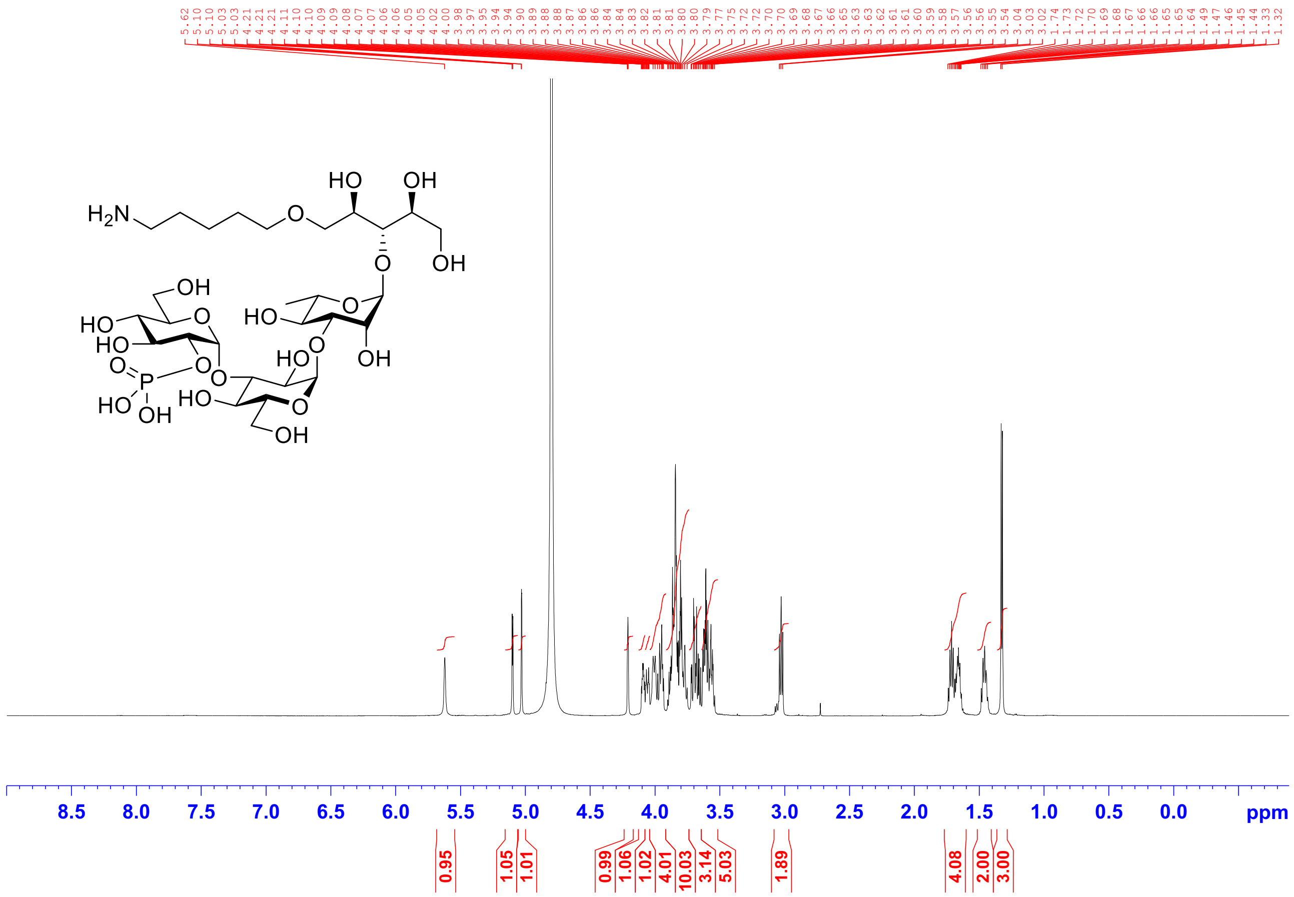

6: ^{13}C NMR (150 MHz, D_2O)

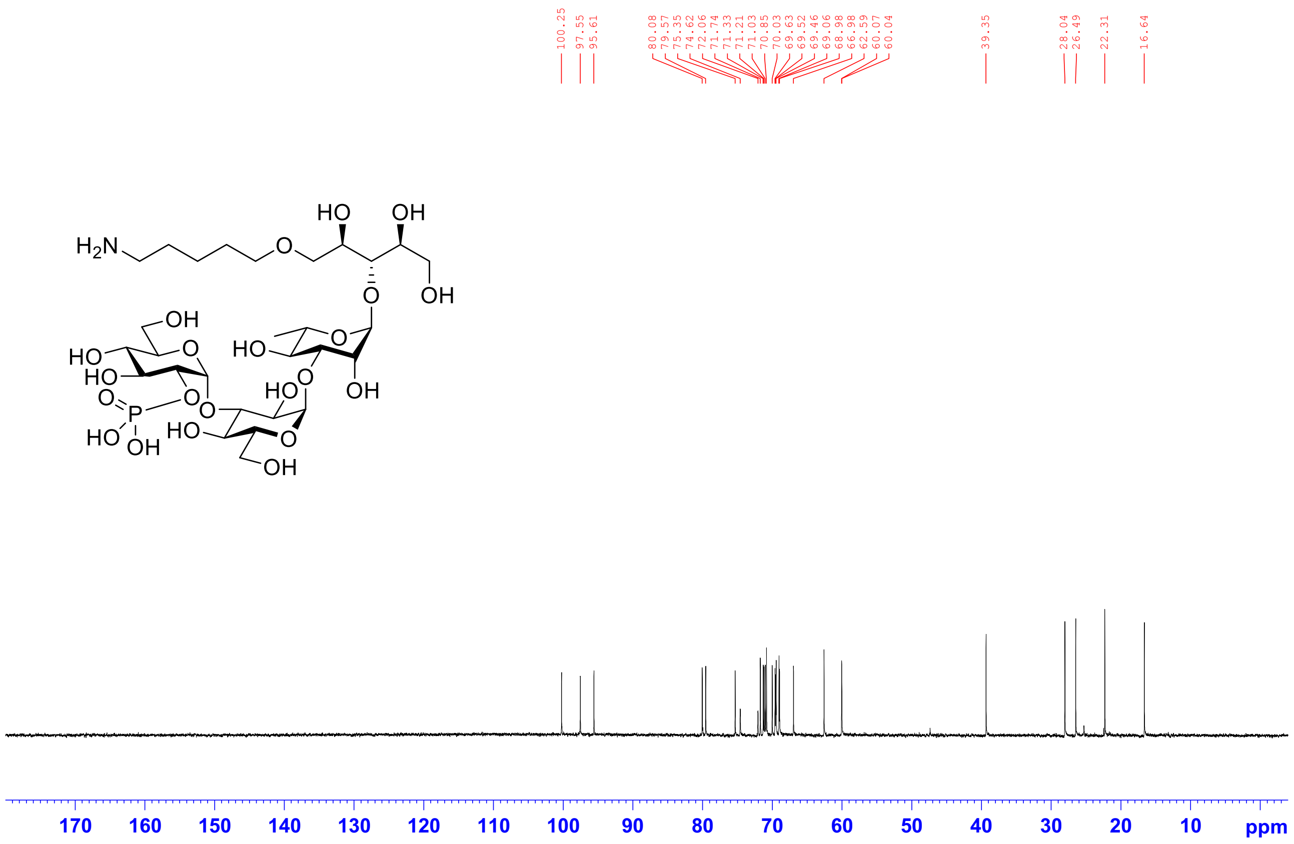

6: DEPT 135 (150 MHz, D_2O)

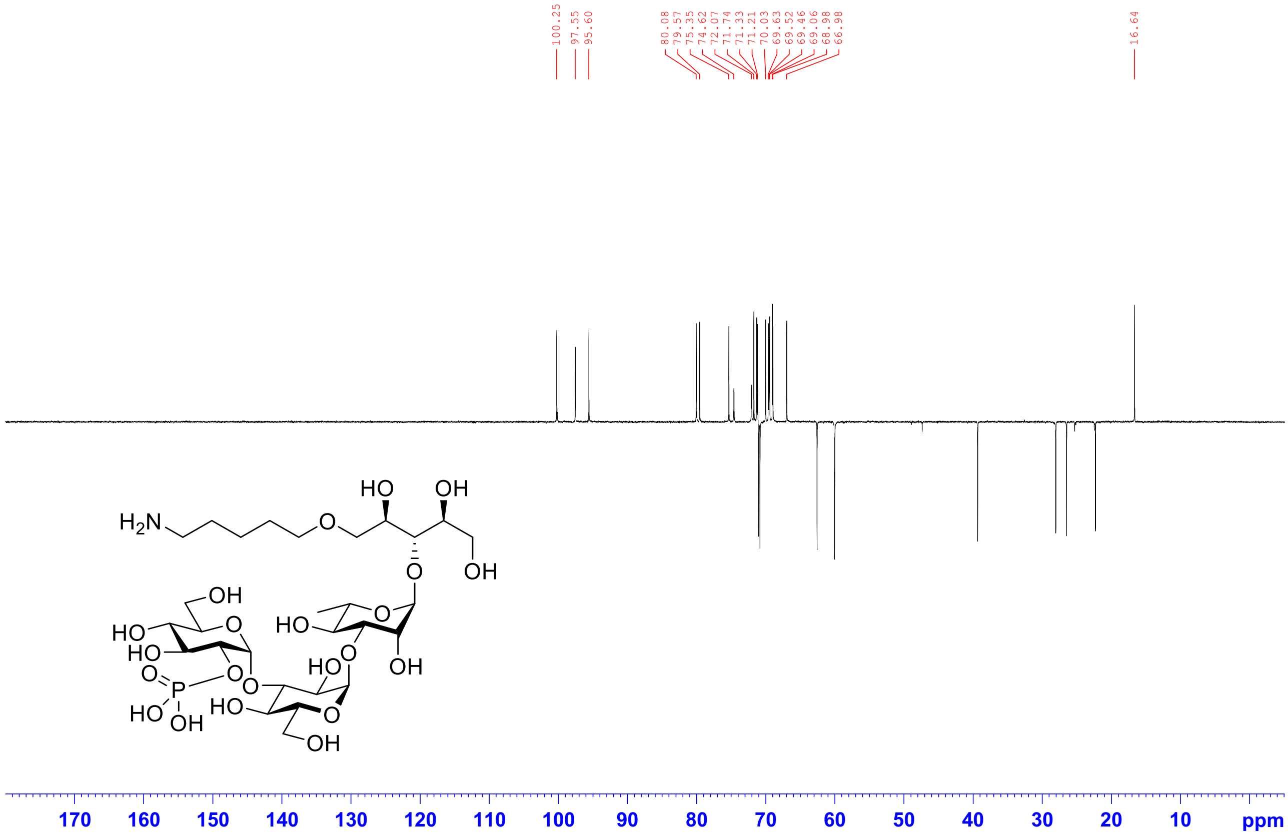

6: ^{31}P NMR (202 MHz, D_2O)

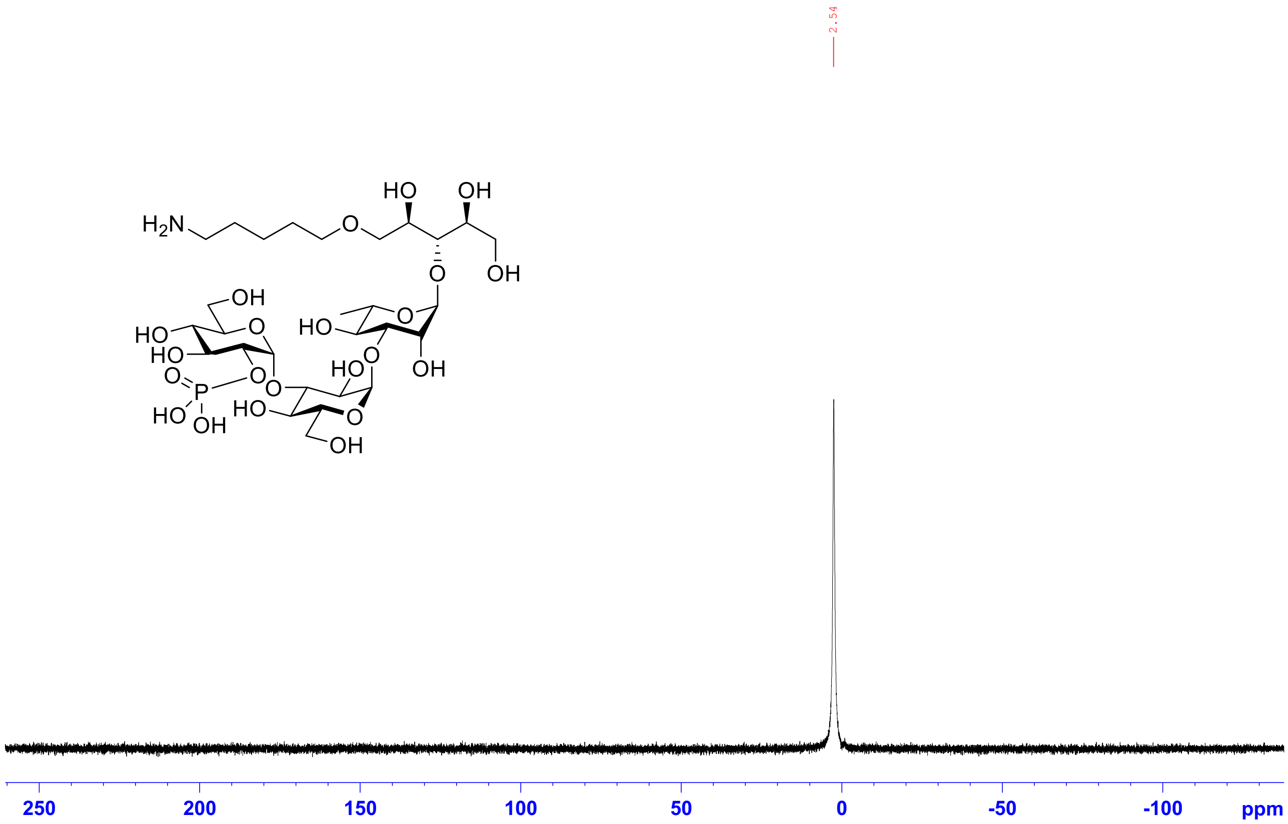

9: ^{1}H NMR (600 MHz, D_2O)

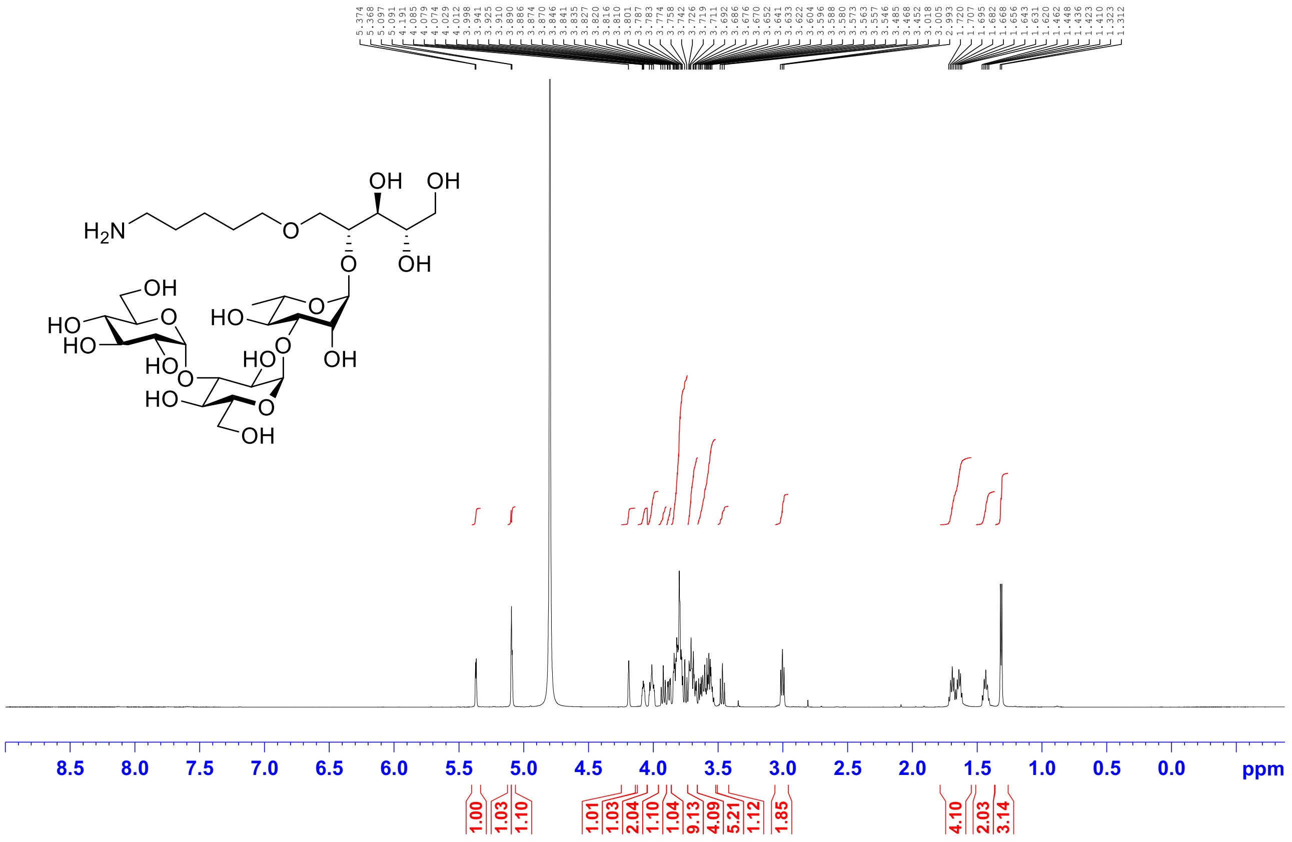

9: ^{13}C NMR (150 MHz, D_2O)

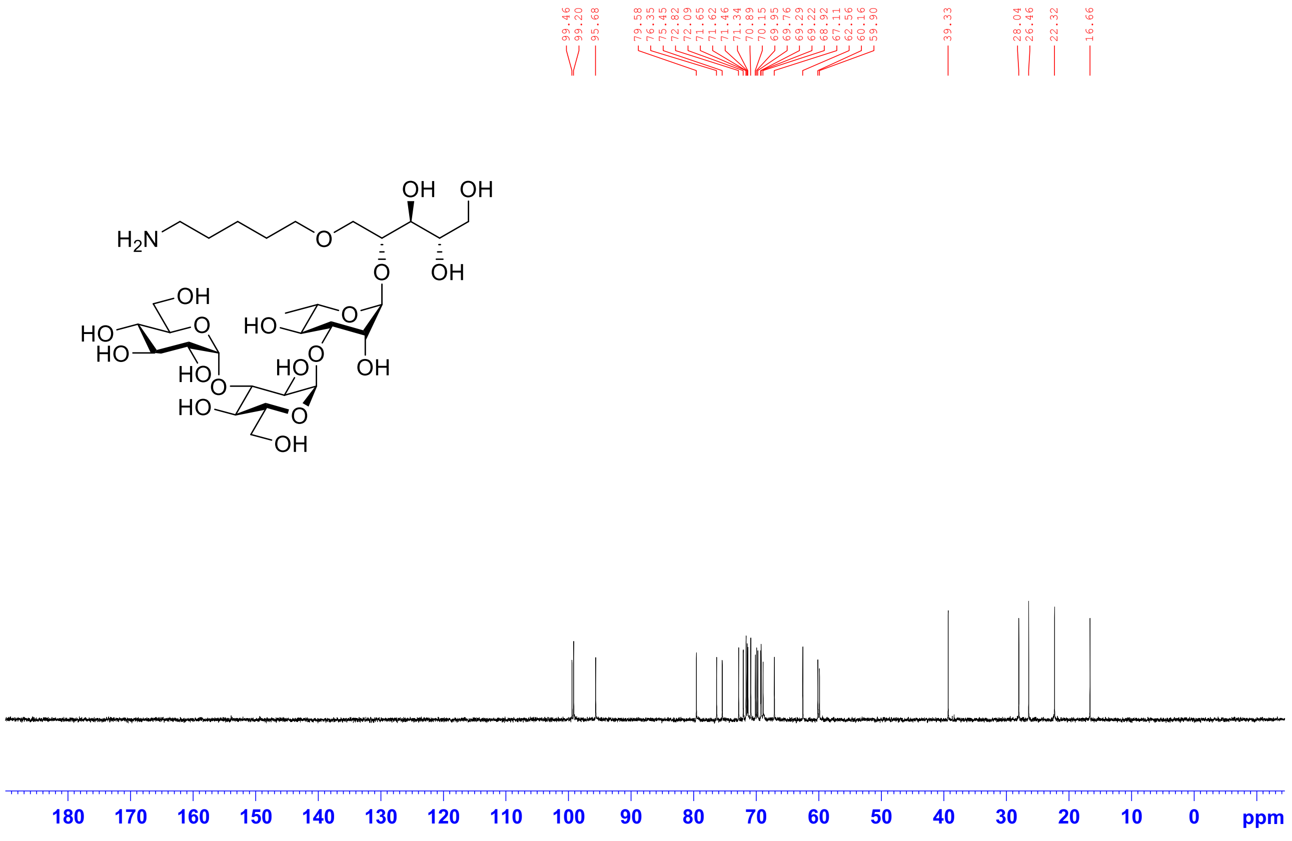

9: DEPT 135 (150 MHz, D_2O)

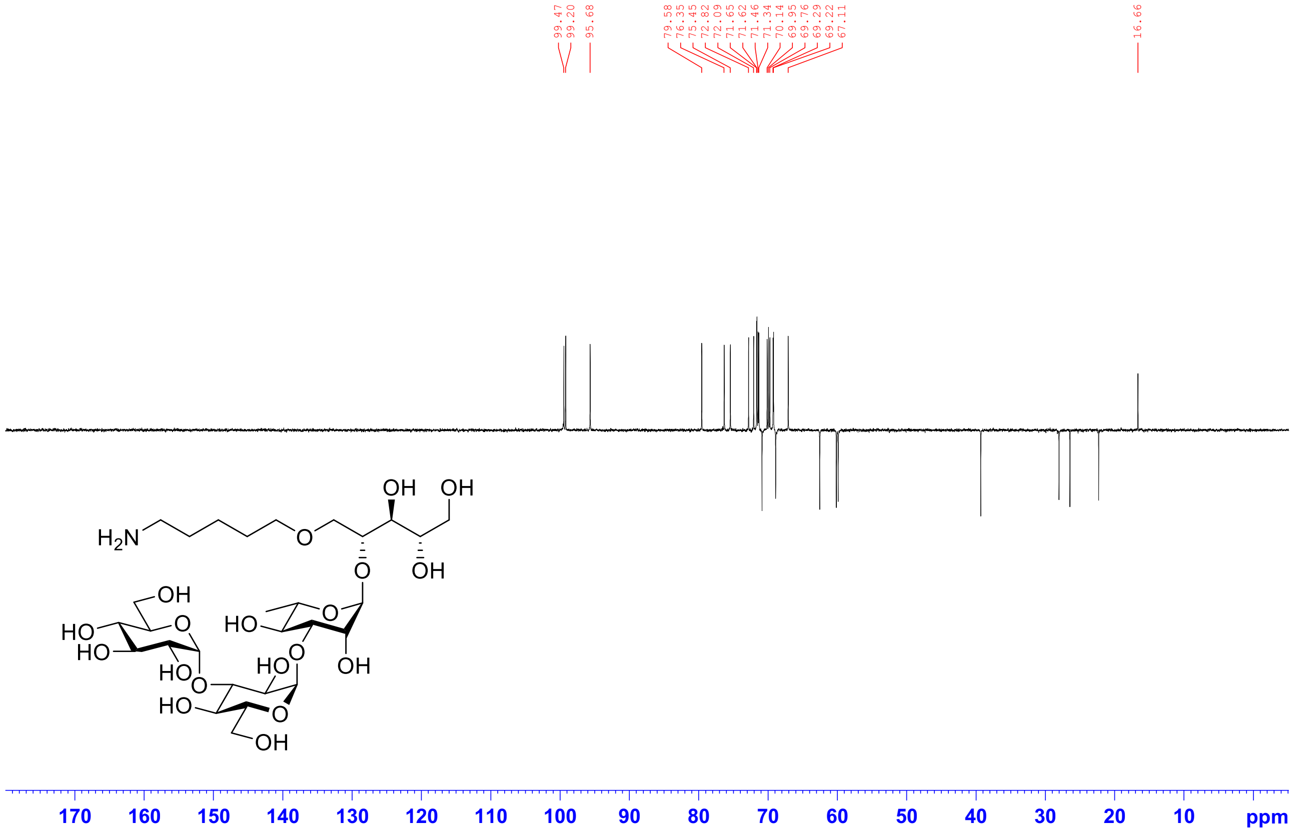

10: ^{1}H NMR (600 MHz, D_2O)

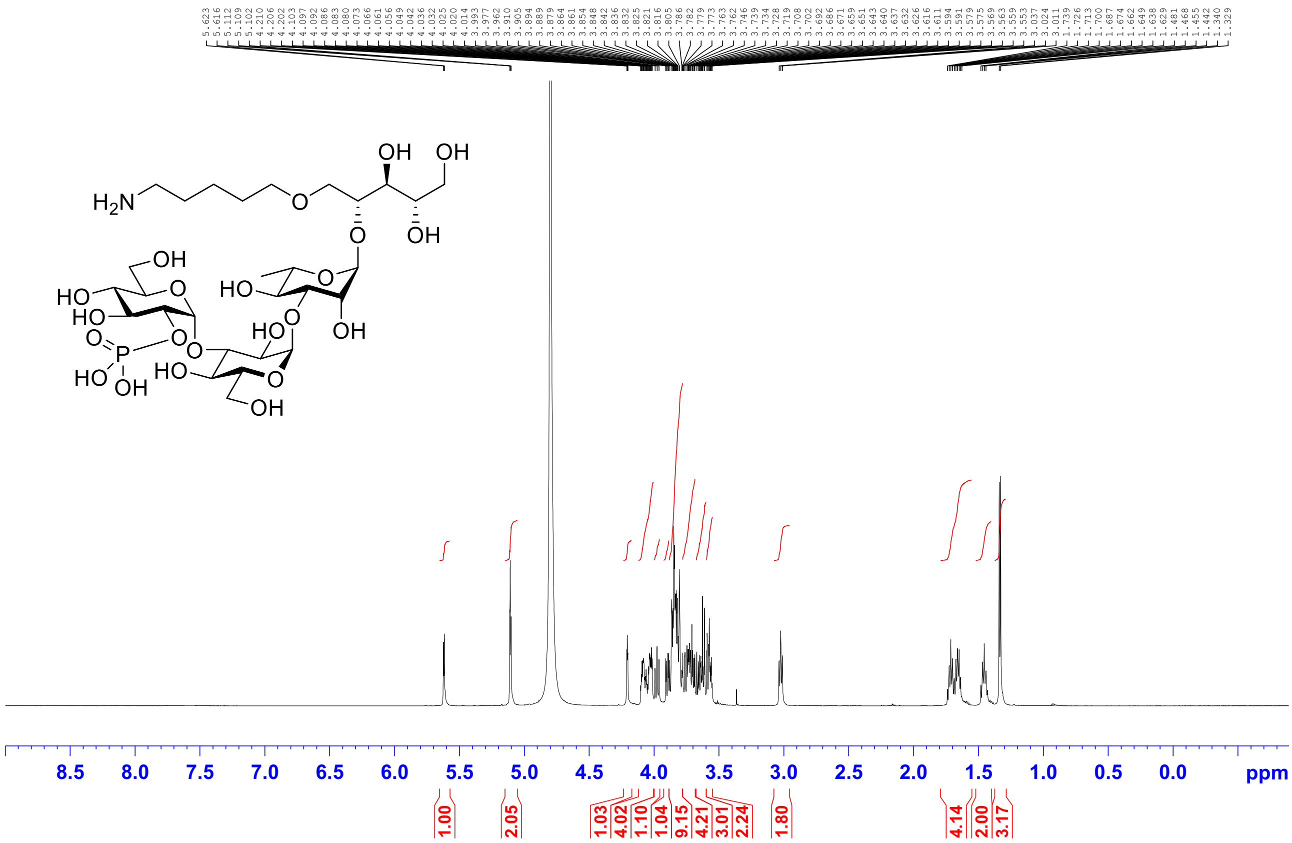

10: ^{13}C NMR (150 MHz, D_2O)

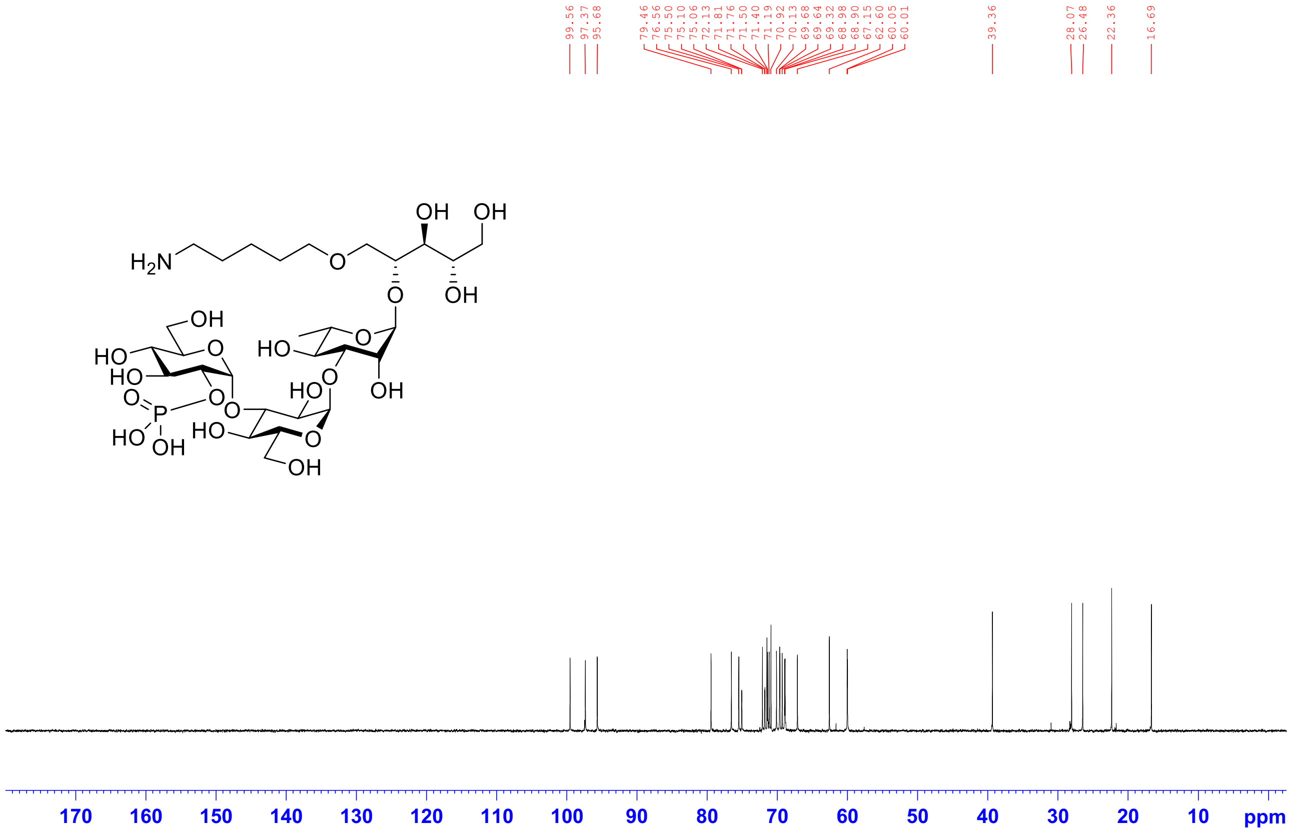

10: DEPT 135 (150 MHz, D_2O)

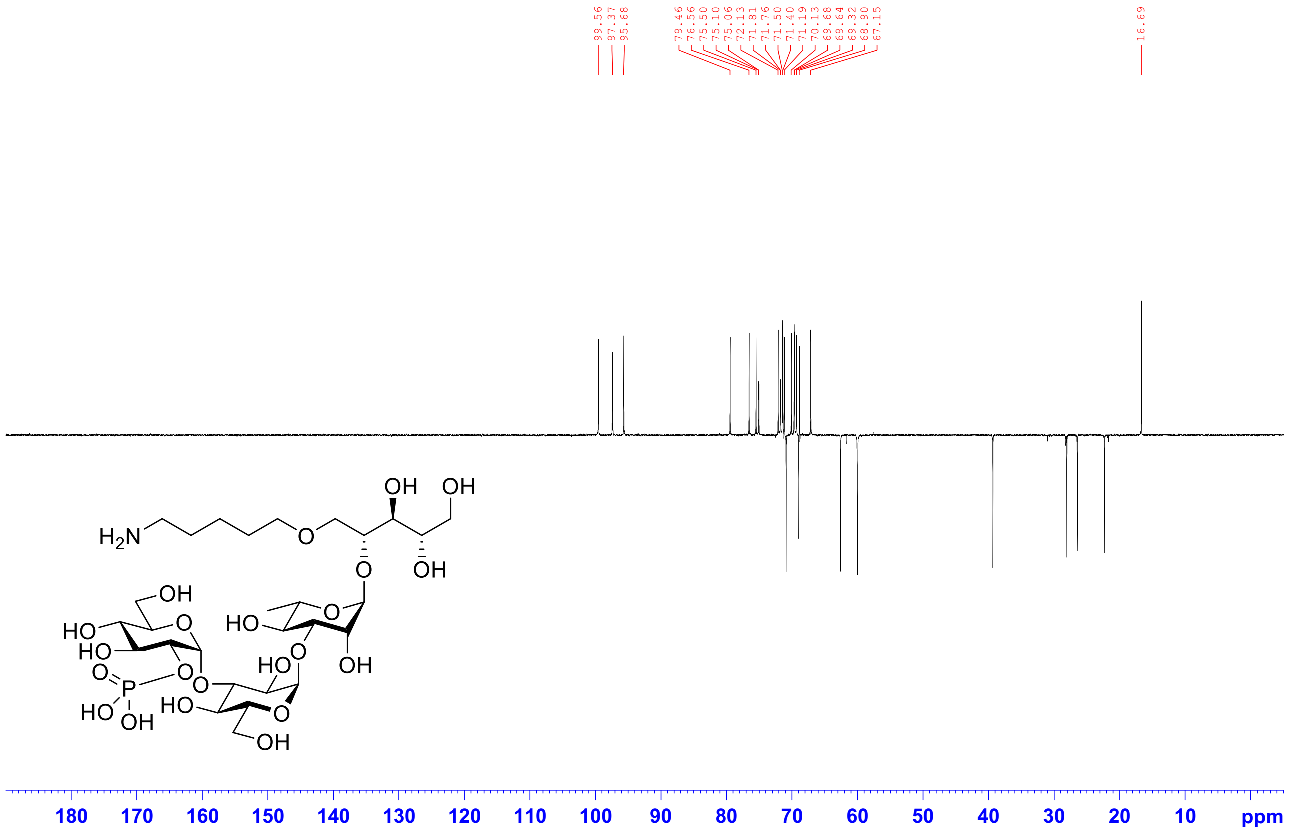

10: ^{31}P NMR (202 MHz, D_2O)

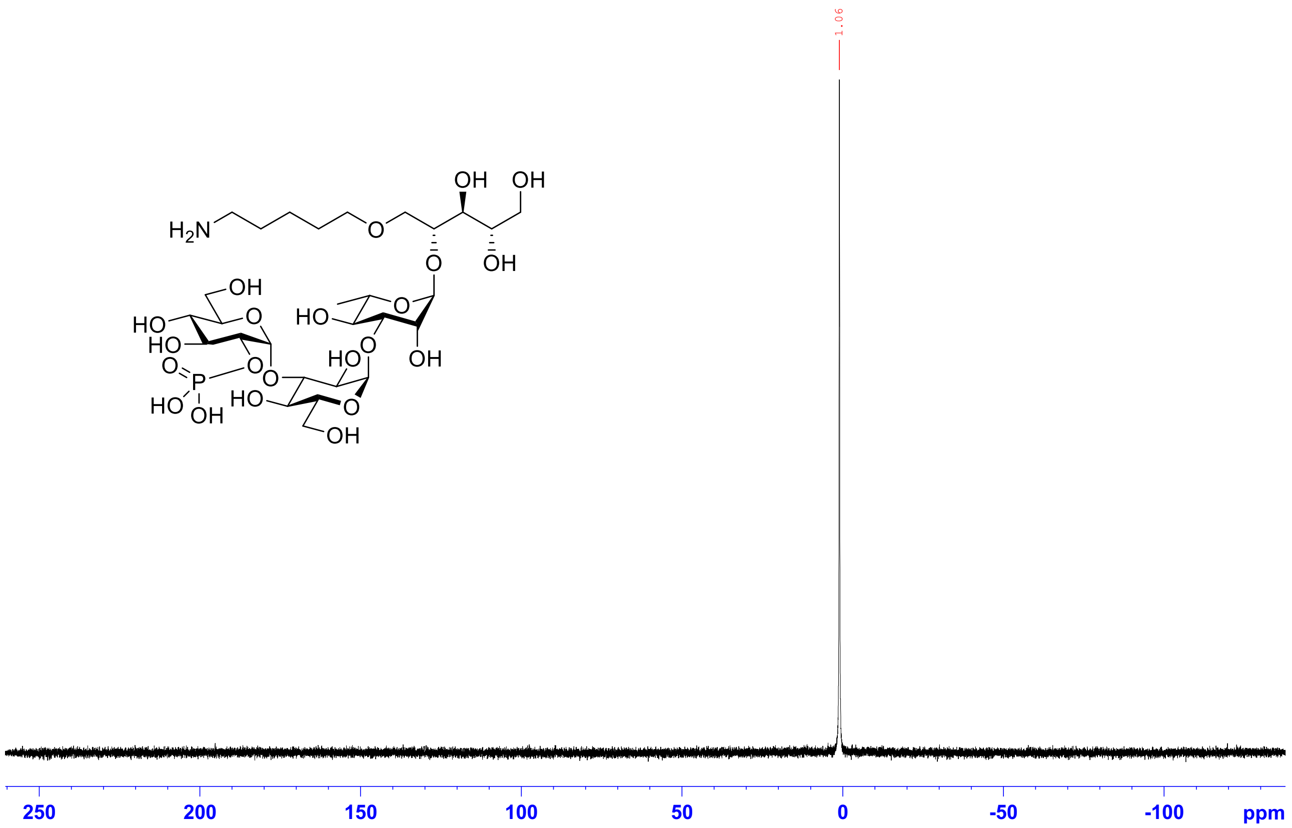

8: ^{1}H NMR (600 MHz, D_2O)

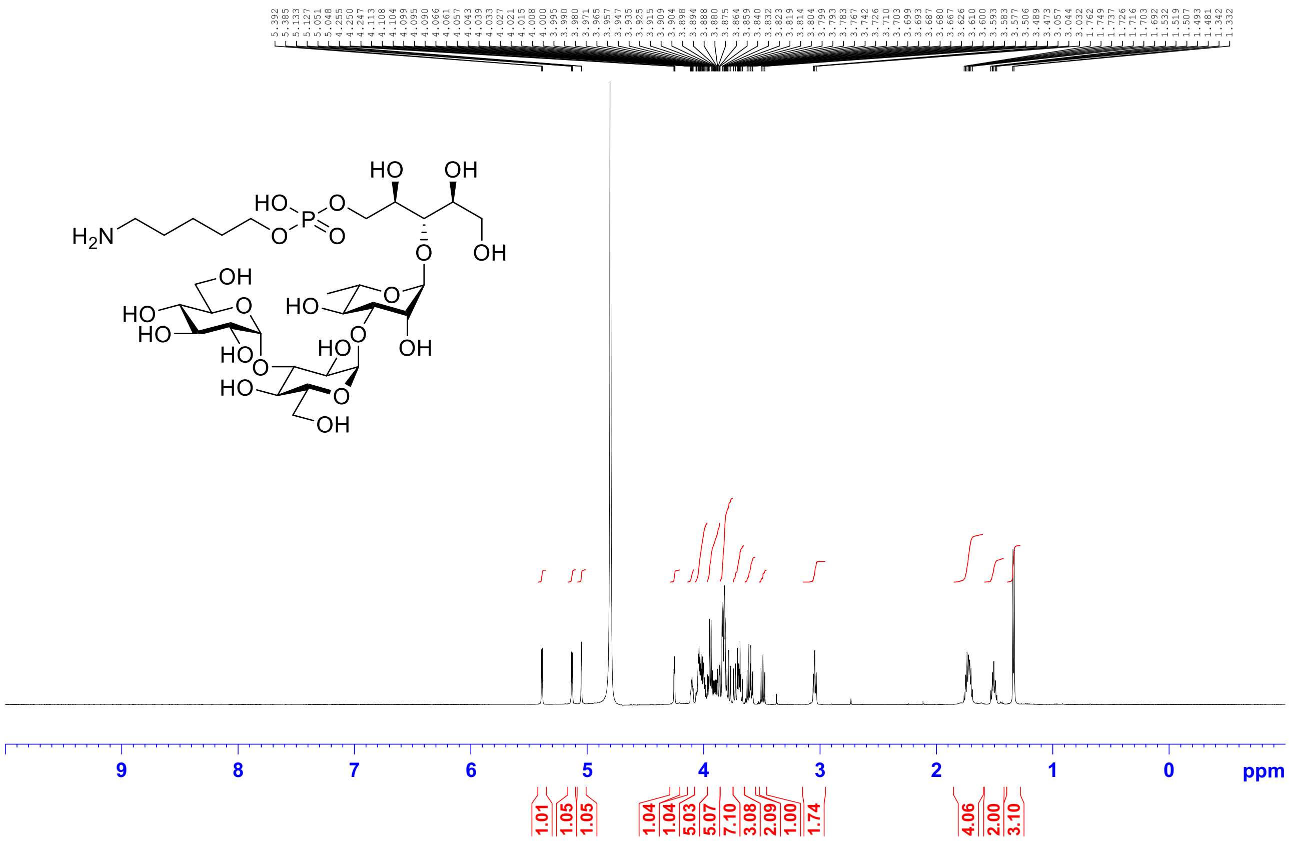

8: ^{13}C NMR (150 MHz, D_2O)

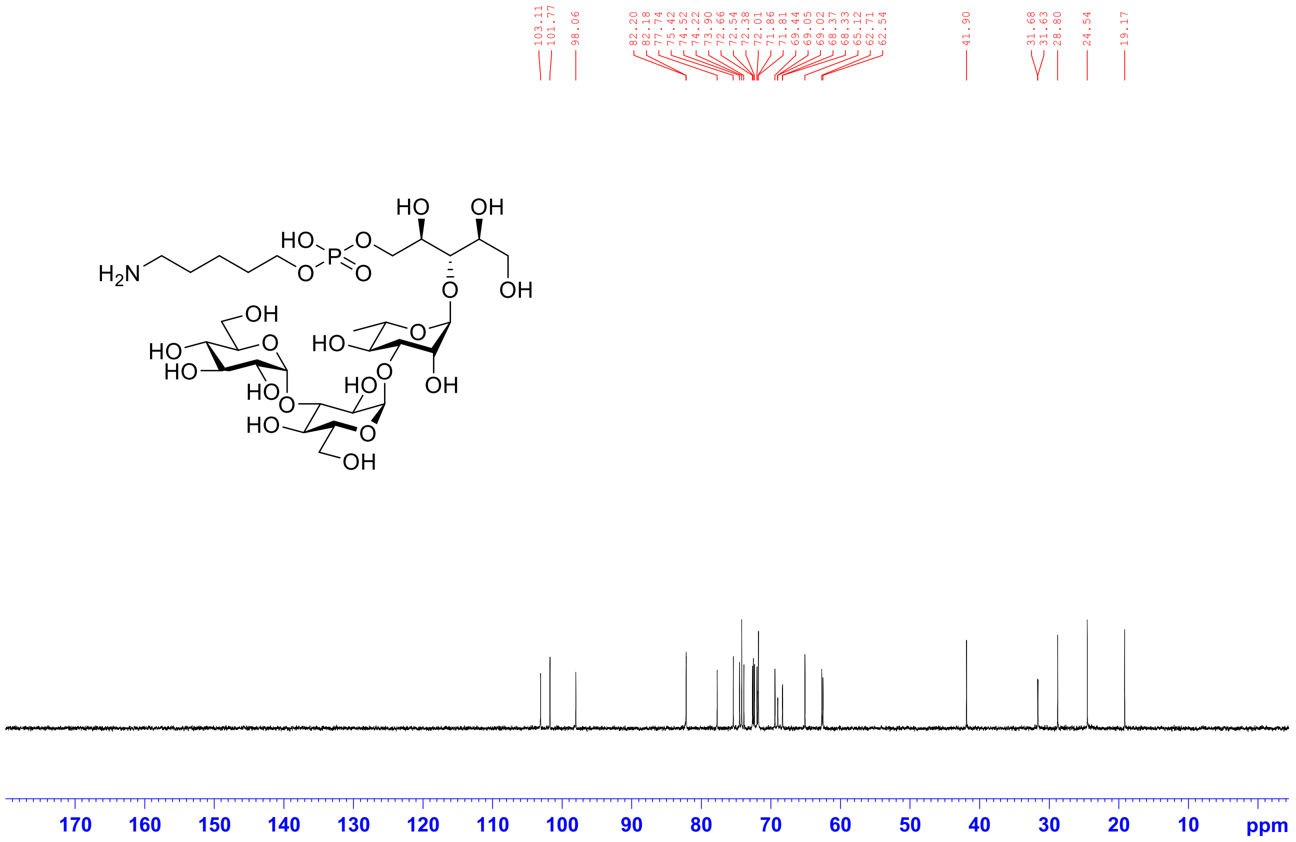

8: DEPT 135 (150 MHz, D_2O)

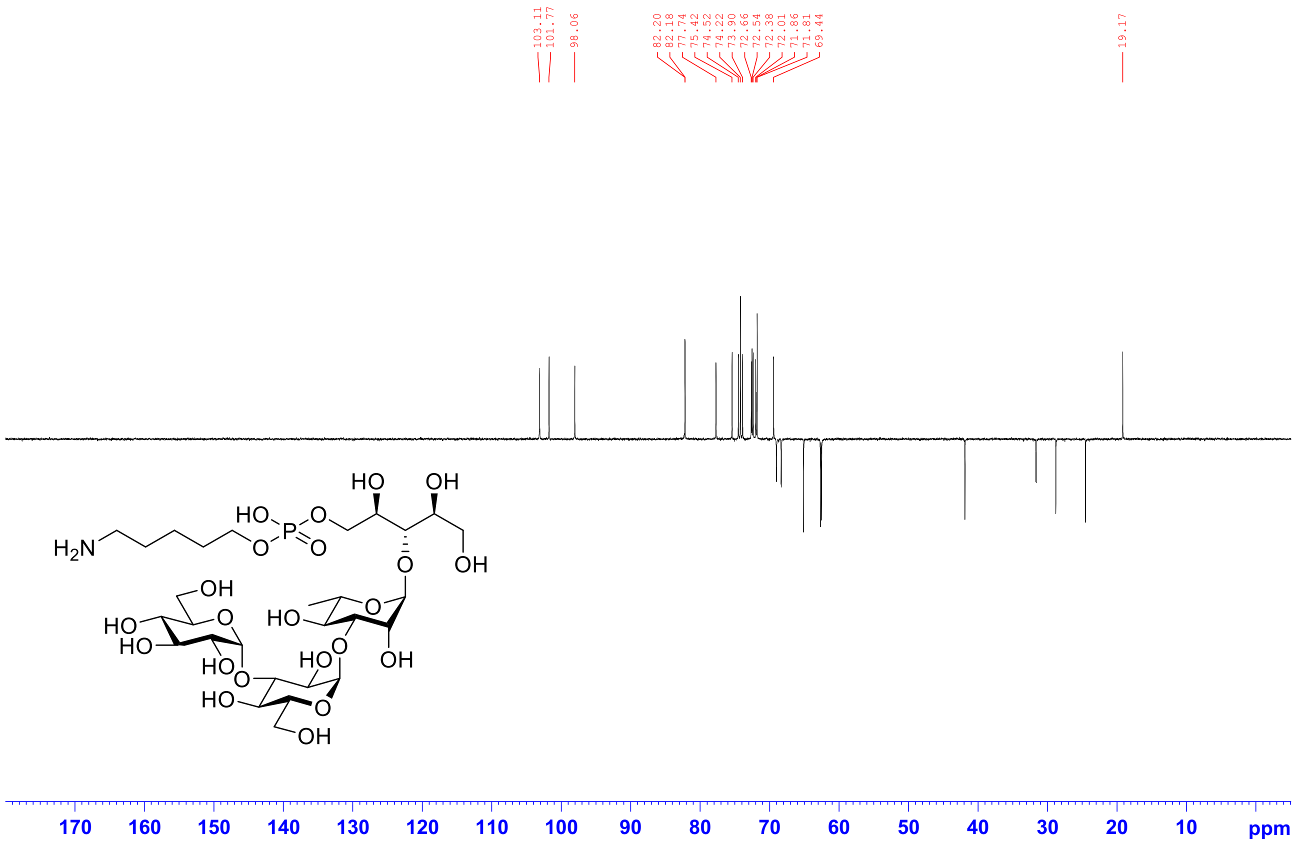

8: ^{31}P NMR (202 MHz, D_2O)

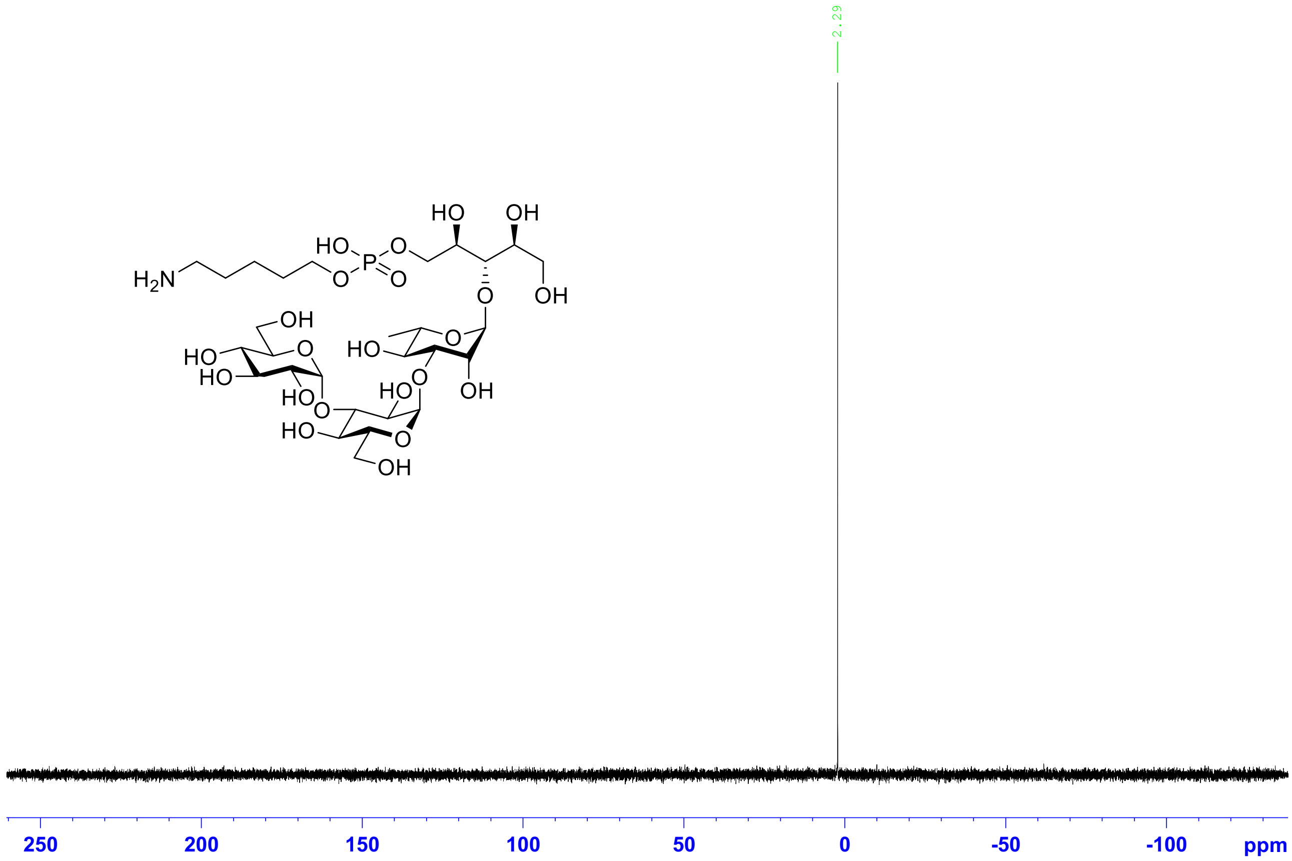

12: ^{1}H NMR (600 MHz, D_2O)

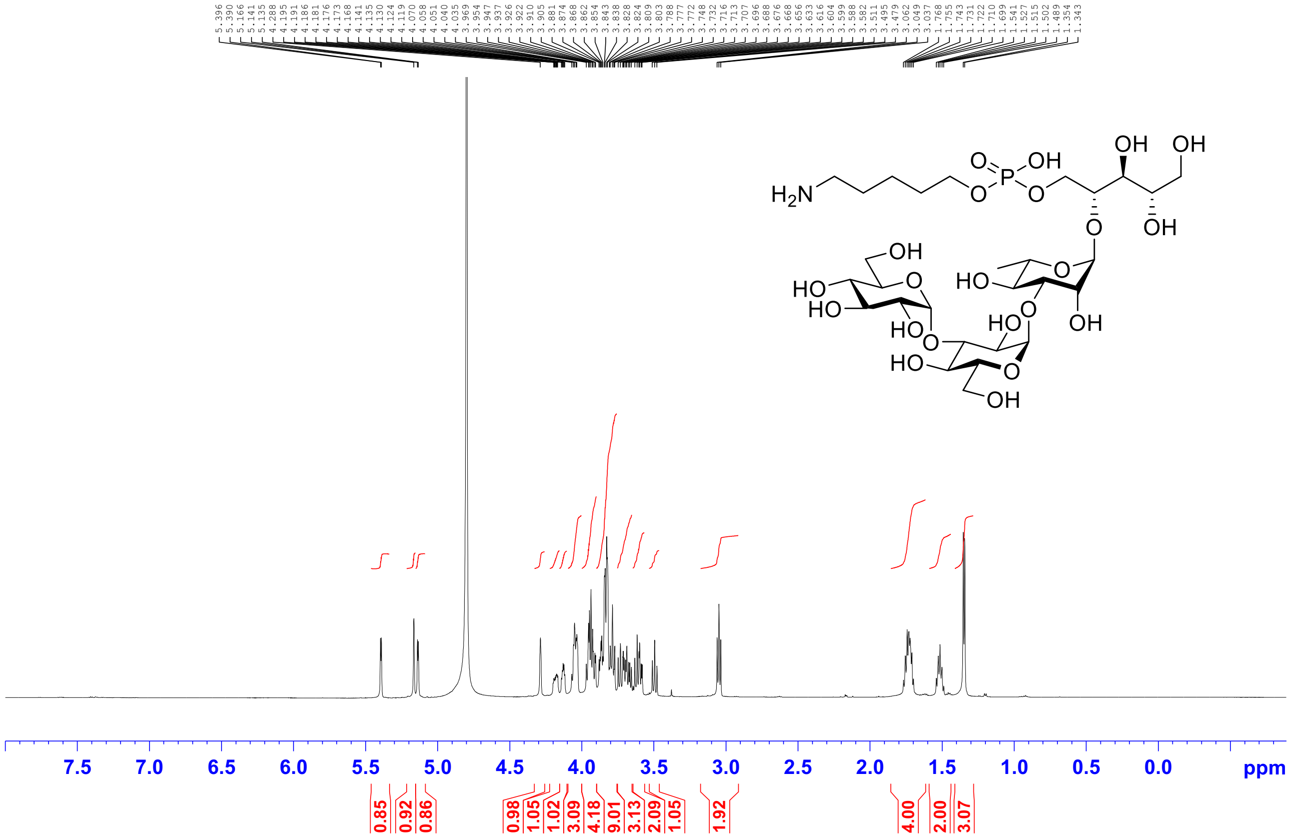

12: ^{13}C NMR (150 MHz, D_2O)

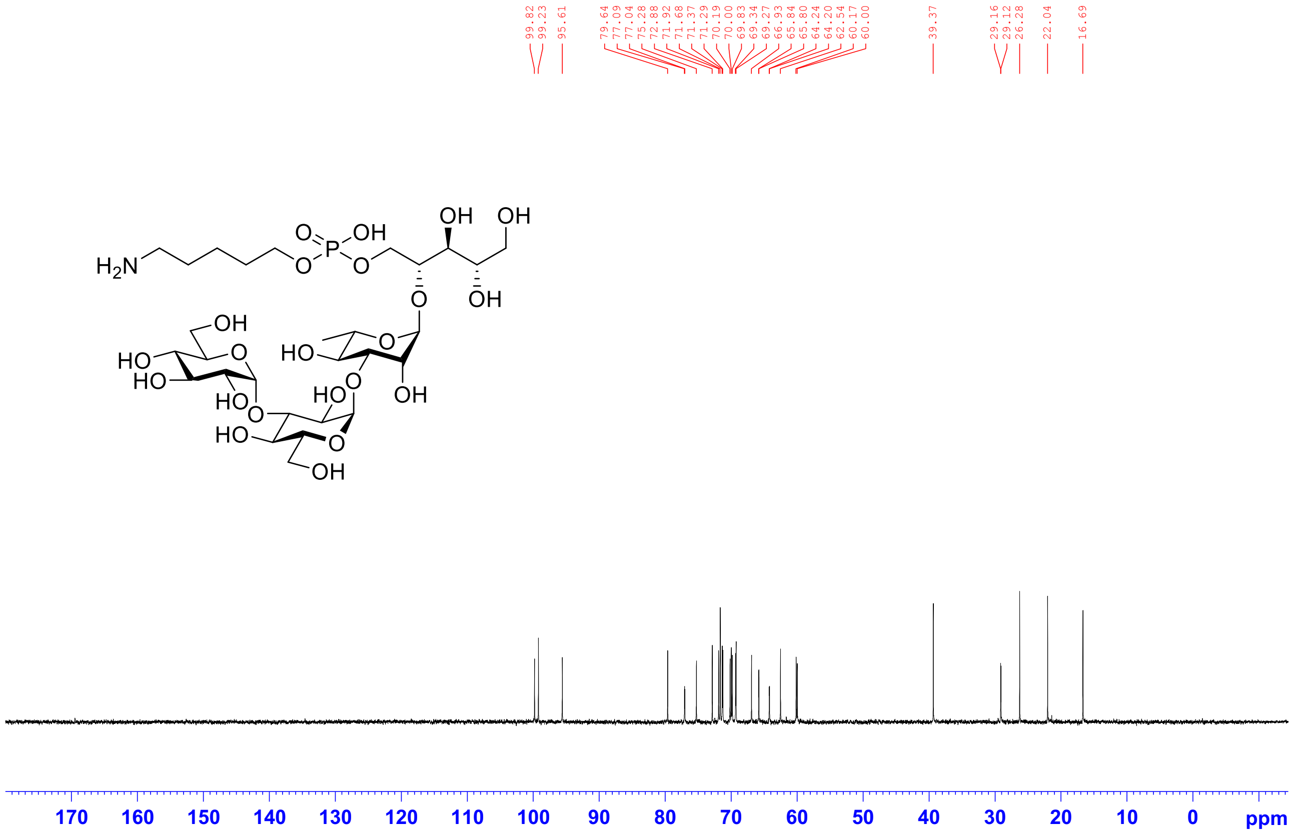

12: DEPT 135 (150 MHz, D_2O)

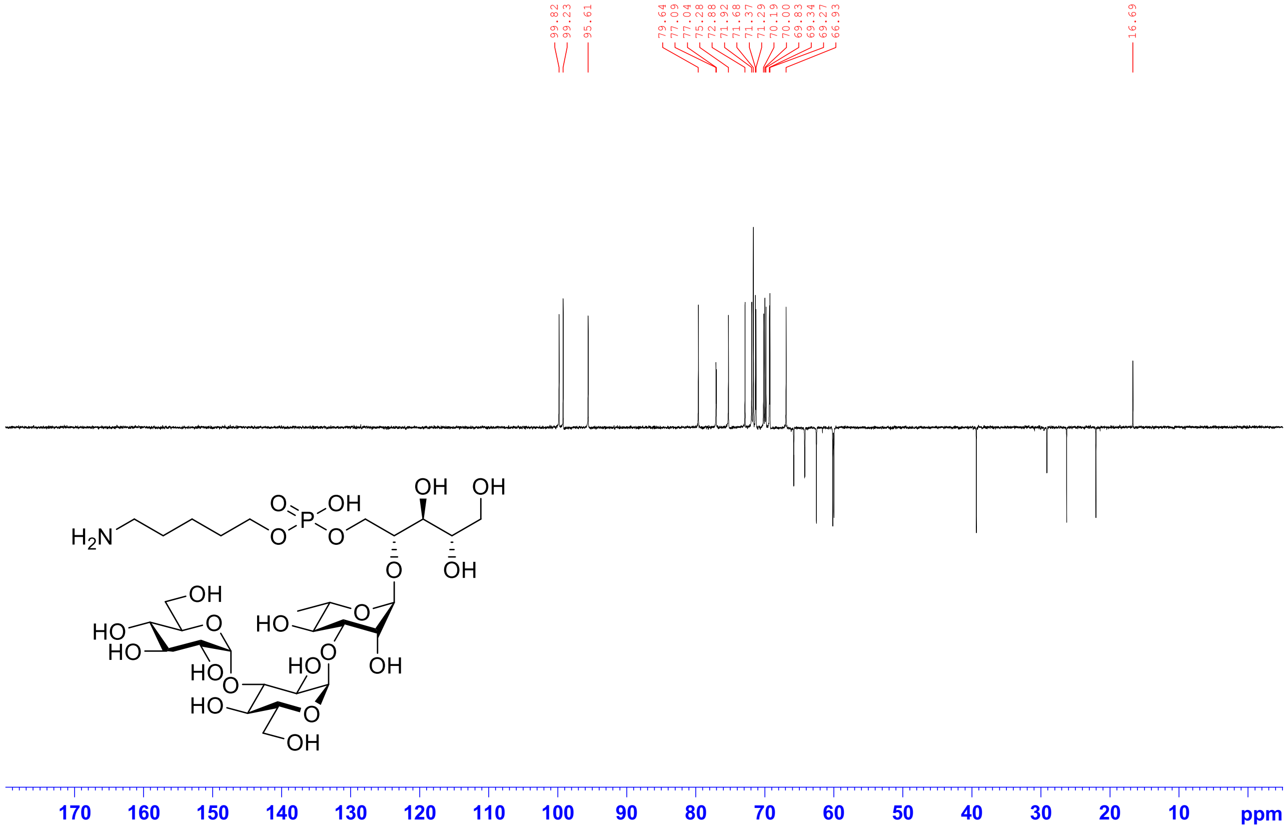

12: ^{31}P NMR (202 MHz, D_2O)

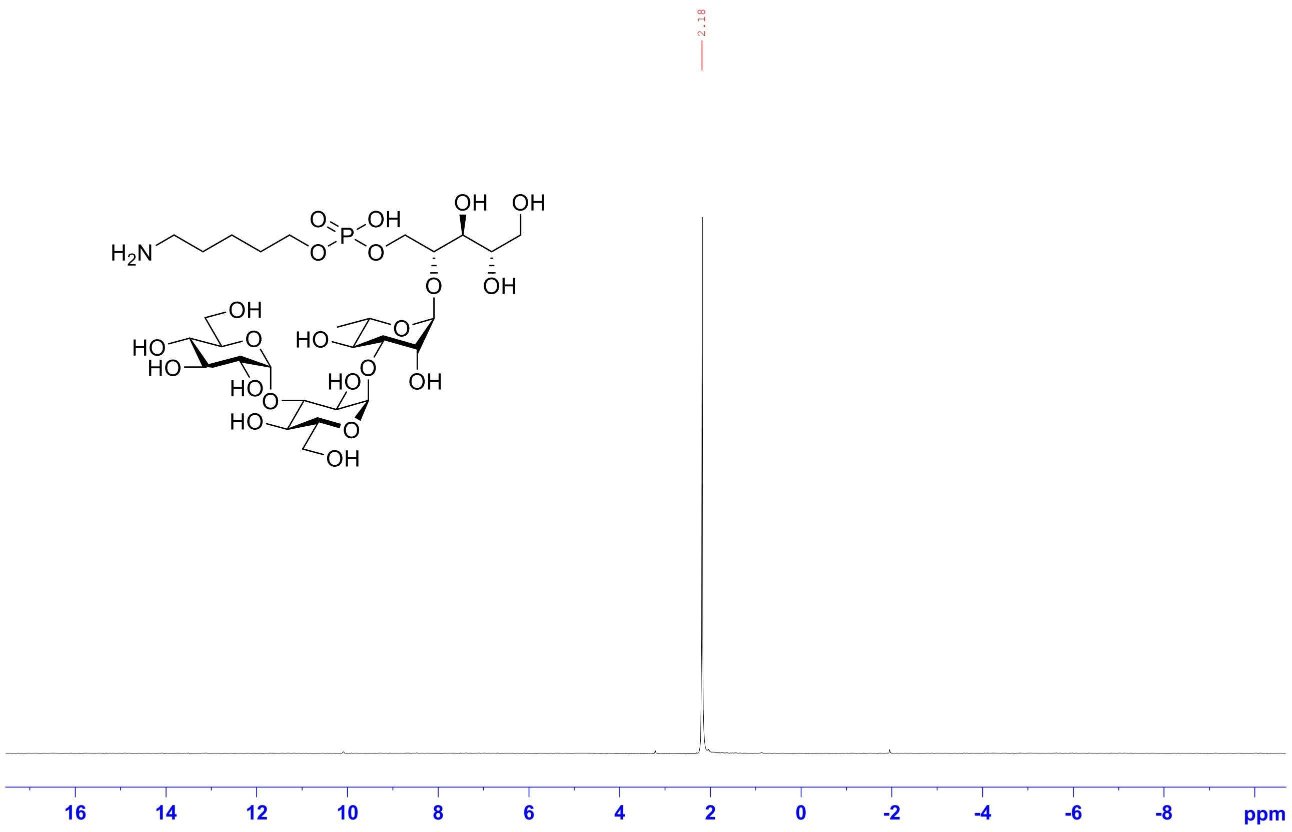

7: ^{1}H NMR (600 MHz, D_2O)

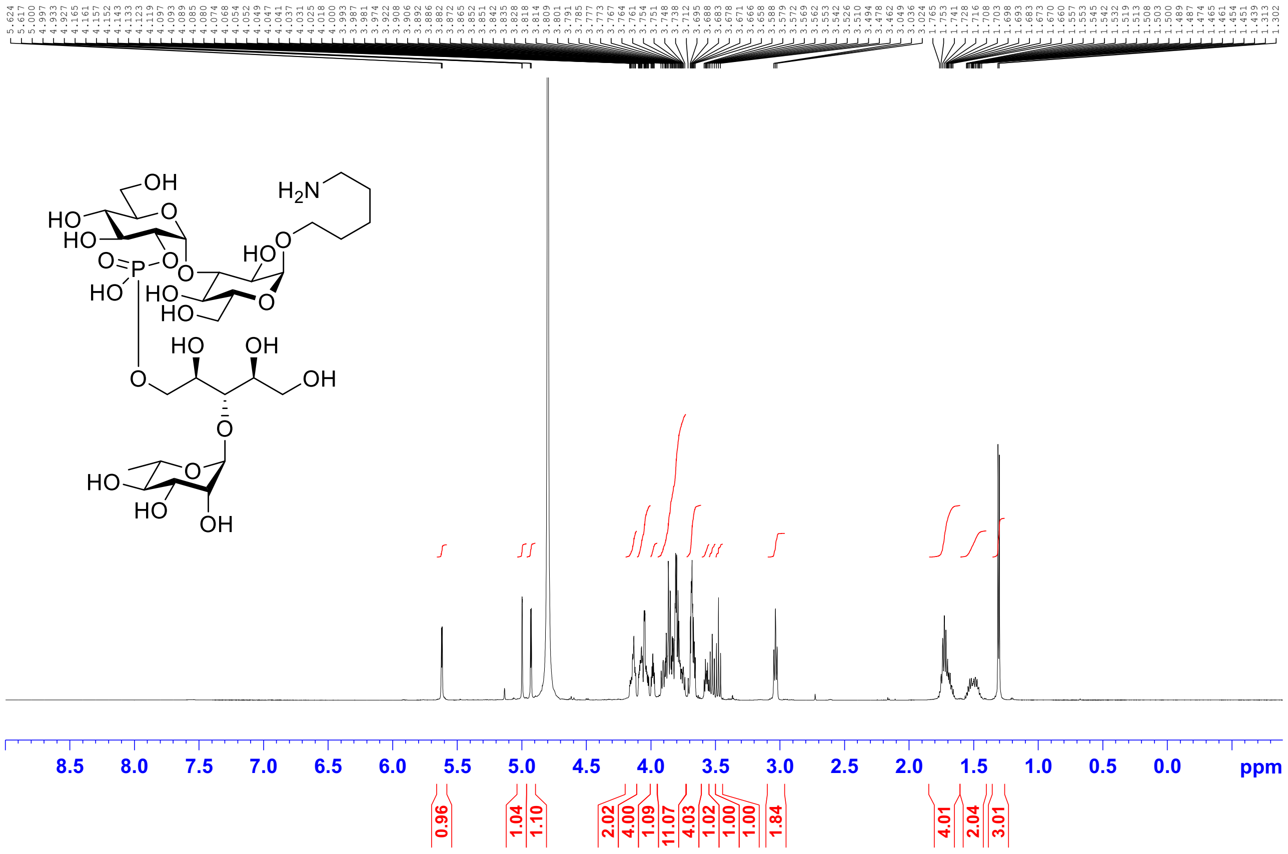

7: ^{13}C NMR (150 MHz, D_2O)

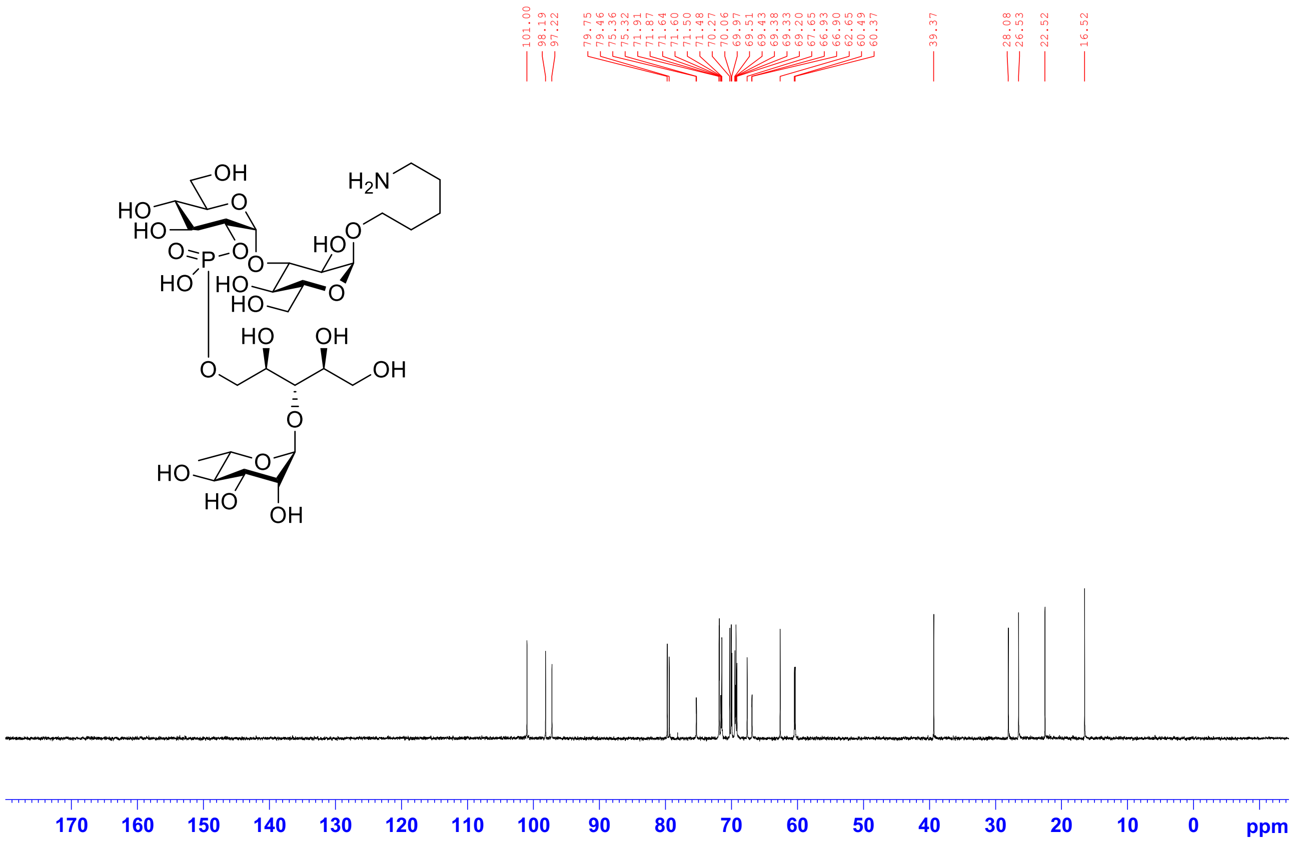

7: DEPT 135 (150 MHz, D_2O)

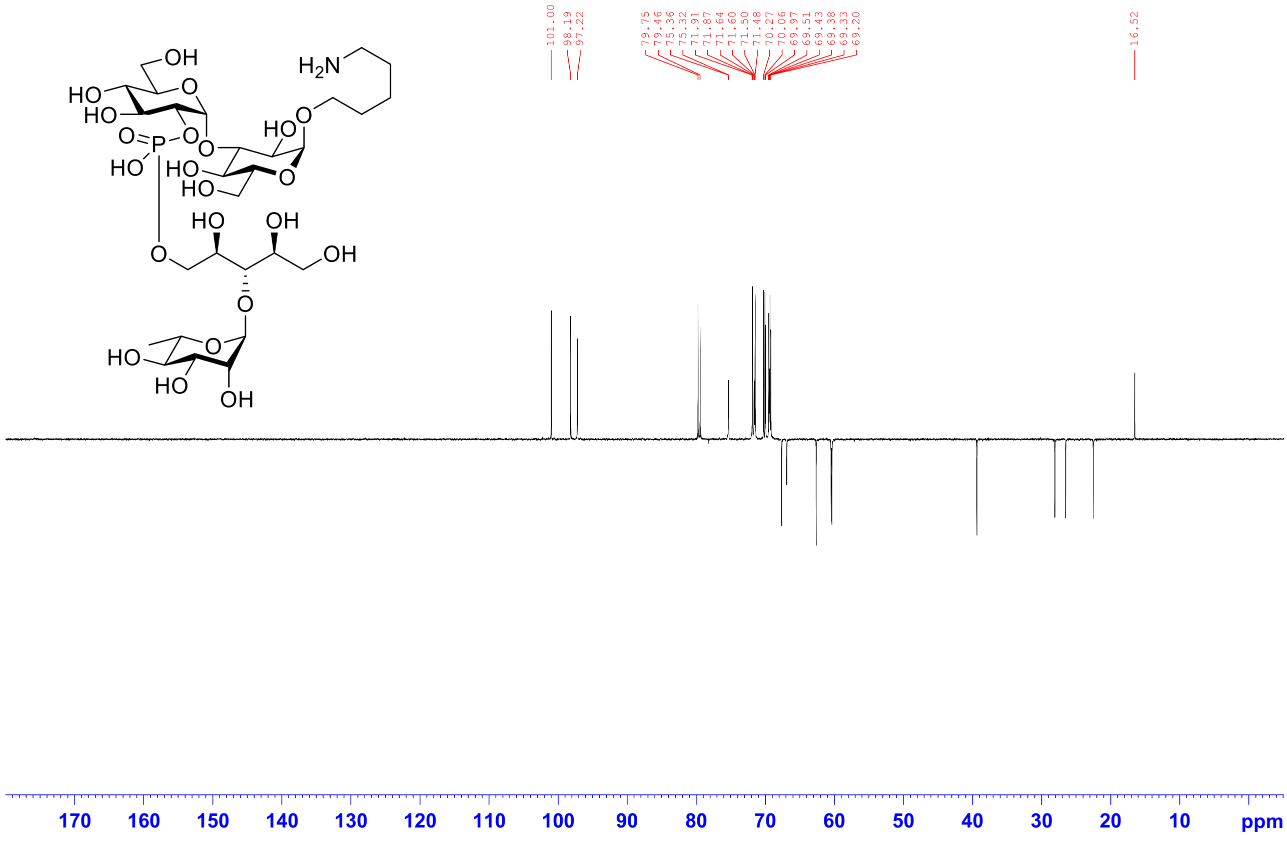

7: ^{31}P NMR (202 MHz, D_2O)

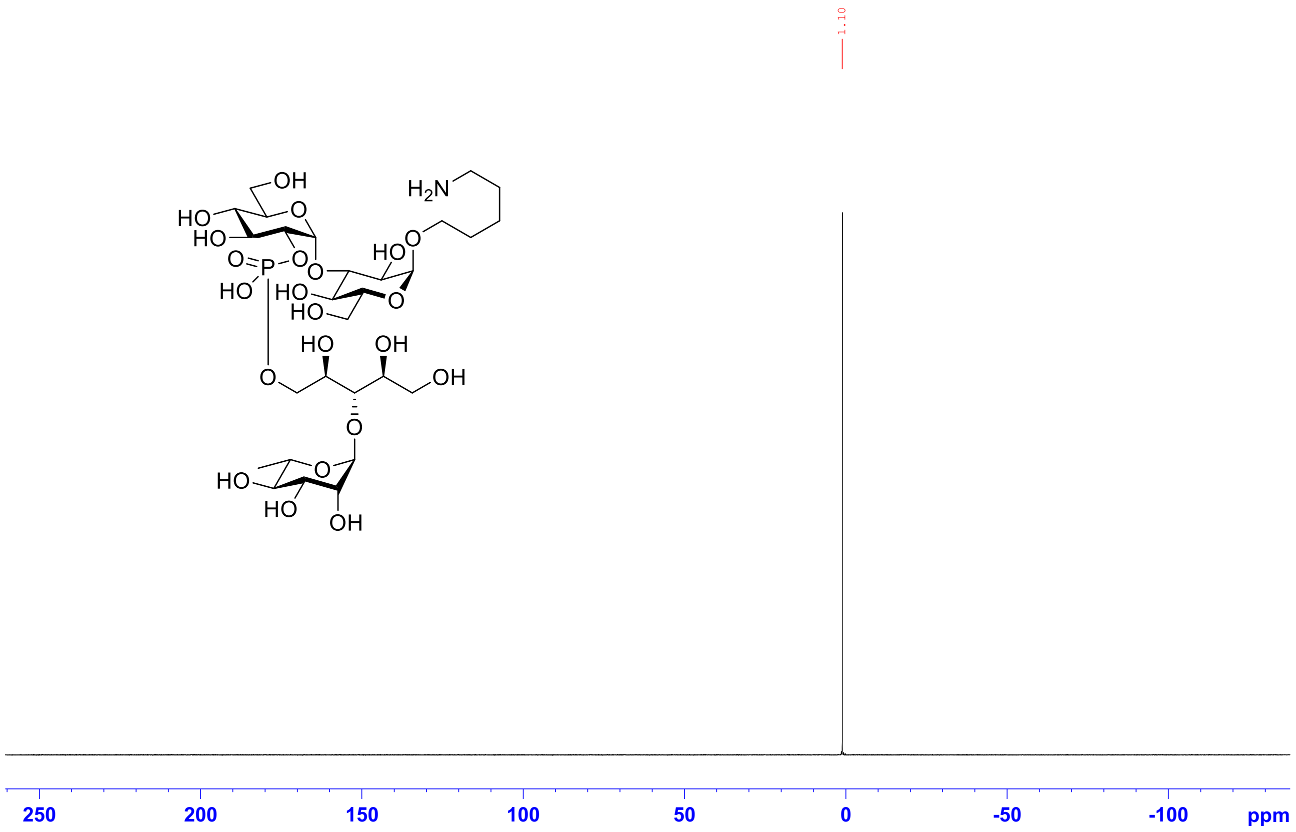

11: ^{1}H NMR (600 MHz, D_2O)

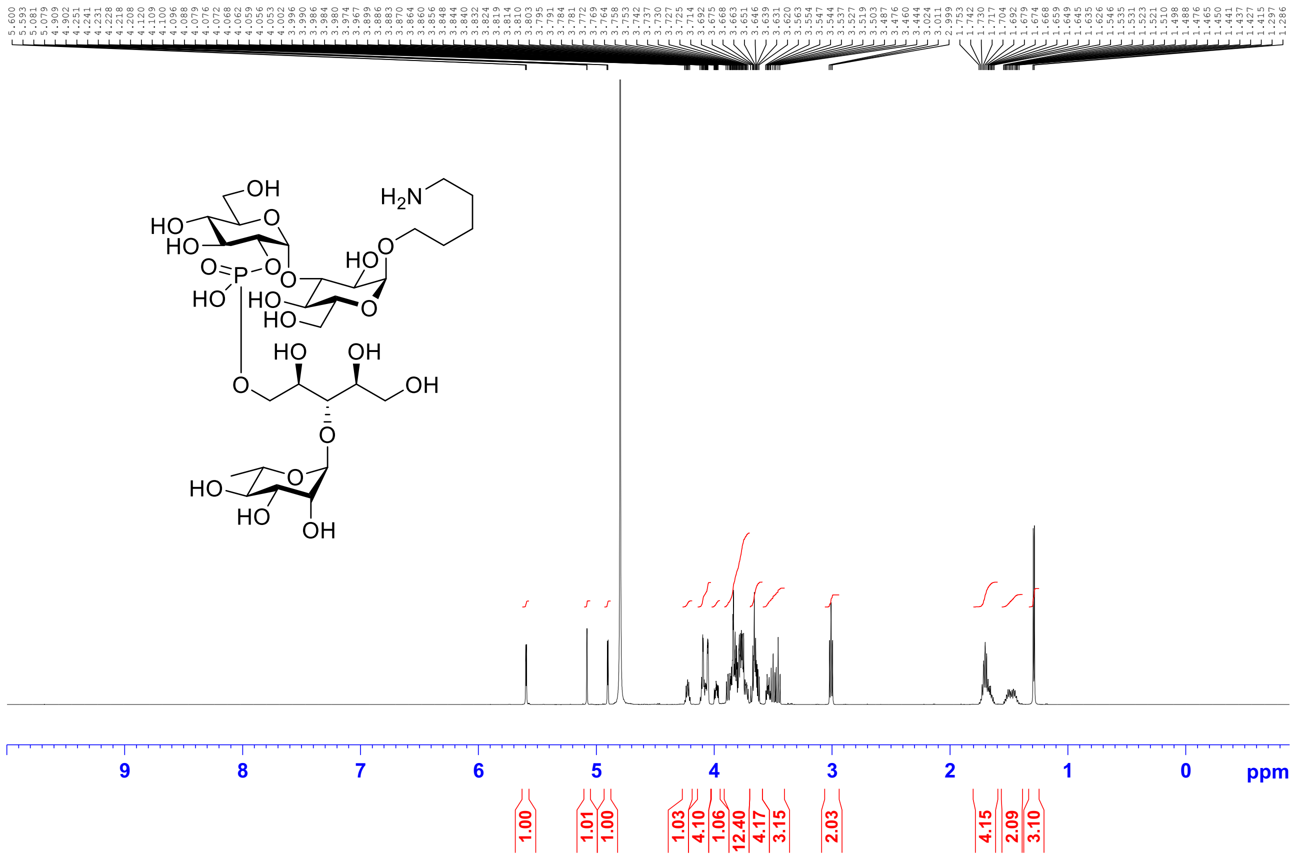

11: ^{13}C NMR (150 MHz, D_2O)

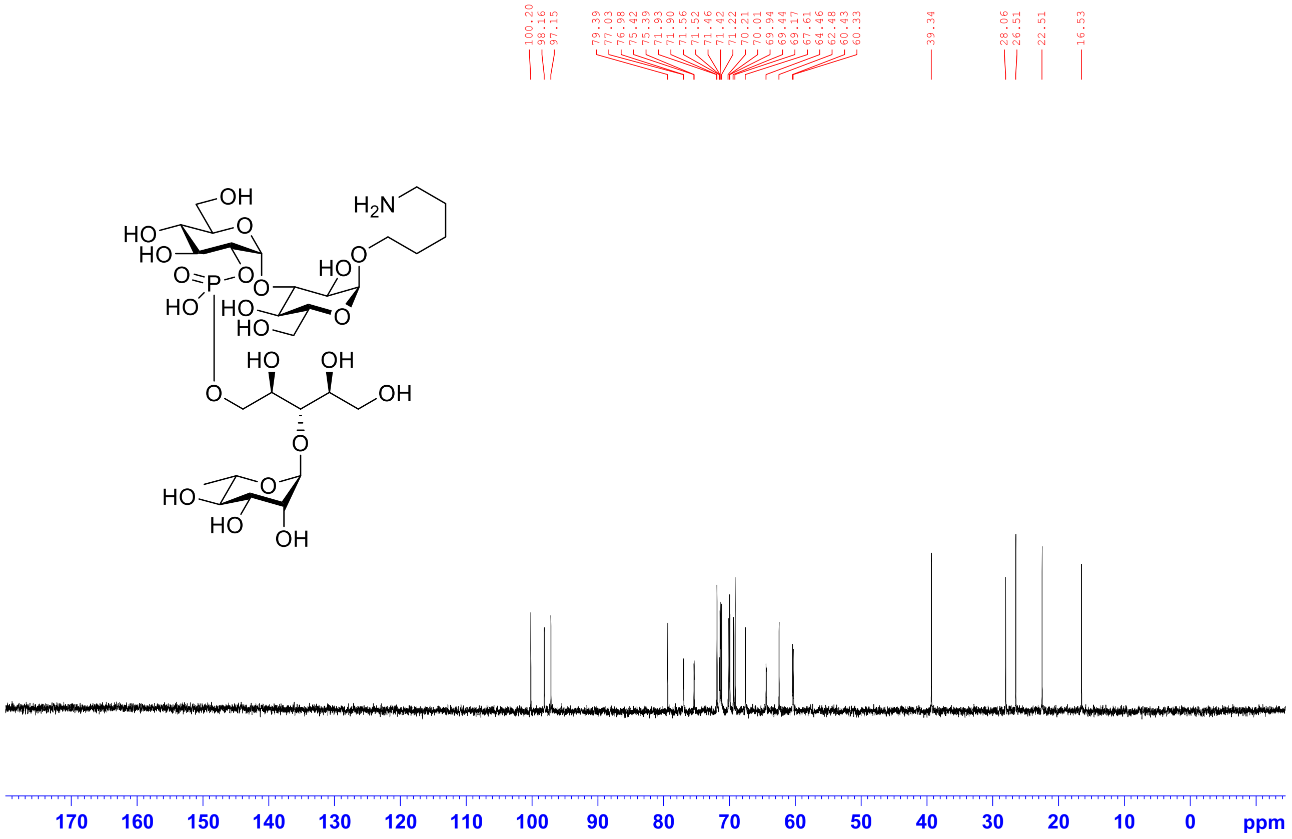

11: DEPT 135 (150 MHz, D_2O)

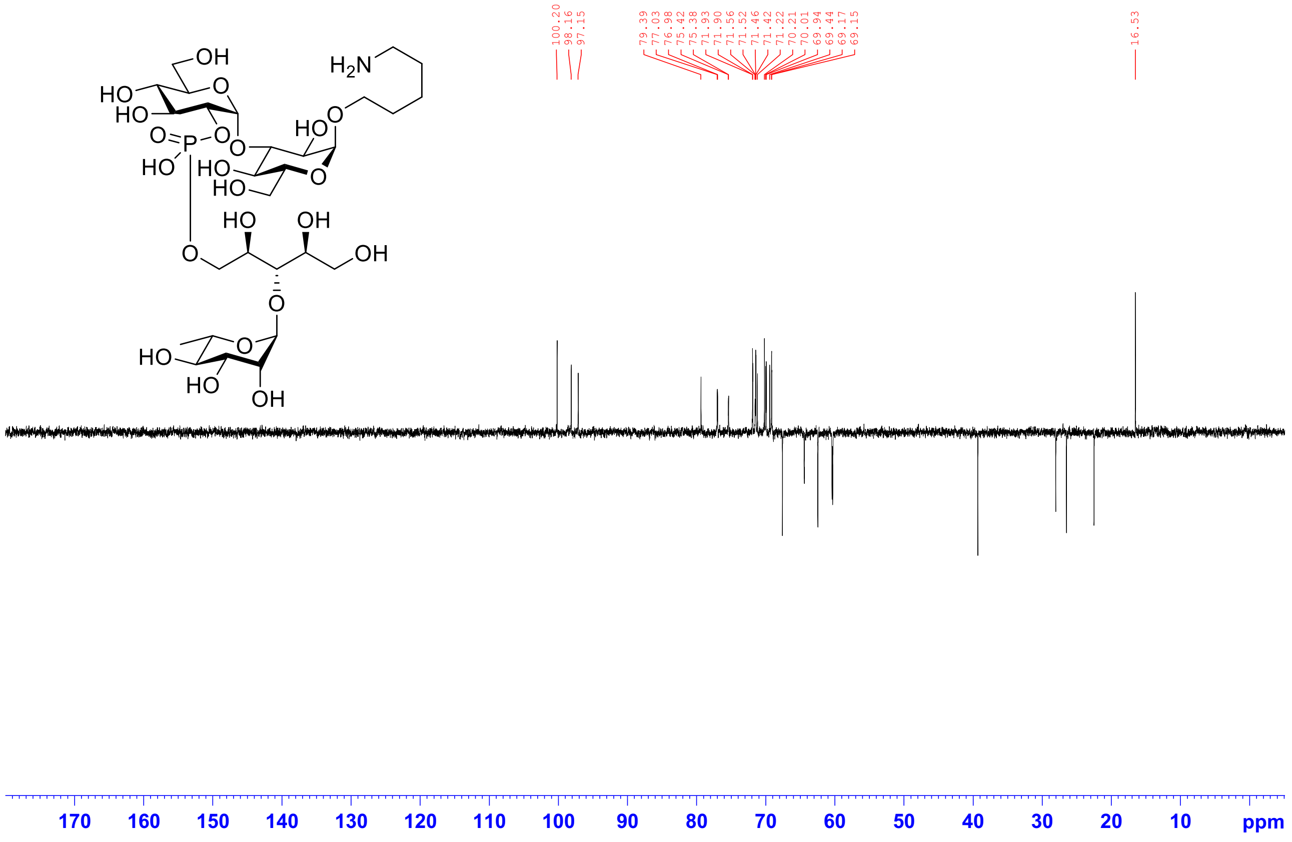

11: ^{31}P NMR (202 MHz, D_2O)

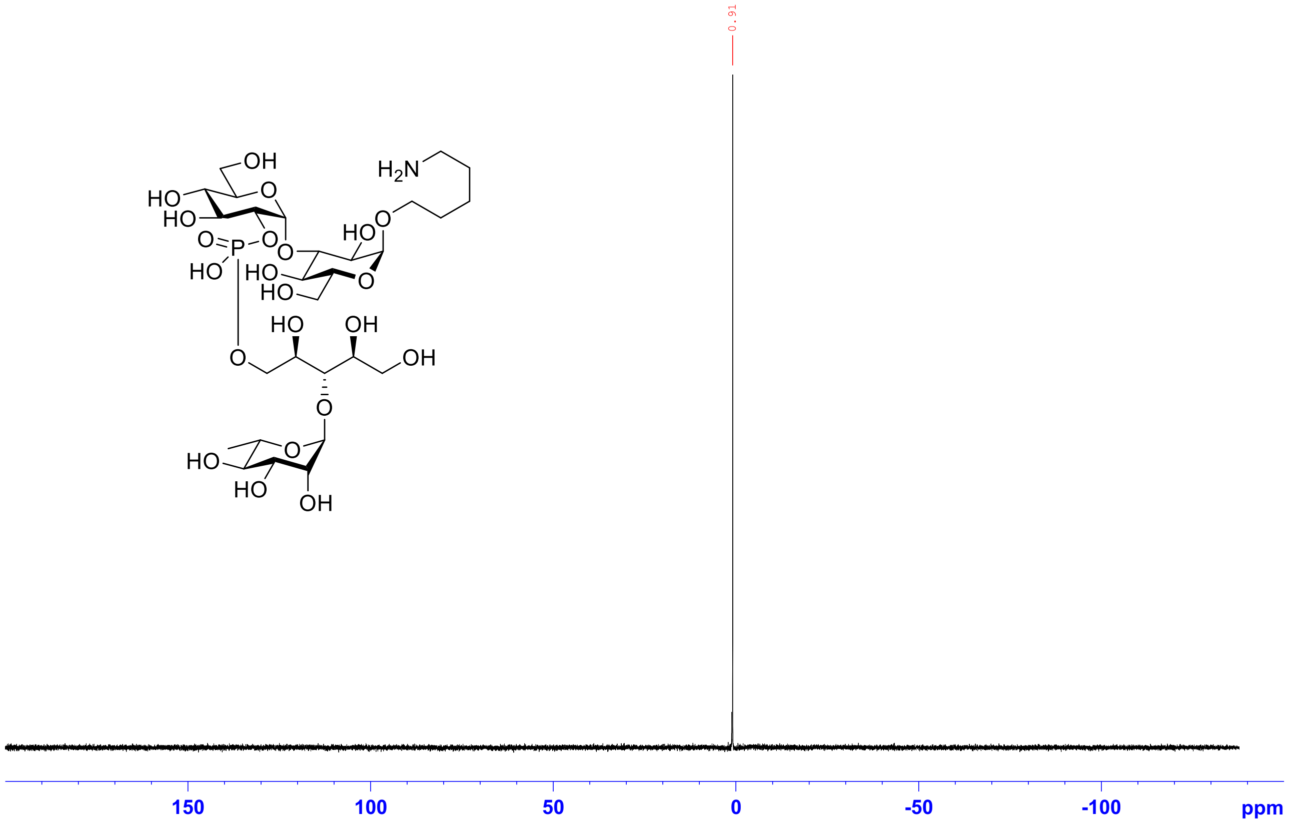

13: ^{1}H NMR (600 MHz, D_2O)

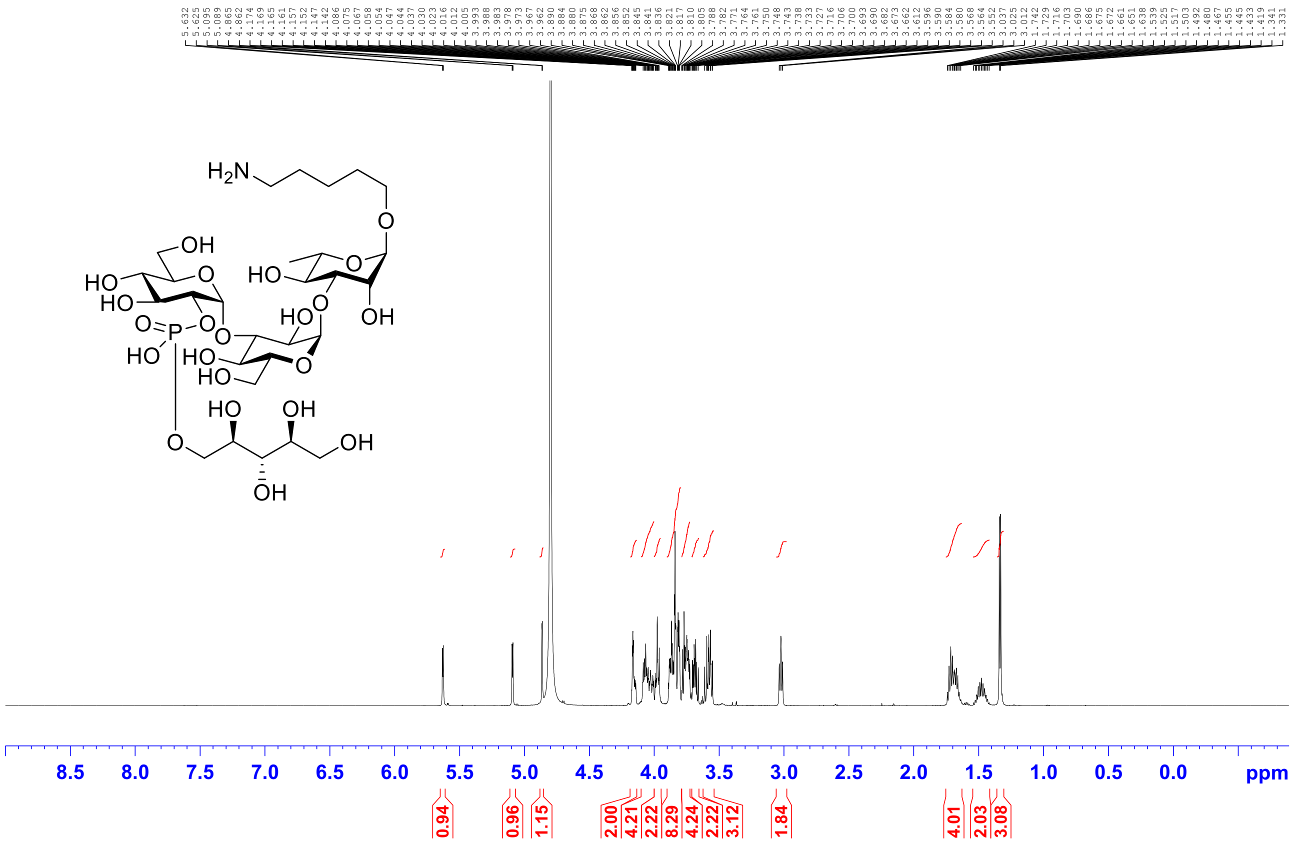

13: ^{13}C NMR (150 MHz, D_2O)

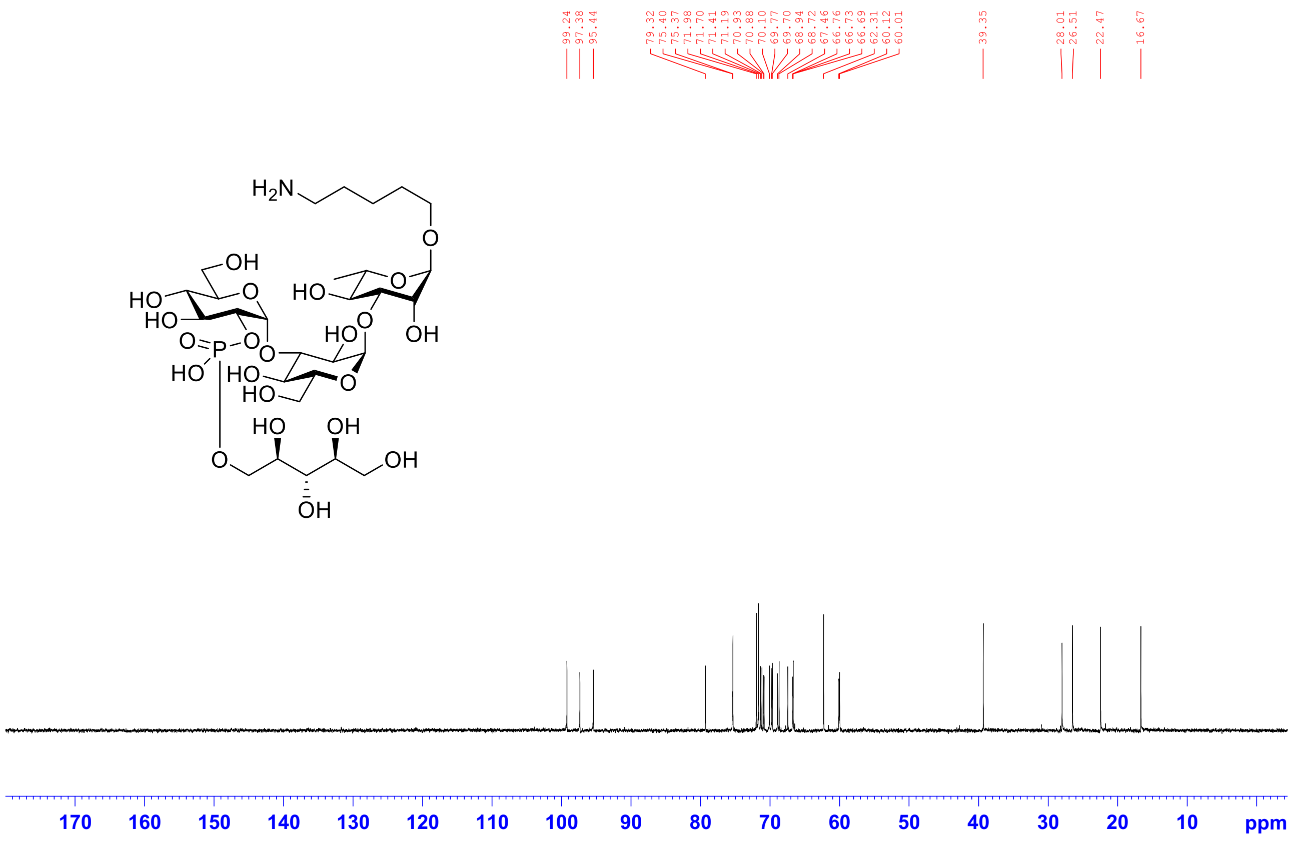

13: DEPT 135 (150 MHz, D_2O)

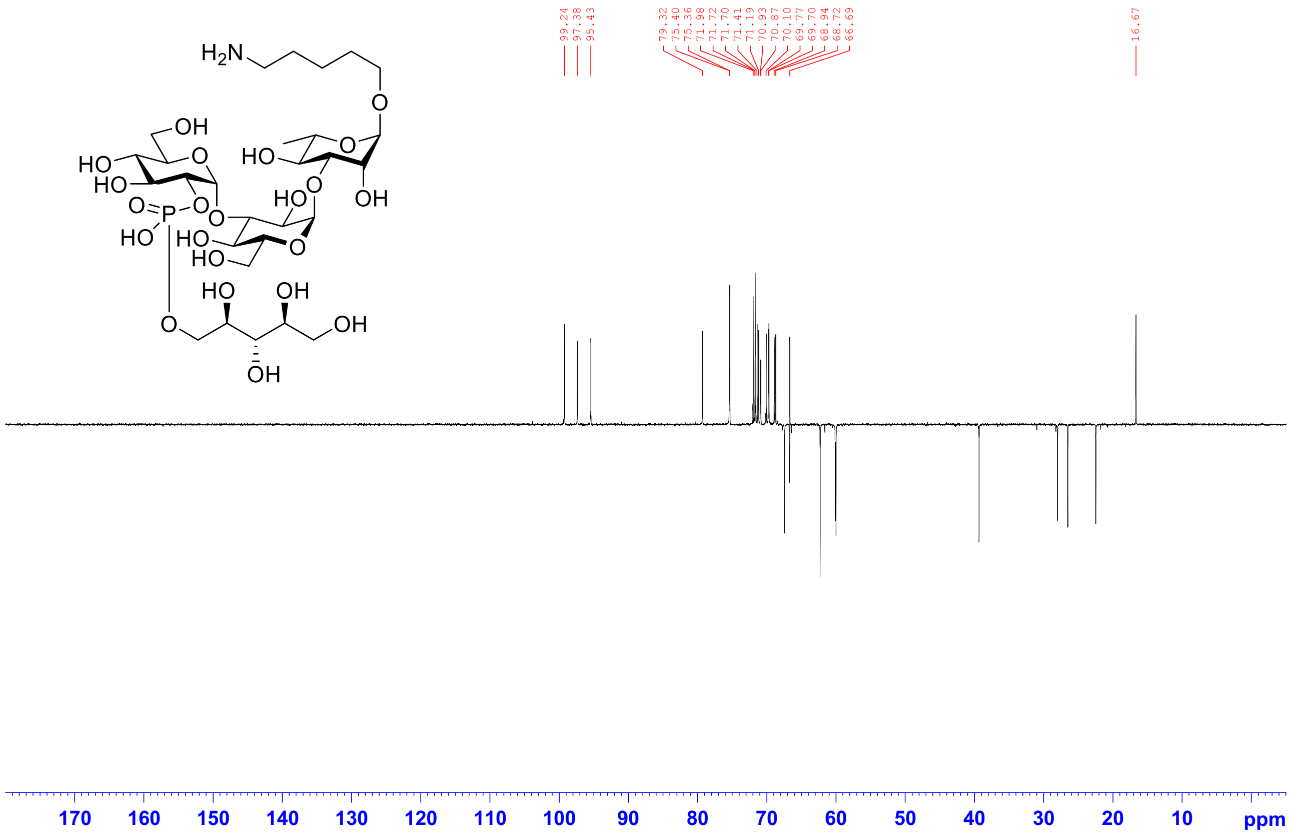

13: ^{31}P NMR (202 MHz, D_2O)

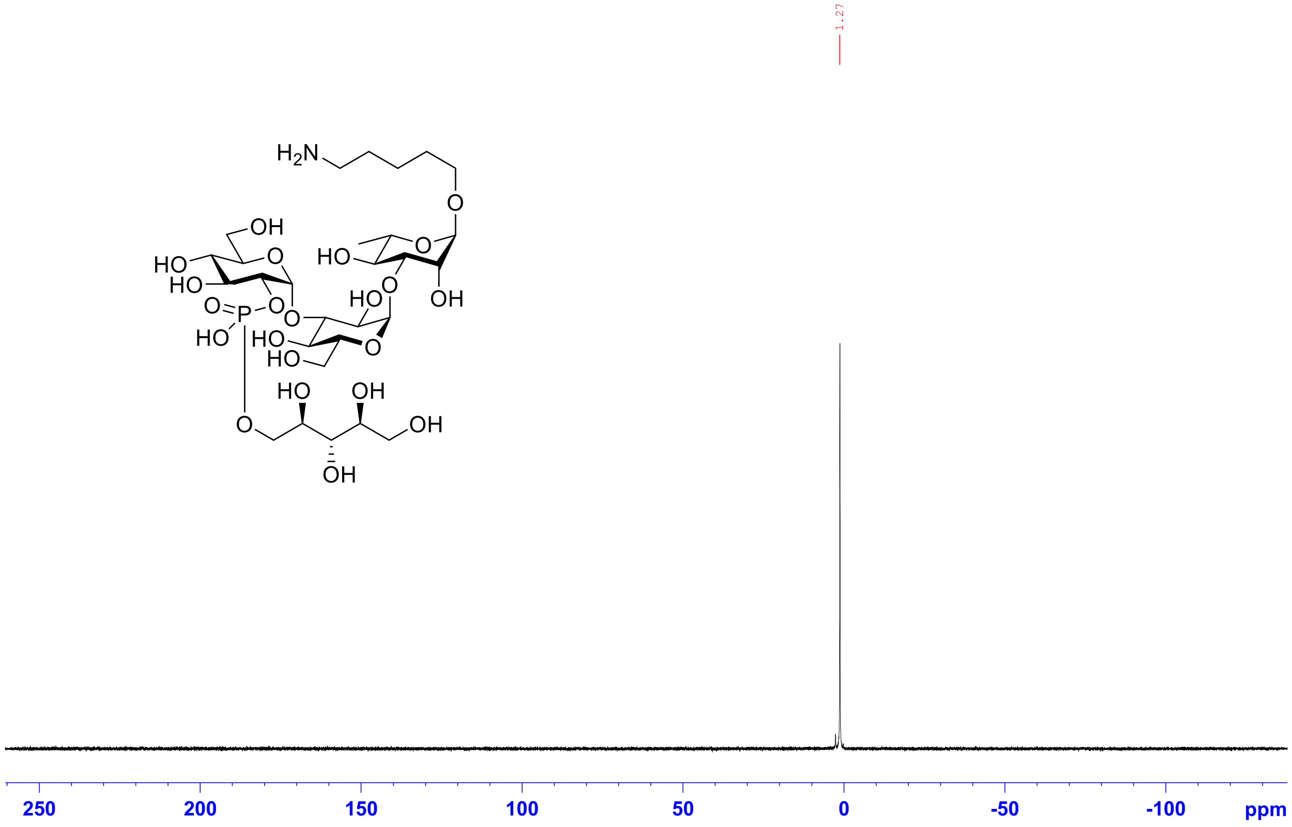

NMR spectra of thiolated pseudo-tetrasaccharides

5-SH: ^{1}H NMR (600 MHz, D_2O)

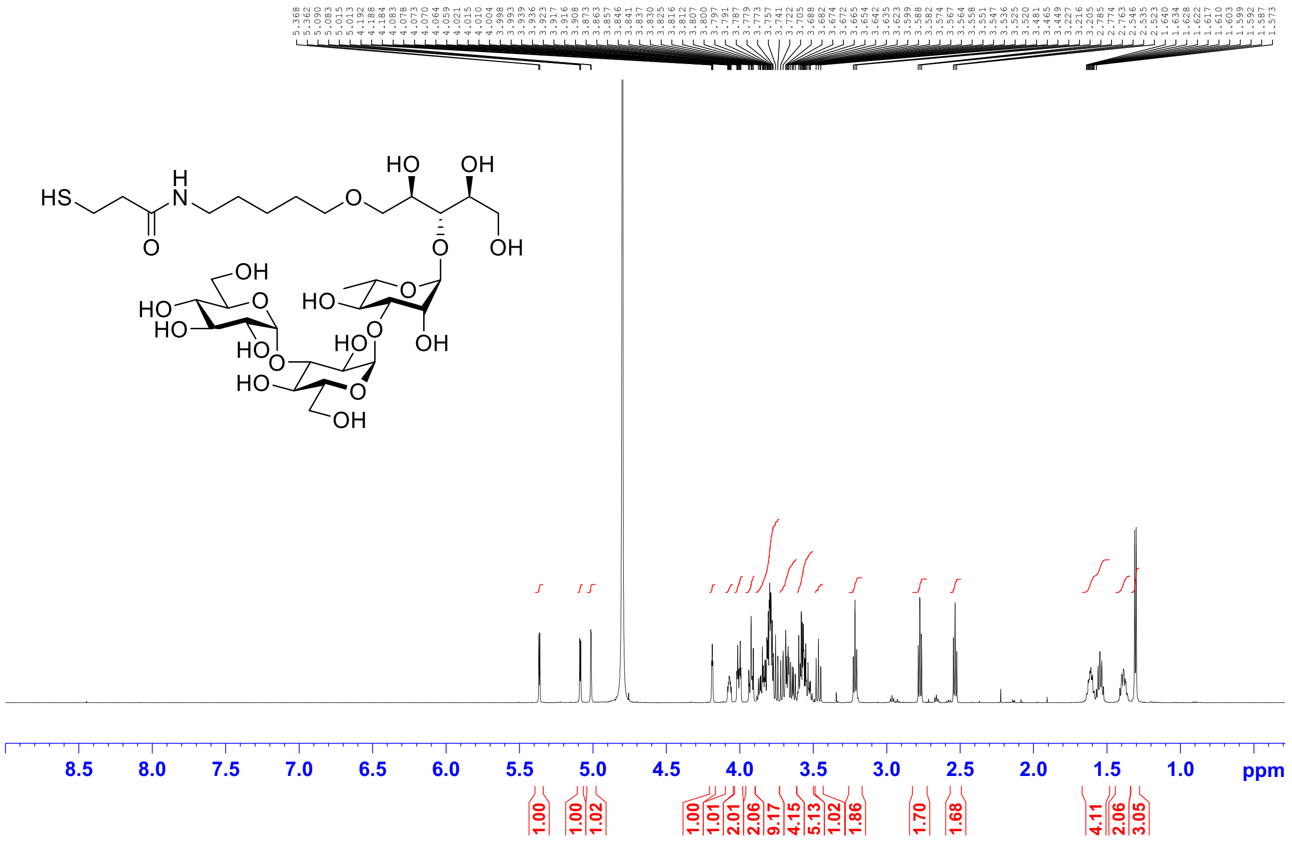

5-SH: ^{13}C NMR (150 MHz, D_2O)

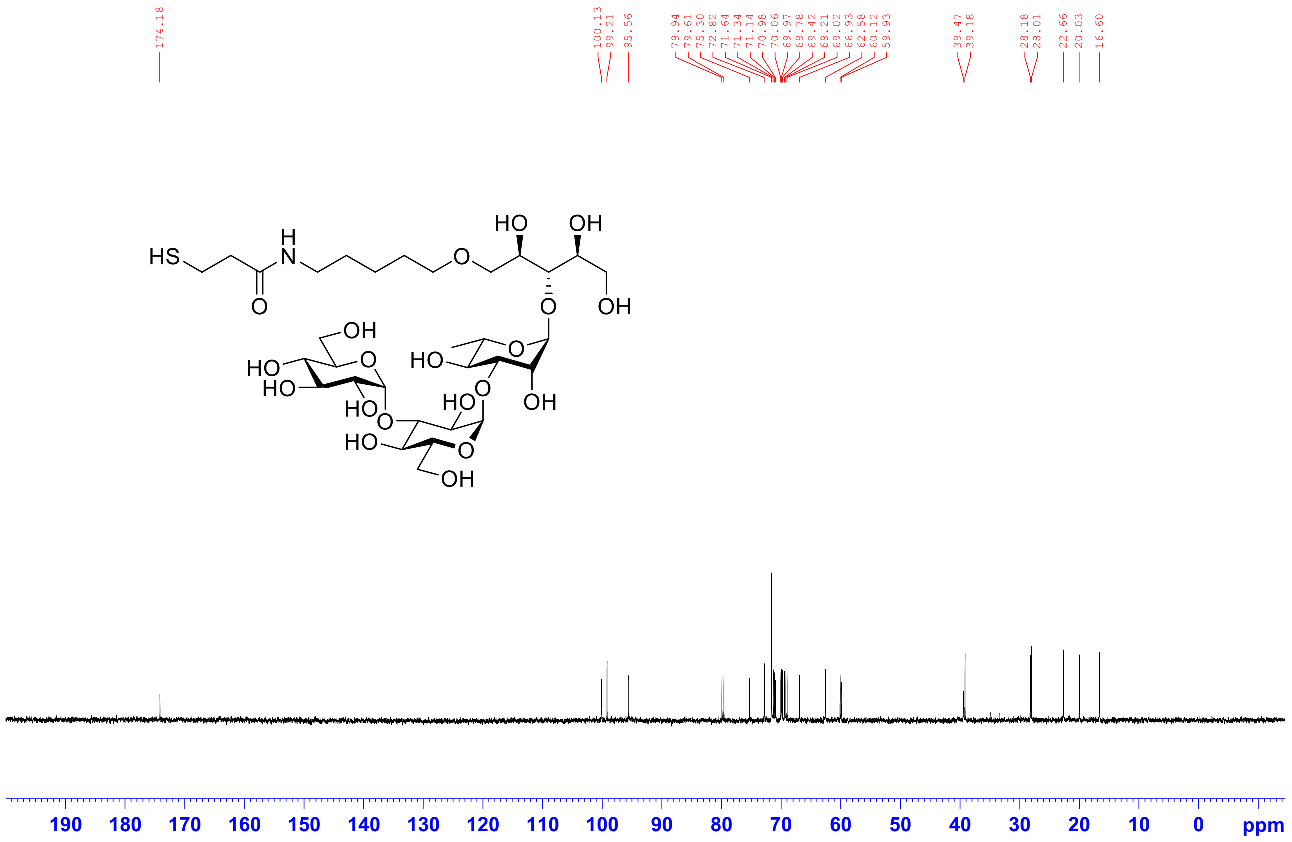

5-SH: DEPT 135 (150 MHz, D_2O)

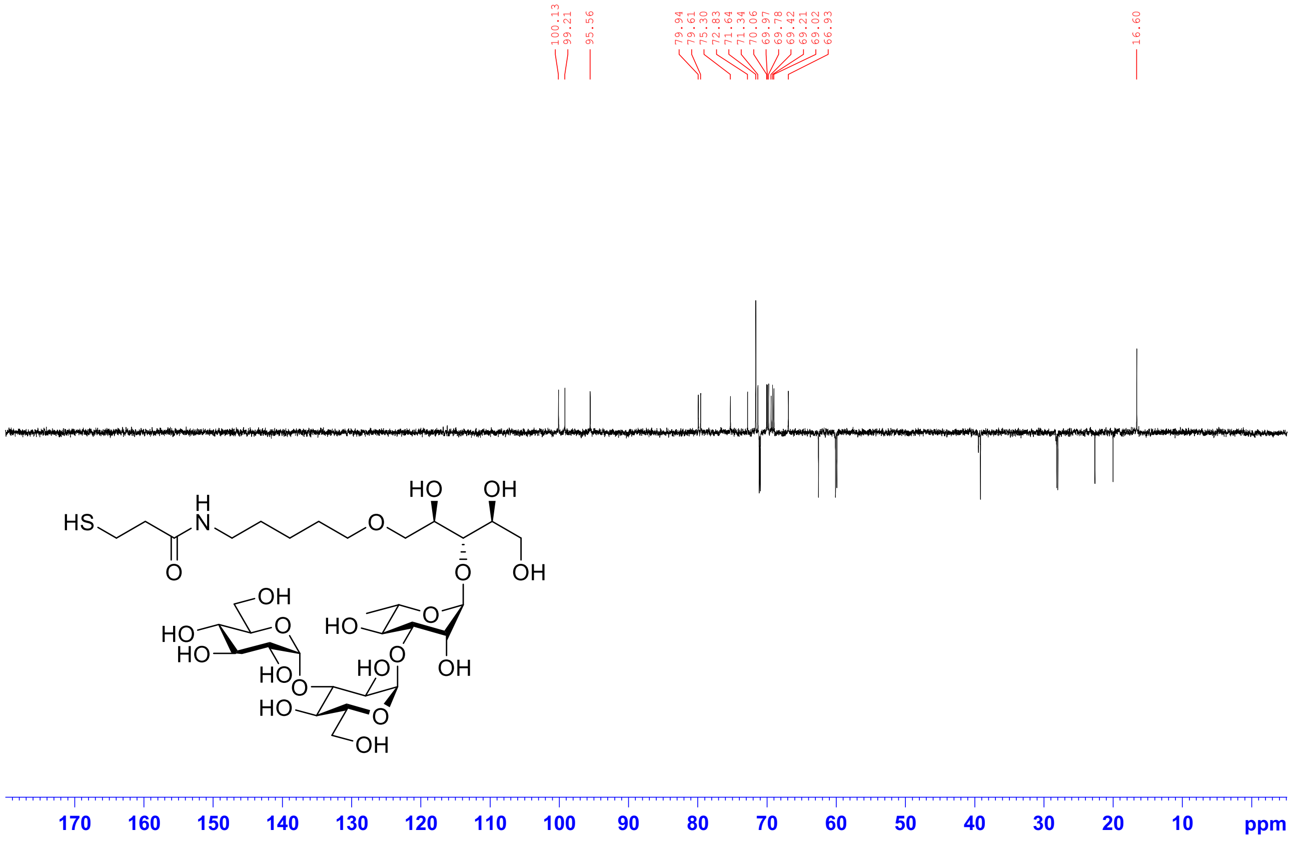

6-SH: ^{1}H NMR (600 MHz, D_2O)

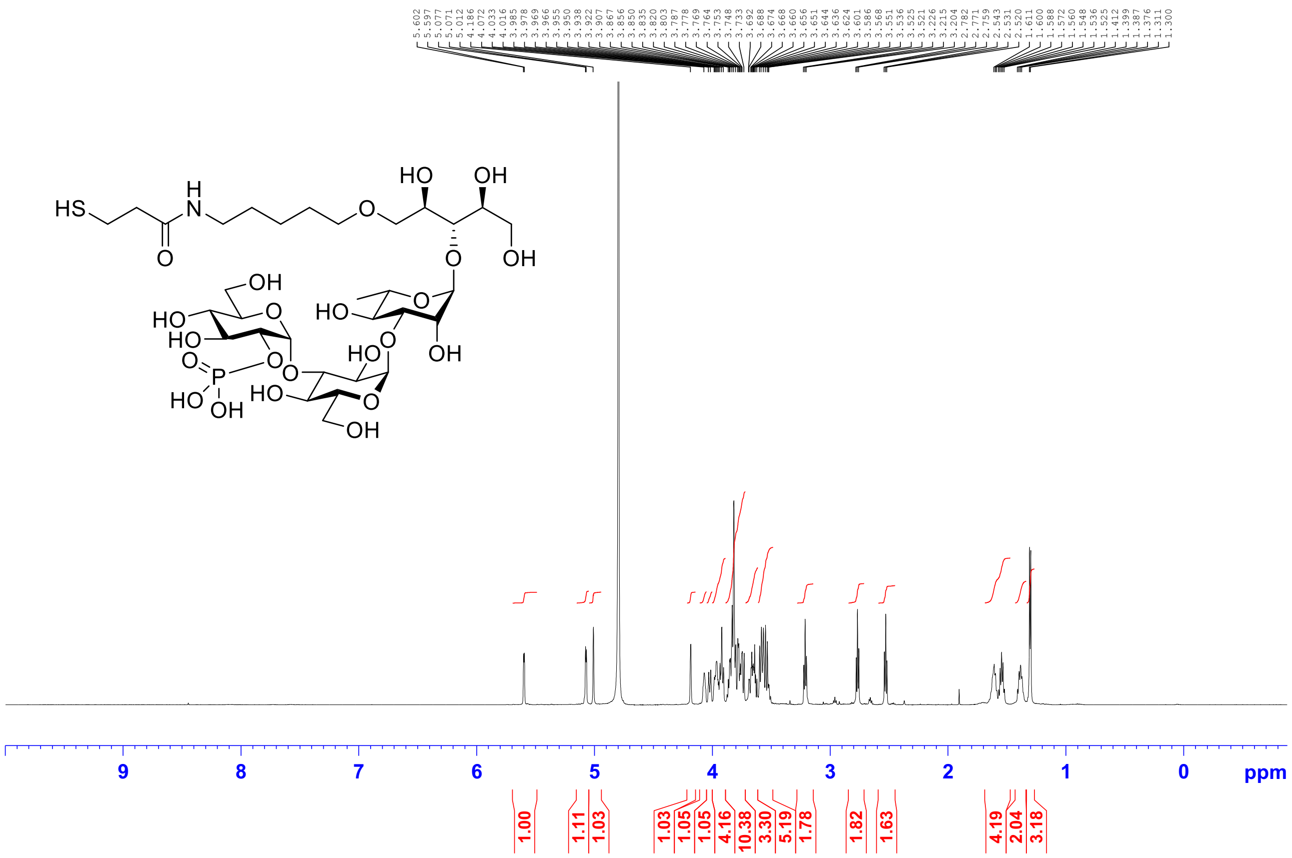

6-SH: ^{13}C NMR (150 MHz, D_2O)

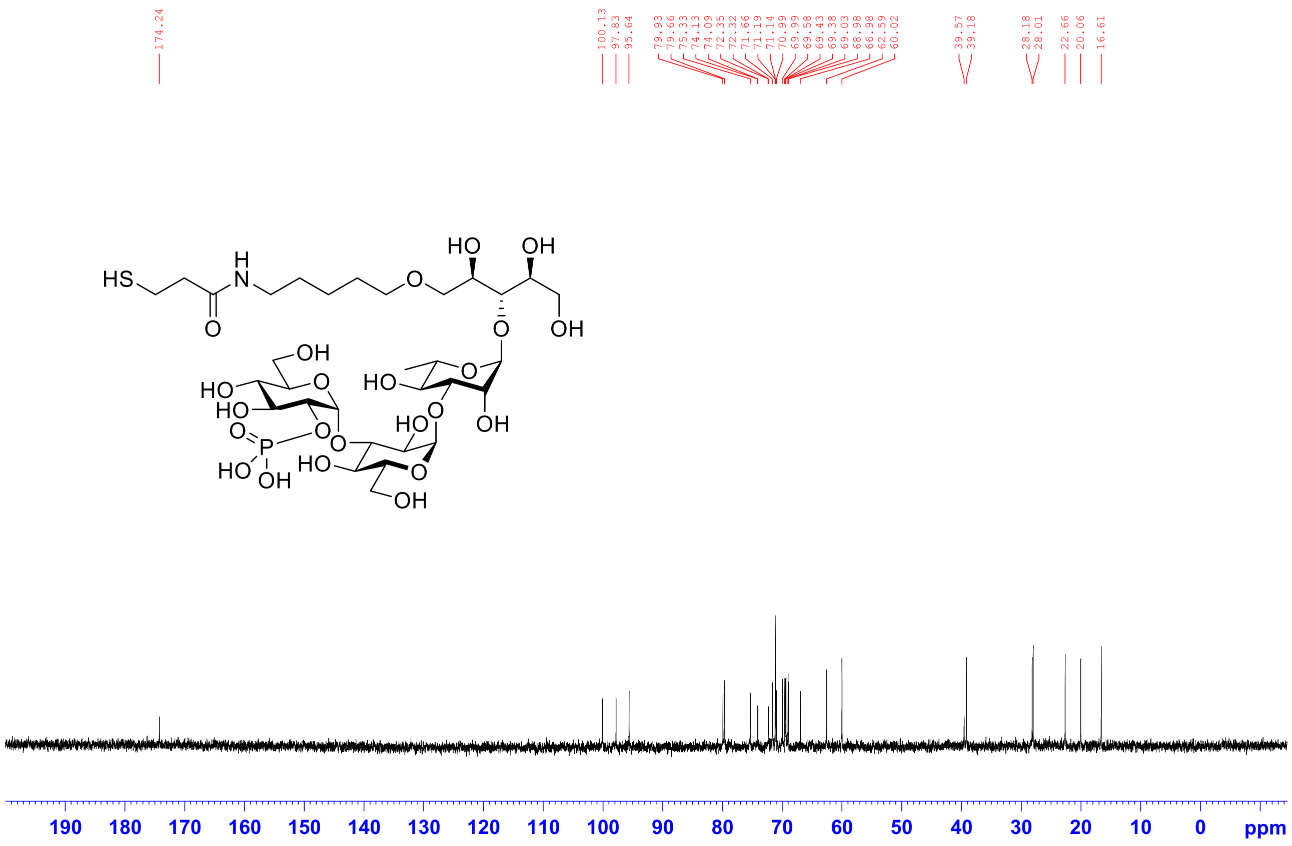

6-SH: DEPT 135 (150 MHz, D_2O)

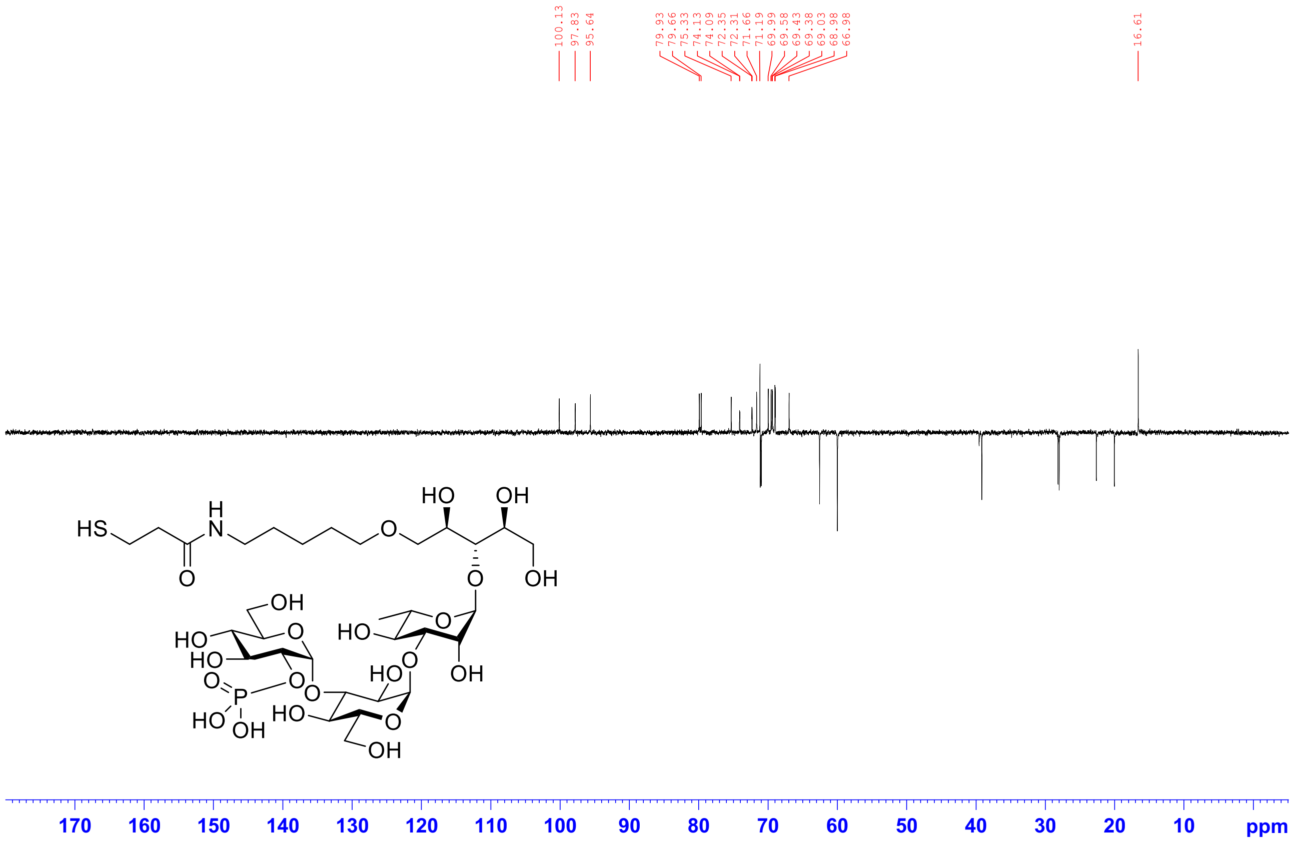

9-SH: ^{1}H NMR (600 MHz, D_2O)

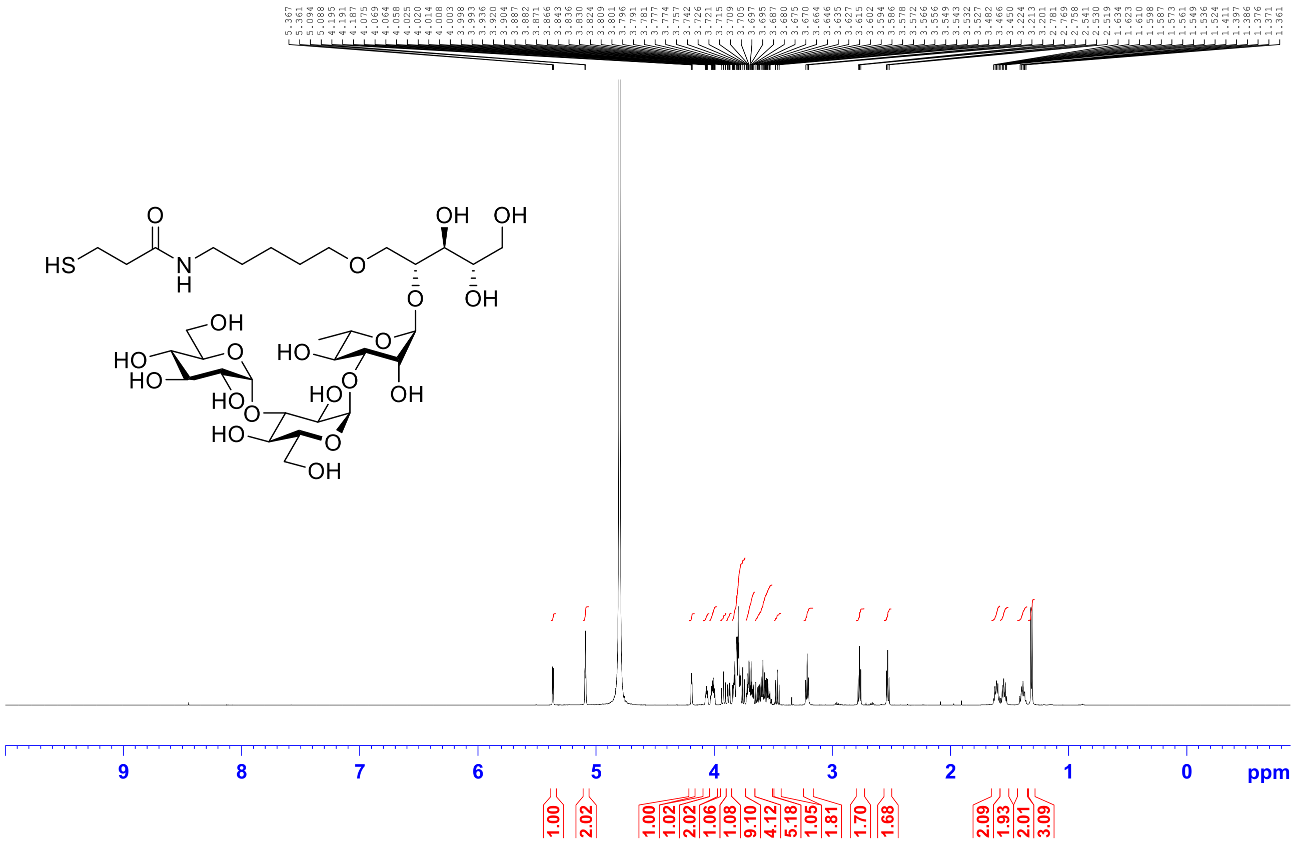

9-SH: ^{13}C NMR (150 MHz, D_2O)

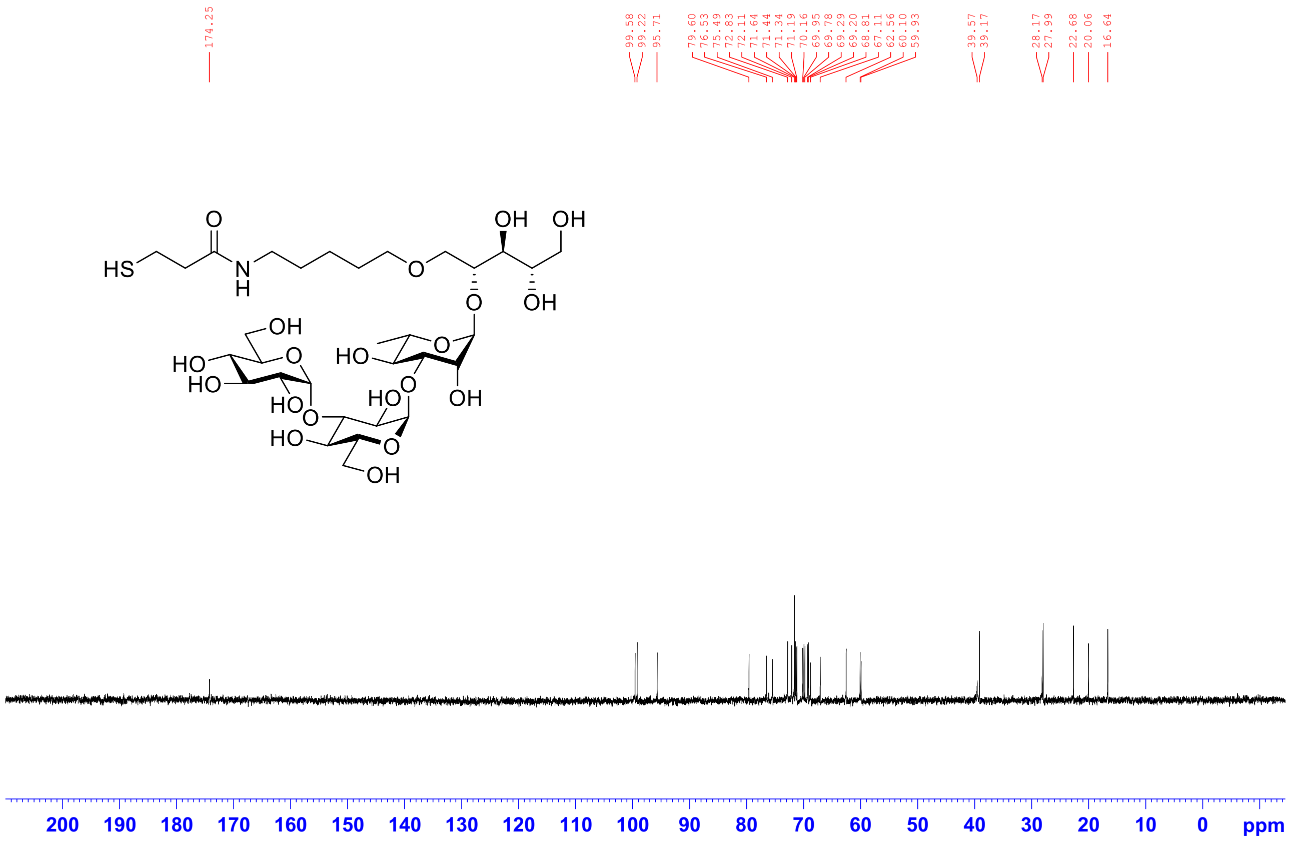

9-SH: DEPT 135 (150 MHz, D_2O)

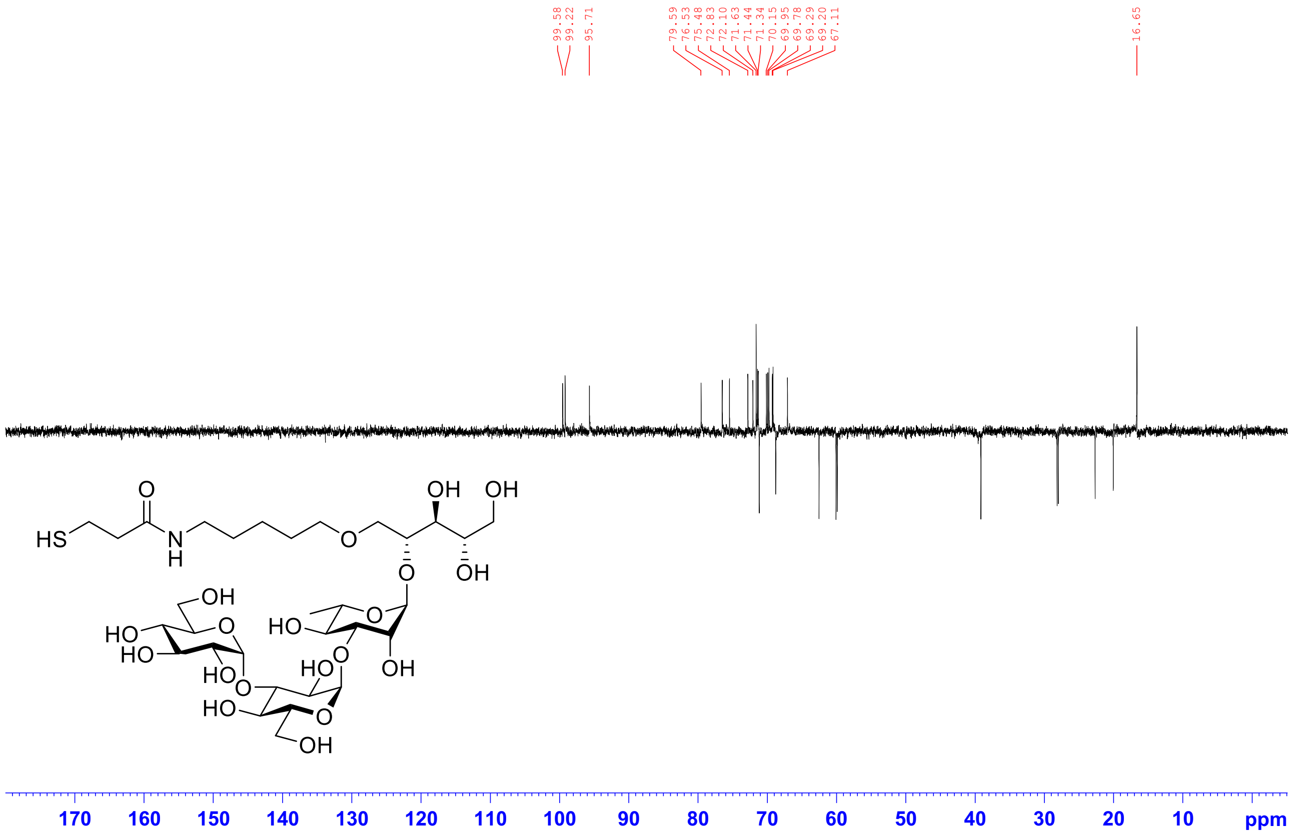

10-SH: ^{1}H NMR (600 MHz, D_2O)

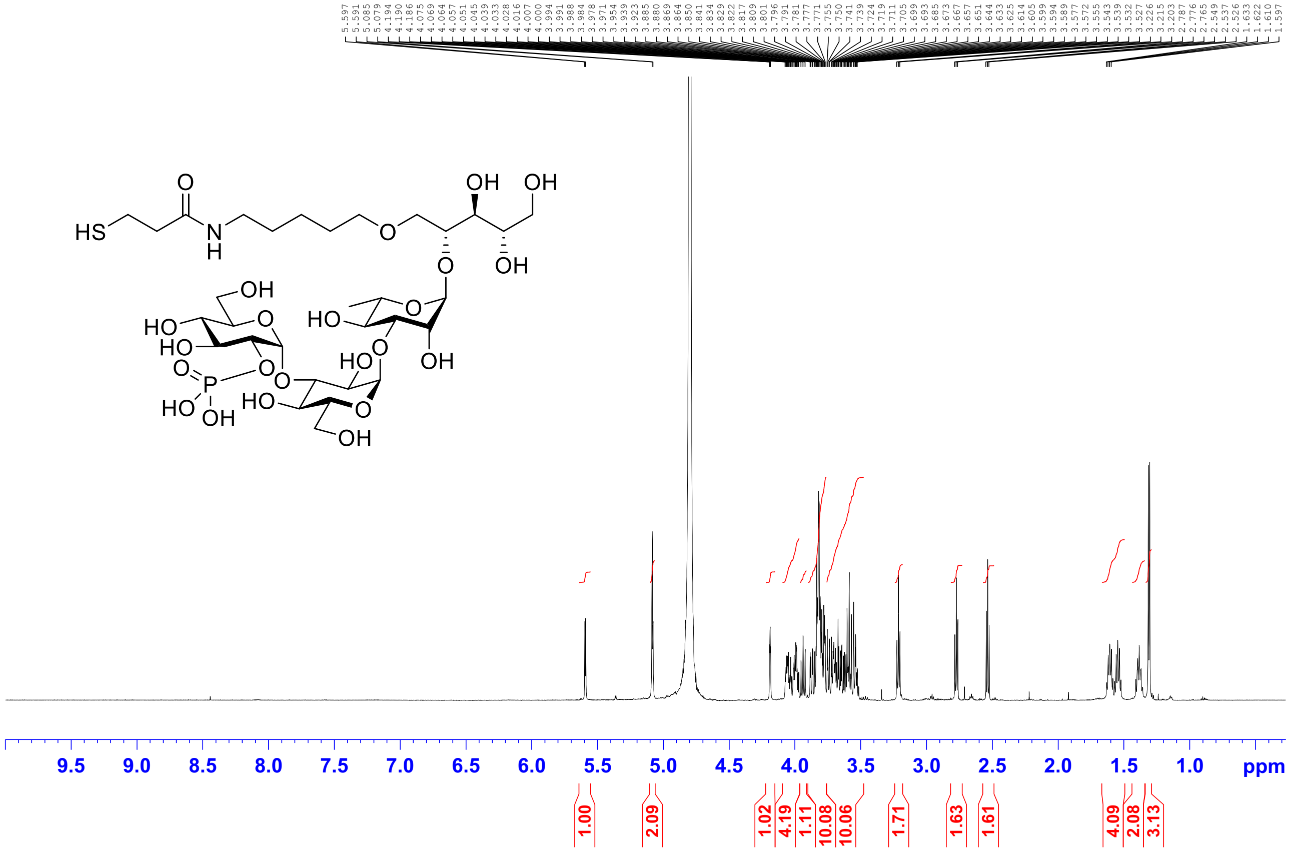

10-SH: ^{13}C NMR (150 MHz, D_2O)

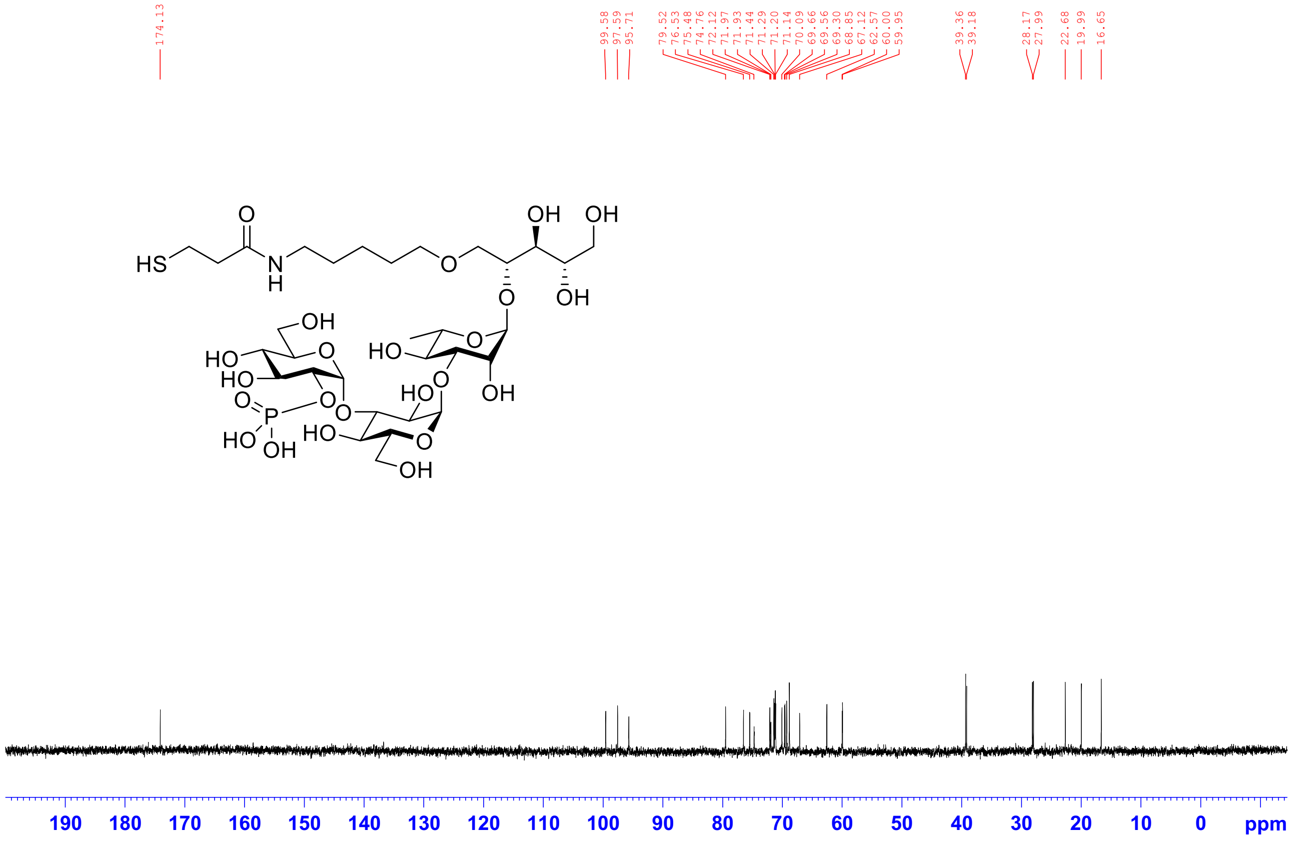

10-SH: DEPT 135 (150 MHz, D_2O)

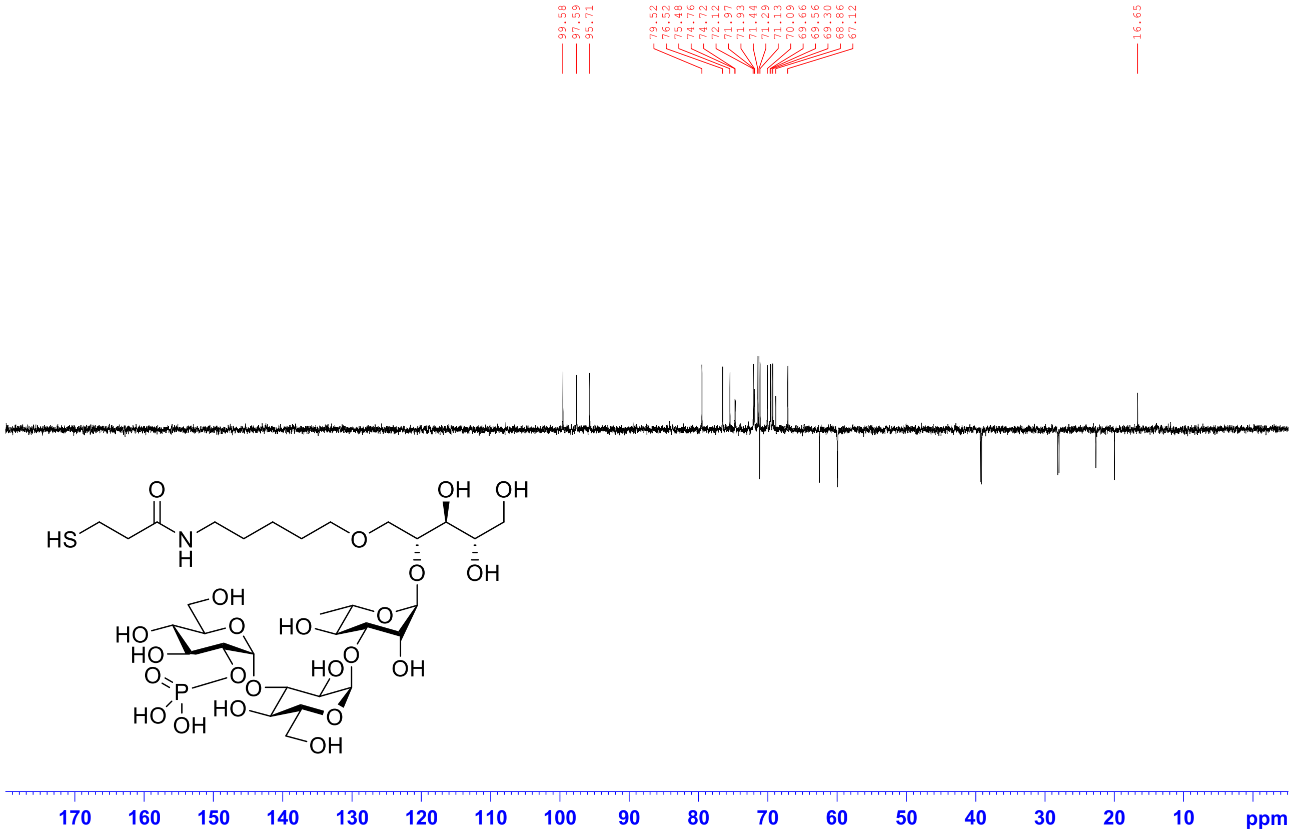

7-SH: ^{1}H NMR (600 MHz, D_2O)

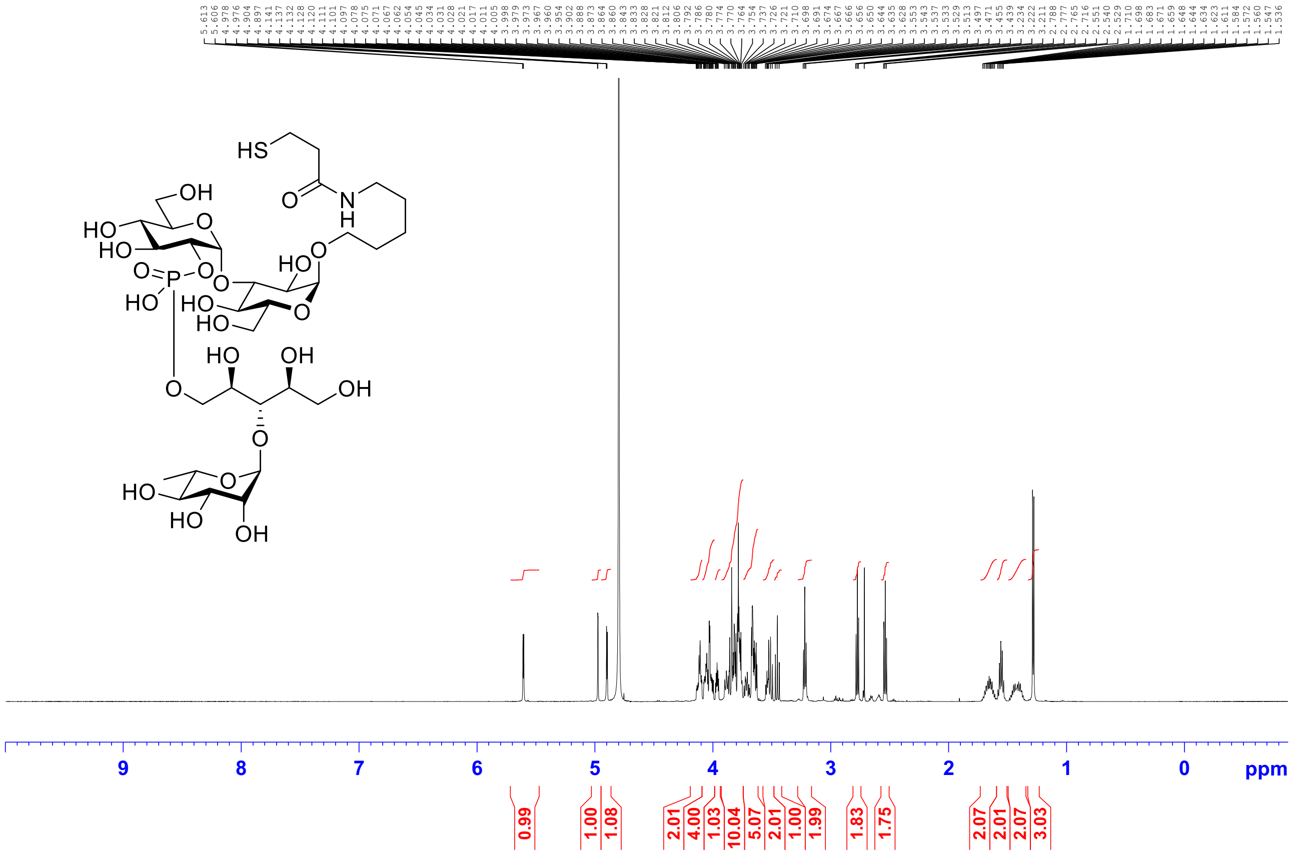

7-SH: ^{13}C NMR (150 MHz, D_2O)

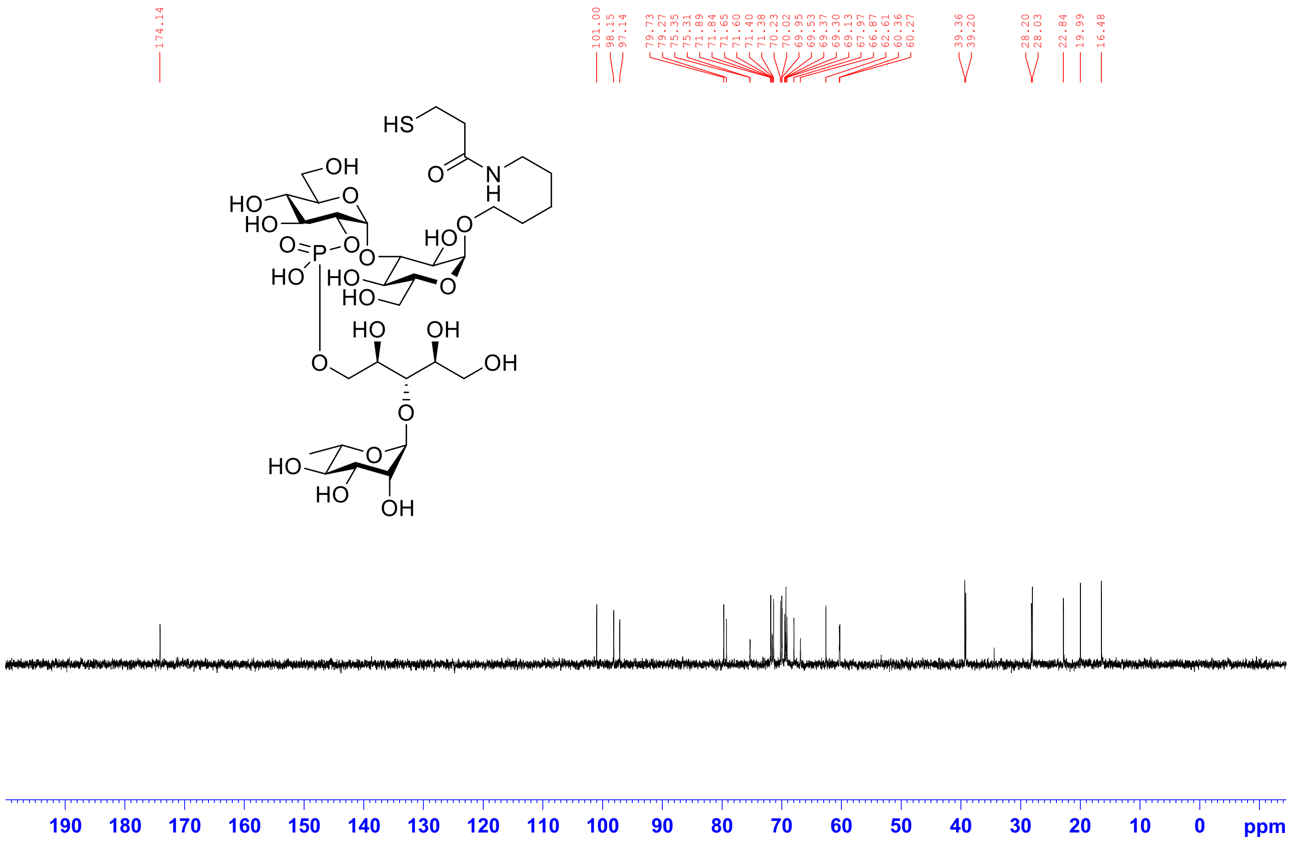

7-SH: DEPT 135 (150 MHz, D_2O)

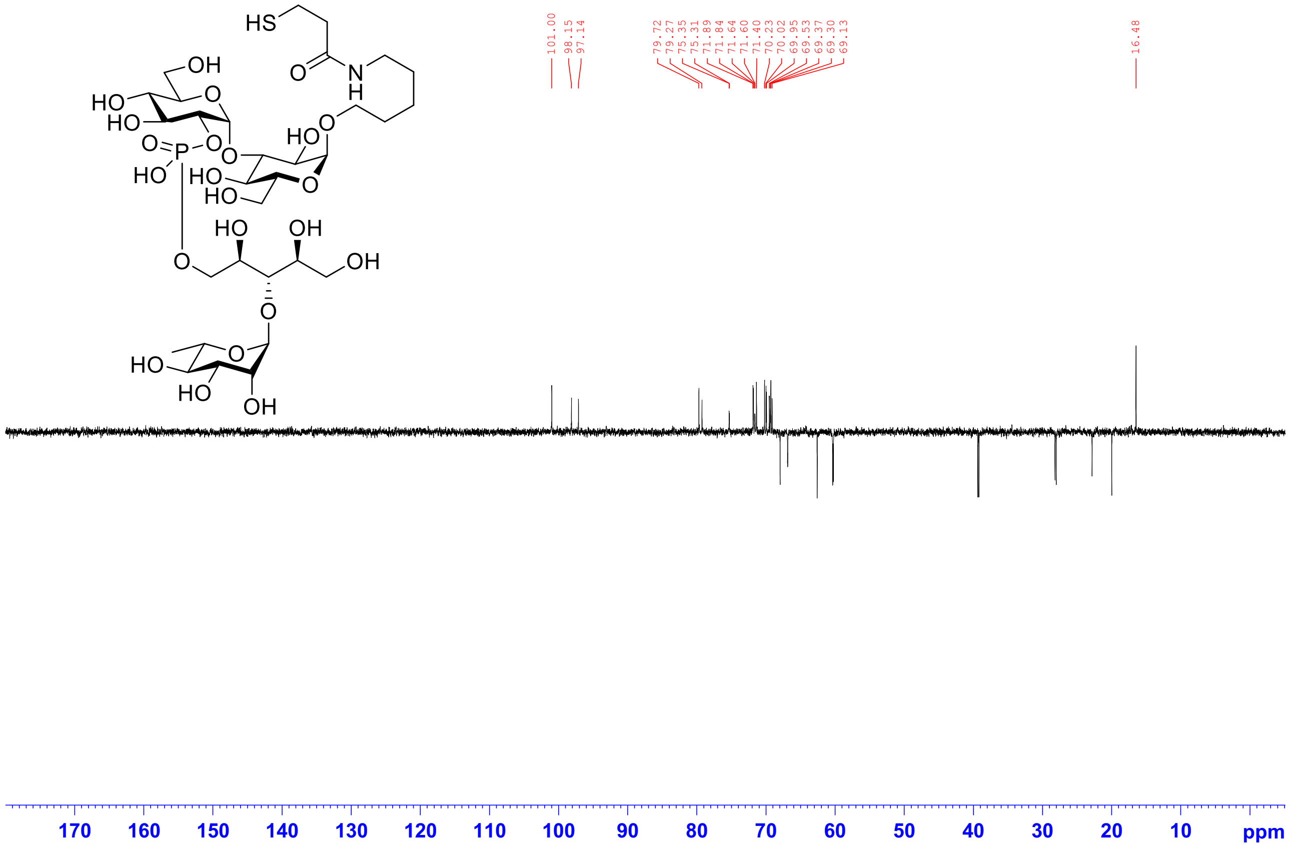

11-SH: ^{1}H NMR (600 MHz, D_2O)

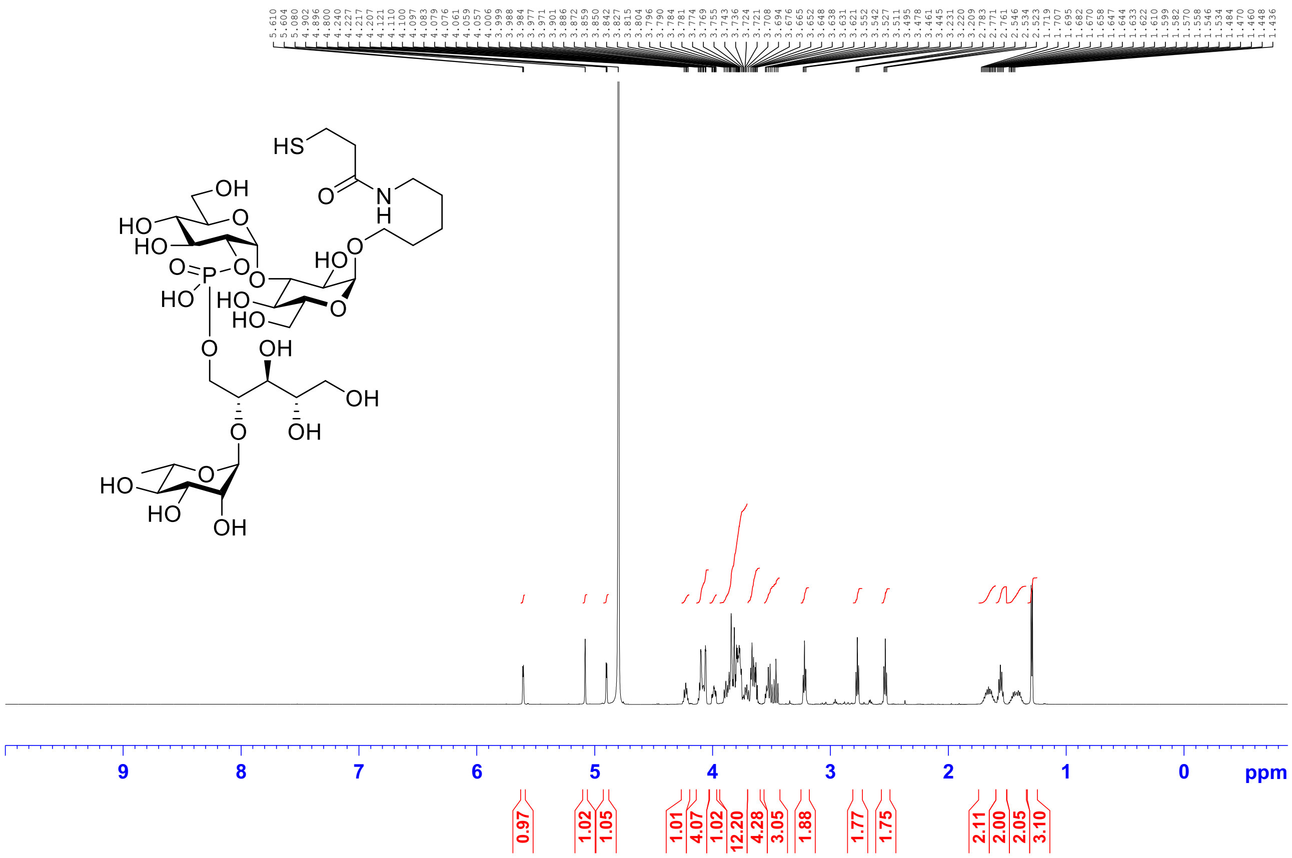

11-SH: ^{13}C NMR (150 MHz, D_2O)

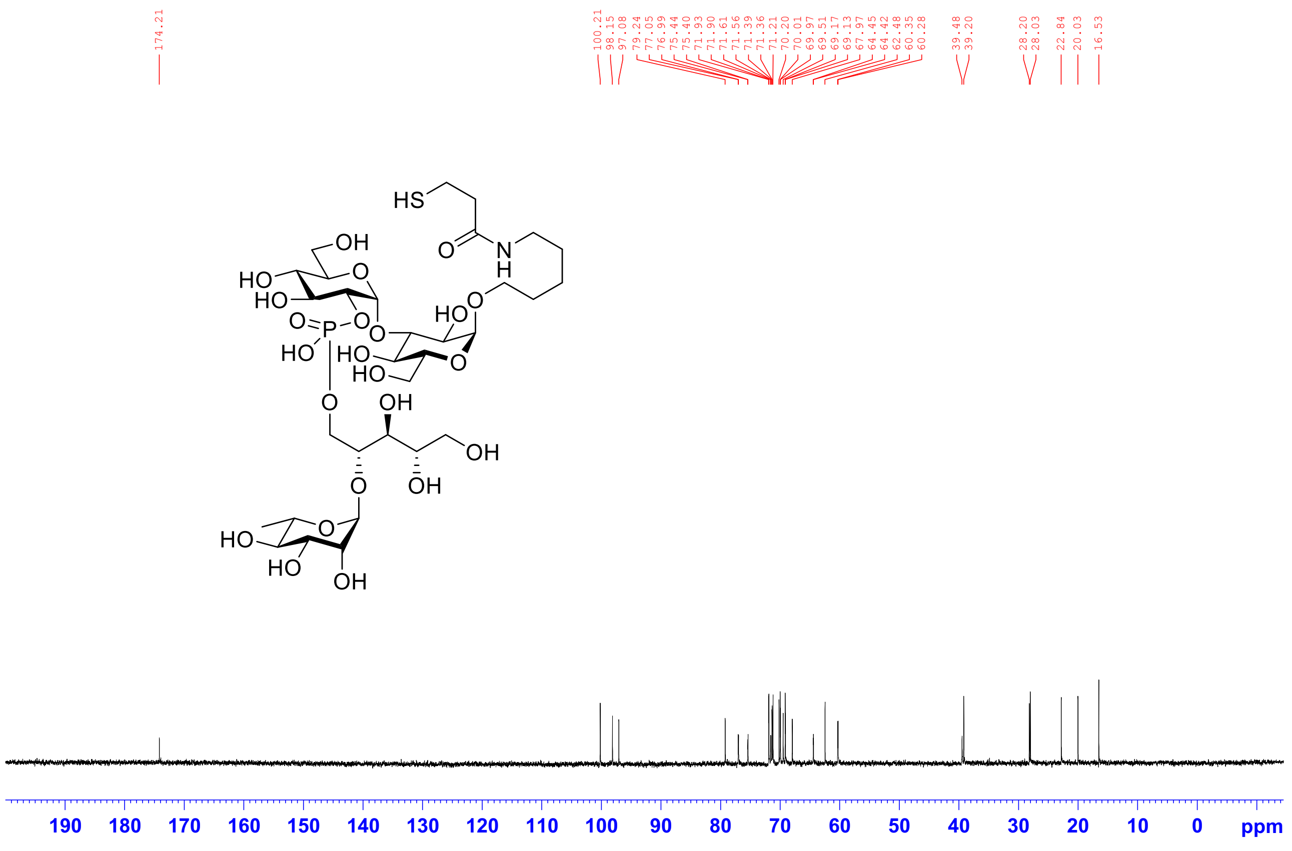

11-SH: DEPT 135 (150 MHz, D_2O)

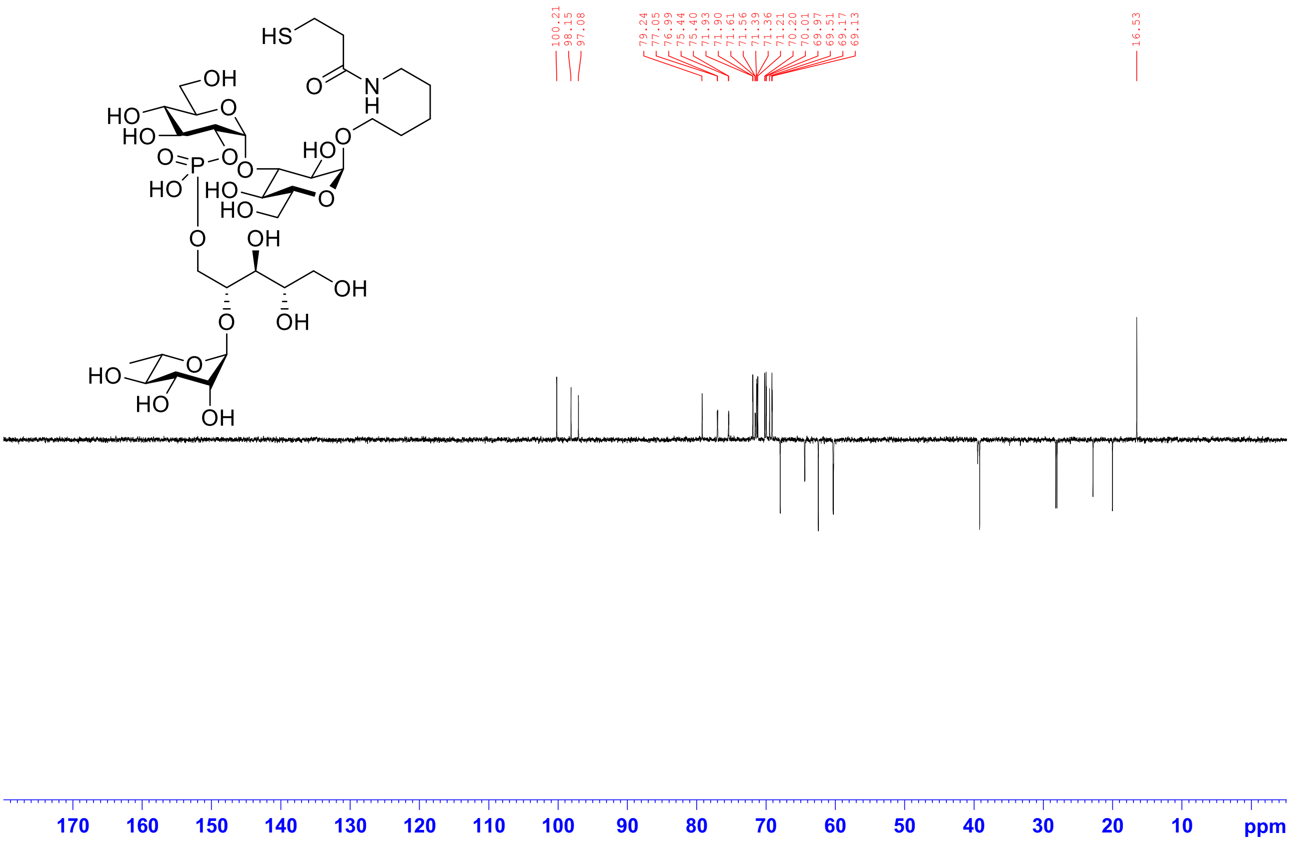

Supplement: Supplementary file 2 — id4c00147_si_002.pdf [file id4c00147_si_002.pdf]
