# Supplementary material for: Chemical Synthesis of Truncated Capsular Oligosaccharide of Serotypes 6C and 6D of Streptococcus pneumoniae with Their Immunological Studies
Source: ACS Infect Dis. 2024 May 21;10(6):2161–71. doi: 10.1021/acsinfecdis.4c00147 (PMC11184553; doi:10.1021/acsinfecdis.4c00147)
Supplement: Supplementary file 3 — id4c00147_si_003.pdf [file id4c00147_si_003.pdf]

Supporting Information – 3

Chemical Synthesis of Truncated Capsular Oligosaccharide of Serotypes 6C and 6D of *Streptococcus pneumoniae* with Their Immunological Studies

Ravinder Mettu,[1 #] Yang-Yu Cheng,[1,2 #] Hanmanth Reddy Vulupala,[1] Yu-Hsuan Lih,[1] Chiang-Yun Chen,[1] Mei-Hua Hsu,[3] Hong-Jay Lo,[1] Kuo-Shiang Liao,[1] Cheng-Hsun Chiu,[3] and Chung-Yi Wu[1]*

[1]Genomics Research Center, Academia Sinica, 128 Academia Road, Section 2, Nankang, Taipei, 11529 Taiwan

[2]Institute of Biochemistry and Molecular Biology, National Yang Ming Chiao Tung University, No. 155, Sec. 2, Linong St., Taipei 112304, Taiwan

[3]Molecular Infectious Disease Research Center, Chang Gung Memorial Hospital, Chang Gung University College of Medicine, 259 Wenhua 1st Road, Guishan, Taoyuan 33302, Taiwan

[#] These authors contributed equally.

List of Figures

Figure S3-S1. MALDI-TOF data of glycoconjugates **C1**–**C3**. S3
Figure S3-S2. MALDI-TOF data of glycoconjugates **D1**–**D3**. S4

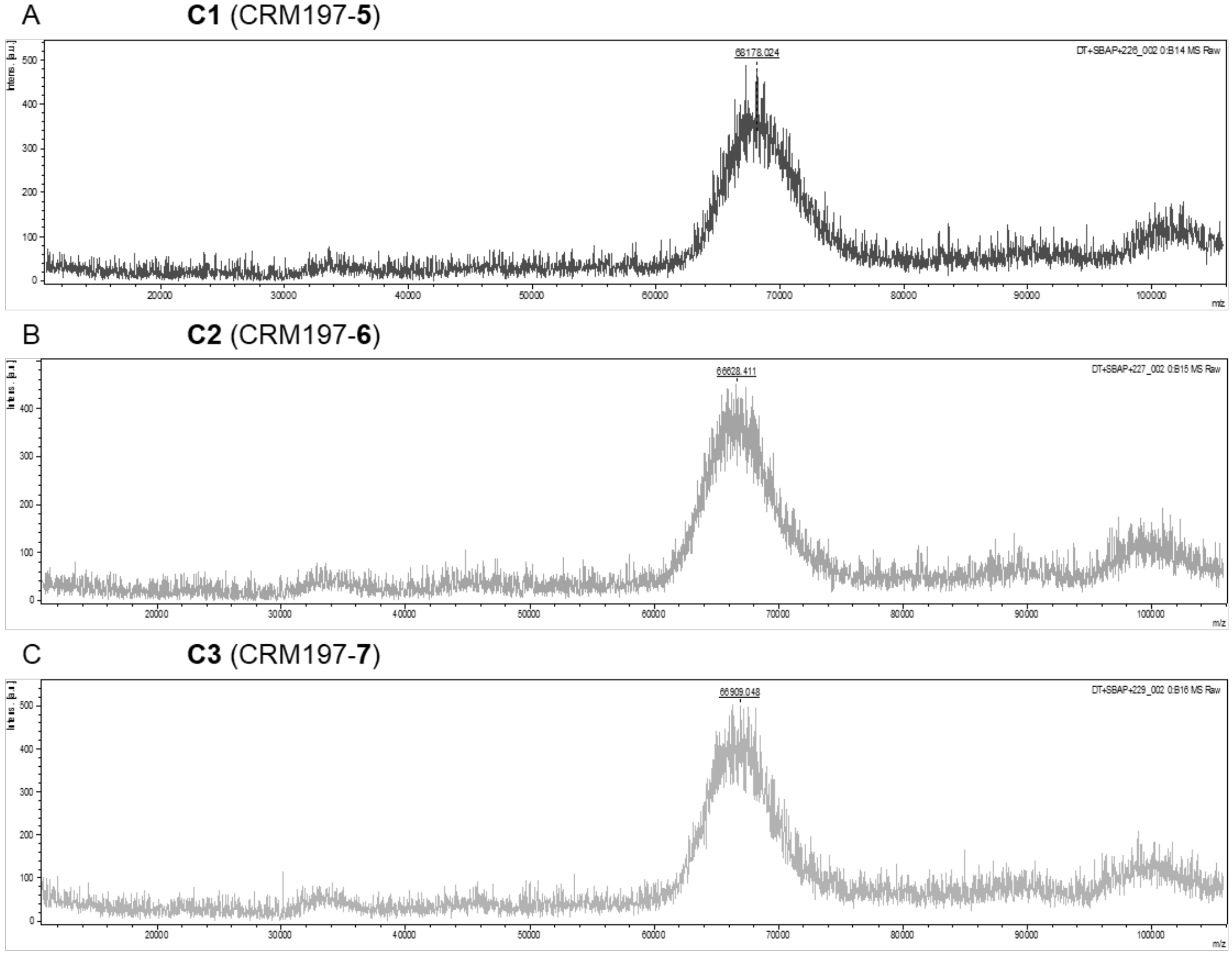

Figure S3-S1. MALDI-TOF data of glycoconjugates **C1**–**C3**.

A **D1** (CRM197-**9**)

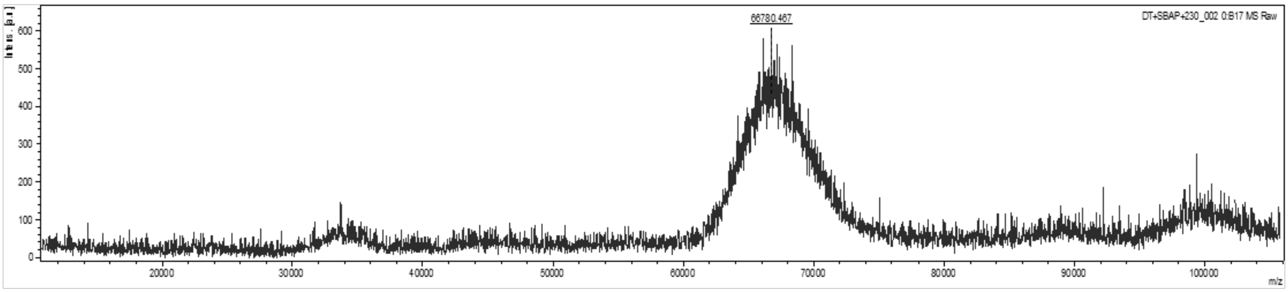

B **D2** (CRM197-**10**)

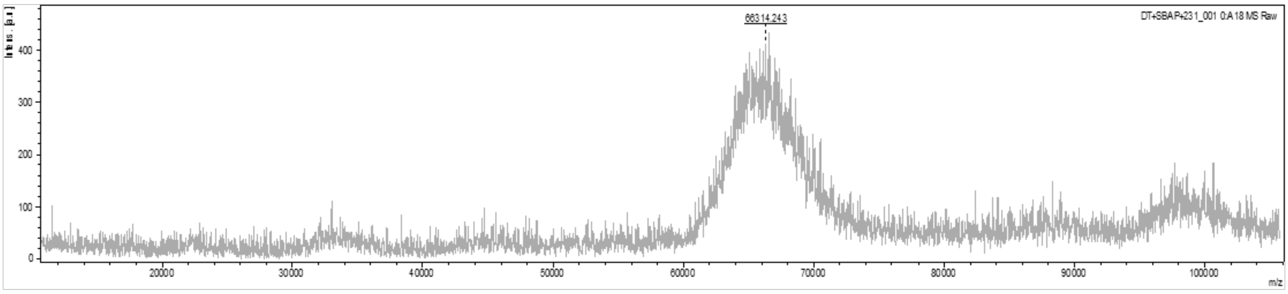

C **D3** (CRM197-**11**)

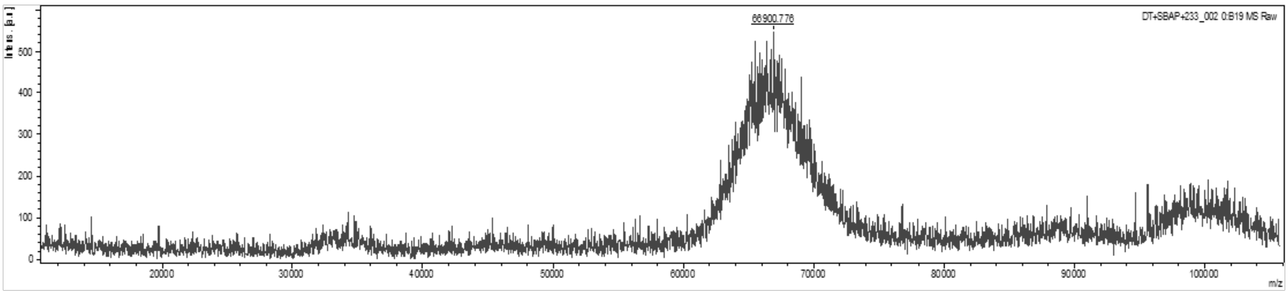

Figure S3-S2. MALDI-TOF data of glycoconjugates **D1**–**D3**.
